# Pitolisant 40 mg for excessive daytime sleepiness in obstructive sleep apnea patients treated or not by CPAP: Randomised phase 3 study

**DOI:** 10.1111/jsr.14373

**Published:** 2024-10-08

**Authors:** Yves Dauvilliers, Sonya Elizabeth Craig, Maria R. Bonsignore, Ferran Barbé, Johan Verbraecken, Jerryl Asin, Ognian Georgiev, Rumen Tiholov, Christian Caussé, Jeanne‐Marie Lecomte, Jean‐Charles Schwartz, Philippe Lehert, Winfried Randerath, Jean‐Louis Pépin, the HAROSA III Study Group, Dejan Dokic, Hristo Metev, Iliev Kartev, Zdravka Karamyan, Ventsislav Nozharov, Daniel Petkov

**Affiliations:** ^1^ Centre National de Référence Narcolepsie, Unité du Sommeil, CHU Montpellier, Hôpital Gui‐de‐Chauliac Service de Neurologie, Université de Montpellier, INSERM INM Montpellier France; ^2^ Liverpool Sleep and Ventilation Centre, University Hospital Aintree Liverpool University Foundation Trust Liverpool UK; ^3^ PROMISE Department University of Palermo, Institute for Biomedical Research and Innovation (IRIB), National Research Council (CNR) Palermo Italy; ^4^ Respiratory Department Institut Ricerca Biomedica de Vilanova Lleida Spain; ^5^ Multidisciplinary Sleep Disorders Centre Antwerp University Hospital and University of Antwerp Edegem‐Antwerp Belgium; ^6^ Department of Pulmonary Medicine, Centre for Sleep Medicine Amphia Hospital Breda The Netherlands; ^7^ Department of Internal Medicine, Pulmonology Alexandrovska Hospital Medical University Sofia Bulgaria; ^8^ Department of Internal Diseases Sv. Ivan Rilski Multiprofile Hospital for Active Treatment Kozloduy Bulgaria; ^9^ Bioprojet Pharma Paris France; ^10^ Faculty of Economics University Louvain Ottignies‐Louvain‐la‐Neuve Belgium; ^11^ Faculty of Medicine University of Melbourne Melbourne Victoria Australia; ^12^ Bethanien Hospital, Institute of Pneumonology University of Cologne Solingen Germany; ^13^ HP2 Laboratory INSERM U1300 University Grenoble Alpes Grenoble France

**Keywords:** continuous positive airway pressure, excessive daytime sleepiness, obstructive sleep apnea, pitolisant

## Abstract

Obstructive sleep apnea (OSA) syndrome commonly leads to excessive daytime sleepiness (EDS). Pitolisant, a selective histamine‐3 receptor antagonist, is efficacious at doses up to 20 mg once daily in OSA treated or not with continuous positive airway pressure (CPAP). We assessed the efficacy and safety of pitolisant at doses up to 40 mg once daily in patients with moderate to severe OSA treated or not with CPAP therapy. In this phase 3, multicentre, randomised, double‐blind, placebo‐controlled clinical trial, patients with OSA were assigned 2:1 to receive pitolisant (according to an individual up‐titration scheme, 10, 20 or 40 mg once daily) or placebo for 12 weeks. The primary endpoint was a change in the Epworth Sleepiness Scale (ESS) score from baseline to week 12. Secondary endpoints included a change in reaction time using the Oxford Sleep Resistance test (OSleR), Clinical Global Impression of Change (CGI‐C), and Patient's Global Opinion of the Effect (PGOE) of study treatment. Overall, 361 patients (mean age 52.4 years, 77.3% male; mean apnea‐hypopnea index [AHI] 27.0 events/h) were randomised to receive pitolisant (*n* = 242; 50% received CPAP) or placebo (*n* = 119; 48.7% CPAP). After the dose‐adjustment phase (week 3), 88.8% of patients received pitolisant 40 mg. Compared with placebo, pitolisant produced a significant reduction in the ESS score at week 12 (least square mean difference −2.6 (95% CI: −3.4; −1.8; *p* < 0.001)) irrespective of CPAP use; and improved the reaction time on OSleR, CGI‐C, and PGOE at week 12. Pitolisant was well tolerated; no new safety signals were identified. In conclusion, pitolisant up to 40 mg once daily was an effective treatment for EDS in patients with moderate to severe OSA irrespective of CPAP use.

## 1 INTRODUCTION

Obstructive sleep apnea (OSA) syndrome is the most frequent sleep‐related breathing disorder, affecting nearly one billion people worldwide (Benjafield et al., 2019; Lévy et al., 2015). OSA is the repetitive occurrence of partial (hypopneas) or complete (apneas) pharyngeal collapses, inducing intermittent hypoxia and sleep fragmentation which commonly leads to excessive daytime sleepiness (EDS). Patients with OSA may experience serious consequences of daytime hypersomnolence in their everyday life, such as deficits in attention or reaction time (Lal et al., 2022; Rosenzweig et al., 2015) with an increased risk of a road (McNicholas & Philip, 2023) or work‐related accident, impaired short‐term memory, and reduced capacity to sustain concentration or focus (Rosenzweig et al., 2015). The proposed mechanisms for EDS in OSA patients are sleep disturbances with alterations in sleep architecture and continuity, microarousals inducing sleep fragmentation, and brain hypoxic insult generated by intermittent hypoxia (Lal et al., 2021).

Continuous positive airway pressure (CPAP) is the first‐line treatment option for patients with moderate to severe OSA (Lévy et al., 2015). In most patients, optimised CPAP primary therapy normalises the apnea‐hypopnea index (AHI) and arterial blood oxygen saturation (SaO_2_), decreases sleep fragmentation, improves sleep quality, and can improve alertness, mood, cognitive function, and quality of life (QoL) (Labarca et al., 2020; Li et al., 2022). However, approximately 5%–10% of patients with OSA experience residual EDS, despite regular use of CPAP treatment (Gasa et al., 2013; Lal et al., 2021; Pépin et al., 2009). Furthermore, long‐term adherence to CPAP therapy is difficult to maintain, with nearly 50% of OSA patients terminating CPAP treatment 3 years after initiation (Pépin et al., 2021).

After a diagnosis of residual EDS and altered daily function associated with OSA has been confirmed, and other contributing conditions have been ruled out, pharmacotherapy with a wake‐promoting agent or stimulant can be considered (Rosenberg et al., 2021). Pitolisant is a selective histamine‐3 receptor antagonist/inverse agonist that is approved for the treatment of EDS in narcolepsy with or without cataplexy (Bassetti et al., 2021). In phase 3 narcolepsy clinical trials, pitolisant at doses up to 40 mg was well tolerated and effective for the treatment of patients with narcolepsy (Dauvilliers et al., 2013; Dauvilliers et al., 2019; Szakacs et al., 2017). In two phase 3 randomised controlled trials of OSA patients with EDS (HAROSA I and HAROSA II), pitolisant produced a significant decrease in the Epworth Sleepiness Scale (ESS) score compared with placebo (Dauvilliers et al., 2020; Pépin, Georgiev, et al., 2021). The HAROSA I and HAROSA II study populations differed, with patients adherent to CPAP in the HAROSA I trial (Pépin, Georgiev, et al., 2021), and patients not tolerant to or refusing CPAP in the HAROSA II trial (Dauvilliers et al., 2020). In these two studies, the maximum dose of pitolisant was 20 mg once daily. Clinical data regarding pitolisant 40 mg once daily in OSA are lacking. It was hypothesised that OSA patients who partially responded to pitolisant at the 20 mg dose in the HAROSA I and II trials may benefit from a higher dose, as shown for narcolepsy patients (Dauvilliers et al., 2019). The safety results in HAROSA I and II were also reassuring for cardiovascular risk factors and diseases in patients with OSA. This good safety profile of pitolisant merits confirmation at higher doses. Therefore, this large multicentre, randomised controlled trial (HAROSA III) aimed to assess the efficacy and safety of pitolisant, at doses up to 40 mg once daily according to an individual up‐titration scheme, in patients with OSA adherent to CPAP therapy or intolerant to or refusing CPAP.

## 2 METHODS

### 2.1 Trial design

This phase 3, multicentre, randomised, double‐blind, placebo‐controlled clinical trial (ClinicalTrials.gov NCT02739568) evaluated the efficacy and safety of pitolisant at doses up to 40 mg according to an individual up‐titration scheme in OSA patients with EDS treated or not by CPAP primary therapy (Figure S1). The study was conducted in seven centres in Bulgaria and North Macedonia. All questionnaires were translated by a specialised company: GSI Translation (Skopje, Macedonia) for Macedonian and BalkanTrials (Sofia, Bulgaria) for Bulgarian. Following an initial 2 week screening period, patients were randomised to receive pitolisant once daily or placebo for 12 weeks. Pitolisant doses were escalated during the first 2 weeks (from 10 to 40 mg, 1 week per dose), adjusted (if necessary, based on efficacy and tolerability according to the physician decision during clinical visits) during the third week and maintained at a stable dose for the following 9 weeks.

### 2.2 Patients

Patients were adults aged 18 years or older diagnosed with OSA according to the International Classification of Sleep Disorders (2nd Edition) criteria (American Academy of Sleep Medicine, 2005), experiencing EDS with an ESS score (Johns, 1991) ≥ 12, and an AHI on diagnosis by polysomnography ≥15/hour of sleep. Full inclusion and exclusion criteria are detailed in Table S1. Specifically, patients were excluded from the study if they presented with any other aetiology of sleepiness (e.g. severe insomnia, narcolepsy, restless leg syndrome), any consumption of hypnotics, or alcohol or other substance abuse or dependance.

### 2.3 Randomisation and masking procedures

Randomisation was centralised and performed via an electronic web randomisation server (Arone Projection; https://www.bioprojet-studies.org/) that automatically assigned a patient number at screening and then automatically assigned a study treatment number when the patient was randomised. The randomisation list was established on a balanced 2:1 (pitolisant: placebo) basis. Randomisation was stratified by CPAP therapy (i.e. adherent or intolerant to CPAP therapy). Study treatments, taken in the morning during breakfast with a glass of water, were provided in sealed capsules that were similar in appearance and taste, containing pitolisant 5 mg (2 tablets: 10 mg dose) or 20 mg (1 tablet: 20 mg dose; 2 tablets: 40 mg dose) or matching placebo. The patients, their sleep and/or respiratory physicians, and staff were blinded to treatment allocation.

### 2.4 Outcomes

The primary efficacy endpoint was a change in ESS score from baseline to the end of treatment at week 12 (end of the double‐blind period).

The key secondary efficacy endpoint was change in reaction time using the OSleR test (Bennett et al., 1997). The OSleR test consisted of three sessions each of 40 minute sleep resistance challenges performed at 9:00 a.m., 11:00 a.m., and 1:00 p.m. The mean sleep latency (OSL; mean of the three tests) and OSleR test success (OSLC; normal reaction time defined by the absence of three to six consecutive errors [indicating microsleep] and seven or more errors [indicating sleep onset], for each of the three tests) were calculated (Bennett et al., 1997; Mazza et al., 2002). Other secondary endpoints were a change in the Clinical Global Impression of Change (CGI‐C) assessed on a 7‐point Likert scale (from very much improved to very much worse), and the Patient's Global Opinion of the Effect (PGOE) of study treatment rated on a 6‐point Likert scale (marked, moderate or minimal effect; no change; minimal effect or much worse effect).

Exploratory efficacy endpoints included the assessment of QoL using the EuroQol‐5D (EQ‐5D) questionnaire, assessment of fatigue using the Pichot Fatigue Scale (Pichot & Brun, 1984), assessment of sleep quality using the Leeds Sleep Evaluation Questionnaire (LSEQ); aggregated ESS and OSleR Z‐scores; therapy response, assessed by (1) an ESS value ≤10, and (2) an ESS value ≤10 or a decrease of ESS by ≥3; and parts A and B of the Trail Making Test (TMT).

Safety was assessed by the incidence of adverse events (AEs) and treatment‐emergent AEs (TEAEs) which were classified by system organ class and preferred term using MedDRA version 19.1. Clinical laboratory parameters (haematology, biochemistry, and electrolytes), vital signs, physical examination, ECG data, Beck Depression Inventory 13‐item (BDI‐13) score, and the patient's overall evaluation of tolerance were also evaluated. Treatment adherence was estimated by asking the patient whether the investigational treatment was taken as prescribed, and by counting remaining tablets of study treatment. Any discrepancies were investigated by discussion with the patient.

### 2.5 Ethical approval

The study was conducted in accordance with the ethical principles of the Declaration of Helsinki (2008), and the International Council for Harmonization of Technical Requirements for Pharmaceuticals for Human Use (ICH) Good Clinical Practice (GCP) guidelines and followed all other local requirements. The study was approved by the Independent Ethics Committees of participating centres and relevant competent authorities in accordance with the requirements of each country.

Written informed consent was obtained from each patient (or patient's legally authorised representative) prior to enrolment.

### 2.6 Statistical analyses

#### 2.6.1 Sample size calculation

The results from exploratory studies of pitolisant estimated ESS residual variability with a standard deviation (SD) = 6. The minimum clinically important difference was fixed to an ESS = 3 (Crook et al., 2019; Patel et al., 2018), corresponding to an effect size of 0.5. The correlation between final and baseline ESS was conservatively estimated as *r* = 0.3. Assuming an analysis of covariance (ANCOVA) at a 0.95 confidence level as the main confirmatory test, a difference of at least three points would be detected with a power of 90%, by including at least 60 patients in each pitolisant treatment group (stratification: 50% with/50% without primary CPAP therapy; 120 in total) and at least 30 patients in each placebo group (60 in total). Applying the same model, a difference of at least three points between CPAP‐treated and non‐CPAP‐treated would be detected with a power of 90%, when 240 and 120 patients (360 patients in total) were included in the pitolisant and placebo groups, respectively. Assuming a 10% drop‐out rate, a total of 400 patients would need to be selected prior to randomisation.

#### 2.6.2 Populations and variables

The full analysis set (FAS) included all randomised patients. The per protocol (PP) population was defined as all patients in the FAS without any major protocol deviations. Analyses on the PP population were not performed if the sample size was ≥95% of the FAS population. The safety population included all patients who received at least one dose of study treatment and had at least one valid post‐baseline evaluation.

For continuous variables, the mean, 95% confidence interval (CI) of the mean, SD, minimum, first quartile, median, third quartile, maximum, and number of available and missing observations were recorded. For categorical variables, the number and percentage were recorded. Missing data for the primary efficacy outcome and secondary therapeutic responses were imputed using the last observation carried forward method.

The significance of the difference between pitolisant versus placebo was assessed using a mixed‐model ANCOVA. Independent variables were treatment as a fixed effect, study centre as a random effect, and the baseline value of the endpoint as a fixed covariate. For sensitivity purposes, two supportive models were used based on the ANCOVA model (Model 1). Model 2 included additional adjustments for CPAP use and Model 3 included CPAP use and treatment‐CPAP interaction. All statistical tests were two‐sided at a 5% level of significance. Analyses were performed using SAS version 9.4 statistical software package. The study protocol (eSAP 1) and statistical analysis plan (eSAP 2) are available for reference.

## 3 RESULTS

### 3.1 Patient demographics and baseline clinical characteristics

**FIGURE 1:**
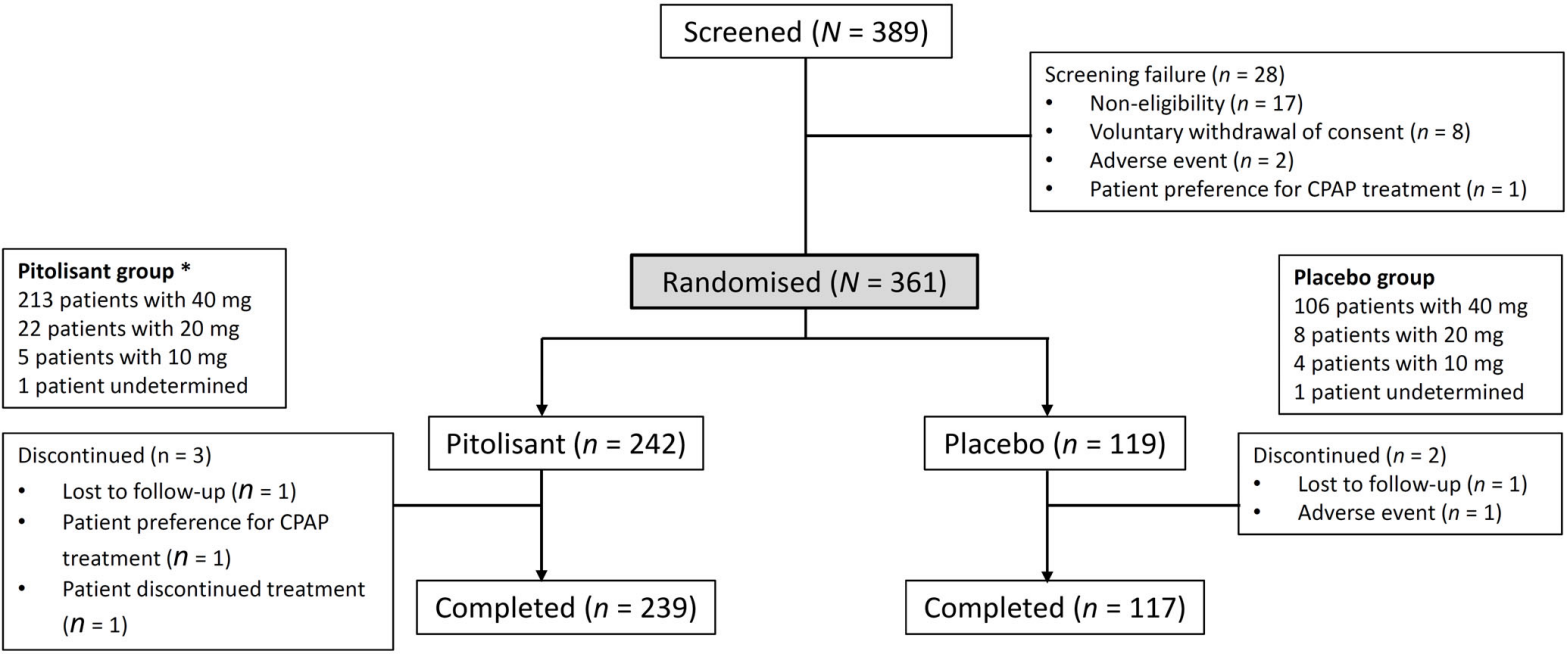
CONSORT Diagram: Patient disposition. *In the pitolisant group, 241 patients took at least one tablet of pitolisant (safety population). CPAP, continuous positive airway pressure.

A total of 389 patients were screened, and 361 were randomised between 7 April 2016 and 24 April 2019, to receive pitolisant (*n* = 242) or placebo (*n* = 119), which represented the FAS. The study flow is depicted in Figure 1. Most patients (98.6%) completed the double‐blind phase of the study (week 12). At the endpoint visit, most patients received 40 mg once daily and a few received 20 mg or 10 mg (88.8%; 9.2%; 2% [pitolisant] versus 89.8%; 6.8%; 3.4% [placebo], respectively); thus, this study mainly reflects the efficacy and safety of pitolisant 40 mg once daily.

The FAS population was a typical OSA population with a mean (SD) age of 52.4 (10.1) years and 77.3% were male (Table 1). Most patients were aged <65 years in the pitolisant (88.8%) and placebo (89.9%) treatment groups. At baseline, according to the stratification process, half of patients in the pitolisant group (50%; *n* = 121) and 48.7% of patients (*n* = 58) in the placebo group were treated with and adherent to CPAP. Mean (SD) time since OSA diagnosis was 35.9 (31.7) months and was similar between treatment groups and in subgroups stratified by CPAP use. Nearly two‐thirds of patients had comorbid cardiovascular diseases (64.8%) and were receiving concomitant medications for the cardiovascular system (66.2%).

**TABLE 1:** Demographics and clinical characteristics of the full analysis set population.

Characteristic	Pitolisant (*n* = 242)	Placebo (*n* = 119)	Total (*n* = 361)
Age (years), mean (SD)	52.3 (9.8)	52.6 (10.6)	52.4 (10.1)
Male/Female, *n* (%)	176 (72.7)/66 (27.3)	90 (75.6)/29 (24.4)	266 (73.7)/95 (26.3)
Time since OSA diagnosis (months), mean (SD)	36.0 (34.2)	35.6 (25.9)	35.9 (31.7)
Body mass index (kg/m^2^), mean (SD)	34.9 (4.0)	34.7 (4.4)	34.9 (4.2)
History of cardiovascular disease, *n* (%)	160 (66.1)	74 (62.2)	234 (64.8)
Concomitant medication, *n* (%)	183 (75.9)	87 (73.1)	270 (74.8)
Cardiovascular system medication^a^	163 (67.6)	76 (63.9)	239 (66.2)
Renin‐angiotensin system agents	135 (56.0)	54 (45.4)	189 (52.4)
Beta‐blockers	83 (34.4)	35 (29.4)	118 (32.7)
Diuretics	54 (22.4)	26 (21.8)	80 (22.2)
Calcium‐channel blockers	35 (14.5)	23 (19.3)	58 (16.1)
Lipid‐modifying agents	35 (14.5)	10 (8.4)	45 (12.5)
Receiving CPAP therapy, *n* (%)	121 (50.0)	58 (48.7)	179 (49.6)
Duration of sleep (hours), mean (SD)	7.25 (1.10)	7.11 (0.91)	7.20 (1.04)
Polysomnography parameters, mean (SD)			
Total sleep time (min)^b^	338.7 (107.0)	335.9 (102.9)	337.8 (105.5)
Sleep latency (min)^c^	20.4 (21.9)	23.9 (57.1)	21.6 (37.7)
Duration of wakefulness after sleep onset (min)^b^	58.2 (46.4)	66.4 (59.9)	61.0 (51.5)
Total sleep time with SaO_2_ < 90% (min)^d^	68.4 (98.5)	74.3 (95.1)	70.4 (97.3)
Mean nocturnal SaO_2_ (%)	91.0 (9.3)	91.1 (5.3)	91.0 (8.2)
Diagnosis AHI (events/h)	25.9 (27.2)	29.2 (29.3)	27.0 (27.9)
Respiratory micro‐arousal index	13.6 (20.3)	14.6 (21.3)	13.9 (20.6)
Non‐respiratory micro‐arousal index	11.6 (11.3)	12.2 (12.1)	11.8 (11.6)
Index of periodic limb movement	6.6 (9.7)	6.2 (11.2)	6.5 (10.2)

Abbreviations: AHI, apnea–hypopnea index; CPAP, continuous positive airway pressure; OSA, obstructive sleep apnea; SaO_2_, O_2_ saturation.

^a^ Reported in ≥10% of patients in any treatment group.

^b^ Pitolisant (*n* = 230), Placebo (*n* = 118), Total (*n* = 348).

^c^ Pitolisant (*n* = 229), Placebo (*n* = 118), Total (*n* = 347).

^d^ Pitolisant (*n* = 234), Placebo (*n* = 117), Total (*n* = 351).

At the baseline diagnosis evaluation, patients exhibited moderate to severe OSA with an overall mean (SD) AHI of 27.0 (27.9) events/hour (Table 1). For patients receiving CPAP (*n* = 179), median (range) CPAP pressure in the pitolisant (*n* = 121) and placebo (*n* = 58) groups was 12.0 (5.0–17.0) and 12.0 (4.0–14.0) cm H_2_O, respectively, and mean (SD) nocturnal SaO_2_ was 92.9% (4.4) and 92.7% (3.6), respectively.

The PP population comprised 355 patients in total (pitolisant, *n* = 240; placebo, *n* = 115). As the sample size was ≥95% of the FAS population (98.3%), no analyses were performed (as outlined in the statistical analysis plan). The safety population consisted of 360 patients who received at least one dose of pitolisant (*n* = 241) or placebo (*n* = 119) and who had at least one valid post‐baseline evaluation.

### 3.2 Primary efficacy endpoint

**FIGURE 2:**
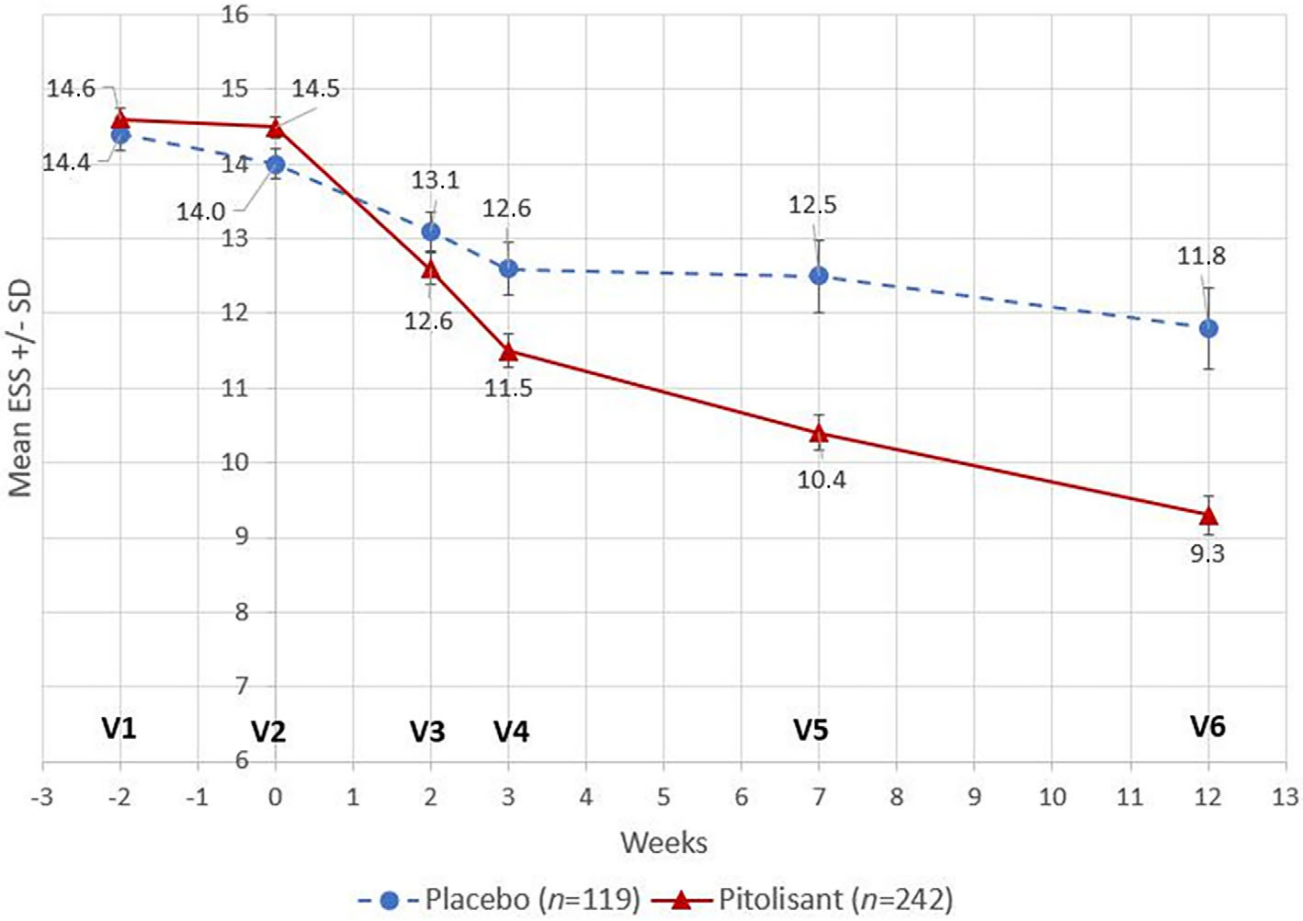
Mean ESS total scores by treatment at each assessment time in the double‐blind period – FAS (*N* = 361). ESS, Epworth Sleepiness Scale; FAS, full analysis set; *N*, number of patients in the FAS; *n*, number of patients in each group; SD, standard deviation; V, visit.

The mean ESS total score decreased from baseline to week 12 by −4.66 (95% CI: −5.05; −4.27) in the pitolisant group and by −1.83 (95% CI: −2.84; −0.82) in the placebo group; the least square mean (LSM) difference was −2.6 (95% CI: −3.4; −1.8; *p* < 0.001) (Table 2; Figure 2). This result was confirmed by sensitivity analyses which showed no significant effect of being treated or not with CPAP primary therapy. In patients with CPAP use, the mean (95% CI) changes were −5.00 (−5.54; −4.46) with pitolisant compared to −1.89 (−3.49; −0.28) with placebo. In patients with no CPAP use, the mean (95% CI) respective changes were −4.31 (−4.87; −3.75) and −1.78 (−3.08; −0.48). No interactions were found between the treatment (pitolisant vs. placebo) effect on ESS baseline, AHI, and CPAP use. The treatment effect was significant for all BMI categories but was higher for patients with a lower BMI. In addition, no treatment effect was found for both random and fixed effects of centres or countries studied, and a non‐significant interaction was found between centre and treatment.

**TABLE 2:** Efficacy results (full analysis set).

Parameter	Pitolisant (*n* = 242)	Placebo (*n* = 119)	*p*‐value
Epworth Sleepiness Scale (ESS)			
ESS score at baseline, mean (SD)	14.5 (2.2)	14.0 (2.2)	–
ESS score at end of treatment, mean (SD)	9.3 (4.1)	11.8 (5.9)	–
Final ESS score, DB‐LOCF, mean (95% CI)	9.86 (9.41; 10.32)	12.18 (11.18; 13.17)	<0.001
ESS score change, DB‐LOCF–baseline, mean (95% CI)	−4.66 (−5.05; −4.27)	−1.83 (−2.84; −0.82)	<0.001
Therapy response 1^a^: ESS ≤10, *n* (%) [95% CI]	125 (51.7) [45.2; 58.1]	45 (37.8) [29.1; 47.2]	<0.0001
Therapy response 2^a^: ESS ≤10 or decrease of ESS by ≥3, *n* (%) [95% CI]	172 (71.1) [64.9; 76.7]	67 (56.3) [46.9; 65.4]	0.0063
OSleR test			
OSL at baseline, geometric mean, minutes	17.45	18.48	–
OSL at week 12, geometric mean, minutes	22.74	19.09	–
Ratio of OSL at week 12/OSL at baseline, geometric mean	1.291	1.034	<0.0001
Patients with OSLC at baseline, *n* (%) [95% CI]	31 (12.8) [8.9; 17.7]	13 (10.9) [5.9; 18.0]	–
Patients with OSLC at week 12, *n* (%) [95% CI]	47 (19.7) [14.8; 25.3]	19 (16.2) [10.1; 24.2]	0.1652
Clinical Global Impression of Change (CGI‐C), n (%)			
Very much improved	34 (14.2)	5 (4.3)	–
Much improved	112 (46.9)	49 (41.9)	–
Minimally improved	75 (31.4)	38 (32.5)	–
No change	18 (7.5)	12 (10.3)	–
Minimally worse	0	11 (9.4)	–
Much worse	0	2 (1.7)	–
CGI‐C improvement at end of DB treatment			
*n* (%) [95% CI]	221 (92.5) [88.4; 95.5]	92 (78.6) [70.1; 85.7]	0.0003
Patient's Global Opinion of the Effect (PGOE)			
Marked effect	62 (25.9)	20 (17.1)	–
Moderate effect	97 (40.6)	52 (44.4)	–
Minimal effect	66 (27.6)	27 (23.1)	–
No change	14 (5.9)	10 (8.5)	–
Minimally worse	0	7 (6.0)	–
Much worse	0	1 (0.9)	–
PGOE improvement at end of DB treatment			
*n* (%) [95% CI]	225 (94.1) [90.4; 96.8]	99 (84.6) [76.8; 90.6]	0.0042
EQ‐5D, mean change in VAS score (95% CI)	6.1 (4.5; 7.7)	3.9 (1.7; 6.0)	NS
Pichot Fatigue Scale score, mean change (95% CI)	−2.2 (−2.8; −1.7)	−0.1 (−1.2; 1.0)	0.0004
Leeds Sleep Evaluation Questionnaire, mean (SD)			
Change in modified getting to sleep	−7.16 (16.74)	−6.31 (15.64)	NS
Change in quality of sleep	10.59 (21.77)	9.17 (19.01)	NS
Change in awake after sleep	12.73 (20.57)	11.03 (19.75)	NS
Change in behaviour after awakening	11.98 (18.54)	10.53 (15.81)	NS
Aggregated ESS and OSleR Z‐scores, mean (95% CI) change at week 12 from baseline	−2.1 (−2.3; −1.8)	−1.0 (−1.4; −0.5)	<0.0001
TMT A, mean change in average time (SD)	−4.9 (13.5)	−2.5 (12.7)	NS
TMT B, mean change in average time (SD)	−6.8 (21.4)	−5.0 (25.4)	NS

Abbreviations: CI, confidence interval; DB, double‐blind; DB‐LOCF, double‐blind with last observation carried forward; EQ‐5D, EuroQol five‐dimension quality of life scale; NS, not statistically significant; OSL, mean sleep latency; OSLC, OSleR success; OSleR, Oxford Sleep Resistance Test; TMT, Trail Making Test (evaluated in seconds); VAS, visual analog scale.

^a^ Exploratory efficacy endpoint.

### 3.3 Secondary efficacy endpoints

Analysis of OSleR results showed that pitolisant improved OSL from baseline to week 12 compared with placebo, with a higher geometric mean of the OSL ratio (baseline/week 12: 1.291 [17.45 to 22.74] vs. 1.034 [18.48 to 19.09]; LSM difference = 1.2, 95% CI: 1.1; 1.3; *p* < 0.0001). This improvement was observed irrespective of CPAP use (Table 2).

The proportion of patients treated with pitolisant versus placebo reporting improvement in CGI‐C at week 12 (92.5% vs. 78.6%) was higher with an odds ratio (OR) of 3.6 (95% CI: 1.8; 7.2; *p* = 0.0003), irrespective of CPAP use or non‐use (interaction test), *p* = 0.945; the risk ratio (pitolisant/placebo) and difference (pitolisant‐placebo) were 1.17 and 13.9%. Similarly, a higher proportion of patients treated with pitolisant reported PGOE improvement at week 12 compared with placebo (94.1% vs. 84.6%) with an OR of 3.071 (95% CI: 1.428; 6.604; *p* = 0.0042), irrespective of CPAP use or non‐use (interaction test, *p* = 0.787); risk ratio (pitolisant/placebo) and difference (pitolisant‐placebo) were 1.11 and 9.5%.

### 3.4 Exploratory efficacy endpoints

Pitolisant improved fatigue, assessed with the Pichot fatigue scale, at week 12 with a mean (95% CI) decrease from baseline of −2.2 (−2.8; −1.7), compared with −0.1 (−1.2; 1.0) for placebo (LSM difference was −1.8, 95% CI: −2.8; −0.8; *p* = 0.0004), with no difference for use or non‐use of CPAP (Table 2).

Pitolisant reduced the ESS and OSleR aggregated Z‐score at week 12 compared with placebo (LSM difference: −1.0; 95% CI: −1.4; −0.6; *p* < 0.0001), irrespective of CPAP use.

A higher proportion of patients treated with pitolisant versus placebo had a therapeutic response defined by (1) an ESS value ≤10, and (2) an ESS value ≤10 or a decrease of ESS by ≥3 (Table 2). The respective proportions of patients with ESS ≤10 were 51.7% vs. 37.8%, with an OR of 3.827 (95% CI: 2.014; 7.272; *p* < 0.0001); and the respective proportions of patients with ESS ≤10 or a decrease of ≥3 were 71.1% vs. 56.3%, with an OR of 2.064 (95% CI: 1.229; 3.466; *p* = 0.0063). When stratified by CPAP use, the proportion of patients with ESS ≤10 treated with pitolisant or placebo was 61.2% vs. 51.7%, respectively, for CPAP use and was 42.1% vs. 24.6%, respectively, without CPAP use (*p* = 0.0436).

Compared with placebo, no significant effects of pitolisant were observed on other exploratory endpoints related to quality of life (EQ‐5D and LSEQ) or cognitive function (TMT parts A and B).

### 3.5 Safety

The incidence of TEAEs was comparable between treatment arms with 78 TEAEs occurring in 54 patients (22.4%) in the pitolisant group and 40 events occurring in 30 patients (25.2%) in the placebo group. The most common TEAEs by system organ class were nervous system disorders reported in 16 (6.6%) and 11 patients (9.2%), respectively, followed by psychiatric disorders in 18 (7.5%) and 6 patients (5.0%), respectively. By preferred term, the most common TEAEs were headache occurring in 15 (6.2%) and 9 patients (7.6%), respectively, followed by insomnia in 12 (5.0%) and 3 patients (2.5%), respectively. The incidence of TEAEs of special interest (anxiety, depression, drug abuse and misuse, drug dependence, fertility disorders, gastric disorders caused by hyperactivity, insomnia, proconvulsive potential, QT interval prolongation, rebound effect, and weight increase) was twice that with pitolisant compared with placebo (7.5% vs. 3.4%), mainly attributable to insomnia (Table 3). Most TEAEs were of mild or moderate intensity. Two patients receiving pitolisant (0.8%) experienced severe TEAEs during the dose‐adjustment phase which were considered treatment‐related: one patient (with concomitant CPAP use) experienced insomnia and anxiety and the other patient (without CPAP use) experienced insomnia. The TEAEs led to dose reduction and were resolved. The incidence of treatment‐related TEAEs was similar between pitolisant (26 patients [10.8%]) and placebo (11 patients [9.2%]) groups. The most common treatment‐related TEAEs by system organ class were psychiatric disorders in 18 (7.5%) and 5 (4.2%) patients, respectively; and by preferred term were insomnia in 12 (5.0%) and 2 (1.7%) patients, respectively. One patient (0.4%) in the pitolisant group experienced a serious TEAE (COPD, moderate intensity; the patient had an ongoing medical history of COPD and was receiving CPAP), which was not considered treatment‐related and did not require dosage adjustment or study discontinuation. No deaths or cases of overdosing were reported.

**TABLE 3:** Treatment‐emergent adverse events (TEAEs) by system organ class (SOC) and preferred term (PT) in the safety population with incidence ≥5% by SOC in either treatment group.

TEAEs by SOC and PT	Pitolisant (*n* = 241)		Pitolisant 40 mg (*n* = 231)		Placebo (*n* = 119)	
	Events, *n*	Patients, *n* (%)	Events, *n*	Patients, *n* (%)	Events, *n*	Patients, *n* (%)
Any TEAE	78	54 (22.4)	59	40 (17.3)	40	30 (25.2)
≥1 treatment‐related TEAE	41	26 (10.8)	30	17 (7.4)	13	11 (9.2)
≥1 severe TEAE	3	2 (0.8)	3	2 (0.9)	0	0
≥1 treatment‐emergent SAE	1	1 (0.4)	1	1 (0.4)	0	0
≥1 TEAE leading to treatment discontinuation	0	0	0	0	1	1 (0.8)
≥1 TEAE of special interest	19	18 (7.5)	14	13 (5.6)	4	4 (3.4)
TEAEs (incidence ≥5% by SOC)						
Nervous system disorders	17	16 (6.6)	12	11 (4.8)	11	11 (9.2)
Headache	16	15 (6.2)	11	10 (4.3)	9	9 (7.6)
Psychiatric disorders	23	18 (7.5)	18	13 (5.6)	6	6 (5.0)
Insomnia	12	12 (5.0)	9	9 (3.9)	3	3 (2.5)
Anxiety	4	4 (1.7)	3	3 (1.3)	1	1 (0.8)
Nightmare	4	4 (1.7)	4	4 (1.7)	0	0

*Note*: TEAEs of special interest included anxiety, depression, drug abuse and misuse, drug dependence, fertility disorders, gastric disorders caused by hyperactivity, insomnia, proconvulsive potential, QT interval prolongation, rebound effect, and weight increase.

Most TEAEs, either treatment‐related or of special interest, were reported in the group receiving pitolisant 40 mg (Table 3); however, because 88.8% of patients received pitolisant 40 mg, a between‐group comparison with other doses is not possible. There were no significant effects of CPAP use on the incidence of TEAEs or treatment‐related TEAEs.

**FIGURE 3:**
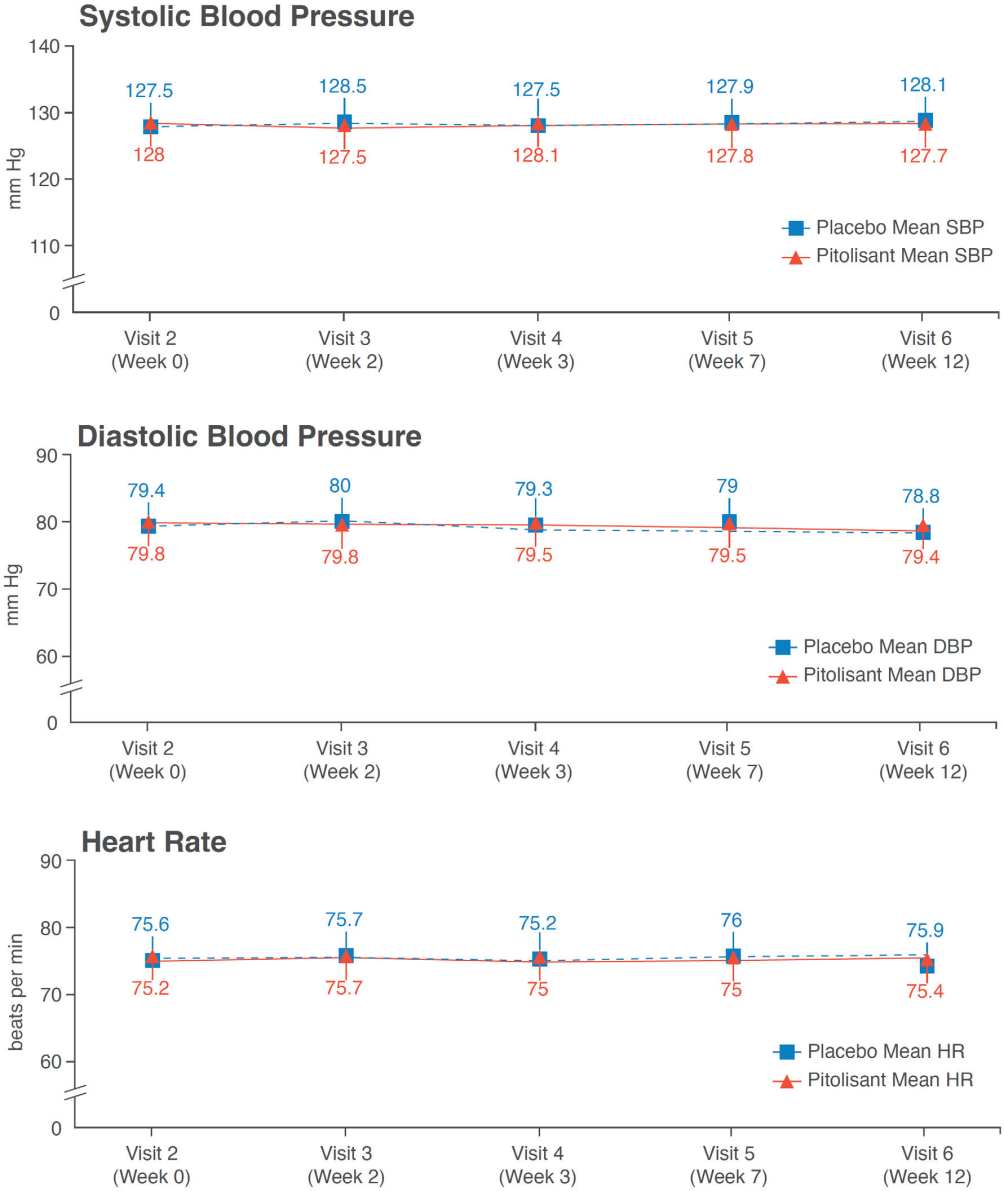
Line graph showing heart rate, systolic blood pressure, and diastolic blood pressure in the pitolisant and placebo groups over the 12 week double‐blind phase. DBP, diastolic blood pressure; HR, heart rate; SBP, systolic blood pressure.

TEAEs classified by system organ class as cardiac disorders were reported in one male patient (0.4%) in the pitolisant group and two male patients (1.7%) in the placebo group, all patients were in the 'non‐CPAP' subgroup. No significant effects of treatment were found on systolic and diastolic blood pressure (BP), heart rate (HR) (Figure 3), PR interval, QRS interval, QT interval, and Bazett‐corrected QT interval (QTcB) (Table 4) or Fridericia‐corrected QT interval (QTcF) at week 12, and between changes from baseline. Moreover, sensitivity analysis showed no significant changes in systolic and diastolic BP and HR during the trial between patients with and without baseline cardiovascular medication (data not shown). We found no significant changes in BMI between baseline and end of the study in the pitolisant and placebo groups (mean kg [95% CI]: −0.4 [−0.5; −0.2] and − 0.4 [−0.6; −0.2], respectively). Blood chemistry and haematological parameters did not change significantly in either group. BDI‐13 total scores were similar in both treatment groups at baseline and at week 12. Mean changes in vital signs from baseline to week 12 were minimal, and no relevant differences between treatment groups were observed. Median (range) treatment adherence in the pitolisant and placebo safety groups was 100% (94.8; 100) and 100% (83.3; 100), respectively. Adherence was also comparable between subgroups after stratification by CPAP use.

**TABLE 4:** Mean change from baseline to week 12 in QTcB (safety population)

QTcB (ms)		Pitolisant (*N* = 241)	Placebo (*N* = 119)
Baseline	*n*	241	119
	Mean (95% CI)	419.5 (416.6; 422.4)	418.3 (413.8; 422.7)
Week 12	*n*	239	117
	Mean (95% CI)	421.4 (418.2; 424.6)	421.0 (416.0; 426.1)
Change week 12–Baseline	*n*	239	117
	Mean (SD)	2.0 (−0.8; 4.7)	2.8 (−1.4; 7.0)
Significance week 12 vs. baseline		NS	NS
Difference pitolisant vs. placebo		−0.84 [−5.75; 4.06]	
*p*‐value		*p* = 0.7362	

Abbreviations: CI, confidence interval; N, number of patients in the treatment group (safety population); n, number of patients with available data; NS, not statistically significant; QTcB, Bazett‐corrected QT interval.

## 4 DISSCUSSION

This randomised phase 3 clinical trial demonstrated the efficacy and safety of pitolisant in treating EDS in patients with OSA with or without concomitant CPAP therapy. At the end of the dose‐adjustment phase, 88.8% of patients were receiving the 40 mg dose. The efficacy of pitolisant was demonstrated by a significant improvement in the ESS score (mean reduction of −2.6) for pitolisant vs. placebo in the 12 week double‐blind period.

The reduction in ESS score with pitolisant is clinically significant as changes in ESS in OSA patients of −2 to −3 are considered to represent a minimum clinically important improvement (Patel et al., 2018). The results are in line with those from the HAROSA I and II clinical trials of OSA patients which reported clinically significant differences in ESS reduction for pitolisant up to 20 mg versus placebo of −2.6 and −2.8, respectively (Dauvilliers et al., 2020; Pépin, Georgiev, et al., 2021). In contrast to previous phase 3 trials of OSA which evaluated pitolisant at once‐daily doses up to 20 mg in patients adhering to CPAP (Pépin, Georgiev, et al., 2021), or not tolerating or refusing CPAP (Dauvilliers et al., 2020; Pépin, Georgiev, et al., 2021), this study assessed patients treated or not with CPAP with once‐daily doses up to 40 mg of pitolisant using an individual dose‐titration approach based on previous studies (European Medicines Agency, 2021; Lin et al., 2008). In patients with narcolepsy, pitolisant at once‐daily doses up to 40 mg had a favourable benefit/risk ratio on EDS for up to 1 year of treatment (Dauvilliers et al., 2013; Dauvilliers et al., 2019).

The current study included a heterogeneous group of OSA patients with residual EDS and, although no direct comparison was made because it was not the focus of the study, patients differed from those in previous phase 3 OSA trials (Dauvilliers et al., 2020; Pépin, Georgiev, et al., 2021), having less severe AHI (26.96 vs. 49.3 events per hour) and a higher mean nocturnal O_2_ saturation (91.0% vs. 90.1%) compared with HAROSA II (Dauvilliers et al., 2020). All three phase 3 HAROSA studies included populations which reflect the diversity and complexity of OSA in terms of the severity of sleep disturbances, age and geographic origin, and doses of pitolisant. Overall, the HAROSA studies suggest broad and robust efficacy of pitolisant in treating EDS in OSA patients who are treated or not with CPAP. In the present study, we found no interaction between pitolisant intake and CPAP use on changes in ESS, but both approaches are additive in their effectiveness. Also, no interaction was found with BMI and the pitolisant effect on ESS.

Compared with placebo, pitolisant improved the reaction time, measured objectively by OSleR, irrespective of CPAP use. According to previous studies which used the OSleR test to measure reaction time with CPAP therapy (Alakuijala et al., 2014; Bennett et al., 1997), our results showed that pitolisant produces a clinically relevant improvement in maintaining alertness. A recent meta‐analysis of individual patient data from the HAROSA I and II trials calculated that pitolisant (20 mg), compared with placebo, improved OSleR by 1.18 and reduced mean ESS by −3.1 (Lehert, 2022). In agreement with results obtained from the HAROSA I and II trials, pitolisant significantly improved both CGI‐C and PGOE compared with placebo, whether or not patients were also treated with CPAP (Dauvilliers et al., 2020; Pépin, Georgiev, et al., 2021).

Irrespective of CPAP use, pitolisant up to 40 mg was well tolerated and no new safety signals were identified. The overall tolerance was good, and incidence rates of global and treatment‐related TEAEs were similar in both treatment groups, with no major safety concerns raised irrespective of CPAP use. These safety results were similar to those observed with pitolisant 20 mg in the HAROSA I and HAROSA II studies. In particular, there were no safety concerns regarding cardiovascular parameters (QTc, HR, BP) which provides reassurance about the safety of pitolisant in this at‐risk OSA population treated or not with CPAP. A favourable cardiovascular safety profile of pitolisant was reported previously in phase 3 trials of patients with OSA up to 20 mg (Dauvilliers et al., 2020; Pépin, Georgiev, et al., 2021) and in narcolepsy up to 40 mg (Dauvilliers et al., 2013; Dauvilliers et al., 2019; Szakacs et al., 2017).

Although the current study has numerous strengths (including the use of polysomnography parameters that were not assessed in HAROSA I and II) and adds to the available literature regarding the efficacy and safety of pitolisant up to 40 mg once daily in patients with OSA, the main limitation relates to the 12 week duration. However, the randomised, double‐blind phase was followed by a 39 week open‐label extension phase to evaluate the long‐term maintenance of efficacy and safety (data to be reported elsewhere). Another limitation relating to study design is the flexible dosing which may have affected treatment efficacy, as less responsive patients are more likely to be titrated to the highest dose. This titration approach to dosing, and the fact that 88.8% of patients received pitolisant 40 mg, prevented a direct comparison of the effectiveness and safety of the different doses of pitolisant (particularly 20 vs. 40 mg) unlike the parallel group, placebo‐controlled trials. A formal comparison of the benefit/risk outcomes of different pitolisant doses may be explored further in post hoc analyses including data from HAROSA I, II and III using a dedicated meta‐analytical adjustment. Although the maintenance of wakefulness test can be used to evaluate daytime sleepiness, we used the OSleR test to assess the ability of individuals to maintain wakefulness and assessed daytime vigilance; the OSleR test is practical, well‐established, reliable, and validated (Krieger et al., 2004). Finally, personalisation of sleep apnea treatment is crucial and adherence to CPAP is a major issue. This study confirmed the excellent adherence to treatment in both populations stratified by CPAP use. However, we did not record the average usage of CPAP treatment throughout the study, CPAP adherence was based on clinical judgement.

In conclusion, pitolisant, at doses up to 40 mg once daily, is a safe and effective treatment for EDS in patients with moderate to severe OSA irrespective of CPAP use, as shown by a clinically relevant and significant reduction in ESS score. Pitolisant also improved reaction time, patient‐reported outcomes and physician disease severity assessment in OSA patients with excessive daytime sleepiness treated or not with CPAP.

## AUTHOR CONTRIBUTIONS

**Yves Dauvilliers:** Conceptualization; methodology; formal analysis; writing – review and editing; investigation. **Sonya Elizabeth Craig:** Conceptualization; methodology; investigation; formal analysis; writing – review and editing. **Maria R. Bonsignore:** Conceptualization; investigation; methodology; writing – review and editing; formal analysis. **Ferran Barbé:** Conceptualization; investigation; methodology; writing – review and editing; formal analysis. **Johan Verbraecken:** Conceptualization; investigation; methodology; writing – review and editing; formal analysis. **Jerryl Asin:** Conceptualization; investigation; methodology; writing – review and editing; formal analysis. **Ognian Georgiev:** Conceptualization; investigation; methodology; writing – review and editing; formal analysis. **Rumen Tiholov:** Conceptualization; investigation; methodology; writing – review and editing; formal analysis. **Christian Caussé:** Conceptualization; investigation; methodology; writing – review and editing; formal analysis. **Jeanne‐Marie Lecomte:** Conceptualization; investigation; methodology; writing – review and editing; formal analysis. **Jean‐Charles Schwartz:** Conceptualization; investigation; methodology; writing – review and editing; formal analysis. **Philippe Lehert:** Conceptualization; investigation; methodology; writing – review and editing; formal analysis. **Winfried Randerath:** Conceptualization; investigation; methodology; writing – review and editing; formal analysis. **Jean‐Louis Pépin:** Conceptualization; investigation; methodology; writing – review and editing; formal analysis.

## FUNDING INFORMATION

The study was supported by Bioprojet Pharma, Paris, France.

## CONFLICT OF INTEREST STATEMENT

JLP has received personal consulting fees, board engagements, and travel to conferences from Bioprojet, Jazz Pharmaceutical, and Idorsia. SC has received board engagements, honoraria for a lecture and travel to conferences from Bioprojet. MRB has received board engagements and travel to conferences from Bioprojet; seminars and travel to conferences from Jazz Pharmaceutical; consulting fees from Takeda Pharmaceuticals. FB has participated on a Data Safety Monitoring Board or Advisory Board for Bioprojet. JV has received funds for seminars, board engagements, and travel to conferences from Air Liquide, Atos Medical, Bioprojet, DEME, Desitin, Ectosense, Idorsia, Inspire Medical Systems, Jazz Pharmaceuticals, Löwenstein Medical, Medidis, Mediq Tefa, MSD, OSG, Philips, ProSomnus, ResMed Narval, SD Worx, Sefam, Somnolog, SomnoMed, Vemedia, Vivisol, and ZOLL Itamar. JA has attended advisory board meetings for Bioprojet; is a Board Member of the Dutch Sleep Medicine Association (SVNL); is a Board Member of the section SRBD (SAS) Dutch Pulmonary Association (NVALT). OG has no conflict of interest. RT has no conflict of interest. CC is an employee of Bioprojet Pharma. JML is the founder and current employee of Bioprojet Pharma. JCS is the co‐founder of Bioprojet. PL has received personal consulting fees from Bioprojet. WR has received personal fees/payment or honoraria for lectures, presentations, speaker's bureaus, manuscript writing or educational events from Heinen & Löwenstein, Habel Medizintechnik, Jazz Pharmaceuticals, Inspire, Philips Respironics, Bioprojet, and Westfalen Medical; personal fees of support for attending meetings and/or travel from: Heinen & Löwenstein, Jazz Pharmaceuticals, Philips Respironics, Habel Medizintechnik, and Bioprojet; personal fees for participation on a Data Safety Monitoring Board or Advisory Board from Bioprojet, Jazz Pharmaceuticals, Philips Respironics, Procter & Gamble; has had a leadership or fiduciary role in another board, society, committee or advocacy group, unpaid from European Respiratory Society Head Assembly 4, Sleep Disordered Breathing. YD has received funds for seminars, board engagements and travel to conferences from Jazz, Orexia, Idorsia, Takeda, Avadel, and Bioprojet.

## Supporting information


**FIGURE S1.** Study design.

**TABLE S1.** Full inclusion and exclusion criteria.

**DATA S1. eSAP1** Study protocol.

**DATA S2. eSAP2** Statistical analysis plan.

**DATA S3.** CONSORT checklist.

## Data Availability

The data that supports the findings of this study are available in the supplementary material of this article.

## HAROSA III STUDY GROUP COLLABORATORS

### 1

Dejan Dokic, MD, PhD, St Cyril and Methodoius University, Skopje, Macedonia; Hristo Metev, MD, PhD, Department of Pneumology, Ruse, Bulgaria; Iliev Kartev, MD, Department of Respiratory Diseases, Medical University, Plodiv, Bulgaria; Zdravka Karamyan, MD, Specialized Hospital for Active Treatment of Pneumology, Bourgas, Bulgaria; Ventsislav Nozharov, MD, Department of Internal Diseases, Multiprofile Hospital, Pleven, Bulgaria; Daniel Petkov, MD ENT, MHAT Transport Hospital, Bourgas, Bulgaria.
