# Supplementary material for: Pitolisant 40 mg for excessive daytime sleepiness in obstructive sleep apnea patients treated or not by CPAP: Randomised phase 3 study
Source: J Sleep Res. 2024 Oct 8;34(3):e14373. doi: 10.1111/jsr.14373 (PMC12069729; doi:10.1111/jsr.14373)
Supplement: Supplementary file 1 — FIGURE S1. Study design. [file JSR-34-e14373-s004.docx]

**eFigure 1 Study design**


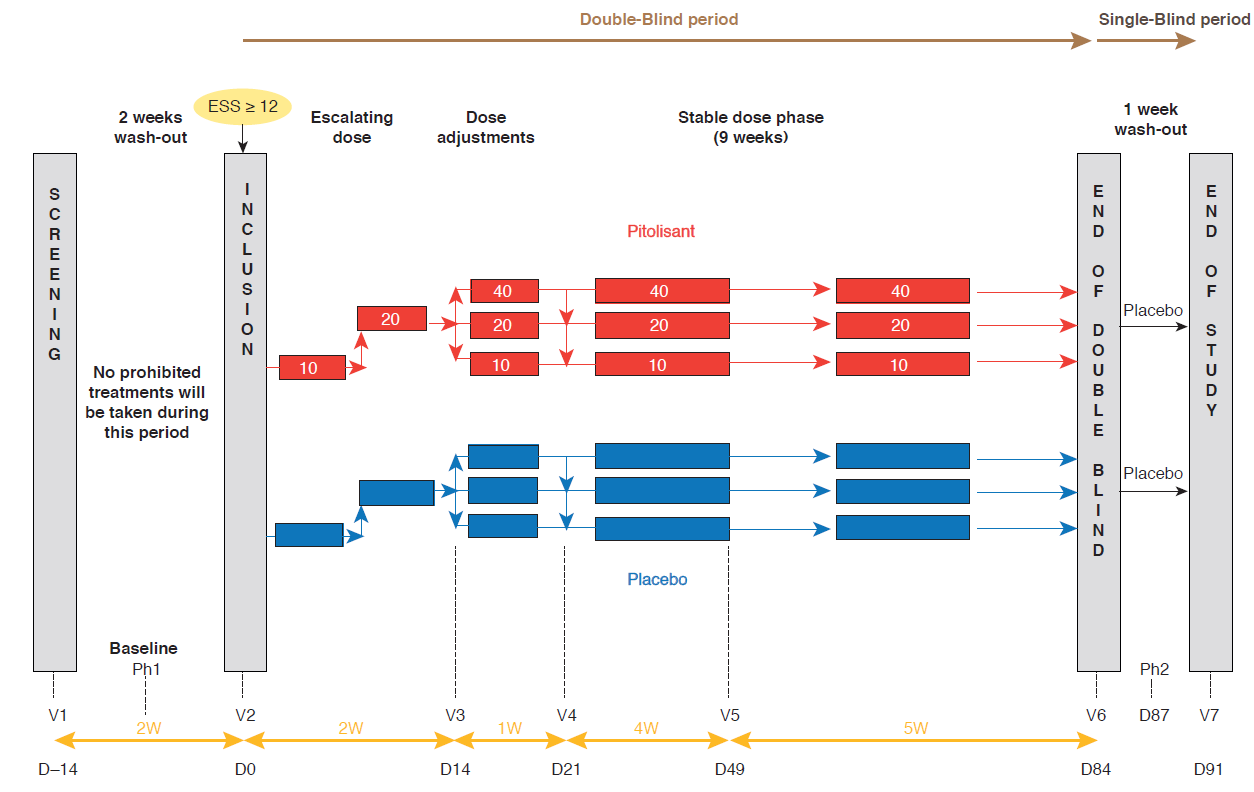


D = Day; ESS = Epworth Sleepiness Scale; Ph = Phone contact; V = Visit; W = Week. The doses of pitolisant stated correspond to mg/day dosing.

Prohibited treatments were: all wake-promoting agents, sodium oxybate, hypnotic drugs, H1R antagonists, tricyclic antidepressants, central antihypertensive drugs, drugs containing codeine or dextropropoxyphene, all surgeries for OSA.
