# Supplementary material for: Pitolisant 40 mg for excessive daytime sleepiness in obstructive sleep apnea patients treated or not by CPAP: Randomised phase 3 study
Source: J Sleep Res. 2024 Oct 8;34(3):e14373. doi: 10.1111/jsr.14373 (PMC12069729; doi:10.1111/jsr.14373)
Supplement: Supplementary file 2 — TABLE S1. Full inclusion and exclusion criteria. [file JSR-34-e14373-s001.docx]

**eTable 1. Full inclusion and exclusion criteria**

| **Inclusion criteria** | **Exclusion criteria** |
| --- | --- |
| 1) Male and/or female outpatients aged from at least 18 years;  2) Patients complaining of EDS refusing to be treated by CPAP therapy or having been submitted to CPAP therapy for a minimum period of 3 months, and still complaining of EDS despite the efforts made beforehand to obtain an efficient CPAP therapy;  3) Polysomnography performed (for patients submitted to CPAP therapy – under CPAP) between V1 and V2 or during the last 12 months with an apnea-hypopnea index (AHI):  o AHI ≥15 for patients without CPAP therapy;  o AHI ≤10 for patients under CPAP therapy;  4) For patients submitted to CPAP therapy: CPAP ≥4 hours/day (compliance checked on the clock-time counter of the CPAP machine);  5) Mini mental state examination (MMSE) ≥28;  6) BDI-13 score <16 and Item G (suicidal ideation) of BDI-13 = 0;  7) Body mass index (BMI) ≤40 kg/m²;  8) ESS ≥12;  9) Female patients with child-bearing potential using a medically accepted method of birth control (i.e. oral contraceptives of normal average dosage) agreeing to continue this method throughout the study, and during the month following treatment discontinuation, being negative to serum pregnancy test performed at the screening visit;  10) If specified by the Investigator, the patient had to be willing not to operate a car (if sleepy at wheel) or heavy machinery for the duration of the study or as long as the Investigator deemed it clinically indicated. In addition, the patient had to be willing to maintain during the study their usual behaviors which could affect their diurnal sleepiness (e.g. circadian rhythm, caffeine consumption, nocturnal sleep duration);  11) Patients having signed and dated the informed consent form. | 1) Patients having previously been exposed to pitolisant either in previous clinical trials, or in compassionate program or being prescribed the commercial form (Wakix^®^), for those enrolled patients from January 2018;  2) Patients suffering from chronic severe insomnia in accordance with the International Classification of Sleep Disorders (2005) without OSA;  3) Patients with co-existing narcolepsy, judged on clinical criteria;  4) Patients with sleep debt not due to OSA (according to the physician’s judgment);  5) Patients with non-respiratory sleep fragmentation (restless leg syndrome, etc.);  6) Shift work, professional drivers;  7) Refusal from the patient to stop any current therapy for EDS or predictable risk for the patient to stop the therapy;  8) Patients suffering from a psychiatric disease;  9) Acute or chronic disease preventing the improvement assessment, e.g. severe chronic obstructive pulmonary disease (COPD);  10) Current or recent (within 1 year) history of drug, alcohol, narcotic, or other substance abuse or dependence;  11) Any significant serious abnormality of the cardiovascular system, e.g. recent myocardial infarction, angina, hypertension, or dysrhythmias (within the previous 6 months), ECG Fridericia corrected QT interval (QTcF) higher than 450 msec, or history of left ventricular hypertrophy or mitral valve prolapse;  12) Severe co-morbid medical or biological conditions that may have jeopardized study participation at the discretion of the Investigator (particularly in the cardiovascular system and the unstable diabetes);  13) Positive serology tests (human immunodeficiency virus [HIV], hepatitis C virus [HCV] and hepatitis B surface antigen [HBsAg]);  14) Pregnant or breastfeeding women;  15) Women with child-bearing potential and no efficient birth control method;  16) Patients unable to understand the study protocol;  17) Patients with suspected or known hypersensitivity to study treatment;  18) Patients with a dominant arm deficiency impeding the achievement of the tests;  19) Patients using a prohibited medication;  20) Congenital galactose poisoning, glucose and galactose malabsorption, deficit in lactase;  21) Patients participating in another study or being in a follow-up period for another study. |
