# Supplementary material for: Pitolisant 40 mg for excessive daytime sleepiness in obstructive sleep apnea patients treated or not by CPAP: Randomised phase 3 study
Source: J Sleep Res. 2024 Oct 8;34(3):e14373. doi: 10.1111/jsr.14373 (PMC12069729; doi:10.1111/jsr.14373)
Supplement: Supplementary file 3 — DATA S1. eSAP1 Study protocol. [file JSR-34-e14373-s003.pdf]

16 APPENDICES

16.1 Study Information

16.1.1 Protocol and Protocol Amendments

The study protocol and amendment documents are provided on the following pages.

Document	Version	Date	Countries
Initial Protocol	1.0	02 December 2015	Bulgaria
**First protocol amendment**<br>Protocol amendment 1	1.0	01 February 2018	Bulgaria
**Amended protocol**<br>Protocol	2.0	01 February 2018	Bulgaria, North Macedonia

Efficacy and Safety of Pitolisant (BF2.649) in the Treatment of Excessive Daytime Sleepiness in Patients with Obstructive Sleep Apnoea Syndrome, Treated or Not by Nasal Continuous Positive Airway Pressure, but Still Complaining of Excessive Daytime Sleepiness

Sponsor
Bioprojet
9, Rue Rameau
75002 Paris, France
Tel.: +33 1 47 03 66 33 - Fax: +33 1 47 03 66 30

Medical Project Manager
[redacted]
Bioprojet
9, Rue Rameau
75002 Paris, France
Tel.: [redacted]

International Coordinator
[redacted]
University Multiprofile Hospital for Active Treatment
(UMHAT) "Aleksandrovska" EAD,
Clinic of Propedeutics of Internal Diseases
1, Sv. Georgi Sofiyski Str.
1432 Sofia, Bulgaria
Tel.: [redacted]
E-mail: [redacted]

Study Managing CRO
BalkanTrials Ltd.
8-B, Saint Kliment Ohridski Street
1504 Sofia, Bulgaria

TABLE OF CONTENTS

1. SYNOPSIS 9
2. STUDY DIAGRAM 17
3. INTRODUCTION 23
3.1. Summary of relevant non clinical studies 24
3.1.1. Pharmacological Profile 24
3.1.2. Safety profile 25
3.1.3. Pharmacology of pitolisant in potential therapeutic indication 26
3.1.3.1. *Effects on Sleep/wakefulness and EEG patterns in cats and mice* 26
3.1.3.2. *Effects on Learning* 27
3.1.3.3. *Effects on Attention Deficit/ Hyperactivity Disorder (ADHD)* 28
3.1.3.4. *Effects on Epilepsy Disorders* 28
3.1.3.5. *Effects on psychotic diseases* 29
3.1.4. Toxicology 29
3.1.4.1. Summary 29
3.1.5. Conclusion 34
3.2. Summary of relevant clinical studies 34
3.2.1. Phase I studies 35
3.2.2. Phase II Studies 36
3.2.2.1. Brief Summary 36
3.2.2.2. Two pilot studies referring to the Sleep Apnea Syndrome 38
3.2.2.3. Minimum effective dose-finding study of BF2.649, in patients with moderate to severe Obstructive Sleep Apnea, experiencing Excessive Daytime Sleepiness (EDS) despite regular use of nCPAP, and patients having refused this therapy. Randomized, double blind study with BF2.649 (5-, 10-, 20-, 40- mg/d), or placebo (P09-16 BF2.649) 43
3.2.3. Phase III studies 44
3.2.3.1. P09-08 Harosa 1 preliminary results 45
3.2.3.1. P09-09 Harosa 2 preliminary results 46
3.2.4. Summary of known and potential benefits and risks 46
4. RATIONALE OF THE CURRENT STUDY 48
5. STUDY OBJECTIVES 51
6. POPULATION 51
6.1. Inclusion Criteria 52
6.2. Non-Inclusion Criteria 53
6.3. Patient completion and early withdrawal of patients from study 54
7. EXPERIMENTAL DESIGN 55
8. STUDY SITES 59

9. INVESTIGATIONAL PRODUCTS ... *60*
9.1. Chemical structure of compounds ... *60*
9.2. Composition of investigational products ... *60*
9.2.1. Composition of 5 mg and 20 mg pitolisant tablet ... *60*
9.2.2. Composition of 5 mg and 20 mg placebo tablets ... *61*
9.2.3. Administration of study treatment ... *61*
9.2.4. Treatment Compliance ... *62*
9.2.5. Packaging and labelling of the treatment boxes ... *62*
9.2.6. Treatment quantity ... *63*
9.2.7. Management and storage of the therapeutic units ... *64*
9.2.8. Dispensation Modalities ... *65*
9.2.9. Pharmacological Forbidden Concomitant Treatments and surgical interventions ... *65*
9.2.10. Randomization of treatments ... *66*
9.2.10.1. Patient Study number ... 66
9.2.10.2. Investigational treatment allocation ... 66
9.2.10.3. Unblinding envelopes ... 67
10. EVALUATION CRITERIA ... *68*
10.1. Efficacy criteria ... *68*
10.1.1. Primary endpoint: Epworth sleepiness scale (ESS) ... *68*
10.1.2. Secondary endpoints ... *69*
10.1.2.1. Percentage of ESS responders ... 69
10.1.2.2. Reduction of sleepiness and sleep episodes on the sleep diary ... 69
10.1.2.3. Improvement in vigilance according to Oxford Sleep Resistance (OSleR) test ... 70
10.1.2.4. Increase in quality of life ... 71
10.1.2.5. Improvement in cognitive function ... 72
10.1.2.6. Improvement in Clinical Global Impression (CGI) ... 73
10.1.2.7. Patient's global opinion on the effect of investigational drugs ... 74
10.1.2.8. Aggregate Z-score of secondary endpoints. ... 74
10.2. Safety criteria ... *75*
10.2.1. Period of observation ... *75*
10.2.2. Adverse Event (AE) ... *75*
10.2.3. Treatment-Emergent and Baseline-Emergent Adverse Events ... *76*
10.2.4. Adverse Drug Reaction (ADR) ... *76*
10.2.5. Unexpected Adverse Drug Reaction ... *76*
10.2.6. Serious Adverse Event (SAE) or Serious Adverse Drug Reaction (Serious ADR) ... *76*
10.2.7. Suspected Unexpected Serious Adverse Reaction (SUSAR) ... *77*
10.2.8. Clarification of the difference in meaning between "severe" and "serious" ... *77*
10.2.9. Analysis of adverse events ... *78*
10.2.9.1. Assessment of intensity ... 78

10.2.9.2. Assessment of Causality 78
10.2.10. Reporting Adverse Event to the Sponsor (Appendix 12) 78
10.2.11. Reporting Serious Adverse Event to the sponsor 79
10.2.12. Reasons for expedited reporting 80
10.2.13. Adverse Events Outcome 81
10.2.14. Safety Endpoints 81
10.2.14.1. Adverse events 81
10.2.14.2. Physical examination 81
10.2.14.3. Vital signs 81
10.2.14.4. Electrocardiogram (ECG) 82
10.2.14.5. Beck Depression Inventory (the reduced version with 13 items) 82
10.2.14.6. Patient's overall evaluation of the tolerance 82
10.2.14.7. Amphetamine-like withdrawal symptoms questionnaire (DSM IV) 83
10.2.14.8. Laboratory tests 83
10.2.14.9. Pharmacokinetic assessments 85
10.2.14.10. Overdosing 86
10.2.14.11. Observance of nightly nCPAP use 86
11. *EMERGENCY PROCEDURES 86*
11.1. *Sponsor contact 86*
11.2. *Emergency identification of Study Products 87*
12. *CONDUCT OF THE TRIAL 88*
12.1. *Part I - Double Blind period 88*
12.1.1. V1 – Screening visit and beginning of initial wash-out period (D -14) 88
12.1.2. Ph1 – Phone contact (D -7) 90
12.1.3. V2 – Inclusion visit and beginning of the Double Blind period: baseline examinations, randomisation, start of escalating dose phase (D 0) 90
12.1.4. V3 – First dose adjustment visit (D 14) 92
12.1.5. V4 – Second dose adjustment visit and beginning of stable dose phase (D 21) 93
12.1.6. V5 – Control visit: continuation of stable dose phase (D 49) 94
12.1.7. V6 – Evaluation visit at the end of the Double Blind period and start of single-blindwash-out period (D 84) 95
12.1.8. Ph2 – Phone contact (V6 + 3D) 96
12.2. *Part II - Open Label Extension period 96*
12.2.1. V7 – End of study visit for patients who are not entering Open Label Extension period or Beginning of the Open Label Extension period for patients who are entering Open Label Extension period: start of new escalating dose phase (D 91) 96
12.2.2. V8-V9 – Dose adjustment visits (D 105 and D 112) 98
12.2.3. V10-V11 – Confirmed dose visits (D 196 and D 280) 99
12.2.4. V12 – Evaluation visit at the end of the Open Label Extension period and start of wash-out period

(D 364) 100
12.2.5. Ph3 – Phone contact (V12 + 3D)....101
12.2.6. V13 – End of study visit for patients who entered into Open Label Extension period (D 371)....101
13. *STATISTICAL ANALYSIS....102*
13.1. *Summary....102*
13.2. *Sample Size Determination and Justification....102*
13.3. *Primary and Secondary Populations....102*
13.4. *Handling of Missing Data....103*
13.5. *Futility Analysis....103*
13.6. *Statistical Analysis....103*
13.7. *Pharmacokinetic Analysis....104*
14. *DATA MANAGEMENT....104*
14.1. *Collection of data....104*
14.2. *Archiving of data, Audit....105*
14.3. *Data Protection and Confidentiality....106*
15. *QUALITY ASSURANCE....107*
15.1. *Good clinical Practice....107*
15.2. *Premature closure of the study....109*
15.2.1. Criteria for terminating the trial....109
15.2.2. Criteria for terminating an investigational site....109
15.3. *Control – Quality....110*
16. *REPORTING AND PUBLICATION OF RESULTS....110*
17. *CONTRACT, LIABILITY AND INSURANCE....110*
18. *FINANCIAL DISCLOSURE....111*
19. *CALENDAR FORECAST....111*
20. *REFERENCES....111*
21. *APPENDIX SECTION....113*
Appendix 1: Declaration of Helsinki....114
Appendix 2: Full-Night Polysomnography....120
Appendix 3: Epworth Sleepiness Scale (ESS)....121
Appendix 4: Sleep Diary....122
Appendix 5: European Quality of Life Questionnaire (EQ-5D)....123
Appendix 6: Leeds Sleep Evaluation Questionnaire (LSEQ)....125
Appendix 7: Pichot Fatigue Scale....126
Appendix 8: Trail Making Test (TMT) Parts A & B....127
Appendix 9: Clinical Global Impression (CGI) Rating Scale....132
Appendix 10: Patient's global opinion on the effect of investigational drugs....134
Appendix 11: DSMB charter....135
Appendix 12: Instructions for Filling In Bioprojet SAE Report Form....142

Appendix 13: Beck Depression Inventory – 13 Items (BDI-13)....................146
Appendix 14: Patient's overall evaluation of the tolerance....................148
Appendix 15: Amphetamine-Like Withdrawal Symptoms Questionnaire (DSM IV)....................149
Appendix 16: Mini Mental State Examination Questionnaire (MMSE)....................150

Signature's Page

The International Coordinator [redacted]
Medical expert

Date: 18.12.2015

[redacted]

The Sponsor [redacted]
Bioprojet Responsible Pharmacist

Date: 09.12.15

Signature [redacted]

Medical Project Manager [redacted]
Bioprojet

Date: 09/12/2015

[redacted]

Investigator Signature's Page

PROTOCOL P1513 / BF2.649

STUDY "HAROSA III"

EFFICACY AND SAFETY OF PITOLISANT (BF2.649) IN THE TREATMENT OF EXCESSIVE DAYTIME SLEEPINESS IN PATIENTS WITH OBSTRUCTIVE SLEEP APNOEA SYNDROME, TREATED OR NOT BY NASAL CONTINUOUS POSITIVE AIRWAY PRESSURE, BUT STILL COMPLAINING OF EXCESSIVE DAYTIME SLEEPINESS

The signature below constitutes the approval of this P1513 / BF2.649 – HAROSA III protocol and the attachments, and provides the necessary assurance that this trial will be conducted in accordance with all stipulations of the protocol, including all statements regarding confidentiality, and in accordance with local legal and GCP regulatory requirements, and ICH guidelines.

The site Investigator:

Name: ……………………………………………………………………………

Hospital: …………………………………………………………………………

Address: …………………………………………………………………………

Date: ___________________ Signature: ______________

1. SYNOPSIS

**TITLE**	Efficacy and Safety of Pitolisant (BF2.649) in the Treatment of Excessive Daytime Sleepiness in Patients with Obstructive Sleep Apnoea Syndrome, Treated or Not by Nasal Continuous Positive Airway Pressure, but Still Complaining of Excessive Daytime Sleepiness – Phase III.
**INTERNATIONAL COORDINATOR AND INVESTIGATORS**	Pr[redacted] – UMHAT "Alexandrovska" – 1, Sv. Georgi Sofiyski Str., Sofia, Bulgaria<br>Several site investigators in Bulgaria.
**STUDY OBJECTIVES**	The purpose of this double blind study is as follows:<br>The first objective of this study is to demonstrate the efficacy and safety of pitolisant given at 10, 20, or 40 mg per day versus placebo during 12 weeks of the Double Blind period, to treat the Excessive Daytime Sleepiness (EDS) in patients with Obstructive Sleep Apnea (OSA) refusing the nasal Continuous Positive Airway Pressure (nCPAP) therapy or treated by nCPAP but still complaining of EDS.<br>The secondary objectives of the study include assessing the long-term tolerance as well as the maintenance of efficacy of pitolisant given at 10, 20 or 40 mg per day during 39 weeks of Open Label Extension period and further investigating the co-variates or co-medications that affect the pharmacokinetics of pitolisant in the target population.
**STUDY DESIGN**	Prospective, multicenter, randomized, double blind, phase III study of pitolisant versus placebo during 12 weeks with, at first, an escalating dose period followed by a treatment at the selected dose.<br>Then, after one week of single-blind placebo wash-out period, if the patient holds the same position towards nCPAP therapy as before, an Open Label Extension period is proposed.<br>Patients who do not participate in the Open Label Extension period will have their end of the study visit.<br>Patients willing to continue the pitolisant treatment administration will be given another information notice together with an informed consent form to be signed. This Open Label Extension period will consist of the same escalating-dose, followed by a selected dose period with the active drug only, until 52 weeks after the treatment beginning. Then, patients will have one week wash-out period prior to the end of study visit.
**NUMBER OF PATIENTS**	Approximately 200 patients will be selected, so that 180 patients can be analyzed.<br>They will be divided into 4 groups as follows:<br>• 60 patients with OSA complaining of EDS refusing the nCPAP therapy will be administered pitolisant active ingredient product;<br>• 30 patients with OSA complaining of EDS refusing the nCPAP therapy will be administered a placebo;<br>• 60 patients with OSA treated by nCPAP but still complaining of EDS will be administered pitolisant active ingredient product;<br>• 30 patients with OSA treated by nCPAP but still complaining of EDS will be administered a placebo.
**POPULATION**	Patients exhibiting OSA, experiencing EDS with Epworth Sleepiness Scale (ESS) score

	≥ 12, refusing to be treated by nCPAP or having been submitted to nCPAP therapy for a minimum period of 3 months, and still complaining of EDS.
**MAIN INCLUSION CRITERIA**	The patients will have to present the following criteria:<br>• Male and/or female outpatients aged from at least 18 years<br>• Patients complaining of EDS refusing to be treated by nCPAP therapy or having been submitted to nCPAP therapy for a minimum period of 3 months, and still complaining of EDS despite the efforts made beforehand to obtain an efficient nCPAP therapy<br>• Polysomnography performed (for patients submitted to nCPAP therapy – under nCPAP) between V1 and V2 or during the last 12 months with Apnea-Hypopnea Index (AHI): for patients without nCPAP therapy ≥ 15; for patients under nCPAP therapy ≤ 10<br>• For patients submitted to nCPAP therapy: nCPAP ≥ 4 hours / day (compliance checked on the clock-time counter of the CPAP machine)<br>• Mini Mental State Examination (MMSE) ≥ 28<br>• Beck Depression Inventory – 13 items (BDI-13) score < 16 and item G (suicidal ideation) of BDI-13 = 0<br>• Body Mass Index (BMI) ≤ 40 kg/m²<br>• Epworth Sleepiness Scale (ESS) ≥ 12<br>• Female patients with child-bearing potential using a medically accepted method of birth control (i.e. oral contraceptives of normal average dosage) agreeing to continue this method throughout the study, and during the month following treatment discontinuation, being negative to serum pregnancy test performed at the screening visit<br>• If specified by the investigator, the patient must be willing not to operate a car (if sleepy at wheel) or heavy machinery for the duration of the trial or as long as the investigator deems it clinically indicated. In addition, the patient should be willing to maintain during the study their usual behaviors which could affect their diurnal sleepiness (e.g. circadian rhythm, caffeine consumption, nocturnal sleep duration)<br>• Patients having signed and dated the informed consent form
**MAIN NON-INCLUSION CRITERIA**	The patients should not present any of the following criteria:<br>• Patients suffering from chronic severe insomnia in accordance with the International Classification of Sleep Disorders (ICSD 2005) without OSA<br>• Patients with co-existing narcolepsy (ICSD 2005), judged on clinical criteria<br>• Patients with sleep debt not due to OSA (according to the physician' s judgment)<br>• Patients with non-respiratory sleep fragmentation (restless leg syndrome…)<br>• Shift work, professional drivers<br>• Refusal from the patient to stop any current therapy for EDS or predictable risk for the patient to stop the therapy<br>• Patients suffering from a psychiatric disease<br>• Acute or chronic disease preventing the improvement assessment, e.g. severe chronic obstructive pulmonary disease (COPD)

	• Current or recent (within one year) history of drug, alcohol, narcotic or other substance abuse or dependence<br>• Any significant serious abnormality of the cardiovascular system, e.g. recent myocardial infarction, angina, hypertension or dysrhythmias (within the previous 6 months), Electrocardiogram Fridericia corrected QT interval higher than 450 ms, history of left ventricular hypertrophy or mitral valve prolapse<br>• Severe co-morbid medical or biological conditions that may jeopardize study participation at the discretion of the investigator (particularly in the cardiovascular system and the instable diabetes)<br>• Positive serology tests (HIV, HCV and HBsAg)<br>• Pregnant or breast-feeding women<br>• Women with child-bearing potential and no efficient birth-control method<br>• Patients unable to understand the study protocol<br>• Patients with suspected or known hypersensitivity to study medication<br>• Patients with a dominant arm deficiency impeding the achievement of the tests<br>• Patients using a prohibited medication<br>• Congenital galactose poisoning, glucose and galactose malabsorption, deficit in lactase<br>• Patients participating in another study or being in a follow-up period for another study
**INVESTIGATIONAL TREATMENT**	Pitolisant and placebo are presented in identical tablets according to dosage, i.e. pitolisant tablets dosed at 5 mg or 20 mg and matching placebo.<br>During the Double Blind period patients will take daily 10 mg (2 tablets of 5 mg) or 20mg (1 tablet of 20mg) or 40 mg (2 tablets of 20 mg) of pitolisant or matching placebo. During the Open Label Extension period patients will take daily 10 mg, 20 mg or 40 mg of pitolisant (i.e. 2 tablets of 5 mg or 1 or 2 tablets of 20 mg, respectively).<br>Administration by oral route, once a day, in the morning, during breakfast, with a glass of water.
**PROHIBITED TREATMENTS**	All treatments indicated for somnolence, all drugs containing sodium oxybate, hypnotic drugs defined by ATC class; tricyclic antidepressants such as clomipramine, imipramine, desmethylimipramine and protriptyline displaying histamine H1 receptor antagonist activity that may affect the activity of pitolisant by abrogating the effect of its endogenous histamine release; H1 receptor antagonists (in particular those having an effect on the central nervous system), psychostimulants (amphetamine and amphetamine-like CNS stimulants, methylphenidate, modafinil or others); codeine; central antihypertensive drugs(Clonidine), and all drugs containing dextropropoxyphene (Di-Antalvic). Similarly, surgical procedures, such as mandibular advancement orthosis, Uvulopalatopharyngoplasty (UPPP) must be excluded.<br>Chronic treatments prescribed at a stable dose since at least one month before V1, unchanged throughout the study period and which do not interfere with daytime sleepiness can be continued and should be noted in the CRF.

**EFFICACY CRITERIA**	**MAIN ENDPOINT:**<br>• **Change of the score of ESS** between the baseline (score at baseline: mean between V1 and V2) and the end of the Double Blind period (mean between V5 and V6)<br>**SECONDARY ENDPOINTS:**<br>• **Percentage of ESS responders**: absolute value < 11 or difference between baseline and the end of the Double Blind period scores ≥ 3<br>• **Reduction of sleepiness and sleep episodes on the sleep diary,** filled in by patients during 3 sequential days of the week preceding each visit<br>• **Improvement in vigilance according to Oxford Sleep Resistance (OSleR) test**: increase in sleep latency and reduction in the number of errors<br>• **Increase in quality of life:** European Quality of Life Questionnaire (EQ-5D), Leeds Sleep Evaluation Questionnaire (LSEQ) and The Pichot Fatigue Scale<br>• **Improvement in cognitive function:** Trail Making Test (TMT) Parts A & B<br>• **Improvement in Clinical Global Impression (CGI-C)** assessment<br>• **Patient's global opinion on the effect of investigational drug**<br>• **Z-score:** composite score including ESS and OSLER results
**SAFETY CRITERIA**	• **Adverse Events** – at each visit<br>• **Physical examination** – at each visit<br>• **Vital signs**: blood pressure and heart rate – at each visit<br>• **Electrocardiogram (ECG)** – at each visit<br>• **Beck Depression Inventory – 13 items** – at visits V1, V2, V6, V7, V9, V10, V11, V12 and V13<br>• **Patient's overall evaluation of the tolerance** – at visits V3, V4, V5, V6, V7, V8, V9, V10, V11, V12 and V13<br>• **Amphetamine-like withdrawal symptoms questionnaire (DSM IV)** – at phone contact 2, V7, phone contact 3 and V13<br>• **Laboratory tests**: evaluation of the biological work-up (hematology, biochemistry at visits V1, V6 and V12, urinalysis at V1). Biological work-up will also be performed as soon as possible for any subject who discontinues the study prior to the completion of the Double Blind period or the Open Label Extension period.<br>• **Overdosing**<br>• **Observance in nightly CPAP use**, as assessed by the clock-time counter of the CPAP machine at each visit (reading of the last day) prior to each visit (for patients submitted to nCPAP therapy).
**STUDY SCHEDULE**	*All tests and examinations to be performed are described in section 2 concerning the study diagram*<br>**V1 – Screening visit and beginning of initial wash-out period (D -14)**<br>Before proposing the study to patients refusing to be treated by nCPAP, the investigator will ensure that the patient still refuses this treatment.

An appropriately signed informed consent will be obtained prior to entry into the study for each patient having made a positive decision to participate. The investigator must confirm that the patient meets all inclusion criteria and none of the non-inclusion criteria

Patients included in the study shall not take any treatment indicated for EDS or other psychotropic drugs noted in chapter prohibited treatment during the following two weeks before being submitted to baseline examination (with the exception of chronic medications taken to treat pathology, and authorized by the protocol).

Patients will be reminded to contact the investigator between the visits for any issue, such as adverse events, difficulties with the treatment, need for a new treatment, associated pathology, and modification of the current concomitant treatment (applicable for the whole study duration).

Ph1 – Phone contact (D -7)

During this phone contact, the investigator should:

- Check if patient has discontinued prohibited treatment
- Check concomitant treatments
- Check occurrence of AEs

V2 – Inclusion visit and beginning of the Double Blind period: baseline examinations, randomisation, start of escalating dose phase (D 0)

The patients will be randomized to either pitolisant, or placebo.

The treatment will be initiated by an individual titration period over 2 weeks.

1st week: At each morning, with a glass of water during breakfast

Patients on pitolisant will receive

During breakfast	10 mg

Patients on Placebo will receive

During breakfast	Placebo

2nd week: At each morning, with a glass of water during breakfast

Patients on pitolisant will receive

During breakfast	20 mg

Patients on Placebo will receive

During breakfast	placebo

The total treatment period in double-blind is 12 weeks.

V3 – First dose adjustment visit (D 14)

The posology is increased at 40 mg/d pitolisant (high dose) or placebo every morning, during breakfast. If the tolerance does not allow it (occurrence of an adverse event i.e. troublesome insomnia), the patient will continue the treatment intake at 20 mg/d pitolisant (medium dose) or placebo or the posology will be eventually reduced at 10 mg/d pitolisant (low dose) or placebo until V4.

V4 – Second dose adjustment visit and beginning of stable dose phase (D 21)

The posology is maintained for the next following 4 weeks.

	If the tolerance does not allow it:<br>- Patients taking 40 mg/d pitolisant (high dose) or placebo could reduce to 20 mg/d pitolisant (medium dose) or placebo for the following 4 weeks.<br>- Patients taking 20 mg/d pitolisant (medium dose) or placebo could reduce to 10 mg/d pitolisant (low dose) or placebo for the following 4 weeks.<br>Any dose increase will not be allowed at this visit.<br>After V4 until the end of Double Blind period any dosage change of pitolisant or placebo will not be allowed.<br>**V5 – Control visit: continuation of stable dose phase (D 49)**<br>Any dosage change of pitolisant or placebo will not be allowed at this visit. Hence, pitolisant or placebo treatment at the same stable dose will be continued for another 5 weeks.<br>**V6 – Evaluation visit at the end of the Double Blind period and start of single blind wash-out period (D 84)**<br>Patients are submitted to the tests and examinations required for the analysis of the double blind study results. Patients will start one-week single blind placebo wash-out period.<br>**Ph2 – Phone contact (V6 + 3D)**<br>During this phone contact, the investigator should:<br>- Get patient's answers to amphetamine-like withdrawal symptoms questionnaire (DSM IV) and find out patient's global opinion on the effect of investigational drug<br>- Check concomitant treatments<br>- Check occurrence of AEs<br>**V7 – End of study visit for patients who are not entering Open Label Extension period or Beginning of the Open Label Extension period for patients who are entering Open Label Extension period: start of new escalating dose phase (D 91)**<br>If the patient holds the same position towards nCPAP therapy as before, he/she will be proposed to enter the Open Label Extension period of the study after another escalating dose phase.<br>Patients not willing to continue the pitolisant treatment administration will end their participation in the study at this visit.<br>Patients willing to continue the pitolisant treatment administration will be given another information leaflet together with an informed consent form to be signed. After having signed the informed consent form to confirm that they agree to participate in the Open Label Extension study period, patients will be administered 10 mg pitolisant OD during one week. Then, on the following week, they will be administered 20 mg pitolisant OD (in the morning, during breakfast, with a glass of water).<br>**V8-V9 – Dose adjustment visits (D 105 and D 112)**<br>The posology is increased to 40 mg/d pitolisant (high dose), every morning, during breakfast. If the tolerance does not allow it (i.e. troublesome insomnia), the patient will get the treatment at dose of 20 mg/d (medium dose) or 10 mg/d (low dose).<br>At each of the visits V8-V11 the investigator will propose nCPAP to the patients refusing to be treated by nCPAP therapy before giving them the study treatment. If the

	patient accepts, he/she will be withdrawn from the study.<br><br>**<u>V10-V11 – Confirmed dose visits (D 196 and D 280)</u>**<br><br>The posology should be 40 mg/d (high dose) if the tolerance is acceptable. If the study drug is not well tolerated, the investigator may decide to decrease the dose to 20 mg/d (medium dose) or 10 mg/d (low dose) during the 12-week period following the visit.<br><br>**<u>V12 – Evaluation visit at the end of the Open Label Extension period and start of wash-out period (D 364)</u>**<br><br>Patients are submitted to the examinations and tests required for the evaluation of long-term tolerance and maintenance of efficacy of pitolisant. Patients will be submitted to a final visit after a one-week wash-out period. No study treatment will be taken during this period.<br><br>**<u>Ph3 – Phone contact (V12 + 3D)</u>**<br>During this phone contact, the investigator should:<br>- Get patient's answers to amphetamine-like withdrawal symptoms questionnaire (DSM IV) and find out patient's global opinion on the effect of investigational drug<br>- Check concomitant treatments<br>- Check occurrence of AEs<br><br>**<u>V13 – End of study visit for patients who entered into Open Label Extension period (D 371)</u>**<br><br>The patients are summoned by the investigator for a final visit. Then, the investigator will decide about the new treatment to prescribe to the patients and the patients will be discharged from the study.<br><br>***The date of each visit could be D ± 3 Days***
STATISTICAL ANALYSIS	<u>Sample Size Determination and Justification:</u><br><br>Results from exploratory studies on pitolisant allow to estimate the ESS residual variability to SD = 6. The Minimum Important Difference MID was fixed to ESS = 3, corresponding to an effect size ES = 0,5. The correlation between Final and Baseline ESS was conservatively estimated to r = 0,3. By assuming Analysis of Covariance test (ANCOVA) at 0,95 confidence level as the main confirmatory test, and a sample ratio 1:2, a difference of at least Delta = 3 should be detected with a power of 90% in using at least 60 patients in placebo group and 120 patients in pitolisant treatment group. Treatment groups will be stratified by center and CPAP use. Considering 10% drop out rate, 200 patients will be selected.<br><br><u>Statistical Analysis:</u><br><br>Final ESS will be compared between the two treatments by an ANCOVA at two-sided 95% in adjusting for ESS at baseline, and by considering the random effect center, and the fixed effect treatment and assessing the additional effect of obesity (BMI) on outcome. This test will be implemented by a Mixed Linear Model. The confirmatory analysis will be based on a simple ANCOVA model assuming no interaction between baseline and treatment (assumption of parallelism).
STUDY DURATION AND DATES	The study will be conducted in two periods.<br><br>Depending on whether the patients will participate in the Double Blind period only, or whether they will continue to be administered the pitolisant treatment in the Open

	Label Extension period, the study duration for each patient will be 15 weeks, or 55 weeks.
**STUDY START**	First patient in: 1-2 Q 2016
**END OF THE STUDY**	Last patient last visit: 2 Q 2017

2. STUDY DIAGRAM

Double Blind period

Period / Visit [1]	V1 D -14	1 Week Wash-out period	Phone cont. 1 D -7	1 Week	V2 D 0	W 1 10 mg	W 2 20 mg	V3 D 14	W 3 10 mg or 20 mg or 40 mg	V4 D 21	W 4 → W 7 Selected dose (4 weeks)	V5 D 49	W 8 → W 12 Selected dose (5 weeks)	V6 D 84	W 13 1 week wash-out period	Phone cont. 2 V6 +3D	V7 [6] End of study D 91
Signature of Consent Form	X																
Medical questionnaire	X		X		X			X		X		X		X		X	X
Physical examination	X				X			X		X		X		X			X
ESS	X				X			X		X		X		X			X
Polysomnography [2]				X													
OSleR test [3]					X									X			
MMSE	X																
TMT parts A & B					X									X			
CGI-S	X				X												
CGI-C														X			X
BDI-13	X				X									X			X
LSEQ, EQ-5D					X									X			
Pichot Fatigue Scale					X									X			X
Patient's global opinion on the effect of investigational drugs														X		X	X
Patient's overall evaluation of the tolerance								X		X		X		X			X
Amphetamine-like withdrawal symptoms questionnaire																X	X
ECG	X				X			X		X		X		X			X
Safety biology [4]	X													X			
Delivery of sleep diary	X				X			X		X		X		X			
Review of sleep diary [5]					X			X		X		X		X			X
Adverse events			X		X			X		X		X		X		X	X

1 – Each visit shall be carried out at the end of the relevant time period ± 3 days.
2 – Overnight polysomnographic recording performed from 22:00 until 7:00 (minimum 8 hours of recording) in the sleep laboratory only between V1 and V2 except if available in the previous 12 months.
3 – OSleR test: 3 sequences at 2 hours interval (at 9:00, 11:00, and 13:00).
4 – Complete biological examination: hematology (hemoglobin, hematocrit, red and white blood cell count (with differential), platelets, mean corpuscular volume, coagulation time (INR)), biochemistry (blood urea nitrogen (BUN), uric acid, creatinine, creatine kinase, ALAT, ASAT, GGT, alkaline phosphatases, total protein, total bilirubin, glucose, electrolytes (sodium, potassium, calcium, chloride, bicarbonates/CO_2), total cholesterol, triglycerides), serology (HIV, HCV, HBsAg at V1, β-HCG (for woman with child-bearing potential)), urianalysis: stick (with microscopy and bacteriological culture, if positive) at V1.
5 – At each visit, the patient shall bring back his sleep diary. Patient will be contacted in advance before each visit to remind him/her to fill in the sleep diary. The patient shall return the unused drug at each visit.
6 – Only for patients who are not entering into Open Label Extension period; for others see Open Label Extension period study diagram.

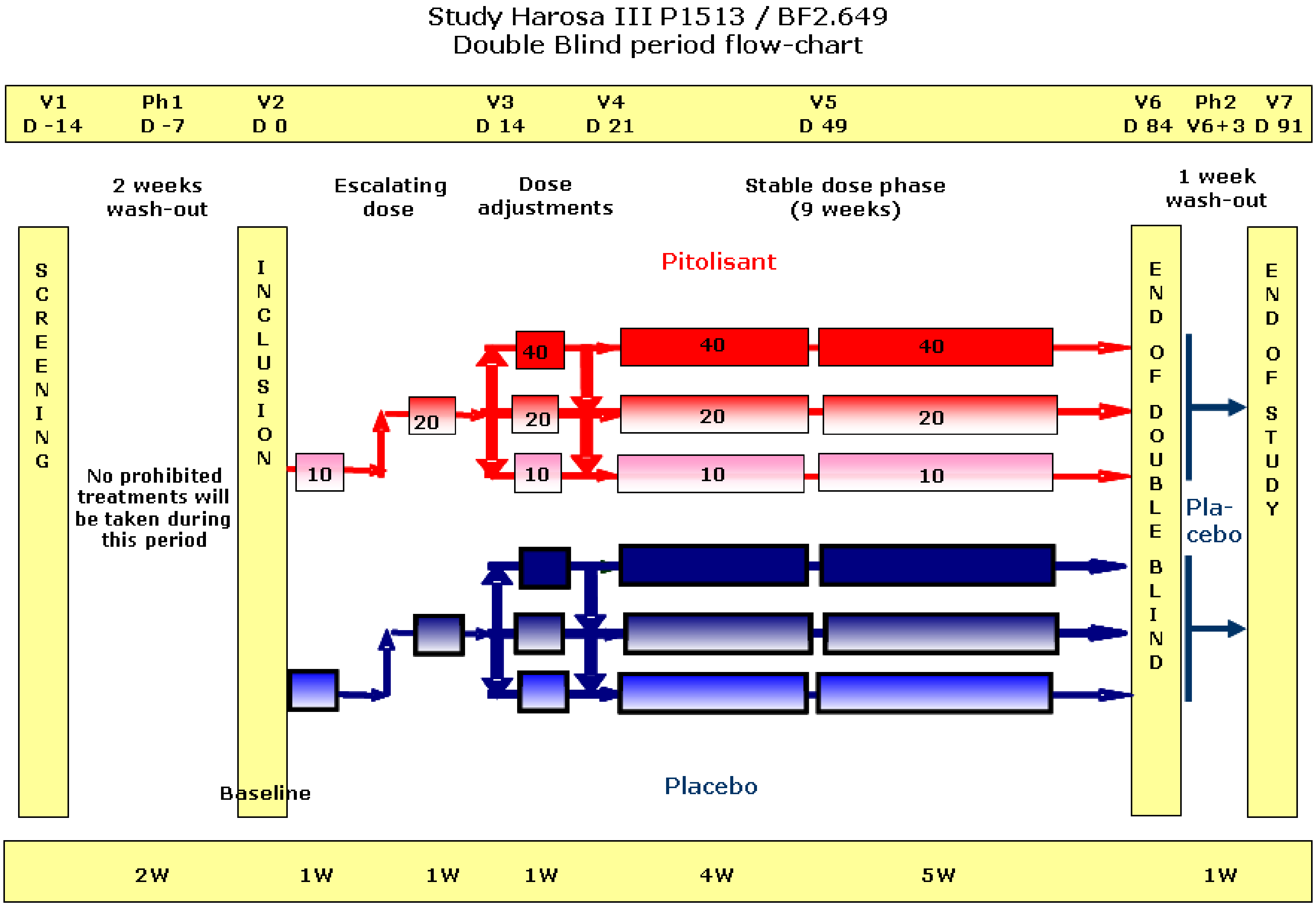
Study Harosa III P1513 / BF2.649
Double Blind period flow-chart
V1 D -14
Ph1 D -7
V2 D 0
V3 D 14
V4 D 21
V5 D 49
V6 D 84
Ph2 V6+3
V7 D 91
2 weeks wash-out
Escalating dose
Dose adjustments
Stable dose phase (9 weeks)
1 week wash-out
SCREENING
INCLUSION
No prohibited treatments will be taken during this period
Pitolisant
40
20
10
Placebo
Pla-cebo
END OF DOUBLE BLIND
END OF STUDY
Baseline
2W
1W
1W
1W
4W
5W
1W

Open Label Extension period

**Period**	**V7** [2] D 91	**W 14**	**W 15**	**V8** D 105	**W 16**	**V9** D 112	**W 17 → W 28**	**V10** D 196	**W 29 → W 40**	**V11** D 280	**W 41 → W 52**	**V12** D 364	**W 53**	**Phone cont. 3** V12 +3D	**V13** End of study D 371
**Visit** [1]		10 mg	20 mg		10 mg or 20 mg or 40 mg		10 mg or 20 mg or 40 mg		10 mg or 20 mg or 40 mg		10 mg or 20 mg or 40 mg		1 week Wash-out period		
Signature of Consent Form	X														
Medical questionnaire	X			X		X		X		X		X		X	X
Physical examination	X			X		X		X		X		X			X
ESS	X			X		X		X		X		X			X
OSleR test [3]	X											X*			
TMT parts A & B	X											X			
CGI-C	X							X		X		X			X
BDI-13	X					X		X		X		X			X
LSEQ, EQ-5D	X											X			
Pichot Fatigue Scale	X					X		X		X		X			X
Patient's global opinion on the effect of investigational drugs	X							X		X		X		X	X
Patient's overall evaluation of the tolerance	X			X		X		X		X		X			X
Amphetamine-like withdrawal symptoms questionnaire	X													X	X
ECG	X			X		X		X		X		X			X
Safety biology [4]												X			
Delivery of sleep diary	X			X		X		X		X		X			
Review of sleep diary [5]	X			X		X		X		X		X			X
Adverse events	X			X		X		X		X		X		X	X
Pharmacokinetics sampling [6]								X							

1 – Each visit shall be carried out at the end of the relevant time period ± 3 days.
2 – Only for patients who are entering into Open Label Extension period; for others see Double Blind period study diagram.
3 – OSleR test: 3 sequences at 2 hours interval (at 9:00, 11:00 and 13:00). Optional at V12.
4 – Complete biological examination: hematology (hemoglobin, hematocrit, red and white blood cell count (with differential), platelets, mean corpuscular volume, coagulation time (INR)), biochemistry (blood urea nitrogen (BUN), uric acid, creatinine, creatine kinase, ALAT, ASAT, GGT, alkaline phosphatases, total protein, total bilirubin, glucose, electrolytes (sodium, potassium, calcium, chloride, bicarbonates/CO_2), total cholesterol, triglycerides), serology (β-HCG (for woman with child-bearing potential)).
5 – At each visit, the patient shall bring back his sleep diary. Patient will be contacted in advance before each visit to remind him/her to fill in the sleep diary. The patient shall return the unused drug at each visit except at V13.
6 – for all patients.

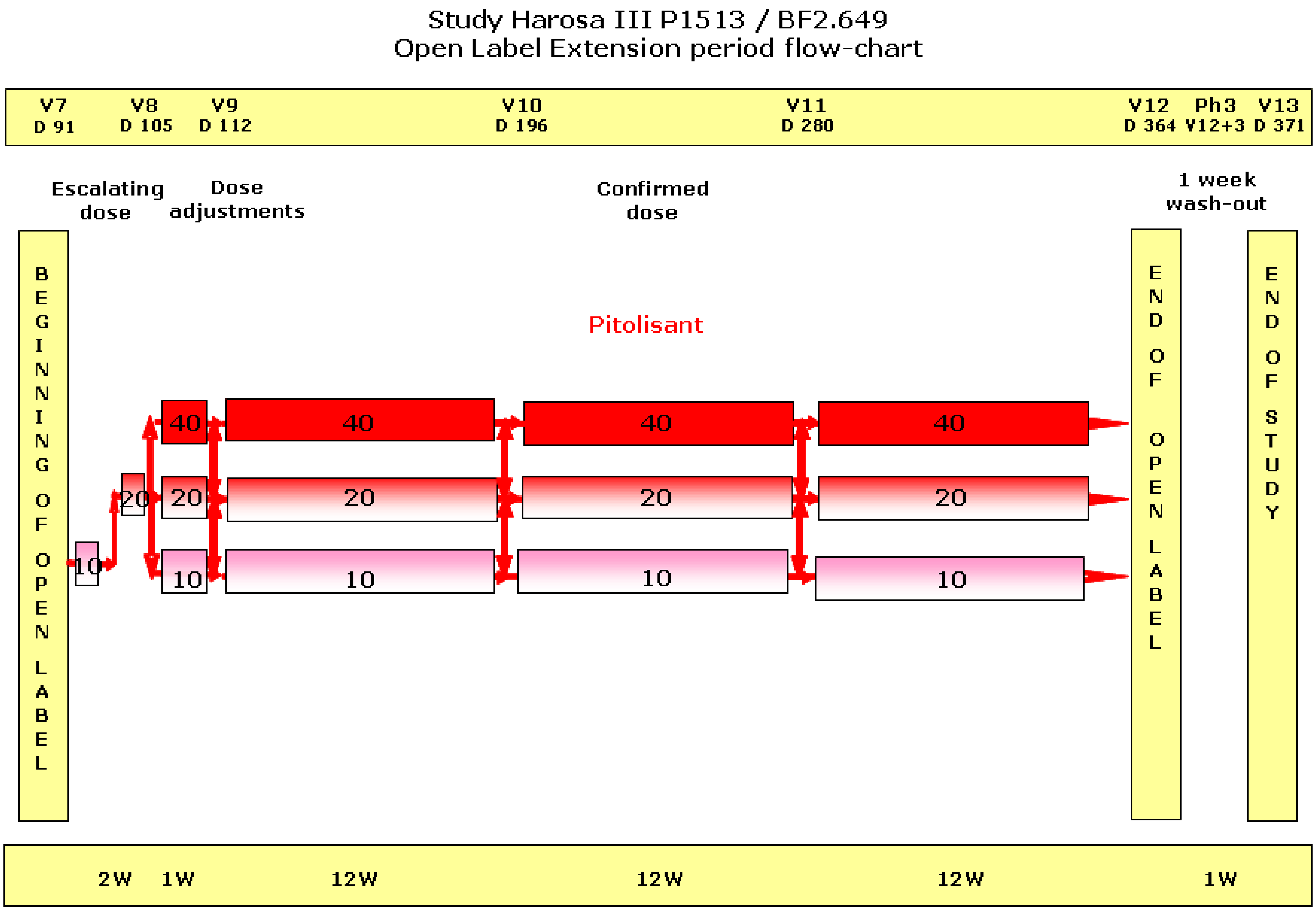
Study Harosa III P1513 / BF2.649
Open Label Extension period flow-chart
V7
D 91
V8
D 105
V9
D 112
V10
D 196
V11
D 280
V12
D 364
Ph3
V12+3
V13
D 371
Escalating dose
Dose adjustments
Confirmed dose
1 week wash-out
BEGINNING OF OPEN LABEL
Pitolisant
10
20
40
40
40
40
20
20
20
20
10
10
10
10
END OF OPEN LABEL
END OF STUDY
2W
1W
12W
12W
12W
1W

ABBREVATIONS USED IN THE PROTOCOL

5-HT	Serotonin or 5-hydroxytryptamine
ADHD	Attention Deficit/Hyperactivity Disorder
AEs	Adverse events
AHI	Apnea Hypopnoea Index
ALAT/SGPT	Alanine aminotransferase
ANCOVA	Analysis of Covariance test
APD50	Action potential duration at 50% repolarization
APD90	Action potential duration at 90% repolarization
ASAT/SGOT	Aspartate aminotransferase
ATC	Anatomical Therapeutic Chemical classification system
AUC	Area under the concentration-time curve
BDI-13	Beck Depression Inventory – 13 items
BMI	Body mass index
CGI	Clinical Global Impression
CGI-C	Clinical Global Impression – Global Improvement or Change
CGI-S	Clinical Global Impression – Severity of Illness
Cmax	Maximum observed concentration
CNS	Central nervous system
COPD	Chronic obstructive pulmonary disease
CPAP	Continuous Positive Airway Pressure
CPK	Creatine kinase
CRF	Case report form
CRO	Contract Research Organisation
DMB	Data Management Book
DSMB	Data Safety Monitoring Board
DSE	Diurnal Sleepiness episodes
EC	Ethics Committee
ECG	Electrocardiogram
EDS	Excessive Daytime Sleepiness
EEG	Electroencephalography
EMEA	European Medicines Agency
EMG	Electromyography channel
ES	Effect Size
ESS	Epworth Sleepiness Scale

EQ-5D	European Quality of Life Questionnaire
FAS	Full analysis set
GABA	Gamma-aminobutyric acid
GCP	Good Clinical Practice
GMP	Good Manufacturing Practice
GGT	Gamma-glutamyltranspeptidase
H3R	Histamine H3 receptor
ICH	International conference on harmonization of technical requirements for registration of pharmaceuticals for human use
ICSD	International Classification of Sleep Disorders
INR	International Normalized Ratio
IRB	Institutional Review Board
MCI	Mild Cognitive Impairment
MID	Minimum Important Difference
MMSE	Mini Mental State Examination
nCPAP	nasal Continuous Positive Airway Pressure
NOAEL	No observable adverse effect levels
OD	Once a day
ODL	Optimal Dose Level
OSA	Obstructive Sleep Apnea syndrome
OSleR	Oxford Sleep Resistance test
PP	Per Protocol
PSG	Polysomnography
SAE	Serious Adverse Event
SaO_2	Oxygen Saturation
SAP	Statistical Analysis Plan
SD	Standard Deviation
ß-hCG	Human chorionic gonadotropin
Tmax	Time to Cmax
TMT	Trail Making Test

3. INTRODUCTION

Excessive daytime sleepiness (EDS) is a major complaint in patients with obstructive sleep apnoea syndrome (OSA) defined as sleep-related breathing disorder (SRBD) with full or partial occlusion of the upper airway during the sleep. OSA afflicts at least 2-4% of the adult population (at least 4% of males and 2% of females)[1,2].

The proposed mechanism for EDS in OSA patients is sleep disturbance and loss of sleep resulting from microarousals produced by increased ventilatory effort[3,4].

Patients may be personally unaware of their snoring and breathing pauses during sleep, but the consequences of daytime hypersomnolence such as deficits in attention or vigilance (road accident, accident at work)[5] impaired short-term memory and reduced capacity to sustain concentration or focus are often essential to rational clinical decision about whom to treat [6].

Nasal continuous positive airway pressure (nCPAP) is the treatment of choice for most patients with OSA[7,8,9,10]. When used properly, nCPAP reduces apnoea and hypopnoea rate, often normalizes arterial blood oxygen saturation, decreases sleep fragmentation, and improved sleep quality. As a result, alertness, mood, cognitive function, and quality of life improve[11]. Furthermore nCPAP reduces risks of cardiovascular events in obstructive sleep apnea patients.

Pitolisant (BF 2.469), novel selective histamine H3 receptor antagonist/agonist inverse exhibits strong waking promoting effects and a good tolerance in a number of preclinical and clinical studies, appeared completely indicated.

Experimental evidence for enhanced wakefulness induced by H3R blockade was previously reported with thioperamide, the prototypic H3R antagonist [*Lin et al., Brain Res. 1990, 523: 325-30*]. Similar vigilance- and attention-promoting effects were described more recently with the antagonist ciproxifan [*Ligneau et al., J. Pharmacol. Exp. Ther. 1998, 287: 658-66*].

Improvement of learning deficit and memory by an H3 antagonist was also demonstrated experimentally in mice [*Miyazaki et al., Life Sci. 1995, 57: 2137-44; Meguro et al., Pharmacol. Biochem. Behav. 1995, 50: 321-5; Onodera et al., Naunyn-Schmiedeberg's Arch. Pharmacol. 1998, 357: 508-13*]. The effects of pitolisant on sleep/wakefulness, and EEG patterns have been investigated in cats and mice. It has been shown that pitolisant increased markedly the duration of waking in these two animal species. Moreover, the EEG results suggest that pitolisant could improve the quality of arousal, by reinforcing e.g. the level of vigilance and attention, in the absence of any noticeable psychomotor activation that is

observed with drugs like amphetamines.

In healthy volunteers, the clinical and biological tolerance of pitolisant was excellent with single oral dose between 1 mg and 240 mg.

3.1. Summary of relevant non clinical studies

3.1.1. Pharmacological Profile

BF2.649 is a novel, highly potent, selective, orally active histamine H3 receptor antagonist/inverse agonist with a Ki between 0.3 nM and 2.4 nM at the human receptor. It does not interact significantly with other histamine receptor subtypes or with a large variety of other receptors.

When given parenterally or orally, BF2.649 enhances the activity of histaminergic neurons in brain, a major wake-promoting system, and thereby improves vigilance, learning and memory and displays pro-cognitive properties. BF2.649 exhibits strong waking effects in several animal species (rodents and cats) and improves the level of vigilance, attention and learning without causing locomotor excitation. BF2.649 showed strong waking effects on normal animals as well as on experimental models of narcolepsy and Parkinson's disease. In agreement, it enhances the activity of major arousal and procognitive systems *projecting to cortical areas* (prefrontal cortex, hippocampus), i.e. not only histaminergic, but also noradrenergic, cholinergic and dopaminergic. However, this pro-waking and pro-cognitive profile is clearly distinct from those of typical psychostimulants of the amphetamine type, including modafinil: *dopamine release is unaffected* in the striatal complex (including the nucleus accumbens), psychomotor activation is not elicited (and methamphetamine-induced locomotor activation is even depressed) and behavioural sensitization does not develop upon repeated administration.

In an animal model of narcolepsy (orexin-knockout mouse) the waking effect is accompanied by a marked inhibition of DREMs (direct transitions from Wake to REM sleep, occurring during cataplexies), again an effect not observed with psychostimulants including modafinil.

Also in animal studies, BF2.649 showed a beneficial effect on models corresponding to human absence epilepsy or temporal lobe seizures. BF2.649 was effective in animal models of schizophrenia, i.e. to block partially the psychomotor responses to amphetamine or dizocilpine. It also decreases the locomotor activity and counteracts the cognitive deficits of

dopamine-transporter knock out mice, a model of psychosis and ADHD. It also potentiates the “antipsychotic” effect of risperidone and olanzapine in e.g. dizolcipine-induced hyperlocomotion.

3.1.2. Safety profile

Safety profile has been assessed on cardiovascular, respiratory, gastric and central nervous functions. Some effects were reported, but only at high concentrations or high doses, when compared to Human therapeutic doses (~ 20 mg/day, repeated):

BF2.649 dose-dependently inhibited hERG channels stably expressed in HEK293 cells, with an IC50 value of 1.32 µM to be compared with therapeutic plasma levels of about 0.4 µM (total concentration) and 0.01 µM (free concentration). IC50 values of BF2.649 on a large panel of cardiac channel currents (Nav1.5, Kv4.3, Kv7.1/mink, Kir2.1, Kv1.5, Cav1.2 and Cav3.2) were above 10 µM or ~ 10 µM (Cav1.2, Kv4.3). On human ventricular cardiomyocytes, BF2.649 decreased significantly APD20 (26±3% and 38±1% at 3 and 10 µM, respectively) and increased non-significantly APD90 by 17±9%, 18±8% and 11±4% at 1, 3 and 10 µM, respectively. Dofetilide (0.1 µM) induced a significant increase of +23±2% in APD90 which was fully abolished when the BF2.649 (10 µM) was associated with dofetilide (100 nM). These results are consistent with the inhibitory action on hERG channel and a calcium blocking effect of BF2.649 recorded at BF2.649 concentration higher than 1 µM. In anaesthetized rabbits, BF2.649 did not significantly affect corrected QT interval and no arrhythmic event was observed even at the highest dose. In addition, in the methoxamine-sensitized rabbit, BF2.649 devoid of any pro-arrhythmic effect by itself was even able to reduce arrhythmias score of clofilium, an agent know to prolong the cardiac repolarization phase. In conscious freely moving rats, BF2.649 (15 mg/kg, p.o.) did not impact on the main ECG parameters (PR, QRS or QT interval). Assessment of cardiovascular risk of BF2.649 (5, 10 and 15 mg/kg, p.o. or 1.5 mg/kg, i.v.) in the conscious male and female Beagle dog monitored by telemetry evidenced no effect of BF2.649 on arterial blood pressure, heart rate, the PR, the QRS, the QT and the QTc intervals. No arrhythmia or other changes in the morphology of the electrocardiogram were observed. These data suggest that BF2.649 would possess no proarrhythmic properties in the context of prolonged QT.

On the respiratory functions, BF2.649 (4 mg/kg and above, i.v.) only increased the tidal volume.

On the central nervous system, no significant effect of BF2.649 was observed at doses up to 30 mg/kg, p.o. in global behaviour and central nervous system activity screen (FOB assay). Straub tail and convulsions were observed only at high doses (60 mg/kg and above in rodents), but no impairment of motor coordination was detected. BF2.649 over the dose-range 3-60 mg/kg, p.o. was without any effect in the Barbital interaction (sleep induction) test in rats. BF2.649 did not occasion conditioned place-preference or behavioural sensitization in rats or the discriminative stimulus effects of cocaine in mice, and was not self-administered in monkeys: this is consistent with its lack of effects on dopamine release in the striatal complex and, by inference, it would not likely elicit psychostimulant's subjective effects in humans nor be abused like psychostimulants. No dependence was elicited by BF2.649 as evidenced by the lack of withdrawal signs following a sub-chronic treatment of rats.

BF2.649 was found devoid of any effects on gastric acid secretion or ulcer formation in rats.

3.1.3. Pharmacology of pitolisant in potential therapeutic indication

Studies conducted to date confirmed that pitolisant can counteract H3R agonist-mediated alterations in central histamine activity in several models.

3.1.3.1. *Effects on Sleep/wakefulness and EEG patterns in cats and mice*

The effects of pitolisant on sleep-wake cycle control have been investigated in freely moving cats (*Buda et al., 2002*) and mice (*Parmentier et al., J Neurosci. 2002, 22, 7695-711*).

Oral administration of pitolisant caused dose-dependent increase in the duration of waking in two animal species: cats and mice. Moreover, the EEG results suggest that pitolisant could improve the quality of arousal, by reinforcing e.g. the level of vigilance and attention, in the absence of any noticeable psychomotor activation which is observed with drugs like amphetamines. When given parenterally or orally, pitolisant enhances the histaminergic transmissions in brain, and thereby improves vigilance, learning and memory, and displays pro-cognitive properties.

In a model of narcolepsy, orexin-KO mice, pitolisant showed strong waking, and antinarcoleptic effects. Oral administration of pitolisant (20 mg/kg, po) significantly improved wakefulness during the awakening period, and considerably reduced the narcoleptic episodes.

On the other hand, the combination of modafinil (64 mg/kg) and pitolisant (20 mg/kg) showed a clear synergistic activity on sleep/wakefulness and narcoleptic periods in this narcolepsy model.

3.1.3.2. *Effects on Learning*

Behavioural studies have shown that pitolisant at the dose of 15 mg/kg significantly reduced (p = 0.0056) scopolamine-induced amnesia and "natural" forgetting in two mice models, suggesting that pitolisant has significant effects on learning and memory.

Two experimental models were used to confirm this hypothesis:

1 Experimental model of amnesia: In this model scopolamine is used to induce learning deficit in mice which is estimated by using the two - trial object recognition paradigm [*Ennaceur and Delecour, Behav. Brain Res., 1988, 31, 47-59*].

   This test is considered as a model of episodic memory and has been shown to be sensitive to the effects of aging and cholinergic dysfunction induced by scopolamine [*Scali et al., Neurosci. Lett., 1994, 170, 117-120; Bartolini et al., Biochem Behav., 1996, 53, 277-283*].

   A 5 mg/kg dose of pitolisant did not modify the time for mice to reach the learning criteria on the first trial whereas this time was significantly increased at a higher dose (15 mg/kg), a change which might reflect enhanced "curiosity" towards the whole novel environment.

2 Experimental model of Natural Forgetting in mice. In this test, the time spent by mice to achieve the exploration of a familiar object is shorter than the time spent for a novel object. In this model, histamine-deficient mice (Histidine-decarboxylase KO mice) showed deficiency to explore objects and to discriminate novel objects from familiar objects *(Dere et al, 2003*). The results of the study (learning test of recognition) conducted in mice suggest that the "natural" forgetting is significantly reduced by tacrine but also by pitolisant at the dose of 15 mg/kg.

These observations of a reversal by pitolisant are consistent with a large variety of observations gathered with the other H3R antagonists/inverse agonists, including the prototypical drug thioperamide in similar models [*Ghi et al., Pharmacol. Biochem. Behaviour, 1999, 64, 761-766; Molinengo et al., Pharmacol Biochem Behav., 1999, 63, 221-*

227; Miyazaki et al., Life Sci., 1995, 57, 2137-2144; Orsetti et al., Behav. Brain Res., 2001, 235-242]

This pro-cognitive effect of pitolisant might be related to the direct "arousing" effect of endogenous histamine (e.g. at the level of the cerebral cortex, hippocampus or thalamus) as well as to its capacity to increase acetylcholine-release in brain [*Blandina et al., Brit. J. Pharmacol., 1996, 119, 1956-1664*].

3.1.3.3. *Effects on Attention Deficit/ Hyperactivity Disorder (ADHD)*

Dopamine-Transporter (DAT$^{(-/-)}$) Knock-out mice have been suggested as a model for ADHD because of their behaviour. These mice exhibit high extracellular dopamine levels in brain, and are reported to be hyperactive in the open field *(Giros et al., Nature* 1996, 379, 606-12).

In this experimental model of hyperactivity, pitolisant (6 mg/kg) decreased the horizontal locomotor activity and the number of rearings, an effect similar to the paradoxical effect of amphetamine-like stimulants which are currently used in the treatment of adult or child ADHD.

However, there is an important difference in the profile of the two classes of compounds: in contrast with amphetamines and methylphenidate, pitolisant has (1) no psychomotor stimulant effect in normal animals, (2) no sympathomimetic effect leading to hypertension, (3) no abuse potential, i.e. important drawbacks which are likely leading to the imposition of a "black label" to this drug class and the present limitation for their use in European countries.

3.1.3.4. *Effects on Epilepsy Disorders*

The potential benefit of pitolisant in epilepsy has been confirmed in various models of seizure in rats and mice. Animal studies indicate that pitolisant could have a beneficial effect on typical absence epilepsy in human as well as on temporal lobe seizures, a form of seizures which is generally drug resistant.

Pitolisant significantly decreased both the number and cumulated durations of spike and wave discharges 20, 40 and 60 minutes after its administration in Genetic Absence Epilepsy Rats of Strasbourg (GAERS), a very predictive model for absence epilepsy [*Danober et al., Prog. Neurobiol., 1998,55, 27-57*]. These results suggested that pitolisant should have an effect on typical absence epilepsy in human. In kainate-induced hippocampal seizures in mice,

pitolisant (10 mg/kg) significantly reduced the cumulated duration and the number of hippocampal discharges during the first 40 min after administration. The anti-epileptic effects observed in kainate mice suggest that pitolisant could be effective on temporal lobe seizures, a form of seizures which is generally drug resistant.

3.1.3.5. *Effects on psychotic diseases*

Pitolisant was found to be effective in various models of schizophrenia such as the models of Methamphetamine-Induced Locomotor Activation, Apomorphine-Induced Climbing Behaviour and MK-801-Induced Locomotor Activation in Mice.

In these experimental models, pitolisant clearly reduces the hyperactivity induced by dopamine stimulation (methamphetamine and apomorphine models), or by glutamate inhibition (MK-801 model). These results suggest that BP2.649 could have a clinical interest in schizophrenia where glutamatergic/dopaminergic imbalance is suspected to occur.

3.1.4. Toxicology

3.1.4.1. Summary

The acute toxicity of BF2.649 has been evaluated by the intravenous route in mouse and rat, and by the oral route in mice, rats, dogs and monkeys. Repeat oral dose toxicity studies have been performed in rats and monkeys.

- Acute oral toxicity in rodents:

  BF2.649 demonstrated a good safety profile in rodents. In mice, the no effect dose was > 30 mg/kg orally and > 5 mg/kg, i.v., with a maximum non lethal oral dose of 100 mg/kg. The minimum lethal doses were 150 mg/kg, p.o. and 10 mg/kg, i.v. In rats, the no effect dose was > 50 mg/kg, orally and 12 mg/kg i.v., with a maximum non lethal oral dose of 100 mg/kg (expressed as single doses). On a mg/kg basis, the no-effect doses were ≥ 38 times higher than the highest BF2.649 dose to be studied in Phase III (i.e., 40 mg; based on a 50-kg human).

- Repeat oral dose toxicity studies in mice, rats and monkeys:

  In a 4-week repeat dose toxicity study, the dose-level of 75 mg/kg/day of BF2.649 given by oral gavage for 4 weeks to CB6F1-nonTgrasH2 mice was considered to be the No Observed Adverse Effect Level (NOAEL) and elicited slight CNS signs (mainly transient hypoactivity) in a limited number of mice. A safety margin of 38 was deduced. At 100

mg/kg/day, hypoactivity was the most frequent and remarkable clinical sign recorded, with a higher frequency in males (lasted 13 days in males and 10 days in females), associated with clonic convulsions in males (5/10) on 1 or 2 days. No convulsions were observed in females. Several cases of staggering gait and a few cases of loss of balance were noted in males and females as well as a few cases of ptyalism and reflux at dosing in males.

In rats, the 13-week and 6-month repeat dose studies showed satisfactory tolerability of BF2.649 up to 30 mg/kg/day, a dose which can be defined as the NOAEL. In the 13-week study, mortality was observed at doses ≥75 mg/kg. The cause of death could not be directly established, but a number of rats that died had lung lesions; in addition, adverse effects on the cardio-respiratory and/or CNS could not be ruled out. In the 6-month study, the 60 mg/kg/day dose showed evidence of CNS toxicity, such as convulsions and tremors without any associated brain histopathology finding. At this dose, other target organs included the adrenals, duodenum, liver, and lungs. The NOAEL dose was found to be of 30 mg/kg/day, leading to a safety margin of 19 when taking into account the sum of BF2.649 and its metabolite BP1.2526 abundant in rats but not in human (a separate trial had established the pro-convulsive potential of this metabolite at exposure levels comparable to those found at convulsive doses of BF2.649).

In monkeys, the 13-week and 9-month repeat dose studies showed no adverse effects of BF2.649 up to 5 mg/kg/day. At 12 mg/kg/day in the 13-week and 9-month studies, occasional emesis occurred only in a very limited number of monkeys. No other significant effects were recorded. Hence, this dose can be considered as the NOAEL in this species leading to a safety margin of 4.3. At a higher dose (30 mg/kg/day) in both studies, the main findings observed in several animals were clinical signs including emesis, tremors and/or occasional convulsions. Some slight changes in serum biochemistry were also reported in the 13-week study (e.g. increases in aminotransferases).

Regarding the main non-conjugated metabolite in human (BP2.951) which did not enter the brain and therefore cannot be responsible for CNS side effects, safety margin ratio calculation provided a value of 6.8 when comparing corresponding AUC at NOAEL doses in monkeys, the species having also BP2.951 as main non-conjugated metabolite, to its AUC in humans at therapeutic dose. Similar calculations made in mice and rats, two species having not BP2.951 as main metabolite, provided safety margins ratio of 3.7 and

1.3, respectively.

Regarding other main metabolites in human, BP1.8054, a glycine conjugated metabolite which was not present in toxicity species, a specific 13-week toxicity study in the rat provided a NOAEL dose at the highest oral dose tested of BP1.8054 (300 mg/kg/day). Safety margin ratio calculations provided values of at least 265 when comparing corresponding AUC at the NOAEL dose to its AUC in humans at therapeutic doses. BP1.9733, another conjugated metabolite (glucuronide) was present at significant levels in the monkey receiving BF2.649. The 9-month toxicity study in this species provided a NOAEL dose of 12 mg/kg/day and safety margin ratio calculations provided values of at least 14 when comparing corresponding AUC at the NOAEL dose to its AUC in humans at therapeutic doses.

- Carcinogenicity:

Two studies were performed:

The 6-month carcinogenicity in CB6F1 TgrasH2 transgenic mice evidenced that the Maximum Tolerated Dose was achieved, based on the clinical signs observed at the dose-levels of 75 mg/kg/day, and on the non-neoplastic histopathological findings seen in the liver and testes from animals treated at 30 mg/kg/day or 75 mg/kg/day. Following the administration of BF2.649 at all the doses investigated (including the high dose-level of 75 mg/kg/day) for 26 weeks, there were no neoplastic findings related to the test item administration in male or female mice. Consequently, under the experimental conditions of this study, the dose-level of 75 mg/kg/day eliciting a significant BF2.649 exposure (C_{max} of 2584 ng/mL and 1634 ng/mL and AUC_{0-24h} of 9896 ng/mL*h and 7228 ng/mL*h in males and females, respectively) was considered to be non carcinogenic in CB6F1-TgrasH2 mice. These can be compared to pharmacokinetic parameters of BF2.649 in humans at expected therapeutic level (C_{max} of 72 ng/mL and AUC_{0-24h} of 804 ng/mL*h) indicating safety margins of 23 and 8,8, respectively.

A 2-year carcinogenicity study in Sprague-Dawley rats investigating the dose-levels of 15, 30 and 60 mg/kg/day, by oral route was performed. Based on the clinical signs and effects on body weight gain observed in animals at the dose-levels of 30 mg/kg/day, the Maximum Tolerated Dose was achieved. Following the administration of BF2.649 at all the doses investigated for 105 weeks, there were no neoplastic findings that were attributed to the test item administration. Consequently, under the experimental conditions of this study, the dose-level of 30 mg/kg/day eliciting a significant BF2.649

exposure (mean exposure values in males for C_{max} of 182 ng/mL and AUC_{0-24h} values of 1565 ng/mL*h in males at week 104), was not carcinogenic. These values, when compared to pharmacokinetic parameters of BF2.649 in humans at therapeutic level (C_{max} of 72 ng/mL and AUC_{0-24h} of 804 ng/mL*h) indicate safety margins of 2,5 and 1,9, respectively

- Genotoxic potential of BF2.649 has been assessed in two *in vitro* systems and one *in vivo* assay. Pitolisant has been found not to be genotoxic (no mutagenic nor clastogenic) *in vitro* or *in vivo* as well as its major metabolites in rats (BP1.2526 and BP2.951) and humans (BP2.951 and BP1.8054) which were tested *in vitro* for mutagenicity *(*BP1.2526, BP2.951 and BP1.8054) and for clastogenicity (BP1.8054).
- Reproductive and developmental toxicity:

Reproductive and developmental toxicity was evaluated in a battery of studies, BF2.649 did not show any adverse effects on fertility and early embryonic development at 30 mg/kg and this dose was a NOAEL in this study ensuring a safety margin of 3.9. At higher doses, CNS clinical signs, increased post-implantation losses, and sperm alterations were reported.

In an *embryotoxicity study in rats*, maternal toxicity and foetal weight reductions were evident at 90 and 110 mg/kg/day p.o. (the highest doses tested). No treatment-related malformations were observed up to the highest dose tested, leading to a safety margin of at least 3,7.

In an *embryotoxicity study in rabbits* at doses up to 150 mg/kg/day, p.o., malformations were observed at 150 mg/kg in the presence of maternal toxicity with a frequency similar to control. The NOAEL for foetal malformations was 67 mg/kg. However, the administration of BF2.649 by oral route provided a low drug exposure in the rabbits. Therefore, additional studies were performed by intramuscular route in order to ensure significant drug levels investigating BF2.649 at the doses of 4, 8 and 16 mg/kg/day i.m. From these, the NOAEL for maternal parameters was considered to be 4 mg/kg/day and the NOAEL for effects on embryo-foetal development was considered to be 8 mg/kg/day, leading to a safety margin of 1.3. There was no evidence of a specific dysmorphogenic effect following treatment of the dams with BF2.649.

During the *pre- post-natal development toxicity study*, BF2.649 induced clinical central signs, dystocia during delivery in pregnant rats, increase in dead-born pups and some major malformations at the dose of 90 mg/kg. Some alterations of the maternal nursing

behaviour and retardation in the physical and motor development of F_1 generation were reported at 52 mg/kg. The 30 mg/kg/day dose was considered as the NOAEL, leading to a safety margin of 3,9.

In a *juvenile toxicity study in rats*, BF2.649 doses of 9, 21 and 48 mg/kg were administered daily by *oral route* from postnatal day (PND) 7 to postnatal day 70. At the two high doses, some premature deaths occurred and CNS signs and ptyalism were observed. There were also histopathologic changes in the lung and larynx (mostly foreign body granulomas) at highest doses. No effect on tibia growth and pre-weaning development, sexual maturation, pairing, mating and fertility, learning and memory, motor activity or reflexes were evidenced. Therefore, the NOAEL dose was established at ~ 9 mg/kg/day corresponding to a low BF2.649 exposure characterized by AUC_{0-24h} values of ~ 100 ng/mL.h and ~ 30 ng/mL.h on PND7 and PND34, respectively. As lung disorders could be due to the penetration of the test item in the airways during gavage, additional investigations were initiated using the intraperitoneal route to avoid these phenomenons and to ensure a higher exposure.

Intraperitoneal administration of BF2.649 daily at 15 or 30 mg/kg/day or twice daily (8 hours apart) at 30 mg/kg/day in the juvenile Sprague-Dawley rat from 7 days of age (PND7) up to 10 weeks of age (PND70) induced a dose-related mortality and convulsive episode at 30 mg/kg/day and 2x30 mg/kg/day. There was no effect on the subsequent development neither on the reproductive function. Pathological changes were limited to microscopic changes in the lungs which consisted of a dose-dependent increase in alveolar macrophages mainly of minimal severity at all doses and both sexes an with a very limited occurrence at the low dose, and pigment in histiocytes in males treated at 30 mg/kg/day and in males and females treated at 2x30 mg/kg/day. Following a treatment-free period, increased alveolar macrophages appeared reversible, but pigmented histiocytes were still observed in males and females previously treated at 30 or 2x30 mg/kg/day after 9 weeks of treatment-free period. These findings were considered non adverse.

Hence the NOAEL was set at 15 mg/kg/day corresponding to an AUC_{0-24h} of 1159 ng/mL*h at PND70 and to a C_{max} value of 395 ng/mL. When compared to exposures of BF2.649 in humans at therapeutic dose (AUC_{0-24h} of 804 ng/mL*h and C_{max} of 72 ng/mL) these values indicate safety margins of 1.4 and 5.5, respectively. The total AUC_{0-24h} of the two major active entities (BF2.649 and its metabolite BP1.2526 abundant in rats) at

PND70 is 1806 ng/mL*h and corresponding C_{max} value is 747 ng/mL. When these values are compared to pharmacotherapeutic level (AUC_{0-24h} of 832 ng/mL*h and C_{max} of 74,4 ng/mL) safety margins of 2., and 10, respectively, are obtained.

Regarding the main non-conjugated metabolite in human (BP2.951), safety margin ratio calculations provided values of 1.3, higher than 1.3, of 24 and 0.4 when comparing corresponding AUCs in rats (fertility and early embryonic development and pre and post-natal development), in rat embryofoetal toxicity, in rabbit embryofoetal toxicity and in rat juvenile toxicity at NOAEL doses, respectively, to its AUC in humans at therapeutic dose.

Regarding a main metabolite in human (BP1.8054, a glycine conjugate) which was not present in embryotoxicity species receiving BF2.649, a specific *embryotoxicity study in rats* performed on BP1.8054 provided a NOAEL dose at the highest oral dose tested (300 mg/kg/day). Safety margin ratio calculations provided values of at least 270 when comparing corresponding AUC at the NOAEL dose to its AUC in humans at therapeutic dose.

No drug abuse liability as well as no dependence potential was evidenced for BF2.649 in several *in vivo* models in rodents and monkeys.

3.1.5. Conclusion

Pharmacological studies have confirmed the potential interest of pitolisant to be tested in excessive diurnal sleepiness in narcolepsy, Parkinson's disease, OSA, and dementia with Lewy's bodies. Safety pharmacology performed on vital functions did not show any potential major adverse events on CNS, cardiovascular and respiratory functions.

Exposure in animals showed that pitolisant is well absorbed orally and largely distributed in its target organ, the brain.

The main metabolites and metabolism pathways have been identified. None of these main metabolites were shown to be active.

3.2. Summary of relevant clinical studies

A relatively large diversity of CNS applications were initially explored taking into account the effects of pitolisant on animal models and the fact that no other agent of its pharmacological class had been tested clinically previously. Accordingly were performed

pilot trials in epilepsy, ADHD, schizophrenia, dementia as well as in pathologies characterized by EDS (narcolepsy, OSA, Parkinson's disease).

In most trials progressive individual titration was adopted, as is the case for other narcolepsy medications, to adapt to individual sensitivity of patients to the waking (and insomnia-eliciting) effects of the drug.

3.2.1. Phase I studies

A total of 14 pharmacokinetics studies have been conducted and included a total of 225 adult subjects (23 females) and 14 patients suffering from photosensitive epilepsy.

In all studies, serum and urine pitolisant and BP2.951 (its major inactive phase I metabolite) were assayed by using mainly a validated HPLC/MS/MS technique (LOQ: 1 ng/ml).

In addition, BP1.8054 and BP1.9733 (another major inactive metabolites (phase II): glycine and glucuronide conjugate of pitolisant phase I metabolite) were quantified in adult (young, elderly, impaired renal and hepatic patients) and children serum samples from various clinical studies. To this aim, bioanalytical methods were developed and validated according to guideline EMEA/CMPH/EWP/192217/2009 Rev.1.

As no suitable intravenous formulation of pitolisant was available for human use, all these studies were conducted with pitolisant tablets taken orally in a once-a-day manner. Single oral doses from 1 to 240 mg were evaluated in healthy volunteers as well as 9 and 28 days repeated dosing with 40 to 50 mg daily. Metabolism and elimination of pitolisant were assessed through a mass balance study with a single 20 mg radio-labelled pitolisant (^{14}Carbon) administration. Results of this study should be interpreted taking into account *in vitro* incubation of pitolisant with liver microsomes and hepatocytes from rat, dog, monkey and human with potential metabolites identified using LC/MS/ESI$^+$. Effect of age, renal and hepatic impairment on pitolisant PK profile was assessed. Interactions with food, grapefruit juice, itraconazole, paroxetine, rifampicine and olanzapine were evaluated. Protein binding was assessed *in vitro* and plasma/blood partition was assessed both in vitro and ex vivo.

Overall, this programme provided a broad identification of pitolisant pharmacokinetic profile after oral administration in adults. No i.v. formulation has been developed for human use, absolute bioavailability and extent of first pass effect are not accurately known, although the Mass Balance study indicates at least 88% oral bioavailability.

Available data show that pitolisant is well and rapidly absorbed by oral route. It is extensively

metabolized by the CYP450 system (pitolisant is a substrate for CYP3A4 and CYP2D6) and conjugation enzymes into numerous inactive metabolites mainly excreted in urine. Pitolisant is not inducer of these catabolic systems at therapeutic concentrations. Inhibitors and activators of CYP450 system have a modest impact on pitolisant PK profile. Pitolisant is bound to around 90% to plasmatic proteins, distributes equally between plasma and blood cells and its apparent volume of distribution is rather large.

Pitolisant apparent terminal elimination half-life ranges between 10 to 12 hours and is dose independent at doses up to 40 mg oad, suggesting that single administration in the morning at adequate dosage is enough to ensure a full day coverage without high levels at night. A population PK analysis of pooled data of healthy subjects (male and female, young and elderly) was performed and showed that i) pitolisant could be modelled according to a bi-compartmental model with a 0-order absorption, ii) pitolisant $t_{1/2}$ was within a range centered on 10-11 h. This means that, considering a once daily administration, steady-state should be reached within less than one week; and iii) CL/F was found to decrease with both pitolisant dose and duration of administration. In other words wake promotion during day may not compromise nocturnal sleep. Old age has a mild effect on pitolisant PK profile whereas moderate hepatic impairment decreases pitolisant catabolism. The consequences of renal impairment appear to be less substantial. In rats, pitolisant crosses the placenta barrier and is excreted in milk (Study QBR117544 BIP/06)..

As evaluated in humans, pitolisant pharmacodynamic profile is on line with what has been reported in preclinical studies. It improves vigilance status and decreases propensity to fall asleep and improves attention. It is devoid of any amphetamine-like psychostimulant potential and of any drug abuse potential.

A population PK study was developed and is the support for determination of optimized PK blood sampling times and the sample size required for a population PK analysis planned in the present study.

3.2.2. Phase II Studies

3.2.2.1. Brief Summary

Nine phases IIa proof of concept studies are completed:

- One in photosensitive epileptic patients (Study P03-06)

- One in Obstructive Sleep Apnea Syndrome patients (Studies P04-01 and P05-01
- Two in narcolepsy (Studies P05-03 and P06-06)
- One in Obstructive Sleep Apnea Syndrome patients (Study P05-01)
- One in Parkinson disease-treated patients (Study P05-05)
- One in Attention Deficit with Hyperactivity Disorder (ADHD) patients (Study P05-07)
- One in pharmaco-resistant epileptic patients (Study P04-07)
- One in hospitalized schizophrenic patients (Study P04-08) is discontinued
- One in Lewy's bodies dementia (LBD) patients (Study P05-08)

The designs of these studies are detailed in the Investigator's Brochure.

The studies of pitolisant in daytime sleepiness in various conditions are summarized as follows

Results obtained in 22 narcoleptic patients (P05-03) showed that pitolisant was effective in reducing the excessive diurnal somnolence of narcoleptic patients at the dose of 40 mg/day compared to placebo. The reduction of somnolence was of 4.9 points on the ESS score which is equivalent to the results obtained with psychostimulants such as modafinil. A level of normal daytime sleepiness was reported by 40% of patients, this result was confirmed by the other criteria of sleepiness evaluated on the patient's diary. The treatment was well tolerated. The main adverse events reported were moderate in intensity and did not lead to stop the treatment. The most frequent were headache, nausea and insomnia. The occurrence of these adverse events seems to be related to high plasmatic levels.

In another study (P06-06) to confirm that pitolisant was effective to reduce excessive daytime sleepiness in narcoleptic patients, the improvement of wakefulness measured by ESS indicated a reduction of 4.8 points. This value was equivalent to the results obtained in P05-03, and was also similar to that published with Modafinil and Sodium oxybate. In this study, the exposure of 26 patients with individual titration from 10 mg to 40 mg was well tolerated.

Out of the 26 patients enrolled in the trial and who completed the first 28-day period of pitolisant administration, 18 patients, entered in the 3-month extension period and completed the 3-month treatment period. Either, 11 of the 18 patients entered in the 9-month extension period, 10 of them completed the 9-month treatment.

Results obtained in 25 patients with Parkinson's disease (P05-05) showed a marked reduction

of daytime sleepiness with pitolisant as compared to the placebo period. A tendency to reduce the motor manifestation was also noticed. The tolerance was good.

One Double Blind period IIb clinical study (P07-02) dose-ranging study in Parkinson's disease was completed in 107 patients in France and Germany. This study included 5 groups of treatment: Placebo, and pitolisant: 5-10-20-40 mg/day for 28 days. The main results on the main criterion showed that the effective dose was the dosage 20 mg (p = 0.0357). The tolerance of the product was good and comparable between the different groups of patients.

Based on these results, the 20 mg pitolisant dose was chosen as the significant effective dose for the phase III development program on EDS in PD patients.

3.2.2.2. Two pilot studies referring to the Sleep Apnea Syndrome

3.2.2.2.1. Single blind, controlled versus placebo study of pitolisant in patients suffering from Obstructive Sleep Apnea Syndrome with daytime somnolence (P04-01 pitolisant) (P. Levy – Grenoble)

Twelve patients with confirmed diagnosis of OSA by PSG were included in this multicenter study (3 centres in France). The design of the study was single blinded comparative versus placebo. Patients received placebo during the 2 first days, then a 3-day treatment of pitolisant at the dose of 40 mg/day followed by a 2-day observation period with a placebo-treatment.

The efficacy of pitolisant on sleepiness and sleep was evaluated by both sleep diary and Epworth sleep scale.

The number of diurnal sleepiness episodes (DSE) was evaluated following each period. The mean absolute change before and after pitolisant treatment corresponded to 38% relative improvement.

Among the 12 patients included in the study, 8 reported some diurnal sleeps on their sleep diaries before treatment. The effect of pitolisant allowed suppressing all sleep needs in these 8 OSA patients.

The mean value of the Epworth Sleepiness Scale (max score 24) showed an improvement in the absolute change of -5.8 ± 4.4 between before and after treatment, corresponding to 38% improvement from baseline value. It is interesting to note that all patients were improved, 5 patients rating the scale less than 8 after pitolisant treatment (threshold considered as normal).

Effect of pitolisant on Epworth score (Study P04-01)

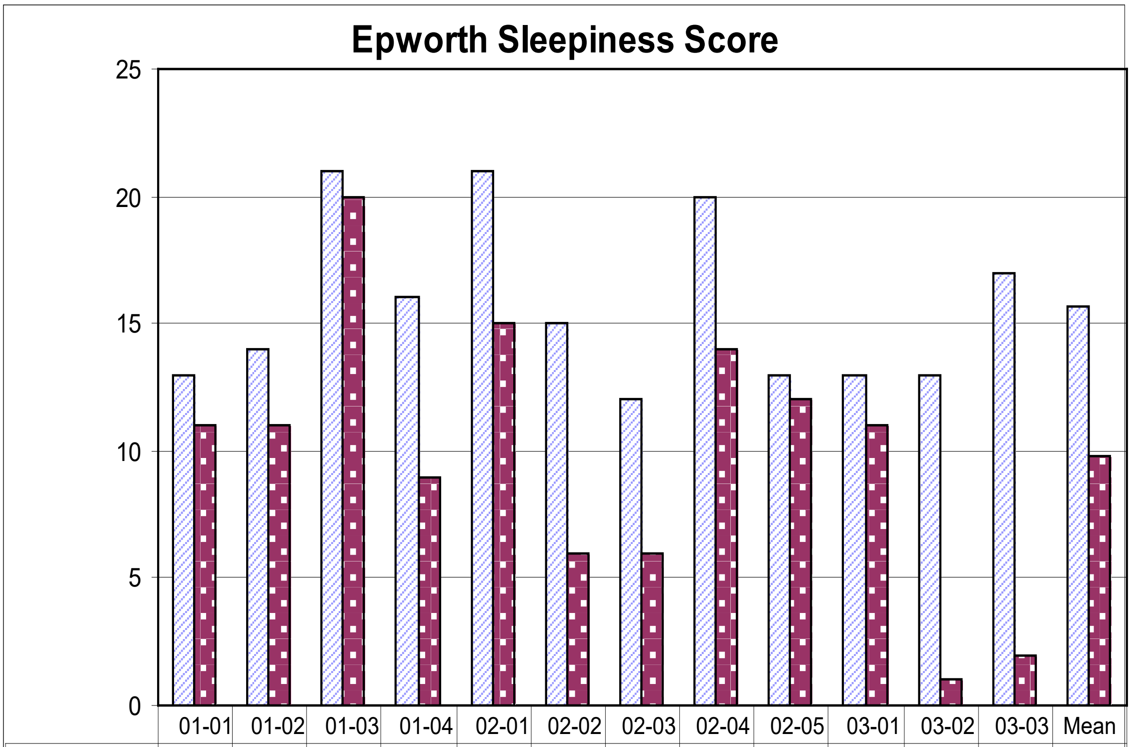

	01-01	01-02	01-03	01-04	02-01	02-02	02-03	02-04	02-05	03-01	03-02	03-03	Mean
Epworth BL	13	14	21	16	21	15	12	20	13	13	13	17	15,7
Epworth Final	11	11	20	9	15	6	6	14	12	11	1	2	9,8

Discussion:

In this study, 3 days of 40 mg of pitolisant oral treatment seems to improve the sleepiness of the OSA patients as measured by the Epworth score, and the daily patient diary.

The blood level of pitolisant was measured in each patient. Regression analysis between pitolisant level and the efficacy assessed either by mean change in DSE or in ESS, showed a positive correlation.

The efficacy was better when the level of pitolisant was higher. However, considering the small number of subjects in this analysis, it was not possible to draw any firm conclusion at this stage of development.

The tolerance to pitolisant was good. During the study, no serious adverse events were reported, 4 patients reported a total of 9 adverse events (rhino sinusitis, weight gain, viral rhinitis, thoracic oppression sensation, yawning).

Their intensity was rated as mild and treatment was not discontinued. Five patients out of 12 reported insomnia during the night following the first intake of 40 mg of pitolisant.

Nevertheless, this undesirable effect was not described as disturbing by the patients.

Furthermore this effect tended to fade away with the treatment continuation.

Conclusion

Overall, this first study according to a single blind design shows that a 3 day-treatment by pitolisant at a dose of 40 mg par day is able to improve ESS score, to decrease the number of DSE, and to suppress diurnal sleep need in patients suffering from a severe OSA. Globally, the tolerance profile of pitolisant was good.

3.2.2.2.2. Effect of pitolisant on vigilance and polysomnographic parameters in patients with Obstructive Sleep Apnea Syndrome (P05-01 BF2.649)

Since the first study in this indication (P04-01) showed preliminary good results on sleepiness improvement in OSA patients, this second study was designed to evaluate quantitatively the effect of pitolisant using the OSleR test (Oxford Sleep Resistance test, modified maintenance of wakefulness test), and the Epworth Sleepiness Scale (ESS) in patients with moderate to severe OSA, eligible but not yet treated with nasal continuous positive air pressure (nCPAP). The effect of pitolisant was also assessed on nocturnal polysomnography (PSG), where respiratory and sleep events are recorded. This study was single blind with two treatment sequences, the patients receiving placebo during one week, then pitolisant (40 mg/d) during the second week. The evaluations (ESS, OSleR test and PSG) were performed at baseline, and at the end of each treatment week.

Results

From 21 recruited patients, 20 reached the normal end of the trial (2 weeks). The characteristics are: Mean age: 51,33 years [25-68], Body Mass Index (BMI): 30,24 (SD=3,83 [22, 36]), OSA Mean duration: 8,5 years (SD=9,15 [1, 38]), AHI: 54,95 (SD=21,52 [20, 91]).

Regarding the main endpoint:

(1) OSleR test: the mean values of OSL_0 (before placebo), OSL_1 (after placebo week) and OSL_f (after pitolisant treatment week), the mean changes OSL_f-OSL_1 and OSL_f-OSL_0 highlight a significant improvement after pitolisant treatment (see the following table). By considering the end of the placebo period as the reference baseline value, a mean improvement of 6,79 (95%CI= [1,90, 11,68] was observed ($p < 0,01$).

	Mean	95%CI	Count
OSL_0	31,43	[26,49, 36,36]	(21)
OSL_1	32,38	[26,61, 38,16]	(21)
OSL_b	31,90	[26,85, 36,96]	(21)
OSL_f	39	[37,72, 40,28]	(19)
OSL_f-OSL_1	5,89	[0,37, 11,42]	(19)
OSL_f-OSL_0	7,68	[2,79, 12,58]	(19)
OSL_f-OSL_b	6,79	[1,90, 11,68]	(19)

(2) Epworth Sleepiness Scale (ESS): A significant mean change (ESS_f –ESS_b) was found between the end of pitolisant treatment (ESS_f) and the baseline pooled value (ESS_b) of -4,36, 95 % CI= [-5,86, -2,85], p < 0,05. The other mean changes based on ESS_0 or ESS_1 separately provide the same results leading to the same conclusion. Therefore, a significant decrease of the ESS has been demonstrated with pitolisant treatment.

	Mean	95%CI	Count
ESS0	14,14	[13,03, 15,25]	(21)
ESS1	11,05	[9,11, 12,98]	(21)
ESSf	8,24	[5,98, 10,49]	(21)
ESSb	12,60	[11,21, 13,99]	(21)
ESS1-ESS0	-3,10	[-4,58, -1,61]	(21)
ESSf-ESS0	-5,90	[-7,57, -4,24]	(21)
ESSf-ESS1	-2,81	[-4,50, -1,12]	(21)
ESSf-ESSb	-4,36	[-5,86, -2,85]	(21)

As to polysomnography (PSG), for each parameter, the mean difference between the end of pitolisant treatment and the end of placebo treatment has been analyzed. Two parameters, awakening intra sleep, and the percentage of the total period of sleep were characterized by a significant change.

Regarding the secondary endpoint based on patient sleep diary, the number of EDS episodes and the total number of EDS + diurnal sleep episodes extracted from the sleep diary were significantly decreased between before and after pitolisant treatment.

The safety profile was very good. No serious adverse events were observed. No patient required pitolisant treatment to be discontinued.

The relation to the study product was well ascertained, and considered as being *plausible* by the physician for three patients: obs. n°203 (nausea), obs. n°201 (epigastralgia), and obs. n°202 (insomnia) during the pitolisant period, that did not lead to stop the treatment.

Laboratory workup and electrocardiograph results showed no pathological changes.

From these findings, it could be ascertained that pitolisant provide reasonable assurance of a good safety profile.

Conclusion

During this single blind study in obstructive sleep apnea (OSA), a significant improvement of excessive diurnal sleepiness was noted.

This improvement was demonstrated on all retained criteria, compared before and after pitolisant treatment, more particularly on the primary criterion assessed by the OSleR test as well as on ESS, patient's diary, and intra sleep awakening (polysomnography).

Pitolisant daily, orally administered during 7 days at 40 mg was safe and well tolerated. No serious adverse event was reported in 21 patients. Globally, the safety profile was very good.

3.2.2.3. Minimum effective dose-finding study of BF2.649, in patients with moderate to severe Obstructive Sleep Apnea, experiencing Excessive Daytime Sleepiness (EDS) despite regular use of nCPAP, and patients having refused this therapy. Randomized, double blind study with BF2.649 (5-, 10-, 20-, 40-mg/d), or placebo (P09-16 BF2.649)

Study P09-16 / BF2.649
Flow-Chart

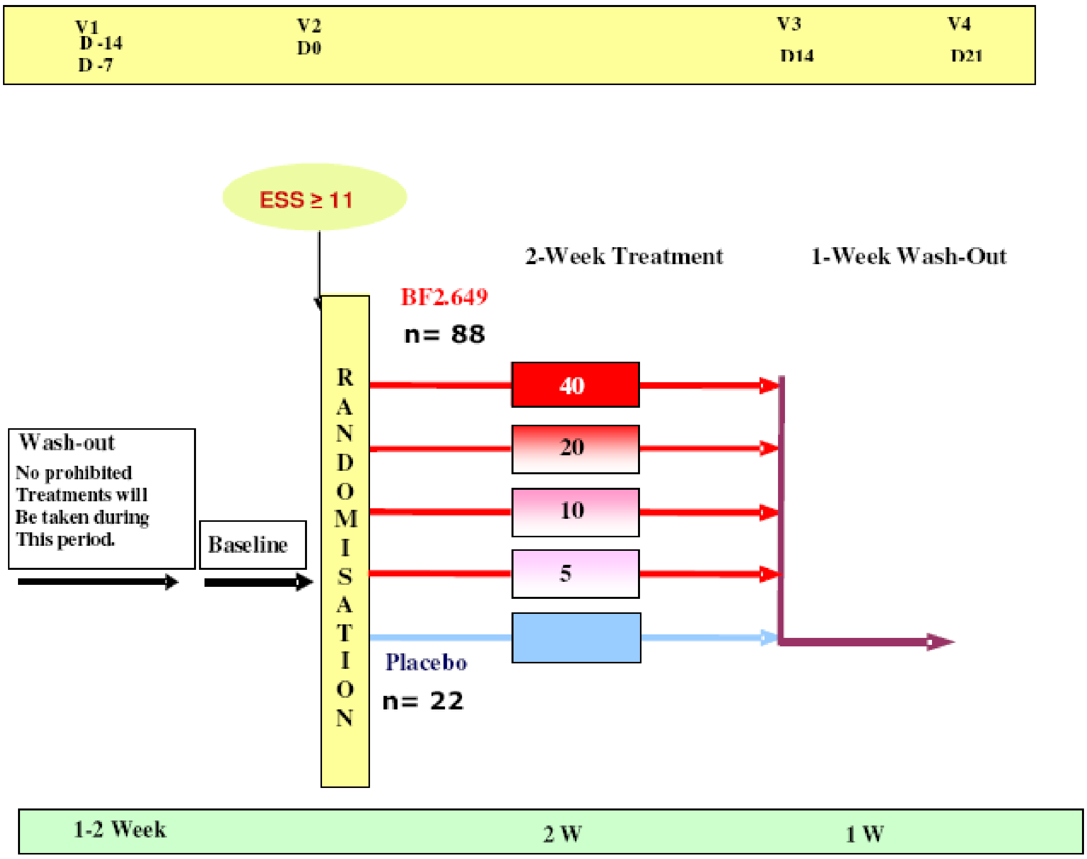

Overall, 116 patients were included and 110 completed the study. One randomized patient was not taken into account for the efficacy intent-to-treat analysis as he did not take any study treatment.

BF2.649 given at doses of 5, 10, 20, or 40 mg OD decreased daytime sleepiness assessed with ESS score and the effect statistically significantly increased with the dose (p=0.0003, Step-down Type 2 Linear Contrasts). The increase was linear up to the 20 mg OD dose. The minimal effective dose of BF2.649 on ESS score (primary endpoint), as well as ESS

responders rate, DSAR and CGI-C (secondary endpoints) was 20 mg OD. The 40 mg OD dose did not seem to have any additional effect on most endpoints. BF2.649 was well tolerated in this population of patients with Obstructive Sleep Apnea and Excessive Daytime Sleepiness.

3.2.3. Phase III studies

To follow up on the good results in OSA provided by phase II trials, two phase III studies were designed to assess the efficacy and safety of pitolisant at doses up to 20mg OD in comparison with placebo during 12 weeks for patients who experience residual sleepiness despite regular nasal Continuous Positive Airway Pressure (nCPAP).

The main endpoint of these studies was to assess the efficacy on excessive daytime sleepiness (based on the score of the Epworth Sleepiness Scale, ESS) and safety of BF2.649 given at 5, 10, or 20 mg per day *versus* placebo. The EDS evaluations (ESS and OSleR test) were performed at baseline, and at the end of the double blind period.

At end of the double blind period the patients could enter an open label period and received pitolisant up to 20mg OD for 40 additional weeks to assess pitolisant efficacy maintenance and safety.

Double blind flow-Chart

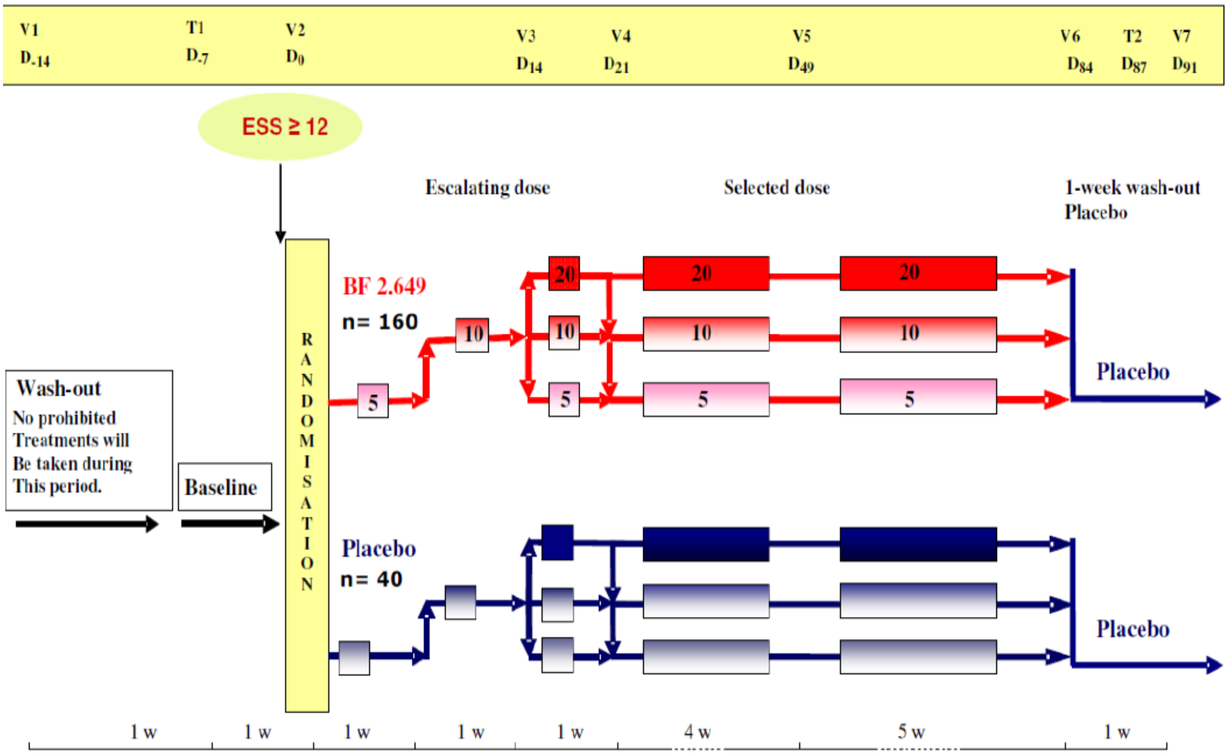

Main Endpoint:

The ESS mean value was compared between the treatment groups at every visit (Mean ± SD, (SE). Baseline ESSb and final ESSf values were calculated as the mean of ESS at visit 1+2 and 5+6, respectively.

Secondary endpoints:

- Percentage of ESS treatment responders;
- Severity of Excessive Daytime Sleepiness (EDS) as measured by the patient sleep diary at V2 and V6;
- Oxford Sleep Resistance Test (OSL) at V2 and V6;
- European Quality of life questionnaire (EQ-5D) at V2 and V6;
- Leeds Sleep Evaluation Questionnaire (LSEQ) at V2 and V6;
- Cognitive Function Test: Trail Making Test (TMT) Parts A & B;
- Illness severity as measured by the Clinical Global Impression of Severity and of Change (CGI-S and CGI-C) at V2 and V6;
- Beck Depression Inventory (BDI) at V2 and V6;
- Pichot Fatigue Scale (PSF) at V2 and V6.

3.2.3.1. P09-08 Harosa 1 preliminary results

244 moderate to severe patients using nCPAP and still complaining of EDS were recruited and randomized on pitolisant (n=183) or placebo (n=61) and constitutes the Full Analysis Set (FAS) population and Extended to Treat (EIT) population. The EIT population and Safety population analyzable samples were identical and constituted 244 patients. The Intent to Treat (IT) was n=243, where one patient (4218) under the pitolisant arm was excluded for not having an ESS value at baseline (V2). Thirty-six patients were found with a major deviation (7 placebo, 29 pitolisant; refer to Table 1.1.3 for reason). The Per Protocol (PP) sample is constituted by 200 patients (52 placebo, 148 pitolsant). Forty-four patients were excluded from the PP (refer to Table 1.1.3 for explanations). Twelve patients (3 placebo, 9 pitolisant) had a premature termination of the trial.

Pitolisant, in moderate to severely affected OSA patients using nCPAP and still complaining of EDS, showed a statistically significant improvement in decreasing ESS score when compared to placebo. The study preliminary results also confirmed pitolisant efficacy on

other EDS criteria such as OSLER test, ESS responders rate and Clinical Global Impression. Pitolisant overall good safety profile was also confirmed in this study.

3.2.3.1. P09-09 Harosa 2 preliminary results

268 patients were recruited and randomized on pitolisant (n=201) or placebo (n=67). One patient (4003) was not treated and withdrew from the study after inclusion, thus the Full Analysis Set (FAS) population and Extended to Treat (EIT) population constitute 267 patients with pitolisant (n=200) and placebo (n=67). The Intent to Treat (IT) population and EIT population and Safety population analyzable samples were identical and constituted 267 patients.

Pitolisant, in moderate to severely affected OSA patients not using nCPAP and complaining of EDS, showed a statistically significant improvement in decreasing ESS score when compared to placebo. The study preliminary results also confirmed pitolisant efficacy on other EDS criteria such as ESS responders rate and Clinical Global Impression. Pitolisant overall good safety profile was also confirmed in this study.

3.2.4. Summary of known and potential benefits and risks

The symptoms of Excessive Daytime Sleepiness resulting from the untreated sleep apnea are thought to have serious consequences during activities where alertness is dangerous such as driving, leading to an increased risk of road traffic accidents. The EDS in sleep apnea patients is also associated with a reduction in quality of life and an impairment of cognitive function.

The study medication, pitolisant (BF2.649), is a highly potent and selective antagonist and inverse agonist of histaminergic receptor type 3 (H_3R). Pitolisant was found to enhance the histaminergic transmissions in the brain and thereby to improve vigilance and alertness.

The exposure to pitolisant in healthy volunteers was assessed in six pharmacokinetic studies in which 66 subjects received single oral dose of pitolisant from 5 to 120 mg and during 28 days with 50 mg once daily. The compound was well tolerated up to oral single dose of 90 mg, however the dose of 120 mg led to irritability. The repeated administration (28 days up to 50 mg/day) was well tolerated. Pitolisant appears clearly as a once – a – day drug with a plasma half-life between 10 and 12 hours. Upon repeated administration, the steady state of pitolisant is achieved after 5-6 days. The elimination is mainly achieved through an inactive

metabolite (BP2.951) that is excreted in the urine

The beneficial effect of pitolisant in Excessive Daytime Sleepiness associated with Obstructive Sleep Apnea syndrome has already been demonstrated in two proof of concept clinical studies, in which 33 patients participated. In one study in which 22 patients were involved, pitolisant given during 7 days reduced significantly the score of the Epworth Sleepiness Scale (ESS) by more than 4 points, when compared with baseline score. Because the reduction of 3 points is considered as an indication of clinically significant response in several studies encountered in literature, the reduction of more than 4 points provided by treatment with pitolisant is clinically meaningful for OSA patients. These results were confirmed in two phase III studies including moderate to severe OSA patients receiving nCPAP (P09-08) or without nCPAP (P09-09) treated for 12 weeks with pitolisant 20mg OD or placebo. These trials included 244 (P09-08) and 267 (P09-09) patients. The ESS score was reduced in the pitolisant group of 6 points compared to baseline score in both studies and was statistically significant when compared to the placebo group.

The other benefits provided by pitolisant treatment resulted from the analysis of data collected with the daily sleep diary, which showed a tendency to suppress diurnal sleep and reduce the sleepiness of the evaluated patients. OSA patients suffering from EDS improved significantly about alertness as confirmed by the OSleR test (Oxford Sleep Resistance test, modified version of maintenance of wakefulness test), thus this finding prove that pitolisant could be relevant for the OSA patients to improve the vigilance mostly involved in traffic road accidents.

Until now, the safety profile of this product was found to be very satisfactory in the patients treated for excessive daytime sleepiness associated to Obstructive Sleep Apnea, or to a Parkinson's disease or to narcolepsy. Pitolisant is currently in its development phase in OSA. As a consequence all the adverse events are not known.

However, during the previous studies, which enrolled approximately 1094 patients,

The most frequent adverse drug reactions (ADRs) reported with pitolisant were insomnia (8.4%), headache (7.7%), nausea (4.8%), anxiety (2.1%), irritability (1.8%), dizziness (1.4%), depression (1.3%), tremor (1.2%), sleep disorders (1.1%), fatigue (1.1%), vomiting (1.0%), vertigo (1.0%), dyspepsia (1.0%), weight increase (0.9%), abdominal pain upper (0.9%). These effects were all transient and most declined spontaneously when the treatment was stopped. No additional significant side effect associated with pitolisant over the long term was

observed.

Regarding the mode of administration planned for this study (one intake in the morning) and clinical relevant benefit despite some adverse drug reactions encountered during the previous studies, the benefit risk ratio could be considered as advantageous for use of pitolisant. According to the tolerance of the product evaluated in the completed studies, it is recommended to start the treatment of EDS with an escalating dose regimen to optimize the tolerance up to the stabilized dose.

4. RATIONALE OF THE CURRENT STUDY

Excessive daytime sleepiness (EDS) is a major complaint in patients with obstructive sleep apnea syndrome (OSA) defined as sleep-related breathing disorder (SRBD) with full or partial occlusion of the upper airway during the sleep. OSA afflicts at least 2-4% of the adult population (at least 4% of males and 2% of females)[1,2].

The proposed mechanism for EDS in OSA patients is sleep disturbance and loss of sleep resulting from microarousals produced by increased ventilatory effort[3,4].

Patients may be personally unaware of their snoring and breathing pauses during sleep, but the consequences of daytime hypersomnolence such as deficit in attention or vigilance (road accident, accident at work)[5], impaired short-term memory and reduced capacity to sustain concentration or focus are often essential to rational clinical decision about whom to treat [6] and the American Academy of Sleep Medecine recommends polysomnography to diagnose and to determine the severity of obstructive sleep apnea.

Nasal continuous positive airway pressure (nCPAP) is the treatment of choice for most patients with OSA[7,8,9,10]. When used properly, nCPAP reduces apnea and hypopnoea rate, often normalizes arterial blood oxygen saturation, decreases sleep fragmentation, and improved sleep quality. As a result, alertness, mood, cognitive function, and quality of life improve[11]. Furthermore nCPAP reduces risks of cardiovascular events in obstructive sleep apnea patients.

However not all patients find CPAP as the ideal treatment. Like all chronic therapies, compliance with CPAP is difficult to maintain over and between 5% and 50% of patients refuse CPAP therapy initially, and another 12% to 25% may discontinue CPAP within 3 years (Akram Khan Sleep review , April 2008).

In a long term study of the use of nCPAP in 137 sleep apnea patients during 8 years, Bizieux-Thaminy *et al* outlined that among 30 patients out of 137 (22%) who stopped nCPAP, 10% of them stopped nCPAP within the 6 first months, 30% within the year, and 67% within 3 years.

The most common problem rated as severe was nasal stuffiness, sensation of cold air, noise and mask pressure. CPAP therapy correlated negatively with CPAP use, indicating that these were problems that the patients blamed for a lack of CPAP use[12].

Patients with obstructive sleep apnea syndrome can experience residual excessive daytime sleepiness despite regular use of nasal Continuous Positive Airway Pressure therapy. As 230,000 obstructive sleep apnea patients are currently treated in France by CPAP, more than 13,800 of them might suffer from residual excessive sleepiness [*Pepin J. L, Viot-Blanc V, Escourrou P, Racineux J-L, Sapene M, Levy P, Dervaux B, Lenne X, Mallart A, Prevalence of residual excessive sleepiness in CPAP-treated sleep apnea patients: The French multicentre study. Eur Resp J 2009; 33: 1062-1067*]. These patients, who often complain of an important EDS which represents a serious embarrassment in their everyday life, also claim a symptomatic treatment.

In view of these issues, alternative modality such as pharmacologic symptomatic treatment may be of great meaning to treat persistent residual excessive daytime sleepiness, which leads to a decreased quality of life. It is within this framework that pitolisant, novel selective histamine H_3 receptor antagonist/agonist inverse which exhibits strong waking promoting effects and a good tolerance in a number of pre-clinical and clinical studies, appeared completely indicated.

Experimental evidence for enhanced wakefulness induced by H_3R blockade was previously reported with thioperamide, the prototypic H_3R antagonist [*Lin et al., Brain Res. 1990, 523: 325-30*]. Similar vigilance - and attention-promoting effects were described more recently with the antagonist ciproxifan [*Ligneau et al., J. Pharmacol. Exp. Ther. 1998, 287: 658-66*].

Improvement of learning deficit and memory by an H_3 antagonist was also demonstrated experimentally in mice [*Miyazaki et al., Life Sci. 1995, 57: 2137-44; Meguro et al., Pharmacol. Biochem. Behav. 1995, 50: 321-5; Onodera et al., Naunyn-Schmiedeberg's Arch. Pharmacol. 1998, 357: 508-13*]. The effects of pitolisant on sleep/wakefulness, and EEG patterns have been investigated in cats and mice. It has been shown that pitolisant increased markedly the duration of waking in these two animal species. Moreover, the EEG results suggest that pitolisant could improve the quality of arousal, by reinforcing e.g. the level of

vigilance and attention, in the absence of any noticeable psychomotor activation that is observed with drugs like amphetamines.

In healthy volunteers, the clinical and biological tolerance of pitolisant was excellent with single oral dose between 1 mg and 90 mg.

Pitolisant was shown to produce an increase in vigilance on critical Flicker Fusion Threshold, and on spectral EEG analysis: increase of rapid rhythms (beta frequencies > 13 Hz) with concomitant decrease of alpha and theta frequencies.

The previous phase II and III clinical studies (P04-01, P05-01, P09-16, P09-08, and P09-09) have demonstrated that pitolisant at doses up to 20mg OD significantly decreases the diurnal somnolence. It seems the effect increases with dose. Therefore, our study will assess in patient, treated with nCPAP or not, pitolisant effect at doses up to 40mg OD.

ESS measures sleepiness recently *[Johns MW Sleep 1992]*. It is a simple self administered questionnaire with eight items which is shown to provide a measurement of the subject's general level of daytime sleepiness in several real life situations. It scores the tendency to fall asleep (from 0-3) during eight everyday situations. It scores from 0-24, and abnormal somnolence is considered as a value greater than 10. ESS has proven a very satisfying reliability, and apparently the best sensitivity and specificity among all the other instruments, even those developed in this specific pathology. A final important characteristic of this instrument is its reproducibility with naturalistic conditions.

The primary endpoint of this study will be measured by the change in Epworth Sleepiness Scale. Its reliability and internal consistency have been demonstrated.

Furthermore, because of the subjectivity of ESS, a secondary criterion will support it with quantitative data related to the diurnal sleepiness: the home patients' diaries.. These diaries will be filled during 3 sequential days of the week preceding each visit and will record the number and duration of sleep and sleepiness episodes. By that mean the EDS change measured by ESS will be confirmed by quantitative measurements of sleepiness.

The main criteria of selection for the OSA population will be: ESS ≥12

The treatment regimen for this study will start at V2 with an escalating dose starting at 10 mg OD pitolisant or placebo on the first week followed by 20 mg OD on the second week. At V3, the posology is increased to 40 mg/d if the tolerance is acceptable. If the study product is not well tolerated (i.e. troublesome insomnia), the patient will continue taking 20 mg/d pitolisant or placebo, or may be the posology will be eventually reduced at 10 mg/d until V4. At V4, the

investigator will decide to maintain, or to reduce the dose according to the tolerance of the study drug. No increase of the dose will be allowed at V4.

Then the adjusted posology will remain stable during the following 9 weeks of the double blind period.

The effects of pitolisant will be compared to those of the placebo by means of a superiority analysis.

5. STUDY OBJECTIVES

The first objective of this study is to demonstrate the efficacy and safety of pitolisant given at 10, 20, or 40 mg per day versus placebo during 12 weeks for the Double Blind period, to treat the EDS in patients with OSA refusing the nCPAP therapy or treated by nCPAP but still complaining of EDS.

The secondary objectives of the study include assessing the long-term tolerance as well as the maintenance of efficacy of pitolisant given at 10, 20 or 40 mg per day during 39 weeks of Open Label Extension period and further investigating the co-variates or co-medications that affect the pharmacokinetics of pitolisant in the target population to allow future comparison to healthy subjects.

6. POPULATION

The study will be performed in patients experiencing EDS as a result of moderate (15-30 sleep obstructive related breathing events per hour of sleep) to severe (more than 30 sleep obstructive related breathing events per hour of sleep) OSA diagnosed by polysomnography, without important cardiovascular risks, and having refused the nCPAP therapy. Also patients having been submitted to nCPAP therapy for a minimum period of 3 months, trying to adapt the latter at its best, and still complaining of EDS will be included.

Patients refusing to be treated by nCPAP therapy will be asked before the beginning of the Open Label Extension period (at V7) and then at V8, V9, V10, V11 taking into account their cardiovascular profile, whether they want to reconsider their opinion about use of nCPAP, and they will be given the opportunity to withdraw from the study if they accept to use nCPAP therapy.

6.1. Inclusion Criteria

All subjects included in the study will have to meet all of the following criteria for inclusion in the study:

- Male and/or female outpatients aged from at least 18 years
- Patients complaining of EDS refusing to be treated by nCPAP therapy or having been submitted to nCPAP therapy for a minimum period of 3 months, and still complaining of EDS despite the efforts made beforehand to obtain an efficient nCPAP therapy
- Polysomnography performed (for patients submitted to nCPAP therapy – under nCPAP) between V1 and V2 or during the last 12 months with Apnea-Hypopnea Index (AHI): for patients without nCPAP therapy ≥ 15; for patients under nCPAP therapy ≤ 10
- For patients submitted to nCPAP therapy: nCPAP ≥ 4 hours / day (compliance checked on the clock-time counter of the CPAP machine)
- Mini Mental State Examination (MMSE) ≥ 28
- Beck Depression Inventory – 13 items (BDI-13) score < 16 and item G (suicidal ideation) of BDI-13 = 0
- Body Mass Index (BMI) ≤ 40 kg/m²
- Epworth Sleepiness Scale (ESS) ≥ 12
- Female patients with child-bearing potential using a medically accepted method of birth control (i.e. oral contraceptives of normal average dosage) agreeing to continue this method throughout the study, and during the month following treatment discontinuation, being negative to serum pregnancy test performed at the screening visit
- If specified by the investigator, the patient must be willing not to operate a car (if sleepy at wheel) or heavy machinery for the duration of the trial or as long as the investigator deems it clinically indicated. In addition, the patient should be willing to maintain during the study their usual behaviors which could affect their diurnal sleepiness (e.g. circadian rhythm, caffeine consumption, nocturnal sleep duration)
- Patients having signed and dated the informed consent form

6.2. Non-Inclusion Criteria

All subjects included in the study must not meet any of the following non-inclusion criteria:

- Patients suffering from chronic severe insomnia in accordance with the International Classification of Sleep Disorders (ICSD 2005) without OSA
- Patients with co-existing narcolepsy (ICSD 2005), judged on clinical criteria
- Patients with sleep debt not due to OSA (according to the physician' s judgment)
- Patients with non-respiratory sleep fragmentation (restless leg syndrome…)
- Shift work, professional drivers
- Refusal from the patient to stop any current therapy for EDS or predictable risk for the patient to stop the therapy
- Patients suffering from a psychiatric disease
- Acute or chronic disease preventing the improvement assessment, e.g. severe chronic obstructive pulmonary disease (COPD)
- Current or recent (within one year) history of drug, alcohol, narcotic or other substance abuse or dependence
- Any significant serious abnormality of the cardiovascular system, e.g. recent myocardial infarction, angina, hypertension or dysrhythmias (within the previous 6 months), Electrocardiogram Fridericia corrected QT interval higher than 450 ms, history of left ventricular hypertrophy or mitral valve prolapse
- Severe co-morbid medical or biological conditions that may jeopardize study participation at the discretion of the investigator (particularly in the cardiovascular system and the instable diabetes)
- Positive serology tests (HIV, HCV and HBsAg)
- Pregnant or breast-feeding women
- Women with child-bearing potential and no efficient birth-control method
- Patients unable to understand the study protocol
- Patients with suspected or known hypersensitivity to study medication
- Patients with a dominant arm deficiency impeding the achievement of the tests
- Patients using a prohibited medication
- Congenital galactose poisoning, glucose and galactose malabsorption, deficit in lactase

- Patients participating in another study or being in a follow-up period for another study

6.3. Patient completion and early withdrawal of patients from study

A patient will be considered to have completed the study if Visit 7 (end of the Double Blind period) or Visit 13 (Open Label Extension period) has been completed.

A withdrawal patient will be any patient who will not have completed Visit 6 (Double Blind period). In accordance with the Declaration of Helsinki, patients will be free to withdraw from the study at any time if they wish to do so, for any reason specified or unspecified.

Before the end of study visit, the investigator has the responsibility and the right to interrupt a patient's participation. The investigator will have to inform the sponsor or his representative of a patient withdrawal.

The following reasons will be accepted for study discontinuation:

- Patient changing his position towards nCPAP therapy (patient not on nCPAP therapy requiring to be treated by nCPAP; patients on nCPAP therapy refusing to continue it)
- Voluntary withdrawal of patient consent, or loss to follow-up, or inability to remain under medical observation
- Intake of any prohibited treatment
- During the whole study, severe depression indicated by BDI-13 $\geq$ 16 and suicide risk (BDI-13 item G > 0)
- During the whole study, electrocardiogram Fridericia corrected QT interval higher than 450
- Non-compliance or major deviation from the protocol
- In case of occurrence of a Serious Adverse Event (SAE), or any other situation where, in the opinion of the investigator, continuation of the study would not be of benefit to the patient or would put the patient at risk
- Discontinuation of the study by the investigator
- In the situation where the double-blind is broken and the code for the individual patient is opened.

Should any of the subjects be withdrawn from the study, the sponsor's representative, the investigator and the scientific committee if necessary, will discuss together the possibility of

replacement. The reason for withdrawal has to be recorded in the CRF for all withdrawn subjects and in the source document.

7. EXPERIMENTAL DESIGN

This is a prospective, multicenter (several sites in Bulgaria), randomised, Double Blind period III study versus placebo.

It will be carried out in patients diagnosed as having OSA, without important cardiovascular disease, experiencing EDS, having refused the nCPAP or having been submitted to nCPAP therapy for a minimum period of 3 months and still complaining of EDS. With the exception of spending an optional one night (if not done during the twelve months preceding the study) in the sleep laboratory for the full recording of nocturnal polysomnography between V1 and V2, the patient will be ambulatory during the whole study period. To further characterize the pharmacokinetic profile of pitolisant administered in OSA patients up to 40 mg OD a PK analysis will be performed. This will also provide additional data on co-variates or co-medications that may impact the pharmacokinetics of pitolisant.

After the first wash-out period at the end of the Double Blind period**,** patients may optionally, and once a new informed consent form being signed, enter the 40-week Open Label Extension period of the study consisting in an escalating-dose of the same regimen than in the 1st part of the study, followed by a selected dose phase, with the active drug only, until week 53 after the start of the treatment.

The study will include about 200 patients. This number will allow having 180 completed treated patients (pitolisant, patients refusing nCPAP therapy = 60; placebo, patients refusing nCPAP therapy = 30, pitolisant, patients treated by nCPAP = 60; placebo, patients treated by nCPAP = 30) sufficient for the results analysis of efficacy during the Double Blind period (12 weeks):

The timeline of examinations and tests to be performed at each visit are indicated in section 2 (study diagram), concerning the study diagram for the patients withdrawing their participation in the study after the Double Blind period as well as for patients pursuing the Open Label Extension period.

V1 – Screening visit and beginning of initial wash-out period (D -14)

Before proposing the study to patients refusing to be treated by nCPAP, the investigator will

ensure that the patient still refuses this treatment.

An appropriately signed informed consent will be obtained prior to entry into the study for each patient having made a positive decision to participate. The investigator must confirm that the patient meets all inclusion criteria and none of the non-inclusion criteria

Patients included in the study shall not take any treatment indicated for EDS or other psychotropic drugs noted in chapter prohibited treatment during the following two weeks before being submitted to baseline examination (with the exception of chronic medications taken to treat pathology, and authorized by the protocol).

Patients will be reminded to contact the investigator between the visits for any issue, such as adverse events, difficulties with the treatment, need for a new treatment, associated pathology, and modification of the current concomitant treatment (applicable for the whole study duration).

Ph1 – Phone contact (D -7)

During this phone contact, the investigator should:

- Check if patient has discontinued prohibited treatment
- Check concomitant treatments
- Check occurrence of AEs

V2 – Inclusion visit and beginning of the Double Blind period: baseline examinations, randomisation, start of escalating dose phase (D 0)

The patients will be randomized to either pitolisant, or placebo.

The treatment will be initiated by an individual titration period over 2 weeks.

1st week: At each morning, with a glass of water during breakfast

Patients on pitolisant will receive

During breakfast	10 mg

Patients on Placebo will receive

During breakfast	Placebo

2nd week: At each morning, with a glass of water during breakfast

Patients on pitolisant will receive

During breakfast	20 mg

Patients on Placebo will receive

During breakfast	placebo

The total treatment period in double-blind is 12 weeks.

V3 – First dose adjustment visit (D 14)

The posology is increased at 40 mg/d pitolisant (high dose) or placebo every morning, during breakfast. If the tolerance does not allow it (occurrence of an adverse event i.e. troublesome insomnia), the patient will continue the treatment intake at 20 mg/d pitolisant (medium dose) or placebo or the posology will be eventually reduced at 10 mg/d pitolisant (low dose) or placebo until V4.

V4 – Second dose adjustment visit and beginning of stable dose phase (D 21)

The posology is maintained for the next following 4 weeks.

If the tolerance does not allow it:

- Patients taking 40 mg/d pitolisant (high dose) or placebo could reduce to 20 mg/d pitolisant (medium dose) or placebo for the following 4 weeks.
- Patients taking 20 mg/d pitolisant (medium dose) or placebo could reduce to 10 mg/d pitolisant (low dose) or placebo or placebo for the following 4 weeks.

Any dose increase will not be allowed at this visit.

After V4 until the end of Double Blind period any dosage change of pitolisant or placebo will not be allowed.

V5 – Control visit: continuation of stable dose phase (D 49)

Any dosage change of pitolisant or placebo will not be allowed at this visit. Hence, pitolisant or placebo treatment at the same stable dose will be continued for another 5 weeks.

V6 – Evaluation visit at the end of the Double Blind period and start of single-blind wash-out period (D 84)

Patients are submitted to the tests and examinations required for the analysis of the double blind study results. Patients will start one-week single blind placebo wash-out period.

Ph2 – Phone contact (V6 + 3D)

During this phone contact, the investigator should:

- Get patient's answers to amphetamine-like withdrawal symptoms questionnaire (DSM IV) and find out patient's global opinion on the effect of investigational drugs
- Check concomitant treatments
- Check occurrence of AEs

V7 – End of study visit for patients who are not entering Open Label Extension period or Beginning of the Open Label Extension period for patients who are entering Open Label Extension period: start of new escalating dose phase (D 91)

If the patient holds the same position towards nCPAP therapy as before, he/she will be proposed to enter the Open Label Extension period of the study after another escalating dose phase.

Patients not willing to continue the pitolisant treatment administration will end their participation in the study at this visit.

Patients willing to continue the pitolisant treatment administration will be given another information leaflet together with an informed consent form to be signed. After having signed the informed consent form to confirm that they agree to participate in the Open Label Extension study period, patients will be administered 10 mg pitolisant OD during one week. Then, on the following week, they will be administered 20 mg pitolisant OD (in the morning, during breakfast, with a glass of water).

V8-V9 – Dose adjustment visits (D 105 and D 112)

The posology is increased to 40 mg/d pitolisant (high dose), every morning, during breakfast. If the tolerance does not allow it (i.e. troublesome insomnia), the patient will get the treatment at dose of 20 mg/d (medium dose) or 10 mg/d (low dose).

At each of the visits V8-V11 the investigator will propose nCPAP to the patients refusing to be treated by nCPAP therapy before giving them the study treatment. If the patient accepts, he/she will be withdrawn from the study.

V10-V11 – Confirmed dose visits (D 196 and D 280)

The posology should be 40 mg/d (high dose) if the tolerance is acceptable. If the study drug is not well tolerated, the investigator may decide to decrease the dose to 20 mg/d (medium dose)

or 10 mg/d (low dose) during the 12-week period following the visit.

Blood samples used for pharmacokinetic purpose will be collected during Visit 10.

V12 – Evaluation visit at the end of the Open Label Extension period and start of wash-out period (D 364)

Patients are submitted to the examinations and tests required for the evaluation of long-term tolerance and maintenance of efficacy of pitolisant. Patients will be submitted to a final visit after a one-week wash-out period. No study treatment will be taken during this period.

Ph3 – Phone contact (V12 + 3D)

During this phone contact, the investigator should:

- Get patient's answers to amphetamine-like withdrawal symptoms questionnaire (DSM IV) and find out patient's global opinion on the effect of investigational drugs
- Check concomitant treatments
- Check occurrence of AEs

V13 – End of study visit for patients who entered into Open Label Extension period (D 371)

The patients are summoned by the investigator for a final visit. Then, the investigator will decide about the new treatment to prescribe to the patients and the patients will be discharged from the study.

8. STUDY SITES

This trial will be conducted under the responsibility of [redacted]: UMHAT "Alexandrovska" – 1, Sv. Georgi Sofiyski Str., Sofia, Bulgaria – Tel.: [redacted] This multicentre study will be conducted in several investigational centres in Bulgaria; they will be opened, in hospitals and sleep disorder centres which will all have the ability to evaluate the efficacy and tolerance of the drug for the treatment of EDS in OSA patients.

9. INVESTIGATIONAL PRODUCTS

9.1. Chemical structure of compounds

Pitolisant (BF2.649) is 1-{3-[3-(4-chloro-phenyl)-propoxy]-propyl}-piperidinium, hydrochloride.

<u>STRUCTURAL FORMULA</u>

Pitolisant

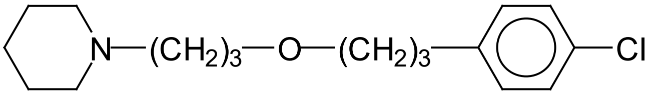

, HCl

<u>MOLECULAR FORMULA</u>

$C_{17}H_{26}ClNO$, HCl %C: 61.44 - %H: 8.19 - %N: 4.21 - %Cl: 21.34 - %O: 4.81

<u>RELATIVE MOLECULAR MASS</u>

Free base	295.85 g
Hydrochloride salt	332.31 g
Base/salt ratio	0.8903

9.2. Composition of investigational products

9.2.1. Composition of 5 mg and 20 mg pitolisant tablet

**Composition of the active tablets**	**Pitolisant 5 mg strength**	**Pitolisant 20 mg strength**
Active substance:		
Pitolisant hydrochloride	5,0 mg	20,0 mg
Excipients:		
Microcrystalline cellulose	20,25 mg	81,0 mg
Crospovidone	2,50 mg	10,0 mg
Talc	2,50 mg	10,0 mg
Magnesium stearate	0,75 mg	3,0 mg
Silica, colloidal anhydrous	0,25 mg	1,0 mg
**Core tablet mass**	**31,25 mg**	**125,0 mg**
Coating:		
OPADRYII HP85F18422 white	2,50 mg	10,0 mg
**Mass of film coated tablet**	**33,75 mg**	**135,0 mg**

9.2.2. Composition of 5 mg and 20 mg placebo tablets

**Composition of the placebo tablets**	**Placebo matching pitolisant 5 mg strength**	**Placebo matching pitolisant 20 mg strength**
Microcrystalline cellulose<br>Crospovidone<br>Talc<br>Magnesium stearate<br>Silica, colloidal anhydrous	23,23 mg<br>2,30 mg<br>2,30 mg<br>0,69 mg<br>0,23 mg	94,536 mg<br>9,360 mg<br>9,360 mg<br>2,808 mg<br>0,936 mg
**Core tablet mass**	28,75 mg	117,0 mg
Coating:<br>OPADRYII HP85F18422 white	2,30 mg	10,0 mg
**Mass of film coated tablet**	31,05 mg	127,0 mg

9.2.3. Administration of study treatment

Both the patient and the investigator will be blind during the first Double Blind period. To make sure that the prescribed dose is appropriately administered to the patient in order to comply with the study conditions, all tablets of pitolisant 5 mg and matching placebo, same as all tablets of pitolisant 20 mg and matching placebo, will have the same mass, colour, shape and size.

During the wash-out period, patients will receive one-week single blind placebo (V6 to V7).

During the Open Label Extension period, the study treatment will be film-coated tablets containing 5 mg or 20 mg of pitolisant.

The patient will be instructed to comply with the posology according to the investigator's judgment during each period.

For the Double Blind period, two 5 mg tablets, one or two 20 mg tablets of pitolisant, or placebo will be administered OD by the oral route according to the following administration scheme:

- In the morning, during breakfast, with a glass of water:

Patients under pitolisant

During breakfast	Low dose<br>10 mg<br>(two 5 mg tablets)	Medium dose<br>20 mg<br>(one 20 mg tablet)	High dose<br>40 mg<br>(two 20 mg tablets)

Patients under placebo will receive tablet(s) of matching placebo.

The therapeutic units will be prescribed to patients according to an individual treatment program and the posology will be determined during the titration phase.

For the Open Label Extension period, patients will be asked to take 2 pitolisant tablets of 5 mg (low dose 10 mg) or 1 pitolisant tablet of 20 mg (medium dose 20 mg) or 2 pitolisant tablets of 20 mg (high dose 40 mg) every morning, during breakfast, with a glass of water according to the schedule of escalating dose and further dose adjustment.

The two first posology (2 tablets of 5 mg and 1 tablet of 20 mg during one week for each) correspond to up-titration and the third one (2 tablets of 20 mg) is the selected dose for the duration of study, except if the tolerance is not acceptable.

9.2.4. Treatment Compliance

At each visit, the compliance to the treatment will be investigated. Patients will be asked whether the investigational treatment was taken as prescribed.

If not, any change in the treatment and the number of forgotten tablets will be recorded in the CRF. In addition, the number of remaining tablets of pitolisant will be counted and compared to the theoretical number which should be left. Any discrepancy will be investigated with the patients.

9.2.5. Packaging and labelling of the treatment boxes

All the study treatments will be supplied, packaged and labelled in compliance with the Good Manufacturing Practices (GMP) and ICH E6 [guidelines for drugs used in clinical trial].

Double Blind period

To provide patients with the required treatment in accordance with the treatment arm and the dosage regimen 3 types of polyethylene high density (PEHD) bottles with active product or placebo will be prepared:

Bottle A (pitolisant 10 mg), containing 20 tablets of pitolisant 5 mg or matching placebo (low dose)

Bottle B (pitolisant 20 mg), containing 10 tablets of pitolisant 20 mg or matching placebo (medium dose)

Bottle C (pitolisant 40 mg), containing 20 tablets of pitolisant 20 mg or matching placebo (high dose)

For single-blind wash-out period bottles containing 20 tablets placebo matching to pitolisant 5 mg, 10 tablets placebo matching to pitolisant 20 mg or 20 tablets placebo matching to pitolisant 20 mg will be used.

The bottles will be packed into boxes. Each patient treatment box will include 35 bottles allowing for all types of study regimen packed into several boxes. The boxes will be packaged into carton-boxes.

The outer packaging of study treatment contains a label with a tear-off part. This tear-off part of the label will be removed and applied on study specific documents. Specific label information will be registered on the corresponding page of the CRF.

Open Label Extension period

For the escalating dose period (at V7) and dose adjustment (at V8), the following PEHD bottles will be prepared:

- Pitolisant 10 mg, containing 20 tablets of pitolisant 5 mg
- Pitolisant 20 mg, containing 10 tablets of pitolisant 20 mg
- Pitolisant 40 mg, containing 20 tablets of pitolisant 20 mg

Each patient treatment box (for the escalating dose period) will include 5 bottles allowing for all types of study regimen packed into 2 boxes. The boxes will be packaged into carton-boxes. For visits from V9 to V11, PEHD bottles will contain 30 tablets of pitolisant 5 mg or 20 mg.

9.2.6. Treatment quantity

Double Blind period

At each visit patients will receive per week 1 bottle containing 20 tablets of 5 mg pitolisant / matching placebo, or 10 tablets of 20 mg pitolisant / matching placebo, or 20 tablets of 20 mg pitolisant / matching placebo; at V6 for the single blind wash out period – containing 20 tablets placebo matching to pitolisant 5 mg, 10 tablets placebo matching to pitolisant 20 mg or 20 tablets placebo matching to pitolisant 20 mg.

Open Label Extension period

For the escalating dose period (at V7) and dose adjustment (at V8) patients will receive per week 1 bottle containing 20 tablets of pitolisant 5 mg, or 10 tablets of pitolisant 20 mg, or 20 tablets of pitolisant 20 mg.

From V9 to V11, at each visit patient will be given in the quantity needed for prescribed treatment bottles of 30 tablets dosed at 5 mg or 20 mg.

9.2.7. Management and storage of the therapeutic units

The necessary therapeutic units will be provided to the hospital pharmacist or the investigator of sleep disorders centres in the minimum period of time before the beginning of the study, and all necessary documents on pitolisant information, according to the Good Clinic Practice (GCP-ICH topic E6 and local regulations) and the local laws.

The hospital Pharmacist or Investigator in sleep disorders centre will be responsible for the management, and the accountability of the therapeutic units (reception, storage, dispensation log, update of the delivery list, return of empty packaging and unused units, and return of therapeutic units). As soon as the treatment units are received, the pharmacist will return the enclosed acknowledgement of receipt sheet distribution, filled and signed.

Treatments under the Pharmacist's or investigator's responsibility must be stored in a locked room and disclosed only to authorized persons having access to this storage room.

In accordance with GCP regulations (ICH topic E6 and local regulations) and the local laws, all study materials (unused treatment units, packaging as well as individual code envelopes) have to be retrieved by the pharmaceutical company responsible of supplying, in accordance with their internal procedure, at the end of the study. The return certificate will indicate for each patient:

- Number of therapeutic units administered,
- Number of therapeutic units unused,
- Number of therapeutic units finally returned to supplier and the dispatch date

In case of a therapeutic unit, and/or individual code envelops loss, the investigator, or the hospital's Pharmacist in charge of the treatment management will have to justify this loss in a written statement, signed and dated, enclosed with the return certificate.

The unused therapeutic units will be retrieved by the pharmaceutical company responsible of

supplying at the end of study (i.e. after final study report signature) after reconciliation of the dispensation log of the therapeutic units. Exceptionally, the unused therapeutic units could be destroyed by the investigator after sponsor being given its agreement

9.2.8. Dispensation Modalities

The study drug will be dispensed only under the restricted conditions defined in the present protocol. Drugs will be administered only by the investigator, or under his direct supervision. The tear-off label of each study drug package will be stuck in the CRF.

9.2.9. Pharmacological Forbidden Concomitant Treatments and surgical interventions

The following pharmacological treatments are forbidden during the study:

- All drugs indicated for somnolence
- All drugs containing sodium oxybate
- All hypnotics drugs defined by ATC class
- Tricyclic antidepressants such as clomipramine, imipramine, desmethylimipramine and protriptyline displaying histamine H_1 receptor antagonist activity that may affect the activity of pitolisant by abrogating the effect of its endogenous histamine release
- H_1 receptor antagonists (in particular those having an effect on the central nervous system)
- Central antihypertensive drugs (Clonidine)
- Any formulation containing codeine
- Psychostimulants (Amphetamine and amphetamine-like CNS stimulants, methylphenidate, modafinil or others)
- Drugs containing dextropropoxyphene (Di-Antalvic)

Surgical interventions including Mandibular Advancement orthosis, uvulopalatopharyngoplasty (UPPP) are also considered as prohibited treatments.

All pharmacological forbidden treatment(s) will be stopped at the selection visit.

A wash out period will be ensured before the inclusion visit.

Exceptionally, or a non-tricyclic antidepressant could be authorized under the same conditions as the chronic treatments prescribed at a stable dose for at least one month before

V1, without any modification throughout the study period and without interfering with daytime sleepiness.

The wash out period will have a minimum of 14 days before inclusion, if the patient is under prohibited treatment.

As far as possible, the concomitant intake of any other treatment will be avoided during the whole study course. All chronic treatments deemed necessary for the patient's condition will remain unchanged the last month prior to enrolment, and during the study period. However the medical state of the patient may require the modification of some treatments for example changing the posology, initiating a new treatment, or stopping a current treatment. All of these treatments have to be reported in the CRF, specifying the posology (name of the drug, type of formulation, unit strength, time and duration of treatment), the prescription date and the reason for prescription.

9.2.10. Randomization of treatments

9.2.10.1. Patient Study number

The study medication will be administered only to patients eligible for the study following the procedures set out in the clinical study protocol.

A patient number (two digits start i.e. 01, 02…) will be assigned at each site sequentially following the number of the study site. The study site number is composed by a sequential number of two digits (01, 02…)

The treatment number is composed by a sequential number of 3 digits for the Double Blind period (DB-XXX) and will remain the same during the dose escalation phase of the Open Label Extension period (OL-XXX). For the stable dose phase, starting at V9, all treatments codes will include a 4 digits number and will be either OL5-YYYY or OL20-YYYY.

A treatment number will be allocated for each patient by ARONE France (4, rue Leroux 94100 Saint-Maur des Fossés – France.

A tracking list of the number allocated with the identification of patients will be filled by the investigator, and kept in his study records.

9.2.10.2. Investigational treatment allocation

Double Blind period

A randomization list will be generated by an entitled company according to Good Clinical Practices. The therapeutic units will be distributed in function of the list managed by ARONE – 4, rue Leroux 94100 Saint-Maur des Fossés – France. Eligible patients will receive a random number corresponding to the treatment assigned in accordance with the randomization schedule. Each treatment unit will have a number printed on the medication label.

The investigator will document the treatment number (or randomization number) on the CRF.

Patients withdrawn from the study will retain their random number. If the decision to replace withdrawn patients is taken by the investigator with the sponsor representative or, if necessary, by the scientific committee, the new patient should be allocated the available treatment.

The necessary quantity of study treatment to be administered during the Double Blind period will be sent to the pharmacist at selection. The study treatment will be delivered to the patient according to their treatment number by the investigator, at inclusion. Then, at each visit, the necessary quantity of study treatment will be given to the patient until the next visit.

The investigator will document the blister number delivered to the patients in the CRF.

9.2.10.3. Unblinding envelopes

The double blind design of the study will result in the ignorance of the assigned treatment to the patients, so during the Double Blind period of the study the assigned treatment will be blinded.

. Two sets of sealed envelopes (one for the pharmacist and another one for the investigator), identified by the study number and treatment number, and containing the unblinded information regarding the type of treatment assigned to the corresponding patient will be sent to the investigators. Another set will be available at the sponsor's level.

In case of emergency the individual code may be broken (see 11.2).

All envelopes will be recovered by the sponsor at the completion of the study.

10. EVALUATION CRITERIA

10.1. Efficacy criteria

10.1.1. Primary endpoint: Epworth sleepiness scale (ESS)

The primary endpoint efficacy criterion is based on the score of Epworth Sleepiness Scale (ESS) by comparing the score differences between baseline (mean between V1 and V2) and at the end of the Double Blind period (mean between V5 and V6) in pitolisant and placebo groups and the sustained improvement of ESS from baseline at week 52 in the Open Label Extension period.

This rating scale is a subjective, validated, sensitive and specific assessment of sleepiness in OSA patients in eight situations described hereafter:

1. Sitting and reading
2. Watching the television
3. Sitting inactive in a public place (cinema, theatre, meetings)
4. Passenger in a car or in the public transportation for at least one hour without stop
5. Lying down to rest in the afternoon in conditions allowing to have rest
6. Sitting and talking to someone
7. Sitting quietly after a lunch
8. In a car when stopped few minutes in a traffic jam

The patient has to rate his or her likelihood of dozing or falling asleep. Each of the eight questions is rated from 0 to 3

0 = never, or no likelihood of falling asleep

1 = rarely falling asleep, or slight likelihood of falling asleep

2 = Often falling asleep, or moderate likelihood of falling asleep

3 = high probability of falling asleep

The responses are summed to yield a score between 0 and 24 with higher scores representing greater sleepiness. The score greater or equal to 11 is considered as abnormal sleepiness.

ESS will be evaluated at each study visit, and reviewed by the investigator.

The questionnaire will be given to the patients at approximately the same time of the visit day and with the same delay after the investigational treatment intake, in order to standardize the

possible impact of treatment on the evaluation.

The patient should be supported in the comprehension of questionnaire until he / she fully understands the eight questions of the score. The patient will be reminded that the ESS measures the subjective sleepiness with regard to the immediate past week.

The value and the sum of each rated question will be uploaded by the investigator in the CRF during the visits.

ESS will be performed at each study visit.

An example of the ESS questionnaire is provided in Appendix 3.

10.1.2. Secondary endpoints

10.1.2.1. Percentage of ESS responders

A responder corresponds to a patient in whom the absolute value of the ESS is < 11, or the difference between the baseline and the end of the Double Blind period scores ≥ 3.

10.1.2.2. Reduction of sleepiness and sleep episodes on the sleep diary

The patient diary is a booklet developed and used to capture, by each patient, his/her subjective evaluation of EDS as a secondary outcome measure. A sleep diary will be reviewed for each patient at each visit except V1 (V2, V3, V4, V5, V6, V7, V8, V9, V10, V11, V12 and V13).

Each patient will be instructed to answer to the sleep diary questions on 3 sequential days of the week preceding the next visit, and to bring back the completed diary.

The patients will answer the following questions:

1. What time did you go to sleep last night?
2. What is the estimated time of your falling asleep last night?
3. Number of awakening episodes during last night.
4. Total duration of these awakening episodes during last night.
5. What time did you wake up this morning?
6. What time did you get up this morning?

7. Number of episodes of sleep and sleepiness during this day.
8. Total duration of sleep and sleepiness during this day.

The analysis of the sleep diary will be based on the responses of the patients.

An example of the sleep diary questionnaire is provided in Appendix 4.

10.1.2.3. Improvement in vigilance according to Oxford Sleep Resistance (OSleR) test

The OSleR test consists in 40 minutes sleep-resistance challenges conducted in a dark room isolated from external noise. The subject, dressed and lying in semi recumbent position, is asked to stay awake without using specific strategies. By hitting a button placed on a box directly connected to a personal computer, the subject is instructed to respond to a visual stimulus (light-emitting-diode flash) which appears for 1 second every 3 seconds.

Any time the red light flashes, the patient is asked to place his/her finger on the yellow button for about 1 second, then to remove his/her finger. He/she is instructed to repeat the cycle every time he/she sees the red light flashing. When the light stops blinking, the test is ended.

All tests are video recorded to check that the subjects are following the instructions. Each patient at V2, V6, V7 and V12 (optionally) undergoes the OSleR in 3 sessions with 2 hours interval between them (at 9:00, 11:00 and 13:00), allowing the assessment of vigilance at different times of the day. The OSleR test algorithm defines the sleep onset when there is no response to 7 consecutive flashes (≥ 21 s)[15].

The standard OSleR test used in the study will yield results of mean sleep latency (MSL) and errors (non-responses to stimulations). For each OSleR test, the consecutive errors are allocated to an error profile (EP) type (i.e. two consecutive errors = EP2, three consecutive errors = EP3).

The EPs are clustered into type:

- EP 1-2 for one and two consecutive errors, indicating lack of attention
- EP 3-6 for three to six consecutive errors, indicating micro-sleep
- EP ≥ 7 for 7 or more consecutive errors, indicating sleep onset

Improvement of vigilance will be evaluated according to increase in sleep latency and reduction in the number of errors.

10.1.2.4. Increase in quality of life

Increase in quality of life will be assessed using European Quality of Life Questionnaire, Leeds Sleep Evaluation Questionnaire and The Pichot Fatigue Scale.

European Quality of Life Questionnaire (EQ-5D)

EQ-5D is a standardized instrument used as measure of health outcome. Applicable to a wide range of health conditions and treatments, it provides a simple descriptive and a single index value for health status.

EQ-5D essentially consists of 2 pages: the EQ-5D descriptive system and the visual analogue scale (VAS).

The EQ-5D descriptive system comprises the following 5 dimensions: mobility, self-care, usual activities, pain/discomfort, and anxiety/depression. Each of the 5 dimensions comprising the EQ-5D descriptive system is divided into 3 levels of perceived problems: Level 1 (no problem); Level 2 (some problems); Level 3 (extreme problems). The patient is asked to indicate his/her health state by ticking (or placing a cross) in the box against the most appropriate statement in each of the 5 dimensions.

A unique health state is defined by combining 1 level from each of the 5 dimensions. Each state is referred to in terms of 5 digit code. For example, state 11111 indicates no problems on any of the 5 dimensions, while state 11223 indicates no problems with mobility and self care, some problems with performing usual activities, moderate pain or discomfort and extreme anxiety or depression.

The EQ-5D VAS records the patient's self-rated health on a vertical, visual analogue 100-point scale where the endpoints are labelled "Best imaginable health state" and "Worst imaginable health state".

EQ-5D will be performed at V2, V6, V7 and V12:

An example of the EQ-5D questionnaire is provided in Appendix 5.

Leeds Sleep Evaluation Questionnaire (LSEQ)

The LSEQ[16] will be used to record key elements of aspects of sleep and early morning behaviour.

The LSEQ contains ten questions pertaining to four consecutive aspects of sleep: getting to

sleep (GTS), quality of sleep (QOS), awakening from sleep (AFS), and behaviour following wakefulness (BFW).

Each patient will be asked 10 questions. A 10-cm line separates the two halves of each question. Each question is answered by placing a vertical mark on the answer line. If no change was experienced, the mark is placed in the middle of the line. If a change was experienced then the position of the mark will indicate the nature and the extent of the change.

The LSEQ will be performed at V2, V6, V7 and V12.

An example of the LSEQ is provided in Appendix 6.

<u>The Pichot Fatigue Scale</u>

The Pichot questionnaire is a practical 24-item self-rating account with three homogeneous sub-scales of 8 items each which measure depressive mood, asthenia-fatigue and anxiety parameters, respectively.

The asthenia-fatigue scale used in this study, consists of 8 questions scored progressively from "0" (not at all) to "4" (extremely) in the following situations:

1) I feel short of stamina
2) Everything I do requires a huge effort
3) I have a feeling of weakness in certain parts of my body
4) My arms or legs are heavy
5) I feel tired for no reason
6) I feel like lying down or resting
7) I have difficulty to concentrate
8) I feel discouraged, my arms and legs are sore and heavy

A score > 22 is in favour of excessive fatigue.

The Pichot Fatigue Scale will be performed at V2, V6, V7, V9, V10, V11, V12 and V13.

An example of the Pichot Fatigue Scale is provided in Appendix 7.

10.1.2.5. Improvement in cognitive function

Improvement in cognitive function will be assessed using Trail Making Test.

Both parts (A and B) of the TMT consist of 25 circles distributed over a sheet of paper.

In Part A, the circles are numbered 1 to 25, and the patient should draw lines to connect the numbers in ascending order.

In Part B, the circles include both: numbers (1 to 13) and letters (A to L). As in Part A, the patient draws lines to connect the circles in an ascending order, but with the added task of alternating numbers and letters (i.e., 1-A-2-B-3-C, etc.).

The patient should be instructed to connect the circles as quickly as possible, without lifting the pen or pencil from the paper. Time used by the patient to connect the "trail" is registered. If the patient makes an error, it is pointed out immediately and the patient is allowed to correct it. Errors affect the patient's score only in that the correction of errors is included in the completion time for the task. It is unnecessary to continue the test if the patient has not completed both parts after five minutes have elapsed.

Step 1: Give the patient a copy of the Trail Making Test Part A worksheet, and a pen or pencil.

Step 2: Explain the test to the patient using the sample sheet (Trail Making Part A – *SAMPLE*).

Step 3: Time the patient as he or she follows the “trail” made by the numbers on the test.

Step 4: Record the time.

Step 5: Repeat the procedure for Trail Making Test Part B.

Scoring:

Results for both TMT A and B are reported as the number of seconds required to complete the task; therefore, higher scores reveal greater impairment.

The average time to complete the test is for Part A: 29 seconds and for Part B: 75 seconds. Scores superior to 78 seconds for Part A and 273 seconds for Part B reveal deficiency.

The TMT parts A and B will be performed at V2, V6, V7 and V12.

An example of the Trail Making Test (TMT) parts A and B is provided in Appendix 8.

10.1.2.6. Improvement in Clinical Global Impression (CGI)

The CGI is a 3-item observer-rated scale which measures illness severity (CGI-S), global improvement or change (CGI-C), and therapeutic response. The CGI is rated on a 7 point scale, with the CGI-S scores ranging from 1 (normal) to 7 (the greatest severity) and CGI-C scores ranging from 1 (very much improved) to 7 (very much worse). Treatment response

ratings should take into account both therapeutic efficacy and treatment-related adverse events, and range from 0 (marked improvement and no side effects) to 4 (unchanged or worse, and side-effects outweigh the therapeutic effects).

The CGI-S test measure will be performed at V1 and V2, CGI-C test measure – at V6, V7, V10, V11, V12 and V13.

An example of the CGI scales is provided in Appendix 9.

10.1.2.7. Patient's global opinion on the effect of investigational drugs

The patient should evaluate the global effect of the treatment, by comparing the period prior to the visit with the patient's pre-study condition. The following six-level scale will be used:

- Marked effect (complete or nearly complete remission of EDS)
- Moderate effect (partial remission of EDS)
- Minimal effect (slight decrease in EDS that does not substantially change the status of the patient)
- No change
- Minimally worse (slight increase in EDS)
- Much worse (substantial increase in EDS)

The Patient's global opinion on the effect of investigational drugs measure will be performed at V6, Phone contact 2, V7, V10, V11, V12, Phone contact 3 and V13,

An example of the Patient's global opinion on the effect of investigational drugs is provided in Appendix 10.

10.1.2.8. Aggregate Z-score of secondary endpoints.

ESS and OSLER constitute two essential symptoms of EDS, whereby it is possible to assess the overall efficacy of the tested drug, through a multivariate approach, by using a unique test on a composite Z-score.
This Z-score will be calculated for ESS (ESSBL and ESSF) and (OSLERBL and OSLERF), for all these variables both at baseline and final visit.

10.2. Safety criteria

A Data Safety Monitoring Board (DSMB) will be created. It will be made up of a group of independent expert's external to the study assessing the progress, safety data and, if needed critical efficacy endpoints of the clinical study.

In order to do so, a DSMB may review unblinded study information (on a patient level or treatment group level) during the conduct of the study. Based on its review, the DSMB provides the sponsor with recommendations regarding study modification, continuation or termination. DSMB Charter is provided in Appendix 11.

10.2.1. Period of observation

For the purpose of this study, the period of observation extends from the time the patient gives informed consent (Visit 1) until one month after the last visit (Visit 7 for patient not entering in the Open Label Extension period, and V13 for patient participating in the Open Label Extension period,). Any adverse events observed by the investigator or reported by the patient during the period of observation must be documented in the CRF.

If the investigator detects a serious adverse event in a patient after the end of the observation period, and considers the event possibly related to the study treatment, he or she should contact the sponsor to determine how the adverse event should be documented and reported.

10.2.2. Adverse Event (AE)

The term Adverse Event: Any untoward medical occurrence in a patient or clinical investigation subject administered a pharmaceutical product and which does not necessarily have a causal relationship with this treatment. An AE can therefore be any unfavourable and unintended sign (including an abnormal laboratory finding), symptom, or disease temporally associated with the use of a medicinal (investigational) product, whether or not related to the medicinal (investigational) product.

No causal relationship with the study treatment or with the clinical study itself is implied by the use of the term "Adverse Event".

Adverse Events fall into the categories "non-serious" and "serious" (see Section "Serious Adverse Event").

AEs include also:

- Clinically abnormal laboratory results
- Overdosage
- Abnormal ECG results

For any reports in the above categories an AE form has to be filled in addition to the exam results.

10.2.3. Treatment-Emergent and Baseline-Emergent Adverse Events

A Treatment-Emergent Adverse Event is defined as any event which is reported as occurring after the first study drug intake or any event already present before first study drug intake and which worsens either in intensity, or frequency following the exposure to the study treatments. A Baseline-Emergent Adverse Event is defined as any event which occurs or worsens during the staged screening process (after informed consent is given) including the randomization visit.

10.2.4. Adverse Drug Reaction (ADR)

All noxious and unintended responses to a medicinal product related to any dose should be considered as an Adverse Drug Reaction. The responses to a medicinal product means that a causal relationship between a medicinal product and an adverse event is at least a reasonable possibility, i.e. the relationship cannot be ruled out.

10.2.5. Unexpected Adverse Drug Reaction

An adverse reaction, in nature or severity which is not consistent with the applicable product information (e.g., Investigator's Brochure for an unapproved investigational product or package insert/summary of product characteristics for an approved product).

10.2.6. Serious Adverse Event (SAE) or Serious Adverse Drug Reaction (Serious ADR)

Any untoward medical occurrence that at any dose:

- Results in death
- Is life-threatening[1]
- Requires inpatient hospitalization or prolongation of existing hospitalization
- Results in persistent or significant disability/incapacity[2]

- Is a congenital anomaly/birth defect, or
- Other: important medical event according to the investigator[3]

1 "Life-threatening" means that the patient was at immediate risk of death at the time of the serious adverse event; it does not refer to a serious adverse event that hypothetically might have caused death if it were more severe.

2 "Persistent or significant disability or incapacity" means that there is a substantial disruption of a person's ability to carry out normal life functions.

3 Medical and scientific judgment should be exercised in deciding whether other adverse events may be considered serious because they jeopardize the patient, or may require intervention to prevent one of the other outcomes listed in the definition above.

The List of Critical Terms (1998 adaptation of WHO Adverse Reaction Terminology Critical Terms List) should be used as guidance for adverse events that may be considered serious because they are medically important.

Cases involving cancer as an Adverse Event should be reported as "serious" using the criterion "medically important" if no other serious criterion is met.

Cases of overdose with an adverse event that meets one of the criteria given above should of course be reported as "serious".

10.2.7. Suspected Unexpected Serious Adverse Reaction (SUSAR)

Suspected Unexpected Serious Adverse Reaction: any Adverse Reaction that is classed as serious and is suspected to be caused by the IMP and is not consistent with the information about the IMP in either the Investigator's Brochure or SmPC.

10.2.8. Clarification of the difference in meaning between "severe" and "serious"

The term "severe" is often used to describe the intensity (severity) of a specific event (such as in mild, moderate, or severe myocardial infarction); the event itself, however, may be of relatively minor medical significance (such as severe headache). This is not the same as "serious," which is based on the outcome or action criteria usually associated with events that induce a threat to life or functioning.

Seriousness (not severity) serves as a guide for defining regulatory reporting obligations.

10.2.9. Analysis of adverse events

10.2.9.1. Assessment of intensity

The assessment of intensity (severity) is independent on the assessment of the seriousness of the AE and is based on the investigator's clinical judgment.

Mild: Awareness of signs and symptoms but no disruption of usual activity. Symptoms do not require therapy or a medical evaluation; signs and symptoms are transient.

Moderate: Event sufficient to affect usual activity (disturbing). Are usually improved by simple therapeutic measures.

Severe: Inability to work or perform usual activities (unacceptable). Generally require systemic drug therapy or other treatment.

10.2.9.2. Assessment of Causality

The causal relationship of the adverse event to the study treatment will be assessed and notified according to the following definitions:

Related / likely: Clearly related to the investigational agent / procedure, i.e. an event that follows a reasonable temporal sequence from administration of the study intervention, follows a known or expected response pattern to the suspected intervention, that can be confirmed by improvement on stopping and reappearance of the event after rechallenge and that could not be reasonably explained by the known characteristics of the subject's clinical state.

Possibly related / Possible: Follows a reasonable temporal sequence from administration of the study intervention, follows a known or expected response pattern to the suspected intervention, but that could readily have been produced by a number of other factors.

Not related / Unlikely: Clearly and incontrovertibly due only to extraneous causes, and does not meet criteria listed under possible (possibly related) or likely (related).

10.2.10. Reporting Adverse Event to the Sponsor (Appendix 12)

All Adverse Events that occur after the patient has signed the informed consent must be documented on the pages provided in the CRF in accordance with the "Instructions for the completion of Adverse Events". These instructions are provided in the investigator study file,

and in the CRF itself.

The following approach will be taken for documentation:

All adverse events (whether serious or non-serious) must be documented on the "Adverse Event" page of the CRF including clinically significant lab or examination results.

If the adverse event is serious, the investigator must complete a "SAE report form" at the time the Adverse Event is considered as Serious.

In the situation when a "significant overdose" occurs, the investigator should complete the "Adverse Event" page of the CRF. If this overdose presents one of the criteria outlined in section 10.2.6 (definition of SAE), the investigator should complete the "SAE report form". Every attempt should be made to describe the Adverse Event in terms of a diagnosis. If appropriate, component symptoms should also be listed below the diagnosis.

If only non-specific signs or symptoms are present, then these should be recorded as a diagnosis.

All patients who experience an Adverse Event, whether considered associated with the use of the study medication or not, must be monitored to determine the outcome. The clinical course of the Adverse Event will be followed up according to accepted standards of medical practice, even after the end of the observation period, until a satisfactory explanation is found, or the investigator considers it medically justifiable to terminate follow-up.

Should the adverse event result in death, a full pathologist's report should be supplied, if possible.

All questions on the completion and supply of Adverse Event report forms, and any further forms issued to the investigator at a later date to clarify unresolved issues should be addressed to the sponsor.

10.2.11. Reporting Serious Adverse Event to the sponsor

Serious Adverse Events and Adverse Events that fulfil a reason for expedited reporting to Pharmacovigilance (significant overdose, pregnancy) must be documented on a SAE report form in accordance with the "Instructions for Completing the SAE report form" (see Appendix 12).

The investigator must inform the site monitor and sponsor in all such cases within 24 hours after the investigator becomes aware of the SAE (or at the latest on the following working day). The SAE form must be completed and faxed [Fax number +33 147036630] or emailed

[email y.joulin@bioprojet.com] to Bioprojet Pharmacovigilance department at latest within 3 calendar days after the investigator becomes aware of the SAE. The initial report must be as complete as possible, including details of the current illness and the Serious Adverse Event, and most importantly an assessment of the causal relationship between the SAE and the study medication. Information not available at the time of the initial report (e.g., end date of the Serious Adverse Event or laboratory values received after the report) must be documented on a follow-up “SAE report form”.

In addition, the following CRF data must be provided as soon as possible to Bioprojet Pharmacovigilance Department: “Demography”, “Relevant diseases/illnesses in the patient’s medical history”, “Previous and concomitant medication”.

The site monitor is responsible to ensure that the entire information is forwarded to Bioprojet Pharmacovigilance department. Bioprojet will ensure that all legal reporting requirements are met.

The SAE report form and the instructions on completion are provided in the investigator’s study file. The “Instructions for Completing the SAE report form” give more detailed guidance on the reporting of serious adverse events, significant overdose cases, and Adverse Events initially reported as non serious that become serious. In the latter situation, where a non-serious event becomes serious, details must be forwarded immediately to the sponsor on a SAE report form”.

10.2.12. Reasons for expedited reporting

No special Adverse Events are subject to reporting as alert terms in this study.

However, cases in which a “significant overdose” of the investigational product is to be reported to the sponsor on a SAE report form is to be dispatched to the sponsor in an expedited manner.

In addition, any pregnancy diagnosed in a subject during treatment with the investigational product must be reported to the sponsor immediately and must be followed up until child birth. Patient should immediately stop the study treatment.

During and after a patient's participation in the trial, the investigator should ensure that adequate medical care is provided to the patient for any adverse events, including clinically significant laboratory values, related to the trial. The investigator should inform the patient when medical care is needed for intercurrent illness(es) of which the investigator becomes aware.

10.2.13. Adverse Events Outcome

All adverse Event outcomes should be documented at the last visit, the latest according to the following criteria:

- Recovered
- Recovered with sequelae
- Worsened
- Not yet recovered
- Death

10.2.14. Safety Endpoints

10.2.14.1. Adverse events

Adverse Events, emergent or not, reported during the study course (frequency, intensity, relationship to study drug, incidence and occurrence) will be analyzed as the main safety parameters.

The events will be categorized by organ class (current MedDRA) and type, seriousness, intensity and relationship to the treatment.

10.2.14.2. Physical examination

A full physical examination will be performed at each visit. Any significant abnormality and change from baseline will be recorded in the CRF and in the source document, and will be analyzed as safety parameters. Any untoward change will be reported in the AE pages of the CRF.

10.2.14.3. Vital signs

Vital signs including systolic and diastolic blood pressures and heart rate will be measured at each visit. Measurements will be recorded in the CRF and in the source document.

Any change in vital signs parameters (heart rate, blood pressure) from baseline will be analyzed as safety parameters. Particular attention will be focused on cardiovascular examination, essentially by verifying the arterial blood pressure at screening and at each study visit.

10.2.14.4. Electrocardiogram (ECG)

ECG will be performed at each study visit using the internationally recognised ECG recording. ECG parameters including heart rate and QTc (electrocardiogram Fridericia corrected QT interval (QT/$^3\sqrt{}$ [60/HR]) interval will be analyzed and reported in the CRF and in the source document. Patients presenting a QTc strictly higher than 450 ms will not be eligible in this study. QTc and other ECG parameters intra individual changes will be reported and analyzed in all treatment groups at V6.

10.2.14.5. Beck Depression Inventory (the reduced version with 13 items)

The Beck Depression Inventory created by Dr. Aaron T. Beck, is a multiple-choice self-report questionnaire, one of the most widely used instruments for measuring the severity of depression. The current reduced version with 13 items is composed of items relating to symptoms of depression such as hopelessness and irritability, cognitions such as guilt or feelings of being punished, as well as physical symptoms such as fatigue, weight loss, and lack of interest in sex (Beck AT (2006) Depression: Causes and Treatment. Philadelphia: university of Pennsylvania Press).

The BDI-13 will be performed at visits V1, V2, V6, V7, V9, V10, V11, V12 and V13.

An example of BDI-13 is provided in Appendix 13.

10.2.14.6. Patient's overall evaluation of the tolerance

The patient should evaluate tolerance of the treatment. The following three-level scale will be used:

- Good
- Moderate
- Poor

Patient's overall evaluation of the tolerance will be performed at visits V3, V4, V5, V6, V7, V8, V9, V10, V11, V12 and V13.

An example of Patient's overall evaluation of the tolerance is provided in Appendix 14.

10.2.14.7. Amphetamine-like withdrawal symptoms questionnaire (DSM IV)

The Amphetamine-like withdrawal syndrome is defined as dysphoria and two or more of the following: fatigue, vivid and unpleasant dreams, insomnia or hypersomnia, increased appetite, and psychomotor retardation or agitation (DSM IV).

This questionnaire will be performed at phone contact 2, V7, phone contact 3 and V13.

An example of Amphetamine-like withdrawal symptoms questionnaire is provided in Appendix 15.

10.2.14.8. Laboratory tests

A full laboratory test should be performed at V1 (screening visit) and at V6 for each patient completing the Double Blind period. Patients entering into the Open Label Extension period will have additionally lab tests at V12. Biological work up will also be performed as soon as possible for any subject who discontinues the study prior to the completion of the Double Blind period or the Open Label Extension period.

Blood samples will be collected and all laboratory tests will be performed according to the Good Laboratory Practices (OECD GLP Principles) by site-dependant Laboratory.

The results of the laboratory tests will be interpreted by the investigator. Laboratory abnormalities will be defined as laboratory test results that are outside the reference range as defined by the normal range from the testing laboratory.

Clinically significant abnormal value at V1 (results available at V2) will be determined by the investigator, and will lead to the exclusion of patients from study participation.

The results of all values of laboratory tests will be reported in the CRF. The original document containing all the tested values will be kept in the patient study file.

Blood laboratory test parameters will be reported in the CRF and intra individual changes in these parameters will be analyzed in both treatment groups at visit 6 for the Double Blind period, and at visit 12 for all patients entered into the Open Label Extension period. Any abnormalities considered clinically significant will be recorded in the adverse event page of the CRF.

The following blood parameters will be evaluated:

Hematology (hemoglobin, hematocrit, white blood cell [WBC] count with differential, red blood cell [RBC] count, platelet count, mean corpuscular volume, and coagulation time [INR]) will be performed at V1, V6, V12 and at early withdrawal visit;

Biochemistry (blood urea nitrogen [BUN], uric acid, creatinine, creatine kinase, SGPT [alanine aminotransferase (ALAT)], SGOT, [aspartate aminotransferase (AST)], γGT [Gamma-Glutamyltranspeptidase], alkaline phosphatase, total protein, total bilirubin, glucose, albumin, electrolytes [sodium, potassium, calcium, chloride, Bicarbonates/CO2], total cholesterol and triglycerides) will be performed at V1, V6, V12 and at early withdrawal visit.

β-HCG serum pregnancy test will be performed at V1, V6, and V12 and at early withdrawal visit in all patients of child-bearing potential. The absence of pregnancy should be confirmed before study drug delivery (at V2 for the Double Blind period and at V7 for the Open Label Extension period).

Serological test (HIV, HBsAg, HCV) will be performed only at the screening visit V1 (patients with positive to serology test will not be included to study).

Urinalysis will be performed using stick test at V1. The results will be recorded in the CRF. Should the stick test be positive, a mid stream sample of urine will be sent for microscopy and bacteriological culture.

Pharmacokinetic sampling will be done at V10. Samples will be taken at pre-dose, 1,5 h, 3 h and 8 h post-dose (see §10.2.14.8 below).

Any laboratory test abnormalities considered clinically significant putting the patient at risk, as judged by the investigator, and if necessary by the scientific committee will lead to immediate discontinuation of the study drug. The Sponsor must be informed immediately.

In case the investigator considers abnormalities will not put the patient at risk, continuation of the drug will be allowed after discussion with the Sponsor and the scientific committee.

The patient will be followed up with appropriate medical care until he/she returns to normal or baseline values, or clinical diagnostics of undercurrent illness is confirmed.

The results of all known laboratory tests required by the protocol will be held and recorded in the patients CRFs. All clinically important abnormal laboratory tests occurring during the study will be repeated at intervals judged appropriate by the investigator until they return to baseline or to a level deemed acceptable by the investigator.

10.2.14.9. Pharmacokinetic assessments

Pharmacokinetic sampling will be done at V10. Blood samples for PK analysis will be performed at pre-dose, 1,5 h, 3 h and 8 h post-dose.

For pitolisant and metabolites determinations, a 4,0 ml blood sample will be collected into one polyethylene terephthalate (PET) tube without any anticoagulant. After clotting (45 minutes at room temperature), serum will be separated in a refrigerated centrifuge (*ca.* +4°C) at *ca.* 1500 *g* for 10 min for and the complete volume of resulting serum (at least 1,8 ml from 4,0 ml blood sample) will be dispensed equally to 3 polypropylene cryotubes (no screwed-tap) with a minimum of 0,55 ml per tube and stored upright at –80 ± 10 °C. Upon collection, the samples will be processed as soon as possible. If not processed immediately, the samples should be kept on wet ice for a maximum time of 1 h.

Each tube will be precisely identified, with:

- Protocol No.
- Subject No.
- Time of study drug administration
- Sampling time (theoretical) (Visit, Date, Time).
- Sampling time (actual time).
- Storage conditions.

Thereafter, the samples will be stored at –80 ± 10°C until the shipment to the bioanalytical centre.

Two (2) series of tubes (serum samples) will be transferred in two different shipments to:

[redacted]
Bioprojet Biotech
4 rue du Chesnay Beauregard,
BP96205, 35762-Saint Grégoire, France
Phone:[redacted]
Email:[redacted]

And the third series of tubes will be stored at the clinical site and can be sent to the bioanalytical center if necessary.

Before any shipment, the site monitor will confirm by appropriate mean of communication (email or fax) to [redacted] the exact dates and hours of each consignment of samples. Only after receipt of [redacted] agreement, the site monitor will organize

the shipment. The shipment of the samples will have to be organized at least 2 days before any official holiday or week-end.

The study monitor will ensure samples will be sent in boxes containing enough dry ice for a period of at least 72 h. A control of temperature will be done upon receipt of the samples by checking the amount of remaining dry ice and if the samples are in a frozen state.

The samples will be registered according to the SOP used in Bioprojet Biotech. The biological samples will be stored at –80 ± 10°C until analysis. The biological samples will be handled using gloves to prevent any risk of contamination.

The analysis of the study samples will be described in a separate analytical protocol and reports (Bioprojet Biotech study code B410).

Serum levels of BF2.649/BP2.951, BP1.8054 and BP1.9733 will be evaluated using validated LC/MS/MS methods (Bioprojet-Biotech study code B258, B349 and B387, respectively). The limits of quantification will be 0,1, 1 and 1 ng/ml, respectively.

10.2.14.10. Overdosing

Rules of conduct in case of pitolisant overdosage:

- From 120 mg onwards, in one single intake in adults, it is recommended to keep the patients under medical supervision.
- From 200 mg onwards, it is preferable to have them hospitalized.

In addition to this treatment, a monitoring of the vital functions shall be performed and a H_2 antihistamine product, such as Tagamet®, shall be administered in case of gasteralgias.

10.2.14.11. Observance of nightly nCPAP use

The observance of nCPAP will be assessed by the clock-time counter of the CPAP machine at each visit (reading of the last day) prior to each visit (for patients submitted to nCPAP therapy).

11. EMERGENCY PROCEDURES

11.1. Sponsor contact

In emergency situations, the investigator may contact the Sponsor representative by telephone at the number listed on the title page of the protocol.

Pharmacovigilance Responsable contact: [redacted], Pharm.D., Ph.D.

Bioprojet – 9, Rue Rameau, 75002 Paris, France

[redacted]

The Medical Project Manager: [redacted]

Bioprojet – 9, Rue Rameau, 75002 Paris, France

[redacted]

11.2. Emergency identification of Study Products

Unblinding envelopes (see 9.2.9.3) must only be opened in case of emergency (i.e. in case an imperative need to know the assigned treatment by the investigator to treat the patient adequately in case of serious adverse event or in case of overdosage or study drug misused, or in the event of death, or when requested by a regulatory agency in the case of a serious adverse event).

The code breaking will be documented on the envelope and in the CRF, with the date, the time and the reasons of breaking and will be signed by the investigator. Before opening the envelope, the investigator will contact the study monitor or medical project manager to explain the situation and validate the need to break the code. Only under extreme emergency situation, the investigator will break the code without authorization. In this case, information on code breaking will be provided to the clinical research associate (CRA) or medical project manager as soon as possible. Information related to the actual treatment given during the double blind period should not be provided to any members of the study team. When a code is broken for a patient, the patient must be withdrawn from the study. In this case an early withdrawal visit and the end of study form must be completed and recorded in the CRF.

12. CONDUCT OF THE TRIAL

This is a prospective, multicenter (several sites in Bulgaria), randomised, double blind study versus placebo.

The study population will be recruited from patients who have a current confirmed diagnosis of OSA, having refused the nCPAP therapy or treated by nCPAP, and who still complain of EDS. Patients should not have important cardiovascular risk factors.

After the Double Blind period**,** patients may optionally, and once a new informed consent form being signed, enter the 40-week Open Label Extension period of the study consisting in an escalating-dose of the same regimen than in the 1st part of the study, followed by a selected dose phase, with the active drug only, until week 53 after the start of the treatment.

The study will include about 200 patients. This number will allow having sufficient completed treated patients – 180 (pitolisant = 120; placebo = 60) for the per protocol analysis of efficacy during the Double Blind period (12 weeks).

The maximum duration of the study will not exceed 55 weeks (2 weeks for baseline without treatment, 12 weeks for the first Double Blind period, 1 week wash-out period with placebo after V6, 39 weeks for the Open Label Extension period and a 1 week-wash out period without placebo after V12). The patient will be ambulatory during the whole study period except staying 1 optional night in the sleep laboratory for the full recording of nocturnal polysomnography (if it was not performed during the last twelve months).

The timeline of examinations and tests to be performed at each visit are indicated in section 2 (study diagram), concerning the patients withdrawing their participation in the study after the Double Blind period as well as for patients pursuing the Open Label Extension period.

The date of each visit could be D ± 3 Days.

12.1. Part I - Double Blind period

12.1.1. V1 – Screening visit and beginning of initial wash-out period (D -14)

Before proposing the study to patients refusing to be treated by nCPAP, the investigator will ensure that the patient still refuses this treatment.

If the patient may be enrolled in the study, the investigator has to give him/her the necessary information which will be made orally and by giving the specific patients' informed consent

form. Objective, treatments, expected benefit, potential risks, constraints and procedures of the study will be carefully explained to the patient, as well as the alternative available treatments.

An adequate delay for the patient to decide if he/she is willing to participate in this study will be respected. An appropriately signed informed consent will be obtained prior to entry into the study for each patient having made a positive decision to participate.

It is only after the patient accepts to be enrolled into the study that any screening tests and examinations can be performed.

During this screening visit, the investigator must confirm that the patient meets all inclusion criteria and none of the non-inclusion criteria, and that his/her state of health allows him/her to discontinue prohibited treatments.

Baseline history and examination will be obtained in order to collect relevant data prior to the beginning of the study.

The investigator will perform the following screening assessments:

- Medical questionnaire including the patient demographic data, OSA and EDS history and documentation of the previously performed tests/questionnaires about illness (such as polysomnography, AHI, Epworth score, OSleR test, BMI, state of sleep-related desaturation), actual symptoms prior to admission and their duration, possible complications, details of concomitant treatments including any current and previous treatments of EDS and any other types of treatment.
- A complete physical examination, in particular vital signs including blood pressure, heart rate, respiratory rate.
- Epworth Sleepiness Scale (ESS). Patients with a score of ESS<12 will not be included in trial.
- The Mini Mental State Examination (MMSE); an example of MMSE questionnaire is provided in Appendix 16. All patients with a score of MMSE < 28 will not be included in the trial.
- Clinical Global Impression – illness severity (CGI-S).
- BDI-13 will be performed to assess the state of depression, and all patients with a score of BDI-13 ≥ 16 and item G (suicidal ideation) positive will be withdrawn from the trial.
- ECG.

- Clinical laboratory tests: biological (hematology biochemistry), serological (β-HCG pregnancy test for woman with child-bearing potential, HIV, HCV, HBsAg) tests and urinalysis (stick test with microscopy and bacteriological culture, if positive). Laboratory test results will be reviewed prior to inclusion in the study.

All these data have to be reported in the CRF and compared to the inclusion and non-inclusion criteria. Only patients who fulfil all inclusion and exclusion criteria and sign the informed consent could be enrolled in this study.

If enrolled, the patient will have to stop his/her current medication against EDS for a two week wash-out period.

Sleep diary will be delivered. The patient will answer to the sleep diary questions during 3 sequential days of the week preceding next visit. An appointment will be fixed for the inclusion visit (V2) 2 weeks later.

An appointment for a one-night polysomnography (see Appendix 2) will be planned between V1 and V2, if it was not performed during the last twelve months.

The patient will be reminded to contact the investigator between the visits for any issue, such as adverse events, difficulties with the treatment, need for a new treatment, associated pathology, and modification of the current concomitant treatment (applicable for the whole study duration).

12.1.2. Ph1 – Phone contact (D -7)

During this phone contact, the investigator should check if patient has discontinued previously used treatment for EDS and the intake of all prohibited treatments. Also other concomitant treatments and occurrence of AEs will be checked.

12.1.3. V2 – Inclusion visit and beginning of the Double Blind period: baseline examinations, randomisation, start of escalating dose phase (D 0)

The following assessments will be performed during this visit:

- Medical questionnaire.
- Physical examination, in particular vital signs of the patient.
- ESS.

- Three sessions of Oxford Sleep Resistance test (OSleR test) at approximately 09:00, 11:00 and 13:00.
- Trail Making Test (TMT) parts A and B.
- CGI-S.
- BDI-13.
- Leeds sleep evaluation questionnaire (LSEQ).
- European Quality of Life Questionnaire (EQ-5D).
- Pichot Fatigue Scale.
- ECG.
- Review of V1 laboratory tests results.
- Analysis of the polysomnography report performed in the 12 previous months before V1 or performed in between V1 and V2.
- Review of the sleep diary.
- Investigation of any adverse event occurrence.
- Verification that the patient has discontinued the intake of all prohibited treatments.
- Confirmation of the inclusion of the patient in this study after verification of all inclusion and non-inclusion criteria.

If the patient is included in this study, he/she will be randomized to either pitolisant, or placebo and receive the experimental treatment which is initiated by an individual titration period over 2 consecutive weeks. Depending on the randomization, the treatment assigned to each patient during the first week will be 10 mg pitolisant or placebo. During the second week, it will be 20 mg pitolisant, or placebo.

Two first medication bottles identified "1st week" and "2nd week" will be given to the patient for the 2 weeks. Patient will be instructed to take, starting from the next day after the visit, 2 tablets from corresponding bottle on the 1st week and 1 tablet form the corresponding bottle on the 2nd week every morning, during breakfast. It is important to ensure that the patient does not forget to take the treatment on the day of the next visit.

Sleep diary will be delivered. The patient will answer to the sleep diary questions three sequential days of the week preceding next visit. The next visit will be fixed 2 weeks later, and the patient should bring back the completed diary and the used experimental treatment bottles.

The total treatment period in double-blind is 12 weeks.

12.1.4. V3 – First dose adjustment visit (D 14)

The visit will imperatively take place in the morning in order to ensure the identical conditions for evaluation. It is important to ensure that the patient does not forget to take the treatment on the day of the visit.

The following assessments will be performed during this visit:

- Medical questionnaire including control of the treatment compliance by counting the remaining tablets and verification of the forgotten treatment intake.
- Physical examination, in particular vital signs of the patient.
- ESS.
- Patient's overall evaluation of the tolerance.
- ECG.
- Review of the sleep diary.
- Investigation of any adverse event occurrence.

Once the investigator has ascertained that the tolerance of the study product is acceptable by performing clinical examination, questionnaire to check adverse event and ECG, the posology is increased to 40 mg/d pitolisant (high dose) or placebo, every morning, during breakfast.

If the tolerance does not allow it (occurrence of an adverse event i.e. troublesome insomnia), the patient will continue the treatment intake at 20 mg/d pitolisant (medium dose) or placebo or the posology will be eventually reduced at 10 mg/d pitolisant (low dose) or placebo until V4.

One corresponding bottle will be given to the patient, and he/she will be instructed by the investigator to take, depending on the prescribed dose, 1 or 2 tablets every morning, during breakfast. It is important to ensure that the patient does not forget to take the treatment on the day of the next visit.

Sleep diary will be delivered. The patient will answer to the sleep diary questions on three sequential days before the next visit, which will be fixed in 1 week. The patient should bring back the completed diary and the used experimental treatment bottle.

12.1.5. V4 – Second dose adjustment visit and beginning of stable dose phase (D 21)

After the 1-week treatment period at the selected dose, the investigator will check the efficacy and safety at this visit.

The visit will take place in the morning to ensure the identical condition for evaluation. It is important to ensure that the patient does not forget to take the treatment on the day of the visit.

The following assessments will be performed during this visit:

- Medical questionnaire including control of the treatment compliance by counting the remaining tablets and verification of the forgotten treatment intake.
- Physical examination, in particular vital signs of the patient.
- ESS.
- Patient's overall evaluation of the tolerance.
- ECG.
- Review of the sleep diary.
- Investigation of any adverse event occurrence.

The dose of study treatment will be individually adjusted according to the assessment of investigators on the basis of tolerance of the study treatment in accordance with the following scheme.

The posology is maintained for the next following 4 weeks.
But, if the tolerance does not allow it:

- patients taking 40 mg/d pitolisant (high dose) or placebo could reduce to 20 mg/d pitolisant (medium dose) or placebo for the following 4 weeks.
- patients taking 20 mg/d pitolisant (medium dose) or placebo could reduce to 10 mg/d pitolisant (low dose) or placebo or placebo for the following 4 weeks.

Any dose increase will not be allowed at this visit.

After V4 until the end of Double Blind period any dosage change of pitolisant or placebo will not be allowed. Hence, pitolisant, or placebo treatment under stable doses will be continued for 9 weeks.

Four corresponding bottles (one per week) will be given to the patient, and he/she will be instructed by the investigator to take, depending on the prescribed dose, 1 or 2 tablets every

morning, during breakfast. It is important to ensure that the patient does not forget to take the treatment on the day of the next visit.

Sleep diary will be delivered. The patient will answer to the sleep diary questions on three sequential days the of week preceding next visit. The next visit will be fixed in 4 weeks and the patient should bring back the completed diary and the used experimental treatment bottles.

12.1.6. V5 – Control visit: continuation of stable dose phase (D 49)

After the 4-week treatment period at the selected dose, the investigator will evaluate the efficacy and safety.

The visit will take place in the morning to ensure the identical condition for evaluation. It is important to ensure that the patient does not forget to take the treatment on the day of the visit.

The following assessments will be performed during this visit:

- Medical questionnaire including control of the treatment compliance by counting the remaining tablets and verification of the forgotten treatment intake.
- Physical examination, in particular vital signs of the patient.
- ESS.
- Patient's overall evaluation of the tolerance.
- ECG.
- Review of the sleep diary.
- Investigation of any adverse event occurrence.

Any dosage change of pitolisant or placebo will not be allowed at this visit. Hence, pitolisant or placebo treatment at the same stable dose will be continued for another 5 weeks.

Five corresponding bottles (one per week) will be given to the patient, and he/she will be instructed by the investigator to take, depending on the prescribed dose, 1 or 2 tablets every morning, during breakfast. It is important to ensure that the patient does not forget to take the treatment on the day of the next visit.

Sleep diary will be delivered. The patient will answer to the sleep diary questions on three sequential days of the week preceding next visit. The next visit will be fixed in 5 weeks and the patient should bring back the completed diary and the used experimental treatment bottles.

12.1.7. V6 – Evaluation visit at the end of the Double Blind period and start of single-blindwash-out period (D 84)

At this visit the patients are submitted to the tests and examinations required for the analysis of the double blind study results.

The patient will be required to come early in the morning at the hospital. It is important to ensure that the patient does not forget to take the treatment on the day of the visit.

The following assessments will be performed during this visit:

- Medical questionnaire including control of the treatment compliance by counting the remaining tablets and verification of the forgotten treatment intake.
- Physical examination, in particular vital signs of the patient.
- ESS.
- Three sessions of OSleR test at approximately 09:00, 11:00 and 13:00.
- TMT parts A and B.
- Clinical Global Impression – illness improvement or change (CGI-C).
- BDI-13.
- LSEQ.
- EQ-5D.
- Pichot Fatigue Scale.
- Patient's global opinion on the effect of investigational drugs.
- Patient's overall evaluation of the tolerance.
- ECG.
- Clinical laboratory tests: biological (hematology biochemistry) and serological (β-HCG pregnancy test for woman with child-bearing potential) tests.
- Review of the sleep diary.
- Investigation of any adverse event occurrence.

The Double Blind period of this study will be completed throughout this visit Patients will start one-week single blind placebo wash-out period.

One corresponding bottle will be given to the patient, and he/she will be instructed by the investigator to take, depending on the prescribed dose, 1 or 2 tablets every morning, during

breakfast. It is important to ensure that the patient does not forget to take the treatment on the day of the next visit.

Sleep diary will be delivered. The patient will answer to the sleep diary questions on three sequential days. The next visit will be fixed in 1 week and the patient should bring back the completed diary and the used experimental treatment bottle.

12.1.8. Ph2 – Phone contact (V6 + 3D)

During this phone contact, the investigator should get patient's answers to Amphetamine-Like Withdrawal Symptoms Questionnaire (DSM IV) to check the tolerance of the study drug withdrawal and find out patient's global opinion on the effect of investigational drugs. Also concomitant treatments and occurrence of AEs will be checked.

12.2. Part II - Open Label Extension period

12.2.1. V7 – End of study visit for patients who are not entering Open Label Extension period or Beginning of the Open Label Extension period for patients who are entering Open Label Extension period: start of new escalating dose phase (D 91)

The visit will take place in the morning to ensure the identical condition for evaluation. It is important to ensure that the patient does not forget to take the treatment on the day of the visit.

The following assessments will be performed during this visit:

- Medical questionnaire including control of the treatment compliance by counting the remaining tablets and verification of the forgotten treatment intake.
- Physical examination, in particular vital signs of the patient.
- ESS.
- Three sessions of OSleR test at approximately 09:00, 11:00 and 13:00 (only in patients entering OpenLabel Extension phase).
- TMT parts A and B (only in patients entering OpenLabel Extension phase).
- CGI-C.
- BDI-13.

- LSEQ (only in patients entering OpenLabel Extension phase).
- EQ-5D (only in patients entering OpenLabel Extension phase).
- Pichot Fatigue Scale.
- Patient's global opinion on the effect of investigational drugs.
- Patient's overall evaluation of the tolerance.
- Amphetamine-like withdrawal symptoms questionnaire (DSM IV).
- ECG.
- Review of V6 laboratory tests results.
- Review of the sleep diary.
- Investigation of any adverse event occurrence.

If the patient holds the same position towards nCPAP therapy as before, he/she will be proposed to enter the Open Label Extension period of the study after another escalating dose phase.

Patients not willing to continue the pitolisant treatment administration will end their participation in the study at this visit.

Patients willing to continue the pitolisant treatment administration will be given another information leaflet explaining the constraints and risks of the Open Label Extension study period together with an informed consent form to be signed. As soon as the patient gives the agreement by signing the informed consent form, he/she will be enrolled in this long-term follow-up period and will start the new escalating dose phase, as described hereunder.

Patients will receive one bottle containing 20 tablets of pitolisant 5 mg and one bottle containing 10 tablets of pitolisant 20 mg.

Patients will be instructed to take 2 tablets of pitolisant 5 mg OD during one week and 1 tablet of pitolisant 20 mg the next week OD (in the morning, during breakfast, with a glass of water). It is important to ensure that the patient does not forget to take the treatment on the day of the next visit.

Sleep diary will be delivered. The patient will answer to the sleep diary questions on three sequential days of the week preceding next visit. The next visit will be fixed in 2 weeks and the patient should bring back the completed diary and the used experimental treatment bottles.

12.2.2. V8-V9 – Dose adjustment visits (D 105 and D 112)

The visits will imperatively take place in the morning in order to ensure the identical conditions for evaluation. It is important to ensure that the patient does not forget to take the treatment on the days of the visits.

The following assessments will be performed during these visits:

- Medical questionnaire including control of the treatment compliance by counting the remaining tablets and verification of the forgotten treatment intake.
- Physical examination, in particular vital signs of the patient.
- ESS.
- BDI-13 (at V9).
- Pichot Fatigue Scale (at V9).
- Patient's overall evaluation of the tolerance.
- ECG.
- Review of the sleep diary.
- Investigation of any adverse event occurrence.

At each of the visits V8-V11 the investigator will propose nCPAP to the patients refusing to be treated by nCPAP therapy before giving them the study treatment. If the patient accepts, he/she will be withdrawn from the study.

The posology is increased to 40 mg/d pitolisant (high dose), every morning, during breakfast. If the tolerance does not allow it (i.e. troublesome insomnia), the patient will get the treatment at dose of 20 mg/d (medium dose) or 10 mg/d (low dose). At V9, "one-step" decrease from high dose to low dose or increase from low dose to high dose is not possible.

At V8, 1 bottle containing 20 tablets of pitolisant 5 mg, or 1 bottle containing 10 tablets of pitolisant 20 mg, or 1 bottle containing 20 tablets of pitolisant 20 mg will be given to the patient; at V9 bottles containing 30 tablets of pitolisant 5 mg or 30 tablets of pitolisant 20 mg, depending on selected posology, will be given to the patient in the quantity needed for prescribed treatment., The patient will be instructed by the investigator to take, depending on the prescribed dose, 1 or 2 tablets every morning, during breakfast. It is important to ensure that the patient does not forget to take the treatment on the next visit days.

Sleep diaries will be delivered. The patient will answer to the sleep diary questions on three sequential days. The next visit will be fixed in 1 week (V9), then in 12 weeks (V10), and the

patient should bring back the completed diary and the used experimental treatment bottles.

12.2.3. V10-V11 – Confirmed dose visits (D 196 and D 280)

The visits will occur in the morning, and the patient should be reminded not to forget to take the treatment on the visit days.

The following assessments will be performed during these visits:

- Medical questionnaire including control of the treatment compliance by counting the remaining tablets and verification of the forgotten treatment intake.
- Physical examination, in particular vital signs of the patient.
- ESS.
- CGI-C.
- BDI-13.
- Pichot Fatigue Scale.
- Patient's global opinion on the effect of investigational drugs.
- Patient's overall evaluation of the tolerance.
- ECG.
- Review of the sleep diary.
- Investigation of any adverse event occurrence.
- Pharmacokinetics sampling (at V10) – predose, 1,5, 3 and 8 h post administration.

The posology should be 40 mg/d (high dose) if the tolerance is acceptable. If the study drug is not well tolerated, the investigator may decide to decrease the dose to 20 mg/d (medium dose) or 10 mg/d (low dose) during the 12-weeks period following the visit. "One-step" decrease from high dose to low dose or increase from low dose to high dose is not possible.

At each visit bottles containing 30 tablets of pitolisant 5 mg or 30 tablets of pitolisant 20 mg, depending on selected posology, will be given to the patient in the quantity needed for prescribed treatment, and he/she will be instructed by the investigator to take, depending on the prescribed dose, 1 or 2 tablets every morning, during breakfast. It is important to ensure that the patient does not forget to take the treatment on the next visit days.

Sleep diaries will be delivered. The patient will answer to the sleep diary questions on three sequential days the of the week preceding next visit. The next visits will be fixed with 12

weeks interval and the patient should bring back the completed diary and the used experimental treatment bottles.

12.2.4. V12 – Evaluation visit at the end of the Open Label Extension period and start of wash-out period (D 364)

At this visit the patients are submitted to the examinations and tests required for the evaluation of long-term tolerance and maintenance of efficacy of pitolisant.

The patient will be required to come early in the morning at the hospital. It is important to ensure that the patient does not forget to take the treatment on the day of the visit.

The following assessments will be performed during this visit:

- Medical questionnaire including control of the treatment compliance by counting the remaining tablets and verification of the forgotten treatment intake.
- Physical examination, in particular vital signs of the patient.
- ESS.
- Optionally: three sessions of OSleR test at approximately 09:00, 11:00 and 13:00.
- TMT parts A and B.
- CGI-C.
- BDI-13.
- LSEQ.
- EQ-5D.
- Pichot Fatigue Scale.
- Patient's global opinion on the effect of investigational drugs.
- Patient's overall evaluation of the tolerance.
- ECG.
- Clinical laboratory tests: biological (hematology biochemistry) and serological (β-HCG pregnancy test for woman with child-bearing potential) tests.
- Review of the sleep diary.
- Investigation of any adverse event occurrence.

The Open Label Extension period of the study will be completed at this visit; the treatment intake will be stopped, patients will not receive any more experimental treatment in this study.

Sleep diary will be delivered. The patient will answer to the sleep diary questions on three sequential days. The next visit will be fixed in 1 weeks and the patient should bring back the completed diary.

12.2.5. Ph3 – Phone contact (V12 + 3D)

During this phone contact, the investigator should get patient's answers to amphetamine-like withdrawal symptoms questionnaire (DSM IV) to check the tolerance of the study drug withdrawal and find out patient's global opinion on the effect of investigational drugs. Also concomitant treatments and occurrence of AEs will be checked.

12.2.6. V13 – End of study visit for patients who entered into Open Label Extension period (D 371)

After a one-week wash-out period, this final visit will occur in the morning.

The following assessments will be performed during this visit:

- Medical questionnaire.
- Physical examination, in particular vital signs of the patient.
- ESS.
- CGI-C.
- BDI-13.
- Pichot Fatigue Scale.
- Patient's global opinion on the effect of investigational drugs.
- Patient's overall evaluation of the tolerance.
- Amphetamine-like withdrawal symptoms questionnaire (DSM IV).
- ECG.
- Review of V12 laboratory tests results.
- Review of the sleep diary.
- Investigation of any adverse event occurrence.

Thereafter, the investigator will decide about the new treatment to prescribe for the patient. And then the patient will be discharged from the study.

13. STATISTICAL ANALYSIS

13.1. Summary

Multicenter, randomised, double blind parallel groups study versus placebo during 3 months followed by a 9 months of Open Label Extension period with pitolisant.

The Full Analysis Set (FAS) constitutes the main selection, Per Protocol (PP) selection will be used as a secondary selection.

The main purpose of the study is to assess efficacy of pitolisant in decreasing daytime somnolence measured by the Epworth Sleepiness Scale. The secondary endpoints will include OSleR, and a Z-score aggregating OSleR and ESS.

Statistical Analysis: final ESS will be compared between the two treatments by an ANCOVA in adjusting for ESS at baseline, and by considering the random effect center, and the fixed effect treatment. This test will be implemented by a Mixed Linear Model.

Safety and tolerability will be assessed by summarizing and analyzing adverse events, change in physical examination, vital signs, electrocardiogram and laboratory data.

13.2. Sample Size Determination and Justification

Results from exploratory studies on pitolisant allow to estimate the ESS residual variability to standard deviation (SD) = 6. The Minimum Important Difference (MID) was fixed to ESS = 3, corresponding to an effect size (ES) = 0,5. The correlation between final and baseline ESS was conservatively estimated to r = 0,3.

By assuming ANCOVA at 0,95 confidence level as the main confirmatory test, a difference of at least Delta = 3 should be detected with a power of 90% in using at least 30 patients in each placebo group (60 in total) and 60 patients in each pitolisant treatment group (120 in total). Treatment groups will be stratified by center and CPAP use. Considering 10% drop out rate, 200 patients will be selected.

13.3. Primary and Secondary Populations

The primary population will be the Full Analysis Set (FAS) in conformity with intent to treat principle: All the randomized patients will be analyzed, irrespective of their outcome. The Per Protocol Analysis is defined as the subset of the FAS set constituted by the patients compliant

to the regimen and finishing on time.

13.4. Handling of Missing Data

The main endpoint will be calculated as the summary mean of the non missing values of ESS at visits 5 and 6. Missing values will be imputed using a pattern mixture model based on the assessment of the interaction between the variables completers/dropout linear model and treatment on the main endpoint. Dropouts before visit 5 will be imputed by baseline value when the patient is withdrawn from the trial for any reason related with drug adverse effects, or a manifest lack of efficacy, or Last Observed Carried Value, in any other case. In any case, this decision will be taken by the Scientific Committee.

13.5. Futility Analysis

A one-stage Futility stopping will be based on Conditional Power, probability to detect a significant result at the end of the Double Blind period, given the results observed at an intermediate time.

Conditional Power will be estimated (Lan and Wittes 1988; Lan and Zucker 1993). This analysis will be carried out by a third party statistician when at least 80 patients are available, and futility threshold will be CPmin=.10 involving a slight increase of type 2 error (Proschan 1999). This intermediate futility analysis does not require any type-I adjustment, this trial does not plan rejection of the null hypothesis before its end.

Further details will be described in the Statistical Analysis Plan (SAP) as appropriate.

An independent DSMB will regularly follow the progress of the clinical trial, monitor safety data and critical efficacy variables, and be consulted concerning the opportunity of modifying the sample size or terminate the trial for futility.

13.6. Statistical Analysis

The main endpoint will be calculated as the summary mean of the non missing values of ESS at visits 5 and 6. Final ESS will be compared between the two treatments by an ANCOVA at two-sided 95% in adjusting for ESS at baseline (mean of ESS of visits 1 and 2), and by considering the random effect center, and the fixed effect treatment and assessing the additional effect of obesity (BMI) on outcome[17, 18]. This test will be implemented by a Mixed

Linear Model.

The confirmatory analysis will be based on a simple ANCOVA model assuming no interaction between baseline and treatment (assumption of parallelism).

13.7. Pharmacokinetic Analysis

The pharmacokinetic analysis will be based on a population approach.

The proposed PK sampling scheme was optimized according to a previous knowledge on pitolisant PK and an initial population PK model developed in healthy subjects. Parameters of this initial population model were used together with study constraints (i.e. limitation of the number of PK blood samples and PK follow-up limited to a maximum of 8 h post-dose). The PFIM software was used for sampling time optimization and determination of population sample size for valuable analysis.

An optimized PK blood sampling times of 4 samples requiring a minimum population of 90 patients was defined to be applied in the present study.

Pitolisant concentrations will be populated with dosing information data and selected covariates in order to be analysed using NONMEM® software for nonlinear mixed model.

The population PK model will be composed of:

- a structural part, defining the general pattern of concentrations time course, parameterized in terms of apparent clearance, volume of distributions, etc.
- a random part, split in inter and intra-individual variability
- a covariate model in which influence of covariates on PK parameters and variability will be investigated.

Methods to be used for the population PK analysis will be fully detailed in a specific analysis plan.

14. DATA MANAGEMENT

14.1. Collection of data

The data of each patient will be collected in a CRF bearing the inclusion number which is the patient number in the study. The following data will be collected:

- Demographic data: age, weight, height, birth date (month/year), medical history

- Results of patient physical examinations, including vital signs
- Results of ESS
- Results of OSleR test
- Results of TMT Parts A & B
- Results of MMSE
- Results of CGI-S and CGI-C
- Results of BDI-13
- Results of LSEQ, EQ-5D and Pichot Fatigue Scale
- Patient's global opinion on the effect of investigational drugs
- Patient's overall evaluation of the tolerance
- Results of amphetamine-like withdrawal symptoms questionnaire (DSM IV)
- ECG
- Results of laboratory tests
- Results of the polysomnographic examination
- Records of sleep diary
- Adverse Events reports
- Administered treatment
- Compliance to treatment and to nCPAP (for patients on nCPAP therapy)

Data collected in the CRFs will be transferred to electronic database.

The database will be validated through computer comparison of the two files and by tests of coherence with computer software, according to the data management book (DMB).

14.2. Archiving of data, Audit

In order to constitute evidence with respect to product safety or regulatory or legal compliance, the investigators, investigational sites and Ethics Committees agree to retain study-related documents in a location that is secure and to which access can be gained if required.

The investigator and the site should retain records for a minimum of 15 years or a delay according to the local regulatory requirement.

At the end of these regulatory timelines, the investigator will inform Bioprojet of his intention to proceed with the destruction of archived data.

If the records need to be retained after that duration, the investigator and the site will be notified by Bioprojet.

These documents are to be available for inspection by authorized representative of Bioprojet or regulatory authorities. Audits may be performed for quality assurance of data handling.

The following records must be retained by the investigator for a minimum of 15 years or a delay according to the local regulatory requirement, after the sponsor has notified the Authorities that the study is completed:

- Signed informed consent documents for all patients
- Patient identification code list and enrolment log
- Record of all communications between the investigator and the EC/IRB
- Composition of the EC/IRB (or other local applicable regulation)
- Record of all communications between the investigator and sponsor (or CRO)
- List of sub-investigators and other appropriately qualified persons to whom the investigator has delegated significant trial-related duties, together with their roles in the study and their signatures
- Copies of case report forms and of documentation of corrections for all patients
- Drug accountability record
- All other source and essential study documents (patient records, hospital records, laboratory records, etc...)

The records should be held in the investigator's archives. However, if the investigator is unable to meet this obligation, he or she must ask the sponsor for permission to make alternative arrangements. Details of these arrangements should be documented.

14.3. Data Protection and Confidentiality

All documents that concern the studied medication and the company's operations belonging to sponsor such as patent applications, formula, manufacturing process, basic scientific data and analysis bulletins; information supplied by the company and not previously published are considered confidential and shall remain the sole property of the sponsor.

The investigator agrees to use this information only in accomplishing this study and they will

not use it for other purposes without written consent from sponsor.

Confidentiality of study source documents

The information included in this document, as the investigator's brochure of the product, the CRF and the results of the present study are considered as confidential and should not be divulged, only in case of legal requirements.

In any event, persons to whom the information is disclosed must be informed that the information is privileged or confidential and may not be further disclosed by them. The signature of investigator in the present protocol is equivalent to a confidential agreement.

It is understood by the investigator that the information from the clinical study will be used by the company in connection with the development of the tested drug and, therefore, may be disclosed as required to other clinical investigators or to government agencies. In order to allow for the use of the information derived from the clinical studies, it is understood that there is an obligation to provide the sponsor with the complete test results as well as all data developed during this study, under the form of a written document or computerized with the following software: Word or SAS under Windows, saved on CD-Rom.

The study drug and the information in this document and in any future information supplied contain trade secrets and commercial information that are privileged or confidential and may not be disclosed unless such disclosure is required by law or regulations.

All or part of the information should only be divulged, submitted for publication or claim for industrial proprietary act with the written consent of Bioprojet.

According to the French law "Computer Information and Liberty", dated January 6th, 1978 modified by the law N°2004-801 of August, 6th 2004 and the decree N°2005-1309 of October 20th 2005, Bioprojet committed on July 28th, 2006 to comply with the methodology MR001 for all electronic and computerized data relative to this study .

15. QUALITY ASSURANCE

15.1. Good clinical Practice

The trial will be run with respect to this protocol, according to the Good Clinical Practice (GCP), following the international regulations (ICH) and the European directives (EMEA) or national laws.

The quality control of the study and the audit/inspection of the Good Clinical Practice could be performed by Bioprojet and its Department of Quality Assurance or by an inspector of the Ministry of Health.

In particular, the current protocol will be submitted to the agreement of the Ethics Committee and Competent Authority by Bioprojet. The conformity of the study progress will be reviewed.

In case of an amendment, it has to be submitted for agreement to the Ethics committee and Competent Authority as appropriate. The Ethics Committee will be consulted through notification for any minor protocol modification.

In case of major written modification (modification that could jeopardize the protection of the people participating to the trial and modifications likely to invalidate the scientific validity of the study), the protocol will be re-submitted to the agreement of the Ethics Committee and Competent Authority.

The investigator will carefully explain the participating conditions to each proposed patient.

Each patient will receive a detailed and written information letter including the name of the product, a summary of its properties, its potential benefit, the unexpected and adverse events, the doses to be administered, the treatment duration, the number of visits, the kind and number of scheduled exams. This information letter will be submitted beforehand to the agreement of the Ethics committee.

The patient will be informed that he can dropout from the study whenever he wants, without any justification.

The system to attribute numbers to patients is based on the anonymity obligation. Patients will receive a number according to their order of inclusion in the study.

The patients will be identified by the sponsor with: the patient number and their date of birth.

Study progress conformity to the protocol will be controlled from the patients' selection, and all along the study. Each investigator has to allow the monitor, or sponsor's mandated representative, the direct access to any control of the study progress, and to any required documents to control data reported in case report forms (hospitalization file, consultation file, results of additional examinations, etc.).

The medical project manager of the study will be reachable, weekend included, at:

PPDPPDPPD

Bioprojet – 9, Rue Rameau – 75002 Paris, France
PPDPPDPPD
PPDPPDPPD
PPDPPDPPDPPDPPD

The investigator will be available to the telephone for each patient whom he will have included.

The anonymity respect shall be applied to the filling of the case report form as to any other archived documents considered as source data (blood tests, informed consent form, etc.).

15.2. Premature closure of the study

The sponsor or the investigator has the right to close this study at any time for valid scientific or administrative reasons, and reasons related to patients' protection. As far as possible, this should occur after mutual consultation. The Competent Authorities (CA) and the EC/IRB must be informed, if required by legislation.

Should the study be closed prematurely, all study materials (completed, partially completed, and blank case report forms, study medication, etc.) must be returned to the sponsor, as if the study had been completed.

15.2.1. Criteria for terminating the trial

Reasons for the study termination may include but are not limited to:

- The discovery of an unexpected, significant or unacceptable risk to patients enrolled in the study
- A decision of Bioprojet to suspend or discontinue the development of the investigational product
- A decision of Bioprojet to suspend after analysis of futility
- Request of the relevant regulatory agency

15.2.2. Criteria for terminating an investigational site

Bioprojet reserves the right to terminate the study at a given investigational site at any time after the study initiation if:

- ICH GCP regulations are not fulfilled

- The protocol is violated without justification
- The data generated are of poor quality

15.3. Control – Quality

Control actions are implemented within the framework of the Quality Insurance System to check that the quality requirements of the study are respected.

The original documents generated in the course of the study will be controlled at each step of the study, both by the sponsor's representative and the investigator, in order to guarantee the accuracy of the analyzed data.

Internal audit systems can be conducted during the study by the sponsor's representative or by an independent organism.

They will enable to check that the study is being run in accordance with the protocol and to current rules and regulations.

16. REPORTING AND PUBLICATION OF RESULTS

The investigator commits himself to:

- Keep the Sponsor (Bioprojet Medical project manager) posted on the study results in written form.
- Review and sign the clinical report of the study (ICH format) for Bioprojet.
- The communication or publication of all or part of the results of this study will be only permitted after written agreement of Bioprojet. The publication or communication shall mention the origin of the substance.

17. CONTRACT, LIABILITY AND INSURANCE

A financial agreement will be made between the parties, institution, investigator and the sponsor, in accordance with each administrative procedure. All agreed costs will be described in the parties' signed contracts before the start of the study

Bioprojet will contract a specific insurance policy for the coverage of the patients in compliance with each National regulation. Liability and insurance provisions for this study are given in the investigator's contract.

18. FINANCIAL DISCLOSURE

Before the start of the study, the investigator will disclose to the sponsor any proprietary or financial interests he or she might hold in the investigational product or the sponsor company as outlined in the financial disclosure form provided by the sponsor. The investigator agrees to update this information in case of significant changes during the study or within one year of its completion. The investigator also agrees that, where required by law or regulation, the sponsor may submit this financial information to domestic or foreign regulatory authorities in applications for marketing authorizations.

Similar information will be provided by each sub-investigator to whom the investigator delegates significant study-related responsibilities.

19. CALENDAR FORECAST

First patient visit is scheduled in 1-2 Q 2016

Last patient visit is scheduled in 2 Q 2017

20. REFERENCES

1) Mc Nicolas W. T., Diagnosis of obstructive sleep apnea in adults. Proc Am Thorax Society 2008; 5: 154-160
2) Young T, Peppard P. E, Gottlieb D. J, Epidemiology of obstructive sleep apnea, A population health perspective. Am J Resp Crit Care Med 2002; 165: 1217-1239
3) Nieto F, Young T, Lind B, et al, Association of sleep-disordered breathing, sleep apnea, and hypertension in a large community-based study. JAMA 2000; 283:1829-1836
4) Peppard P, Young T, Palta M, et al, Prospective study of the association between sleep-disordered breathing and hypertension. N Engl J Med 2000; 342: 1378-1384
5) Bizieux-Thaminy A, Gagnadoux F, Binquet C, Meslier N, Person C, Racineux J.L, Long-term use of nCPAP therapy in sleep apnea patients. Rev Mal Respir 2005; 22:00-00
6) Felever-Grant J.C, Bruce A. S, Zimmerman M et al, Working memory in obstructive sleep apnea: construct validity and treatments effects. J Clin Sleep Med 2007; 3 (6): 589-594
7) Loube D, Gay P, Strohl K, et al, indications for positive airway pressure treatment of adult obstructive sleep apnea patients: a consensus statement. Chest 1999; 115: 863-866
8) Strollo P. J, Rogers R. Obstructive sleep apnea. N Engl J Med 1996; 334:99-104

9) Kushida C. A, Littner M. R, Hirshkowitz M et al, Practice parameters for the use of continuous and bilevel positive airway pressure devices to treat adult patients with sleep-related breathing disorders. Sleep 2006; 29 (3): 375-380
10) Kushida C. A, Chediak A, Berry R. B, Brown L. K, Grozal D, Iber C, Parthasarathy S, Quan S. F, Rowley J. A. Positive airway pressure titration task force of the American Academy of Sleep Medicine. Clinical guidelines for the manual titration of positive airway pressure in patients with obstructive sleep apnea. J Clin Sleep Med 2008; 4(2): 157-171
11) Schwartz J.R.L, Hirshkowitz M, Erman E. K, Schimdt-Nowara W, Modafinil as adjunct therapy for daytime sleepiness in obstructive sleep apnea. Chest 2003; 124: 2192-21999
12) Douglas N.J, Engleman H, CPAP therapy: Outcomes and patient use. Thorax 1998; 53 (Suppl 3): S47-48
13) Garcia-Diaz E, Quintana-Gallego E, Ruiz A, Carmona-Bernal C, Sanchez-Armengol A, Benhamou-Botebol G et al, Respiratory polygraphy with actigraphy in the diagnosis of sleep apnea-hypoapnea syndrome. Chest 2007; 131: 725-732
14) Woodford H.J and Georges J, Cognitive assessment in the elderly: a review of clinical methods; Q j Med 2007; 100: 469-484
15) Mazza S, Pepin J.L, Deschaux C, Naegele B, Levy P, Analysis of Error Profiles occurring during the OSLER test. Am J Respir Crit Care Med 2002; 166: 474-478
16) Hindmarch I, A 1, 4 benzodiazepine, temazepam (K3917): its effect on some psychological parameters of sleep and behaviour. Arzneimittel-Forschung (Drug research) 1975; 25 (11): 1836-1839
17) Schwartz A R, Patil S P, Laffan A M, Polotsky V, Schneider H, Smith P L, Obesity and obstructive sleep apnea. Pathogenic mechanisms and therapeutic approaches. Proc Am Thor Soc 2008; 5: 185-192
18) Shiroh Isono, Obstructive sleep apnea of obese adults. Pathophysiololgy and perioperative airway management. Anaesthesiology 2009; 110 (4): 908-921

21. APPENDIX SECTION

Appendix 1: Declaration of Helsinki

Appendix 2: Full-night polysomnography

Appendix 3: Epworth Sleepiness Scale (ESS)

Appendix 4: Sleep diary

Appendix 5: European Quality of Life questionnaire (EQ-5D)

Appendix 6: Leeds Sleep Evaluation Questionnaire (LSEQ)

Appendix 7: Pichot Fatigue Scale

Appendix 8: Trail Making Test (TMT) parts A & B

Appendix 9: Clinical Global Impression (CGI) rating scale

Appendix 10: Patient's global opinion on the effect of investigational drugs

Appendix 11: DSMB charter

Appendix 12: Instructions for filling in Bioprojet SAE Report Form

Appendix 13: Beck depression Inventory – 13 items (BDI-13)

Appendix 13: Patient's overall evaluation of the tolerance

Appendix 15: Amphetamine-like withdrawal symptoms questionnaire (DSM IV)

Appendix 16: Mini Mental State Examination questionnaire (MMSE)

Appendix 1: Declaration of Helsinki

Adopted by the 18th WMA General Assembly, Helsinki, Finland, June 1964
and amended by the:
29th WMA General Assembly, Tokyo, Japan, October 1975
35th WMA General Assembly, Venice, Italy, October 1983
41st WMA General Assembly, Hong Kong, September 1989
48th WMA General Assembly, Somerset West, Republic of South Africa, October 1996
52nd WMA General Assembly, Edinburgh, Scotland, October 2000
53rd WMA General Assembly, Washington DC, USA, October 2002 (Note of Clarification added)
55th WMA General Assembly, Tokyo, Japan, October 2004 (Note of Clarification added)
59th WMA General Assembly, Seoul, Republic of Korea, October 2008
64th WMA General Assembly, Fortaleza, Brazil, October 2013

Preamble

1. The World Medical Association (WMA) has developed the Declaration of Helsinki as a statement of ethical principles for medical research involving human subjects, including research on identifiable human material and data.

The Declaration is intended to be read as a whole and each of its constituent paragraphs should be applied with consideration of all other relevant paragraphs.

2. Consistent with the mandate of the WMA, the Declaration is addressed primarily to physicians. The WMA encourages others who are involved in medical research involving human subjects to adopt these principles.

General Principles

3. The Declaration of Geneva of the WMA binds the physician with the words, “The health of my patient will be my first consideration,” and the International Code of Medical Ethics declares that, “A physician shall act in the patient's best interest when providing medical care.”

4. It is the duty of the physician to promote and safeguard the health, well-being and rights of patients, including those who are involved in medical research. The physician's knowledge and conscience are dedicated to the fulfilment of this duty.

5. Medical progress is based on research that ultimately must include studies involving human subjects.

6. The primary purpose of medical research involving human subjects is to understand the causes, development and effects of diseases and improve preventive, diagnostic and therapeutic interventions (methods, procedures and treatments). Even the best proven interventions must be evaluated continually through research for their safety, effectiveness,

efficiency, accessibility and quality.

7. Medical research is subject to ethical standards that promote and ensure respect for all human subjects and protect their health and rights.

8. While the primary purpose of medical research is to generate new knowledge, this goal can never take precedence over the rights and interests of individual research subjects.

9. It is the duty of physicians who are involved in medical research to protect the life, health, dignity, integrity, right to self-determination, privacy, and confidentiality of personal information of research subjects. The responsibility for the protection of research subjects must always rest with the physician or other health care professionals and never with the research subjects, even though they have given consent.

10. Physicians must consider the ethical, legal and regulatory norms and standards for research involving human subjects in their own countries as well as applicable international norms and standards. No national or international ethical, legal or regulatory requirement should reduce or eliminate any of the protections for research subjects set forth in this Declaration.

11. Medical research should be conducted in a manner that minimises possible harm to the environment.

12. Medical research involving human subjects must be conducted only by individuals with the appropriate ethics and scientific education, training and qualifications. Research on patients or healthy volunteers requires the supervision of a competent and appropriately qualified physician or other health care professional.

13. Groups that are underrepresented in medical research should be provided appropriate access to participation in research.

14. Physicians who combine medical research with medical care should involve their patients in research only to the extent that this is justified by its potential preventive, diagnostic or therapeutic value and if the physician has good reason to believe that participation in the research study will not adversely affect the health of the patients who serve as research subjects.

15. Appropriate compensation and treatment for subjects who are harmed as a result of participating in research must be ensured.

Risks, Burdens and Benefits

16. In medical practice and in medical research, most interventions involve risks and burdens. Medical research involving human subjects may only be conducted if the importance of the objective outweighs the risks and burdens to the research subjects.

17. All medical research involving human subjects must be preceded by careful assessment of predictable risks and burdens to the individuals and groups involved in the research in comparison with foreseeable benefits to them and to other individuals or groups affected by

the condition under investigation.
Measures to minimise the risks must be implemented. The risks must be continuously monitored, assessed and documented by the researcher.
18. Physicians may not be involved in a research study involving human subjects unless they are confident that the risks have been adequately assessed and can be satisfactorily managed.
When the risks are found to outweigh the potential benefits or when there is conclusive proof of definitive outcomes, physicians must assess whether to continue, modify or immediately stop the study.

Vulnerable Groups and Individuals
19. Some groups and individuals are particularly vulnerable and may have an increased likelihood of being wronged or of incurring additional harm.
All vulnerable groups and individuals should receive specifically considered protection.
20. Medical research with a vulnerable group is only justified if the research is responsive to the health needs or priorities of this group and the research cannot be carried out in a non-vulnerable group. In addition, this group should stand to benefit from the knowledge, practices or interventions that result from the research.
Scientific Requirements and Research Protocols
21. Medical research involving human subjects must conform to generally accepted scientific principles, be based on a thorough knowledge of the scientific literature, other relevant sources of information, and adequate laboratory and, as appropriate, animal experimentation.
The welfare of animals used for research must be respected.
22. The design and performance of each research study involving human subjects must be clearly described and justified in a research protocol.
The protocol should contain a statement of the ethical considerations involved and should indicate how the principles in this Declaration have been addressed. The protocol should include information regarding funding, sponsors, institutional affiliations, potential conflicts of interest, incentives for subjects and information regarding provisions for treating and/or compensating subjects who are harmed as a consequence of participation in the research study.
In clinical trials, the protocol must also describe appropriate arrangements for post-trial provisions.

Research Ethics Committees
23. The research protocol must be submitted for consideration, comment, guidance and approval to the concerned research ethics committee before the study begins. This committee must be transparent in its functioning, must be independent of the researcher, the sponsor and any other undue influence and must be duly qualified. It must take into consideration the laws

and regulations of the country or countries in which the research is to be performed as well as applicable international norms and standards but these must not be allowed to reduce or eliminate any of the protections for research subjects set forth in this Declaration.
The committee must have the right to monitor ongoing studies. The researcher must provide monitoring information to the committee, especially information about any serious adverse events. No amendment to the protocol may be made without consideration and approval by the committee. After the end of the study, the researchers must submit a final report to the committee containing a summary of the study's findings and conclusions.

Privacy and Confidentiality

24. Every precaution must be taken to protect the privacy of research subjects and the confidentiality of their personal information.

Informed Consent

25. Participation by individuals capable of giving informed consent as subjects in medical research must be voluntary. Although it may be appropriate to consult family members or community leaders, no individual capable of giving informed consent may be enrolled in a research study unless he or she freely agrees.

26. In medical research involving human subjects capable of giving informed consent, each potential subject must be adequately informed of the aims, methods, sources of funding, any possible conflicts of interest, institutional affiliations of the researcher, the anticipated benefits and potential risks of the study and the discomfort it may entail, post-study provisions and any other relevant aspects of the study. The potential subject must be informed of the right to refuse to participate in the study or to withdraw consent to participate at any time without reprisal. Special attention should be given to the specific information needs of individual potential subjects as well as to the methods used to deliver the information.
After ensuring that the potential subject has understood the information, the physician or another appropriately qualified individual must then seek the potential subject's freely-given informed consent, preferably in writing. If the consent cannot be expressed in writing, the non-written consent must be formally documented and witnessed.
All medical research subjects should be given the option of being informed about the general outcome and results of the study.

27. When seeking informed consent for participation in a research study the physician must be particularly cautious if the potential subject is in a dependent relationship with the physician or may consent under duress. In such situations the informed consent must be sought by an appropriately qualified individual who is completely independent of this relationship.

28. For a potential research subject who is incapable of giving informed consent, the physician must seek informed consent from the legally authorised representative. These

individuals must not be included in a research study that has no likelihood of benefit for them unless it is intended to promote the health of the group represented by the potential subject, the research cannot instead be performed with persons capable of providing informed consent, and the research entails only minimal risk and minimal burden.

29. When a potential research subject who is deemed incapable of giving informed consent is able to give assent to decisions about participation in research, the physician must seek that assent in addition to the consent of the legally authorised representative. The potential subject's dissent should be respected.

30. Research involving subjects who are physically or mentally incapable of giving consent, for example, unconscious patients, may be done only if the physical or mental condition that prevents giving informed consent is a necessary characteristic of the research group. In such circumstances the physician must seek informed consent from the legally authorised representative. If no such representative is available and if the research cannot be delayed, the study may proceed without informed consent provided that the specific reasons for involving subjects with a condition that renders them unable to give informed consent have been stated in the research protocol and the study has been approved by a research ethics committee. Consent to remain in the research must be obtained as soon as possible from the subject or a legally authorised representative.

31. The physician must fully inform the patient which aspects of their care are related to the research. The refusal of a patient to participate in a study or the patient's decision to withdraw from the study must never adversely affect the patient-physician relationship.

32. For medical research using identifiable human material or data, such as research on material or data contained in biobanks or similar repositories, physicians must seek informed consent for its collection, storage and/or reuse. There may be exceptional situations where consent would be impossible or impracticable to obtain for such research. In such situations the research may be done only after consideration and approval of a research ethics committee.

Use of Placebo

33. The benefits, risks, burdens and effectiveness of a new intervention must be tested against those of the best proven intervention(s), except in the following circumstances:

Where no proven intervention exists, the use of placebo, or no intervention, is acceptable; or

Where for compelling and scientifically sound methodological reasons the use of any intervention less effective than the best proven one, the use of placebo, or no intervention is necessary to determine the efficacy or safety of an intervention and the patients who receive any intervention less effective than the best proven one, placebo, or no intervention will not be subject to additional risks of serious or irreversible harm as a result of not receiving the best proven intervention.

Extreme care must be taken to avoid abuse of this option.

Post-Trial Provisions

34. In advance of a clinical trial, sponsors, researchers and host country governments should make provisions for post-trial access for all participants who still need an intervention identified as beneficial in the trial. This information must also be disclosed to participants during the informed consent process.

Research Registration and Publication and Dissemination of Results

35. Every research study involving human subjects must be registered in a publicly accessible database before recruitment of the first subject.

36. Researchers, authors, sponsors, editors and publishers all have ethical obligations with regard to the publication and dissemination of the results of research. Researchers have a duty to make publicly available the results of their research on human subjects and are accountable for the completeness and accuracy of their reports. All parties should adhere to accepted guidelines for ethical reporting. Negative and inconclusive as well as positive results must be published or otherwise made publicly available. Sources of funding, institutional affiliations and conflicts of interest must be declared in the publication. Reports of research not in accordance with the principles of this Declaration should not be accepted for publication.

Unproven Interventions in Clinical Practice

37. In the treatment of an individual patient, where proven interventions do not exist or other known interventions have been ineffective, the physician, after seeking expert advice, with informed consent from the patient or a legally authorised representative, may use an unproven intervention if in the physician's judgement it offers hope of saving life, re-establishing health or alleviating suffering. This intervention should subsequently be made the object of research, designed to evaluate its safety and efficacy. In all cases, new information must be recorded and, where appropriate, made publicly available.

Informed Consent Process

- Information provided to the patient by the site
- Review and signature of Informed Consent
- One copy is kept at the site
- One copy is given to the patient

Appendix 2: Full-Night Polysomnography

Polysomnography is a standard full-night diagnostic study. Continuous recordings of the electroencephalogram (C3/A2-C4/A1-CZ/O1 from the International 10-20 Electrode Placement System), eye movement measurements, chin electromyogram (EMG) and electrocardiogram (ECG). Respiratory effort is monitored with uncalibrated inductance respiratory plethysmography. Airflow is measured with nasal pressure, as well as with the sum of buccal and nasal thermistor signals. An additional signal of respiratory effort (i.e., pulse transit time or oesophageal pressure) is recorded concurrently. Oxygen saturation is measured using a pulse oximeter (Biox- Ohmeda 3700; Ohmeda; Liberty Corner, NJ).

The polysomnogram is scored manually according to standard criteria (*).

Episodes of apnea are defined as complete cessations of airflow for more than 10 s; episodes of hypopnoea as decreases in oronasal airflow of more than 50% and lasting at least 10 s, a decrease of more than 30% associated with a decrease in oxygen saturation of more than 3%, or a microarousal. Apnea/hypopnoea events are classified as central, obstructive, or mixed depending on the absence or presence of breathing efforts.

**Rechtshaffen, A., and A. Kales. 1968. A manual of standardized terminology, technique and scoring system for sleep stages of human sleep. Brain Information Service, Brain Information Institute, University of California, Los Angeles.*

The Polysomnography will be performed between V1 and the inclusion visit V2 (if no polysomnography was performed during the previous 12 months).

Appendix 3: Epworth Sleepiness Scale (ESS)

The Epworth Sleepiness Scale is used to determine the level of daytime sleepiness. A score of 10 or more is considered sleepy. A score of 18 or more is very sleepy. If you score 10 or more on this test, you should consider whether you are obtaining adequate sleep, need to improve your sleep hygiene and/or need to see a sleep specialist. These issues should be discussed with your personal physician.

Use the following scale to choose the most appropriate number for each situation:

0 *= Would never doze or sleep*

1 *= Slight chance of dozing or sleeping*

2 *= Moderate chance of dozing or sleeping*

3 *= High chance of dozing or sleeping*

Print out this test, fill in your answers and see where you stand.

SITUATION	CHANCE OF DOZING OR FALLING ASLEEP
Sitting and reading	____
Watching TV	____
Sitting inactive in a public place	____
Being a passenger in a motor vehicle for an hour or more	____
Lying down in the afternoon	____
Sitting and talking to someone	____
Sitting quietly after lunch (no alcohol)	____
Stopped for a few minutes in traffic while driving	____
**Total score (add the scores up)**<br>(This is your Epworth score)	____

Appendix 4: Sleep Diary

BIOPROJET	Protocol P 09-09 / BF 2.649	Patient Number: ⌊_⌊_⌋	Centre Number: ⌊_⌊_⌋	SLEEP DIARY N°: ⌊_⌊_⌋ *First day of filling*

1. Complete the below grid along the day according to the following codes :

- ↓ Go to bed ↑ Get up (to begin the day)
- Hatch the sleep periods (diurnal and nocturnal) //////////////// and keep in blank the waking periods
- Indicate the sleepiness episode by a ⋆
- Indicate your sudden on sleep episode by a ●

Example

Date (DD/MM/YY)	Study treatment intake
Example: 31/10/06	10h00
To be completed by the patient before going to sleep	

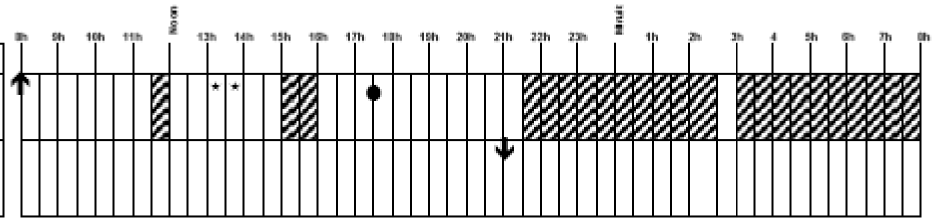

2. Please complete the following information according to the above grid:

- **What time did you wake up this morning? :** ⌊_⌊_⌋h⌊_⌊_⌋ min
- **What time did you get up this morning? :** ⌊_⌊_⌋h⌊_⌊_⌋ min
- **Number of episodes of sleep and sleepiness during the day** (naps and ⋆) : ⌊_⌊_⌋
- **Total duration of sleep and sleepiness during the day**(naps and ⋆) : ⌊_⌊_⌋h⌊_⌊_⌋ min
- **What time did you go to sleep last night? :** ⌊_⌊_⌋h⌊_⌊_⌋ min
- **What is the estimated time of your falling asleep last night? :** ⌊_⌊_⌋h⌊_⌊_⌋ min
- **Number of awakening episodes during last night (●):** ⌊_⌊_⌋
- **Total duration of these awakening episodes during last night.:** ⌊_⌊_⌋h⌊_⌊_⌋ min

Appendix 5: European Quality of Life Questionnaire (EQ-5D)

Date: __ __ / __ __ __/__ __ __ __

Visit: ☐ **Baseline**

EQ - 5D **Health Questionnaire**
(English version for the UK) *- (validated for use in Eire)*

By placing a tick in one box in each group below, please indicate which statements best describe your own health state today.

Mobility

I have no problems in walking about ☐

I have some problems in walking about ☐

I am confined to bed ☐

Self-Care

I have no problems with self-care ☐

I have some problems washing or dressing myself ☐

I am unable to wash or dress myself ☐

Usual Activities *(e.g. work, study, housework, family or leisure activities)*

I have no problems with performing my usual activities ☐

I have some problems with performing my usual activities ☐

I am unable to perform my usual activities ☐

Pain/Discomfort

I have no pain or discomfort ☐

I have moderate pain or discomfort ☐

I have extreme pain or discomfort ☐

Anxiety/Depression

I am not anxious or depressed ☐

I am moderately anxious or depressed ☐

I am extremely anxious or depressed ☐

To help people say how good or bad a health state is, we have drawn a scale (rather like a thermometer) on which the best state you can imagine is marked 100 and the worst state you can imagine is marked 0.

We would like you to indicate on this scale how good or bad your own health is today, in your opinion. Please do this by drawing a line from the box below to whichever point on the scale indicates how good or bad your health state is today.

Your own health state today

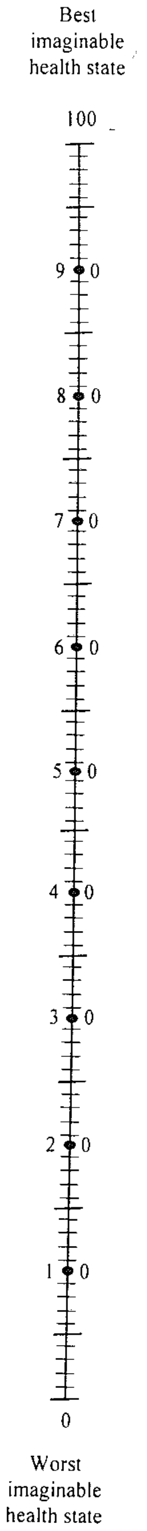

Appendix 6: Leeds Sleep Evaluation Questionnaire (LSEQ)

Leeds Sleep Evaluation Questionnaire

How would you describe the way you currently fall asleep in comparison to usual?

1. More difficult than usual ________________ Easier than usual
2. Slower than usual ________________ More quickly than usual
3. I feel less sleepy than usual ________________ More sleepy than usual

GTS - getting to sleep

How would you describe the quality of your sleep compared to normal sleep?

4. More restless than usual ________________ Calmer than usual
5. With more wakeful periods than usual ________________ With less wakeful periods than usual

QOS - quality of sleep

How would you describe your awakening in comparison to usual?

6. More difficult than usual ________________ Easier than usual
7. Requires a period of time longer than usual ________________ Shorter than usual

AFS – Awake following sleep

How do you feel when you wake up?

8. Tired ________________ Alert

How do you feel now?

9. Tired ________________ Alert

BFW – behaviour following wakening

How would you describe your balance and co-ordination upon awakening?

10. More disrupted than usual ________________ Less disrupted than usual

Appendix 7: Pichot Fatigue Scale

(Ref. "Scales and tools of evaluation in general medicine" J. Gardenas and Coll. - The General practitioner Supplement of N°2187; March 2002).

Fatigue is a feeling of physical or psychic decline which normally arises after a sustained effort, and which makes it necessary to take a rest.
We speak about pathological fatigue whenever the person feels at a disadvantage with regard to his/her usual physical condition to carry out his/her daily activities.

Pichot subjective scale was proposed to measure the importance of this impairment.

First name: .... Name: .... Date of birth: ....
Date of test: ....Current Treatment ……..................................... ....

Among the following eight suggestions, determine those who best correspond to your state by scoring each item between 0 and 4:

(0 = not at all; 1= a little, 2 = moderately, 3= much, 4 = extremely)

- I feel short of stamina... 0 1 2 3 4
- Everything I do requires a huge effort.......................................0 1 2 3 4
- I have a feeling of weakness in certain parts of my body….......0 1 2 3 4
- My arms or legs are heavy……………………… 0 1 2 3 4
- I feel tired for no reason…………………..............................0 1 2 3 4
- I feel like lying down or resting ……………………….............0 1 2 3 4
- I have difficulty to concentrate 0 1 2 3 4
- I feel discouraged, my arms and legs are sore and heavy 0 1 2 3 4

Total (out of 32):.........................

A score <u>higher than 22</u> is in favour of an excessive fatigue; you are perhaps suffering from an inefficient sleep.

NB. This questionnaire is used to measure your general level of Fatigue and cannot be considered as a medical diagnosis. Bring it to your doctor to discuss the causes and consequences of this fatigue in your life.

Appendix 8: Trail Making Test (TMT) Parts A & B

Trail Making Test (TMT) Parts A & B

Instructions:

Both parts of the Trail Making Test consist of 25 circles distributed over a sheet of paper. In Part A, the circles are numbered 1 – 25, and the patient should draw lines to connect the numbers in ascending order. In Part B, the circles include both numbers (1 – 13) and letters (A – L); as in Part A, the patient draws lines to connect the circles in an ascending pattern, but with the added task of alternating between the numbers and letters (i.e., 1-A-2-B-3-C, etc.). The patient should be instructed to connect the circles as quickly as possible, without lifting the pen or pencil from the paper. Time the patient as he or she connects the "trail." If the patient makes an error, point it out immediately and allow the patient to correct it. Errors affect the patient's score only in that the correction of errors is included in the completion time for the task. It is unnecessary to continue the test if the patient has not completed both parts after five minutes have elapsed.

Step 1: Give the patient a copy of the Trail Making Test Part A worksheet and a pen or pencil.

Step 2: Demonstrate the test to the patient using the sample sheet (Trail Making Part A – *SAMPLE*).

Step 3: Time the patient as he or she follows the "trail" made by the numbers on the test.

Step 4: Record the time.

Step 5: Repeat the procedure for Trail Making Test Part B.

Scoring:

Results for both TMT A and B are reported as the number of seconds required to complete the task; therefore, higher scores reveal greater impairment.

	Average	Deficient	Rule of Thumb
**Trail A**	29 seconds	> 78 seconds	Most in 90 seconds
**Trail B**	75 seconds	> 273 seconds	Most in 3 minutes

Sources:

- Corrigan JD, Hinkeldey MS. Relationships between parts A and B of the Trail Making Test. *J Clin Psychol.* 1987;43(4):402–409.
- Gaudino EA, Geisler MW, Squires NK. Construct validity in the Trail Making Test: what makes Part B harder? *J Clin Exp Neuropsychol.* 1995;17(4):529-535.
- Lezak MD, Howieson DB, Loring DW. *Neuropsychological Assessment.* 4th ed. New York: Oxford University Press; 2004.
- Reitan RM. Validity of the Trail Making test as an indicator of organic brain damage. *Percept Mot Skills.* 1958;8:271-276.

Trail Making Test Part A

Patient's Name: ______________________ Date: ______________

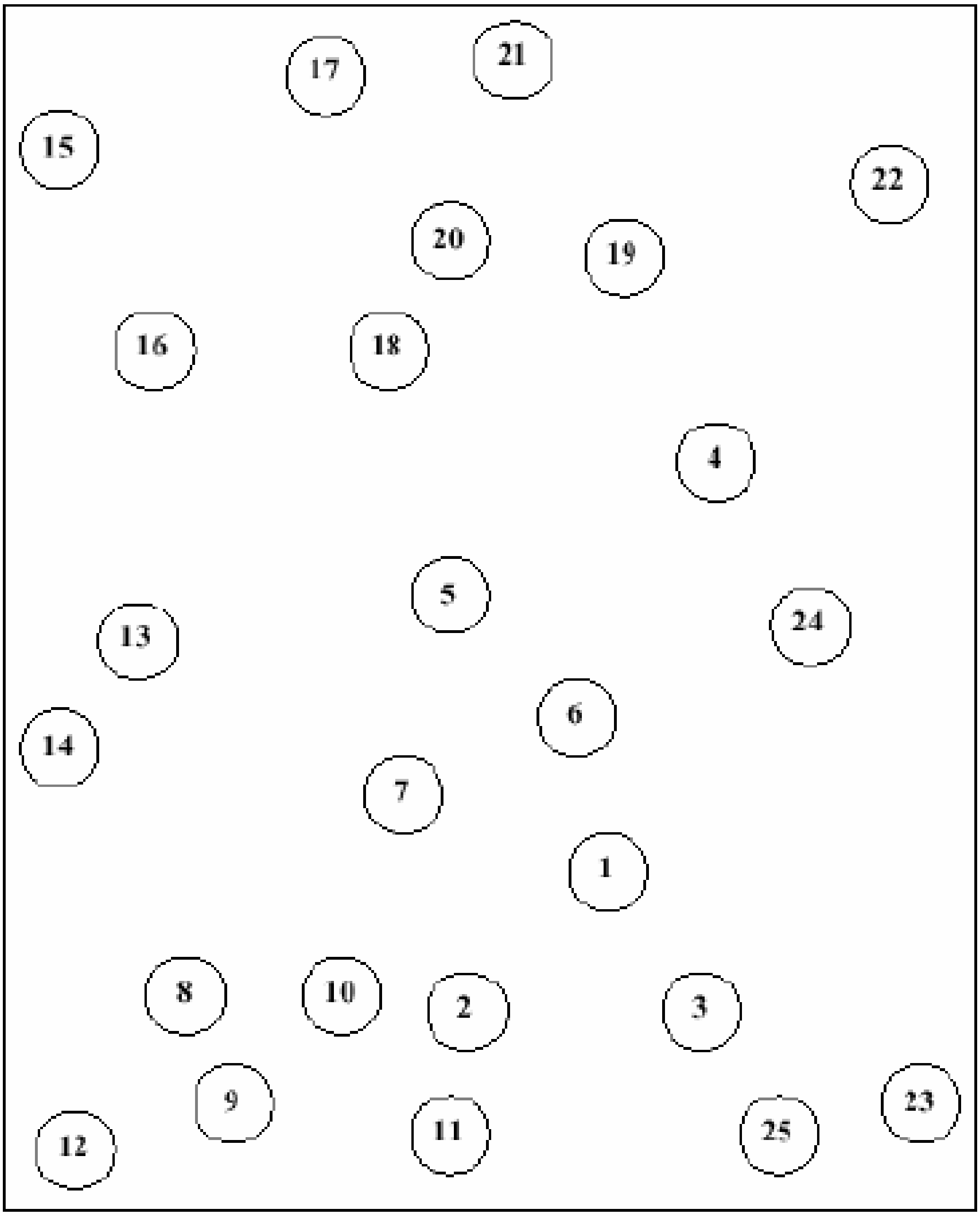

Trail Making Test Part A – *SAMPLE*

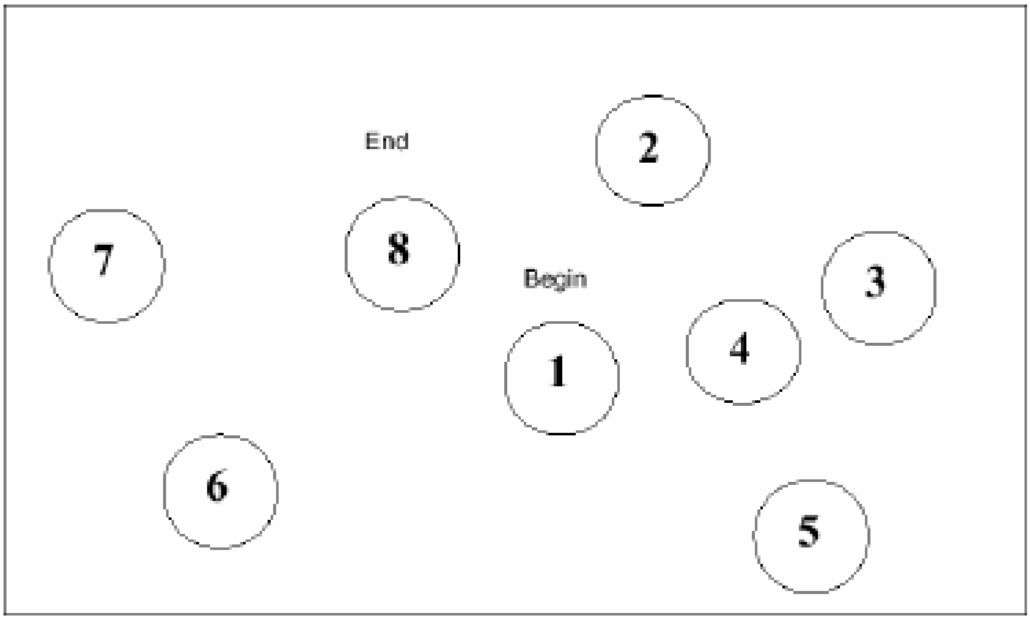

Trail Making Test Part B

Patient's Name: ______________________ Date: ________________

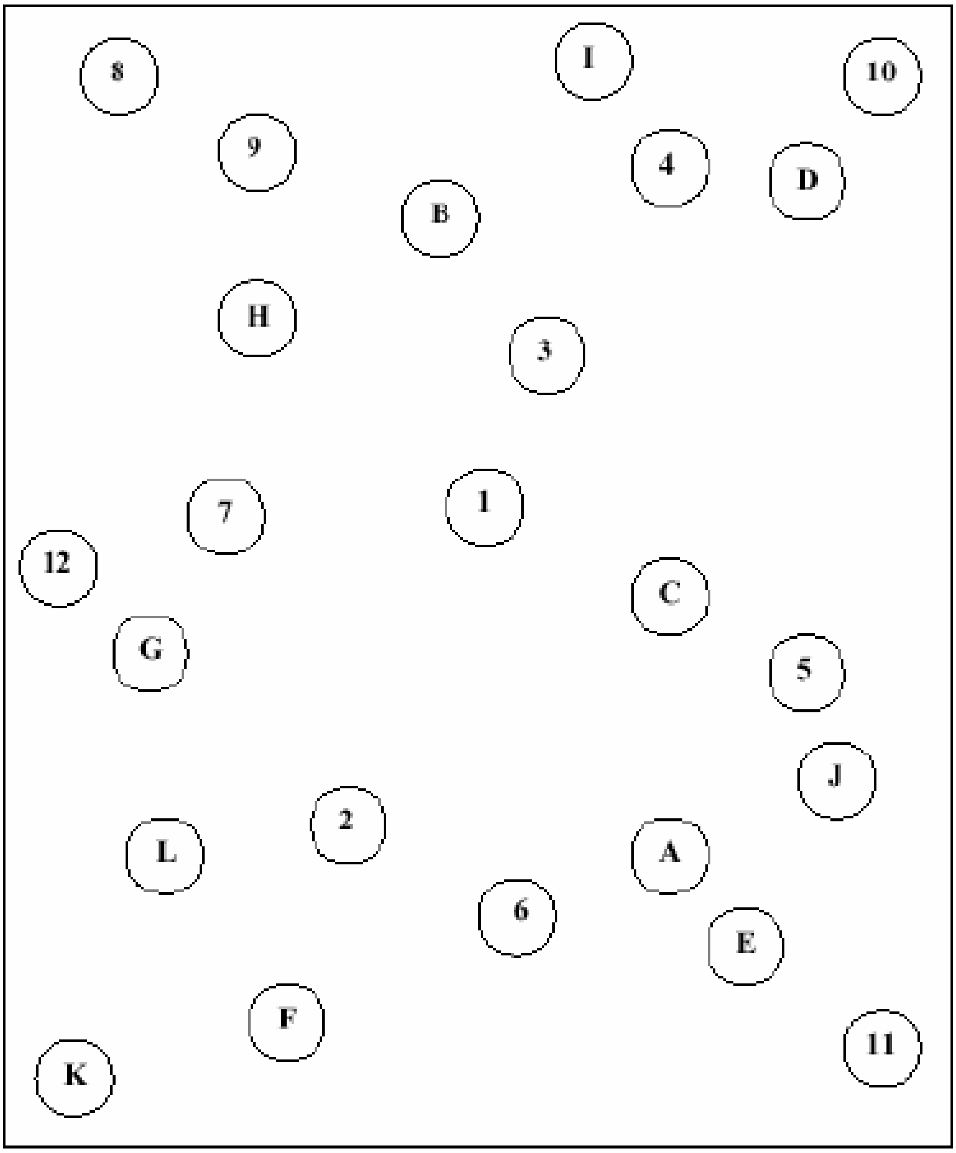

Trail Making Test Part B – *SAMPLE*

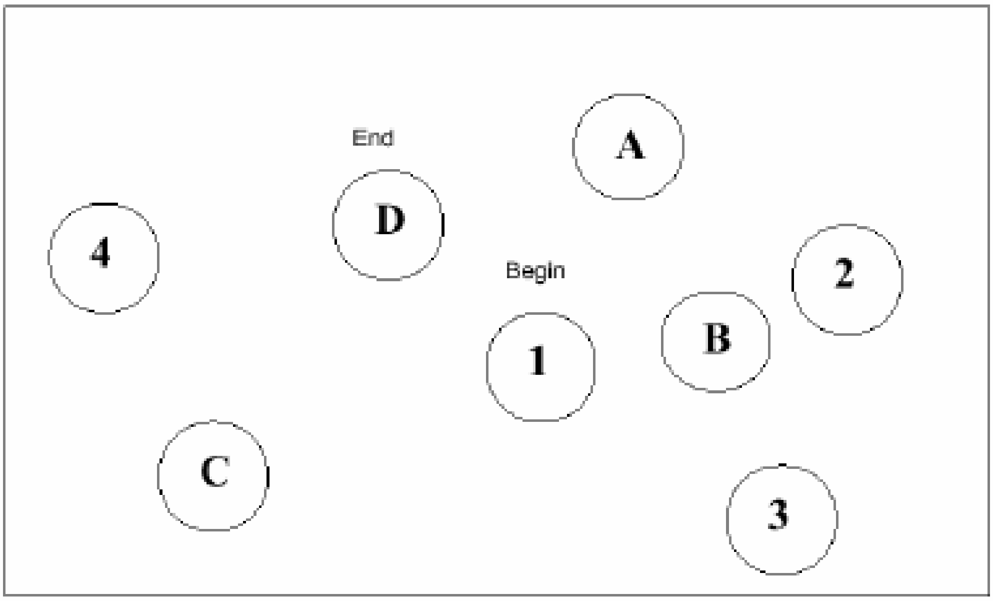

Appendix 9: Clinical Global Impression (CGI) Rating Scale

Clinical Global Impression (CGI)

***Reference:* Guy W, editor. ECDEU Assessment Manual for Psychopharmacology. 1976. Rockville, MD, U.S. Department of Health, Education, and Welfare**

Rating Clinician-rated

Administration time Varies with familiarity with patient

Main purpose To provide a global rating of illness severity, improvement and response to treatment

Population Adults

Commentary

Amongst the most widely used of extant brief assessment tools in psychiatry, the CGI is a 3-item observer-rated scale that measures illness severity (CGIS), global improvement or change (CGIC) and therapeutic response. The illness severity and improvement sections of the instrument are used more frequently than the therapeutic response section in both clinical and research settings. The Early Clinical Drug Evaluation Program (ECDEU) version of the CGI (reproduced here) is the most widely used format, and asks that the clinician rate the patient relative to their past experience with other patients with the same diagnosis, with or without collateral information. Several alternative versions of the CGI have been developed, however, such as the FDA Clinicians' Interview-Based Impression of Change (CIBIC), which uses only information collected during the interview, not collateral. The CGI has proved to be a robust measure of efficacy in many clinical drug trials, and is easy and quick to administer, provided that the clinician knows the patient well.

Scoring

The CGI is rated on a 7-point scale, with the severity of illness scale using a range of responses from 1 (normal) through to 7 (amongst the most severely ill patients). CGI-C scores range from 1 (very much improved) through to 7 (very much worse). Treatment response ratings should take account of both therapeutic efficacy and treatment-related adverse events and range from 0 (marked improvement and no side-effects) and 4 (unchanged or worse and side-effects outweigh the therapeutic effects). Each component of the CGI is rated separately; the instrument does not yield a global score.

Versions

CGI for bipolar disorder (CGI-BD), FDA Clinicians' Interview-Based Impression of Change (CIBIC), Clinicians' Interview-Based Impression of Change-Plus (CIBIC+), NYU CIBIC+, Parke-Davis Pharmaceuticals Clinical Interview-Based Impression (CIBI); the CGI has been translated into most languages.

Additional references

Leon AC, Shear MK, Klerman GL, Portera L, Rosenbaum JF, Goldenberg I. A comparison of symptom determinants of patient and clinician global ratings in patients with panic disorder and depression. J Clin Psychopharmacol 1993; 13(5):327–31.

Spearing MK, Post RM, Leverich GS, Brandt D, Nolen W. Modification of the Clinical Global Impressions (CGI) Scale for use in bipolar illness (BP): the CGI-BP. Psychiatry Res 1997; 73(3):159–71.

Zaider TI, Heimberg RG, Fresco DM, Schneier FR, Liebowitz MR. Evaluation of the clinical global impression scale among individuals with social anxiety disorder. Psychol Med 2003; 33(4):611–22.

Address for correspondence

Not applicable – the CGI is in the public domain.

Clinical Global Impression (CGI)

1. **Severity of illness**
   Considering your total clinical experience with this particular population, how mentally ill is the patient at this time?

0 = Not assessed	4 = Moderately ill
1 = Normal, not at all ill	5 = Markedly ill
2 = Borderline mentally ill	6 = Severely ill
3 = Mildly ill	7 = Among the most extremely ill patients

2. **Global improvement:** Rate total improvement whether or not, in your judgement, it is due entirely to drug treatment.
   Compared to his condition at admission to the project, how much has he changed?

0 = Not assessed	4 = No change
1 = Very much improved	5 = Minimally worse
2 = Much improved	6 = Much worse
3 = Minimally improved	7 = Very much worse

3. **Efficacy index:** Rate this item on the basis of **drug effect only.**
   Select the terms which best describe the degrees of therapeutic effect and side effects and record the number in the box where the two items intersect.
   EXAMPLE: Therapeutic effect is rated as 'Moderate' and side effects are judged 'Do not significantly interfere with patient's functioning'.

**Therapeutic effect**		**Side effects**			
		None	*Do not significantly interfere with patient's functioning*	*Significantly interferes with patient's functioning*	*Outweighs therapeutic effect*
**Marked**	Vast improvement. Complete or nearly complete remission of all symptoms	01	02	03	04
**Moderate**	Decided improvement. Partial remission of symptoms	05	06	07	08
**Minimal**	Slight improvement which doesn't alter status of care of patient	09	10	11	12
**Unchanged or worse**		13	14	15	16

Not assessed = 00

Reproduced from Guy W, editor. ECDEU Assessment Manual for Psychopharmacology. 1976. Rockville, MD, U.S. Department of Health, Education, and Welfare

Appendix 10: Patient's global opinion on the effect of investigational drugs

**PATIENT'S GLOBAL OPINION on the effect of the investigational drugs**	
Ask the patient to complete the questionnaire by comparing the treatment period referenced to the prestudy conditions.<br>Report hereunder the date and time of performance and the patient's answer.	
Date: |__|__|/|__|__|/|__|__| (DD/MM/YY)	Time: |__|__|h|__|__|min
❒ Marked effect<br>(complete or nearly complete remission of EDS)	❒ Moderate effect<br>(partial remission of EDS)
❒ Minimal effect<br>(slight decrease in EDS that does not substantially alter the status of the patient)	❒ No change
❒ Minimally worse<br>(slight increase in EDS)	❒ Much worse<br>(substantial increase in EDS)

Appendix 11: DSMB charter

DATA SAFETY MONITORING BOARD (DSMB)

CHARTER

Protocol P1513

Efficacy and Safety of Pitolisant (BF2.649) in the Treatment of Excessive Daytime Sleepiness in Patients with Obstructive Sleep Apnoea Syndrome, Treated or Not by Nasal Continuous Positive Airway Pressure, but Still Complaining of Excessive Daytime Sleepiness – Phase III.

According to ICH E9 an independent DSMB may be established by the sponsor to assess at intervals the progress of a clinical trial, safety data, and critical efficacy variables and recommend to the sponsor whether to continue, modify or terminate a trial. The DSMB should have written operating procedures and maintain records of all its meetings, including interim results; these should be available for review when the trial is complete. The independence of the DSMB is intended to control the sharing of important comparative information and to protect the integrity of the clinical trial from adverse impact resulting from access to trial information. The DSMB is a separate entity from an Institutional review Board (IRB) or an Independent Ethics Committee (IEC), and its composition should include clinical trial scientists knowledgeable in the appropriate disciplines including statistics.
When there are sponsor representatives on the DSMB, their role should be clearly defined in the operating procedures of the committee (for example, covering whether or not they can vote on key issues). Since these sponsor staff would have access to unblinded information, the procedures should also address the control of dissemination of interim trial results within the sponsor organization.

I. Roles and Responsibilities

The Data and Safety Monitoring Board (DSMB) is an independent group of experts that advises the study investigators. The members of the DSMB serve in an individual capacity and provide their expertise and recommendations.
The primary responsibilities of the DSMB are to :

1) Periodically review and evaluate the accumulated study data for participant safety, study conduct and progress, and, when appropriate, efficacy, and
2) Make recommendations concerning the continuation, modification, or termination of the trial. The DSMB considers study-specific data as well as relevant background knowledge about the disease, test agent, or patient population under study.
The DSMB is also responsible for maintaining the confidentiality of its internal discussions and activities as well as the contents of reports provided to it.
The DSMB should review each protocol for any major concern prior to implementation. During the trial, the DSMB should review cumulative study data to evaluate safety, study conduct, and scientific validity and integrity of the trial. As part of this responsibility, DSMB members must be satisfied that the timeliness, completeness, and accuracy of the data submitted to them for review are sufficient for evaluation of the safety and welfare of study participants. The DSMB should also assess the performance of overall study operations and any other relevant issues, as necessary.
Items reviewed by the DSMB include:

- Interim/cumulative data for evidence of study-related adverse events;
- Interim/cumulative data for evidence of efficacy according to pre-established statistical guidelines, if appropriate;
- Data quality, completeness, and timeliness;
- Performance of individual centers;
- Adequacy of compliance with goals for recruitment and retention, including those related to the participation of women and minorities;
- Adherence to the protocol;
- Factors that might affect the study outcome or compromise the confidentiality of the trial data (such as protocol violations, unmasking, etc.); and,
- Factors external to the study such as scientific or therapeutic developments that may impact participant safety or the ethics of the study.

The DSMB should conclude each review with their recommendations as to whether the study should continue without change, be modified, or terminated. Recommendations regarding modification of the design and conduct of the study could include:

- Modifications of the study protocol based upon the review of the safety data;
- Suspension or early termination of the study or of one or more study arms because of serious concerns about subjects' safety, inadequate performance or rate of enrollment;
- Suspension or early termination of the study or of one or more study arms because study objectives have been obtained according to pre-established statistical guidelines;
- Optional approaches for investigators to consider when the DSMB determines that the incidence of primary study outcomes is substantially less than expected such as

recommendations to increase the number of trial centers or extend the recruitment period; and,

- Corrective actions regarding a study center whose performance appears unsatisfactory or suspicious.

Confidentiality must always be maintained during all phases of DSMB review and deliberations. Usually, only voting members of the DSMB should have access to interim analyses of outcome data by treatment group. Exceptions may be made when the DSMB deems it appropriate. The reason and to whom the exceptions for access to interim analyses is granted will be documented in the Closed Session Report. DSMB members must maintain strict confidentiality concerning all privileged trial results ever provided to them. The DSMB should review data only by masked study group (such as X vs. Y rather than experimental vs. control) unless or until the DSMB determines that the identities of the groups are necessary for their decision-making. Whenever masked data are presented to the DSMB, the key to the group coding must be available for immediate unmasking.

II. Membership

The membership of the DSMB should reflect the disciplines and medical specialties necessary to interpret the data from the clinical trial and to fully evaluate participant safety. The number of DSMB members depends on the phase of the trial, range of medical issues, complexity in design and analysis, and potential level of risk but generally consists of three to seven members including, at a minimum:

- Expert(s) in the clinical aspects of the disease/patient population being studied;
- One or more biostatisticians; and,
- Investigators with expertise in current clinical trials conduct and methodology.

Ad hoc specialists may be invited to participate as non-voting members at any time if additional expertise is desired. Some trials, depending on the population and nature of the intervention, may well be served by inclusion of a bioethicist on the DSMB, Steering Committee, or Advisory Panel.

Conflict of Interest

No member of the DSMB should have direct involvement in the conduct of the study. Furthermore, no member should have financial, proprietary, professional, or other interests that may affect impartial, independent decision-making by the DSMB. Interests that may create a potential conflict of interest should be disclosed to the DSMB prior to any discussion. The DSMB will determine how to handle such potential conflict. The DSMB can require that a member with a potential conflict not vote or take other means deemed appropriate.

Selection and Invitation to Participate

Participation is generally for the duration of the study. Participation for standing DSMBs convened to monitor multiple protocols or lengthy studies may be for fixed terms.

III. Meetings

The frequency of DSMB meetings depends on several factors including the rate of enrollment, safety issues or unanticipated adverse events, availability of data, and, where relevant, scheduled interim analyses. The representative's Sponsor is responsible for convening meetings, selecting a venue when the meeting is not convened by teleconference, and coordinating the distribution of meeting materials to DSMB members and other meeting participants. The agenda for each meeting is generally developed jointly by the representative's Sponsor, the Principal Investigator (regardless of whether a contract, cooperative agreement, or grant), the study statistician, and DSMB Chair.

The initial DSMB meeting should occur preferably before the start of the trial or as soon thereafter as possible. At this meeting the DSMB should discuss the protocol and the DSMB charter which includes triggers set for data review or analyses, definition of a quorum, and guidelines for monitoring the study. Guidelines should also address stopping the study for safety concerns and, where relevant, for efficacy based on plans specified in the protocol. At this meeting, the DSMB should also develop procedures for conducting business (e.g., voting rules, attendance, etc.).

Once a study is implemented, the DSMB should convene as often as necessary, but at least once annually, to examine the accumulated safety and enrolment data, review study progress, and discuss other factors (internal or external to the study) that might impact continuation of the study as designed. A DSMB meeting may be requested by DSMB members, industrial collaborator, IRB, or study Principal Investigator at any time to discuss safety concerns. In the event a DSMB member cannot attend a meeting, he/she may receive a copy of the closed session DSMB report (see below) and either participate by conference call or provide written comments to the DSMB Chair for consideration at the meeting.

DSMB Meeting Format

The recommended meeting format consists of Open Sessions, Closed Session, and Closed Executive Session.

Open Session: Open session reports generally include administrative reports by site that describe participants screened, enrolled, completed, and discontinued, as well as baseline characteristics of the study population. Other general information on study status may also be presented. Listings of adverse events and serious adverse events, but none of the data should be presented in an unblinded manner.

Issues relating to the general conduct and progress of the study are discussed including adverse events and toxicity issues, accrual, demographic characteristics of enrollees, disease

status of enrollees (if relevant), comparability of groups with respect to baseline factors, protocol compliance, site performance, quality control, and timeliness and completeness of follow-up. Outcome results must not be discussed during this session. DSMB members, voting and invited *ex officio* members, ad hoc experts attend this session. The lead investigator and the study biostatistician should be in attendance in order to present results and respond to questions. This session is open to representatives for industrial collaborators, study investigators.

Closed Session: Grouped safety data_and, if appropriate, efficacy data are presented by the study statistician(s) at this session. Grouped data should be presented by coded treatment arm. This session is normally attended only by voting members, and invited *ex officio* members. The DSMB may invite the participation of other individuals for all or part of the session.

Closed Executive Session: This final session involves only DSMB voting members to ensure complete objectivity as they discuss outcome results, make decisions, and formulate recommendations regarding the study. If treatment codes have been made accessible to the DSMB, then the DSMB may unmask the data based on procedures identified in advance.

Voting

A quorum, as defined by the DSMB in the initial meeting, must be present either in person or by conference call. After a thorough discussion of DSMB members' opinions and rationale and an attempt to reach clarity regarding individual recommendations, the final recommendations of each DSMB member should be solicited in Closed Executive Session (*ex officio* members shall not vote and shall not be present at this voting session).). A consensus opinion or recommendation among members is not required; each member may have individual opinions. The final recommendations are recorded and either identified as majority or minority positions or are accompanied by actual vote tallies for each divergent recommendation, i.e., as number of votes for or against a particular action, such as continuing or terminating a study, etc.

IV. Study Reports for DSMB Meetings

Summary safety and enrollment data should be forwarded periodically to the DSMB. The DSMB should receive all protocol revisions and may receive other documents relating to the study including Clinical Investigator's Brochure (CIB) and all CIB revision.

Reports are prepared by the study statistician(s). The study statistician should provide suggested formats or templates for data presentation for the initial meeting of the DSMB. At subsequent meetings, additions or modifications to these reports may be directed by the DSMB on a one-time or continuing basis. Written reports should be sent to DSMB members prior to the meeting and should allow sufficient time for review.

Reports for meetings of the DSMB consist in the data presented in the reports reflecting both the need for the fullest possible information on trial results and the need to assure reliability and accuracy of the information included.

This report may contain data on study outcomes, including safety data and, depending on the study, efficacy data coded by group. Interim analyses of efficacy data are presented only when planned in advance and appropriate statistical criteria for assessing evidence of efficacy have been clearly addressed. Supplemental information may need to be furnished immediately after the meeting if the DSMB decides that such follow-up is needed in order to conclude their deliberations.

The Closed Session Report is confidential and marked accordingly. Copies of reports distributed prior to and during a meeting are collected by the study statistician(s) at the end of the Closed Session. Procedures for securing closed reports distributed to telephone and videoconference participants should be specified in advance of the meeting.

V. Other Reports of Study Progress

Masked safety and enrollment data may be forwarded periodically to all DSMB members or to the member who serves as the Independent Safety Monitor. The DSMB receives all protocol revisions and may receive other documents relating to the study, such as annual reports, manuscripts, and newsletters.

VI. Reports from the DSMB

Summary Report: The DSMB will issue a written summary report that identifies topics discussed by the DSMB and describes their individual findings, overall safety assessment and recommendations. The rationale for recommendations will be included when appropriate. This report will generally not include confidential information. The DSMB Chair or designee is responsible for drafting, circulating and obtaining approval from other DSMB members within two (2) weeks of the meeting. The final summary report will be forwarded through a designated study team representative (usually the Principal Investigator) and to other appropriate staff. The study team representative is responsible for disseminating the DSMB summary report to site investigators who must, in turn, submit the report to their local IRBs.

Closed Session Report: (optional): The DSMB may also prepare confidential minutes that include details of closed session discussions. Meeting minutes are to be held in strict confidence, accessible only to voting members of the DSMB until such time when the study is closed or the DSMB recommends early termination or in the event the minutes are requested by the Competent Authorities for participant safety reasons or for regulatory purposes.

Immediate Action Report: The DSMB Chair will notify the representative's Sponsor of any findings of a serious and immediate nature or recommendations to discontinue all or part of the trial. Recommendations to discontinue or substantially modify the design or conduct of a study must be conveyed in writing by e-mail, fax, or courier on the day of the DSMB meeting. This written, confidential report may include the DSMB member's rationale for their recommendations.

VII. Relationship between DSMBs and IRBs

The DSMB should provide feedback at regular and defined intervals to the IRBs. A brief summary report should be sent to each investigator after each meeting. The report should document that a review of data and outcomes across all centers took place on a given date. It should summarize the DSMB members' review of the cumulative toxicities reported from all participating sites without specific disclosure by treatment arm. It should also inform study investigators of the DSMB members' conclusions with respect to progress or need for modification of the protocol. The investigator is required to transmit the report to his/her local IRB.

VIII. Trial Description and Study Design

- Trial name: P1513
- Trial sponsor: bioprojet
- Study drug tested: pitolisant (BF2.649)
- Trial design: Double blind placebo controlled parallel groups (12 weeks study treatment) with 39 weeks Open Label Extension period
- Phase: phase III
- Number of expected patients: 180
- Study sites: several sites in Bulgaria

IX. DSMB Description

- This DSMB will be coordinated by bioprojet 's representative
- This DSMB will be independent of bioprojet, regulatory agencies, IRB/EC, and investigators.
- This charter will be approved by its DSMB members as attested to by signature of the chairperson.

DSMB members

The DSMB members have been selected by the sponsor.

Appendix 12: Instructions for Filling In Bioprojet SAE Report Form

General instructions

The form must be completed in English. Please use capital letters and black pen to increase legibility.

Avoid using abbreviations as these may differ across centres and countries. If abbreviations are used in the narrative section, they should be explained in the text. Use medical terminology and be clear, concise and unambiguous.

Patient details and history

Enter patient number (patient number, screening number and/or randomisation number).

If the event occurs prior to patient number allocation, please enter inevitably the screening number.

Resume the relevant patient medical history.

Seriousness criterion

Please indicate the reason why the adverse event is serious (tick all that apply) in accordance with the following criteria:

- results in death,
- is immediately life threatening
- requires in-patient hospitalisation or prolongation of existing hospitalisation
- results in persistent or significant disability or incapacity
- is a congenital abnormality/birth defect
- is an important medical event that may jeopardise the patient or may require medical intervention to prevent one to the outcomes listed above.

Please note the following:

- ***Life-threatening*** refers to an event in which the patient was at risk of death at the time of the event; it does not refer to an event which hypothetically might have caused death if it were more severe.
- ***Hospitalisation*** describes a period of at least 24 hours. Over-night stay for observation, stay at emergency room or treatment on an out-patient basis do not constitute a hospitalisation. However, medical judgement must always be exercised and when in doubt the case should be considered as serious (i.e. if case fulfils the criterion for a medically important event). Hospitalisation for administrative or social purpose does not constitute a serious adverse event. Hospital admissions and/or surgical operation planned before study inclusion are not considered adverse event if the illness or disease existed before the patient was enrolled in the study, provided that the condition did not deteriorate during the study.
- ***Disability/Incapacity*** means a substantial disruption of a person's ability to conduct normal

life functions. In doubt, the decision should be left to medical judgement by the investigator.
- ***Important medical events*** are events that may not be immediately life-threatening, or result in death or hospitalisation but may jeopardise the patient or may require intervention to prevent one of the other outcomes listed in the definitions above. Examples of important medical events include events that suggest a significant hazard, contraindication or precaution, occurrence of malignancy or development of drug dependency or drug abuse. Medical and scientific judgement should be exercised in deciding whether events qualify as medically important.
An adverse event caused by an *overdose* is considered serious if a criterion listed in the definition above is fulfilled.

Serious Adverse Event

Enter the main adverse event as a diagnosis if available. If not, enter the separate sign(s) and symptom(s) which resulted in this report. Write **one** diagnosis/symptom per line. Please note that the AE verbatim on the SAE Form must match the AE verbatim on the Adverse Event Form.
- Note that death is not an event but the cause of death is the event
- Note that procedures should be captured along with the reason for conducting the procedures. The reason (e.g. appendicitis) should be entered as the main event and the procedure (e.g. appendicectomy) should be entered as a treatment.
- Note that pre-existing conditions, elective surgery and decreases in intensity are not adverse events.

Enter *onset* date. Onset date is the date when the first sign(s) or symptom(s) were noted (e.g. if the patient was hospitalised for meningitis, and symptoms such as fever, headache, nausea started before the date of hospitalisation, the onset date should be the day before hospitalisation when the first symptoms started).
If the adverse event is an abnormal laboratory test (such as "platelets low"), enter onset date as the date of the analysis.
Enter *stop date*, if applicable. If the event is not resolved enter "NA" (not applicable). *Duration* less than 24 hours should be entered in hours (or minutes if applicable).
Enter *intensity*: according to following definition:
Mild: Awareness of signs and symptoms but no disruption of usual activity. Symptoms do not require therapy or a medical evaluation; signs and symptoms are transient.
Moderate: Event sufficient to affect usual activity (disturbing). Are usually improved by simple therapeutic measures.
Severe: Inability to work or perform usual activities (unacceptable). Generally require systemic drug therapy or other treatment.
Enter *outcome* (died, recovered, recovered with sequelae, not yet recovered). If the patient recovered with sequelae, specify so in the narrative field. If the outcome is "not yet recovered",

remember to follow-up on the outcome.

Study drug / Suspected drugs

Enter the study drug (with dose taken at the time of the event) as main suspect drug. If other drugs than the study drug is suspected to have caused the event, please add these drugs as additional suspected drugs.

If the study is blinded, enter "code not broken" and the different therapeutic options e.g. Drug X/Placebo.

Enter the *treatment number*.

Enter *dose/unit/frequency/route*. If treatment differs from the protocol, please specify the difference in the narrative.

Enter date for treatment initiation, and if applicable date for discontinuation. If therapy is ongoing, enter "on going".

Enter date of last dose prior event: if less than 24 hours, precise the duration in hours (or minutes if applicable).

Enter the investigator's assessment of the *causal relationship* between the event and the investigational drug(s) according to following definition:

Related / likely: Clearly related to the investigational agent / procedure, i.e. an event that follows a reasonable temporal sequence from administration of the study intervention, follows a known or expected response pattern to the suspected intervention, that can be confirmed by improvement on stopping and reappearance of the event after rechallenge and that could not be reasonably explained by the known characteristics of the subject's clinical state.

Possibly related / Possible: Follows a reasonable temporal sequence from administration of the study intervention, follows a known or expected response pattern to the suspected intervention, but that could readily have been produced by a number of other factors.

Not related / Unlikely: Clearly and incontrovertibly due only to extraneous causes, and does not meet criteria listed under possible (possibly related) or likely (related).

Narrative and documents

Describe the cause of event(s) as accurately and thoroughly as possible. The scope of the narrative is to give a full overview of the cause of event(s), making the case understandable to people without existing knowledge of the patient/subject and without access to the patient's/subject's chart.

The narrative should include information on signs and symptoms, relevant tests and outcome.

If the patient was hospitalized, resume the relevant information of the hospitalization report.

Remember that information on adverse effects are collected in order to establish the safety profile of the drug and that the information collected will be included in the labelling. It is therefore imperative that the information is of high data quality/consistency as the labelling

should reflect the safety profile of the drug accurately and adequately.

Follow-up notification

If you have any further relevant information after the initial notification, please complete the complementary section immediately and send it like the initial notification.

Enter *patient identification number* (patient number or screening number or randomisation number).

Appendix 13: Beck Depression Inventory – 13 Items (BDI-13)

Short form of the Beck Depression Inventory

Instructions

This is a questionnaire. On the questionnaire are groups of statements. Please read the entire group of statements in each category. Then tick out the one statement in that group which best describes the way you feel today, that is, *right now!* Circle the number beside the statement you have chosen. If several statements in the group seem to apply equally well, circle each one.

A. (Sadness)
- 0 - I do not feel sad
- 1 - I feel sad or blue
- 2 - I am blue or sad all the time and I can't snap out of it
- 3 - I am so sad or unhappy that I can't stand it

B. (Pessimism)
- 0 - I am not particularly pessimistic or discouraged about the future
- 1 - I feel discouraged about the future
- 2 - I feel I have nothing to look forward to
- 3 - I feel that the future is hopeless and that things cannot improve

C. (Sense of Failure)
- 0 - I do not feel like a failure
- 1 - I feel I have failed more than the average person
- 2 - As I look back on my life, all I can see is a lot of failures
- 3 - I feel I am a complete failure as a person (parent, husband, wife)

D. (Dissatisfaction)
- 0 - I am not particularly dissatisfied
- 1 - I don't enjoy things the way I used to
- 2 - I don't get satisfaction out of anything anymore
- 3 - I am dissatisfied with everything

E. (Guilt)
- 0 - I don't feel particularly guilty
- 1 - I feel bad or unworthy a good part of the time
- 2 - I feel quite guilty
- 3 - I feel as though I am very bad or worthless

F. (Self-Dislike)
- 0 - I don't feel disappointed in myself
- 1 - I am disappointed in myself
- 2 - I am disgusted with myself
- 3 - I hate myself

G. (Self-Harm)
0 - I don't have any thoughts of harming myself
1 - I feel I would be better off dead
2 - I have definite plans about committing suicide
3 - I would like kill myself if I had the chance

H. (Social Withdrawal)
0 - I have not lost interest in other people
1 - I am less interested in other people than I used to be
2 - I have lost most of my interest in other people and have little feeling for them
3 - I have lost all of my interest in other people and don't care about them at all

I. (Indecisiveness)
0 - I make decisions about as well as ever
1 - I try to put off making decisions
2 - I have great difficulty in making decisions
3 - I can't make any decisions at all any time

J. (Self-Image Change)
0 I don't feel I look any worse than I used to
1 I am worried than I am looking old or unattractive
2 I feel that there are permanent changes in my appearance and they make me look unattractive
3 I feel that I am ugly or repulsive looking

K. (Work Difficulty)
0 - I can work about as well as before
1 - It takes extra effort to get started at doing something
2 - I have to push myself very hard to do anything
3 - I can't do any work at all

L. (Irritability)
0 - I am no more irritated by things than I ever am
1 - I am slightly more irritated now than usual.
2 - I am quite annoyed or irritated a good deal of the time.
3 - I feel irritated all the time now.

M. (Anorexia)
0 - My appetite is no worse than usual
1 - My appetite is not as good as it used to be
2 - My appetite is much worse now
3 - I have no appetite at all any more

Scoring:

0 – 4 : none or minimal depression
4 – 7 : mild depression
8 – 15: moderate depression
≥ 16 : severe depression

Appendix 14: Patient's overall evaluation of the tolerance

PATIENT'S OVERALL EVALUATION OF THE TOLERANCE
❒ Good<br>❒ Moderate<br>❒ Poor

Appendix 15: Amphetamine-Like Withdrawal Symptoms Questionnaire (DSM IV)

**AMPHETAMINE LIKE WITHDRAWAL SYMPTOMS**		
Dysphoria	❒ yes	❒ no
Fatigue	❒ yes	❒ no
Vivid and unpleasant dreams	❒ yes	❒ no
Insomnia or hypersomnia	❒ yes	❒ no
Increased appetite	❒ yes	❒ no
Psychomotor retardation or agitation	❒ yes	❒ no

Appendix 16: Mini Mental State Examination Questionnaire (MMSE)

The Mini mental state examination (MMSE) is a brief 30-point questionnaire test that is used to screen for cognitive impairment. In the time span about 10 minutes it samples various functions including arithmetic, memory and orientation. Any score over 28 (out of 30) is effectively normal. Below this, 20-26 indicates some cognitive impairment; 10-19 moderate to severe cognitive impairment, and below 10 very severe cognitive impairment. The MMSE will be performed at V1. The patient will not be included in the study if a score of MMSE<28.

MMSE (1 / 2)	
**1. Orientation** *point for each correct answer (maximum 10 points)*	
1. What is the year?	I__I
2. What is the season?	I__I
3. What is the month?	I__I
4. What is the date?	I__I
5. What is the day?	I__I
6. Where are we? (country)	I__I
7. Where are we? (county)	I__I
8. Where are we? (town)	I__I
9. Where are we? (which hospital)	I__I
10. Where are we? (which floor)	I__I
**2. Registration** Name three common objects (e.g. "apple", "table" and "penny"). Take one second to say each one. Then ask the patient to repeat all three after you have said them. Give one point for each correct answer. Repeat the object names until all three are learned (up to 6 trials) *(maximum 3 points)*	
11. "Apple"	I__I
12. "Table"	I__I
13. "Penny"	I__I
**3. Attention and Calculation**	
14. Spell "world" backwards. Give one point for each letter that is in the right place. (D_L_R_O_W). *(maximum 5 points)*	I__I
Alternatively, do serial 7s: Ask the person to count backwards from 100 in blocks of 7. Stop after 5 subtractions. Give one point for each correct answer. If one answer is incorrect (e.g. 92) but the following answer is 7 less than the previous answer (i.e.), count the second answer as correct. *(maximum 5 points)*	
14. « 93 »	I__I
15. « 86 »	I__I
16. « 79 »	I__I
17. « 72 »	I__I
18. « 65 »	I__I

MMSE (2 / 2)	
**4. Recall** Ask for the 3 objects repeated above (e.g., apple, table and penny). Give 1 point for each correct object *(maximum 3 points).*	
19. "Apple"	I__I
20. "Table"	I__I
21. "Penny"	I__I
**5. Language**	
22. Point to a pencil and ask the person to name this object (1 point)	I__I
23. Do the same thing with a wrist-watch (1 point)	I__I
24. Ask the patient to repeat the following sentence: "No ifs, ands or buts". Allow only one trial (1 point)	I__I
Give the patient a piece of blank white paper and ask them to follow a 3 stage command (1 point for each command correctly followed) :	
25. "Take a paper in your right hand'	I__I
26. "Fold it in half"	I__I
27. " Put it on the floor"	I__I
28. Write "CLOSE YOUR EYES" in large letter and show it to the patient. Ask him or her to read the message and do what it says (give 1 point if they actually close their eyes)	I__I
29. Ask the patient to write a sentence of their choice on a blank of paper. The sentence must contain a subject and a verb and must make sense. Spelling, punctuation and grammar are not important (1 point)	I__I
30. Ask the patient to copy the design exactly as it is (1 point) 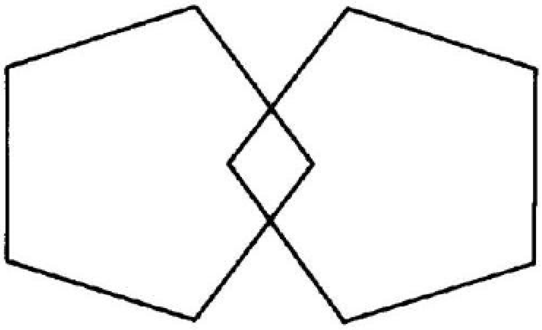	I__I
Total Score	I__I__I

Efficacy and Safety of Pitolisant (BF2.649) in the Treatment of Excessive Daytime Sleepiness in Patients with Obstructive Sleep Apnoea Syndrome, Treated or Not by Nasal Continuous Positive Airway Pressure, but Still Complaining of Excessive Daytime Sleepiness

Amended Protocol, version 2.0, dated 01 February 2018

Sponsor
Bioprojet
9, Rue Rameau
75002 Paris, France
Tel.: +33 1 47 03 66 33 - Fax: +33 1 47 03 66 30

Medical Project Manager
[redacted]
Bioprojet
9, Rue Rameau
75002 Paris, France
[redacted]

International Coordinator
[redacted]
University Multiprofile Hospital for Active Treatment
(UMHAT) "Aleksandrovska" EAD,
Clinic of Propedeutics of Internal Diseases
1, Sv. Georgi Sofiyski Str.
1432 Sofia, Bulgaria
[redacted]

Study Managing CRO
BalkanTrials Ltd.
8-B, Saint Kliment Ohridski Street
1504 Sofia, Bulgaria

TABLE OF CONTENTS

1. SYNOPSIS ... 9

2. STUDY DIAGRAM ... 17

3. INTRODUCTION ... 23

3.1. *Summary of relevant non clinical studies* ... 24

3.1.1. Pharmacological Profile ... 24

3.1.2. Safety profile ... 25

3.1.3. Pharmacology of pitolisant in potential therapeutic indication ... 26

3.1.3.1. Effects on Sleep/wakefulness and EEG patterns in cats and mice ... 26

3.1.3.2. Effects on Learning ... 27

3.1.3.3. Effects on Attention Deficit/ Hyperactivity Disorder (ADHD) ... 28

3.1.3.4. Effects on Epilepsy Disorders ... 28

3.1.3.5. Effects on psychotic diseases ... 28

3.1.4. Toxicology ... 29

3.1.4.1. Summary ... 29

3.1.5. Conclusion ... 34

3.2. *Summary of relevant clinical studies* ... 34

3.2.1. Phase I studies ... 34

3.2.2. Phase II Studies ... 36

3.2.2.1. Brief Summary ... 36

3.2.2.2. Two pilot studies referring to the Sleep Apnea Syndrome ... 38

3.2.2.3. Minimum effective dose-finding study of BF2.649, in patients with moderate to severe Obstructive Sleep Apnea, experiencing Excessive Daytime Sleepiness (EDS) despite regular use of nCPAP, and patients having refused this therapy. Randomized, double blind study with BF2.649 (5-, 10-, 20-, 40- mg/d), or placebo (P09-16 BF2.649) ... 42

3.2.3. Phase III studies ... 43

3.2.3.1. P09-08 Harosa 1 preliminary results ... 45

3.2.3.2. P09-09 Harosa 2 preliminary results ... 45

3.2.4. Summary of known and potential benefits and risks ... 46

4. RATIONALE OF THE CURRENT STUDY ... 47

5. STUDY OBJECTIVES ... 50

6. POPULATION ... 51

6.1. *Inclusion Criteria* ... 51

6.2. *Non-Inclusion Criteria* ... 52

6.3. *Patient completion and early withdrawal of patients from study* ... 53

7. EXPERIMENTAL DESIGN ... 54

8. STUDY SITES ... 59

9. INVESTIGATIONAL PRODUCTS ... 59
9.1. Chemical structure of compounds ... 59
9.2. Composition of investigational products ... 60
9.2.1. Composition of 5 mg and 20 mg pitolisant tablet ... 60
9.2.2. Composition of 5 mg and 20 mg placebo tablets ... 60
9.2.3. Administration of study treatment ... 60
9.2.4. Treatment Compliance ... 61
9.2.5. Packaging and labelling of the treatment boxes ... 61
9.2.6. Treatment quantity ... 63
9.2.7. Management and storage of the therapeutic units ... 63
9.2.8. Dispensation Modalities ... 64
9.2.9. Pharmacological Forbidden Concomitant Treatments and surgical interventions ... 64
9.2.10. Randomization of treatments ... 65
9.2.10.1. Patient Study number ... 65
9.2.10.2. Investigational treatment allocation ... 66
9.2.10.3. Unblinding envelopes ... 66
10. EVALUATION CRITERIA ... 67
10.1. Efficacy criteria ... 67
10.1.1. Primary endpoint: Epworth sleepiness scale (ESS) ... 67
10.1.2. Secondary endpoints ... 68
10.1.2.1. Percentage of ESS responders ... 68
10.1.2.2. Reduction of sleepiness and sleep episodes on the sleep diary ... 68
10.1.2.3. Improvement in vigilance according to Oxford Sleep Resistance (OSleR) test ... 69
10.1.2.4. Increase in quality of life ... 69
10.1.2.5. Improvement in cognitive function ... 71
10.1.2.6. Improvement in Clinical Global Impression (CGI) ... 72
10.1.2.7. Patient's global opinion on the effect of investigational drugs ... 73
10.1.2.8. Aggregate Z-score of secondary endpoints ... 73
10.2. Safety criteria ... 73
10.2.1. Period of observation ... 74
10.2.2. Adverse Event (AE) ... 74
10.2.3. Treatment-Emergent and Baseline-Emergent Adverse Events ... 74
10.2.4. Adverse Drug Reaction (ADR) ... 75
10.2.5. Unexpected Adverse Drug Reaction ... 75
10.2.6. Serious Adverse Event (SAE) or Serious Adverse Drug Reaction (Serious ADR) ... 75
10.2.7. Suspected Unexpected Serious Adverse Reaction (SUSAR) ... 76
10.2.8. Clarification of the difference in meaning between "severe" and "serious" ... 76
10.2.9. Analysis of adverse events ... 76
10.2.9.1. Assessment of intensity ... 76

10.2.9.2. Assessment of Causality 77
10.2.10. Reporting Adverse Event to the Sponsor (Appendix 12) 77
10.2.11. Reporting Serious Adverse Event to the sponsor 78
10.2.12. Reasons for expedited reporting 79
10.2.13. Adverse Events Outcome 79
10.2.14. Safety Endpoints 80
10.2.14.1. Adverse events 80
10.2.14.2. Physical examination 80
10.2.14.3. Vital signs 80
10.2.14.4. Electrocardiogram (ECG) 80
10.2.14.5. Beck Depression Inventory (the reduced version with 13 items) 80
10.2.14.6. Patient's overall evaluation of the tolerance 81
10.2.14.7. Amphetamine-like withdrawal symptoms questionnaire (DSM IV) 81
10.2.14.8. Laboratory tests 81
10.2.14.9. Pharmacokinetic assessments 83
10.2.14.10. Overdosing 85
10.2.14.11. Observance of nightly nCPAP use 85
11. *EMERGENCY PROCEDURES* 85
11.1. *Sponsor contact* 85
11.2. *Emergency identification of Study Products* 86
12. *CONDUCT OF THE TRIAL* 86
12.1. *Part I - Double Blind period* 87
12.1.1. V1 – Screening visit and beginning of initial wash-out period (D -14) 87
12.1.2. Ph1 – Phone contact (D -7) 89
12.1.3. V2 – Inclusion visit and beginning of the Double Blind period: baseline examinations, randomisation, start of escalating dose phase (D 0) 89
12.1.4. V3 – First dose adjustment visit (D 14) 90
12.1.5. V4 – Second dose adjustment visit and beginning of stable dose phase (D 21) 91
12.1.6. V5 – Control visit: continuation of stable dose phase (D 49) 92
12.1.7. V6 – Evaluation visit at the end of the Double Blind period and start of single-blindwash-out period (D 84) 93
12.1.8. Ph2 – Phone contact (V6 + 3D) 94
12.2. *Part II - Open Label Extension period* 94
12.2.1. V7 – End of study visit for patients who are not entering Open Label Extension period or Beginning of the Open Label Extension period for patients who are entering Open Label Extension period: start of new escalating dose phase (D 91) 94
12.2.2. V8-V9 – Dose adjustment visits (D 105 and D 112) 96
12.2.3. V10-V11 – Confirmed dose visits (D 196 and D 280) 97
12.2.4. V12 – Evaluation visit at the end of the Open Label Extension period and start of wash-out period

(D 364) 98
12.2.5. Ph3 – Phone contact (V12 + 3D) 99
12.2.6. V13 – End of study visit for patients who entered into Open Label Extension period (D 371) 99
13. *STATISTICAL ANALYSIS 100*
13.1. *Summary 100*
13.2. *Sample Size Determination and Justification 101*
13.3. *Primary and Secondary Populations 101*
13.4. *Handling of Missing Data 101*
13.5. *Futility Analysis 102*
13.6. *Statistical Analysis 102*
13.7. *Pharmacokinetic Analysis 103*
14. *DATA MANAGEMENT 104*
14.1. *Collection of data 104*
14.2. *Archiving of data, Audit 105*
14.3. *Data Protection and Confidentiality 106*
15. *QUALITY ASSURANCE 107*
15.1. *Good clinical Practice 107*
15.2. *Premature closure of the study 108*
15.2.1. Criteria for terminating the trial 108
15.2.2. Criteria for terminating an investigational site 108
15.3. *Control – Quality 109*
16. *REPORTING AND PUBLICATION OF RESULTS 109*
17. *CONTRACT, LIABILITY AND INSURANCE 109*
18. *FINANCIAL DISCLOSURE 110*
19. *CALENDAR FORECAST 110*
20. *REFERENCES 110*
21. *APPENDIX SECTION 112*
Appendix 1: Declaration of Helsinki 113
Appendix 2: Full-Night Polysomnography 119
Appendix 3: Epworth Sleepiness Scale (ESS) 120
Appendix 4: Sleep Diary 121
Appendix 5: European Quality of Life Questionnaire (EQ-5D) 122
Appendix 6: Leeds Sleep Evaluation Questionnaire (LSEQ) 124
Appendix 7: Pichot Fatigue Scale 125
Appendix 8: Trail Making Test (TMT) Parts A & B 126
Appendix 9: Clinical Global Impression (CGI) Rating Scale 131
Appendix 10: Patient's global opinion on the effect of investigational drugs 133
Appendix 11: DSMB charter 134
Appendix 12: Instructions for Filling In Bioprojet SAE Report Form 141

Appendix 13: Beck Depression Inventory – 13 Items (BDI-13)....................145
Appendix 14: Patient's overall evaluation of the tolerance....................147
Appendix 15: Amphetamine-Like Withdrawal Symptoms Questionnaire (DSM IV)....................148
Appendix 16: Mini Mental State Examination Questionnaire (MMSE)....................149

Signature's Page

The International Coordinator
Medical expert

[redacted]

Date: 20.02.2018

[redacted]

The Sponsor
Bioprojet

[redacted]

Date: 01.02.2018

Signature: [redacted]

Medical Project Manager
Bioprojet

[redacted]

Date: 01 FEB 2018

Signature: [redacted]

Investigator Signature's Page

PROTOCOL P1513 / BF2.649

STUDY "HAROSA III"

EFFICACY AND SAFETY OF PITOLISANT (BF2.649) IN THE TREATMENT OF EXCESSIVE DAYTIME SLEEPINESS IN PATIENTS WITH OBSTRUCTIVE SLEEP APNOEA SYNDROME, TREATED OR NOT BY NASAL CONTINUOUS POSITIVE AIRWAY PRESSURE, BUT STILL COMPLAINING OF EXCESSIVE DAYTIME SLEEPINESS

The signature below constitutes the approval of this P1513 / BF2.649 – HAROSA III protocol and the attachments, and provides the necessary assurance that this trial will be conducted in accordance with all stipulations of the protocol, including all statements regarding confidentiality, and in accordance with local legal and GCP regulatory requirements, and ICH guidelines.

The Investigator site:

Name: ………………………………………………………………………

Hospital: …………………………………………………………………

Address: …………………………………………………………………

Date: ___________________ Signature: ______________

1. SYNOPSIS

**TITLE**	Efficacy and Safety of Pitolisant (BF2.649) in the Treatment of Excessive Daytime Sleepiness in Patients with Obstructive Sleep Apnoea Syndrome, Treated or Not by Nasal Continuous Positive Airway Pressure, but Still Complaining of Excessive Daytime Sleepiness – Phase III.
**INTERNATIONAL COORDINATOR AND INVESTIGATORS**	PPDPPDPPD – UMHAT "Alexandrovska" – 1, Sv. Georgi Sofiyski Str., Sofia, Bulgaria<br>Several investigator sites in Europe.
**STUDY OBJECTIVES**	The purpose of this double blind study is as follows:<br>The first objective of this study is to demonstrate the efficacy and safety of pitolisant given at 10, 20, or 40 mg per day versus placebo during 12 weeks of the Double Blind period, to treat the Excessive Daytime Sleepiness (EDS) in patients with Obstructive Sleep Apnea (OSA) refusing the nasal Continuous Positive Airway Pressure (nCPAP) therapy or treated by nCPAP but still complaining of EDS. The efficacy of pitolisant will be assessed separately in patients treated with CPAP and in patients without CPAP use.<br>The secondary objectives of the study include assessing the long-term tolerance as well as the maintenance of efficacy of pitolisant given at 10, 20 or 40 mg per day during 39 weeks of Open Label Extension period and further investigating the co-variates or co-medications that affect the pharmacokinetics of pitolisant in the target population.
**STUDY DESIGN**	Prospective, multicenter, randomized, double blind, phase III study of pitolisant versus placebo during 12 weeks with, at first, an escalating dose period followed by a treatment at the selected dose.<br>Then, after one week of single-blind placebo wash-out period, if the patient holds the same position towards nCPAP therapy as before, an Open Label Extension period is proposed.<br>Patients who do not participate in the Open Label Extension period will have their end of the study visit.<br>Patients willing to continue the pitolisant treatment administration will be given another information notice together with an informed consent form to be signed. This Open Label Extension period will consist of the same escalating-dose, followed by a selected dose period with the active drug only, until 52 weeks after the treatment beginning. Then, patients will have one week wash-out period prior to the end of study visit.
**NUMBER OF PATIENTS**	Approximately 400 patients will be selected, so that 360 patients can be analyzed.<br>They will be divided into 4 groups as follows:<br>• 120 patients with OSA complaining of EDS refusing the nCPAP therapy will be administered pitolisant active ingredient product;<br>• 60 patients with OSA complaining of EDS refusing the nCPAP therapy will be administered a placebo;<br>• 120 patients with OSA treated by nCPAP but still complaining of EDS will be administered pitolisant active ingredient product;<br>• 60 patients with OSA treated by nCPAP but still complaining of EDS will be administered a placebo.

**POPULATION**	Patients exhibiting OSA, experiencing EDS with Epworth Sleepiness Scale (ESS) score ≥ 12, refusing to be treated by nCPAP or having been submitted to nCPAP therapy for a minimum period of 3 months, and still complaining of EDS.
**MAIN INCLUSION CRITERIA**	The patients will have to present the following criteria:<br>• Male and/or female outpatients aged from at least 18 years<br>• Patients complaining of EDS refusing to be treated by nCPAP therapy or having been submitted to nCPAP therapy for a minimum period of 3 months, and still complaining of EDS despite the efforts made beforehand to obtain an efficient nCPAP therapy<br>• Polysomnography performed (for patients submitted to nCPAP therapy – under nCPAP) between V1 and V2 or during the last 12 months with Apnea-Hypopnea Index (AHI): for patients without nCPAP therapy ≥ 15; for patients under nCPAP therapy ≤ 10<br>• For patients submitted to nCPAP therapy: nCPAP ≥ 4 hours / day (compliance checked on the clock-time counter of the CPAP machine)<br>• Mini Mental State Examination (MMSE) ≥ 28<br>• Beck Depression Inventory – 13 items (BDI-13) score < 16 and item G (suicidal ideation) of BDI-13 = 0<br>• Body Mass Index (BMI) ≤ 40 kg/m²<br>• Epworth Sleepiness Scale (ESS) ≥ 12<br>• Female patients with child-bearing potential using a medically accepted method of birth control (i.e. oral contraceptives of normal average dosage) agreeing to continue this method throughout the study, and during the month following treatment discontinuation, being negative to serum pregnancy test performed at the screening visit<br>• If specified by the investigator, the patient must be willing not to operate a car (if sleepy at wheel) or heavy machinery for the duration of the trial or as long as the investigator deems it clinically indicated. In addition, the patient should be willing to maintain during the study their usual behaviors which could affect their diurnal sleepiness (e.g. circadian rhythm, caffeine consumption, nocturnal sleep duration)<br>• Patients having signed and dated the informed consent form
**MAIN NON-INCLUSION CRITERIA**	The patients should not present any of the following criteria:<br>• Patients having previously been exposed to pitolisant either in previous clinical trials, or in a compassionate program or being prescribed the commercial form (Wakix®), for patients enrolled from January 2018<br>• Patients suffering from chronic severe insomnia in accordance with the International Classification of Sleep Disorders (ICSD 2005) without OSA<br>• Patients with co-existing narcolepsy (ICSD 2005), judged on clinical criteria<br>• Patients with sleep debt not due to OSA (according to the physician' s judgment)<br>• Patients with non-respiratory sleep fragmentation (restless leg syndrome…)<br>• Shift work, professional drivers

	• Refusal from the patient to stop any current therapy for EDS or predictable risk for the patient to stop the therapy<br>• Patients suffering from a psychiatric disease<br>• Acute or chronic disease preventing the improvement assessment, e.g. severe chronic obstructive pulmonary disease (COPD)<br>• Current or recent (within one year) history of drug, alcohol, narcotic or other substance abuse or dependence<br>• Any significant serious abnormality of the cardiovascular system, e.g. recent myocardial infarction, angina, hypertension or dysrhythmias (within the previous 6 months), Electrocardiogram Fridericia corrected QT interval higher than 450 ms, history of left ventricular hypertrophy or mitral valve prolapse<br>• Severe co-morbid medical or biological conditions that may jeopardize study participation at the discretion of the investigator (particularly in the cardiovascular system and the instable diabetes)<br>• Positive serology tests (HIV, HCV and HBsAg)<br>• Pregnant or breast-feeding women<br>• Women with child-bearing potential and no efficient birth-control method<br>• Patients unable to understand the study protocol<br>• Patients with suspected or known hypersensitivity to study medication<br>• Patients with a dominant arm deficiency impeding the achievement of the tests<br>• Patients using a prohibited medication<br>• Congenital galactose poisoning, glucose and galactose malabsorption, deficit in lactase<br>• Patients participating in another study or being in a follow-up period for another study
**INVESTIGATIONAL TREATMENT**	Pitolisant and placebo are presented in identical tablets according to dosage, i.e. pitolisant tablets dosed at 5 mg or 20 mg and matching placebo.<br>During the Double Blind period patients will take daily 10 mg (2 tablets of 5 mg) or 20 mg (1 tablet of 20mg) or 40 mg (2 tablets of 20 mg) of pitolisant or matching placebo. During the Open Label Extension period patients will take daily 10 mg, 20 mg or 40 mg of pitolisant (i.e. 2 tablets of 5 mg or 1 or 2 tablets of 20 mg, respectively).<br>Administration by oral route, once a day, in the morning, during breakfast, with a glass of water.
**PROHIBITED TREATMENTS**	All treatments indicated for somnolence, all drugs containing sodium oxybate, hypnotic drugs defined by ATC class; tricyclic antidepressants such as clomipramine, imipramine, desmethylimipramine and protriptyline displaying histamine H1 receptor antagonist activity that may affect the activity of pitolisant by abrogating the effect of its endogenous histamine release; H1 receptor antagonists (in particular those having an effect on the central nervous system), psychostimulants (amphetamine and amphetamine-like CNS stimulants, methylphenidate, modafinil or others); codeine; central antihypertensive drugs(Clonidine), and all drugs containing dextropropoxyphene (Di-Antalvic).

	Similarly, surgical procedures, such as mandibular advancement orthosis, Uvulopalatopharyngoplasty (UPPP) must be excluded.<br>Chronic treatments prescribed at a stable dose since at least one month before V1, unchanged throughout the study period and which do not interfere with daytime sleepiness can be continued and should be noted in the CRF.
**EFFICACY CRITERIA**	**MAIN ENDPOINT:**<br>• **Change of the score of ESS** between the baseline (score at baseline: mean between V1 and V2) and the end of the Double Blind period (mean between V5 and V6)<br>**SECONDARY ENDPOINTS:**<br>• **Percentage of ESS responders**: absolute value < 11 or difference between baseline and the end of the Double Blind period scores ≥ 3<br>• **Reduction of sleepiness and sleep episodes on the sleep diary,** filled in by patients during 3 sequential days of the week preceding each visit<br>• **Improvement in vigilance according to Oxford Sleep Resistance (OSleR) test**: increase in sleep latency and reduction in the number of errors<br>• **Increase in quality of life:** European Quality of Life Questionnaire (EQ-5D), Leeds Sleep Evaluation Questionnaire (LSEQ) and The Pichot Fatigue Scale<br>• **Improvement in cognitive function:** Trail Making Test (TMT) Parts A & B<br>• **Improvement in Clinical Global Impression (CGI-C)** assessment<br>• **Patient's global opinion on the effect of investigational drug**<br>• **Z-score:** composite score including ESS and OSLER results
**SAFETY CRITERIA**	• **Adverse Events** – at each visit<br>• **Physical examination** – at each visit<br>• **Vital signs**: blood pressure and heart rate – at each visit<br>• **Electrocardiogram (ECG)** – at each visit<br>• **Beck Depression Inventory – 13 items** – at visits V1, V2, V6, V7, V9, V10, V11, V12 and V13<br>• **Patient's overall evaluation of the tolerance** – at visits V3, V4, V5, V6, V7, V8, V9, V10, V11, V12 and V13<br>• **Amphetamine-like withdrawal symptoms questionnaire (DSM IV)** – at phone contact 2, V7, phone contact 3 and V13<br>• **Laboratory tests**: evaluation of the biological work-up (hematology, biochemistry at visits V1, V6 and V12, urinalysis at V1). Biological work-up will also be performed as soon as possible for any subject who discontinues the study prior to the completion of the Double Blind period or the Open Label Extension period.<br>• **Overdosing**<br>• **Observance in nightly CPAP use**, as assessed by the clock-time counter of the CPAP machine at each visit (reading of the last day) prior to each visit (for patients submitted to nCPAP therapy).

STUDY SCHEDULE

All tests and examinations to be performed are described in section 2 concerning the study diagram

V1 – Screening visit and beginning of initial wash-out period (D -14)

Before proposing the study to patients refusing to be treated by nCPAP, the investigator will ensure that the patient still refuses this treatment.

An appropriately signed informed consent will be obtained prior to entry into the study for each patient having made a positive decision to participate. The investigator must confirm that the patient meets all inclusion criteria and none of the non-inclusion criteria

Patients included in the study shall not take any treatment indicated for EDS or other psychotropic drugs noted in chapter prohibited treatment during the following two weeks before being submitted to baseline examination (with the exception of chronic medications taken to treat pathology, and authorized by the protocol).

Patients will be reminded to contact the investigator between the visits for any issue, such as adverse events, difficulties with the treatment, need for a new treatment, associated pathology, and modification of the current concomitant treatment (applicable for the whole study duration).

Ph1 – Phone contact (D -7)

During this phone contact, the investigator should:
- Check if patient has discontinued prohibited treatment
- Check concomitant treatments
- Check occurrence of AEs

V2 – Inclusion visit and beginning of the Double Blind period: baseline examinations, randomisation, start of escalating dose phase (D 0)

The patients will be randomized to either pitolisant, or placebo.

The treatment will be initiated by an individual titration period over 2 weeks.

1st week: At each morning, with a glass of water during breakfast

Patients on pitolisant will receive

During breakfast	10 mg

Patients on Placebo will receive

During breakfast	Placebo

2nd week: At each morning, with a glass of water during breakfast

Patients on pitolisant will receive

During breakfast	20 mg

Patients on Placebo will receive

During breakfast	placebo

The total treatment period in double-blind is 12 weeks.

V3 – First dose adjustment visit (D 14)

The posology is increased at 40 mg/d pitolisant (high dose) or placebo every morning,

	during breakfast. If the tolerance does not allow it (occurrence of an adverse event i.e. troublesome insomnia), the patient will continue the treatment intake at 20 mg/d pitolisant (medium dose) or placebo or the posology will be eventually reduced at 10 mg/d pitolisant (low dose) or placebo until V4.<br><br>**V4 – Second dose adjustment visit and beginning of stable dose phase (D 21)**<br><br>The posology is maintained for the next following 4 weeks.<br><br>If the tolerance does not allow it:<br><br>- Patients taking 40 mg/d pitolisant (high dose) or placebo could reduce to 20 mg/d pitolisant (medium dose) or placebo for the following 4 weeks.<br>- Patients taking 20 mg/d pitolisant (medium dose) or placebo could reduce to 10 mg/d pitolisant (low dose) or placebo for the following 4 weeks.<br><br>Any dose increase will not be allowed at this visit.<br><br>After V4 until the end of Double Blind period any dosage change of pitolisant or placebo will not be allowed.<br><br>**V5 – Control visit: continuation of stable dose phase (D 49)**<br><br>Any dosage change of pitolisant or placebo will not be allowed at this visit. Hence, pitolisant or placebo treatment at the same stable dose will be continued for another 5 weeks.<br><br>**V6 – Evaluation visit at the end of the Double Blind period and start of single blind wash-out period (D 84)**<br><br>Patients are submitted to the tests and examinations required for the analysis of the double blind study results. Patients will start one-week single blind placebo wash-out period.<br><br>**Ph2 – Phone contact (V6 + 3D)**<br>During this phone contact, the investigator should:<br>- Get patient's answers to amphetamine-like withdrawal symptoms questionnaire (DSM IV) and find out patient's global opinion on the effect of investigational drug<br>- Check concomitant treatments<br>- Check occurrence of AEs<br><br>**V7 – End of study visit for patients who are not entering Open Label Extension period or Beginning of the Open Label Extension period for patients who are entering Open Label Extension period: start of new escalating dose phase (D 91)**<br><br>If the patient holds the same position towards nCPAP therapy as before, he/she will be proposed to enter the Open Label Extension period of the study after another escalating dose phase.<br><br>Patients not willing to continue the pitolisant treatment administration will end their participation in the study at this visit.<br><br>Patients willing to continue the pitolisant treatment administration will be given another information leaflet together with an informed consent form to be signed. After having signed the informed consent form to confirm that they agree to participate in the Open Label Extension study period, patients will be administered 10 mg pitolisant OD during one week. Then, on the following week, they will be administered 20 mg pitolisant OD (in the morning, during breakfast, with a glass of water).

	**V8-V9 – Dose adjustment visits (D 105 and D 112)**<br><br>The posology is increased to 40 mg/d pitolisant (high dose), every morning, during breakfast. If the tolerance does not allow it (i.e. troublesome insomnia), the patient will get the treatment at dose of 20 mg/d (medium dose) or 10 mg/d (low dose).<br><br>At each of the visits V8-V11 the investigator will propose nCPAP to the patients refusing to be treated by nCPAP therapy before giving them the study treatment. If the patient accepts, he/she will be withdrawn from the study.<br><br>**V10-V11 – Confirmed dose visits (D 196 and D 280)**<br><br>The posology should be 40 mg/d (high dose) if the tolerance is acceptable. If the study drug is not well tolerated, the investigator may decide to decrease the dose to 20 mg/d (medium dose) or 10 mg/d (low dose) during the 12-week period following the visit.<br><br>**V12 – Evaluation visit at the end of the Open Label Extension period and start of wash-out period (D 364)**<br><br>Patients are submitted to the examinations and tests required for the evaluation of long-term tolerance and maintenance of efficacy of pitolisant. Patients will be submitted to a final visit after a one-week wash-out period. No study treatment will be taken during this period.<br><br>**Ph3 – Phone contact (V12 + 3D)**<br>During this phone contact, the investigator should:<br>- Get patient's answers to amphetamine-like withdrawal symptoms questionnaire (DSM IV) and find out patient's global opinion on the effect of investigational drug<br>- Check concomitant treatments<br>- Check occurrence of AEs<br><br>**V13 – End of study visit for patients who entered into Open Label Extension period (D 371)**<br><br>The patients are summoned by the investigator for a final visit. Then, the investigator will decide about the new treatment to prescribe to the patients and the patients will be discharged from the study.<br><br>***The date of each visit could be D ± 3 Days***
STATISTICAL ANALYSIS	Sample Size Determination and Justification:<br><br>Results from exploratory studies on pitolisant allow to estimate the ESS residual variability to SD = 6. The Minimum Important Difference MID was fixed to ESS = 3, corresponding to an effect size ES = 0.5. The correlation between Final and Baseline ESS was conservatively estimated to r = 0.3.<br><br>a) By assuming Analysis of Covariance (ANCOVA) test at 0.95 confidence level as the main confirmatory test, and a sample ratio 1:2, a difference of at least Delta = 3 should be detected with a power of 90% in using at least 60 patients in placebo group and 120 patients in pitolisant treatment group.<br>b) By assuming the same model, an interaction of at least 3 between CPAP and non-CPAP will be detected with a power of 90% when 120 and 240 patients (thus 360 patients in total) are treated in Placebo and pitolisant group respectively. The null hypothesis here should be<br>Interaction $(Y_{pt}-Y_{pl})_{CPAP} - (Y_{pt}-Y_{pl})_{non\text{-}CPAP} > 3$

	Under these two conditions, a stepdown test can be organized: first a test of superiority of pitolisant will be conducted. If this test concludes into superiority, the interaction test will be conducted.<br><br>The sample size will be 120 and 240, (thus at least 360 patients in total) as this size corresponds to the interaction test requiring the largest sample size.<br><br><u>Statistical Analysis:</u><br><br>Final ESS will be compared between the two treatments by an ANCOVA at two-sided 95% in adjusting for ESS at baseline, and by considering the random effect center, and the fixed effect treatment and assessing the additional effect of obesity (BMI) on outcome. This test will be implemented by a Mixed Linear Model. The confirmatory analysis will be based on a simple ANCOVA model assuming no interaction between baseline and treatment (assumption of parallelism). This analysis will be conducted both on the CPAP and non-CPAP groups as primary selections.<br><br>Safety and tolerability will be assessed by summarizing and analyzing adverse events (AEs), change in physical examination, vital signs, electrocardiogram (ECG) and laboratory data.
**STUDY DURATION AND DATES**	The study will be conducted in two periods.<br><br>Depending on whether the patients will participate in the Double Blind period only, or whether they will continue to be administered the pitolisant treatment in the Open Label Extension period, the study duration for each patient will be 15 weeks, or 55 weeks.
**PLANNED STUDY START**	First patient in: 1-2 Q 2016
**PLANNED END OF THE STUDY**	Last patient last visit: 2 Q 2019

2. STUDY DIAGRAM

Double Blind period

PERIOD / Visit [1]	V1 D -14	1 Week Wash-out period	Phone cont. 1 D -7	1 Week	V2 D 0	W 1 10 mg	W 2 20 mg	V3 D 14	W 3 10 mg or 20 mg or 40 mg	V4 D 21	W 4 → W 7 Selected dose (4 weeks)	V5 D 49	W 8 → W 12 Selected dose (5 weeks)	V6 D 84	W 13 1 week wash-out period	Phone cont. 2 V6 +3D	V7 [6] End of study D 91
Signature of Consent Form	X																
Medical questionnaire	X		X		X			X		X		X		X		X	X
Physical examination	X				X			X		X		X		X			X
ESS	X				X			X		X		X		X			X
Polysomnography [2]				X													
OSleR test [3]					X									X			
MMSE	X																
TMT parts A & B					X									X			
CGI-S	X				X												
CGI-C														X			X
BDI-13	X				X									X			X
LSEQ, EQ-5D					X									X			
Pichot Fatigue Scale					X									X			X
Patient's global opinion on the effect of investigational drugs														X		X	X
Patient's overall evaluation of the tolerance								X		X		X		X			X
Amphetamine-like withdrawal symptoms questionnaire																X	X
ECG	X				X			X		X		X		X			X
Safety biology [4]	X													X			
Delivery of sleep diary	X				X			X		X		X		X			
Review of sleep diary [5]					X			X		X		X		X			X
Adverse events			X		X			X		X		X		X		X	X

1 – Each visit shall be carried out at the end of the relevant time period ± 3 days.
2 – Overnight polysomnographic recording performed from 22:00 until 7:00 (minimum 8 hours of recording) in the sleep laboratory only between V1 and V2 except if available in the previous 12 months.
3 – OSleR test: 3 sequences at 2 hours interval (at 9:00, 11:00, and 13:00).
4 – Complete biological examination: hematology (hemoglobin, hematocrit, red and white blood cell count (with differential), platelets, mean corpuscular volume, coagulation time (INR)), biochemistry (blood urea nitrogen (BUN), uric acid, creatinine, creatine kinase, ALAT, ASAT, GGT, alkaline phosphatases, total protein, total bilirubin, glucose, electrolytes (sodium, potassium, calcium, chloride, bicarbonates/CO_2), total cholesterol, triglycerides), serology (HIV, HCV, HBsAg at V1, β-HCG (for woman with child-bearing potential)), urianalysis: stick (with microscopy and bacteriological culture, if positive) at V1.
5 – At each visit, the patient shall bring back his sleep diary. Patient will be contacted in advance before each visit to remind him/her to fill in the sleep diary. The patient shall return the unused drug at each visit.
6 – Only for patients who are not entering into Open Label Extension period; for others see Open Label Extension period study diagram.

Study Harosa III P1513 / BF2.649
Double Blind period flow-chart

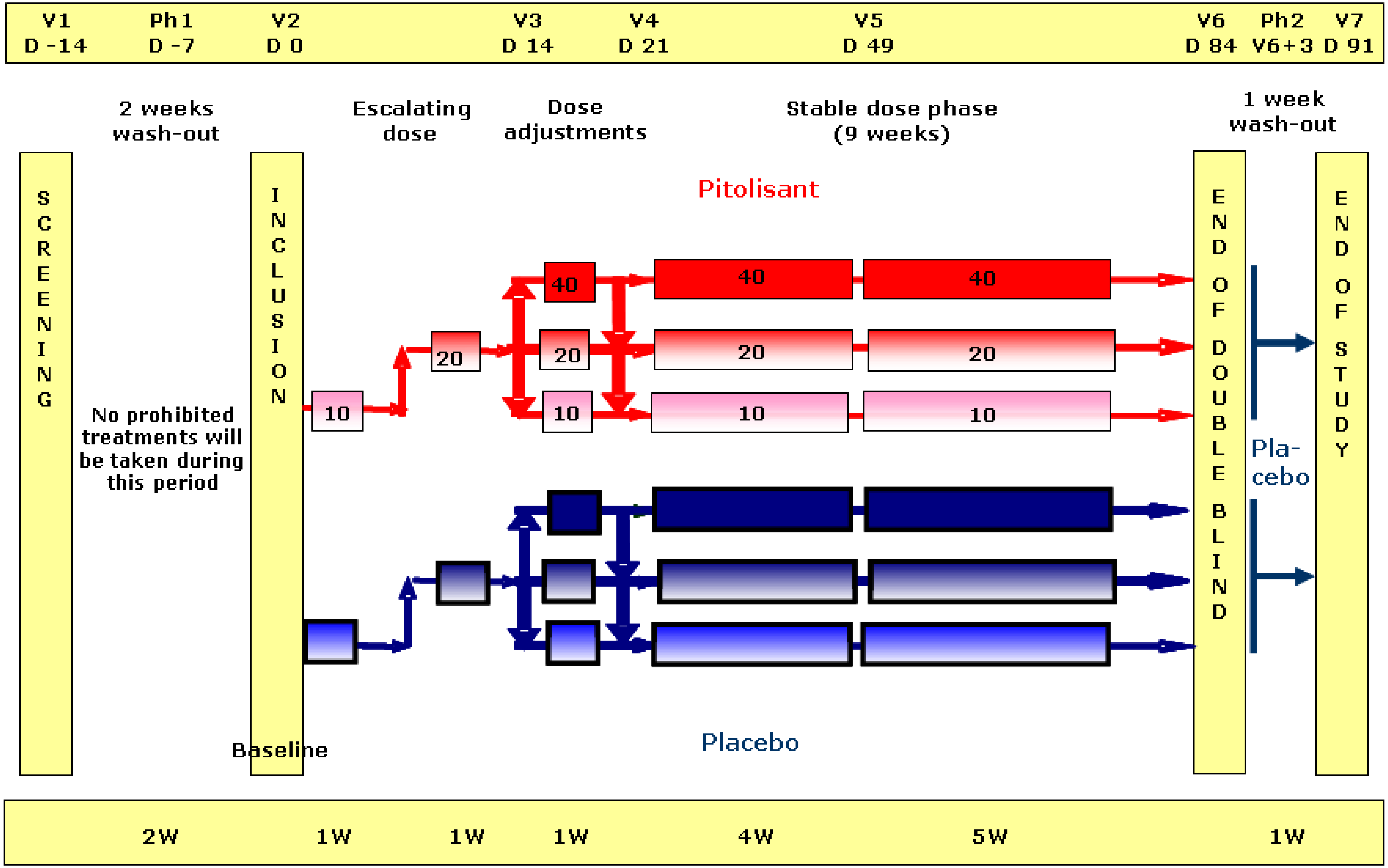

Open Label Extension period

PERIOD		W 14	W 15		W 16		W 17 → W 28		W 29 → W 40		W 41 → W 52		W 53		
**Visit** [1]	**V7** [2] D 91	10 mg	20 mg	**V8** D 105	10 mg or 20 mg or 40 mg	**V9** D 112	10 mg or 20 mg or 40 mg	**V10** D 196	10 mg or 20 mg or 40 mg	**V11** D 280	10 mg or 20 mg or 40 mg	**V12** D 364	1 week Wash-out period	**Phone cont. 3** V12 +3D	**V13** End of study D 371
Signature of Consent Form	X														
Medical questionnaire	X			X		X		X		X		X		X	X
Physical examination	X			X		X		X		X		X			X
ESS	X			X		X		X		X		X			X
OSleR test [3]	X											X*			
TMT parts A & B	X											X			
CGI-C	X							X		X		X			X
BDI-13	X					X		X		X		X			X
LSEQ, EQ-5D	X											X			
Pichot Fatigue Scale	X					X		X		X		X			X
Patient's global opinion on the effect of investigational drugs	X							X		X		X		X	X
Patient's overall evaluation of the tolerance	X			X		X		X		X		X			X
Amphetamine-like withdrawal symptoms questionnaire	X													X	X
ECG	X			X		X		X		X		X			X
Safety biology [4]												X			
Delivery of sleep diary	X			X		X		X		X		X			
Review of sleep diary [5]	X			X		X		X		X		X			X
Adverse events	X			X		X		X		X		X		X	X
Pharmacokinetics sampling [6]								X							

1 – Each visit shall be carried out at the end of the relevant time period ± 3 days.
2 – Only for patients who are entering into Open Label Extension period; for others see Double Blind period study diagram.
3 – OSleR test: 3 sequences at 2 hours interval (at 9:00, 11:00 and 13:00). Optional at V12.
4 – Complete biological examination: hematology (hemoglobin, hematocrit, red and white blood cell count (with differential), platelets, mean corpuscular volume, coagulation time (INR)), biochemistry (blood urea nitrogen (BUN), uric acid, creatinine, creatine kinase, ALAT, ASAT, GGT, alkaline phosphatases, total protein, total bilirubin, glucose, electrolytes (sodium, potassium, calcium, chloride, bicarbonates/CO_2), total cholesterol, triglycerides), serology (β-HCG (for woman with child-bearing potential)).
5 – At each visit, the patient shall bring back his sleep diary. Patient will be contacted in advance before each visit to remind him/her to fill in the sleep diary. The patient shall return the unused drug at each visit except at V13.
6 – for a pre-defined number of patients.

Study Harosa III P1513 / BF2.649
Open Label Extension period flow-chart

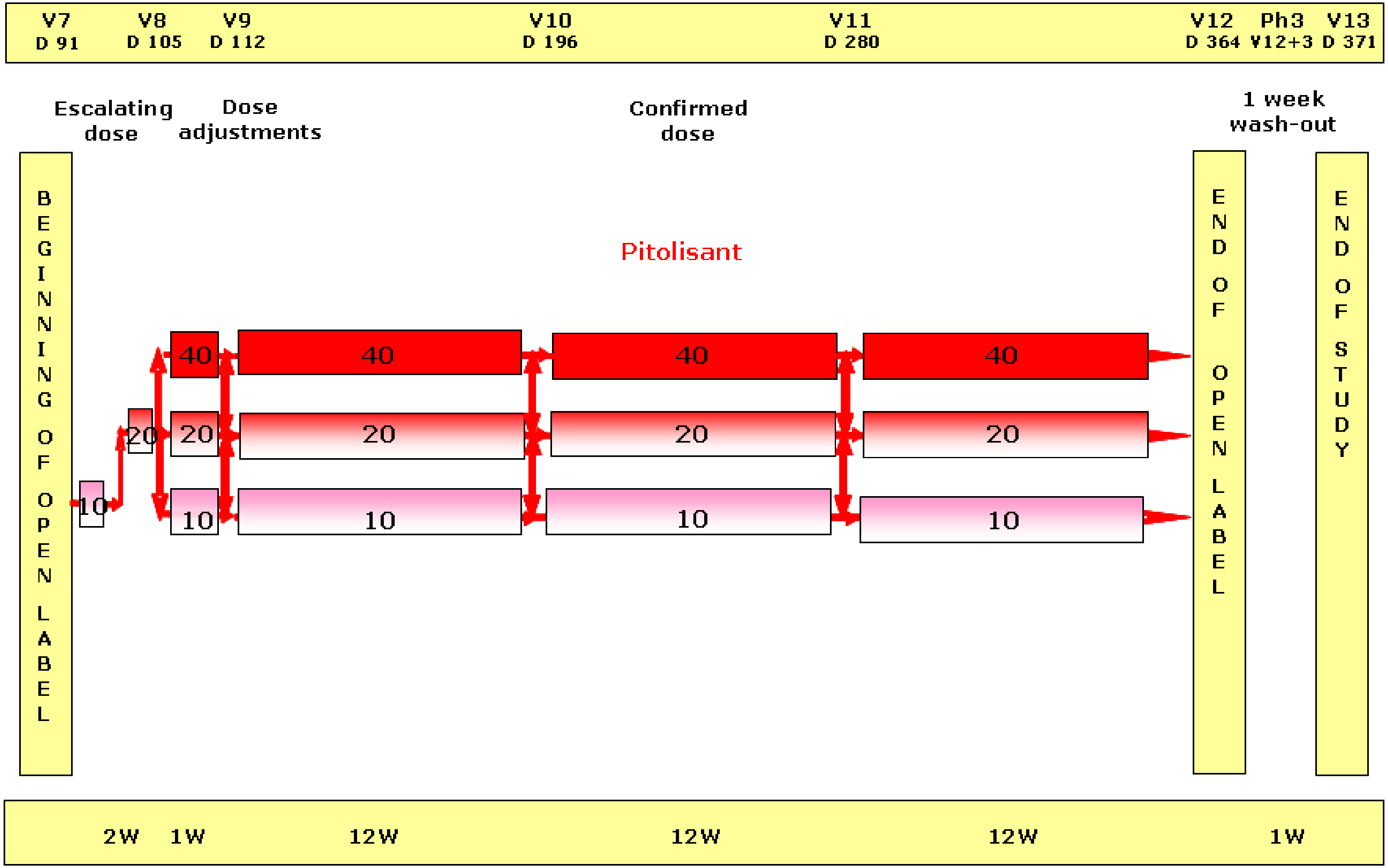

ABBREVATIONS USED IN THE PROTOCOL

5-HT	Serotonin or 5-hydroxytryptamine
ADHD	Attention Deficit/Hyperactivity Disorder
AEs	Adverse events
AHI	Apnea Hypopnoea Index
ALAT/SGPT	Alanine aminotransferase
ANCOVA	Analysis of Covariance test
APD50	Action potential duration at 50% repolarization
APD90	Action potential duration at 90% repolarization
ASAT/SGOT	Aspartate aminotransferase
ATC	Anatomical Therapeutic Chemical classification system
AUC	Area under the concentration-time curve
BDI-13	Beck Depression Inventory – 13 items
BMI	Body mass index
CGI	Clinical Global Impression
CGI-C	Clinical Global Impression – Global Improvement or Change
CGI-S	Clinical Global Impression – Severity of Illness
Cmax	Maximum observed concentration
CNS	Central nervous system
COPD	Chronic obstructive pulmonary disease
CPAP	Continuous Positive Airway Pressure
CPK	Creatine kinase
CRF	Case report form
CRO	Contract Research Organisation
DMB	Data Management Book
DSMB	Data Safety Monitoring Board
DSE	Diurnal Sleepiness episodes
EC	Ethics Committee
ECG	Electrocardiogram
EDS	Excessive Daytime Sleepiness
EEG	Electroencephalography
EMEA	European Medicines Agency
EMG	Electromyography channel
ES	Effect Size
ESS	Epworth Sleepiness Scale

EQ-5D	European Quality of Life Questionnaire
FAS	Full analysis set
GABA	Gamma-aminobutyric acid
GCP	Good Clinical Practice
GMP	Good Manufacturing Practice
GGT	Gamma-glutamyltranspeptidase
H3R	Histamine H3 receptor
ICH	International conference on harmonization of technical requirements for registration of pharmaceuticals for human use
ICSD	International Classification of Sleep Disorders
INR	International Normalized Ratio
IRB	Institutional Review Board
MCI	Mild Cognitive Impairment
MID	Minimum Important Difference
MMSE	Mini Mental State Examination
nCPAP	nasal Continuous Positive Airway Pressure
NOAEL	No observable adverse effect levels
OD	Once a day
ODL	Optimal Dose Level
OSA	Obstructive Sleep Apnea syndrome
OSleR	Oxford Sleep Resistance test
PP	Per Protocol
PSG	Polysomnography
SAE	Serious Adverse Event
SaO_2	Oxygen Saturation
SAP	Statistical Analysis Plan
SD	Standard Deviation
ß-hCG	Human chorionic gonadotropin
Tmax	Time to Cmax
TMT	Trail Making Test

3. INTRODUCTION

Excessive daytime sleepiness (EDS) is a major complaint in patients with obstructive sleep apnoea syndrome (OSA) defined as sleep-related breathing disorder (SRBD) with full or partial occlusion of the upper airway during the sleep. OSA afflicts at least 2-4% of the adult population (at least 4% of males and 2% of females)[1,2].

The proposed mechanism for EDS in OSA patients is sleep disturbance and loss of sleep resulting from microarousals produced by increased ventilatory effort[3,4].

Patients may be personally unaware of their snoring and breathing pauses during sleep, but the consequences of daytime hypersomnolence such as deficits in attention or vigilance (road accident, accident at work)[5] impaired short-term memory and reduced capacity to sustain concentration or focus are often essential to rational clinical decision about whom to treat [6].

Nasal continuous positive airway pressure (nCPAP) is the treatment of choice for most patients with OSA[7,8,9,10]. When used properly, nCPAP reduces apnoea and hypopnoea rate, often normalizes arterial blood oxygen saturation, decreases sleep fragmentation, and improved sleep quality. As a result, alertness, mood, cognitive function, and quality of life improve[11]. Furthermore nCPAP reduces risks of cardiovascular events in obstructive sleep apnea patients.

Pitolisant (BF 2.469), novel selective histamine H3 receptor antagonist/agonist inverse exhibits strong waking promoting effects and a good tolerance in a number of preclinical and clinical studies, appeared completely indicated.

Experimental evidence for enhanced wakefulness induced by H3R blockade was previously reported with thioperamide, the prototypic H3R antagonist [*Lin et al., Brain Res. 1990, 523: 325-30*]. Similar vigilance- and attention-promoting effects were described more recently with the antagonist ciproxifan [*Ligneau et al., J. Pharmacol. Exp. Ther. 1998, 287: 658-66*].

Improvement of learning deficit and memory by an H3 antagonist was also demonstrated experimentally in mice [*Miyazaki et al., Life Sci. 1995, 57: 2137-44; Meguro et al., Pharmacol. Biochem. Behav. 1995, 50: 321-5; Onodera et al., Naunyn-Schmiedeberg's Arch. Pharmacol. 1998, 357: 508-13*]. The effects of pitolisant on sleep/wakefulness, and EEG patterns have been investigated in cats and mice. It has been shown that pitolisant increased markedly the duration of waking in these two animal species. Moreover, the EEG results suggest that pitolisant could improve the quality of arousal, by reinforcing e.g. the level of vigilance and attention, in the absence of any noticeable psychomotor activation that is observed with drugs like amphetamines.

In healthy volunteers, the clinical and biological tolerance of pitolisant was excellent with single oral dose between 1 mg and 240 mg.

3.1. Summary of relevant non clinical studies

3.1.1. Pharmacological Profile

BF2.649 is a novel, highly potent, selective, orally active histamine H3 receptor antagonist/inverse agonist with a Ki between 0.3 nM and 2.4 nM at the human receptor. It does not interact significantly with other histamine receptor subtypes or with a large variety of other receptors.

When given parenterally or orally, BF2.649 enhances the activity of histaminergic neurons in brain, a major wake-promoting system, and thereby improves vigilance, learning and memory and displays pro-cognitive properties. BF2.649 exhibits strong waking effects in several animal species (rodents and cats) and improves the level of vigilance, attention and learning without causing locomotor excitation. BF2.649 showed strong waking effects on normal animals as well as on experimental models of narcolepsy and Parkinson's disease. In agreement, it enhances the activity of major arousal and procognitive systems *projecting to cortical areas* (prefrontal cortex, hippocampus), i.e. not only histaminergic, but also noradrenergic, cholinergic and dopaminergic. However, this pro-waking and pro-cognitive profile is clearly distinct from those of typical psychostimulants of the amphetamine type, including modafinil: *dopamine release is unaffected* in the striatal complex (including the nucleus accumbens), psychomotor activation is not elicited (and methamphetamine-induced locomotor activation is even depressed) and behavioural sensitization does not develop upon repeated administration.

In an animal model of narcolepsy (orexin-knockout mouse) the waking effect is accompanied by a marked inhibition of DREMs (direct transitions from Wake to REM sleep, occurring during cataplexies), again an effect not observed with psychostimulants including modafinil.

Also in animal studies, BF2.649 showed a beneficial effect on models corresponding to human absence epilepsy or temporal lobe seizures. BF2.649 was effective in animal models of schizophrenia, i.e. to block partially the psychomotor responses to amphetamine or dizocilpine. It also decreases the locomotor activity and counteracts the cognitive deficits of dopamine-transporter knock out mice, a model of psychosis and ADHD. It also potentiates the "antipsychotic" effect of risperidone and olanzapine in e.g. dizolcipine-induced hyperlocomotion.

3.1.2. Safety profile

Safety profile has been assessed on cardiovascular, respiratory, gastric and central nervous functions. Some effects were reported, but only at high concentrations or high doses, when compared to Human therapeutic doses (~ 20 mg/day, repeated):

BF2.649 dose-dependently inhibited hERG channels stably expressed in HEK293 cells, with an IC50 value of 1.32 µM to be compared with therapeutic plasma levels of about 0.4 µM (total concentration) and 0.01 µM (free concentration). IC50 values of BF2.649 on a large panel of cardiac channel currents (Nav1.5, Kv4.3, Kv7.1/mink, Kir2.1, Kv1.5, Cav1.2 and Cav3.2) were above 10 µM or ~ 10 µM (Cav1.2, Kv4.3). On human ventricular cardiomyocytes, BF2.649 decreased significantly APD20 (26±3% and 38±1% at 3 and 10 µM, respectively) and increased non-significantly APD90 by 17±9%, 18±8% and 11±4% at 1, 3 and 10 µM, respectively. Dofetilide (0.1 µM) induced a significant increase of +23±2% in APD90 which was fully abolished when the BF2.649 (10 µM) was associated with dofetilide (100 nM). These results are consistent with the inhibitory action on hERG channel and a calcium blocking effect of BF2.649 recorded at BF2.649 concentration higher than 1 µM. In anaesthetized rabbits, BF2.649 did not significantly affect corrected QT interval and no arrhythmic event was observed even at the highest dose. In addition, in the methoxamine-sensitized rabbit, BF2.649 devoid of any pro-arrhythmic effect by itself was even able to reduce arrhythmias score of clofilium, an agent know to prolong the cardiac repolarization phase. In conscious freely moving rats, BF2.649 (15 mg/kg, p.o.) did not impact on the main ECG parameters (PR, QRS or QT interval). Assessment of cardiovascular risk of BF2.649 (5, 10 and 15 mg/kg, p.o. or 1.5 mg/kg, i.v.) in the conscious male and female Beagle dog monitored by telemetry evidenced no effect of BF2.649 on arterial blood pressure, heart rate, the PR, the QRS, the QT and the QTc intervals. No arrhythmia or other changes in the morphology of the electrocardiogram were observed. These data suggest that BF2.649 would possess no proarrhythmic properties in the context of prolonged QT.

On the respiratory functions, BF2.649 (4 mg/kg and above, i.v.) only increased the tidal volume.

On the central nervous system, no significant effect of BF2.649 was observed at doses up to 30 mg/kg, p.o. in global behaviour and central nervous system activity screen (FOB assay). Straub tail and convulsions were observed only at high doses (60 mg/kg and above in rodents), but no impairment of motor coordination was detected. BF2.649 over the dose-range 3-60 mg/kg, p.o.

was without any effect in the Barbital interaction (sleep induction) test in rats. BF2.649 did not occasion conditioned place-preference or behavioural sensitization in rats or the discriminative stimulus effects of cocaine in mice, and was not self-administered in monkeys: this is consistent with its lack of effects on dopamine release in the striatal complex and, by inference, it would not likely elicit psychostimulant's subjective effects in humans nor be abused like psychostimulants. No dependence was elicited by BF2.649 as evidenced by the lack of withdrawal signs following a sub-chronic treatment of rats.

BF2.649 was found devoid of any effects on gastric acid secretion or ulcer formation in rats.

3.1.3. Pharmacology of pitolisant in potential therapeutic indication

Studies conducted to date confirmed that pitolisant can counteract H3R agonist-mediated alterations in central histamine activity in several models.

3.1.3.1. Effects on Sleep/wakefulness and EEG patterns in cats and mice

The effects of pitolisant on sleep-wake cycle control have been investigated in freely moving cats (*Buda et al., 2002*) and mice (*Parmentier et al., J Neurosci. 2002, 22, 7695-711*).

Oral administration of pitolisant caused dose-dependent increase in the duration of waking in two animal species: cats and mice. Moreover, the EEG results suggest that pitolisant could improve the quality of arousal, by reinforcing e.g. the level of vigilance and attention, in the absence of any noticeable psychomotor activation which is observed with drugs like amphetamines. When given parenterally or orally, pitolisant enhances the histaminergic transmissions in brain, and thereby improves vigilance, learning and memory, and displays pro-cognitive properties.

In a model of narcolepsy, orexin-KO mice, pitolisant showed strong waking, and antinarcoleptic effects. Oral administration of pitolisant (20 mg/kg, po) significantly improved wakefulness during the awakening period, and considerably reduced the narcoleptic episodes.

On the other hand, the combination of modafinil (64 mg/kg) and pitolisant (20 mg/kg) showed a clear synergistic activity on sleep/wakefulness and narcoleptic periods in this narcolepsy model.

3.1.3.2. Effects on Learning

Behavioural studies have shown that pitolisant at the dose of 15 mg/kg significantly reduced (p = 0.0056) scopolamine-induced amnesia and "natural" forgetting in two mice models, suggesting that pitolisant has significant effects on learning and memory.

Two experimental models were used to confirm this hypothesis:

1. Experimental model of amnesia: In this model scopolamine is used to induce learning deficit in mice which is estimated by using the two - trial object recognition paradigm [*Ennaceur and Delecour, Behav. Brain Res., 1988, 31, 47-59*].

   This test is considered as a model of episodic memory and has been shown to be sensitive to the effects of aging and cholinergic dysfunction induced by scopolamine [*Scali et al., Neurosci. Lett., 1994, 170, 117-120; Bartolini et al., Biochem Behav., 1996, 53, 277-283*].

   A 5 mg/kg dose of pitolisant did not modify the time for mice to reach the learning criteria on the first trial whereas this time was significantly increased at a higher dose (15 mg/kg), a change which might reflect enhanced "curiosity" towards the whole novel environment.

2. Experimental model of Natural Forgetting in mice. In this test, the time spent by mice to achieve the exploration of a familiar object is shorter than the time spent for a novel object. In this model, histamine-deficient mice (Histidine-decarboxylase KO mice) showed deficiency to explore objects and to discriminate novel objects from familiar objects *(Dere et al, 2003*). The results of the study (learning test of recognition) conducted in mice suggest that the "natural" forgetting is significantly reduced by tacrine but also by pitolisant at the dose of 15 mg/kg.

These observations of a reversal by pitolisant are consistent with a large variety of observations gathered with the other H3R antagonists/inverse agonists, including the prototypical drug thioperamide in similar models [*Ghi et al., Pharmacol. Biochem. Behaviour, 1999, 64, 761-766; Molinengo et al., Pharmacol Biochem Behav., 1999, 63, 221-227; Miyazaki et al., Life Sci., 1995, 57, 2137-2144; Orsetti et al., Behav. Brain Res., 2001, 235-242*]

This pro-cognitive effect of pitolisant might be related to the direct "arousing" effect of endogenous histamine (e.g. at the level of the cerebral cortex, hippocampus or thalamus) as well as to its capacity to increase acetylcholine-release in brain [*Blandina et al., Brit. J. Pharmacol., 1996, 119, 1956-1664*].

3.1.3.3. Effects on Attention Deficit/ Hyperactivity Disorder (ADHD)

Dopamine-Transporter ($DAT^{(-/-)}$) Knock-out mice have been suggested as a model for ADHD because of their behaviour. These mice exhibit high extracellular dopamine levels in brain, and are reported to be hyperactive in the open field *(Giros et al., Nature* 1996, 379, 606-12).

In this experimental model of hyperactivity, pitolisant (6 mg/kg) decreased the horizontal locomotor activity and the number of rearings, an effect similar to the paradoxical effect of amphetamine-like stimulants which are currently used in the treatment of adult or child ADHD.

However, there is an important difference in the profile of the two classes of compounds: in contrast with amphetamines and methylphenidate, pitolisant has (1) no psychomotor stimulant effect in normal animals, (2) no sympathomimetic effect leading to hypertension, (3) no abuse potential, i.e. important drawbacks which are likely leading to the imposition of a "black label" to this drug class and the present limitation for their use in European countries.

3.1.3.4. Effects on Epilepsy Disorders

The potential benefit of pitolisant in epilepsy has been confirmed in various models of seizure in rats and mice. Animal studies indicate that pitolisant could have a beneficial effect on typical absence epilepsy in human as well as on temporal lobe seizures, a form of seizures which is generally drug resistant.

Pitolisant significantly decreased both the number and cumulated durations of spike and wave discharges 20, 40 and 60 minutes after its administration in Genetic Absence Epilepsy Rats of Strasbourg (GAERS), a very predictive model for absence epilepsy [*Danober et al., Prog. Neurobiol., 1998,55, 27-57*]. These results suggested that pitolisant should have an effect on typical absence epilepsy in human. In kainate-induced hippocampal seizures in mice, pitolisant (10 mg/kg) significantly reduced the cumulated duration and the number of hippocampal discharges during the first 40 min after administration. The anti-epileptic effects observed in kainate mice suggest that pitolisant could be effective on temporal lobe seizures, a form of seizures which is generally drug resistant.

3.1.3.5. Effects on psychotic diseases

Pitolisant was found to be effective in various models of schizophrenia such as the models of

Methamphetamine-Induced Locomotor Activation, Apomorphine-Induced Climbing Behaviour and MK-801-Induced Locomotor Activation in Mice.

In these experimental models, pitolisant clearly reduces the hyperactivity induced by dopamine stimulation (methamphetamine and apomorphine models), or by glutamate inhibition (MK-801 model). These results suggest that BP2.649 could have a clinical interest in schizophrenia where glutamatergic/dopaminergic imbalance is suspected to occur.

3.1.4. Toxicology

3.1.4.1. Summary

The acute toxicity of BF2.649 has been evaluated by the intravenous route in mouse and rat, and by the oral route in mice, rats, dogs and monkeys. Repeat oral dose toxicity studies have been performed in rats and monkeys.

- Acute oral toxicity in rodents:

  BF2.649 demonstrated a good safety profile in rodents. In mice, the no effect dose was > 30 mg/kg orally and > 5 mg/kg, i.v., with a maximum non lethal oral dose of 100 mg/kg. The minimum lethal doses were 150 mg/kg, p.o. and 10 mg/kg, i.v. In rats, the no effect dose was > 50 mg/kg, orally and 12 mg/kg i.v., with a maximum non lethal oral dose of 100 mg/kg (expressed as single doses). On a mg/kg basis, the no-effect doses were ≥ 38 times higher than the highest BF2.649 dose to be studied in Phase III (i.e., 40 mg; based on a 50-kg human).

- Repeat oral dose toxicity studies in mice, rats and monkeys:

  In a 4-week repeat dose toxicity study, the dose-level of 75 mg/kg/day of BF2.649 given by oral gavage for 4 weeks to CB6F1-nonTgrasH2 mice was considered to be the No Observed Adverse Effect Level (NOAEL) and elicited slight CNS signs (mainly transient hypoactivity) in a limited number of mice. A safety margin of 38 was deduced. At 100 mg/kg/day, hypoactivity was the most frequent and remarkable clinical sign recorded, with a higher frequency in males (lasted 13 days in males and 10 days in females), associated with clonic convulsions in males (5/10) on 1 or 2 days. No convulsions were observed in females. Several cases of staggering gait and a few cases of loss of balance were noted in males and females as well as a few cases of ptyalism and reflux at dosing in males.

  In rats, the 13-week and 6-month repeat dose studies showed satisfactory tolerability of

BF2.649 up to 30 mg/kg/day, a dose which can be defined as the NOAEL. In the 13-week study, mortality was observed at doses ≥75 mg/kg. The cause of death could not be directly established, but a number of rats that died had lung lesions; in addition, adverse effects on the cardio-respiratory and/or CNS could not be ruled out. In the 6-month study, the 60 mg/kg/day dose showed evidence of CNS toxicity, such as convulsions and tremors without any associated brain histopathology finding. At this dose, other target organs included the adrenals, duodenum, liver, and lungs. The NOAEL dose was found to be of 30 mg/kg/day, leading to a safety margin of 19 when taking into account the sum of BF2.649 and its metabolite BP1.2526 abundant in rats but not in human (a separate trial had established the pro-convulsive potential of this metabolite at exposure levels comparable to those found at convulsive doses of BF2.649).

In monkeys, the 13-week and 9-month repeat dose studies showed no adverse effects of BF2.649 up to 5 mg/kg/day. At 12 mg/kg/day in the 13-week and 9-month studies, occasional emesis occurred only in a very limited number of monkeys. No other significant effects were recorded. Hence, this dose can be considered as the NOAEL in this species leading to a safety margin of 4.3. At a higher dose (30 mg/kg/day) in both studies, the main findings observed in several animals were clinical signs including emesis, tremors and/or occasional convulsions. Some slight changes in serum biochemistry were also reported in the 13-week study (e.g. increases in aminotransferases).

Regarding the main non-conjugated metabolite in human (BP2.951) which did not enter the brain and therefore cannot be responsible for CNS side effects, safety margin ratio calculation provided a value of 6.8 when comparing corresponding AUC at NOAEL doses in monkeys, the species having also BP2.951 as main non-conjugated metabolite, to its AUC in humans at therapeutic dose. Similar calculations made in mice and rats, two species having not BP2.951 as main metabolite, provided safety margins ratio of 3.7 and 1.3, respectively.

Regarding other main metabolites in human, BP1.8054, a glycine conjugated metabolite which was not present in toxicity species, a specific 13-week toxicity study in the rat provided a NOAEL dose at the highest oral dose tested of BP1.8054 (300 mg/kg/day). Safety margin ratio calculations provided values of at least 265 when comparing corresponding AUC at the NOAEL dose to its AUC in humans at therapeutic doses. BP1.9733, another conjugated metabolite (glucuronide) was present at significant levels in the monkey receiving BF2.649. The 9-month toxicity study in this species provided a

NOAEL dose of 12 mg/kg/day and safety margin ratio calculations provided values of at least 14 when comparing corresponding AUC at the NOAEL dose to its AUC in humans at therapeutic doses.

➢Carcinogenicity:

Two studies were performed:

The 6-month carcinogenicity in CB6F1 TgrasH2 transgenic mice evidenced that the Maximum Tolerated Dose was achieved, based on the clinical signs observed at the dose-levels of 75 mg/kg/day, and on the non-neoplastic histopathological findings seen in the liver and testes from animals treated at 30 mg/kg/day or 75 mg/kg/day. Following the administration of BF2.649 at all the doses investigated (including the high dose-level of 75 mg/kg/day) for 26 weeks, there were no neoplastic findings related to the test item administration in male or female mice. Consequently, under the experimental conditions of this study, the dose-level of 75 mg/kg/day eliciting a significant BF2.649 exposure (C_{max} of 2584 ng/mL and 1634 ng/mL and AUC_{0-24h} of 9896 ng/mL*h and 7228 ng/mL*h in males and females, respectively) was considered to be non carcinogenic in CB6F1-TgrasH2 mice. These can be compared to pharmacokinetic parameters of BF2.649 in humans at expected therapeutic level (C_{max} of 72 ng/mL and AUC_{0-24h} of 804 ng/mL*h) indicating safety margins of 23 and 8,8, respectively.

A 2-year carcinogenicity study in Sprague-Dawley rats investigating the dose-levels of 15, 30 and 60 mg/kg/day, by oral route was performed. Based on the clinical signs and effects on body weight gain observed in animals at the dose-levels of 30 mg/kg/day, the Maximum Tolerated Dose was achieved. Following the administration of BF2.649 at all the doses investigated for 105 weeks, there were no neoplastic findings that were attributed to the test item administration. Consequently, under the experimental conditions of this study, the dose-level of 30 mg/kg/day eliciting a significant BF2.649 exposure (mean exposure values in males for C_{max} of 182 ng/mL and AUC_{0-24h} values of 1565 ng/mL*h in males at week 104), was not carcinogenic. These values, when compared to pharmacokinetic parameters of BF2.649 in humans at therapeutic level (C_{max} of 72 ng/mL and AUC_{0-24h} of 804 ng/mL*h) indicate safety margins of 2,5 and 1,9, respectively

➢Genotoxic potential of BF2.649 has been assessed in two *in vitro* systems and one *in vivo* assay. Pitolisant has been found not to be genotoxic (no mutagenic nor clastogenic) *in vitro* or *in vivo* as well as its major metabolites in rats (BP1.2526 and BP2.951) and humans (BP2.951 and BP1.8054) which were tested *in vitro* for mutagenicity *(*BP1.2526, BP2.951

and BP1.8054) and for clastogenicity (BP1.8054).

➢Reproductive and developmental toxicity:

Reproductive and developmental toxicity was evaluated in a battery of studies, BF2.649 did not show any adverse effects on fertility and early embryonic development at 30 mg/kg and this dose was a NOAEL in this study ensuring a safety margin of 3.9. At higher doses, CNS clinical signs, increased post-implantation losses, and sperm alterations were reported.

In an *embryotoxicity study in rats*, maternal toxicity and foetal weight reductions were evident at 90 and 110 mg/kg/day p.o. (the highest doses tested). No treatment-related malformations were observed up to the highest dose tested, leading to a safety margin of at least 3,7.

In an *embryotoxicity study in rabbits* at doses up to 150 mg/kg/day, p.o., malformations were observed at 150 mg/kg in the presence of maternal toxicity with a frequency similar to control. The NOAEL for foetal malformations was 67 mg/kg. However, the administration of BF2.649 by oral route provided a low drug exposure in the rabbits. Therefore, additional studies were performed by intramuscular route in order to ensure significant drug levels investigating BF2.649 at the doses of 4, 8 and 16 mg/kg/day i.m. From these, the NOAEL for maternal parameters was considered to be 4 mg/kg/day and the NOAEL for effects on embryo-foetal development was considered to be 8 mg/kg/day, leading to a safety margin of 1.3. There was no evidence of a specific dysmorphogenic effect following treatment of the dams with BF2.649.

During the *pre- post-natal development toxicity study*, BF2.649 induced clinical central signs, dystocia during delivery in pregnant rats, increase in dead-born pups and some major malformations at the dose of 90 mg/kg. Some alterations of the maternal nursing behaviour and retardation in the physical and motor development of F_1 generation were reported at 52 mg/kg. The 30 mg/kg/day dose was considered as the NOAEL, leading to a safety margin of 3,9.

In a *juvenile toxicity study in rats*, BF2.649 doses of 9, 21 and 48 mg/kg were administered daily by *oral route* from postnatal day (PND) 7 to postnatal day 70. At the two high doses, some premature deaths occurred and CNS signs and ptyalism were observed. There were also histopathologic changes in the lung and larynx (mostly foreign body granulomas) at highest doses. No effect on tibia growth and pre-weaning development, sexual maturation, pairing, mating and fertility, learning and memory, motor activity or reflexes were

evidenced. Therefore, the NOAEL dose was established at ~ 9 mg/kg/day corresponding to a low BF2.649 exposure characterized by AUC_{0-24h} values of ~ 100 ng/mL.h and ~ 30 ng/mL.h on PND7 and PND34, respectively. As lung disorders could be due to the penetration of the test item in the airways during gavage, additional investigations were initiated using the intraperitoneal route to avoid these phenomenons and to ensure a higher exposure.

Intraperitoneal administration of BF2.649 daily at 15 or 30 mg/kg/day or twice daily (8 hours apart) at 30 mg/kg/day in the juvenile Sprague-Dawley rat from 7 days of age (PND7) up to 10 weeks of age (PND70) induced a dose-related mortality and convulsive episode at 30 mg/kg/day and 2x30 mg/kg/day. There was no effect on the subsequent development neither on the reproductive function. Pathological changes were limited to microscopic changes in the lungs which consisted of a dose-dependent increase in alveolar macrophages mainly of minimal severity at all doses and both sexes an with a very limited occurrence at the low dose, and pigment in histiocytes in males treated at 30 mg/kg/day and in males and females treated at 2x30 mg/kg/day. Following a treatment-free period, increased alveolar macrophages appeared reversible, but pigmented histiocytes were still observed in males and females previously treated at 30 or 2x30 mg/kg/day after 9 weeks of treatment-free period. These findings were considered non adverse.

Hence the NOAEL was set at 15 mg/kg/day corresponding to an AUC_{0-24h} of 1159 ng/mL*h at PND70 and to a C_{max} value of 395 ng/mL. When compared to exposures of BF2.649 in humans at therapeutic dose (AUC_{0-24h} of 804 ng/mL*h and C_{max} of 72 ng/mL) these values indicate safety margins of 1.4 and 5.5, respectively. The total AUC_{0-24h} of the two major active entities (BF2.649 and its metabolite BP1.2526 abundant in rats) at PND70 is 1806 ng/mL*h and corresponding C_{max} value is 747 ng/mL. When these values are compared to pharmacotherapeutic level (AUC_{0-24h} of 832 ng/mL*h and C_{max} of 74,4 ng/mL) safety margins of 2., and 10, respectively, are obtained.

Regarding the main non-conjugated metabolite in human (BP2.951), safety margin ratio calculations provided values of 1.3, higher than 1.3, of 24 and 0.4 when comparing corresponding AUCs in rats (fertility and early embryonic development and pre and post-natal development), in rat embryofoetal toxicity, in rabbit embryofoetal toxicity and in rat juvenile toxicity at NOAEL doses, respectively, to its AUC in humans at therapeutic dose.

Regarding a main metabolite in human (BP1.8054, a glycine conjugate) which was not present in embryotoxicity species receiving BF2.649, a specific *embryotoxicity study in*

rats performed on BP1.8054 provided a NOAEL dose at the highest oral dose tested (300 mg/kg/day). Safety margin ratio calculations provided values of at least 270 when comparing corresponding AUC at the NOAEL dose to its AUC in humans at therapeutic dose.

No drug abuse liability as well as no dependence potential was evidenced for BF2.649 in several *in vivo* models in rodents and monkeys.

3.1.5. Conclusion

Pharmacological studies have confirmed the potential interest of pitolisant to be tested in excessive diurnal sleepiness in narcolepsy, Parkinson's disease, OSA, and dementia with Lewy's bodies. Safety pharmacology performed on vital functions did not show any potential major adverse events on CNS, cardiovascular and respiratory functions.

Exposure in animals showed that pitolisant is well absorbed orally and largely distributed in its target organ, the brain.

The main metabolites and metabolism pathways have been identified. None of these main metabolites were shown to be active.

3.2. Summary of relevant clinical studies

A relatively large diversity of CNS applications were initially explored taking into account the effects of pitolisant on animal models and the fact that no other agent of its pharmacological class had been tested clinically previously. Accordingly were performed pilot trials in epilepsy, ADHD, schizophrenia, dementia as well as in pathologies characterized by EDS (narcolepsy, OSA, Parkinson's disease).

In most trials progressive individual titration was adopted, as is the case for other narcolepsy medications, to adapt to individual sensitivity of patients to the waking (and insomnia-eliciting) effects of the drug.

3.2.1. Phase I studies

A total of 14 pharmacokinetics studies have been conducted and included a total of 225 adult subjects (23 females) and 14 patients suffering from photosensitive epilepsy.

In all studies, serum and urine pitolisant and BP2.951 (its major inactive phase I metabolite) were assayed by using mainly a validated HPLC/MS/MS technique (LOQ: 1 ng/ml).

In addition, BP1.8054 and BP1.9733 (another major inactive metabolites (phase II): glycine and glucuronide conjugate of pitolisant phase I metabolite) were quantified in adult (young, elderly, impaired renal and hepatic patients) and children serum samples from various clinical studies. To this aim, bioanalytical methods were developed and validated according to guideline EMEA/CMPH/EWP/192217/2009 Rev.1.

As no suitable intravenous formulation of pitolisant was available for human use, all these studies were conducted with pitolisant tablets taken orally in a once-a-day manner. Single oral doses from 1 to 240 mg were evaluated in healthy volunteers as well as 9 and 28 days repeated dosing with 40 to 50 mg daily. Metabolism and elimination of pitolisant were assessed through a mass balance study with a single 20 mg radio-labelled pitolisant (^{14}Carbon) administration. Results of this study should be interpreted taking into account *in vitro* incubation of pitolisant with liver microsomes and hepatocytes from rat, dog, monkey and human with potential metabolites identified using LC/MS/ESI^{+}. Effect of age, renal and hepatic impairment on pitolisant PK profile was assessed. Interactions with food, grapefruit juice, itraconazole, paroxetine, rifampicine and olanzapine were evaluated. Protein binding was assessed *in vitro* and plasma/blood partition was assessed both in vitro and ex vivo.

Overall, this programme provided a broad identification of pitolisant pharmacokinetic profile after oral administration in adults. No i.v. formulation has been developed for human use, absolute bioavailability and extent of first pass effect are not accurately known, although the Mass Balance study indicates at least 88% oral bioavailability.

Available data show that pitolisant is well and rapidly absorbed by oral route. It is extensively metabolized by the CYP450 system (pitolisant is a substrate for CYP3A4 and CYP2D6) and conjugation enzymes into numerous inactive metabolites mainly excreted in urine. Pitolisant is not inducer of these catabolic systems at therapeutic concentrations. Inhibitors and activators of CYP450 system have a modest impact on pitolisant PK profile. Pitolisant is bound to around 90% to plasmatic proteins, distributes equally between plasma and blood cells and its apparent volume of distribution is rather large.

Pitolisant apparent terminal elimination half-life ranges between 10 to 12 hours and is dose independent at doses up to 40 mg oad, suggesting that single administration in the morning at adequate dosage is enough to ensure a full day coverage without high levels at night. A population PK analysis of pooled data of healthy subjects (male and female, young and elderly) was performed and showed that i) pitolisant could be modelled according to a bi-compartmental

model with a 0-order absorption, ii) pitolisant $t_{1/2}$ was within a range centered on 10-11 h. This means that, considering a once daily administration, steady-state should be reached within less than one week; and iii) CL/F was found to decrease with both pitolisant dose and duration of administration. In other words wake promotion during day may not compromise nocturnal sleep. Old age has a mild effect on pitolisant PK profile whereas moderate hepatic impairment decreases pitolisant catabolism. The consequences of renal impairment appear to be less substantial. In rats, pitolisant crosses the placenta barrier and is excreted in milk (Study QBR117544 BIP/06).

As evaluated in humans, pitolisant pharmacodynamic profile is on line with what has been reported in preclinical studies. It improves vigilance status and decreases propensity to fall asleep and improves attention. It is devoid of any amphetamine-like psychostimulant potential and of any drug abuse potential.

A population PK study was developed and is the support for determination of optimized PK blood sampling times and the sample size required for a population PK analysis planned in the present study.

3.2.2. Phase II Studies

3.2.2.1. Brief Summary

Nine phases IIa proof of concept studies are completed:

- One in photosensitive epileptic patients (Study P03-06)
- One in Obstructive Sleep Apnea Syndrome patients (Studies P04-01 and P05-01)
- Two in narcolepsy (Studies P05-03 and P06-06)
- One in Obstructive Sleep Apnea Syndrome patients (Study P05-01)
- One in Parkinson disease-treated patients (Study P05-05)
- One in Attention Deficit with Hyperactivity Disorder (ADHD) patients (Study P05-07)
- One in pharmaco-resistant epileptic patients (Study P04-07)
- One in hospitalized schizophrenic patients (Study P04-08) is discontinued
- One in Lewy's bodies dementia (LBD) patients (Study P05-08)

The designs of these studies are detailed in the Investigator's Brochure.

The studies of pitolisant in daytime sleepiness in various conditions are summarized as

follows

Results obtained in 22 narcoleptic patients (P05-03) showed that pitolisant was effective in reducing the excessive diurnal somnolence of narcoleptic patients at the dose of 40 mg/day compared to placebo. The reduction of somnolence was of 4.9 points on the ESS score which is equivalent to the results obtained with psychostimulants such as modafinil. A level of normal daytime sleepiness was reported by 40% of patients, this result was confirmed by the other criteria of sleepiness evaluated on the patient's diary. The treatment was well tolerated. The main adverse events reported were moderate in intensity and did not lead to stop the treatment. The most frequent were headache, nausea and insomnia. The occurrence of these adverse events seems to be related to high plasmatic levels.

In another study (P06-06) to confirm that pitolisant was effective to reduce excessive daytime sleepiness in narcoleptic patients, the improvement of wakefulness measured by ESS indicated a reduction of 4.8 points. This value was equivalent to the results obtained in P05-03, and was also similar to that published with Modafinil and Sodium oxybate. In this study, the exposure of 26 patients with individual titration from 10 mg to 40 mg was well tolerated.

Out of the 26 patients enrolled in the trial and who completed the first 28-day period of pitolisant administration, 18 patients, entered in the 3-month extension period and completed the 3-month treatment period. Either, 11 of the 18 patients entered in the 9-month extension period, 10 of them completed the 9-month treatment.

Results obtained in 25 patients with Parkinson's disease (P05-05) showed a marked reduction of daytime sleepiness with pitolisant as compared to the placebo period. A tendency to reduce the motor manifestation was also noticed. The tolerance was good.

One Double Blind period IIb clinical study (P07-02) dose-ranging study in Parkinson's disease was completed in 107 patients in France and Germany. This study included 5 groups of treatment: Placebo, and pitolisant: 5-10-20-40 mg/day for 28 days. The main results on the main criterion showed that the effective dose was the dosage 20 mg ($p = 0.0357$). The tolerance of the product was good and comparable between the different groups of patients.

Based on these results, the 20 mg pitolisant dose was chosen as the significant effective dose for the phase III development program on EDS in PD patients.

3.2.2.2. Two pilot studies referring to the Sleep Apnea Syndrome

3.2.2.2.1. Single blind, controlled versus placebo study of pitolisant in patients suffering from Obstructive Sleep Apnea Syndrome with daytime somnolence (P04-01 pitolisant) (P. Levy – Grenoble)

Twelve patients with confirmed diagnosis of OSA by PSG were included in this multicenter study (3 centres in France). The design of the study was single blinded comparative versus placebo. Patients received placebo during the 2 first days, then a 3-day treatment of pitolisant at the dose of 40 mg/day followed by a 2-day observation period with a placebo-treatment.

The efficacy of pitolisant on sleepiness and sleep was evaluated by both sleep diary and Epworth sleep scale.

The number of diurnal sleepiness episodes (DSE) was evaluated following each period. The mean absolute change before and after pitolisant treatment corresponded to 38% relative improvement.

Among the 12 patients included in the study, 8 reported some diurnal sleeps on their sleep diaries before treatment. The effect of pitolisant allowed suppressing all sleep needs in these 8 OSA patients.

The mean value of the Epworth Sleepiness Scale (max score 24) showed an improvement in the absolute change of -5.8 ± 4.4 between before and after treatment, corresponding to 38% improvement from baseline value. It is interesting to note that all patients were improved, 5 patients rating the scale less than 8 after pitolisant treatment (threshold considered as normal).

Effect of pitolisant on Epworth score (Study P04-01)

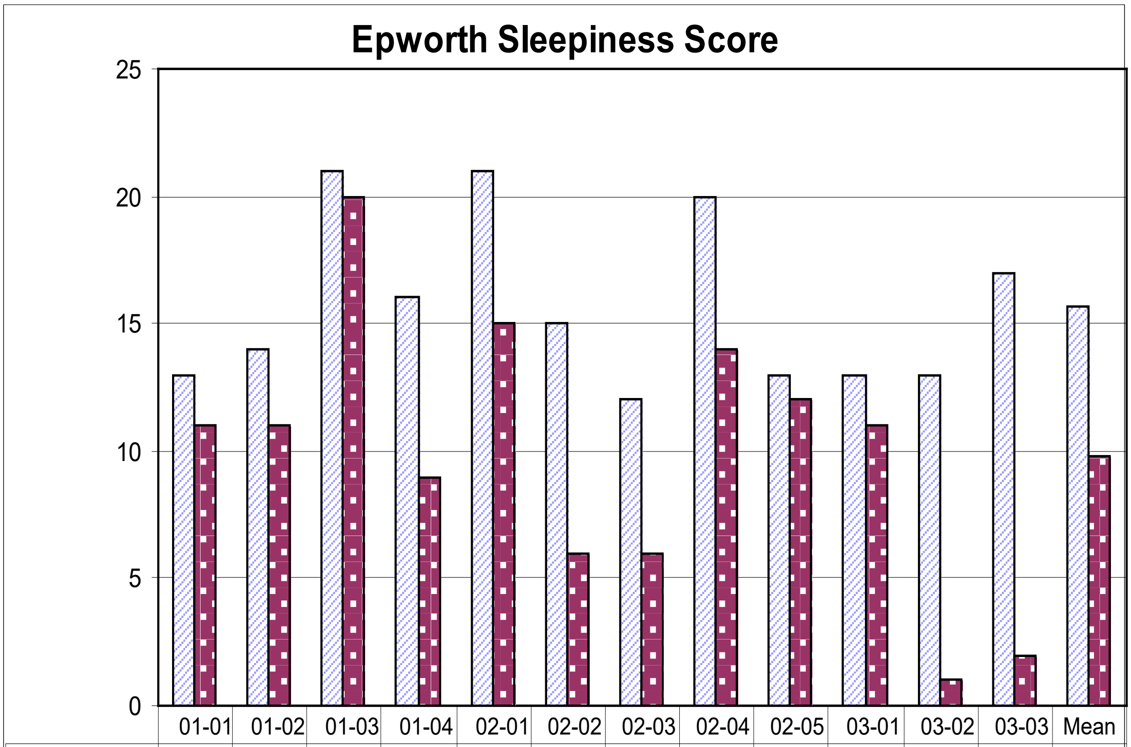

	01-01	01-02	01-03	01-04	02-01	02-02	02-03	02-04	02-05	03-01	03-02	03-03	Mean
Epworth BL	13	14	21	16	21	15	12	20	13	13	13	17	15,7
Epworth Final	11	11	20	9	15	6	6	14	12	11	1	2	9,8

Discussion:

In this study, 3 days of 40 mg of pitolisant oral treatment seems to improve the sleepiness of the OSA patients as measured by the Epworth score, and the daily patient diary.

The blood level of pitolisant was measured in each patient. Regression analysis between pitolisant level and the efficacy assessed either by mean change in DSE or in ESS, showed a positive correlation.

The efficacy was better when the level of pitolisant was higher. However, considering the small number of subjects in this analysis, it was not possible to draw any firm conclusion at this stage of development.

The tolerance to pitolisant was good. During the study, no serious adverse events were reported, 4 patients reported a total of 9 adverse events (rhino sinusitis, weight gain, viral rhinitis, thoracic oppression sensation, yawning).

Their intensity was rated as mild and treatment was not discontinued. Five patients out of 12 reported insomnia during the night following the first intake of 40 mg of pitolisant.

Nevertheless, this undesirable effect was not described as disturbing by the patients.

Furthermore this effect tended to fade away with the treatment continuation.

Conclusion

Overall, this first study according to a single blind design shows that a 3 day-treatment by pitolisant at a dose of 40 mg par day is able to improve ESS score, to decrease the number of DSE, and to suppress diurnal sleep need in patients suffering from a severe OSA. Globally, the tolerance profile of pitolisant was good.

3.2.2.2.2. Effect of pitolisant on vigilance and polysomnographic parameters in patients with Obstructive Sleep Apnea Syndrome (P05-01 BF2.649)

Since the first study in this indication (P04-01) showed preliminary good results on sleepiness improvement in OSA patients, this second study was designed to evaluate quantitatively the effect of pitolisant using the OSleR test (Oxford Sleep Resistance test, modified maintenance of wakefulness test), and the Epworth Sleepiness Scale (ESS) in patients with moderate to severe OSA, eligible but not yet treated with nasal continuous positive air pressure (nCPAP). The effect of pitolisant was also assessed on nocturnal polysomnography (PSG), where respiratory and sleep events are recorded. This study was single blind with two treatment sequences, the patients receiving placebo during one week, then pitolisant (40 mg/d) during the second week. The evaluations (ESS, OSleR test and PSG) were performed at baseline, and at the end of each treatment week.

Results

From 21 recruited patients, 20 reached the normal end of the trial (2 weeks). The characteristics are: Mean age: 51,33 years [25-68], Body Mass Index (BMI): 30,24 (SD=3,83 [22, 36]), OSA Mean duration: 8,5 years (SD=9,15 [1, 38]), AHI: 54,95 (SD=21,52 [20, 91]).

Regarding the main endpoint:

(1) OSleR test: the mean values of OSL_0 (before placebo), OSL_1 (after placebo week) and OSL_f (after pitolisant treatment week), the mean changes OSL_f-OSL_1 and OSL_f-OSL_0 highlight a significant improvement after pitolisant treatment (see the following table). By considering the end of the placebo period as the reference baseline value, a mean improvement of 6,79 (95%CI= [1,90, 11,68] was observed ($p < 0,01$).

	Mean	95%CI	Count
OSL_0	31,43	[26,49, 36,36]	(21)
OSL_1	32,38	[26,61, 38,16]	(21)
OSL_b	31,90	[26,85, 36,96]	(21)

OSL_f	39	[37,72, 40,28]	(19)
OSL_f-OSL_1	5,89	[0,37, 11,42]	(19)
OSL_f-OSL_0	7,68	[2,79, 12,58]	(19)
OSL_f-OSL_b	6,79	[1,90, 11,68]	(19)

(2) Epworth Sleepiness Scale (ESS): A significant mean change (ESS_f –ESS_b) was found between the end of pitolisant treatment (ESS_f) and the baseline pooled value (ESS_b) of -4,36, 95 % CI= [-5,86, -2,85], p < 0,05. The other mean changes based on ESS_0 or ESS_1 separately provide the same results leading to the same conclusion. Therefore, a significant decrease of the ESS has been demonstrated with pitolisant treatment.

	Mean	95%CI	Count
ESS0	14,14	[13,03, 15,25]	(21)
ESS1	11,05	[9,11, 12,98]	(21)
ESSf	8,24	[5,98, 10,49]	(21)
ESSb	12,60	[11,21, 13,99]	(21)
ESS1-ESS0	-3,10	[-4,58, -1,61]	(21)
ESSf-ESS0	-5,90	[-7,57, -4,24]	(21)
ESSf-ESS1	-2,81	[-4,50, -1,12]	(21)
ESSf-ESSb	-4,36	[-5,86, -2,85]	(21)

As to polysomnography (PSG), for each parameter, the mean difference between the end of pitolisant treatment and the end of placebo treatment has been analyzed. Two parameters, awakening intra sleep, and the percentage of the total period of sleep were characterized by a significant change.

Regarding the secondary endpoint based on patient sleep diary, the number of EDS episodes and the total number of EDS + diurnal sleep episodes extracted from the sleep diary were significantly decreased between before and after pitolisant treatment.

The safety profile was very good. No serious adverse events were observed. No patient required pitolisant treatment to be discontinued.

The relation to the study product was well ascertained, and considered as being *plausible* by the physician for three patients: obs. n°203 (nausea), obs. n°201 (epigastralgia), and obs. n°202 (insomnia) during the pitolisant period, that did not lead to stop the treatment.

Laboratory workup and electrocardiograph results showed no pathological changes.

From these findings, it could be ascertained that pitolisant provide reasonable assurance of a

good safety profile.

Conclusion

During this single blind study in obstructive sleep apnea (OSA), a significant improvement of excessive diurnal sleepiness was noted.

This improvement was demonstrated on all retained criteria, compared before and after pitolisant treatment, more particularly on the primary criterion assessed by the OSleR test as well as on ESS, patient's diary, and intra sleep awakening (polysomnography).

Pitolisant daily, orally administered during 7 days at 40 mg was safe and well tolerated. No serious adverse event was reported in 21 patients. Globally, the safety profile was very good.

3.2.2.3. Minimum effective dose-finding study of BF2.649, in patients with moderate to severe Obstructive Sleep Apnea, experiencing Excessive Daytime Sleepiness (EDS) despite regular use of nCPAP, and patients having refused this therapy. Randomized, double blind study with BF2.649 (5-, 10-, 20-, 40- mg/d), or placebo (P09-16 BF2.649)

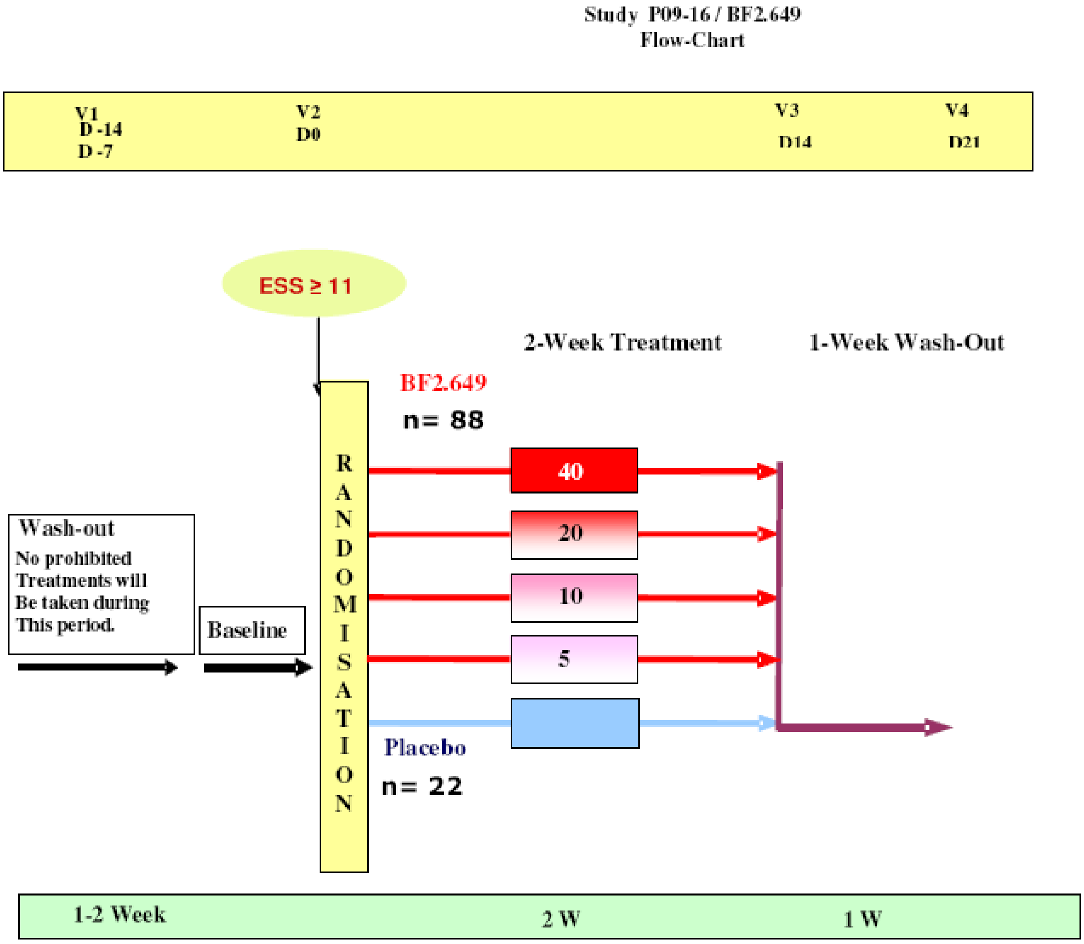

Overall, 116 patients were included and 110 completed the study. One randomized patient was

not taken into account for the efficacy intent-to-treat analysis as he did not take any study treatment.

BF2.649 given at doses of 5, 10, 20, or 40 mg OD decreased daytime sleepiness assessed with ESS score and the effect statistically significantly increased with the dose (p=0.0003, Step-down Type 2 Linear Contrasts). The increase was linear up to the 20 mg OD dose. The minimal effective dose of BF2.649 on ESS score (primary endpoint), as well as ESS responders rate, DSAR and CGI-C (secondary endpoints) was 20 mg OD. The 40 mg OD dose did not seem to have any additional effect on most endpoints. BF2.649 was well tolerated in this population of patients with Obstructive Sleep Apnea and Excessive Daytime Sleepiness.

3.2.3. Phase III studies

To follow up on the good results in OSA provided by phase II trials, two phase III studies were designed to assess the efficacy and safety of pitolisant at doses up to 20 mg OD in comparison with placebo during 12 weeks for patients who experience residual sleepiness despite regular nasal Continuous Positive Airway Pressure (nCPAP).

The main endpoint of these studies was to assess the efficacy on excessive daytime sleepiness (based on the score of the Epworth Sleepiness Scale, ESS) and safety of BF2.649 given at 5, 10, or 20 mg per day *versus* placebo. The EDS evaluations (ESS and OSleR test) were performed at baseline, and at the end of the double blind period.

At end of the double blind period the patients could enter an open label period and received pitolisant up to 20mg OD for 40 additional weeks to assess pitolisant efficacy maintenance and safety.

Double blind flow-Chart

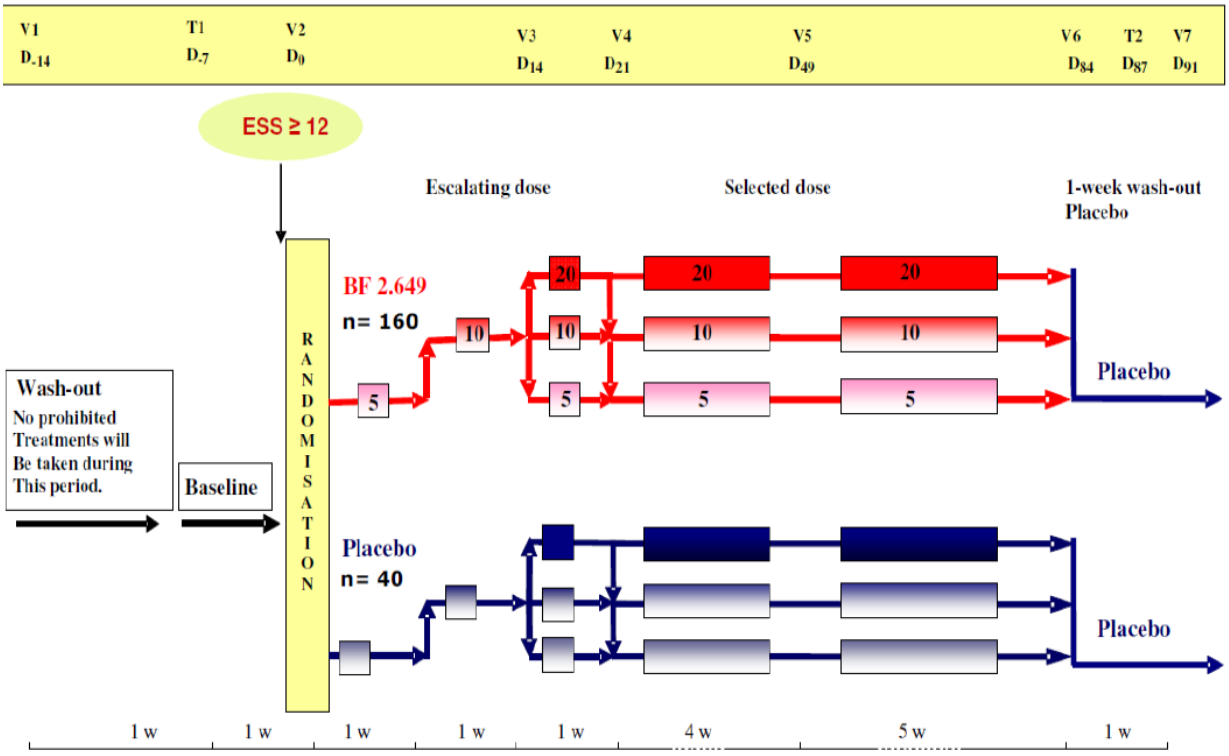

Main Endpoint:

The ESS mean value was compared between the treatment groups at every visit (Mean ± SD, (SE). Baseline ESSb and final ESSf values were calculated as the mean of ESS at visit 1+2 and 5+6, respectively.

Secondary endpoints:

- Percentage of ESS treatment responders;
- Severity of Excessive Daytime Sleepiness (EDS) as measured by the patient sleep diary at V2 and V6;
- Oxford Sleep Resistance Test (OSL) at V2 and V6;
- European Quality of life questionnaire (EQ-5D) at V2 and V6;
- Leeds Sleep Evaluation Questionnaire (LSEQ) at V2 and V6;
- Cognitive Function Test: Trail Making Test (TMT) Parts A & B;
- Illness severity as measured by the Clinical Global Impression of Severity and of Change (CGI-S and CGI-C) at V2 and V6;
- Beck Depression Inventory (BDI) at V2 and V6;
- Pichot Fatigue Scale (PSF) at V2 and V6.

3.2.3.1. P09-08 Harosa 1 preliminary results

244 moderate to severe patients using nCPAP and still complaining of EDS were recruited and randomized on pitolisant (n=183) or placebo (n=61) and constitutes the Full Analysis Set (FAS) population and Extended to Treat (EIT) population. The EIT population and Safety population analyzable samples were identical and constituted 244 patients. The Intent to Treat (IT) was n=243, where one patient (4218) under the pitolisant arm was excluded for not having an ESS value at baseline (V2). Thirty-six patients were found with a major deviation (7 placebo, 29 pitolisant; refer to Table 1.1.3 for reason). The Per Protocol (PP) sample is constituted by 200 patients (52 placebo, 148 pitolsant). Forty-four patients were excluded from the PP (refer to Table 1.1.3 for explanations). Twelve patients (3 placebo, 9 pitolisant) had a premature termination of the trial.

Pitolisant, in moderate to severely affected OSA patients using nCPAP and still complaining of EDS, showed a statistically significant improvement in decreasing ESS score when compared to placebo. The study preliminary results also confirmed pitolisant efficacy on other EDS criteria such as OSLER test, ESS responders rate and Clinical Global Impression. Pitolisant overall good safety profile was also confirmed in this study.

3.2.3.2. P09-09 Harosa 2 preliminary results

268 patients were recruited and randomized on pitolisant (n=201) or placebo (n=67). One patient (4003) was not treated and withdrew from the study after inclusion, thus the Full Analysis Set (FAS) population and Extended to Treat (EIT) population constitute 267 patients with pitolisant (n=200) and placebo (n=67). The Intent to Treat (IT) population and EIT population and Safety population analyzable samples were identical and constituted 267 patients.

Pitolisant, in moderate to severely affected OSA patients not using nCPAP and complaining of EDS, showed a statistically significant improvement in decreasing ESS score when compared to placebo. The study preliminary results also confirmed pitolisant efficacy on other EDS criteria such as ESS responders rate and Clinical Global Impression. Pitolisant overall good safety profile was also confirmed in this study.

3.2.4. Summary of known and potential benefits and risks

The symptoms of Excessive Daytime Sleepiness resulting from the untreated sleep apnea are thought to have serious consequences during activities where alertness is dangerous such as driving, leading to an increased risk of road traffic accidents. The EDS in sleep apnea patients is also associated with a reduction in quality of life and an impairment of cognitive function.

The study medication, pitolisant (BF2.649), is a highly potent and selective antagonist and inverse agonist of histaminergic receptor type 3 (H_3R). Pitolisant was found to enhance the histaminergic transmissions in the brain and thereby to improve vigilance and alertness.

The exposure to pitolisant in healthy volunteers was assessed in six pharmacokinetic studies in which 66 subjects received single oral dose of pitolisant from 5 to 120 mg and during 28 days with 50 mg once daily. The compound was well tolerated up to oral single dose of 90 mg, however the dose of 120 mg led to irritability. The repeated administration (28 days up to 50 mg/day) was well tolerated. Pitolisant appears clearly as a once – a – day drug with a plasma half-life between 10 and 12 hours. Upon repeated administration, the steady state of pitolisant is achieved after 5-6 days. The elimination is mainly achieved through an inactive metabolite (BP2.951) that is excreted in the urine

The beneficial effect of pitolisant in Excessive Daytime Sleepiness associated with Obstructive Sleep Apnea syndrome has already been demonstrated in two proof of concept clinical studies, in which 33 patients participated. In one study in which 22 patients were involved, pitolisant given during 7 days reduced significantly the score of the Epworth Sleepiness Scale (ESS) by more than 4 points, when compared with baseline score. Because the reduction of 3 points is considered as an indication of clinically significant response in several studies encountered in literature, the reduction of more than 4 points provided by treatment with pitolisant is clinically meaningful for OSA patients. These results were confirmed in two phase III studies including moderate to severe OSA patients receiving nCPAP (P09-08) or without nCPAP (P09-09) treated for 12 weeks with pitolisant 20mg OD or placebo. These trials included 244 (P09-08) and 267 (P09-09) patients. The ESS score was reduced in the pitolisant group of 6 points compared to baseline score in both studies and was statistically significant when compared to the placebo group.

The other benefits provided by pitolisant treatment resulted from the analysis of data collected with the daily sleep diary, which showed a tendency to suppress diurnal sleep and reduce the sleepiness of the evaluated patients. OSA patients suffering from EDS improved significantly

about alertness as confirmed by the OSleR test (Oxford Sleep Resistance test, modified version of maintenance of wakefulness test), thus this finding prove that pitolisant could be relevant for the OSA patients to improve the vigilance mostly involved in traffic road accidents.

Until now, the safety profile of this product was found to be very satisfactory in the patients treated for excessive daytime sleepiness associated to Obstructive Sleep Apnea, or to a Parkinson's disease or to narcolepsy. Pitolisant is currently in its development phase in OSA. As a consequence all the adverse events are not known.

However, during the previous studies, which enrolled approximately 1094 patients, the most frequent adverse drug reactions (ADRs) reported with pitolisant were insomnia (8.4%), headache (7.7%), nausea (4.8%), anxiety (2.1%), irritability (1.8%), dizziness (1.4%), depression (1.3%), tremor (1.2%), sleep disorders (1.1%), fatigue (1.1%), vomiting (1.0%), vertigo (1.0%), dyspepsia (1.0%), weight increase (0.9%), abdominal pain upper (0.9%). These effects were all transient and most declined spontaneously when the treatment was stopped. No additional significant side effect associated with pitolisant over the long term was observed.

Regarding the mode of administration planned for this study (one intake in the morning) and clinical relevant benefit despite some adverse drug reactions encountered during the previous studies, the benefit risk ratio could be considered as advantageous for use of pitolisant. According to the tolerance of the product evaluated in the completed studies, it is recommended to start the treatment of EDS with an escalating dose regimen to optimize the tolerance up to the stabilized dose.

4. RATIONALE OF THE CURRENT STUDY

Excessive daytime sleepiness (EDS) is a major complaint in patients with obstructive sleep apnea syndrome (OSA) defined as sleep-related breathing disorder (SRBD) with full or partial occlusion of the upper airway during the sleep. OSA afflicts at least 2-4% of the adult population (at least 4% of males and 2% of females)[1,2].

The proposed mechanism for EDS in OSA patients is sleep disturbance and loss of sleep resulting from microarousals produced by increased ventilatory effort[3,4].

Patients may be personally unaware of their snoring and breathing pauses during sleep, but the consequences of daytime hypersomnolence such as deficit in attention or vigilance (road accident, accident at work)[5], impaired short-term memory and reduced capacity to sustain concentration or focus are often essential to rational clinical decision about whom to treat [6] and

the American Academy of Sleep Medecine recommends polysomnography to diagnose and to determine the severity of obstructive sleep apnea.

Nasal continuous positive airway pressure (nCPAP) is the treatment of choice for most patients with OSA[7,8,9,10]. When used properly, nCPAP reduces apnea and hypopnoea rate, often normalizes arterial blood oxygen saturation, decreases sleep fragmentation, and improved sleep quality. As a result, alertness, mood, cognitive function, and quality of life improve[11]. Furthermore nCPAP reduces risks of cardiovascular events in obstructive sleep apnea patients.

However not all patients find CPAP as the ideal treatment. Like all chronic therapies, compliance with CPAP is difficult to maintain over and between 5% and 50% of patients refuse CPAP therapy initially, and another 12% to 25% may discontinue CPAP within 3 years (Akram Khan Sleep review , April 2008).

In a long term study of the use of nCPAP in 137 sleep apnea patients during 8 years, Bizieux-Thaminy *et al* outlined that among 30 patients out of 137 (22%) who stopped nCPAP, 10% of them stopped nCPAP within the 6 first months, 30% within the year, and 67% within 3 years.

The most common problem rated as severe was nasal stuffiness, sensation of cold air, noise and mask pressure. CPAP therapy correlated negatively with CPAP use, indicating that these were problems that the patients blamed for a lack of CPAP use[12].

Patients with obstructive sleep apnea syndrome can experience residual excessive daytime sleepiness despite regular use of nasal Continuous Positive Airway Pressure therapy. As 230,000 obstructive sleep apnea patients are currently treated in France by CPAP, more than 13,800 of them might suffer from residual excessive sleepiness [*Pepin J. L, Viot-Blanc V, Escourrou P, Racineux J-L, Sapene M, Levy P, Dervaux B, Lenne X, Mallart A, Prevalence of residual excessive sleepiness in CPAP-treated sleep apnea patients: The French multicentre study. Eur Resp J 2009; 33: 1062-1067*]. These patients, who often complain of an important EDS which represents a serious embarrassment in their everyday life, also claim a symptomatic treatment.

In view of these issues, alternative modality such as pharmacologic symptomatic treatment may be of great meaning to treat persistent residual excessive daytime sleepiness, which leads to a decreased quality of life. It is within this framework that pitolisant, novel selective histamine H_3 receptor antagonist/agonist inverse which exhibits strong waking promoting effects and a good tolerance in a number of pre-clinical and clinical studies, appeared completely indicated.

Experimental evidence for enhanced wakefulness induced by H_3R blockade was previously

reported with thioperamide, the prototypic H_3R antagonist [*Lin et al., Brain Res. 1990, 523: 325-30*]. Similar vigilance - and attention-promoting effects were described more recently with the antagonist ciproxifan [*Ligneau et al., J. Pharmacol. Exp. Ther. 1998, 287: 658-66*].

Improvement of learning deficit and memory by an H_3 antagonist was also demonstrated experimentally in mice [*Miyazaki et al., Life Sci. 1995, 57: 2137-44; Meguro et al., Pharmacol. Biochem. Behav. 1995, 50: 321-5; Onodera et al., Naunyn-Schmiedeberg's Arch. Pharmacol. 1998, 357: 508-13*]. The effects of pitolisant on sleep/wakefulness, and EEG patterns have been investigated in cats and mice. It has been shown that pitolisant increased markedly the duration of waking in these two animal species. Moreover, the EEG results suggest that pitolisant could improve the quality of arousal, by reinforcing e.g. the level of vigilance and attention, in the absence of any noticeable psychomotor activation that is observed with drugs like amphetamines.

In healthy volunteers, the clinical and biological tolerance of pitolisant was excellent with single oral dose between 1 mg and 90 mg.

Pitolisant was shown to produce an increase in vigilance on critical Flicker Fusion Threshold, and on spectral EEG analysis: increase of rapid rhythms (beta frequencies > 13 Hz) with concomitant decrease of alpha and theta frequencies.

The previous phase II and III clinical studies (P04-01, P05-01, P09-16, P09-08, and P09-09) have demonstrated that pitolisant at doses up to 20mg OD significantly decreases the diurnal somnolence. It seems the effect increases with dose. Therefore, our study will assess in patient, treated with nCPAP or not, pitolisant effect at doses up to 40mg OD.

ESS measures sleepiness recently *[Johns MW Sleep 1992]*. It is a simple self administered questionnaire with eight items which is shown to provide a measurement of the subject's general level of daytime sleepiness in several real life situations. It scores the tendency to fall asleep (from 0-3) during eight everyday situations. It scores from 0-24, and abnormal somnolence is considered as a value greater than 10. ESS has proven a very satisfying reliability, and apparently the best sensitivity and specificity among all the other instruments, even those developed in this specific pathology. A final important characteristic of this instrument is its reproducibility with naturalistic conditions.

The primary endpoint of this study will be measured by the change in Epworth Sleepiness Scale. Its reliability and internal consistency have been demonstrated.

Furthermore, because of the subjectivity of ESS, a secondary criterion will support it with

quantitative data related to the diurnal sleepiness: the home patients' diaries.. These diaries will be filled during 3 sequential days of the week preceding each visit and will record the number and duration of sleep and sleepiness episodes. By that mean the EDS change measured by ESS will be confirmed by quantitative measurements of sleepiness.

The main criteria of selection for the OSA population will be: ESS ≥12

The treatment regimen for this study will start at V2 with an escalating dose starting at 10 mg OD pitolisant or placebo on the first week followed by 20 mg OD on the second week. At V3, the posology is increased to 40 mg/d if the tolerance is acceptable. If the study product is not well tolerated (i.e. troublesome insomnia), the patient will continue taking 20 mg/d pitolisant or placebo, or may be the posology will be eventually reduced at 10 mg/d until V4. At V4, the investigator will decide to maintain, or to reduce the dose according to the tolerance of the study drug. No increase of the dose will be allowed at V4.

Then the adjusted posology will remain stable during the following 9 weeks of the double blind period.

The effects of pitolisant will be compared to those of the placebo by means of a superiority analysis.

5. STUDY OBJECTIVES

The first objective of this study is to demonstrate the efficacy and safety of pitolisant given at 10, 20, or 40 mg per day versus placebo during 12 weeks for the Double Blind period, to treat the EDS in patients with OSA refusing the nCPAP therapy or treated by nCPAP but still complaining of EDS. The efficacy of pitolisant will be assessed separately in patients treated with nCPAP and in patients without nCPAP use.

The secondary objectives of the study include assessing the long-term tolerance as well as the maintenance of efficacy of pitolisant given at 10, 20 or 40 mg per day during 39 weeks of Open Label Extension period and further investigating the co-variates or co-medications that affect the pharmacokinetics of pitolisant in the target population to allow future comparison to healthy subjects.

6. POPULATION

The study will be performed in patients experiencing EDS as a result of moderate (15-30 sleep obstructive related breathing events per hour of sleep) to severe (more than 30 sleep obstructive related breathing events per hour of sleep) OSA diagnosed by polysomnography, without important cardiovascular risks, and having refused the nCPAP therapy. Also patients having been submitted to nCPAP therapy for a minimum period of 3 months, trying to adapt the latter at its best, and still complaining of EDS will be included.

Patients refusing to be treated by nCPAP therapy will be asked before the beginning of the Open Label Extension period (at V7) and then at V8, V9, V10, V11 taking into account their cardiovascular profile, whether they want to reconsider their opinion about use of nCPAP, and they will be given the opportunity to withdraw from the study if they accept to use nCPAP therapy.

6.1. Inclusion Criteria

All subjects included in the study will have to meet all of the following criteria for inclusion in the study:

- Male and/or female outpatients aged from at least 18 years
- Patients complaining of EDS refusing to be treated by nCPAP therapy or having been submitted to nCPAP therapy for a minimum period of 3 months, and still complaining of EDS despite the efforts made beforehand to obtain an efficient nCPAP therapy
- Polysomnography performed (for patients submitted to nCPAP therapy – under nCPAP) between V1 and V2 or during the last 12 months with Apnea-Hypopnea Index (AHI): for patients without nCPAP therapy ≥ 15; for patients under nCPAP therapy ≤ 10
- For patients submitted to nCPAP therapy: nCPAP ≥ 4 hours / day (compliance checked on the clock-time counter of the CPAP machine)
- Mini Mental State Examination (MMSE) ≥ 28
- Beck Depression Inventory – 13 items (BDI-13) score < 16 and item G (suicidal ideation) of BDI-13 = 0
- Body Mass Index (BMI) ≤ 40 kg/m²
- Epworth Sleepiness Scale (ESS) ≥ 12

- Female patients with child-bearing potential using a medically accepted method of birth control (i.e. oral contraceptives of normal average dosage) agreeing to continue this method throughout the study, and during the month following treatment discontinuation, being negative to serum pregnancy test performed at the screening visit
- If specified by the investigator, the patient must be willing not to operate a car (if sleepy at wheel) or heavy machinery for the duration of the trial or as long as the investigator deems it clinically indicated. In addition, the patient should be willing to maintain during the study their usual behaviors which could affect their diurnal sleepiness (e.g. circadian rhythm, caffeine consumption, nocturnal sleep duration)
- Patients having signed and dated the informed consent form

6.2. Non-Inclusion Criteria

All subjects included in the study must not meet any of the following non-inclusion criteria:

- Patients having previously been exposed to pitolisant either in previous clinical trials, or in compassionate program or being prescribed the commercial form (Wakix®), for those enrolled patients from January 2018
- Patients suffering from chronic severe insomnia in accordance with the International Classification of Sleep Disorders (ICSD 2005) without OSA
- Patients with co-existing narcolepsy (ICSD 2005), judged on clinical criteria
- Patients with sleep debt not due to OSA (according to the physician' s judgment)
- Patients with non-respiratory sleep fragmentation (restless leg syndrome…)
- Shift work, professional drivers
- Refusal from the patient to stop any current therapy for EDS or predictable risk for the patient to stop the therapy
- Patients suffering from a psychiatric disease
- Acute or chronic disease preventing the improvement assessment, e.g. severe chronic obstructive pulmonary disease (COPD)
- Current or recent (within one year) history of drug, alcohol, narcotic or other substance abuse or dependence
- Any significant serious abnormality of the cardiovascular system, e.g. recent myocardial

infarction, angina, hypertension or dysrhythmias (within the previous 6 months), Electrocardiogram Fridericia corrected QT interval higher than 450 ms, history of left ventricular hypertrophy or mitral valve prolapse

- Severe co-morbid medical or biological conditions that may jeopardize study participation at the discretion of the investigator (particularly in the cardiovascular system and the instable diabetes)
- Positive serology tests (HIV, HCV and HBsAg)
- Pregnant or breast-feeding women
- Women with child-bearing potential and no efficient birth-control method
- Patients unable to understand the study protocol
- Patients with suspected or known hypersensitivity to study medication
- Patients with a dominant arm deficiency impeding the achievement of the tests
- Patients using a prohibited medication
- Congenital galactose poisoning, glucose and galactose malabsorption, deficit in lactase
- Patients participating in another study or being in a follow-up period for another study

6.3. Patient completion and early withdrawal of patients from study

A patient will be considered to have completed the study if Visit 7 (end of the Double Blind period) or Visit 13 (Open Label Extension period) has been completed.

A withdrawal patient will be any patient who will not have completed Visit 6 (Double Blind period). In accordance with the Declaration of Helsinki, patients will be free to withdraw from the study at any time if they wish to do so, for any reason specified or unspecified.

Before the end of study visit, the investigator has the responsibility and the right to interrupt a patient's participation. The investigator will have to inform the sponsor or his representative of a patient withdrawal.

The following reasons will be accepted for study discontinuation:

- Patient changing his position towards nCPAP therapy (patient not on nCPAP therapy requiring to be treated by nCPAP; patients on nCPAP therapy refusing to continue it)
- Voluntary withdrawal of patient consent, or loss to follow-up, or inability to remain under medical observation

- Intake of any prohibited treatment
- During the whole study, severe depression indicated by BDI-13 ≥ 16 and suicide risk (BDI-13 item G > 0)
- During the whole study, electrocardiogram Fridericia corrected QT interval higher than 450
- Non-compliance or major deviation from the protocol
- In case of occurrence of a Serious Adverse Event (SAE), or any other situation where, in the opinion of the investigator, continuation of the study would not be of benefit to the patient or would put the patient at risk
- Discontinuation of the study by the investigator
- In the situation where the double-blind is broken and the code for the individual patient is opened.

Should any of the subjects be withdrawn from the study, the sponsor's representative, the investigator and the scientific committee if necessary, will discuss together the possibility of replacement. The reason for withdrawal has to be recorded in the CRF for all withdrawn subjects and in the source document.

7. EXPERIMENTAL DESIGN

This is a prospective, multicenter (in Europe), randomised, Double Blind phase III study versus placebo.

It will be carried out in patients diagnosed as having OSA, without important cardiovascular disease, experiencing EDS, having refused the nCPAP or having been submitted to nCPAP therapy for a minimum period of 3 months and still complaining of EDS. With the exception of spending an optional one night (if not done during the twelve months preceding the study) in the sleep laboratory for the full recording of nocturnal polysomnography between V1 and V2, the patient will be ambulatory during the whole study period. To further characterize the pharmacokinetic profile of pitolisant administered in OSA patients up to 40 mg OD a PK analysis will be performed. This will also provide additional data on co-variates or co-medications that may impact the pharmacokinetics of pitolisant.

After the first wash-out period at the end of the Double Blind period, patients may optionally, and once a new informed consent form being signed, enter the 40-week Open Label Extension

period of the study consisting in an escalating-dose of the same regimen than in the 1st part of the study, followed by a selected dose phase, with the active drug only, until week 53 after the start of the treatment.

The study will include about 400 patients. This number will allow having 360 completed treated patients (pitolisant, patients refusing nCPAP therapy = 120; placebo, patients refusing nCPAP therapy = 60, pitolisant, patients treated by nCPAP = 120; placebo, patients treated by nCPAP = 60) sufficient for the results analysis of efficacy during the Double Blind period (12 weeks).

The timeline of examinations and tests to be performed at each visit are indicated in section 2 (study diagram), concerning the study diagram for the patients withdrawing their participation in the study after the Double Blind period as well as for patients pursuing the Open Label Extension period.

V1 – Screening visit and beginning of initial wash-out period (D -14)

Before proposing the study to patients refusing to be treated by nCPAP, the investigator will ensure that the patient still refuses this treatment.

An appropriately signed informed consent will be obtained prior to entry into the study for each patient having made a positive decision to participate. The investigator must confirm that the patient meets all inclusion criteria and none of the non-inclusion criteria

Patients included in the study shall not take any treatment indicated for EDS or other psychotropic drugs noted in chapter prohibited treatment during the following two weeks before being submitted to baseline examination (with the exception of chronic medications taken to treat pathology, and authorized by the protocol).

Patients will be reminded to contact the investigator between the visits for any issue, such as adverse events, difficulties with the treatment, need for a new treatment, associated pathology, and modification of the current concomitant treatment (applicable for the whole study duration).

Ph1 – Phone contact (D -7)

During this phone contact, the investigator should:

- Check if patient has discontinued prohibited treatment
- Check concomitant treatments
- Check occurrence of AEs

V2 – Inclusion visit and beginning of the Double Blind period: baseline examinations, randomisation, start of escalating dose phase (D 0)

The patients will be randomized to either pitolisant, or placebo.

The treatment will be initiated by an individual titration period over 2 weeks.

1st week: At each morning, with a glass of water during breakfast

Patients on pitolisant will receive

During breakfast	10 mg

Patients on Placebo will receive

During breakfast	Placebo

2nd week: At each morning, with a glass of water during breakfast

Patients on pitolisant will receive

During breakfast	20 mg

Patients on Placebo will receive

During breakfast	placebo

The total treatment period in double-blind is 12 weeks.

V3 – First dose adjustment visit (D 14)

The posology is increased at 40 mg/d pitolisant (high dose) or placebo every morning, during breakfast. If the tolerance does not allow it (occurrence of an adverse event i.e. troublesome insomnia), the patient will continue the treatment intake at 20 mg/d pitolisant (medium dose) or placebo or the posology will be eventually reduced at 10 mg/d pitolisant (low dose) or placebo until V4.

V4 – Second dose adjustment visit and beginning of stable dose phase (D 21)

The posology is maintained for the next following 4 weeks.

If the tolerance does not allow it:

- Patients taking 40 mg/d pitolisant (high dose) or placebo could reduce to 20 mg/d pitolisant (medium dose) or placebo for the following 4 weeks.
- Patients taking 20 mg/d pitolisant (medium dose) or placebo could reduce to 10 mg/d

pitolisant (low dose) or placebo or placebo for the following 4 weeks.

Any dose increase will not be allowed at this visit.

After V4 until the end of Double Blind period any dosage change of pitolisant or placebo will not be allowed.

V5 – Control visit: continuation of stable dose phase (D 49)

Any dosage change of pitolisant or placebo will not be allowed at this visit. Hence, pitolisant or placebo treatment at the same stable dose will be continued for another 5 weeks.

V6 – Evaluation visit at the end of the Double Blind period and start of single-blind wash-out period (D 84)

Patients are submitted to the tests and examinations required for the analysis of the double blind study results. Patients will start one-week single blind placebo wash-out period.

Ph2 – Phone contact (V6 + 3D)

During this phone contact, the investigator should:

- Get patient's answers to amphetamine-like withdrawal symptoms questionnaire (DSM IV) and find out patient's global opinion on the effect of investigational drugs
- Check concomitant treatments
- Check occurrence of AEs

V7 – End of study visit for patients who are not entering Open Label Extension period or Beginning of the Open Label Extension period for patients who are entering Open Label Extension period: start of new escalating dose phase (D 91)

If the patient holds the same position towards nCPAP therapy as before, he/she will be proposed to enter the Open Label Extension period of the study after another escalating dose phase.

Patients not willing to continue the pitolisant treatment administration will end their participation in the study at this visit.

Patients willing to continue the pitolisant treatment administration will be given another information leaflet together with an informed consent form to be signed. After having signed the informed consent form to confirm that they agree to participate in the Open Label Extension study period, patients will be administered 10 mg pitolisant OD during one week. Then, on the following week, they will be administered 20 mg pitolisant OD (in the morning, during breakfast, with a glass of water).

V8-V9 – Dose adjustment visits (D 105 and D 112)

The posology is increased to 40 mg/d pitolisant (high dose), every morning, during breakfast. If the tolerance does not allow it (i.e. troublesome insomnia), the patient will get the treatment at dose of 20 mg/d (medium dose) or 10 mg/d (low dose).

At each of the visits V8-V11 the investigator will propose nCPAP to the patients refusing to be treated by nCPAP therapy before giving them the study treatment. If the patient accepts, he/she will be withdrawn from the study.

V10-V11 – Confirmed dose visits (D 196 and D 280)

The posology should be 40 mg/d (high dose) if the tolerance is acceptable. If the study drug is not well tolerated, the investigator may decide to decrease the dose to 20 mg/d (medium dose) or 10 mg/d (low dose) during the 12-week period following the visit.

Blood samples used for pharmacokinetic purpose will be collected during Visit 10.

V12 – Evaluation visit at the end of the Open Label Extension period and start of wash-out period (D 364)

Patients are submitted to the examinations and tests required for the evaluation of long-term tolerance and maintenance of efficacy of pitolisant. Patients will be submitted to a final visit after a one-week wash-out period. No study treatment will be taken during this period.

Ph3 – Phone contact (V12 + 3D)

During this phone contact, the investigator should:

- Get patient's answers to amphetamine-like withdrawal symptoms questionnaire (DSM IV) and find out patient's global opinion on the effect of investigational drugs
- Check concomitant treatments
- Check occurrence of AEs

V13 – End of study visit for patients who entered into Open Label Extension period (D 371)

The patients are summoned by the investigator for a final visit. Then, the investigator will decide about the new treatment to prescribe to the patients and the patients will be discharged from the study.

8. STUDY SITES

This trial will be conducted under the responsibility of [redacted]: UMHAT "Alexandrovska" – 1, Sv. Georgi Sofiyski Str., Sofia, Bulgaria – [redacted] This multicentre study will be conducted in several investigational centres in Europe; they will be opened, in hospitals and sleep disorder centres which will all have the ability to evaluate the efficacy and tolerance of the drug for the treatment of EDS in OSA patients.

9. INVESTIGATIONAL PRODUCTS

9.1. Chemical structure of compounds

Pitolisant (BF2.649) is 1-{3-[3-(4-chloro-phenyl)-propoxy]-propyl}-piperidinium, hydrochloride.

STRUCTURAL FORMULA

Pitolisant

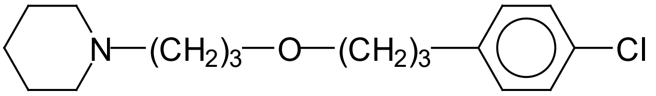

, HCl

MOLECULAR FORMULA

$C_{17}H_{26}ClNO$, HCl %C: 61.44 - %H: 8.19 - %N: 4.21 - %Cl: 21.34 - %O: 4.81

RELATIVE MOLECULAR MASS

Free base	295.85 g
Hydrochloride salt	332.31 g
Base/salt ratio	0.8903

9.2. Composition of investigational products

9.2.1. Composition of 5 mg and 20 mg pitolisant tablet

Composition of the active tablets	Pitolisant 5 mg strength	Pitolisant 20 mg strength
Active substance:<br>Pitolisant hydrochloride	 5,0 mg	 20,0 mg
Excipients:<br>Microcrystalline cellulose<br>Crospovidone<br>Talc<br>Magnesium stearate<br>Silica, colloidal anhydrous	 20,25 mg<br>2,50 mg<br>2,50 mg<br>0,75 mg<br>0,25 mg	 81,0 mg<br>10,0 mg<br>10,0 mg<br>3,0 mg<br>1,0 mg
**Core tablet mass**	**31,25 mg**	**125,0 mg**
Coating:<br>OPADRYII HP85F18422 white	 2,50 mg	 10,0 mg
**Mass of film coated tablet**	**33,75 mg**	**135,0 mg**

9.2.2. Composition of 5 mg and 20 mg placebo tablets

Composition of the placebo tablets	Placebo matching pitolisant 5 mg strength	Placebo matching pitolisant 20 mg strength
Microcrystalline cellulose<br>Crospovidone<br>Talc<br>Magnesium stearate<br>Silica, colloidal anhydrous	23,23 mg<br>2,30 mg<br>2,30 mg<br>0,69 mg<br>0,23 mg	94,536 mg<br>9,360 mg<br>9,360 mg<br>2,808 mg<br>0,936 mg
**Core tablet mass**	28,75 mg	117,0 mg
Coating:<br>OPADRYII HP85F18422 white	 2,30 mg	 10,0 mg
**Mass of film coated tablet**	31,05 mg	127,0 mg

9.2.3. Administration of study treatment

Both the patient and the investigator will be blind during the first Double Blind period. To make sure that the prescribed dose is appropriately administered to the patient in order to comply with the study conditions, all tablets of pitolisant 5 mg and matching placebo, same as all tablets of pitolisant 20 mg and matching placebo, will have the same mass, colour, shape and size.

During the wash-out period, patients will receive one-week single blind placebo (V6 to V7).

During the Open Label Extension period, the study treatment will be film-coated tablets

containing 5 mg or 20 mg of pitolisant.

The patient will be instructed to comply with the posology according to the investigator's judgment during each period.

For the Double Blind period, two 5 mg tablets, one or two 20 mg tablets of pitolisant, or placebo will be administered OD by the oral route according to the following administration scheme:

- In the morning, during breakfast, with a glass of water:

Patients under pitolisant

During breakfast	Low dose<br>10 mg<br>(two 5 mg tablets)	Medium dose<br>20 mg<br>(one 20 mg tablet)	High dose<br>40 mg<br>(two 20 mg tablets)

Patients under placebo will receive tablet(s) of matching placebo.

The therapeutic units will be prescribed to patients according to an individual treatment program and the posology will be determined during the titration phase.

For the Open Label Extension period, patients will be asked to take 2 pitolisant tablets of 5 mg (low dose 10 mg) or 1 pitolisant tablet of 20 mg (medium dose 20 mg) or 2 pitolisant tablets of 20 mg (high dose 40 mg) every morning, during breakfast, with a glass of water according to the schedule of escalating dose and further dose adjustment.

The two first posology (2 tablets of 5 mg and 1 tablet of 20 mg during one week for each) correspond to up-titration and the third one (2 tablets of 20 mg) is the selected dose for the duration of study, except if the tolerance is not acceptable.

9.2.4. Treatment Compliance

At each visit, the compliance to the treatment will be investigated. Patients will be asked whether the investigational treatment was taken as prescribed.

If not, any change in the treatment and the number of forgotten tablets will be recorded in the CRF. In addition, the number of remaining tablets of pitolisant will be counted and compared to the theoretical number which should be left. Any discrepancy will be investigated with the patients.

9.2.5. Packaging and labelling of the treatment boxes

All the study treatments will be supplied, packaged and labelled in compliance with the Good

Manufacturing Practices (GMP) and ICH E6 [guidelines for drugs used in clinical trial].

Double Blind period

To provide patients with the required treatment in accordance with the treatment arm and the dosage regimen 3 types of polyethylene high density (PEHD) bottles with active product or placebo will be prepared:

Bottle A (pitolisant 10 mg), containing 20 tablets of pitolisant 5 mg or matching placebo (low dose)

Bottle B (pitolisant 20 mg), containing 10 tablets of pitolisant 20 mg or matching placebo (medium dose)

Bottle C (pitolisant 40 mg), containing 20 tablets of pitolisant 20 mg or matching placebo (high dose)

For single-blind wash-out period bottles containing 20 tablets placebo matching to pitolisant 5 mg, 10 tablets placebo matching to pitolisant 20 mg or 20 tablets placebo matching to pitolisant 20 mg will be used.

The bottles will be packed into boxes. Each patient treatment box will include 35 bottles allowing for all types of study regimen packed into several boxes. The boxes will be packaged into carton-boxes.

The outer packaging of study treatment contains a label with a tear-off part. This tear-off part of the label will be removed and applied on study specific documents. Specific label information will be registered on the corresponding page of the CRF.

Open Label Extension period

For the escalating dose period (at V7) and dose adjustment (at V8), the following PEHD bottles will be prepared:

- Pitolisant 10 mg, containing 20 tablets of pitolisant 5 mg
- Pitolisant 20 mg, containing 10 tablets of pitolisant 20 mg
- Pitolisant 40 mg, containing 20 tablets of pitolisant 20 mg

Each patient treatment box (for the escalating dose period) will include 5 bottles allowing for all types of study regimen packed into 2 boxes. The boxes will be packaged into carton-boxes. For visits from V9 to V11, PEHD bottles will contain 30 tablets of pitolisant 5 mg or 20 mg.

9.2.6. Treatment quantity

Double Blind period

At each visit patients will receive per week 1 bottle containing 20 tablets of 5 mg pitolisant / matching placebo, or 10 tablets of 20 mg pitolisant / matching placebo, or 20 tablets of 20 mg pitolisant / matching placebo; at V6 for the single blind wash out period – containing 20 tablets placebo matching to pitolisant 5 mg, 10 tablets placebo matching to pitolisant 20 mg or 20 tablets placebo matching to pitolisant 20 mg.

Open Label Extension period

For the escalating dose period (at V7) and dose adjustment (at V8) patients will receive per week 1 bottle containing 20 tablets of pitolisant 5 mg, or 10 tablets of pitolisant 20 mg, or 20 tablets of pitolisant 20 mg.

From V9 to V11, at each visit patient will be given in the quantity needed for prescribed treatment bottles of 30 tablets dosed at 5 mg or 20 mg.

9.2.7. Management and storage of the therapeutic units

The necessary therapeutic units will be provided to the hospital pharmacist or the investigator of sleep disorders centres in the minimum period of time before the beginning of the study, and all necessary documents on pitolisant information, according to the Good Clinic Practice (GCP-ICH topic E6 and local regulations) and the local laws.

The hospital Pharmacist or Investigator in sleep disorders centre will be responsible for the management, and the accountability of the therapeutic units (reception, storage, dispensation log, update of the delivery list, return of empty packaging and unused units, and return of therapeutic units). As soon as the treatment units are received, the pharmacist will return the enclosed acknowledgement of receipt sheet distribution, filled and signed.

Treatments under the Pharmacist's or investigator's responsibility must be stored in a locked room and disclosed only to authorized persons having access to this storage room.

In accordance with GCP regulations (ICH topic E6 and local regulations) and the local laws, all study materials (unused treatment units, packaging as well as individual code envelopes) have to be retrieved by the pharmaceutical company responsible of supplying, in accordance

with their internal procedure, at the end of the study. The return certificate will indicate for each patient:

- Number of therapeutic units administered,
- Number of therapeutic units unused,
- Number of therapeutic units finally returned to supplier and the dispatch date

In case of a therapeutic unit, and/or individual code envelops loss, the investigator, or the hospital's Pharmacist in charge of the treatment management will have to justify this loss in a written statement, signed and dated, enclosed with the return certificate.

The unused therapeutic units will be retrieved by the pharmaceutical company responsible of supplying at the end of study (i.e. after final study report signature) after reconciliation of the dispensation log of the therapeutic units. Exceptionally, the unused therapeutic units could be destroyed by the investigator after sponsor being given its agreement

9.2.8. Dispensation Modalities

The study drug will be dispensed only under the restricted conditions defined in the present protocol. Drugs will be administered only by the investigator, or under his direct supervision. The tear-off label of each study drug package will be stuck in the CRF.

9.2.9. Pharmacological Forbidden Concomitant Treatments and surgical interventions

The following pharmacological treatments are forbidden during the study:

- All drugs indicated for somnolence
- All drugs containing sodium oxybate
- All hypnotics drugs defined by ATC class
- Tricyclic antidepressants such as clomipramine, imipramine, desmethylimipramine and protriptyline displaying histamine H_1 receptor antagonist activity that may affect the activity of pitolisant by abrogating the effect of its endogenous histamine release
- H_1 receptor antagonists (in particular those having an effect on the central nervous system)
- Central antihypertensive drugs (Clonidine)
- Any formulation containing codeine

- Psychostimulants (Amphetamine and amphetamine-like CNS stimulants, methylphenidate, modafinil or others)
- Drugs containing dextropropoxyphene (Di-Antalvic)

Surgical interventions including Mandibular Advancement orthosis, uvulopalatopharyngoplasty (UPPP) are also considered as prohibited treatments.

All pharmacological forbidden treatment(s) will be stopped at the selection visit.

A wash out period will be ensured before the inclusion visit.

Exceptionally, or a non-tricyclic antidepressant could be authorized under the same conditions as the chronic treatments prescribed at a stable dose for at least one month before V1, without any modification throughout the study period and without interfering with daytime sleepiness.

The wash out period will have a minimum of 14 days before inclusion, if the patient is under prohibited treatment.

As far as possible, the concomitant intake of any other treatment will be avoided during the whole study course. All chronic treatments deemed necessary for the patient's condition will remain unchanged the last month prior to enrolment, and during the study period. However the medical state of the patient may require the modification of some treatments for example changing the posology, initiating a new treatment, or stopping a current treatment. All of these treatments have to be reported in the CRF, specifying the posology (name of the drug, type of formulation, unit strength, time and duration of treatment), the prescription date and the reason for prescription.

9.2.10. Randomization of treatments

9.2.10.1. Patient Study number

The study medication will be administered only to patients eligible for the study following the procedures set out in the clinical study protocol.

A patient number (two digits start i.e. 01, 02…) will be assigned at each site sequentially following the number of the study site. The study site number is composed by a sequential number of two digits (01, 02…).

The treatment number is composed by a sequential number of 3 digits for the Double Blind period (DB-XXX) and will remain the same during the dose escalation phase of the Open Label Extension period (OL-XXX). For the stable dose phase, starting at V9, all treatments codes will

include a 4 digits number and will be either OL5-YYYY or OL20-YYYY.

A treatment number will be allocated for each patient by ARONE France (4, rue Leroux 94100 Saint-Maur des Fossés – France.

A tracking list of the number allocated with the identification of patients will be filled by the investigator, and kept in his study records.

9.2.10.2. *Investigational treatment allocation*

<u>Double Blind period</u>

A randomization list will be generated by an entitled company according to Good Clinical Practices. The therapeutic units will be distributed in function of the list managed by ARONE – 4, rue Leroux 94100 Saint-Maur des Fossés – France. Eligible patients will receive a random number corresponding to the treatment assigned in accordance with the randomization schedule. Each treatment unit will have a number printed on the medication label.
The investigator will document the treatment number (or randomization number) on the CRF. Patients withdrawn from the study will retain their random number. If the decision to replace withdrawn patients is taken by the investigator with the sponsor representative or, if necessary, by the scientific committee, the new patient should be allocated the available treatment.

The necessary quantity of study treatment to be administered during the Double Blind period will be sent to the pharmacist at selection. The study treatment will be delivered to the patient according to their treatment number by the investigator, at inclusion. Then, at each visit, the necessary quantity of study treatment will be given to the patient until the next visit.

The investigator will document the blister number delivered to the patients in the CRF.

9.2.10.3. *Unblinding envelopes*

The double blind design of the study will result in the ignorance of the assigned treatment to the patients, so during the Double Blind period of the study the assigned treatment will be blinded.

. Two sets of sealed envelopes (one for the pharmacist and another one for the investigator), identified by the study number and treatment number, and containing the unblinded information regarding the type of treatment assigned to the corresponding patient will be sent to the investigators. Another set will be available at the sponsor's level.

In case of emergency the individual code may be broken (see 11.2).

All envelopes will be recovered by the sponsor at the completion of the study.

10. EVALUATION CRITERIA

10.1. Efficacy criteria

10.1.1. Primary endpoint: Epworth sleepiness scale (ESS)

The primary endpoint efficacy criterion is based on the score of Epworth Sleepiness Scale (ESS) by comparing the score differences between baseline (mean between V1 and V2) and at the end of the Double Blind period (mean between V5 and V6) in pitolisant and placebo groups and the sustained improvement of ESS from baseline at week 52 in the Open Label Extension period.

This rating scale is a subjective, validated, sensitive and specific assessment of sleepiness in OSA patients in eight situations described hereafter:

1. Sitting and reading
2. Watching the television
3. Sitting inactive in a public place (cinema, theatre, meetings)
4. Passenger in a car or in the public transportation for at least one hour without stop
5. Lying down to rest in the afternoon in conditions allowing to have rest
6. Sitting and talking to someone
7. Sitting quietly after a lunch
8. In a car when stopped few minutes in a traffic jam

The patient has to rate his or her likelihood of dozing or falling asleep. Each of the eight questions is rated from 0 to 3

0 = never, or no likelihood of falling asleep

1 = rarely falling asleep, or slight likelihood of falling asleep

2 = Often falling asleep, or moderate likelihood of falling asleep

3 = high probability of falling asleep

The responses are summed to yield a score between 0 and 24 with higher scores representing greater sleepiness. The score greater or equal to 11 is considered as abnormal sleepiness.

ESS will be evaluated at each study visit, and reviewed by the investigator.

The questionnaire will be given to the patients at approximately the same time of the visit day

and with the same delay after the investigational treatment intake, in order to standardize the possible impact of treatment on the evaluation.

The patient should be supported in the comprehension of questionnaire until he / she fully understands the eight questions of the score. The patient will be reminded that the ESS measures the subjective sleepiness with regard to the immediate past week.

The value and the sum of each rated question will be uploaded by the investigator in the CRF during the visits.

ESS will be performed at each study visit.

An example of the ESS questionnaire is provided in Appendix 3.

10.1.2. Secondary endpoints

10.1.2.1. Percentage of ESS responders

A responder corresponds to a patient in whom the absolute value of the ESS is < 11, or the difference between the baseline and the end of the Double Blind period scores ≥ 3.

10.1.2.2. Reduction of sleepiness and sleep episodes on the sleep diary

The patient diary is a booklet developed and used to capture, by each patient, his/her subjective evaluation of EDS as a secondary outcome measure. A sleep diary will be reviewed for each patient at each visit except V1 (V2, V3, V4, V5, V6, V7, V8, V9, V10, V11, V12 and V13).

Each patient will be instructed to answer to the sleep diary questions on 3 sequential days of the week preceding the next visit, and to bring back the completed diary.

The patients will answer the following questions:

1. What time did you go to sleep last night?
2. What is the estimated time of your falling asleep last night?
3. Number of awakening episodes during last night.
4. Total duration of these awakening episodes during last night.
5. What time did you wake up this morning?
6. What time did you get up this morning?
7. Number of episodes of sleep and sleepiness during this day.
8. Total duration of sleep and sleepiness during this day.

The analysis of the sleep diary will be based on the responses of the patients.

An example of the sleep diary questionnaire is provided in Appendix 4.

10.1.2.3. *Improvement in vigilance according to Oxford Sleep Resistance (OSleR) test*

The OSleR test consists in 40 minutes sleep-resistance challenges conducted in a dark room isolated from external noise. The subject, dressed and lying in semi recumbent position, is asked to stay awake without using specific strategies. By hitting a button placed on a box directly connected to a personal computer, the subject is instructed to respond to a visual stimulus (light-emitting-diode flash) which appears for 1 second every 3 seconds.

Any time the red light flashes, the patient is asked to place his/her finger on the yellow button for about 1 second, then to remove his/her finger. He/she is instructed to repeat the cycle every time he/she sees the red light flashing. When the light stops blinking, the test is ended.

All tests are video recorded to check that the subjects are following the instructions. Each patient at V2, V6, V7 and V12 (optionally) undergoes the OSleR in 3 sessions with 2 hours interval between them (at 9:00, 11:00 and 13:00), allowing the assessment of vigilance at different times of the day. The OSleR test algorithm defines the sleep onset when there is no response to 7 consecutive flashes (≥ 21 s)[15].

The standard OSleR test used in the study will yield results of mean sleep latency (MSL) and errors (non-responses to stimulations). For each OSleR test, the consecutive errors are allocated to an error profile (EP) type (i.e. two consecutive errors = EP2, three consecutive errors = EP3).

The EPs are clustered into type:

- EP 1-2 for one and two consecutive errors, indicating lack of attention
- EP 3-6 for three to six consecutive errors, indicating micro-sleep
- EP ≥ 7 for 7 or more consecutive errors, indicating sleep onset

Improvement of vigilance will be evaluated according to increase in sleep latency and reduction in the number of errors.

10.1.2.4. *Increase in quality of life*

Increase in quality of life will be assessed using European Quality of Life Questionnaire, Leeds Sleep Evaluation Questionnaire and The Pichot Fatigue Scale.

European Quality of Life Questionnaire (EQ-5D)

EQ-5D is a standardized instrument used as measure of health outcome. Applicable to a wide range of health conditions and treatments, it provides a simple descriptive and a single index value for health status.

EQ-5D essentially consists of 2 pages: the EQ-5D descriptive system and the visual analogue scale (VAS).

The EQ-5D descriptive system comprises the following 5 dimensions: mobility, self-care, usual activities, pain/discomfort, and anxiety/depression. Each of the 5 dimensions comprising the EQ-5D descriptive system is divided into 3 levels of perceived problems: Level 1 (no problem); Level 2 (some problems); Level 3 (extreme problems). The patient is asked to indicate his/her health state by ticking (or placing a cross) in the box against the most appropriate statement in each of the 5 dimensions.

A unique health state is defined by combining 1 level from each of the 5 dimensions. Each state is referred to in terms of 5 digit code. For example, state 11111 indicates no problems on any of the 5 dimensions, while state 11223 indicates no problems with mobility and self care, some problems with performing usual activities, moderate pain or discomfort and extreme anxiety or depression.

The EQ-5D VAS records the patient's self-rated health on a vertical, visual analogue 100-point scale where the endpoints are labelled "Best imaginable health state" and "Worst imaginable health state".

EQ-5D will be performed at V2, V6, V7 and V12:

An example of the EQ-5D questionnaire is provided in Appendix 5.

Leeds Sleep Evaluation Questionnaire (LSEQ)

The LSEQ[16] will be used to record key elements of aspects of sleep and early morning behaviour.

The LSEQ contains ten questions pertaining to four consecutive aspects of sleep: getting to sleep (GTS), quality of sleep (QOS), awakening from sleep (AFS), and behaviour following wakefulness (BFW).

Each patient will be asked 10 questions. A 10-cm line separates the two halves of each question. Each question is answered by placing a vertical mark on the answer line. If no change was experienced, the mark is placed in the middle of the line. If a change was experienced then the

position of the mark will indicate the nature and the extent of the change.

The LSEQ will be performed at V2, V6, V7 and V12.

An example of the LSEQ is provided in Appendix 6.

The Pichot Fatigue Scale

The Pichot questionnaire is a practical 24-item self-rating account with three homogeneous sub-scales of 8 items each which measure depressive mood, asthenia-fatigue and anxiety parameters, respectively.

The asthenia-fatigue scale used in this study, consists of 8 questions scored progressively from "0" (not at all) to "4" (extremely) in the following situations:

1) I feel short of stamina
2) Everything I do requires a huge effort
3) I have a feeling of weakness in certain parts of my body
4) My arms or legs are heavy
5) I feel tired for no reason
6) I feel like lying down or resting
7) I have difficulty to concentrate
8) I feel discouraged, my arms and legs are sore and heavy

A score > 22 is in favour of excessive fatigue.

The Pichot Fatigue Scale will be performed at V2, V6, V7, V9, V10, V11, V12 and V13.

An example of the Pichot Fatigue Scale is provided in Appendix 7.

10.1.2.5. Improvement in cognitive function

Improvement in cognitive function will be assessed using Trail Making Test.

Both parts (A and B) of the TMT consist of 25 circles distributed over a sheet of paper.

In Part A, the circles are numbered 1 to 25, and the patient should draw lines to connect the numbers in ascending order.

In Part B, the circles include both: numbers (1 to 13) and letters (A to L). As in Part A, the patient draws lines to connect the circles in an ascending order, but with the added task of alternating numbers and letters (i.e., 1-A-2-B-3-C, etc.).

The patient should be instructed to connect the circles as quickly as possible, without lifting the pen or pencil from the paper. Time used by the patient to connect the "trail" is registered. If the patient makes an error, it is pointed out immediately and the patient is allowed to correct it. Errors affect the patient's score only in that the correction of errors is included in the completion time for the task. It is unnecessary to continue the test if the patient has not completed both parts after five minutes have elapsed.

Step 1: Give the patient a copy of the Trail Making Test Part A worksheet, and a pen or pencil.

Step 2: Explain the test to the patient using the sample sheet (Trail Making Part A – *SAMPLE*).

Step 3: Time the patient as he or she follows the "trail" made by the numbers on the test.

Step 4: Record the time.

Step 5: Repeat the procedure for Trail Making Test Part B.

Scoring:

Results for both TMT A and B are reported as the number of seconds required to complete the task; therefore, higher scores reveal greater impairment.

The average time to complete the test is for Part A: 29 seconds and for Part B: 75 seconds. Scores superior to 78 seconds for Part A and 273 seconds for Part B reveal deficiency.

The TMT parts A and B will be performed at V2, V6, V7 and V12.

An example of the Trail Making Test (TMT) parts A and B is provided in Appendix 8.

10.1.2.6. Improvement in Clinical Global Impression (CGI)

The CGI is a 3-item observer-rated scale which measures illness severity (CGI-S), global improvement or change (CGI-C), and therapeutic response. The CGI is rated on a 7 point scale, with the CGI-S scores ranging from 1 (normal) to 7 (the greatest severity) and CGI-C scores ranging from 1 (very much improved) to 7 (very much worse). Treatment response ratings should take into account both therapeutic efficacy and treatment-related adverse events, and range from 0 (marked improvement and no side effects) to 4 (unchanged or worse, and side-effects outweigh the therapeutic effects).

The CGI-S test measure will be performed at V1 and V2, CGI-C test measure – at V6, V7, V10, V11, V12 and V13.

An example of the CGI scales is provided in Appendix 9.

10.1.2.7. Patient's global opinion on the effect of investigational drugs

The patient should evaluate the global effect of the treatment, by comparing the period prior to the visit with the patient's pre-study condition. The following six-level scale will be used:

- Marked effect (complete or nearly complete remission of EDS)
- Moderate effect (partial remission of EDS)
- Minimal effect (slight decrease in EDS that does not substantially change the status of the patient)
- No change
- Minimally worse (slight increase in EDS)
- Much worse (substantial increase in EDS)

The Patient's global opinion on the effect of investigational drugs measure will be performed at V6, Phone contact 2, V7, V10, V11, V12, Phone contact 3 and V13,

An example of the Patient's global opinion on the effect of investigational drugs is provided in Appendix 10.

10.1.2.8. Aggregate Z-score of secondary endpoints

ESS and OSLER constitute two essential symptoms of EDS, whereby it is possible to assess the overall efficacy of the tested drug, through a multivariate approach, by using a unique test on a composite Z-score.
This Z-score will be calculated for ESS (ESSBL and ESSF) and (OSLERBL and OSLERF), for all these variables both at baseline and final visit.

10.2. Safety criteria

A Data Safety Monitoring Board (DSMB) will be created. It will be made up of a group of independent expert's external to the study assessing the progress, safety data and, if needed critical efficacy endpoints of the clinical study.
In order to do so, a DSMB may review unblinded study information (on a patient level or treatment group level) during the conduct of the study. Based on its review, the DSMB provides the sponsor with recommendations regarding study modification, continuation or termination.
DSMB Charter is provided in Appendix 11.

10.2.1. Period of observation

For the purpose of this study, the period of observation extends from the time the patient gives informed consent (Visit 1) until one month after the last visit (Visit 7 for patient not entering in the Open Label Extension period, and V13 for patient participating in the Open Label Extension period,). Any adverse events observed by the investigator or reported by the patient during the period of observation must be documented in the CRF.

If the investigator detects a serious adverse event in a patient after the end of the observation period, and considers the event possibly related to the study treatment, he or she should contact the sponsor to determine how the adverse event should be documented and reported.

10.2.2. Adverse Event (AE)

The term Adverse Event: Any untoward medical occurrence in a patient or clinical investigation subject administered a pharmaceutical product and which does not necessarily have a causal relationship with this treatment. An AE can therefore be any unfavourable and unintended sign (including an abnormal laboratory finding), symptom, or disease temporally associated with the use of a medicinal (investigational) product, whether or not related to the medicinal (investigational) product.

No causal relationship with the study treatment or with the clinical study itself is implied by the use of the term "Adverse Event".

Adverse Events fall into the categories "non-serious" and "serious" (see Section "Serious Adverse Event").

AEs include also:

- Clinically abnormal laboratory results
- Overdosage
- Abnormal ECG results

For any reports in the above categories an AE form has to be filled in addition to the exam results.

10.2.3. Treatment-Emergent and Baseline-Emergent Adverse Events

A Treatment-Emergent Adverse Event is defined as any event which is reported as occurring after the first study drug intake or any event already present before first study drug intake and

which worsens either in intensity, or frequency following the exposure to the study treatments. A Baseline-Emergent Adverse Event is defined as any event which occurs or worsens during the staged screening process (after informed consent is given) including the randomization visit.

10.2.4. Adverse Drug Reaction (ADR)

All noxious and unintended responses to a medicinal product related to any dose should be considered as an Adverse Drug Reaction. The responses to a medicinal product means that a causal relationship between a medicinal product and an adverse event is at least a reasonable possibility, i.e. the relationship cannot be ruled out.

10.2.5. Unexpected Adverse Drug Reaction

An adverse reaction, in nature or severity which is not consistent with the applicable product information (e.g., Investigator's Brochure for an unapproved investigational product or package insert/summary of product characteristics for an approved product).

10.2.6. Serious Adverse Event (SAE) or Serious Adverse Drug Reaction (Serious ADR)

Any untoward medical occurrence that at any dose:

- Results in death
- Is life-threatening[1]
- Requires inpatient hospitalization or prolongation of existing hospitalization
- Results in persistent or significant disability/incapacity[2]
- Is a congenital anomaly/birth defect, or
- Other: important medical event according to the investigator[3]

1 "Life-threatening" means that the patient was at immediate risk of death at the time of the serious adverse event; it does not refer to a serious adverse event that hypothetically might have caused death if it were more severe.

2 "Persistent or significant disability or incapacity" means that there is a substantial disruption of a person's ability to carry out normal life functions.

3 Medical and scientific judgment should be exercised in deciding whether other adverse events may be considered serious because they jeopardize the patient, or may require intervention to

prevent one of the other outcomes listed in the definition above.

The List of Critical Terms (1998 adaptation of WHO Adverse Reaction Terminology Critical Terms List) should be used as guidance for adverse events that may be considered serious because they are medically important.

Cases involving cancer as an Adverse Event should be reported as "serious" using the criterion "medically important" if no other serious criterion is met.

Cases of overdose with an adverse event that meets one of the criteria given above should of course be reported as "serious".

10.2.7. Suspected Unexpected Serious Adverse Reaction (SUSAR)

Suspected Unexpected Serious Adverse Reaction: any Adverse Reaction that is classed as serious and is suspected to be caused by the IMP and is not consistent with the information about the IMP in either the Investigator's Brochure or SmPC.

10.2.8. Clarification of the difference in meaning between "severe" and "serious"

The term "severe" is often used to describe the intensity (severity) of a specific event (such as in mild, moderate, or severe myocardial infarction); the event itself, however, may be of relatively minor medical significance (such as severe headache). This is not the same as "serious," which is based on the outcome or action criteria usually associated with events that induce a threat to life or functioning.

Seriousness (not severity) serves as a guide for defining regulatory reporting obligations.

10.2.9. Analysis of adverse events

10.2.9.1. Assessment of intensity

The assessment of intensity (severity) is independent on the assessment of the seriousness of the AE and is based on the investigator's clinical judgment.

Mild: Awareness of signs and symptoms but no disruption of usual activity. Symptoms do not require therapy or a medical evaluation; signs and symptoms are transient.

Moderate: Event sufficient to affect usual activity (disturbing). Are usually improved by simple therapeutic measures.

Severe: Inability to work or perform usual activities (unacceptable). Generally require systemic drug therapy or other treatment.

10.2.9.2. Assessment of Causality

The causal relationship of the adverse event to the study treatment will be assessed and notified according to the following definitions:

Related / likely: Clearly related to the investigational agent / procedure, i.e. an event that follows a reasonable temporal sequence from administration of the study intervention, follows a known or expected response pattern to the suspected intervention, that can be confirmed by improvement on stopping and reappearance of the event after rechallenge and that could not be reasonably explained by the known characteristics of the subject's clinical state.

Possibly related / Possible: Follows a reasonable temporal sequence from administration of the study intervention, follows a known or expected response pattern to the suspected intervention, but that could readily have been produced by a number of other factors.

Not related / Unlikely: Clearly and incontrovertibly due only to extraneous causes, and does not meet criteria listed under possible (possibly related) or likely (related).

10.2.10. Reporting Adverse Event to the Sponsor (Appendix 12)

All Adverse Events that occur after the patient has signed the informed consent must be documented on the pages provided in the CRF in accordance with the "Instructions for the completion of Adverse Events". These instructions are provided in the investigator study file, and in the CRF itself.

The following approach will be taken for documentation:

All adverse events (whether serious or non-serious) must be documented on the "Adverse Event" page of the CRF including clinically significant lab or examination results.

If the adverse event is serious, the investigator must complete a "SAE report form" at the time the Adverse Event is considered as Serious.

In the situation when a "significant overdose" occurs, the investigator should complete the "Adverse Event" page of the CRF. If this overdose presents one of the criteria outlined in section 10.2.6 (definition of SAE), the investigator should complete the "SAE report form".

Every attempt should be made to describe the Adverse Event in terms of a diagnosis. If appropriate, component symptoms should also be listed below the diagnosis.

If only non-specific signs or symptoms are present, then these should be recorded as a diagnosis.

All patients who experience an Adverse Event, whether considered associated with the use of the study medication or not, must be monitored to determine the outcome. The clinical course of the Adverse Event will be followed up according to accepted standards of medical practice, even after the end of the observation period, until a satisfactory explanation is found, or the investigator considers it medically justifiable to terminate follow-up.

Should the adverse event result in death, a full pathologist's report should be supplied, if possible.

All questions on the completion and supply of Adverse Event report forms, and any further forms issued to the investigator at a later date to clarify unresolved issues should be addressed to the sponsor.

10.2.11. Reporting Serious Adverse Event to the sponsor

Serious Adverse Events and Adverse Events that fulfil a reason for expedited reporting to Pharmacovigilance (significant overdose, pregnancy) must be documented on a SAE report form in accordance with the "Instructions for Completing the SAE report form" (see Appendix 12).

The investigator must inform the site monitor and sponsor in all such cases within 24 hours after the investigator becomes aware of the SAE (or at the latest on the following working day). The SAE form must be completed and faxed [[redacted]] or emailed [redacted]] to Bioprojet Pharmacovigilance department at latest within 3 calendar days after the investigator becomes aware of the SAE. The initial report must be as complete as possible, including details of the current illness and the Serious Adverse Event, and most importantly an assessment of the causal relationship between the SAE and the study medication. Information not available at the time of the initial report (e.g., end date of the Serious Adverse Event or laboratory values received after the report) must be documented on a follow-up "SAE report form".

In addition, the following CRF data must be provided as soon as possible to Bioprojet Pharmacovigilance Department: "Demography", "Relevant diseases/illnesses in the patient's medical history", "Previous and concomitant medication".

The site monitor is responsible to ensure that the entire information is forwarded to Bioprojet Pharmacovigilance department. Bioprojet will ensure that all legal reporting requirements are met.

The SAE report form and the instructions on completion are provided in the investigator's study file. The "Instructions for Completing the SAE report form" give more detailed guidance on the reporting of serious adverse events, significant overdose cases, and Adverse Events initially reported as non serious that become serious. In the latter situation, where a non-serious event becomes serious, details must be forwarded immediately to the sponsor on a SAE report form".

10.2.12. Reasons for expedited reporting

No special Adverse Events are subject to reporting as alert terms in this study.

However, cases in which a "significant overdose" of the investigational product is to be reported to the sponsor on a SAE report form is to be dispatched to the sponsor in an expedited manner.

In addition, any pregnancy diagnosed in a subject during treatment with the investigational product must be reported to the sponsor immediately and must be followed up until child birth. Patient should immediately stop the study treatment.

During and after a patient's participation in the trial, the investigator should ensure that adequate medical care is provided to the patient for any adverse events, including clinically significant laboratory values, related to the trial. The investigator should inform the patient when medical care is needed for intercurrent illness(es) of which the investigator becomes aware.

10.2.13. Adverse Events Outcome

All Adverse Event outcomes should be documented at the last visit, the latest according to the following criteria:

- Recovered
- Recovered with sequelae
- Worsened
- Not yet recovered
- Death

10.2.14. Safety Endpoints

10.2.14.1. Adverse events

Adverse Events, emergent or not, reported during the study course (frequency, intensity, relationship to study drug, incidence and occurrence) will be analyzed as the main safety parameters.

The events will be categorized by organ class (current MedDRA) and type, seriousness, intensity and relationship to the treatment.

10.2.14.2. Physical examination

A full physical examination will be performed at each visit. Any significant abnormality and change from baseline will be recorded in the CRF and in the source document, and will be analyzed as safety parameters. Any untoward change will be reported in the AE pages of the CRF.

10.2.14.3. Vital signs

Vital signs including systolic and diastolic blood pressures and heart rate will be measured at each visit. Measurements will be recorded in the CRF and in the source document.

Any change in vital signs parameters (heart rate, blood pressure) from baseline will be analyzed as safety parameters. Particular attention will be focused on cardiovascular examination, essentially by verifying the arterial blood pressure at screening and at each study visit.

10.2.14.4. Electrocardiogram (ECG)

ECG will be performed at each study visit using the internationally recognised ECG recording. ECG parameters including heart rate and QTc (electrocardiogram Fridericia corrected QT interval ($QT/\sqrt[3]{[60/HR]}$) interval will be analyzed and reported in the CRF and in the source document. Patients presenting a QTc strictly higher than 450 ms will not be eligible in this study. QTc and other ECG parameters intra individual changes will be reported and analyzed in all treatment groups at V6.

10.2.14.5. Beck Depression Inventory (the reduced version with 13 items)

The Beck Depression Inventory created by Dr. Aaron T. Beck, is a multiple-choice self-report

questionnaire, one of the most widely used instruments for measuring the severity of depression. The current reduced version with 13 items is composed of items relating to symptoms of depression such as hopelessness and irritability, cognitions such as guilt or feelings of being punished, as well as physical symptoms such as fatigue, weight loss, and lack of interest in sex (Beck AT (2006) Depression: Causes and Treatment. Philadelphia: university of Pennsylvania Press).

The BDI-13 will be performed at visits V1, V2, V6, V7, V9, V10, V11, V12 and V13.

An example of BDI-13 is provided in Appendix 13.

10.2.14.6. Patient's overall evaluation of the tolerance

The patient should evaluate tolerance of the treatment. The following three-level scale will be used:

- Good
- Moderate
- Poor

Patient's overall evaluation of the tolerance will be performed at visits V3, V4, V5, V6, V7, V8, V9, V10, V11, V12 and V13.

An example of Patient's overall evaluation of the tolerance is provided in Appendix 14.

10.2.14.7. Amphetamine-like withdrawal symptoms questionnaire (DSM IV)

The Amphetamine-like withdrawal syndrome is defined as dysphoria and two or more of the following: fatigue, vivid and unpleasant dreams, insomnia or hypersomnia, increased appetite, and psychomotor retardation or agitation (DSM IV).

This questionnaire will be performed at phone contact 2, V7, phone contact 3 and V13.

An example of Amphetamine-like withdrawal symptoms questionnaire is provided in Appendix 15.

10.2.14.8. Laboratory tests

A full laboratory test should be performed at V1 (screening visit) and at V6 for each patient completing the Double Blind period. Patients entering into the Open Label Extension period will have additionally lab tests at V12. Biological work up will also be performed as soon as

possible for any subject who discontinues the study prior to the completion of the Double Blind period or the Open Label Extension period.

Blood samples will be collected and all laboratory tests will be performed according to the Good Laboratory Practices (OECD GLP Principles) by site-dependant Laboratory.

The results of the laboratory tests will be interpreted by the investigator. Laboratory abnormalities will be defined as laboratory test results that are outside the reference range as defined by the normal range from the testing laboratory.

Clinically significant abnormal value at V1 (results available at V2) will be determined by the investigator, and will lead to the exclusion of patients from study participation.

The results of all values of laboratory tests will be reported in the CRF. The original document containing all the tested values will be kept in the patient study file.

Blood laboratory test parameters will be reported in the CRF and intra individual changes in these parameters will be analyzed in both treatment groups at visit 6 for the Double Blind period, and at visit 12 for all patients entered into the Open Label Extension period. Any abnormalities considered clinically significant will be recorded in the adverse event page of the CRF.

The following blood parameters will be evaluated:

Hematology (hemoglobin, hematocrit, white blood cell [WBC] count with differential, red blood cell [RBC] count, platelet count, mean corpuscular volume, and coagulation time [INR]) will be performed at V1, V6, V12 and at early withdrawal visit;

Biochemistry (blood urea nitrogen [BUN], uric acid, creatinine, creatine kinase, SGPT [alanine aminotransferase (ALAT)], SGOT, [aspartate aminotransferase (AST)], γGT [Gamma-Glutamyltranspeptidase], alkaline phosphatase, total protein, total bilirubin, glucose, albumin, electrolytes [sodium, potassium, calcium, chloride, Bicarbonates/CO2], total cholesterol and triglycerides) will be performed at V1, V6, V12 and at early withdrawal visit.

β-HCG serum pregnancy test will be performed at V1, V6, and V12 and at early withdrawal visit in all patients of child-bearing potential. The absence of pregnancy should be confirmed before study drug delivery (at V2 for the Double Blind period and at V7 for the Open Label Extension period).

Serological test (HIV, HBsAg, HCV) will be performed only at the screening visit V1 (patients with positive to serology test will not be included to study).

Urinalysis will be performed using stick test at V1. The results will be recorded in the CRF. Should the stick test be positive, a mid stream sample of urine will be sent for microscopy and bacteriological culture.

Pharmacokinetic sampling will be done at V10 for a number of patients (no samples to be done during the study extension). Samples will be taken at pre-dose, 1,5 h, 3 h and 8 h post-dose (see §10.2.14.9 below).

Any laboratory test abnormalities considered clinically significant putting the patient at risk, as judged by the investigator, and if necessary by the scientific committee will lead to immediate discontinuation of the study drug. The Sponsor must be informed immediately.

In case the investigator considers abnormalities will not put the patient at risk, continuation of the drug will be allowed after discussion with the Sponsor and the scientific committee.

The patient will be followed up with appropriate medical care until he/she returns to normal or baseline values, or clinical diagnostics of undercurrent illness is confirmed.

The results of all known laboratory tests required by the protocol will be held and recorded in the patients CRFs. All clinically important abnormal laboratory tests occurring during the study will be repeated at intervals judged appropriate by the investigator until they return to baseline or to a level deemed acceptable by the investigator.

10.2.14.9. Pharmacokinetic assessments

Pharmacokinetic sampling will be done at V10 for a number of patients (no samples to be done during the study extension). Blood samples for PK analysis will be performed at pre-dose, 1.5 h, 3 h and 8 h post-dose.

For pitolisant and metabolites determinations, a 4.0 ml blood sample will be collected into one polyethylene terephthalate (PET) tube without any anticoagulant. After clotting (45 minutes at room temperature), serum will be separated in a refrigerated centrifuge (*ca.* +4°C) at *ca.* 1500 *g* for 10 min for and the complete volume of resulting serum (at least 1.8 ml from 4.0 ml blood sample) will be dispensed equally to 3 polypropylene cryotubes (no screwed-tap) with a minimum of 0.55 ml per tube and stored upright at –80 ± 10 °C. Upon collection, the samples will be processed as soon as possible. If not processed immediately, the samples should be kept on wet ice for a maximum time of 1 h.

Each tube will be precisely identified, with:

- Protocol No.

- Subject No.
- Time of study drug administration
- Sampling time (theoretical) (Visit, Date, Time).
- Sampling time (actual time).
- Storage conditions.

Thereafter, the samples will be stored at –80 ± 10°C until the shipment to the bioanalytical centre.

Two (2) series of tubes (serum samples) will be transferred in two different shipments to:

[redacted]

Bioprojet Biotech

4 rue du Chesnay Beauregard,

BP96205, 35762-Saint Grégoire, France

[redacted]

And the third series of tubes will be stored at the clinical site and can be sent to the bioanalytical center if necessary.

Before any shipment, the site monitor will confirm by appropriate mean of communication (email or fax) to [redacted] the exact dates and hours of each consignment of samples. Only after receipt of [redacted] agreement, the site monitor will organize the shipment. The shipment of the samples will have to be organized at least 2 days before any official holiday or week-end.

The study monitor will ensure samples will be sent in boxes containing enough dry ice for a period of at least 72 h. A control of temperature will be done upon receipt of the samples by checking the amount of remaining dry ice and if the samples are in a frozen state.

The samples will be registered according to the SOP used in Bioprojet Biotech. The biological samples will be stored at –80 ± 10°C until analysis. The biological samples will be handled using gloves to prevent any risk of contamination.

The analysis of the study samples will be described in a separate analytical protocol and reports (Bioprojet Biotech study code B410).

Serum levels of BF2.649/BP2.951, BP1.8054 and BP1.9733 will be evaluated using validated LC/MS/MS methods (Bioprojet-Biotech study code B258, B349 and B387, respectively). The limits of quantification will be 0.1, 1 and 1 ng/ml, respectively.

10.2.14.10. Overdosing

Rules of conduct in case of pitolisant overdosage:

- From 120 mg onwards, in one single intake in adults, it is recommended to keep the patients under medical supervision.
- From 200 mg onwards, it is preferable to have them hospitalized.

In addition to this treatment, a monitoring of the vital functions shall be performed and a H_2 antihistamine product, such as Tagamet®, shall be administered in case of gasteralgias.

10.2.14.11. Observance of nightly nCPAP use

The observance of nCPAP will be assessed by the clock-time counter of the CPAP machine at each visit (reading of the last day) prior to each visit (for patients submitted to nCPAP therapy).

11. EMERGENCY PROCEDURES

11.1. Sponsor contact

In emergency situations, the investigator may contact the Sponsor representative by telephone at the number listed on the title page of the protocol.

Pharmacovigilance Responsable contact: [redacted] Pharm.D., Ph.D.

Bioprojet – 9, Rue Rameau, 75002 Paris, France
[redacted] m

The Medical Project Manager: [redacted]

Bioprojet – 9, Rue Rameau, 75002 Paris, France
[redacted]

11.2. Emergency identification of Study Products

Unblinding envelopes (see 9.2.9.3) must only be opened in case of emergency (i.e. in case an imperative need to know the assigned treatment by the investigator to treat the patient adequately in case of serious adverse event or in case of overdosage or study drug misused, or in the event of death, or when requested by a regulatory agency in the case of a serious adverse event).

The code breaking will be documented on the envelope and in the CRF, with the date, the time and the reasons of breaking and will be signed by the investigator. Before opening the envelope, the investigator will contact the study monitor or medical project manager to explain the situation and validate the need to break the code. Only under extreme emergency situation, the investigator will break the code without authorization. In this case, information on code breaking will be provided to the clinical research associate (CRA) or medical project manager as soon as possible. Information related to the actual treatment given during the double blind period should not be provided to any members of the study team. When a code is broken for a patient, the patient must be withdrawn from the study. In this case an early withdrawal visit and the end of study form must be completed and recorded in the CRF.

12. CONDUCT OF THE TRIAL

This is a prospective, multicenter (several sites in Europe), randomised, double blind study versus placebo.

The study population will be recruited from patients who have a current confirmed diagnosis of OSA, having refused the nCPAP therapy or treated by nCPAP, and who still complain of EDS. Patients should not have important cardiovascular risk factors.

After the Double Blind period**,** patients may optionally, and once a new informed consent form being signed, enter the 40-week Open Label Extension period of the study consisting in an escalating-dose of the same regimen than in the 1st part of the study, followed by a selected dose phase, with the active drug only, until week 53 after the start of the treatment.

The study will include about 400 patients. This number will allow having sufficient completed treated patients – 360 (pitolisant = 240; placebo = 120) for the per protocol analysis of efficacy during the Double Blind period (12 weeks).

The maximum duration of the study will not exceed 55 weeks (2 weeks for baseline without treatment, 12 weeks for the first Double Blind period, 1 week wash-out period with placebo after V6, 39 weeks for the Open Label Extension period and a 1 week-wash out period without placebo after V12). The patient will be ambulatory during the whole study period except staying 1 optional night in the sleep laboratory for the full recording of nocturnal polysomnography (if it was not performed during the last twelve months).

The timeline of examinations and tests to be performed at each visit are indicated in section 2 (study diagram), concerning the patients withdrawing their participation in the study after the Double Blind period as well as for patients pursuing the Open Label Extension period.

The date of each visit could be D ± 3 Days.

12.1. Part I - Double Blind period

12.1.1. V1 – Screening visit and beginning of initial wash-out period (D -14)

Before proposing the study to patients refusing to be treated by nCPAP, the investigator will ensure that the patient still refuses this treatment.
If the patient may be enrolled in the study, the investigator has to give him/her the necessary information which will be made orally and by giving the specific patients' informed consent form. Objective, treatments, expected benefit, potential risks, constraints and procedures of the study will be carefully explained to the patient, as well as the alternative available treatments. An adequate delay for the patient to decide if he/she is willing to participate in this study will be respected. An appropriately signed informed consent will be obtained prior to entry into the study for each patient having made a positive decision to participate.

It is only after the patient accepts to be enrolled into the study that any screening tests and examinations can be performed.

During this screening visit, the investigator must confirm that the patient meets all inclusion criteria and none of the non-inclusion criteria, and that his/her state of health allows him/her to discontinue prohibited treatments.

Baseline history and examination will be obtained in order to collect relevant data prior to the beginning of the study.

The investigator will perform the following screening assessments:
- Medical questionnaire including the patient demographic data, OSA and EDS history and

documentation of the previously performed tests/questionnaires about illness (such as polysomnography, AHI, Epworth score, OSleR test, BMI, state of sleep-related desaturation), actual symptoms prior to admission and their duration, possible complications, details of concomitant treatments including any current and previous treatments of EDS and any other types of treatment.

- For patients using nCPAP therapy, when possible, the observance prior to screening will be assessed by the clock-time counter of the CPAP machine for a minimum period of 3 months. If available, a printout report should be archived in the patient's medical file. Otherwise it should be documented in the patient's chart through the patient's medical interview during the screening visit.
- A complete physical examination, in particular vital signs including blood pressure, heart rate, respiratory rate.
- Epworth Sleepiness Scale (ESS). Patients with a score of ESS<12 will not be included in trial.
- The Mini Mental State Examination (MMSE); an example of MMSE questionnaire is provided in Appendix 16. All patients with a score of MMSE < 28 will not be included in the trial.
- Clinical Global Impression – illness severity (CGI-S).
- BDI-13 will be performed to assess the state of depression, and all patients with a score of BDI-13 ≥ 16 and item G (suicidal ideation) positive will be withdrawn from the trial.
- ECG.
- Clinical laboratory tests: biological (hematology biochemistry), serological (β-HCG pregnancy test for woman with child-bearing potential, HIV, HCV, HBsAg) tests and urinalysis (stick test with microscopy and bacteriological culture, if positive). Laboratory test results will be reviewed prior to inclusion in the study.

All these data have to be reported in the CRF and compared to the inclusion and non-inclusion criteria. Only patients who fulfil all inclusion and exclusion criteria and sign the informed consent could be enrolled in this study.

If enrolled, the patient will have to stop his/her current medication against EDS for a two week wash-out period.

Sleep diary will be delivered. The patient will answer to the sleep diary questions during 3 sequential days of the week preceding next visit. An appointment will be fixed for the inclusion

visit (V2) 2 weeks later.

An appointment for a one-night polysomnography (see Appendix 2) will be planned between V1 and V2, if it was not performed during the last twelve months.

The patient will be reminded to contact the investigator between the visits for any issue, such as adverse events, difficulties with the treatment, need for a new treatment, associated pathology, and modification of the current concomitant treatment (applicable for the whole study duration).

12.1.2. Ph1 – Phone contact (D -7)

During this phone contact, the investigator should check if patient has discontinued previously used treatment for EDS and the intake of all prohibited treatments. Also other concomitant treatments and occurrence of AEs will be checked.

12.1.3. V2 – Inclusion visit and beginning of the Double Blind period: baseline examinations, randomisation, start of escalating dose phase (D 0)

The following assessments will be performed during this visit:

- Medical questionnaire.
- Physical examination, in particular vital signs of the patient.
- ESS.
- Three sessions of Oxford Sleep Resistance test (OSleR test) at approximately 09:00, 11:00 and 13:00.
- Trail Making Test (TMT) parts A and B.
- CGI-S.
- BDI-13.
- Leeds sleep evaluation questionnaire (LSEQ).
- European Quality of Life Questionnaire (EQ-5D).
- Pichot Fatigue Scale.
- ECG.
- Review of V1 laboratory tests results.
- Analysis of the polysomnography report performed in the 12 previous months before V1 or

performed in between V1 and V2.

- Review of the sleep diary.
- Investigation of any adverse event occurrence.
- Verification that the patient has discontinued the intake of all prohibited treatments.
- Confirmation of the inclusion of the patient in this study after verification of all inclusion and non-inclusion criteria.

If the patient is included in this study, he/she will be randomized to either pitolisant, or placebo and receive the experimental treatment which is initiated by an individual titration period over 2 consecutive weeks. Depending on the randomization, the treatment assigned to each patient during the first week will be 10 mg pitolisant or placebo. During the second week, it will be 20 mg pitolisant, or placebo.

Two first medication bottles identified "1st week" and "2nd week" will be given to the patient for the 2 weeks. Patient will be instructed to take, starting from the next day after the visit, 2 tablets from corresponding bottle on the 1st week and 1 tablet form the corresponding bottle on the 2nd week every morning, during breakfast. It is important to ensure that the patient does not forget to take the treatment on the day of the next visit.

Sleep diary will be delivered. The patient will answer to the sleep diary questions three sequential days of the week preceding next visit. The next visit will be fixed 2 weeks later, and the patient should bring back the completed diary and the used experimental treatment bottles.

The total treatment period in double-blind is 12 weeks.

12.1.4. V3 – First dose adjustment visit (D 14)

The visit will imperatively take place in the morning in order to ensure the identical conditions for evaluation. It is important to ensure that the patient does not forget to take the treatment on the day of the visit.

The following assessments will be performed during this visit:

- Medical questionnaire including control of the treatment compliance by counting the remaining tablets and verification of the forgotten treatment intake.
- Physical examination, in particular vital signs of the patient.
- ESS.
- Patient's overall evaluation of the tolerance.

- ECG.
- Review of the sleep diary.
- Investigation of any adverse event occurrence.

Once the investigator has ascertained that the tolerance of the study product is acceptable by performing clinical examination, questionnaire to check adverse event and ECG, the posology is increased to 40 mg/d pitolisant (high dose) or placebo, every morning, during breakfast.

If the tolerance does not allow it (occurrence of an adverse event i.e. troublesome insomnia), the patient will continue the treatment intake at 20 mg/d pitolisant (medium dose) or placebo or the posology will be eventually reduced at 10 mg/d pitolisant (low dose) or placebo until V4.

One corresponding bottle will be given to the patient, and he/she will be instructed by the investigator to take, depending on the prescribed dose, 1 or 2 tablets every morning, during breakfast. It is important to ensure that the patient does not forget to take the treatment on the day of the next visit.

Sleep diary will be delivered. The patient will answer to the sleep diary questions on three sequential days before the next visit, which will be fixed in 1 week. The patient should bring back the completed diary and the used experimental treatment bottle.

12.1.5. V4 – Second dose adjustment visit and beginning of stable dose phase (D 21)

After the 1-week treatment period at the selected dose, the investigator will check the efficacy and safety at this visit.

The visit will take place in the morning to ensure the identical condition for evaluation. It is important to ensure that the patient does not forget to take the treatment on the day of the visit.

The following assessments will be performed during this visit:

- Medical questionnaire including control of the treatment compliance by counting the remaining tablets and verification of the forgotten treatment intake.
- Physical examination, in particular vital signs of the patient.
- ESS.
- Patient's overall evaluation of the tolerance.
- ECG.

- Review of the sleep diary.
- Investigation of any adverse event occurrence.

The dose of study treatment will be individually adjusted according to the assessment of investigators on the basis of tolerance of the study treatment in accordance with the following scheme.

The posology is maintained for the next following 4 weeks.

But, if the tolerance does not allow it:

- patients taking 40 mg/d pitolisant (high dose) or placebo could reduce to 20 mg/d pitolisant (medium dose) or placebo for the following 4 weeks.
- patients taking 20 mg/d pitolisant (medium dose) or placebo could reduce to 10 mg/d pitolisant (low dose) or placebo or placebo for the following 4 weeks.

Any dose increase will not be allowed at this visit.

After V4 until the end of Double Blind period any dosage change of pitolisant or placebo will not be allowed. Hence, pitolisant, or placebo treatment under stable doses will be continued for 9 weeks.

Four corresponding bottles (one per week) will be given to the patient, and he/she will be instructed by the investigator to take, depending on the prescribed dose, 1 or 2 tablets every morning, during breakfast. It is important to ensure that the patient does not forget to take the treatment on the day of the next visit.

Sleep diary will be delivered. The patient will answer to the sleep diary questions on three sequential days the of week preceding next visit. The next visit will be fixed in 4 weeks and the patient should bring back the completed diary and the used experimental treatment bottles.

12.1.6. V5 – Control visit: continuation of stable dose phase (D 49)

After the 4-week treatment period at the selected dose, the investigator will evaluate the efficacy and safety.

The visit will take place in the morning to ensure the identical condition for evaluation. It is important to ensure that the patient does not forget to take the treatment on the day of the visit.

The following assessments will be performed during this visit:

- Medical questionnaire including control of the treatment compliance by counting the remaining tablets and verification of the forgotten treatment intake.

- Physical examination, in particular vital signs of the patient.
- ESS.
- Patient's overall evaluation of the tolerance.
- ECG.
- Review of the sleep diary.
- Investigation of any adverse event occurrence.

Any dosage change of pitolisant or placebo will not be allowed at this visit. Hence, pitolisant or placebo treatment at the same stable dose will be continued for another 5 weeks.

Five corresponding bottles (one per week) will be given to the patient, and he/she will be instructed by the investigator to take, depending on the prescribed dose, 1 or 2 tablets every morning, during breakfast. It is important to ensure that the patient does not forget to take the treatment on the day of the next visit.

Sleep diary will be delivered. The patient will answer to the sleep diary questions on three sequential days of the week preceding next visit. The next visit will be fixed in 5 weeks and the patient should bring back the completed diary and the used experimental treatment bottles.

12.1.7. V6 – Evaluation visit at the end of the Double Blind period and start of single-blindwash-out period (D 84)

At this visit the patients are submitted to the tests and examinations required for the analysis of the double blind study results.

The patient will be required to come early in the morning at the hospital. It is important to ensure that the patient does not forget to take the treatment on the day of the visit.

The following assessments will be performed during this visit:

- Medical questionnaire including control of the treatment compliance by counting the remaining tablets and verification of the forgotten treatment intake.
- Physical examination, in particular vital signs of the patient.
- ESS.
- Three sessions of OSleR test at approximately 09:00, 11:00 and 13:00.
- TMT parts A and B.
- Clinical Global Impression – illness improvement or change (CGI-C).

- BDI-13.
- LSEQ.
- EQ-5D.
- Pichot Fatigue Scale.
- Patient's global opinion on the effect of investigational drugs.
- Patient's overall evaluation of the tolerance.
- ECG.
- Clinical laboratory tests: biological (hematology biochemistry) and serological (β-HCG pregnancy test for woman with child-bearing potential) tests.
- Review of the sleep diary.
- Investigation of any adverse event occurrence.

The Double Blind period of this study will be completed throughout this visit Patients will start one-week single blind placebo wash-out period.

One corresponding bottle will be given to the patient, and he/she will be instructed by the investigator to take, depending on the prescribed dose, 1 or 2 tablets every morning, during breakfast. It is important to ensure that the patient does not forget to take the treatment on the day of the next visit.

Sleep diary will be delivered. The patient will answer to the sleep diary questions on three sequential days. The next visit will be fixed in 1 week and the patient should bring back the completed diary and the used experimental treatment bottle.

12.1.8. Ph2 – Phone contact (V6 + 3D)

During this phone contact, the investigator should get patient's answers to Amphetamine-Like Withdrawal Symptoms Questionnaire (DSM IV) to check the tolerance of the study drug withdrawal and find out patient's global opinion on the effect of investigational drugs. Also concomitant treatments and occurrence of AEs will be checked.

12.2. Part II - Open Label Extension period

12.2.1. V7 – End of study visit for patients who are not entering Open Label Extension period or Beginning of the Open Label Extension period for

patients who are entering Open Label Extension period: start of new escalating dose phase (D 91)

The visit will take place in the morning to ensure the identical condition for evaluation. It is important to ensure that the patient does not forget to take the treatment on the day of the visit.

The following assessments will be performed during this visit:

- Medical questionnaire including control of the treatment compliance by counting the remaining tablets and verification of the forgotten treatment intake.
- Physical examination, in particular vital signs of the patient.
- ESS.
- Three sessions of OSleR test at approximately 09:00, 11:00 and 13:00 (only in patients entering OpenLabel Extension phase).
- TMT parts A and B (only in patients entering OpenLabel Extension phase).
- CGI-C.
- BDI-13.
- LSEQ (only in patients entering OpenLabel Extension phase).
- EQ-5D (only in patients entering OpenLabel Extension phase).
- Pichot Fatigue Scale.
- Patient's global opinion on the effect of investigational drugs.
- Patient's overall evaluation of the tolerance.
- Amphetamine-like withdrawal symptoms questionnaire (DSM IV).
- ECG.
- Review of V6 laboratory tests results.
- Review of the sleep diary.
- Investigation of any adverse event occurrence.

If the patient holds the same position towards nCPAP therapy as before, he/she will be proposed to enter the Open Label Extension period of the study after another escalating dose phase.

Patients not willing to continue the pitolisant treatment administration will end their participation in the study at this visit.

Patients willing to continue the pitolisant treatment administration will be given another information leaflet explaining the constraints and risks of the Open Label Extension study

period together with an informed consent form to be signed. As soon as the patient gives the agreement by signing the informed consent form, he/she will be enrolled in this long-term follow-up period and will start the new escalating dose phase, as described hereunder.

Patients will receive one bottle containing 20 tablets of pitolisant 5 mg and one bottle containing 10 tablets of pitolisant 20 mg.

Patients will be instructed to take 2 tablets of pitolisant 5 mg OD during one week and 1 tablet of pitolisant 20 mg the next week OD (in the morning, during breakfast, with a glass of water). It is important to ensure that the patient does not forget to take the treatment on the day of the next visit.

Sleep diary will be delivered. The patient will answer to the sleep diary questions on three sequential days of the week preceding next visit. The next visit will be fixed in 2 weeks and the patient should bring back the completed diary and the used experimental treatment bottles.

12.2.2. V8-V9 – Dose adjustment visits (D 105 and D 112)

The visits will imperatively take place in the morning in order to ensure the identical conditions for evaluation. It is important to ensure that the patient does not forget to take the treatment on the days of the visits.

The following assessments will be performed during these visits:

- Medical questionnaire including control of the treatment compliance by counting the remaining tablets and verification of the forgotten treatment intake.
- Physical examination, in particular vital signs of the patient.
- ESS.
- BDI-13 (at V9).
- Pichot Fatigue Scale (at V9).
- Patient's overall evaluation of the tolerance.
- ECG.
- Review of the sleep diary.
- Investigation of any adverse event occurrence.

At each of the visits V8-V11 the investigator will propose nCPAP to the patients refusing to be treated by nCPAP therapy before giving them the study treatment. If the patient accepts, he/she

will be withdrawn from the study.

The posology is increased to 40 mg/d pitolisant (high dose), every morning, during breakfast. If the tolerance does not allow it (i.e. troublesome insomnia), the patient will get the treatment at dose of 20 mg/d (medium dose) or 10 mg/d (low dose). At V9, "one-step" decrease from high dose to low dose or increase from low dose to high dose is not possible.

At V8, 1 bottle containing 20 tablets of pitolisant 5 mg, or 1 bottle containing 10 tablets of pitolisant 20 mg, or 1 bottle containing 20 tablets of pitolisant 20 mg will be given to the patient; at V9 bottles containing 30 tablets of pitolisant 5 mg or 30 tablets of pitolisant 20 mg, depending on selected posology, will be given to the patient in the quantity needed for prescribed treatment., The patient will be instructed by the investigator to take, depending on the prescribed dose, 1 or 2 tablets every morning, during breakfast. It is important to ensure that the patient does not forget to take the treatment on the next visit days.

Sleep diaries will be delivered. The patient will answer to the sleep diary questions on three sequential days. The next visit will be fixed in 1 week (V9), then in 12 weeks (V10), and the patient should bring back the completed diary and the used experimental treatment bottles.

12.2.3. V10-V11 – Confirmed dose visits (D 196 and D 280)

The visits will occur in the morning, and the patient should be reminded not to forget to take the treatment on the visit days.

The following assessments will be performed during these visits:

- Medical questionnaire including control of the treatment compliance by counting the remaining tablets and verification of the forgotten treatment intake.
- Physical examination, in particular vital signs of the patient.
- ESS.
- CGI-C.
- BDI-13.
- Pichot Fatigue Scale.
- Patient's global opinion on the effect of investigational drugs.
- Patient's overall evaluation of the tolerance.
- ECG.

- Review of the sleep diary.
- Investigation of any adverse event occurrence.
- Pharmacokinetics sampling (at V10) – predose, 1,5, 3 and 8 h post administration.

The posology should be 40 mg/d (high dose) if the tolerance is acceptable. If the study drug is not well tolerated, the investigator may decide to decrease the dose to 20 mg/d (medium dose) or 10 mg/d (low dose) during the 12-weeks period following the visit. "One-step" decrease from high dose to low dose or increase from low dose to high dose is not possible.

At each visit bottles containing 30 tablets of pitolisant 5 mg or 30 tablets of pitolisant 20 mg, depending on selected posology, will be given to the patient in the quantity needed for prescribed treatment, and he/she will be instructed by the investigator to take, depending on the prescribed dose, 1 or 2 tablets every morning, during breakfast. It is important to ensure that the patient does not forget to take the treatment on the next visit days.

Sleep diaries will be delivered. The patient will answer to the sleep diary questions on three sequential days the of the week preceding next visit. The next visits will be fixed with 12 weeks interval and the patient should bring back the completed diary and the used experimental treatment bottles.

12.2.4. V12 – Evaluation visit at the end of the Open Label Extension period and start of wash-out period (D 364)

At this visit the patients are submitted to the examinations and tests required for the evaluation of long-term tolerance and maintenance of efficacy of pitolisant.
The patient will be required to come early in the morning at the hospital. It is important to ensure that the patient does not forget to take the treatment on the day of the visit.

The following assessments will be performed during this visit:

- Medical questionnaire including control of the treatment compliance by counting the remaining tablets and verification of the forgotten treatment intake.
- Physical examination, in particular vital signs of the patient.
- ESS.
- Optionally: three sessions of OSleR test at approximately 09:00, 11:00 and 13:00.
- TMT parts A and B.
- CGI-C.

- BDI-13.
- LSEQ.
- EQ-5D.
- Pichot Fatigue Scale.
- Patient's global opinion on the effect of investigational drugs.
- Patient's overall evaluation of the tolerance.
- ECG.
- Clinical laboratory tests: biological (hematology biochemistry) and serological (β-HCG pregnancy test for woman with child-bearing potential) tests.
- Review of the sleep diary.
- Investigation of any adverse event occurrence.

The Open Label Extension period of the study will be completed at this visit; the treatment intake will be stopped, patients will not receive any more experimental treatment in this study.

Sleep diary will be delivered. The patient will answer to the sleep diary questions on three sequential days. The next visit will be fixed in 1 weeks and the patient should bring back the completed diary.

12.2.5. Ph3 – Phone contact (V12 + 3D)

During this phone contact, the investigator should get patient's answers to amphetamine-like withdrawal symptoms questionnaire (DSM IV) to check the tolerance of the study drug withdrawal and find out patient's global opinion on the effect of investigational drugs. Also concomitant treatments and occurrence of AEs will be checked.

12.2.6. V13 – End of study visit for patients who entered into Open Label Extension period (D 371)

After a one-week wash-out period, this final visit will occur in the morning.

The following assessments will be performed during this visit:

- Medical questionnaire.
- Physical examination, in particular vital signs of the patient.
- ESS.

- CGI-C.
- BDI-13.
- Pichot Fatigue Scale.
- Patient's global opinion on the effect of investigational drugs.
- Patient's overall evaluation of the tolerance.
- Amphetamine-like withdrawal symptoms questionnaire (DSM IV).
- ECG.
- Review of V12 laboratory tests results.
- Review of the sleep diary.
- Investigation of any adverse event occurrence.

Thereafter, the investigator will decide about the new treatment to prescribe for the patient. And then the patient will be discharged from the study.

13. STATISTICAL ANALYSIS

13.1. Summary

Multicenter, randomised, double blind parallel groups study versus placebo during 3 months followed by a 9 months of Open Label Extension period with pitolisant.

The Full Analysis Set (FAS) constitutes the main selection, Per Protocol (PP) selection will be used as a secondary selection.

The main purpose of the study is to assess efficacy of pitolisant in decreasing daytime somnolence measured by the Epworth Sleepiness Scale. The secondary endpoints will include OSleR, and a Z-score aggregating OSleR and ESS.

Statistical Analysis: final ESS will be compared between the two treatments by an ANCOVA in adjusting for ESS at baseline, and by considering the random effect center, and the fixed effect treatment. This test will be implemented by a Mixed Linear Model. This analysis will be conducted both on the CPAP and non-CPAP groups as primary selections.

Safety and tolerability will be assessed by summarizing and analyzing adverse events, change in physical examination, vital signs, electrocardiogram and laboratory data.

13.2. Sample Size Determination and Justification

Results from exploratory studies on pitolisant allow to estimate the ESS residual variability to standard deviation (SD) = 6. The Minimum Important Difference (MID) was fixed to ESS = 3, corresponding to an effect size (ES) = 0.5. The correlation between final and baseline ESS was conservatively estimated to r = 0.3.

a) By assuming ANCOVA at 0.95 confidence level as the main confirmatory test, and a sample ratio 1:2, a difference of at least Delta = 3 should be detected with a power of 90% in using at least 60 patients in each placebo group and 120 patients in each pitolisant treatment group.
b) By assuming the same model, an interaction of at least 3 between CPAP and non-CPAP will be detected with a power of 90% when 120 and 240 patients (thus 360 patients in total) are treated in Placebo and pitolisant group, respectively. The null hypothesis here should be

   Interaction $(Y_{pt}\text{-}Y_{pl})_{CPAP}$ - $(Y_{pt}\text{-}Y_{pl})_{non\text{-}CPAP} > 3$

Under these two conditions, a stepdown test can be organized: First a test of superiority of pitolisant will be conducted. If this test concludes into superiority, the interaction test will be conducted.

The sample size will be 120 and 240 (thus at least 360 patients in total) as this size corresponds to the interaction test requiring the largest sample size. Considering 10% drop out rate, approximately 400 patients will be selected.

13.3. Primary and Secondary Populations

The primary population will be the Full Analysis Set (FAS) in conformity with intent to treat principle: All the randomized patients will be analyzed, irrespective of their outcome. The Per Protocol Analysis is defined as the subset of the FAS set constituted by the patients compliant to the regimen and finishing on time.

13.4. Handling of Missing Data

The main endpoint will be calculated as the summary mean of the non missing values of ESS at visits 5 and 6. Missing values will be imputed using a pattern mixture model based on the assessment of the interaction between the variables completers/dropout linear model and

treatment on the main endpoint. Dropouts before visit 5 will be imputed by baseline value when the patient is withdrawn from the trial for any reason related with drug adverse effects, or a manifest lack of efficacy, or Last Observed Carried Value, in any other case. In any case, this decision will be taken by the Scientific Committee.

13.5. Futility Analysis

During the first part of the study, a one-stage Futility stopping was to be done based on Conditional Power, probability to detect a significant result at the end of the Double Blind period, given the results observed at an intermediate time. This analysis was done between pitolisant and placebo irrespective of CPAP use.

Conditional Power will be estimated (Lan and Wittes 1988; Lan and Zucker 1993). This analysis was carried out by a third party statistician when at least 80 patients are available, and futility threshold will be CPmin=.10 involving a slight increase of type 2 error (Proschan 1999). This intermediate futility analysis did not require any type-I adjustment, this trial did not plan rejection of the null hypothesis before its end.

In the new context of the study, the question remains on the significance of the difference within each subgroup. A one-stage Futility stopping might be conducted. In this case the analysis should be based on the Conditional Power of the interaction test, probability to detect a significant result at the end of the study, given the results observed at an intermediate time. Conditional Power (CP) will be estimated through B-values and (Lan and Wittes 1988; Lan and Zucker 1993). This analysis will be carried out by a third party statistician when at least 180 patients are available, and futility threshold will be CPmin=.10 involving a slight increase of type 2 error (Proschan 1999). This intermediate futility analysis does not require any type-I adjustment, this trial does not plan rejection of the null hypothesis before its end.

Further details will be described in the Statistical Analysis Plan (SAP) as appropriate.

An independent DSMB will regularly follow the progress of the clinical trial, monitor safety data and critical efficacy variables, and be consulted concerning the opportunity of modifying the sample size or terminate the trial for futility.

13.6. Statistical Analysis

The main endpoint will be calculated as the summary mean of the non missing values of ESS at visits 5 and 6. Final ESS will be compared between the two treatments by an ANCOVA at

two-sided 95% in adjusting for ESS at baseline (mean of ESS of visits 1 and 2), and by considering the random effect center, and the fixed effect treatment and assessing the additional effect of obesity (BMI) on outcome[17, 18]. This test will be implemented by a Mixed Linear Model.

The confirmatory analysis will be based on a simple ANCOVA model assuming no interaction between baseline and treatment (assumption of parallelism).

13.7. Pharmacokinetic Analysis

The pharmacokinetic analysis will be based on a population approach.

The proposed PK sampling scheme was optimized according to a previous knowledge on pitolisant PK and an initial population PK model developed in healthy subjects. Parameters of this initial population model were used together with study constraints (i.e. limitation of the number of PK blood samples and PK follow-up limited to a maximum of 8 h post-dose). The PFIM software was used for sampling time optimization and determination of population sample size for valuable analysis.

An optimized PK blood sampling times of 4 samples requiring a minimum population of 90 patients was defined to be applied in the present study.

Pitolisant concentrations will be populated with dosing information data and selected covariates in order to be analysed using NONMEM® software for nonlinear mixed model.

The population PK model will be composed of:

- a structural part, defining the general pattern of concentrations time course, parameterized in terms of apparent clearance, volume of distributions, etc.
- a random part, split in inter and intra-individual variability
- a covariate model in which influence of covariates on PK parameters and variability will be investigated.

Methods to be used for the population PK analysis will be fully detailed in a specific analysis plan.

14. DATA MANAGEMENT

14.1. Collection of data

The data of each patient will be collected in a CRF bearing the inclusion number which is the patient number in the study. The following data will be collected:

- Demographic data: age, weight, height, birth date (month/year), medical history
- Results of patient physical examinations, including vital signs
- Results of ESS
- Results of OSleR test
- Results of TMT Parts A & B
- Results of MMSE
- Results of CGI-S and CGI-C
- Results of BDI-13
- Results of LSEQ, EQ-5D and Pichot Fatigue Scale
- Patient’s global opinion on the effect of investigational drugs
- Patient’s overall evaluation of the tolerance
- Results of amphetamine-like withdrawal symptoms questionnaire (DSM IV)
- ECG
- Results of laboratory tests
- Results of the polysomnographic examination
- Records of sleep diary
- Adverse Events reports
- Administered treatment
- Compliance to treatment and to nCPAP (for patients on nCPAP therapy)

Data collected in the CRFs will be transferred to electronic database.

The database will be validated through computer comparison of the two files and by tests of coherence with computer software, according to the data management book (DMB).

14.2. Archiving of data, Audit

In order to constitute evidence with respect to product safety or regulatory or legal compliance, the investigators, investigational sites and Ethics Committees agree to retain study-related documents in a location that is secure and to which access can be gained if required.

The investigator and the site should retain records for a minimum of 15 years or a delay according to the local regulatory requirement.

At the end of these regulatory timelines, the investigator will inform Bioprojet of his intention to proceed with the destruction of archived data.

If the records need to be retained after that duration, the investigator and the site will be notified by Bioprojet.

These documents are to be available for inspection by authorized representative of Bioprojet or regulatory authorities. Audits may be performed for quality assurance of data handling.

The following records must be retained by the investigator for a minimum of 15 years or a delay according to the local regulatory requirement, after the sponsor has notified the Authorities that the study is completed:

- Signed informed consent documents for all patients
- Patient identification code list and enrolment log
- Record of all communications between the investigator and the EC/IRB
- Composition of the EC/IRB (or other local applicable regulation)
- Record of all communications between the investigator and sponsor (or CRO)
- List of sub-investigators and other appropriately qualified persons to whom the investigator has delegated significant trial-related duties, together with their roles in the study and their signatures
- Copies of case report forms and of documentation of corrections for all patients
- Drug accountability record
- All other source and essential study documents (patient records, hospital records, laboratory records, etc...)

The records should be held in the investigator's archives. However, if the investigator is unable to meet this obligation, he or she must ask the sponsor for permission to make alternative arrangements. Details of these arrangements should be documented.

14.3. Data Protection and Confidentiality

All documents that concern the studied medication and the company's operations belonging to sponsor such as patent applications, formula, manufacturing process, basic scientific data and analysis bulletins; information supplied by the company and not previously published are considered confidential and shall remain the sole property of the sponsor.

The investigator agrees to use this information only in accomplishing this study and they will not use it for other purposes without written consent from sponsor.

Confidentiality of study source documents

The information included in this document, as the investigator's brochure of the product, the CRF and the results of the present study are considered as confidential and should not be divulged, only in case of legal requirements.

In any event, persons to whom the information is disclosed must be informed that the information is privileged or confidential and may not be further disclosed by them. The signature of investigator in the present protocol is equivalent to a confidential agreement.

It is understood by the investigator that the information from the clinical study will be used by the company in connection with the development of the tested drug and, therefore, may be disclosed as required to other clinical investigators or to government agencies. In order to allow for the use of the information derived from the clinical studies, it is understood that there is an obligation to provide the sponsor with the complete test results as well as all data developed during this study, under the form of a written document or computerized with the following software: Word or SAS under Windows, saved on CD-Rom.

The study drug and the information in this document and in any future information supplied contain trade secrets and commercial information that are privileged or confidential and may not be disclosed unless such disclosure is required by law or regulations.

All or part of the information should only be divulged, submitted for publication or claim for industrial proprietary act with the written consent of Bioprojet.

According to the French law "Computer Information and Liberty", dated January 6th, 1978 modified by the law N°2004-801 of August, 6th 2004 and the decree N°2005-1309 of October 20th 2005, Bioprojet committed on July 28th, 2006 to comply with the methodology MR001 for all electronic and computerized data relative to this study .

15. QUALITY ASSURANCE

15.1. Good clinical Practice

The trial will be run with respect to this protocol, according to the Good Clinical Practice (GCP), following the international regulations (ICH) and the European directives (EMEA) or national laws.

The quality control of the study and the audit/inspection of the Good Clinical Practice could be performed by Bioprojet and its Department of Quality Assurance or by an inspector of the Ministry of Health.

In particular, the current protocol will be submitted to the agreement of the Ethics Committee and Competent Authority by Bioprojet. The conformity of the study progress will be reviewed.

In case of an amendment, it has to be submitted for agreement to the Ethics committee and Competent Authority as appropriate. The Ethics Committee will be consulted through notification for any minor protocol modification.

In case of major written modification (modification that could jeopardize the protection of the people participating to the trial and modifications likely to invalidate the scientific validity of the study), the protocol will be re-submitted to the agreement of the Ethics Committee and Competent Authority.

The investigator will carefully explain the participating conditions to each proposed patient.

Each patient will receive a detailed and written information letter including the name of the product, a summary of its properties, its potential benefit, the unexpected and adverse events, the doses to be administered, the treatment duration, the number of visits, the kind and number of scheduled exams. This information letter will be submitted beforehand to the agreement of the Ethics committee.

The patient will be informed that he can dropout from the study whenever he wants, without any justification.

The system to attribute numbers to patients is based on the anonymity obligation. Patients will receive a number according to their order of inclusion in the study.

The patients will be identified by the sponsor with: the patient number and their date of birth.

Study progress conformity to the protocol will be controlled from the patients' selection, and all along the study. Each investigator has to allow the monitor, or sponsor's mandated representative, the direct access to any control of the study progress, and to any required

documents to control data reported in case report forms (hospitalization file, consultation file, results of additional examinations, etc.).

The medical project manager of the study will be reachable, weekend included, at:

PPDPPDPPD
Bioprojet – 9, Rue Rameau – 75002 Paris, France
PPDPPDPPDPPDPPDPPDPPDPPD
PPDPPDPPDPPDPPD

The investigator will be available to the telephone for each patient whom he will have included.

The anonymity respect shall be applied to the filling of the case report form as to any other archived documents considered as source data (blood tests, informed consent form, etc.).

15.2. Premature closure of the study

The sponsor or the investigator has the right to close this study at any time for valid scientific or administrative reasons, and reasons related to patients' protection. As far as possible, this should occur after mutual consultation. The Competent Authorities (CA) and the EC/IRB must be informed, if required by legislation.

Should the study be closed prematurely, all study materials (completed, partially completed, and blank case report forms, study medication, etc.) must be returned to the sponsor, as if the study had been completed.

15.2.1. Criteria for terminating the trial

Reasons for the study termination may include but are not limited to:

- The discovery of an unexpected, significant or unacceptable risk to patients enrolled in the study
- A decision of Bioprojet to suspend or discontinue the development of the investigational product
- A decision of Bioprojet to suspend after analysis of futility
- Request of the relevant regulatory agency

15.2.2. Criteria for terminating an investigational site

Bioprojet reserves the right to terminate the study at a given investigational site at any time

after the study initiation if:

- ICH GCP regulations are not fulfilled
- The protocol is violated without justification
- The data generated are of poor quality

15.3. Control – Quality

Control actions are implemented within the framework of the Quality Insurance System to check that the quality requirements of the study are respected.

The original documents generated in the course of the study will be controlled at each step of the study, both by the sponsor's representative and the investigator, in order to guarantee the accuracy of the analyzed data.

Internal audit systems can be conducted during the study by the sponsor's representative or by an independent organism.

They will enable to check that the study is being run in accordance with the protocol and to current rules and regulations.

16. REPORTING AND PUBLICATION OF RESULTS

The investigator commits himself to:

- Keep the Sponsor (Bioprojet Medical project manager) posted on the study results in written form.
- Review and sign the clinical report of the study (ICH format) for Bioprojet.
- The communication or publication of all or part of the results of this study will be only permitted after written agreement of Bioprojet. The publication or communication shall mention the origin of the substance.

17. CONTRACT, LIABILITY AND INSURANCE

A financial agreement will be made between the parties, institution, investigator and the sponsor, in accordance with each administrative procedure. All agreed costs will be described in the parties' signed contracts before the start of the study

Bioprojet will contract a specific insurance policy for the coverage of the patients in compliance with each National regulation. Liability and insurance provisions for this study are given in the investigator's contract.

18. FINANCIAL DISCLOSURE

Before the start of the study, the investigator will disclose to the sponsor any proprietary or financial interests he or she might hold in the investigational product or the sponsor company as outlined in the financial disclosure form provided by the sponsor. The investigator agrees to update this information in case of significant changes during the study or within one year of its completion. The investigator also agrees that, where required by law or regulation, the sponsor may submit this financial information to domestic or foreign regulatory authorities in applications for marketing authorizations.

Similar information will be provided by each sub-investigator to whom the investigator delegates significant study-related responsibilities.

19. CALENDAR FORECAST

First patient visit is scheduled in 1-2 Q 2016

Last patient visit is scheduled in 2 Q 2019

20. REFERENCES

1) Mc Nicolas W. T., Diagnosis of obstructive sleep apnea in adults. Proc Am Thorax Society 2008; 5: 154-160

2) Young T, Peppard P. E, Gottlieb D. J, Epidemiology of obstructive sleep apnea, A population health perspective. Am J Resp Crit Care Med 2002; 165: 1217-1239

3) Nieto F, Young T, Lind B, et al, Association of sleep-disordered breathing, sleep apnea, and hypertension in a large community-based study. JAMA 2000; 283:1829-1836

4) Peppard P, Young T, Palta M, et al, Prospective study of the association between sleep-disordered breathing and hypertension. N Engl J Med 2000; 342: 1378-1384

5) Bizieux-Thaminy A, Gagnadoux F, Binquet C, Meslier N, Person C, Racineux J.L, Long-term use of nCPAP therapy in sleep apnea patients. Rev Mal Respir 2005; 22:00-00

6) Felever-Grant J.C, Bruce A. S, Zimmerman M et al, Working memory in obstructive sleep apnea: construct validity and treatments effects. J Clin Sleep Med 2007; 3 (6): 589-594

7) Loube D, Gay P, Strohl K, et al, indications for positive airway pressure treatment of adult obstructive sleep apnea patients: a consensus statement. Chest 1999; 115: 863-866
8) Strollo P. J, Rogers R. Obstructive sleep apnea. N Engl J Med 1996; 334:99-104
9) Kushida C. A, Littner M. R, Hirshkowitz M et al, Practice parameters for the use of continuous and bilevel positive airway pressure devices to treat adult patients with sleep-related breathing disorders. Sleep 2006; 29 (3): 375-380
10) Kushida C. A, Chediak A, Berry R. B, Brown L. K, Grozal D, Iber C, Parthasarathy S, Quan S. F, Rowley J. A. Positive airway pressure titration task force of the American Academy of Sleep Medicine. Clinical guidelines for the manual titration of positive airway pressure in patients with obstructive sleep apnea. J Clin Sleep Med 2008; 4(2): 157-171
11) Schwartz J.R.L, Hirshkowitz M, Erman E. K, Schimdt-Nowara W, Modafinil as adjunct therapy for daytime sleepiness in obstructive sleep apnea. Chest 2003; 124: 2192-21999
12) Douglas N.J, Engleman H, CPAP therapy: Outcomes and patient use. Thorax 1998; 53 (Suppl 3): S47-48
13) Garcia-Diaz E, Quintana-Gallego E, Ruiz A, Carmona-Bernal C, Sanchez-Armengol A, Benhamou-Botebol G et al, Respiratory polygraphy with actigraphy in the diagnosis of sleep apnea-hypoapnea syndrome. Chest 2007; 131: 725-732
14) Woodford H.J and Georges J, Cognitive assessment in the elderly: a review of clinical methods; Q j Med 2007; 100: 469-484
15) Mazza S, Pepin J.L, Deschaux C, Naegele B, Levy P, Analysis of Error Profiles occurring during the OSLER test. Am J Respir Crit Care Med 2002; 166: 474-478
16) Hindmarch I, A 1, 4 benzodiazepine, temazepam (K3917): its effect on some psychological parameters of sleep and behaviour. Arzneimittel-Forschung (Drug research) 1975; 25 (11): 1836-1839
17) Schwartz A R, Patil S P, Laffan A M, Polotsky V, Schneider H, Smith P L, Obesity and obstructive sleep apnea. Pathogenic mechanisms and therapeutic approaches. Proc Am Thor Soc 2008; 5: 185-192
18) Shiroh Isono, Obstructive sleep apnea of obese adults. Pathophysiololgy and perioperative airway management. Anaesthesiology 2009; 110 (4): 908-921

21. APPENDIX SECTION

Appendix 1: Declaration of Helsinki

Appendix 2: Full-night polysomnography

Appendix 3: Epworth Sleepiness Scale (ESS)

Appendix 4: Sleep diary

Appendix 5: European Quality of Life questionnaire (EQ-5D)

Appendix 6: Leeds Sleep Evaluation Questionnaire (LSEQ)

Appendix 7: Pichot Fatigue Scale

Appendix 8: Trail Making Test (TMT) parts A & B

Appendix 9: Clinical Global Impression (CGI) rating scale

Appendix 10: Patient's global opinion on the effect of investigational drugs

Appendix 11: DSMB charter

Appendix 12: Instructions for filling in Bioprojet SAE Report Form

Appendix 13: Beck depression Inventory – 13 items (BDI-13)

Appendix 13: Patient's overall evaluation of the tolerance

Appendix 15: Amphetamine-like withdrawal symptoms questionnaire (DSM IV)

Appendix 16: Mini Mental State Examination questionnaire (MMSE)

Appendix 1: Declaration of Helsinki

Adopted by the 18th WMA General Assembly, Helsinki, Finland, June 1964
and amended by the:
29th WMA General Assembly, Tokyo, Japan, October 1975
35th WMA General Assembly, Venice, Italy, October 1983
41st WMA General Assembly, Hong Kong, September 1989
48th WMA General Assembly, Somerset West, Republic of South Africa, October 1996
52nd WMA General Assembly, Edinburgh, Scotland, October 2000
53rd WMA General Assembly, Washington DC, USA, October 2002 (Note of Clarification added)
55th WMA General Assembly, Tokyo, Japan, October 2004 (Note of Clarification added)
59th WMA General Assembly, Seoul, Republic of Korea, October 2008
64th WMA General Assembly, Fortaleza, Brazil, October 2013

Preamble

1. The World Medical Association (WMA) has developed the Declaration of Helsinki as a statement of ethical principles for medical research involving human subjects, including research on identifiable human material and data.

The Declaration is intended to be read as a whole and each of its constituent paragraphs should be applied with consideration of all other relevant paragraphs.

2. Consistent with the mandate of the WMA, the Declaration is addressed primarily to physicians. The WMA encourages others who are involved in medical research involving human subjects to adopt these principles.

General Principles

3. The Declaration of Geneva of the WMA binds the physician with the words, “The health of my patient will be my first consideration,” and the International Code of Medical Ethics declares that, “A physician shall act in the patient's best interest when providing medical care.”

4. It is the duty of the physician to promote and safeguard the health, well-being and rights of patients, including those who are involved in medical research. The physician's knowledge and conscience are dedicated to the fulfilment of this duty.

5. Medical progress is based on research that ultimately must include studies involving human subjects.

6. The primary purpose of medical research involving human subjects is to understand the causes, development and effects of diseases and improve preventive, diagnostic and therapeutic interventions (methods, procedures and treatments). Even the best proven interventions must be evaluated continually through research for their safety, effectiveness, efficiency, accessibility and quality.

7. Medical research is subject to ethical standards that promote and ensure respect for all human subjects and protect their health and rights.
8. While the primary purpose of medical research is to generate new knowledge, this goal can never take precedence over the rights and interests of individual research subjects.
9. It is the duty of physicians who are involved in medical research to protect the life, health, dignity, integrity, right to self-determination, privacy, and confidentiality of personal information of research subjects. The responsibility for the protection of research subjects must always rest with the physician or other health care professionals and never with the research subjects, even though they have given consent.
10. Physicians must consider the ethical, legal and regulatory norms and standards for research involving human subjects in their own countries as well as applicable international norms and standards. No national or international ethical, legal or regulatory requirement should reduce or eliminate any of the protections for research subjects set forth in this Declaration.
11. Medical research should be conducted in a manner that minimises possible harm to the environment.
12. Medical research involving human subjects must be conducted only by individuals with the appropriate ethics and scientific education, training and qualifications. Research on patients or healthy volunteers requires the supervision of a competent and appropriately qualified physician or other health care professional.
13. Groups that are underrepresented in medical research should be provided appropriate access to participation in research.
14. Physicians who combine medical research with medical care should involve their patients in research only to the extent that this is justified by its potential preventive, diagnostic or therapeutic value and if the physician has good reason to believe that participation in the research study will not adversely affect the health of the patients who serve as research subjects.
15. Appropriate compensation and treatment for subjects who are harmed as a result of participating in research must be ensured.

Risks, Burdens and Benefits

16. In medical practice and in medical research, most interventions involve risks and burdens. Medical research involving human subjects may only be conducted if the importance of the objective outweighs the risks and burdens to the research subjects.
17. All medical research involving human subjects must be preceded by careful assessment of predictable risks and burdens to the individuals and groups involved in the research in comparison with foreseeable benefits to them and to other individuals or groups affected by the condition under investigation.
Measures to minimise the risks must be implemented. The risks must be continuously monitored, assessed and documented by the researcher.

18. Physicians may not be involved in a research study involving human subjects unless they are confident that the risks have been adequately assessed and can be satisfactorily managed. When the risks are found to outweigh the potential benefits or when there is conclusive proof of definitive outcomes, physicians must assess whether to continue, modify or immediately stop the study.

Vulnerable Groups and Individuals

19. Some groups and individuals are particularly vulnerable and may have an increased likelihood of being wronged or of incurring additional harm.

All vulnerable groups and individuals should receive specifically considered protection.

20. Medical research with a vulnerable group is only justified if the research is responsive to the health needs or priorities of this group and the research cannot be carried out in a non-vulnerable group. In addition, this group should stand to benefit from the knowledge, practices or interventions that result from the research.

Scientific Requirements and Research Protocols

21. Medical research involving human subjects must conform to generally accepted scientific principles, be based on a thorough knowledge of the scientific literature, other relevant sources of information, and adequate laboratory and, as appropriate, animal experimentation. The welfare of animals used for research must be respected.

22. The design and performance of each research study involving human subjects must be clearly described and justified in a research protocol.

The protocol should contain a statement of the ethical considerations involved and should indicate how the principles in this Declaration have been addressed. The protocol should include information regarding funding, sponsors, institutional affiliations, potential conflicts of interest, incentives for subjects and information regarding provisions for treating and/or compensating subjects who are harmed as a consequence of participation in the research study. In clinical trials, the protocol must also describe appropriate arrangements for post-trial provisions.

Research Ethics Committees

23. The research protocol must be submitted for consideration, comment, guidance and approval to the concerned research ethics committee before the study begins. This committee must be transparent in its functioning, must be independent of the researcher, the sponsor and any other undue influence and must be duly qualified. It must take into consideration the laws and regulations of the country or countries in which the research is to be performed as well as applicable international norms and standards but these must not be allowed to reduce or eliminate any of the protections for research subjects set forth in this Declaration.

The committee must have the right to monitor ongoing studies. The researcher must provide

monitoring information to the committee, especially information about any serious adverse events. No amendment to the protocol may be made without consideration and approval by the committee. After the end of the study, the researchers must submit a final report to the committee containing a summary of the study's findings and conclusions.

Privacy and Confidentiality

24. Every precaution must be taken to protect the privacy of research subjects and the confidentiality of their personal information.

Informed Consent

25. Participation by individuals capable of giving informed consent as subjects in medical research must be voluntary. Although it may be appropriate to consult family members or community leaders, no individual capable of giving informed consent may be enrolled in a research study unless he or she freely agrees.

26. In medical research involving human subjects capable of giving informed consent, each potential subject must be adequately informed of the aims, methods, sources of funding, any possible conflicts of interest, institutional affiliations of the researcher, the anticipated benefits and potential risks of the study and the discomfort it may entail, post-study provisions and any other relevant aspects of the study. The potential subject must be informed of the right to refuse to participate in the study or to withdraw consent to participate at any time without reprisal. Special attention should be given to the specific information needs of individual potential subjects as well as to the methods used to deliver the information.

After ensuring that the potential subject has understood the information, the physician or another appropriately qualified individual must then seek the potential subject's freely-given informed consent, preferably in writing. If the consent cannot be expressed in writing, the non-written consent must be formally documented and witnessed.

All medical research subjects should be given the option of being informed about the general outcome and results of the study.

27. When seeking informed consent for participation in a research study the physician must be particularly cautious if the potential subject is in a dependent relationship with the physician or may consent under duress. In such situations the informed consent must be sought by an appropriately qualified individual who is completely independent of this relationship.

28. For a potential research subject who is incapable of giving informed consent, the physician must seek informed consent from the legally authorised representative. These individuals must not be included in a research study that has no likelihood of benefit for them unless it is intended to promote the health of the group represented by the potential subject, the research cannot instead be performed with persons capable of providing informed consent, and the research entails only minimal risk and minimal burden.

29. When a potential research subject who is deemed incapable of giving informed consent is

able to give assent to decisions about participation in research, the physician must seek that assent in addition to the consent of the legally authorised representative. The potential subject's dissent should be respected.

30. Research involving subjects who are physically or mentally incapable of giving consent, for example, unconscious patients, may be done only if the physical or mental condition that prevents giving informed consent is a necessary characteristic of the research group. In such circumstances the physician must seek informed consent from the legally authorised representative. If no such representative is available and if the research cannot be delayed, the study may proceed without informed consent provided that the specific reasons for involving subjects with a condition that renders them unable to give informed consent have been stated in the research protocol and the study has been approved by a research ethics committee. Consent to remain in the research must be obtained as soon as possible from the subject or a legally authorised representative.

31. The physician must fully inform the patient which aspects of their care are related to the research. The refusal of a patient to participate in a study or the patient's decision to withdraw from the study must never adversely affect the patient-physician relationship.

32. For medical research using identifiable human material or data, such as research on material or data contained in biobanks or similar repositories, physicians must seek informed consent for its collection, storage and/or reuse. There may be exceptional situations where consent would be impossible or impracticable to obtain for such research. In such situations the research may be done only after consideration and approval of a research ethics committee.

Use of Placebo

33. The benefits, risks, burdens and effectiveness of a new intervention must be tested against those of the best proven intervention(s), except in the following circumstances:

Where no proven intervention exists, the use of placebo, or no intervention, is acceptable; or

Where for compelling and scientifically sound methodological reasons the use of any intervention less effective than the best proven one, the use of placebo, or no intervention is necessary to determine the efficacy or safety of an intervention and the patients who receive any intervention less effective than the best proven one, placebo, or no intervention will not be subject to additional risks of serious or irreversible harm as a result of not receiving the best proven intervention.

Extreme care must be taken to avoid abuse of this option.

Post-Trial Provisions

34. In advance of a clinical trial, sponsors, researchers and host country governments should make provisions for post-trial access for all participants who still need an intervention identified as beneficial in the trial. This information must also be disclosed to participants during the

informed consent process.

Research Registration and Publication and Dissemination of Results

35. Every research study involving human subjects must be registered in a publicly accessible database before recruitment of the first subject.

36. Researchers, authors, sponsors, editors and publishers all have ethical obligations with regard to the publication and dissemination of the results of research. Researchers have a duty to make publicly available the results of their research on human subjects and are accountable for the completeness and accuracy of their reports. All parties should adhere to accepted guidelines for ethical reporting. Negative and inconclusive as well as positive results must be published or otherwise made publicly available. Sources of funding, institutional affiliations and conflicts of interest must be declared in the publication. Reports of research not in accordance with the principles of this Declaration should not be accepted for publication.

Unproven Interventions in Clinical Practice

37. In the treatment of an individual patient, where proven interventions do not exist or other known interventions have been ineffective, the physician, after seeking expert advice, with informed consent from the patient or a legally authorised representative, may use an unproven intervention if in the physician's judgement it offers hope of saving life, re-establishing health or alleviating suffering. This intervention should subsequently be made the object of research, designed to evaluate its safety and efficacy. In all cases, new information must be recorded and, where appropriate, made publicly available.

Informed Consent Process

- Information provided to the patient by the site
- Review and signature of Informed Consent
- One copy is kept at the site
- One copy is given to the patient

Appendix 2: Full-Night Polysomnography

Polysomnography is a standard full-night diagnostic study. Continuous recordings of the electroencephalogram (C3/A2-C4/A1-CZ/O1 from the International 10-20 Electrode Placement System), eye movement measurements, chin electromyogram (EMG) and electrocardiogram (ECG). Respiratory effort is monitored with uncalibrated inductance respiratory plethysmography. Airflow is measured with nasal pressure, as well as with the sum of buccal and nasal thermistor signals. An additional signal of respiratory effort (i.e., pulse transit time or oesophageal pressure) is recorded concurrently. Oxygen saturation is measured using a pulse oximeter (Biox- Ohmeda 3700; Ohmeda; Liberty Corner, NJ).

The polysomnogram is scored manually according to standard criteria (*).

Episodes of apnea are defined as complete cessations of airflow for more than 10 s; episodes of hypopnoea as decreases in oronasal airflow of more than 50% and lasting at least 10 s, a decrease of more than 30% associated with a decrease in oxygen saturation of more than 3%, or a microarousal. Apnea/hypopnoea events are classified as central, obstructive, or mixed depending on the absence or presence of breathing efforts.

**Rechtshaffen, A., and A. Kales. 1968. A manual of standardized terminology, technique and scoring system for sleep stages of human sleep. Brain Information Service, Brain Information Institute, University of California, Los Angeles.*

The Polysomnography will be performed between V1 and the inclusion visit V2 (if no polysomnography was performed during the previous 12 months).

Appendix 3: Epworth Sleepiness Scale (ESS)

The Epworth Sleepiness Scale is used to determine the level of daytime sleepiness. A score of 10 or more is considered sleepy. A score of 18 or more is very sleepy. If you score 10 or more on this test, you should consider whether you are obtaining adequate sleep, need to improve your sleep hygiene and/or need to see a sleep specialist. These issues should be discussed with your personal physician.

Use the following scale to choose the most appropriate number for each situation:

***0** = Would never doze or sleep*

***1** = Slight chance of dozing or sleeping*

***2** = Moderate chance of dozing or sleeping*

***3** = High chance of dozing or sleeping*

Print out this test, fill in your answers and see where you stand.

SITUATION	CHANCE OF DOZING OR FALLING ASLEEP
Sitting and reading	____
Watching TV	____
Sitting inactive in a public place	____
Being a passenger in a motor vehicle for an hour or more	____
Lying down in the afternoon	____
Sitting and talking to someone	____
Sitting quietly after lunch (no alcohol)	____
Stopped for a few minutes in traffic while driving	____
**Total score (add the scores up)**<br>(This is your Epworth score)	____

Appendix 4: Sleep Diary

BIOPROJET	Protocol P 09-09 / BF 2.649	Patient Number: ⊔⊔	Centre Number: ⊔⊔	SLEEP DIARY N°: ⊔⊔ *First day of filling*

1. Complete the below grid along the day according to the following codes :

- ↓ Go to bed ↑ Get up (to begin the day)
- Hatch the sleep periods (diurnal and nocturnal) ////////////// and keep in blank the waking periods
- Indicate the sleepiness episode by a ★
- Indicate your sudden on sleep episode by a ●

Example

Date (DD/MM/YY)	Study treatment intake
Example: 31/10/06	10h00
To be completed by the patient before going to sleep	

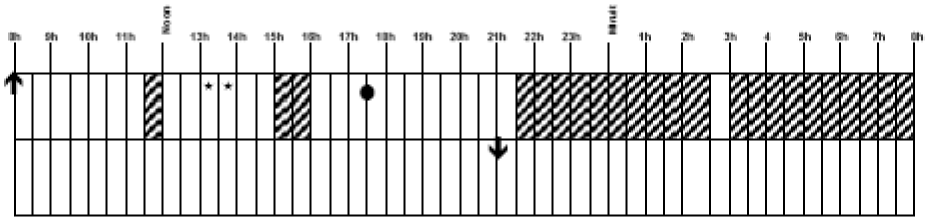

2. Please complete the following information according to the above grid:

- **What time did you wake up this morning? :** ⊔⊔h⊔⊔ min
- **What time did you get up this morning? :** ⊔⊔h⊔⊔ min
- **Number of episodes of sleep and sleepiness during the day** (naps and ★) : ⊔⊔
- **Total duration of sleep and sleepiness during the day**(naps and ★) : ⊔⊔h⊔⊔ min
- **What time did you go to sleep last night? :** ⊔⊔h⊔⊔ min
- **What is the estimated time of your falling asleep last night? :** ⊔⊔h⊔⊔ min
- **Number of awakening episodes during last night (●):** ⊔⊔
- **Total duration of these awakening episodes during last night.:** ⊔⊔h⊔⊔ min

Appendix 5: European Quality of Life Questionnaire (EQ-5D)

Date: __ __ / __ __ __/ __ __ __ __

Visit: ☐ **Baseline**

EQ - 5D **Health Questionnaire**
(English version for the UK) *- (validated for use in Eire)*

By placing a tick in one box in each group below, please indicate which statements best describe your own health state today.

Mobility

I have no problems in walking about ☐

I have some problems in walking about ☐

I am confined to bed ☐

Self-Care

I have no problems with self-care ☐

I have some problems washing or dressing myself ☐

I am unable to wash or dress myself ☐

Usual Activities *(e.g. work, study, housework, family or leisure activities)*

I have no problems with performing my usual activities ☐

I have some problems with performing my usual activities ☐

I am unable to perform my usual activities ☐

Pain/Discomfort

I have no pain or discomfort ☐

I have moderate pain or discomfort ☐

I have extreme pain or discomfort ☐

Anxiety/Depression

I am not anxious or depressed ☐

I am moderately anxious or depressed ☐

I am extremely anxious or depressed ☐

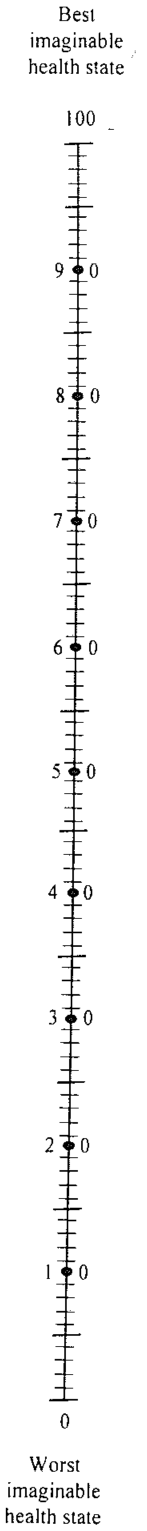

To help people say how good or bad a health state is, we have drawn a scale (rather like a thermometer) on which the best state you can imagine is marked 100 and the worst state you can imagine is marked 0.

We would like you to indicate on this scale how good or bad your own health is today, in your opinion. Please do this by drawing a line from the box below to whichever point on the scale indicates how good or bad your health state is today.

Your own health state today

Appendix 6: Leeds Sleep Evaluation Questionnaire (LSEQ)

Leeds Sleep Evaluation Questionnaire

How would you describe the way you currently fall asleep in comparison to usual?

GTS - getting to sleep

No.				
1.	More difficult than usual	______	Easier than usual	
2.	Slower than usual	______	More quickly than usual	
3.	I feel less sleepy than usual	______	More sleepy than usual	

How would you describe the quality of your sleep compared to normal sleep?

QOS - quality of sleep

No.				
4.	More restless than usual	______	Calmer than usual	
5.	With more wakeful periods than usual	______	With less wakeful periods than usual	

How would you describe your awakening in comparison to usual?

AFS – Awake following sleep

No.				
6.	More difficult than usual	______	Easier than usual	
7.	Requires a period of time longer than usual	______	Shorter than usual	

BFW – behaviour following wakening

How do you feel when you wake up?

No.				
8.	Tired	______	Alert	

How do you feel now?

No.				
9.	Tired	______	Alert	

How would you describe your balance and co-ordination upon awakening?

No.				
10.	More disrupted than usual	______	Less disrupted than usual	

Appendix 7: Pichot Fatigue Scale

(Ref. "Scales and tools of evaluation in general medicine" J. Gardenas and Coll. - The General practitioner Supplement of N°2187; March 2002).

Fatigue is a feeling of physical or psychic decline which normally arises after a sustained effort, and which makes it necessary to take a rest.
We speak about pathological fatigue whenever the person feels at a disadvantage with regard to his/her usual physical condition to carry out his/her daily activities.

Pichot subjective scale was proposed to measure the importance of this impairment.

First name: .... Name: .... Date of birth: ....
Date of test: ....Current Treatment ……..................................... ....

Among the following eight suggestions, determine those who best correspond to your state by scoring each item between 0 and 4:

(0 = not at all; 1= a little, 2 = moderately, 3= much, 4 = extremely)

- I feel short of stamina... 0 1 2 3 4
- Everything I do requires a huge effort.......................................0 1 2 3 4
- I have a feeling of weakness in certain parts of my body….......0 1 2 3 4
- My arms or legs are heavy……………………… 0 1 2 3 4
- I feel tired for no reason…………………….............................0 1 2 3 4
- I feel like lying down or resting ……………………….............0 1 2 3 4
- I have difficulty to concentrate 0 1 2 3 4
- I feel discouraged, my arms and legs are sore and heavy 0 1 2 3 4

Total (out of 32):.........................

A score <u>higher than 22</u> is in favour of an excessive fatigue; you are perhaps suffering from an inefficient sleep.

NB. This questionnaire is used to measure your general level of Fatigue and cannot be considered as a medical diagnosis. Bring it to your doctor to discuss the causes and consequences of this fatigue in your life.

Appendix 8: Trail Making Test (TMT) Parts A & B

Trail Making Test (TMT) Parts A & B

Instructions:

Both parts of the Trail Making Test consist of 25 circles distributed over a sheet of paper. In Part A, the circles are numbered 1 – 25, and the patient should draw lines to connect the numbers in ascending order. In Part B, the circles include both numbers (1 – 13) and letters (A – L); as in Part A, the patient draws lines to connect the circles in an ascending pattern, but with the added task of alternating between the numbers and letters (i.e., 1-A-2-B-3-C, etc.). The patient should be instructed to connect the circles as quickly as possible, without lifting the pen or pencil from the paper. Time the patient as he or she connects the "trail." If the patient makes an error, point it out immediately and allow the patient to correct it. Errors affect the patient's score only in that the correction of errors is included in the completion time for the task. It is unnecessary to continue the test if the patient has not completed both parts after five minutes have elapsed.

Step 1: Give the patient a copy of the Trail Making Test Part A worksheet and a pen or pencil.

Step 2: Demonstrate the test to the patient using the sample sheet (Trail Making Part A – *SAMPLE*).

Step 3: Time the patient as he or she follows the "trail" made by the numbers on the test.

Step 4: Record the time.

Step 5: Repeat the procedure for Trail Making Test Part B.

Scoring:

Results for both TMT A and B are reported as the number of seconds required to complete the task; therefore, higher scores reveal greater impairment.

	Average	Deficient	Rule of Thumb
Trail A	29 seconds	> 78 seconds	Most in 90 seconds
Trail B	75 seconds	> 273 seconds	Most in 3 minutes

Sources:

- Corrigan JD, Hinkeldey MS. Relationships between parts A and B of the Trail Making Test. *J Clin Psychol.* 1987;43(4):402–409.
- Gaudino EA, Geisler MW, Squires NK. Construct validity in the Trail Making Test: what makes Part B harder? *J Clin Exp Neuropsychol.* 1995;17(4):529-535.
- Lezak MD, Howieson DB, Loring DW. *Neuropsychological Assessment.* 4th ed. New York: Oxford University Press; 2004.
- Reitan RM. Validity of the Trail Making test as an indicator of organic brain damage. *Percept Mot Skills.* 1958;8:271-276.

Trail Making Test Part A

Patient's Name: ______________________ Date: ______________

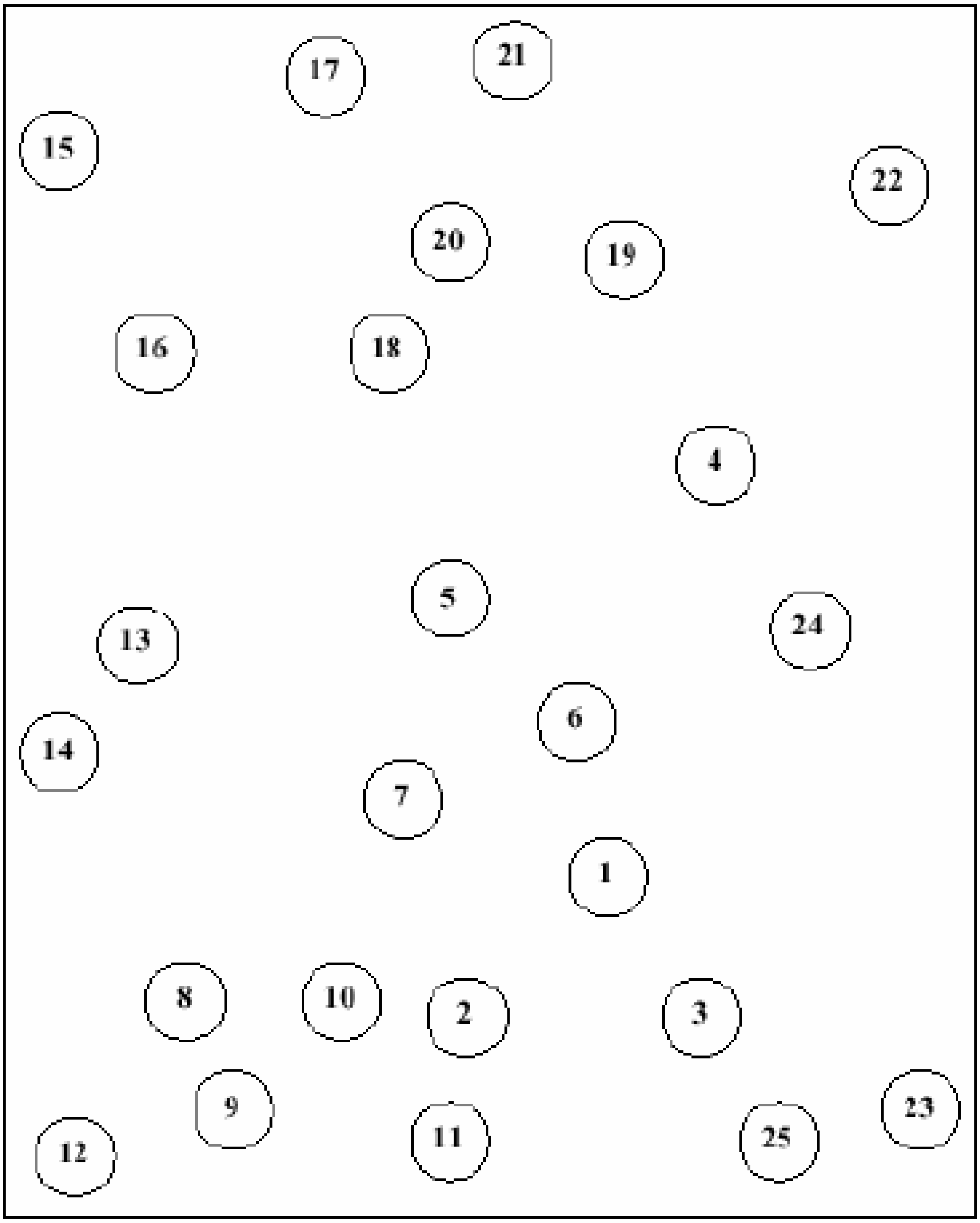

Trail Making Test Part A – *SAMPLE*

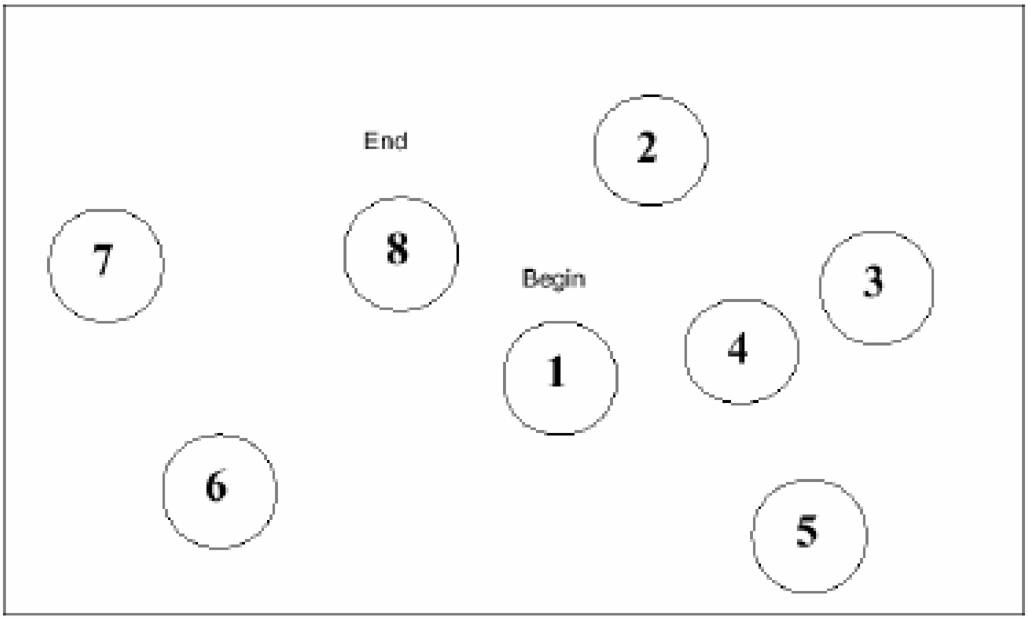

Trail Making Test Part B

Patient's Name: ______________________________ Date: ____________________

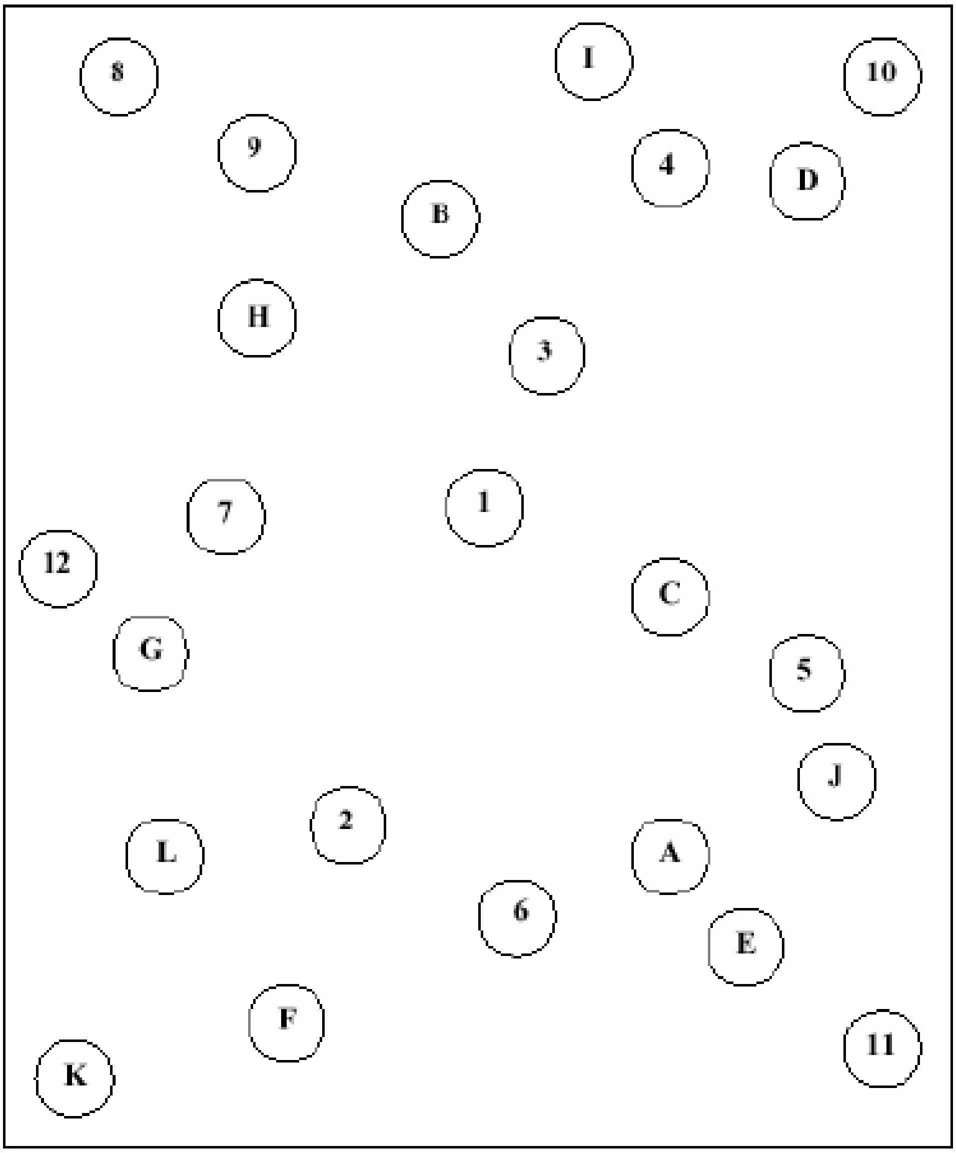

Trail Making Test Part B – *SAMPLE*

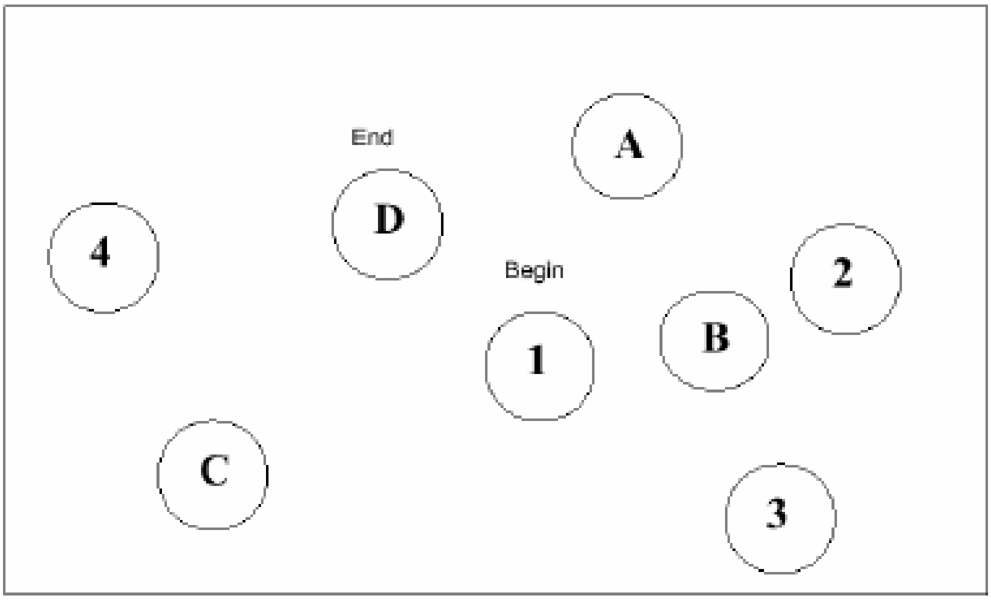

Appendix 9: Clinical Global Impression (CGI) Rating Scale

Clinical Global Impression (CGI)

***Reference:* Guy W, editor. ECDEU Assessment Manual for Psychopharmacology. 1976. Rockville, MD, U.S. Department of Health, Education, and Welfare**

Rating Clinician-rated

Administration time Varies with familiarity with patient

Main purpose To provide a global rating of illness severity, improvement and response to treatment

Population Adults

Commentary

Amongst the most widely used of extant brief assessment tools in psychiatry, the CGI is a 3-item observer-rated scale that measures illness severity (CGIS), global improvement or change (CGIC) and therapeutic response. The illness severity and improvement sections of the instrument are used more frequently than the therapeutic response section in both clinical and research settings. The Early Clinical Drug Evaluation Program (ECDEU) version of the CGI (reproduced here) is the most widely used format, and asks that the clinician rate the patient relative to their past experience with other patients with the same diagnosis, with or without collateral information. Several alternative versions of the CGI have been developed, however, such as the FDA Clinicians' Interview-Based Impression of Change (CIBIC), which uses only information collected during the interview, not collateral. The CGI has proved to be a robust measure of efficacy in many clinical drug trials, and is easy and quick to administer, provided that the clinician knows the patient well.

Scoring

The CGI is rated on a 7-point scale, with the severity of illness scale using a range of responses from 1 (normal) through to 7 (amongst the most severely ill patients). CGI-C scores range from 1 (very much improved) through to 7 (very much worse). Treatment response ratings should take account of both therapeutic efficacy and treatment-related adverse events and range from 0 (marked improvement and no side-effects) and 4 (unchanged or worse and side-effects outweigh the therapeutic effects). Each component of the CGI is rated separately; the instrument does not yield a global score.

Versions

CGI for bipolar disorder (CGI-BD), FDA Clinicians' Interview-Based Impression of Change (CIBIC), Clinicians' Interview-Based Impression of Change-Plus (CIBIC+), NYU CIBIC+, Parke-Davis Pharmaceuticals Clinical Interview-Based Impression (CIBI); the CGI has been translated into most languages.

Additional references

Leon AC, Shear MK, Klerman GL, Portera L, Rosenbaum JF, Goldenberg I. A comparison of symptom determinants of patient and clinician global ratings in patients with panic disorder and depression. J Clin Psychopharmacol 1993; 13(5):327–31.

Spearing MK, Post RM, Leverich GS, Brandt D, Nolen W. Modification of the Clinical Global Impressions (CGI) Scale for use in bipolar illness (BP): the CGI-BP. Psychiatry Res 1997; 73(3):159–71.

Zaider TI, Heimberg RG, Fresco DM, Schneier FR, Liebowitz MR. Evaluation of the clinical global impression scale among individuals with social anxiety disorder. Psychol Med 2003; 33(4):611–22.

Address for correspondence

Not applicable – the CGI is in the public domain.

Clinical Global Impression (CGI)

1. **Severity of illness**
   Considering your total clinical experience with this particular population, how mentally ill is the patient at this time?

0 = Not assessed	4 = Moderately ill
1 = Normal, not at all ill	5 = Markedly ill
2 = Borderline mentally ill	6 = Severely ill
3 = Mildly ill	7 = Among the most extremely ill patients

2. **Global improvement:** Rate total improvement whether or not, in your judgement, it is due entirely to drug treatment.
   Compared to his condition at admission to the project, how much has he changed?

0 = Not assessed	4 = No change
1 = Very much improved	5 = Minimally worse
2 = Much improved	6 = Much worse
3 = Minimally improved	7 = Very much worse

3. **Efficacy index:** Rate this item on the basis of **drug effect only.**
   Select the terms which best describe the degrees of therapeutic effect and side effects and record the number in the box where the two items intersect.
   EXAMPLE: Therapeutic effect is rated as 'Moderate' and side effects are judged 'Do not significantly interfere with patient's functioning'.

**Therapeutic effect**		**Side effects**			
		None	*Do not significantly interfere with patient's functioning*	*Significantly interferes with patient's functioning*	*Outweighs therapeutic effect*
**Marked**	Vast improvement. Complete or nearly complete remission of all symptoms	01	02	03	04
**Moderate**	Decided improvement. Partial remission of symptoms	05	06	07	08
**Minimal**	Slight improvement which doesn't alter status of care of patient	09	10	11	12
**Unchanged or worse**		13	14	15	16

Not assessed = 00

Reproduced from Guy W, editor. ECDEU Assessment Manual for Psychopharmacology. 1976. Rockville, MD, U.S. Department of Health, Education, and Welfare

Appendix 10: Patient's global opinion on the effect of investigational drugs

**PATIENT'S GLOBAL OPINION on the effect of the investigational drugs**	
Ask the patient to complete the questionnaire by comparing the treatment period referenced to the prestudy conditions.<br>Report hereunder the date and time of performance and the patient's answer.	
Date: |__|__|/|__|__|/|__|__| (DD/MM/YY) Time: |__|__|h|__|__|min	
❒ Marked effect<br>(complete or nearly complete remission of EDS)	❒ Moderate effect<br>(partial remission of EDS)
❒ Minimal effect<br>(slight decrease in EDS that does not substantially alter the status of the patient)	❒ No change
❒ Minimally worse<br>(slight increase in EDS)	❒ Much worse<br>(substantial increase in EDS)

Appendix 11: DSMB charter

DATA SAFETY MONITORING BOARD (DSMB)

CHARTER

Protocol P1513

Efficacy and Safety of Pitolisant (BF2.649) in the Treatment of Excessive Daytime Sleepiness in Patients with Obstructive Sleep Apnoea Syndrome, Treated or Not by Nasal Continuous Positive Airway Pressure, but Still Complaining of Excessive Daytime Sleepiness – Phase III.

According to ICH E9 an independent DSMB may be established by the sponsor to assess at intervals the progress of a clinical trial, safety data, and critical efficacy variables and recommend to the sponsor whether to continue, modify or terminate a trial. The DSMB should have written operating procedures and maintain records of all its meetings, including interim results; these should be available for review when the trial is complete. The independence of the DSMB is intended to control the sharing of important comparative information and to protect the integrity of the clinical trial from adverse impact resulting from access to trial information. The DSMB is a separate entity from an Institutional review Board (IRB) or an Independent Ethics Committee (IEC), and its composition should include clinical trial scientists knowledgeable in the appropriate disciplines including statistics.
When there are sponsor representatives on the DSMB, their role should be clearly defined in the operating procedures of the committee (for example, covering whether or not they can vote on key issues). Since these sponsor staff would have access to unblinded information, the procedures should also address the control of dissemination of interim trial results within the sponsor organization.

I. Roles and Responsibilities

The Data and Safety Monitoring Board (DSMB) is an independent group of experts that advises the study investigators. The members of the DSMB serve in an individual capacity and provide their expertise and recommendations.
The primary responsibilities of the DSMB are to :

1) Periodically review and evaluate the accumulated study data for participant safety, study conduct and progress, and, when appropriate, efficacy, and
2) Make recommendations concerning the continuation, modification, or termination of the trial. The DSMB considers study-specific data as well as relevant background knowledge about the disease, test agent, or patient population under study.
The DSMB is also responsible for maintaining the confidentiality of its internal discussions and activities as well as the contents of reports provided to it.
The DSMB should review each protocol for any major concern prior to implementation. During the trial, the DSMB should review cumulative study data to evaluate safety, study conduct, and scientific validity and integrity of the trial. As part of this responsibility, DSMB members must be satisfied that the timeliness, completeness, and accuracy of the data submitted to them for review are sufficient for evaluation of the safety and welfare of study participants. The DSMB should also assess the performance of overall study operations and any other relevant issues, as necessary.
Items reviewed by the DSMB include:

- Interim/cumulative data for evidence of study-related adverse events;
- Interim/cumulative data for evidence of efficacy according to pre-established statistical guidelines, if appropriate;
- Data quality, completeness, and timeliness;
- Performance of individual centers;
- Adequacy of compliance with goals for recruitment and retention, including those related to the participation of women and minorities;
- Adherence to the protocol;
- Factors that might affect the study outcome or compromise the confidentiality of the trial data (such as protocol violations, unmasking, etc.); and,
- Factors external to the study such as scientific or therapeutic developments that may impact participant safety or the ethics of the study.

The DSMB should conclude each review with their recommendations as to whether the study should continue without change, be modified, or terminated. Recommendations regarding modification of the design and conduct of the study could include:

- Modifications of the study protocol based upon the review of the safety data;
- Suspension or early termination of the study or of one or more study arms because of serious concerns about subjects' safety, inadequate performance or rate of enrollment;
- Suspension or early termination of the study or of one or more study arms because study objectives have been obtained according to pre-established statistical guidelines;
- Optional approaches for investigators to consider when the DSMB determines that the incidence of primary study outcomes is substantially less than expected such as

recommendations to increase the number of trial centers or extend the recruitment period; and,

- Corrective actions regarding a study center whose performance appears unsatisfactory or suspicious.

Confidentiality must always be maintained during all phases of DSMB review and deliberations. Usually, only voting members of the DSMB should have access to interim analyses of outcome data by treatment group. Exceptions may be made when the DSMB deems it appropriate. The reason and to whom the exceptions for access to interim analyses is granted will be documented in the Closed Session Report. DSMB members must maintain strict confidentiality concerning all privileged trial results ever provided to them. The DSMB should review data only by masked study group (such as X vs. Y rather than experimental vs. control) unless or until the DSMB determines that the identities of the groups are necessary for their decision-making. Whenever masked data are presented to the DSMB, the key to the group coding must be available for immediate unmasking.

II. Membership

The membership of the DSMB should reflect the disciplines and medical specialties necessary to interpret the data from the clinical trial and to fully evaluate participant safety. The number of DSMB members depends on the phase of the trial, range of medical issues, complexity in design and analysis, and potential level of risk but generally consists of three to seven members including, at a minimum:

- Expert(s) in the clinical aspects of the disease/patient population being studied;
- One or more biostatisticians; and,
- Investigators with expertise in current clinical trials conduct and methodology.

Ad hoc specialists may be invited to participate as non-voting members at any time if additional expertise is desired. Some trials, depending on the population and nature of the intervention, may well be served by inclusion of a bioethicist on the DSMB, Steering Committee, or Advisory Panel.

Conflict of Interest

No member of the DSMB should have direct involvement in the conduct of the study. Furthermore, no member should have financial, proprietary, professional, or other interests that may affect impartial, independent decision-making by the DSMB. Interests that may create a potential conflict of interest should be disclosed to the DSMB prior to any discussion. The DSMB will determine how to handle such potential conflict. The DSMB can require that a member with a potential conflict not vote or take other means deemed appropriate.

Selection and Invitation to Participate

Participation is generally for the duration of the study. Participation for standing DSMBs convened to monitor multiple protocols or lengthy studies may be for fixed terms.

III. Meetings

The frequency of DSMB meetings depends on several factors including the rate of enrollment, safety issues or unanticipated adverse events, availability of data, and, where relevant, scheduled interim analyses. The representative's Sponsor is responsible for convening meetings, selecting a venue when the meeting is not convened by teleconference, and coordinating the distribution of meeting materials to DSMB members and other meeting participants. The agenda for each meeting is generally developed jointly by the representative's Sponsor, the Principal Investigator (regardless of whether a contract, cooperative agreement, or grant), the study statistician, and DSMB Chair.

The initial DSMB meeting should occur preferably before the start of the trial or as soon thereafter as possible. At this meeting the DSMB should discuss the protocol and the DSMB charter which includes triggers set for data review or analyses, definition of a quorum, and guidelines for monitoring the study. Guidelines should also address stopping the study for safety concerns and, where relevant, for efficacy based on plans specified in the protocol. At this meeting, the DSMB should also develop procedures for conducting business (e.g., voting rules, attendance, etc.).

Once a study is implemented, the DSMB should convene as often as necessary, but at least once annually, to examine the accumulated safety and enrolment data, review study progress, and discuss other factors (internal or external to the study) that might impact continuation of the study as designed. A DSMB meeting may be requested by DSMB members, industrial collaborator, IRB, or study Principal Investigator at any time to discuss safety concerns. In the event a DSMB member cannot attend a meeting, he/she may receive a copy of the closed session DSMB report (see below) and either participate by conference call or provide written comments to the DSMB Chair for consideration at the meeting.

DSMB Meeting Format

The recommended meeting format consists of Open Sessions, Closed Session, and Closed Executive Session.

Open Session: Open session reports generally include administrative reports by site that describe participants screened, enrolled, completed, and discontinued, as well as baseline characteristics of the study population. Other general information on study status may also be presented. Listings of adverse events and serious adverse events, but none of the data should be presented in an unblinded manner.

Issues relating to the general conduct and progress of the study are discussed including adverse events and toxicity issues, accrual, demographic characteristics of enrollees, disease status of

enrollees (if relevant), comparability of groups with respect to baseline factors, protocol compliance, site performance, quality control, and timeliness and completeness of follow-up. Outcome results must not be discussed during this session. DSMB members, voting and invited *ex officio* members, ad hoc experts attend this session. The lead investigator and the study biostatistician should be in attendance in order to present results and respond to questions. This session is open to representatives for industrial collaborators, study investigators.

Closed Session: Grouped safety data_and, if appropriate, efficacy data are presented by the study statistician(s) at this session. Grouped data should be presented by coded treatment arm. This session is normally attended only by voting members, and invited *ex officio* members. The DSMB may invite the participation of other individuals for all or part of the session.

Closed Executive Session: This final session involves only DSMB voting members to ensure complete objectivity as they discuss outcome results, make decisions, and formulate recommendations regarding the study. If treatment codes have been made accessible to the DSMB, then the DSMB may unmask the data based on procedures identified in advance.

Voting

A quorum, as defined by the DSMB in the initial meeting, must be present either in person or by conference call. After a thorough discussion of DSMB members' opinions and rationale and an attempt to reach clarity regarding individual recommendations, the final recommendations of each DSMB member should be solicited in Closed Executive Session (*ex officio* members shall not vote and shall not be present at this voting session).). A consensus opinion or recommendation among members is not required; each member may have individual opinions. The final recommendations are recorded and either identified as majority or minority positions or are accompanied by actual vote tallies for each divergent recommendation, i.e., as number of votes for or against a particular action, such as continuing or terminating a study, etc.

IV. Study Reports for DSMB Meetings

Summary safety and enrollment data should be forwarded periodically to the DSMB. The DSMB should receive all protocol revisions and may receive other documents relating to the study including Clinical Investigator's Brochure (CIB) and all CIB revision.

Reports are prepared by the study statistician(s). The study statistician should provide suggested formats or templates for data presentation for the initial meeting of the DSMB. At subsequent meetings, additions or modifications to these reports may be directed by the DSMB on a one-time or continuing basis. Written reports should be sent to DSMB members prior to the meeting and should allow sufficient time for review.

Reports for meetings of the DSMB consist in the data presented in the reports reflecting both the need for the fullest possible information on trial results and the need to assure reliability and accuracy of the information included.

This report may contain data on study outcomes, including safety data and, depending on the study, efficacy data coded by group. Interim analyses of efficacy data are presented only when

planned in advance and appropriate statistical criteria for assessing evidence of efficacy have been clearly addressed. Supplemental information may need to be furnished immediately after the meeting if the DSMB decides that such follow-up is needed in order to conclude their deliberations.

The Closed Session Report is confidential and marked accordingly. Copies of reports distributed prior to and during a meeting are collected by the study statistician(s) at the end of the Closed Session. Procedures for securing closed reports distributed to telephone and videoconference participants should be specified in advance of the meeting.

V. Other Reports of Study Progress

Masked safety and enrollment data may be forwarded periodically to all DSMB members or to the member who serves as the Independent Safety Monitor. The DSMB receives all protocol revisions and may receive other documents relating to the study, such as annual reports, manuscripts, and newsletters.

VI. Reports from the DSMB

Summary Report: The DSMB will issue a written summary report that identifies topics discussed by the DSMB and describes their individual findings, overall safety assessment and recommendations. The rationale for recommendations will be included when appropriate. This report will generally not include confidential information. The DSMB Chair or designee is responsible for drafting, circulating and obtaining approval from other DSMB members within two (2) weeks of the meeting. The final summary report will be forwarded through a designated study team representative (usually the Principal Investigator) and to other appropriate staff. The study team representative is responsible for disseminating the DSMB summary report to site investigators who must, in turn, submit the report to their local IRBs.

Closed Session Report: (optional): The DSMB may also prepare confidential minutes that include details of closed session discussions. Meeting minutes are to be held in strict confidence, accessible only to voting members of the DSMB until such time when the study is closed or the DSMB recommends early termination or in the event the minutes are requested by the Competent Authorities for participant safety reasons or for regulatory purposes.

Immediate Action Report: The DSMB Chair will notify the representative's Sponsor of any findings of a serious and immediate nature or recommendations to discontinue all or part of the trial. Recommendations to discontinue or substantially modify the design or conduct of a study must be conveyed in writing by e-mail, fax, or courier on the day of the DSMB meeting. This written, confidential report may include the DSMB member's rationale for their recommendations.

VII. Relationship between DSMBs and IRBs

The DSMB should provide feedback at regular and defined intervals to the IRBs. A brief summary report should be sent to each investigator after each meeting. The report should document that a review of data and outcomes across all centers took place on a given date. It should summarize the DSMB members' review of the cumulative toxicities reported from all participating sites without specific disclosure by treatment arm. It should also inform study investigators of the DSMB members' conclusions with respect to progress or need for modification of the protocol. The investigator is required to transmit the report to his/her local IRB.

VIII. Trial Description and Study Design

- Trial name: P1513
- Trial sponsor: bioprojet
- Study drug tested: pitolisant (BF2.649)
- Trial design: Double blind placebo controlled parallel groups (12 weeks study treatment) with 39 weeks Open Label Extension period
- Phase: phase III
- Number of expected patients: 180
- Study sites: several sites in Bulgaria

IX. DSMB Description

- This DSMB will be coordinated by bioprojet 's representative
- This DSMB will be independent of bioprojet, regulatory agencies, IRB/EC, and investigators.
- This charter will be approved by its DSMB members as attested to by signature of the chairperson.

DSMB members

The DSMB members have been selected by the sponsor.

Appendix 12: Instructions for Filling In Bioprojet SAE Report Form

General instructions

The form must be completed in English. Please use capital letters and black pen to increase legibility.

Avoid using abbreviations as these may differ across centres and countries. If abbreviations are used in the narrative section, they should be explained in the text. Use medical terminology and be clear, concise and unambiguous.

Patient details and history

Enter patient number (patient number, screening number and/or randomisation number).

If the event occurs prior to patient number allocation, please enter inevitably the screening number.

Resume the relevant patient medical history.

Seriousness criterion

Please indicate the reason why the adverse event is serious (tick all that apply) in accordance with the following criteria:

- results in death,
- is immediately life threatening
- requires in-patient hospitalisation or prolongation of existing hospitalisation
- results in persistent or significant disability or incapacity
- is a congenital abnormality/birth defect
- is an important medical event that may jeopardise the patient or may require medical intervention to prevent one to the outcomes listed above.

Please note the following:

- ***Life-threatening*** refers to an event in which the patient was at risk of death at the time of the event; it does not refer to an event which hypothetically might have caused death if it were more severe.
- ***Hospitalisation*** describes a period of at least 24 hours. Over-night stay for observation, stay at emergency room or treatment on an out-patient basis do not constitute a hospitalisation. However, medical judgement must always be exercised and when in doubt the case should be considered as serious (i.e. if case fulfils the criterion for a medically important event). Hospitalisation for administrative or social purpose does not constitute a serious adverse event. Hospital admissions and/or surgical operation planned before study inclusion are not considered adverse event if the illness or disease existed before the patient was enrolled in the study, provided that the condition did not deteriorate during the study.
- ***Disability/Incapacity*** means a substantial disruption of a person's ability to conduct normal life

functions. In doubt, the decision should be left to medical judgement by the investigator.
- ***Important medical events*** are events that may not be immediately life-threatening, or result in death or hospitalisation but may jeopardise the patient or may require intervention to prevent one of the other outcomes listed in the definitions above. Examples of important medical events include events that suggest a significant hazard, contraindication or precaution, occurrence of malignancy or development of drug dependency or drug abuse. Medical and scientific judgement should be exercised in deciding whether events qualify as medically important.
An adverse event caused by an *overdose* is considered serious if a criterion listed in the definition above is fulfilled.

Serious Adverse Event

Enter the main adverse event as a diagnosis if available. If not, enter the separate sign(s) and symptom(s) which resulted in this report. Write **one** diagnosis/symptom per line. Please note that the AE verbatim on the SAE Form must match the AE verbatim on the Adverse Event Form.
- Note that death is not an event but the cause of death is the event
- Note that procedures should be captured along with the reason for conducting the procedures. The reason (e.g. appendicitis) should be entered as the main event and the procedure (e.g. appendicectomy) should be entered as a treatment.
- Note that pre-existing conditions, elective surgery and decreases in intensity are not adverse events.

Enter *onset* date. Onset date is the date when the first sign(s) or symptom(s) were noted (e.g. if the patient was hospitalised for meningitis, and symptoms such as fever, headache, nausea started before the date of hospitalisation, the onset date should be the day before hospitalisation when the first symptoms started).
If the adverse event is an abnormal laboratory test (such as "platelets low"), enter onset date as the date of the analysis.
Enter *stop date*, if applicable. If the event is not resolved enter "NA" (not applicable). *Duration* less than 24 hours should be entered in hours (or minutes if applicable).
Enter *intensity*: according to following definition:
Mild: Awareness of signs and symptoms but no disruption of usual activity. Symptoms do not require therapy or a medical evaluation; signs and symptoms are transient.
Moderate: Event sufficient to affect usual activity (disturbing). Are usually improved by simple therapeutic measures.
Severe: Inability to work or perform usual activities (unacceptable). Generally require systemic drug therapy or other treatment.
Enter *outcome* (died, recovered, recovered with sequelae, not yet recovered). If the patient recovered with sequelae, specify so in the narrative field. If the outcome is "not yet recovered", remember to follow-up on the outcome.

Study drug / Suspected drugs

Enter the study drug (with dose taken at the time of the event) as main suspect drug. If other drugs than the study drug is suspected to have caused the event, please add these drugs as additional suspected drugs.

If the study is blinded, enter "code not broken" and the different therapeutic options e.g. Drug X/Placebo.

Enter the *treatment number*.

Enter *dose/unit/frequency/route*. If treatment differs from the protocol, please specify the difference in the narrative.

Enter date for treatment initiation, and if applicable date for discontinuation. If therapy is ongoing, enter "on going".

Enter date of last dose prior event: if less than 24 hours, precise the duration in hours (or minutes if applicable).

Enter the investigator's assessment of the *causal relationship* between the event and the investigational drug(s) according to following definition:

Related / likely: Clearly related to the investigational agent / procedure, i.e. an event that follows a reasonable temporal sequence from administration of the study intervention, follows a known or expected response pattern to the suspected intervention, that can be confirmed by improvement on stopping and reappearance of the event after rechallenge and that could not be reasonably explained by the known characteristics of the subject's clinical state.

Possibly related / Possible: Follows a reasonable temporal sequence from administration of the study intervention, follows a known or expected response pattern to the suspected intervention, but that could readily have been produced by a number of other factors.

Not related / Unlikely: Clearly and incontrovertibly due only to extraneous causes, and does not meet criteria listed under possible (possibly related) or likely (related).

Narrative and documents

Describe the cause of event(s) as accurately and thoroughly as possible. The scope of the narrative is to give a full overview of the cause of event(s), making the case understandable to people without existing knowledge of the patient/subject and without access to the patient's/subject's chart.

The narrative should include information on signs and symptoms, relevant tests and outcome.

If the patient was hospitalized, resume the relevant information of the hospitalization report.

Remember that information on adverse effects are collected in order to establish the safety profile of the drug and that the information collected will be included in the labelling. It is therefore imperative that the information is of high data quality/consistency as the labelling should reflect the safety profile of the drug accurately and adequately.

Follow-up notification

If you have any further relevant information after the initial notification, please complete the complementary section immediately and send it like the initial notification.

Enter *patient identification number* (patient number or screening number or randomisation number).

Appendix 13: Beck Depression Inventory – 13 Items (BDI-13)

Short form of the Beck Depression Inventory

Instructions

This is a questionnaire. On the questionnaire are groups of statements. Please read the entire group of statements in each category. Then tick out the one statement in that group which best describes the way you feel today, that is, *right now!* Circle the number beside the statement you have chosen. If several statements in the group seem to apply equally well, circle each one.

A. (Sadness)
0 - I do not feel sad
1 - I feel sad or blue
2 - I am blue or sad all the time and I can't snap out of it
3 - I am so sad or unhappy that I can't stand it

B. (Pessimism)
0 - I am not particularly pessimistic or discouraged about the future
1 - I feel discouraged about the future
2 - I feel I have nothing to look forward to
3 - I feel that the future is hopeless and that things cannot improve

C. (Sense of Failure)
0 - I do not feel like a failure
1 - I feel I have failed more than the average person
2 - As I look back on my life, all I can see is a lot of failures
3 - I feel I am a complete failure as a person (parent, husband, wife)

D. (Dissatisfaction)
0 - I am not particularly dissatisfied
1 - I don't enjoy things the way I used to
2 - I don't get satisfaction out of anything anymore
3 - I am dissatisfied with everything

E. (Guilt)
0 - I don't feel particularly guilty
1 - I feel bad or unworthy a good part of the time
2 - I feel quite guilty
3 - I feel as though I am very bad or worthless

F. (Self-Dislike)
0 - I don't feel disappointed in myself
1 - I am disappointed in myself
2 - I am disgusted with myself
3 - I hate myself

G. (Self-Harm)
- 0 - I don't have any thoughts of harming myself
- 1 - I feel I would be better off dead
- 2 - I have definite plans about committing suicide
- 3 - I would like kill myself if I had the chance

H. (Social Withdrawal)
- 0 - I have not lost interest in other people
- 1 - I am less interested in other people than I used to be
- 2 - I have lost most of my interest in other people and have little feeling for them
- 3 - I have lost all of my interest in other people and don't care about them at all

I. (Indecisiveness)
- 0 - I make decisions about as well as ever
- 1 - I try to put off making decisions
- 2 - I have great difficulty in making decisions
- 3 - I can't make any decisions at all any time

J. (Self-Image Change)
- 0 I don't feel I look any worse than I used to
- 1 I am worried than I am looking old or unattractive
- 2 I feel that there are permanent changes in my appearance and they make me look unattractive
- 3 I feel that I am ugly or repulsive looking

K. (Work Difficulty)
- 0 - I can work about as well as before
- 1 - It takes extra effort to get started at doing something
- 2 - I have to push myself very hard to do anything
- 3 - I can't do any work at all

L. (Irritability)
- 0 - I am no more irritated by things than I ever am
- 1 - I am slightly more irritated now than usual.
- 2 - I am quite annoyed or irritated a good deal of the time.
- 3 - I feel irritated all the time now.

M. (Anorexia)
- 0 - My appetite is no worse than usual
- 1 - My appetite is not as good as it used to be
- 2 - My appetite is much worse now
- 3 - I have no appetite at all any more

Scoring:

0 – 4 : none or minimal depression
4 – 7 : mild depression
8 – 15: moderate depression
≥ 16 : severe depression

Appendix 14: Patient's overall evaluation of the tolerance

PATIENT'S OVERALL EVALUATION OF THE TOLERANCE
❒ Good<br>❒ Moderate<br>❒ Poor

Appendix 15: Amphetamine-Like Withdrawal Symptoms Questionnaire (DSM IV)

AMPHETAMINE LIKE WITHDRAWAL SYMPTOMS		
Dysphoria	❒ yes	❒ no
Fatigue	❒ yes	❒ no
Vivid and unpleasant dreams	❒ yes	❒ no
Insomnia or hypersomnia	❒ yes	❒ no
Increased appetite	❒ yes	❒ no
Psychomotor retardation or agitation	❒ yes	❒ no

Appendix 16: Mini Mental State Examination Questionnaire (MMSE)

The Mini mental state examination (MMSE) is a brief 30-point questionnaire test that is used to screen for cognitive impairment. In the time span about 10 minutes it samples various functions including arithmetic, memory and orientation. Any score over 28 (out of 30) is effectively normal. Below this, 20-26 indicates some cognitive impairment; 10-19 moderate to severe cognitive impairment, and below 10 very severe cognitive impairment. The MMSE will be performed at V1. The patient will not be included in the study if a score of MMSE<28.

**MMSE (1 / 2)**	
**1. Orientation** *point for each correct answer (maximum 10 points)*	
1. What is the year?	I__I
2. What is the season?	I__I
3. What is the month?	I__I
4. What is the date?	I__I
5. What is the day?	I__I
6. Where are we? (country)	I__I
7. Where are we? (county)	I__I
8. Where are we? (town)	I__I
9. Where are we? (which hospital)	I__I
10. Where are we? (which floor)	I__I
**2. Registration** Name three common objects (e.g. "apple", "table" and "penny"). Take one second to say each one. Then ask the patient to repeat all three after you have said them. Give one point for each correct answer. Repeat the object names until all three are learned (up to 6 trials) *(maximum 3 points)*	
11. "Apple"	I__I
12. "Table"	I__I
13. "Penny"	I__I
**3. Attention and Calculation**	
14. Spell "world" backwards. Give one point for each letter that is in the right place. (D_L_R_O_W). *(maximum 5 points)*	I__I
Alternatively, do serial 7s: Ask the person to count backwards from 100 in blocks of 7. Stop after 5 subtractions. Give one point for each correct answer. If one answer is incorrect (e.g. 92) but the following answer is 7 less than the previous answer (i.e.), count the second answer as correct. *(maximum 5 points)*	
14. « 93 »	I__I
15. « 86 »	I__I
16. « 79 »	I__I
17. « 72 »	I__I
18. « 65 »	I__I

**MMSE (2 / 2)**	
**4. Recall** Ask for the 3 objects repeated above (e.g., apple, table and penny). Give 1 point for each correct object *(maximum 3 points).*	
19. "Apple"	I__I
20. "Table"	I__I
21. "Penny"	I__I
**5. Language**	
22. Point to a pencil and ask the person to name this object (1 point)	I__I
23. Do the same thing with a wrist-watch (1 point)	I__I
24. Ask the patient to repeat the following sentence: "No ifs, ands or buts". Allow only one trial (1 point)	I__I
Give the patient a piece of blank white paper and ask them to follow a 3 stage command (1 point for each command correctly followed) :	
25. "Take a paper in your right hand'	I__I
26. "Fold it in half"	I__I
27. " Put it on the floor"	I__I
28. Write "CLOSE YOUR EYES" in large letter and show it to the patient. Ask him or her to read the message and do what it says (give 1 point if they actually close their eyes)	I__I
29. Ask the patient to write a sentence of their choice on a blank of paper. The sentence must contain a subject and a verb and must make sense. Spelling, punctuation and grammar are not important (1 point)	I__I
30. Ask the patient to copy the design exactly as it is (1 point) 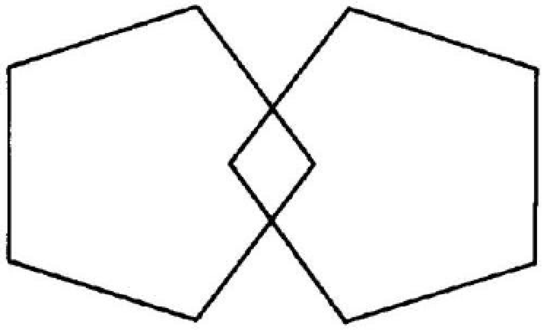	I__I
Total Score	I__I__I

Efficacy and Safety of Pitolisant (BF2.649) in the Treatment of Excessive Daytime Sleepiness in Patients with Obstructive Sleep Apnoea Syndrome, Treated or Not by Nasal Continuous Positive Airway Pressure, but Still Complaining of Excessive Daytime Sleepiness – HAROSA III

Amendment 1
(Protocol version 1.2, 29 Jan 2018)

Rationale for the amendment and description of changes from version 1.0 (dated 02 Dec 2015) to version 2.0 (01 Feb 2018)

Rationale of the Amendment 1

The current HAROSA III clinical trial was originally designed as to provide evidence of the efficacy of pitolisant (BP2.649) compared with placebo in excessive daytime sleepiness (EDS) measured by Epworth ESS scale. Bioprojet Pharma wishes to strengthen and enhance the level of evidence of this trial in assessing separately the efficacy of the studied drug both on Continuous Positive Airway Pressure (CPAP) and non-CPAP use. The statistical considerations of this trial were reviewed under this new objective as follows:
Results from exploratory studies on pitolisant allow to estimate the ESS residual variability to SD = 6. The Minimum Important Difference (MID) was fixed to ESS = 3, corresponding to an effect size ES = 0.5. The correlation between Final and baseline ESS was conservatively estimated to r = 0.3.
a) By assuming ANCOVA test at 0.95 confidence level as the main confirmatory test, and a sample ratio 1:2, a difference of at least Delta = 3 should be detected with a power of 90% in using at least 60 patients in placebo group and 120 patients in pitolisant treatment group.
b) By assuming the same model, an interaction of at least 3 between CPAP and non-CPAP will be detected with a power of 90% when 120 and 240 patients (thus 360 patients in total) are treated in Placebo and pitolisant group respectively. The null hypothesis here should be:
Interaction $(Y_{pt}\text{-}Y_{pl})_{CPAP}$ - $(Y_{pt}\text{-}Y_{pl})_{non\text{-}CPAP} > 3$
Under these two conditions, a stepdown test can be organized: first a test of superiority of pitolisant will be conducted. If this test concludes into superiority, the interaction test will be conducted.
The sample size will be 120 and 240, (thus at least 360 patients in total) as this size corresponds to the interaction test requiring the largest sample size.

Description of the Amendment 1

The first objective of the study has been modified to specify that the efficacy of pitolisant will be assessed separately in patients treated with CPAP and in patients without CPAP use.
Consequently the sample size of the study was increased from 180 to 360 to be analysed, i.e., 180 patients presenting with Obstructive Sleep Apnea Syndrome (OSA) complaining of excessive daytime sleepiness (EDS) and refusing nasal Continuous Positive Airway Pressure (nCPAP), and 180 patients with OSA treated by nCPAP but still complaining of EDS. The randomization will be 2:1 (active:placebo) leading to 4 groups:

- 120 patients with OSA complaining of EDS refusing the nCPAP therapy will be administered pitolisant active ingredient product
- 60 patients with OSA complaining of EDS refusing the nCPAP therapy will be administered a placebo
- 120 patients with OSA treated by nCPAP but still complaining of EDS will be administered pitolisant active ingredient product
- 60 patients with OSA treated by nCPAP but still complaining of EDS will be administered a placebo.

Changes made in the study protocol are further described; in the “old text” sections, additions of text are in bold and deletions are crossed in the text. All are highlighted in grey.
The Informed Consent Forms (i.e., for Double blind and Open Label periods) have been updated accordingly.

1. Synopsis, "International Coordinator and Investigators"

Old text:

Several **investigator** site**s** ~~investigators~~ in **Europe** ~~Bulgaria~~.

New text:

Several investigator sites in Europe.

1. Synopsis, "Study Objectives"

Old text:

The first objective of this study is to demonstrate the efficacy and safety of pitolisant given at 10, 20, or 40 mg per day versus placebo during 12 weeks of the Double Blind period, to treat the Excessive Daytime Sleepiness (EDS) in patients with Obstructive Sleep Apnea (OSA) refusing the nasal Continuous Positive Airway Pressure (nCPAP) therapy or treated by nCPAP but still complaining of EDS. **The efficacy of pitolisant will be assessed separately in patients treated with CPAP and in patients without CPAP use.**

New text:

The first objective of this study is to demonstrate the efficacy and safety of pitolisant given at 10, 20, or 40 mg per day versus placebo during 12 weeks of the Double Blind period, to treat the Excessive Daytime Sleepiness (EDS) in patients with Obstructive Sleep Apnea (OSA) refusing the nasal Continuous Positive Airway Pressure (nCPAP) therapy or treated by nCPAP but still complaining of EDS. The efficacy of pitolisant will be assessed separately in patients treated with CPAP and in patients without CPAP use.

1. Synopsis, "Number of patients"

Old text:

Approximately **400** ~~200~~ patients will be selected, so that **360** ~~180~~ patients can be analyzed.

They will be divided into 4 groups as follows:

- **120** ~~60~~ patients with OSA complaining of EDS refusing the nCPAP therapy will be administered pitolisant active ingredient product;
- **60** ~~30~~ patients with OSA complaining of EDS refusing the nCPAP therapy will be administered a placebo;
- **120** ~~60~~ patients with OSA treated by nCPAP but still complaining of EDS will be administered pitolisant active ingredient product;
- **60** ~~30~~ patients with OSA treated by nCPAP but still complaining of EDS will be administered a placebo.

New text:

Approximately 400 patients will be selected, so that 360 patients can be analyzed.

They will be divided into 4 groups as follows:

- 120 patients with OSA complaining of EDS refusing the nCPAP therapy will be administered pitolisant active ingredient product;
- 60 patients with OSA complaining of EDS refusing the nCPAP therapy will be

administered a placebo;
- 120 patients with OSA treated by nCPAP but still complaining of EDS will be administered pitolisant active ingredient product;
- 60 patients with OSA treated by nCPAP but still complaining of EDS will be administered a placebo.

1. Synopsis, "Main non-inclusion criteria"

Old text:

The patients should not present any of the following criteria:

- **Patients having previously been exposed to pitolisant either in previous clinical trials, or in a compassionate program or being prescribed the commercial form (Wakix®), for patients enrolled from January 2018**
- Patients suffering from chronic severe insomnia in accordance with the International Classification of Sleep Disorders (ICSD 2005) without OSA

New text:

The patients should not present any of the following criteria:

- Patients having previously been exposed to pitolisant either in previous clinical trials, or in a compassionate program or being prescribed the commercial form (Wakix®), for patients enrolled from January 2018
- Patients suffering from chronic severe insomnia in accordance with the International Classification of Sleep Disorders (ICSD 2005) without OSA

1. Synopsis, "Statistical analysis"

Old text:

Sample Size Determination and Justification:

Results from exploratory studies on pitolisant allow to estimate the ESS residual variability to SD = 6. The Minimum Important Difference MID was fixed to ESS = 3, corresponding to an effect size ES = 0.~~,~~5. The correlation between Final and Baseline ESS was conservatively estimated to r = 0.~~,~~3.

a) By assuming Analysis of Covariance ~~test~~ (ANCOVA) **test** at 0.95 confidence level as the main confirmatory test, and a sample ratio 1:2, a difference of at least Delta = 3 should be detected with a power of 90% in using at least 60 patients in placebo group and 120 patients in pitolisant treatment group.

b) By assuming the same model, an interaction of at least 3 between CPAP and non-CPAP will be detected with a power of 90% when 120 and 240 patients (thus 360 patients in total) are treated in Placebo and pitolisant group respectively. The null hypothesis here should be
Interaction $(Y_{pt}-Y_{pl})_{CPAP} - (Y_{pt}-Y_{pl})_{non-CPAP} > 3$

~~b) Treatment groups will be stratified by center and CPAP use. Considering 10% drop out rate, 200 patients will be selected.~~

Under these two conditions, a stepdown test can be organized: first a test of superiority of pitolisant will be conducted. If this test concludes into superiority, the interaction test will be conducted.

The sample size will be 120 and 240, (thus at least 360 patients in total) as this size

corresponds to the interaction test requiring the largest sample size.

Statistical Analysis:

Final ESS will be compared between the two treatments by an ANCOVA at two-sided 95% in adjusting for ESS at baseline, and by considering the random effect center, and the fixed effect treatment and assessing the additional effect of obesity (BMI) on outcome. This test will be implemented by a Mixed Linear Model. The confirmatory analysis will be based on a simple ANCOVA model assuming no interaction between baseline and treatment (assumption of parallelism). **This analysis will be conducted both on the CPAP and non-CPAP groups as primary selections.**

Safety and tolerability will be assessed by summarizing and analyzing adverse events (AEs), change in physical examination, vital signs, electrocardiogram (ECG) and laboratory data.

New text:

Sample Size Determination and Justification:

Results from exploratory studies on pitolisant allow to estimate the ESS residual variability to SD = 6. The Minimum Important Difference MID was fixed to ESS = 3, corresponding to an effect size ES = 0.5. The correlation between Final and Baseline ESS was conservatively estimated to r = 0.3.

a) By assuming Analysis of Covariance (ANCOVA) test at 0.95 confidence level as the main confirmatory test, and a sample ratio 1:2, a difference of at least Delta = 3 should be detected with a power of 90% in using at least 60 patients in placebo group and 120 patients in pitolisant treatment group.
b) By assuming the same model, an interaction of at least 3 between CPAP and non-CPAP will be detected with a power of 90% when 120 and 240 patients (thus 360 patients in total) are treated in Placebo and pitolisant group respectively. The null hypothesis here should be
   Interaction $(Y_{pt}-Y_{pl})_{CPAP}$ - $(Y_{pt}-Y_{pl})_{non\text{-}CPAP} > 3$

Under these two conditions, a stepdown test can be organized: first a test of superiority of pitolisant will be conducted. If this test concludes into superiority, the interaction test will be conducted.

The sample size will be 120 and 240, (thus at least 360 patients in total) as this size corresponds to the interaction test requiring the largest sample size.

Statistical Analysis:

Final ESS will be compared between the two treatments by an ANCOVA at two-sided 95% in adjusting for ESS at baseline, and by considering the random effect center, and the fixed effect treatment and assessing the additional effect of obesity (BMI) on outcome. This test will be implemented by a Mixed Linear Model. The confirmatory analysis will be based on a simple ANCOVA model assuming no interaction between baseline and treatment (assumption of parallelism). This analysis will be conducted both on the CPAP and non-CPAP groups as primary selections.

Safety and tolerability will be assessed by summarizing and analyzing adverse events (AEs), change in physical examination, vital signs, electrocardiogram (ECG) and laboratory data.

**1. Synopsis, "Planned End of the Study"**
Old text:<br>Last patient last visit: 2 Q **2019** ~~2017~~
New text:<br>Last patient last visit: 2 Q 2019

**2. Study diagram / Open Label Extension period**
Old text:<br>…/…<br>5 – At each visit, the patient shall bring back his sleep diary. Patient will be contacted in advance before e visit to remind him/her to fill in the sleep diary. The patient shall return the unused drug at each visit excep V13.<br>6 – for **a pre-defined number of** ~~all~~ patients.
New text:<br>…/…<br>5 – At each visit, the patient shall bring back his sleep diary. Patient will be contacted in advance before e visit to remind him/her to fill in the sleep diary. The patient shall return the unused drug at each visit excep V13.<br>6 – for a pre-defined number of patients.

**5. Study objectives**
Old text:<br>The first objective of this study is to demonstrate the efficacy and safety of pitolisant given at 10, 20, or 40 mg per day versus placebo during 12 weeks for the Double Blind period, to treat the EDS in patients with OSA refusing the nCPAP therapy or treated by nCPAP but still complaining of EDS. **The efficacy of pitolisant will be assessed separately in patients treated with CPAP and in patients without CPAP use.**
New text:<br>The first objective of this study is to demonstrate the efficacy and safety of pitolisant given at 10, 20, or 40 mg per day versus placebo during 12 weeks for the Double Blind period, to treat the EDS in patients with OSA refusing the nCPAP therapy or treated by nCPAP but still complaining of EDS. The efficacy of pitolisant will be assessed separately in patients treated with CPAP and in patients without CPAP use.

**6. Population / 6.2. Non-inclusion criteria**
Old text:<br>All subjects included in the study must not meet any of the following non-inclusion criteria:<br>- **Patients having previously been exposed to pitolisant either in previous clinical trials, or in compassionate program or being prescribed the commercial form**

**(Wakix®), for those enrolled patients from January 2018**<br>- Patients suffering from chronic severe insomnia in accordance with the International Classification of Sleep Disorders (ICSD 2005) without OSA
New text:<br>All subjects included in the study must not meet any of the following non-inclusion criteria:<br>- Patients having previously been exposed to pitolisant either in previous clinical trials, or in compassionate program or being prescribed the commercial form (Wakix®), for those enrolled patients from January 2018<br>- Patients suffering from chronic severe insomnia in accordance with the International Classification of Sleep Disorders (ICSD 2005) without OSA

**7. Experimental design**
Old text:<br>This is a prospective, multicenter (in **Europe** ~~Bulgaria~~), randomised, Double Blind **phase** ~~period~~ III study versus placebo.<br>…/…<br>The study will include about **400** ~~200~~ patients. This number will allow having **360** ~~180~~ completed treated patients (pitolisant, patients refusing nCPAP therapy = **120** ~~60~~; placebo, patients refusing nCPAP therapy = **60** ~~30~~, pitolisant, patients treated by nCPAP = **120** ~~60~~; placebo, patients treated by nCPAP = **60** ~~30~~) sufficient for the results analysis of efficacy during the Double Blind period (12 weeks).
New text:<br>This is a prospective, multicenter (in Europe), randomised, Double Blind phase III study versus placebo.<br>…/…<br>The study will include about 400 patients. This number will allow having 360 completed treated patients (pitolisant, patients refusing nCPAP therapy = 120; placebo, patients refusing nCPAP therapy = 60, pitolisant, patients treated by nCPAP = 120; placebo, patients treated by nCPAP = 60) sufficient for the results analysis of efficacy during the Double Blind period (12 weeks).

**8. Study sites**
Old text:<br>This trial will be conducted under the responsibility of [redacted] UMHAT "Alexandrovska" – 1, Sv. Georgi Sofiyski Str., Sofia, Bulgaria – [redacted]. This multicentre study will be conducted in several investigational centres in **Europe** ~~Bulgaria~~; they will be opened, in hospitals and sleep disorder centres which will all have the ability to evaluate the efficacy and tolerance of the drug for the treatment of EDS in OSA patients.

New text:

This trial will be conducted under the responsibility of PPDPPDPPDPPD UMHAT “Alexandrovska” – 1, Sv. Georgi Sofiyski Str., Sofia, Bulgaria – PPDPPD PPDPPDPPDPPDPPDPPDPPDPPDPPDPPDPPDPPD This multicentre study will be conducted in several investigational centres in Europe; they will be opened, in hospitals and sleep disorder centres which will all have the ability to evaluate the efficacy and tolerance of the drug for the treatment of EDS in OSA patients.

10. Evaluation criteria / 10.2. Safety criteria / 10.2.14. Safety endpoints / 10.2.14.8. Laboratory tests

Old text:

Urinalysis will be performed using stick test at V1. The results will be recorded in the CRF. Should the stick test be positive, a mid stream sample of urine will be sent for microscopy and bacteriological culture.

Pharmacokinetic sampling will be done at V10 **for a number of patients (no samples to be done during the study extension)**. Samples will be taken at pre-dose, 1,5 h, 3 h and 8 h post-dose (see §10.2.14.8~~9~~ below).

Any laboratory test abnormalities considered clinically significant putting the patient at risk, as judged by the investigator, and if necessary by the scientific committee will lead to immediate discontinuation of the study drug. The Sponsor must be informed immediately.

New text:

Urinalysis will be performed using stick test at V1. The results will be recorded in the CRF. Should the stick test be positive, a mid stream sample of urine will be sent for microscopy and bacteriological culture.

Pharmacokinetic sampling will be done at V10 for a number of patients (no samples to be done during the study extension). Samples will be taken at pre-dose, 1,5 h, 3 h and 8 h post-dose (see §10.2.14.8 below).

Any laboratory test abnormalities considered clinically significant putting the patient at risk, as judged by the investigator, and if necessary by the scientific committee will lead to immediate discontinuation of the study drug. The Sponsor must be informed immediately.

10. Evaluation criteria / 10.2. Safety criteria / 10.2.14. Safety endpoints / 10.2.14.9. Pharmacokinetic assessments

Old text:

Pharmacokinetic sampling will be done at V10 **for a number of patients (no samples to be done during the study extension)**. Blood samples for PK analysis will be performed at pre-dose, 1.5 h, 3 h and 8 h post-dose.

New text:

Pharmacokinetic sampling will be done at V10 for a number of patients (no samples to be done during the study extension). Blood samples for PK analysis will be performed at pre-dose, 1.5 h, 3 h and 8 h post-dose.

12. Conduct of the trial

Old text:

This is a prospective, multicenter (several sites in **Europe** ~~Bulgaria~~), randomised, double blind study versus placebo.

…/…

The study will include about **400** ~~200~~ patients. This number will allow having sufficient completed treated patients – **360** ~~180~~ (pitolisant = **240** ~~120~~; placebo = **120** ~~60~~) for the per protocol analysis of efficacy during the Double Blind period (12 weeks).

New text:

This is a prospective, multicenter (several sites in Europe), randomised, double blind study versus placebo.

…/…

The study will include about 400 patients. This number will allow having sufficient completed treated patients – 360 (pitolisant = 240; placebo = 120) for the per protocol analysis of efficacy during the Double Blind period (12 weeks).

12. Conduct of the trial / 12.1 Part I – Double Blind period / 12.1.1. V1 – Screening visit and beginning of initial wash-out period (D – 14)

Old text:

The investigator will perform the following screening assessments:

- Medical questionnaire including the patient demographic data, OSA and EDS history and documentation of the previously performed tests/questionnaires about illness (such as polysomnography, AHI, Epworth score, OSleR test, BMI, state of sleep-related desaturation), actual symptoms prior to admission and their duration, possible complications, details of concomitant treatments including any current and previous treatments of EDS and any other types of treatment.
- **For patients using nCPAP therapy, when possible, the observance prior to screening will be assessed by the clock-time counter of the CPAP machine for a minimum period of 3 months. If available, a printout report should be archived in the patient's medical file. Otherwise it should be documented in the patient's chart through the patient's medical interview during the screening visit.**
- A complete physical examination, in particular vital signs including blood pressure, heart rate, respiratory rate.

New text:

The investigator will perform the following screening assessments:

- Medical questionnaire including the patient demographic data, OSA and EDS

history and documentation of the previously performed tests/questionnaires about illness (such as polysomnography, AHI, Epworth score, OSleR test, BMI, state of sleep-related desaturation), actual symptoms prior to admission and their duration, possible complications, details of concomitant treatments including any current and previous treatments of EDS and any other types of treatment.

- For patients using nCPAP therapy, when possible, the observance prior to screening will be assessed by the clock-time counter of the CPAP machine for a minimum period of 3 months. If available, a printout report should be archived in the patient's medical file. Otherwise it should be documented in the patient's chart through the patient's medical interview during the screening visit.
- A complete physical examination, in particular vital signs including blood pressure, heart rate, respiratory rate.

13. Statistical analysis / 13.1. Summary

Old text:

Statistical Analysis: final ESS will be compared between the two treatments by an ANCOVA in adjusting for ESS at baseline, and by considering the random effect center, and the fixed effect treatment. This test will be implemented by a Mixed Linear Model. **This analysis will be conducted both on the CPAP and non-CPAP groups as primary selections.**

New text:

Statistical Analysis: final ESS will be compared between the two treatments by an ANCOVA in adjusting for ESS at baseline, and by considering the random effect center, and the fixed effect treatment. This test will be implemented by a Mixed Linear Model. This analysis will be conducted both on the CPAP and non-CPAP groups as primary selections.

13. Statistical analysis / 13.2. Sample size determination and justification

Old text:

Results from exploratory studies on pitolisant allow to estimate the ESS residual variability to standard deviation (SD) = 6. The Minimum Important Difference (MID) was fixed to ESS = 3, corresponding to an effect size (ES) = 0.~~,~~5. The correlation between final and baseline ESS was conservatively estimated to r = 0.~~,~~3.

a) By assuming ANCOVA at 0.~~,~~95 confidence level as the main confirmatory test, **and a sample ratio 1:2,** a difference of at least Delta = 3 should be detected with a power of 90% in using at least **60** ~~30~~ patients in each placebo group ~~(60 in total)~~ and **120** ~~60~~ patients in each pitolisant treatment group ~~(120 in total)~~.

b) By assuming the same model, an interaction of at least 3 between CPAP and non-CPAP will be detected with a power of 90% when 120 and 240 patients (thus 360 patients in total) are treated in Placebo and pitolisant group, respectively. The null hypothesis here should be

Interaction $(Y_{pt}-Y_{pl})_{CPAP} - (Y_{pt}-Y_{pl})_{non\text{-}CPAP} > 3$

Under these two conditions, a stepdown test can be organized: First a test of superiority of pitolisant will be conducted. If this test concludes into superiority, the interaction test will be conducted.

The sample size will be 120 and 240 (thus at least 360 patients in total) as this size corresponds to the interaction test requiring the largest sample size. ~~Treatment groups will be stratified by center and CPAP use.~~ Considering 10% drop out rate, **approximately 400** ~~200~~ patients will be selected.

New text:

Results from exploratory studies on pitolisant allow to estimate the ESS residual variability to standard deviation (SD) = 6. The Minimum Important Difference (MID) was fixed to ESS = 3, corresponding to an effect size (ES) = 0.5. The correlation between final and baseline ESS was conservatively estimated to r = 0.3.

a) By assuming ANCOVA at 0.95 confidence level as the main confirmatory test, and a sample ratio 1:2, a difference of at least Delta = 3 should be detected with a power of 90% in using at least 60 patients in each placebo group and 120 patients in each pitolisant treatment group.
b) By assuming the same model, an interaction of at least 3 between CPAP and non-CPAP will be detected with a power of 90% when 120 and 240 patients (thus 360 patients in total) are treated in Placebo and pitolisant group, respectively. The null hypothesis here should be

Interaction $(Y_{pt}\text{-}Y_{pl})_{CPAP}$ - $(Y_{pt}\text{-}Y_{pl})_{non\text{-}CPAP}$ > 3

Under these two conditions, a stepdown test can be organized: First a test of superiority of pitolisant will be conducted. If this test concludes into superiority, the interaction test will be conducted.

The sample size will be 120 and 240 (thus at least 360 patients in total) as this size corresponds to the interaction test requiring the largest sample size. Considering 10% drop out rate, approximately 400 patients will be selected.

13. Statistical analysis / 13.5. Futility analysis

Old text:

During the first part of the study, a ~~A~~ one-stage Futility stopping **was to** ~~will~~ be **done** based on Conditional Power, probability to detect a significant result at the end of the Double Blind period, given the results observed at an intermediate time. **This analysis was done between pitolisant and placebo irrespective of CPAP use.**

Conditional Power will be estimated (Lan and Wittes 1988; Lan and Zucker 1993). This analysis **was** ~~will be~~ carried out by a third party statistician when at least 80 patients are available, and futility threshold will be CPmin=.10 involving a slight increase of type 2 error (Proschan 1999). This intermediate futility analysis **did** ~~does~~ not require any type-I adjustment, this trial **did** ~~does~~ not plan rejection of the null hypothesis before its end.

In the new context of the study, the question remains on the significance of the difference within each subgroup. A one-stage Futility stopping might be conducted. In this case the analysis should be based on the Conditional Power of the interaction test, probability to detect a significant result at the end of the study, given the results observed at an intermediate time. Conditional Power (CP) will be estimated through B-values and (Lan and Wittes 1988; Lan and Zucker 1993). This analysis will be carried out by a third party statistician when at least 180 patients are available, and futility threshold will be CPmin=.10 involving a slight increase of type 2 error (Proschan 1999). This intermediate futility analysis does not require any type-I adjustment, this trial does not plan rejection of the null hypothesis before its end.

New text:<br>During the first part of the study, a one-stage Futility stopping was to be done based on Conditional Power, probability to detect a significant result at the end of the Double Blind period, given the results observed at an intermediate time. This analysis was done between pitolisant and placebo irrespective of CPAP use.<br>Conditional Power will be estimated (Lan and Wittes 1988; Lan and Zucker 1993). This analysis was carried out by a third party statistician when at least 80 patients are available, and futility threshold will be CPmin=.10 involving a slight increase of type 2 error (Proschan 1999). This intermediate futility analysis did not require any type-I adjustment, this trial did not plan rejection of the null hypothesis before its end.<br>In the new context of the study, the question remains on the significance of the difference within each subgroup. A one-stage Futility stopping might be conducted. In this case the analysis should be based on the Conditional Power of the interaction test, probability to detect a significant result at the end of the study, given the results observed at an intermediate time. Conditional Power (CP) will be estimated through B-values and (Lan and Wittes 1988; Lan and Zucker 1993). This analysis will be carried out by a third party statistician when at least 180 patients are available, and futility threshold will be CPmin=.10 involving a slight increase of type 2 error (Proschan 1999). This intermediate futility analysis does not require any type-I adjustment, this trial does not plan rejection of the null hypothesis before its end.

**19. Calendar forecast**
Old text:<br>Last patient visit is scheduled in 2 Q **2019** ~~2017~~
New text:<br>Last patient visit is scheduled in 2 Q 2019

bioprojet PHARMA

Sponsor Study Reference: P1513 / BF2.649
EudraCT number: 2015-004561-85

NOTE TO FILE (NTF) N° 001

Issue:
Protocol Amendment 1, version 1.0 dated 01 February 2018 refers to Protocol version 1.2, 29 Jan 2018 in the title of the document while it should refer to Protocol version 2.0, 01 Feb 2018.

Impact of the issue:
- ☐ significantly affects subject protection
- ☐ significantly affects reliability of trial results
- ☐ potentially affects subject protection
- ☐ potentially affects reliability of trial results
- ☐ has consequence on the study conduct
- ☒ has no impact on the study conduct, subject protection and reliability of trial results

Root cause analysis:
This is a typo error related to the previous draft version of the document.

Action taken:
No action has been taken as the Amendment 1 was submitted in February 2018 in Bulgaria and did not lead to question or request of modification. Both EC and RA approvals were granted based on this version. No update is to be done to CA.

Comments:
The current Note to File will be filed with the original document in the study TMF.

Written by:

[redacted]

Name and signature

31OCT2018
Date (DD/MMM/YYYY)

Approved by:

[redacted]

Name and signature

31 OCT 2018
Date (DD/MMM/YYYY)

In case of major deviation, please transmit the noncompliance form to the Clinical QA:

☐ Non compliance (registration number :...)

Name and signature

Date (DD/MMM/YYYY)

Original to be retained in the TMF.
A photocopy to be retained in the ISF if the issue relates to the site and/or PI responsibilities.
