# Supplementary material for: Pitolisant 40 mg for excessive daytime sleepiness in obstructive sleep apnea patients treated or not by CPAP: Randomised phase 3 study
Source: J Sleep Res. 2024 Oct 8;34(3):e14373. doi: 10.1111/jsr.14373 (PMC12069729; doi:10.1111/jsr.14373)
Supplement: Supplementary file 4 — DATA S2. eSAP2 Statistical analysis plan. [file JSR-34-e14373-s005.pdf]

16.1.9 Documentation of statistical methods

16.1.9.1 Statistical Analysis Plans

The statistical analyses were performed according to the following Statistical Analysis Plans (SAP):

- Double Blind period, version 1.0 dated 08/01/2021
- Open Label period, version 3.0 dated 30/06/2021

Deviations to these plans occurred in the analyses and are described in the following documents:

Deviations for planned analysis for study HAROSA III – Double-Blind, version 3.0 dated 17/02/2022

Deviations for planned analysis for study HAROSA III – Double-Blind, version 2.0 dated 17/02/2022.

The documents are provided on the following pages.

Statistical Analysis Plan for the Double-Blind Part of Study P1513:

Efficacy and Safety of Pitolisant (BF2.649) in the Treatment of Excessive Daytime Sleepiness in Patients with Obstructive Sleep Apnoea Syndrome, Treated or Not by Nasal Continuous Positive Airway Pressure, but still complaining of Excessive Daytime Sleepiness

EUDRACT NUMBER: 2015-004561-85

Version	Date	Author	Job Title
0.1	30 March 2018	[redacted]	Statistician
0.2	05 January 2021		Statistician
1.0	08 January 2021		Statistician

For Approval:

Senior Consulting Statistician: [redacted]
Faculty of Economics
UCL University, Mons Signature ______________ Date ________
185, chaussée de Binche
B-7000 Mons
BELGIUM

Head of Clinical Development: [redacted]
Bioprojet
9, rue Rameau
F-75002 Paris
FRANCE

[redacted]

Signature Date

Statistical Analysis Plan for the Double-Blind Part of Study P1513:

Efficacy and Safety of Pitolisant (BF2.649) in the Treatment of Excessive Daytime Sleepiness in Patients with Obstructive Sleep Apnoea Syndrome, Treated or Not by Nasal Continuous Positive Airway Pressure, but still complaining of Excessive Daytime Sleepiness

EUDRACT NUMBER: 2015-004561-85

Version	Date	Author	Job Title
0.1	30 March 2018	[redacted]	Statistician
0.2	05 January 2021	[redacted]	Statistician
1.0	08 January 2021	[redacted]	Statistician

For Approval:

Senior Consulting Statistician:	Prof. Dr. [redacted]<br>Faculty of Economics<br>UCL University, Mons<br>185, chaussée de Binche<br>B-7000 Mons<br>BELGIUM	[redacted]<br>Signature	8-1-21<br>Date
Head of Clinical Development:	Dr. [redacted]<br>Bioprojet<br>9, rue Rameau<br>F-75002 Paris<br>FRANCE	____________<br>Signature	______<br>Date

Table of Contents

Table of Contents 2

1 Introduction 5

1.1 Study Design 5

1.2 Study Treatment 6

1.3 Objectives 6

1.4 Sample Size Justification 6

2 Data Considered for the Analysis 7

2.1 Data Only Recorded at Onset of the Study 7

2.2 Efficacy Data 8

2.3 Safety Data 11

2.4 Course of the Study 14

2.5 Study Treatment and Compliance 15

3 Statistical Methodology 17

3.1 Statistical Software 17

3.2 Analysis Sets 17

3.3 Protocol Deviations 17

3.4 Baseline 17

3.5 Missing Data 17

3.6 Summary Statistics 18

3.7 Inferential Aspects 18

3.7.1 Notes 20

3.7.2 Reporting format 20

3.8 Accounting for Multiple Centers 20

3.9 Outcomes and Multiple Testing 20

3.10 Intermediate Analyses 20

3.11 Changes from the Analyses Foreseen in the Protocol 21

3.12 Miscellaneous .... **Error! Bookmark not defined.**

4 Contents Description 21

4.1 Sample Description 21

4.2 Course of the Study 22

4.3 Primary Efficacy Variable and Related Variables (ESS) 22

4.4 Secondary Efficacy Variables 22

4.4.1 OSleR Test 22

4.4.2 Clinical Global Impression (CGI) and Patient Global Impression(PGI) 23

4.5 Exploratory endpoints 23

4.5.1 Aggregate z-score of ESS and OSleR 23

4.5.2 Therapy Response 23

4.5.3 Sleep Diary 23

4.5.4 Quality of Life Test Euro Qol-5D (EQ-5D) 24

4.5.5 Leeds Sleep Evaluation Questionnaire (LSEQ) , Trail Making Tests A&B, and Pichot Fatigue 24

4.6 Safety Analysis 24
4.6.1 Adverse Events 24
4.6.2 Laboratory Evaluations 25
4.6.3 Vital Signs 25
4.6.4 Physical Examination 25
4.6.5 ECG Data 25
4.6.6 Beck Depression Inventory (BDI) 26
4.6.7 Amphetamine-like Withdrawal Symptoms 26
4.6.8 Patient's Overall Evaluation of Tolerance 26
4.7 Concomitant Medications 26
4.8 Exposure, Dosing, and Compliance 26
5 Statistical Tables 27
5.1 Demography and Baseline Characteristics 27
5.1.1 Demography 27
5.1.2 Medical History 27
5.1.3 Obstructive Sleep Apnoea (OSA) and Excessive Daytime Sleepiness 27
5.1.4 Nocturnal Sleep 27
5.1.5 MMSE 27
5.1.6 Polysomnography 27
5.2 Efficacy 28
5.2.1 Primary Efficacy Variable and Related Variables 28
5.2.2 Secondary Efficacy Variables 28
5.3 Safety 30
5.3.1 Adverse Events 30
5.3.2 Laboratory Evaluations 31
5.3.3 Vital Signs 31
5.3.4 Physical Examination 31
5.3.5 ECG Data 31
5.3.6 BDI 31
5.3.7 Amphetamine-like Withdrawal Symptoms 32
5.3.8 Patient's Overall Evaluation of Tolerance 32
5.4 Concomitant Medications 32
5.5 Exposure, Dosing, and Compliance 32
5.6 Sample Description 32
5.7 Course of the Study 33
6 Listings of Individual Data 33
7 Overview of Assessments 35

ABBREVIATIONS

AE	Adverse Event
AESI	Adverse Event of Special Interest
AHI	Apnoea Hypopnoea Index
ALAT/SGPT	Alanine Aminotransferase
ANCOVA	Analysis of Covariance Test
ASAT/SGOT	Aspartate Aminotransferase
BDI-13	Beck Depression Inventory-13 items
BMI	Body Mass Index
BUN	Blood Urea Nitrogen
CI	Confidence Interval
CPK	Creatine Phosphokinase
CRF	Case Report Form
ECG	Electrocardiogram
EDS	Excessive Daytime Sleepiness
ES	Effect Size
ESS	Epworth Sleepiness Scale
FAS	Full Analysis Set
GCP	Good Clinical Practice
GGT	Gamma-glutamyltranspeptidase
ß-hCG	Human Chorionic Gonadotropin
INR	International Normalized Ratio
LSEQ	Leeds Sleep Evaluation Questionnaire
MCV	Mean Corpuscular Volume
MedDRA	Medical Dictionary for Regulatory Activities
MMSE	Mini Mental State Examination
nCPAP	nasal Continuous Positive Airway Pressure
OD	Once a day
OSA	Obstructive Sleep Apnoea
OSleR	Oxford Sleep Resistance Test
PGOE	Patient’s Global Opinion of the Effect
PP	Per Protocol
SAE	Serious Adverse Event
SAF	Safety Population
SAP	Statistical Analysis Plan
SaO_2	Oxygen Saturation
SD	Standard Deviation
TEAE	Treatment-Emergent Adverse Event
TEAESI	Treatment-Emergent Adverse Event of Special Interest
TFLs	TABLEs, Figures, and Listings
TMT	Trail Making Test
TSP	Total Sleep Period
TST	Total Sleep Time
WHO	World Health Organization

1 Introduction

This statistical analysis plan (SAP) is based on the last version of the protocol of the study, being Version 1.0, dated 2 December 2015 and of the CRF Version 1.2, dated 16 February 2016.

The purpose of this SAP for the double-blind part of the study is to provide details of the statistical analysis, and to ensure that the statistical methodologies that will be used, and the summary tables, figures, and listings (TFLs), are in line with standard practice, and complete and appropriate to reach valid conclusions regarding the study objectives.

1.1 Study Design

This is a prospective, multicenter (several centers in Bulgaria), randomized, phase III study of pitolisant given at 10, 20, or 40 mg per day versus placebo.

It is carried out in patients diagnosed with Obstructive Sleep Apnoea (OSA) syndrome, without important cardiovascular disease, experiencing Excessive Daytime Sleepiness (EDS) with Epworth Sleepiness Scale (ESS) score ≥ 12, having refused to be treated by nasal Continuous Positive Airway Pressure (nCPAP) or having been submitted to nCPAP therapy for a minimum period of 3 months, and still complaining of EDS.

The study consists of two parts, a 12-week double-blind part starting with an escalating dose period followed by treatment with the selected dose. Then, after one week of single-blind placebo wash-out period, if the patient holds the same position towards nCPAP therapy as before, an Open Label Extension period is proposed.

Patients who do not participate in the Open Label Extension period will have their end of the study visit. Patients willing to continue the pitolisant treatment administration will be given another information notice together with an informed consent form to be signed. This Open Label Extension period will consist of the same escalating-dose regimen as in the 1st part of the study, followed by a selected dose period with the active drug only, until 52 weeks after the treatment beginning. Then, patients will have a one week wash-out period prior to the end of study visit.

Seven visits and two phone calls were planned in the double-blind part of the study:

- V1 = Screening visit and beginning of initial wash-out (Day -14)
- PC1 = Phone contact n°1 (D-7)
- V2 = Inclusion visit and beginning of the double-blind period (D0)
- V3 = Visit 3 (D14)
- V4 = Visit 4 (D21)
- V5 = Visit 5 (D49)
- V6 = Visit 6 – End of double-blind – Evaluation visit (D84)
- PC2 = Phone contact n°2 (V6 + 3 days)
- V7 = Visit 7 – Final visit (D91)

The date of each visit could be +/- 3 days.

An overview of the visits and assessments is given in Section 6.

Patients submitted to nCPAP therapy should be treated for at least 4 hours per day. The compliance is checked on the clock-time counter of the CPAP machine. These patients should have an Apnea-Hypopnea Index (AHI) ≤ 10. Patients without nCPAP therapy should have AHI ≥ 15.

Polysomnography is to be performed (for patients submitted to nCPAP therapy – under nCPAP) between V1 and V2 or during the last 12 months.

With the exception of spending an optional one night (if not done during the twelve months preceding the study) in the sleep laboratory for the full recording of nocturnal polysomnography between V1 and V2, the patient are to be ambulatory during the whole study period.

1.2 Study Treatment

The study was to screen approximately 400 patients so that 360 patients could be analyzed. These patients were to be divided into 4 groups:

- 60 patients with OSA complaining of EDS refusing the nCPAP therapy will be administered pitolisant active ingredient product;
- 30 patients with OSA complaining of EDS refusing the nCPAP therapy will be administered a placebo;
- 60 patients with OSA treated by nCPAP but still complaining of EDS will be administered pitolisant active ingredient product;
- 30 patients with OSA treated by nCPAP but still complaining of EDS will be administered a placebo.

Pitolisant and placebo are presented in identical tablets according to dosage, i.e. pitolisant tablets dosed at 5 mg or 20 mg and matching placebo.

During the double-blind phase patients were to take daily 10 mg (2 tablets of 5 mg) or 20 mg (1 tablet of 20 mg) or 40 mg (2 tablets of 20 mg) of pitolisant or matching placebo. During the open label extension period patients were to take daily 10 mg, 20 mg or 40 mg of pitolisant (i.e. 2 tablets of 5 mg or 1 or 2 tablets of 20 mg, respectively).

Study medication was to be administered orally, once a day (OD), in the morning, during breakfast, with a glass of water.

1.3 Objectives

The first objective of this study is to demonstrate the efficacy and safety of pitolisant given at 10, 20, or 40 mg/day versus placebo, during the 12 weeks of the double-blind period, for the treatment of EDS in patients with OSA refusing nCPAP therapy or treated with nCPAP but still complaining of EDS.

The secondary objectives include assessing the long-term tolerance, as well as the maintenance of efficacy of pitolisant given at 10, 20, or 40 mg per day during 39 weeks of the open label extension period and further investigating the co-variates or co-medications that affect the pharmacokinetics of pitolisant in the target population to allow future comparison to healthy subjects.

1.4 Sample Size Justification

Results from exploratory studies on pitolisant allow to estimate the ESS residual variability to standard deviation (SD) = 6. The Minimum Important Difference (MID) was fixed to ESS = 3, corresponding to an effect size (ES) = 0.5. The correlation between final and baseline ESS was conservatively estimated as r = 0.3.

a) By assuming ANCOVA at 0.95 confidence level as the main confirmatory test, a difference of at least Delta = 3 should be detected with a power of 90% in using at least 30 patients in each placebo group (60 in total) and 60 patients in each pitolisant treatment group (120 in total).

b) By assuming the same model, an interaction of at least 3 between CPAP and non-CPAP will be detected with a power of 90% when 120 and 240 patients (thus 360 patients in total) are treated in Placebo and pitolisant group respectively.

Treatment groups will be stratified by center and CPAP use. Considering 10% drop out rate, 400 patients will be selected.

The main endpoint remains the efficacy of the IMP compared with placebo on ESS out of CPAP consideration, but this sample size provides enough power to assess the interaction of the IMP with CPAP.

2 Data Considered for the Analysis

2.1 Data Only Recorded at Onset of the Study

- Center number and patient number
- Date of double-blind period consent
- Date of screening visit
- Age (year)
  Age group is calculated considering the following categories:
  < 35 years, ≥ 35 and < 50 years, ≥ 50 and < 65 years, ≥ 65 years
- Sex
- For women, childbearing potential (yes, no), and if yes, contraceptive methods
- Professional activity (yes, no), and if yes, nature, number of hours/24h, number of days/week, shift worker/professional driver (yes, no)
- Patient under nCPAP (yes, no): recorded at V1 and V2
- Relevant medical and surgical history; Any history (no, yes), and if yes, for each of 26 system organ classes and others:
  - Status (no, yes)
  - Date start and date end or present at study start (no, yes)
  - Specification
  - Prevents inclusion (yes, no)
- Obstructive Sleep Apnea (OSA) and Excessive Daytime Sleepiness History:
  - Time since diagnosis (month) calculated as:
    12 x (date of screening visit–date of diagnosis) / 365.25
    In case the day of diagnosis is missing and the month and year are available day is set to 1.
    In case the day and month of diagnosis are missing, time since diagnosis is calculated as:
    12 x (year of screening visit–year of diagnosis)
  - Apnea-hypopnea index (AHI)
  - If under nCPAP:
    - Time since residual sleepiness on nCPAP (OSA nCPAP beginning) (month) calculated as:
      12 x (date of screening visit–date of OSA nCPAP beginning) / 365/25

In case the day of OSA nCPAP beginning is missing and the month and year are available the day is set to 1. In case the day and month of OSA nCPAP beginning are missing, time since residual sleepiness on nCPAP is calculated as:
12 x (year of screening visit–year of OSA nCPAP beginning)

- nCPAP pressure (cm H_2O)
- Mean nocturnal oxygen saturation (SaO_2) (%)

- Sleep:
  - Number of daytime sleep and sleepiness episodes
  - Duration of daytime sleep and sleepiness episodes (h)
  - Number of nocturnal awakening episodes
  - Duration of nocturnal awakening episodes (h)
- Durations are calculated as hours + (minutes/60).

Mini-Mental State Examination (MMSE):

- Polysomnography (V2):
  - Total sleep time [TST] (min), total sleep period [TSP] (min)
  - For sleep latency, wake after sleep onset, Stage 1, Stage 2, Stage 3, and REM sleep: Duration (min) and % of TST (calculated if duration and TST are available)
  - Duration of TST < 90 (O_2 saturation) (min), mean nocturnal SaO_2 (%), Apnea-Hypopnea Index (AHI), number of awakenings
  - Respiratory Micro Arousal Index, Non-respiratory Micro Arousal Index, Index of periodic limb movements
  - Leak of CPAP mask (for patient under nCPAP): No leak (yes, no), minor leak (yes, no), major leak (yes, no). The following variable is calculated based on these data:
    - No leak
    - Minor only
    - Major only
    - Major and Minor
    - Unspecified

2.2 Efficacy Data

- Epworth Sleepiness Scale (ESS) (V1 to V7):
  - Scores for each item (0 = never or no likelihood or falling asleep, 1 = rarely falling asleep or slight likelihood of falling asleep, 2 = Often falling asleep or moderate likelihood of falling asleep, 3 = high probability of falling asleep) and total score
  - The final total score is defined as the average of the non-missing values at V5 and V6. If both values are missing two definitions are used for estimating the final score:
    DBF-LOCF ESS (last observation carried forward) = last available ESS at V2, V3, and V4
    DBF-BOCF ESS (baseline observation carried forward) = ESS at V2

  - The primary efficacy variable is (DBF-LOCF ESS – ESS at V2)
  - Additional efficacy variables based on the ESS are:
    - DBF-BOCF ESS – ESS at V2
    - 100 x (DBF-LOCF ESS – ESS at V2) / ESS at V2
    - 100 x (DBF-BOCF ESS – ESS at V2) / ESS at V2
    - Response definition 1 (R_1): DBF-LOCF ESS ≤ 10 (yes, no)
    - Response definition 2 (R_2):
      [(DBF-LOCF ESS ≤ 10) or (DBF-LOCF ESS ≤ ESS at V2 - 3)] (yes, no)
- Sleep Diary; For 3 days:
  - SDR1: At what time did you get up this morning?
  - SDR2: How many sleep/sleepiness episodes did you experience today?
  - SDR3: At what time did you go to sleep last night?
  - SDR4: What was the total duration of your sleep/sleepiness episodes today?

  The following variables are calculated based on the available data for each period (V1-V2, V2-V3, V3-V4, V4-V5, V5-V6, V6-V7):
  - Wakefulness duration for Day i = SDR3 of Day i+1 - SDR1 of Day i
  - SDWD = Mean daily wakefulness duration = mean wakefulness duration for Days 1 and 2
  - Alertness duration for Day i = SDR3 of Day i+1 - SDR1 of Day i – SDR4 of Day i
  - SDAD = Mean daily alertness duration = mean alertness duration for Days 1 and 2
  - SDNS = Mean daily number of sleep/sleepiness episodes = mean SDR2 for Days 1, 2, and 3
  - SDDS = Mean daily duration of sleep/sleepiness episodes = mean SDR4 for Days 1, 2, and 3
- Oxford Sleep Resistance Test (OSleR) (V2, V6):
  - OSL = Mean sleep latency (mean of the 3 tests conducted at three times of the day). Higher values indicate better performance. In order to be able to calculate geometric means, values of 0 for mean sleep latency are replaced by 10 seconds (half the minimal possible positive value).
  - OSLC (yes, no):
    Success (OSLC='yes') defined as number of 3-6 and ≥7 errors = 0 for each of the 3 tests.
    In case any of the variables is missing OSLC is considered to be missing.
- Aggregate z-score of ESS and OSleR (V2, V6):
  - ESSOSZ = Z(ESS) + Z(log(OSL)
    using the mean and SD of ESS and log(OSL) at V2 of the Full Analysis Set (FAS).
- EQ-5D Health Questionnaire (V2, V6):
  - Descriptive system (5 items scored as 1, 2, or 3);
    EQ5DS = sum of the 5 non-missing items.
  - Regrouped items:
    1 (no problems) is transformed to 0
    2 (some problems) and 3 (extreme problems) are transformed to 1

  - EQ5DVAS = Visual Analog Scale (VAS):
    (0 = Worst imaginable health state, 100 = Best imaginable health state)
  - EQ5DZ = Z(EQ5DS) + Z(EQ5DVAS)

- Leeds Sleep Evaluation Questionnaire (LSEQ) (V2, V6):
  - Scores for items 1 to 10

  The following scales are used in the analysis:
  - GTS (getting to sleep) = Mean of items 1, 2, and 3
  - QOS (quality of sleep) = Mean of items 4 and 5
  - AFS (awake following sleep) = Mean of items 6 and 7
  - BFW (behavior following wakening) = Mean of items 8, 9, and 10

  In case one item of a scale is missing the average is calculated of the remaining items. In case more than one is missing the scale is considered missing.
- Trail Making Test (TMT) (V2, V6):
  - TMTA (sec)
  - TMTA deficiency: TMTA > 78 sec
  - TMTB (sec)
  - TMTB deficiency: TMTB > 273 sec
- Clinical Global Impression of Severity (CGI-S) regarding excessive daytime sleepiness (V1, V2):
  - CGI-S assessed as: 1=normal (not at all ill), 2=borderline ill, 3=mildly ill, 4=moderately ill, 5=markedly ill, 6=severely ill, 7=among the most extremely ill patients

- Clinical Global Impression of Improvement (CGI-C) regarding excessive daytime sleepiness symptoms (V6, V7):
  - CGI-C assessed as: 1=very much improved, 2=much improved, 3=minimally improved, 4=no change, 5=minimally worse, 6=much worse, 7=very much worse
  - CGI-C improvement:
    - Yes = Very much improved, much improved, or minimally improved
    - No = No change, minimally worse, much worse, or very much worse
- Patient's Global Opinion of the Effect (PGOE) of the Investigational Drug compared to pre-study condition (V6, Phone contact 2, V7):
  - PGOE assessed as: Marked effect, moderate effect, minimal effect, no change, minimally worse, much worse
  - PGOE improvement:
    - Yes = Marked effect, moderate effect, or minimal effect
    - No = No change, minimally worse, or much worse
- Pichot Fatigue Scale (V2, V6, V7):
  - Pichot Fatigue Scale Score

- Activity (Phone contact 1, V2 to V7):
  - Did the patient maintain a stable activity since the last visit (yes, no)

2.3 Safety Data

- Adverse events (AEs); The following data are recorded for each event:
  - Description
  - Visit number when AE was reported
  - Dose of study drug when AE occurred (10 mg, 20 mg, 40 mg, NA)
  - Date of onset or worsening and end date or ongoing
  - Intensity (mild, moderate, severe)
  - Frequency (once, intermittent and number and period, continuous, unknown)
  - Action taken with studied drug (none, dose modification, temporary interruption, discontinued)
  - Action taken with event (none, corrective treatment, additional exploration, hospitalization ≤ 24 hr, hospitalization > 24 hr)
  - Serious adverse event (SAE) (yes, no)
  - Outcome (recovered, recovered with sequelae, worsened, not yet recovered, death)
  - Imputability (likely related, possibly related, unlikely related)
  - Etiology (studied drug, concomitant treatment, associated disease, other, unknown)

  Adverse events are coded using the Medical Dictionary for Regulatory Activities (MedDRA). All adverse events are attributed a MedDRA Preferred Term (PT) and the primary System Organ Class (SOC).

  Treatment-emergent adverse events (TEAEs) are events which start on or after the first intake of study medication (or whose intensity worsened on or after that time). For patients consenting to continue in the open label phase, events which start at least one day after Visit 7 are attributed to the open label phase.

  In case of a partial or missing date for the start of an adverse event it will be assumed that the event was treatment-emergent unless it can be determined from the partial start or stop date that the event definitely started before the first study medication.

  An event is considered to be related to the study treatment if the assessment of imputability is likely related or possibly related, or if the assessment is missing.

  In the Case Report Form (CRF) adverse events occurring with different intensity, frequency, seriousness, or action taken could be documented as separate episodes. For the analysis:
  - In case the first episode started on or after the first intake of study medication or the last episode stopped before the first intake of study medication, the episodes will be collapsed into a single event. While collapsing episodes, the highest seriousness, worst intensity and all actions taken with respect to study medication will be retained. The event will be counted as a single event.
  - In case the adverse event started before the first intake of study medication and continued till after the first intake of study medication, and:

    - The severity did not increase on or after the first intake of study medication the episodes will be collapsed into a single adverse event.
    - The severity increased on or after the first intake of study medication, the episodes from the first increase on, on or after the first intake of study medication will be collapsed into a single adverse event and the episodes before the first intake of study medication will be collapsed into another single adverse event.

  When listing the data, the individual episodes will be presented.

- Adverse events of special interest (AESIs); As per the Risk Management Plan for Pitolisant [Wakix (Pitolisant) EMEA_H_C_2616 PRAC Rapp Updated RMP update AR 2017-05-26.doc], AESIs are:
  - Anxiety
  - Depression
  - Drug abuse and misuse
  - Drug dependence
  - Fertility disorders
  - Gastric disorders caused by hyperactivity
  - Insomnia
  - Proconvulsive potential
  - QT-interval prolongation
  - Rebound effect
  - Weight increase

  The MedDRA PT of the AESIs will be identified by Bioproject in the database. If pertinent MedDRA PTs may be combined for the analysis.

- Safety Laboratory Data (V1, V6); Value, unit, status (normal, abnormal not clinically significant, abnormal clinically significant) for each of the following data:
  - Hematology: Red blood cells, hemoglobin, hematocrit, mean corpuscular volume (MCV), white blood cells, 5 differential (neutrophils, lymphocytes, monocytes, eosinophils, basophils) or 3 differential (granulocytes, lymphocytes, monocytes), platelets, coagulation time INR
  - Biochemistry: Blood urea nitrogen (BUN), uric acid, creatinine, creatine phosphokinase (CPK), ALAT/SGPT, ASAT/SGOT, GGT, alkaline phosphatase, total protein, total bilirubin, glucose, total cholesterol, triglycerides
  - Electrolytes: Sodium, potassium, calcium, chloride, bicarbonates/CO_2
  - ß-HCG serum pregnancy test assessed as positive or negative
  - Urine analysis: Dipstick test assessed as positive or negative (only V1)
  - HBsAg, HCV, HIV assessed as positive or negative (only V1)
- Vital signs (V1 to V7):
  - Height (cm) (only at V1)
  - Weight (kg) and body mass index (BMI) (kg/m^2)
  - Systolic and diastolic blood pressure (mm Hg)

  - Heart rate (bpm)
- Physical Examination (V1 to V7); Status (normal, abnormal, not done), and significant abnormalities for each of the following body systems:
  - General status
  - Cardiovascular
  - Respiratory
  - Abdominal
  - Neurology
  - Locomotor system
  - Dermatology
  - Other

  At V2 to V7 any change since the previous visit was also recorded.
- Electrocardiogram (ECG) (V1 to V7):
  - Heart rate (bpm)
  - Sinusal rhythm (no, yes)
  - PR (msec)
  - QRS (msec)
  - QT (msec)
  - QTcF (msec) (QT x $(HR/60)^{1/3}$)
  - QTcB (msec) calculated as QT x $(HR/60)^{1/2}$
  - Result (normal, abnormal)

  Baseline is defined as the average of the non-missing data at V1 and V2.

  Stable treatment value is defined as the average of the non-missing data at V4, V5, and V6.
  - Additional variables based on QT and QTcF:
    - QT500: Any occurrence of QT post-dose (V3 to V7) > 500 msec (yes, no)
    - QTcF450: Any occurrence of QTcF post-dose (V3 to V7) > 450 msec (yes, no)
    - ΔQTcF ≥ 60 msec (yes, no), with
      ΔQTcF = Max [QTcF post-dose (V3 to V7)] – Min [QTcF pre-dose (V1, V2)]
- Beck Depression Inventory (BDI) (V1, V2, V6, V7):
  - 13 items score
  - 13 items score category: 0-4, 5-7, 8-15, ≥ 16
  - Item G
- Amphetamine-like withdrawal symptoms (Phone contact 2, V7) (yes, no) for each of the following symptoms:
  - Dysphoria
  - Fatigue
  - Vivid and unpleasant dreams

  - Insomnia or hypersomnia
  - Increased appetite
  - Psychomotor retardation or agitation

  Amphetamine-like withdrawal syndrome is defined as dysphoria and 2 or more of the other symptoms. In case the assessment of dysphoria is missing or the assessment of more than one of the other symptoms are missing amphetamine-like withdrawal syndrome is considered missing.

- Patient's Overall Evaluation of Tolerance (V3 for 1st and 2nd week, V4, V5, V6, V7) (good, moderate, poor)
- Prior medications and concomitant medications recorded throughout the study:
  - Generic name
  - Galenics
  - Dose and unit
  - Frequency
  - Route
  - Reason for use (medical history diagnosis, AE diagnosis, other reasons)
  - Date beginning and date end
  - Ongoing at screening visit and at end of study (yes, no)

  Medications will be coded according to the World Health Organization (WHO) drug dictionary, at the level of the Anatomical Therapeutic Chemical (ATC) coding and preferred drug name, taking indication and route into account, if available.

  Concomitant medications are defined as medications ongoing at or started on or after the first intake of study medication. For patients consenting to continue in the open label phase, medications that start on or after Visit 7 are attributed to the open label phase.

2.4 Course of the Study

The following end of study data are collected for all patients:

- Date of first treatment intake
- Date of last treatment intake
- Maximal dose administered
- End of study date
- Did the patient complete the study (yes, no) and if not, reasons and specification:
  - Non-eligible patient
  - Discontinuation by the investigator
  - Adverse event
  - Double-blind broken
  - Lost to follow-up and dates of first call, second call, and last letter sent by the investigator
  - Severe depression (BDI-13 ≥ 16 or item G > 0)
  - ECG Fridericia corrected QT interval > 450 msec
  - Voluntary withdrawal of patient consent

  - Major protocol deviation or non-compliance
  - Prohibited treatment intake
  - Patient changing position towards nCPAP therapy
  - Other reason

Based on these data and on study dates the following variables are calculated:

- Date of end of double-blind phase:
  - For patients consenting to continue in the open label phase:
    The last of the dates of (V1, Phone contact 1, V2, V3, V4, V5, V6, Phone contact 2, V7)
  - For patients stopping after the double-blind phase:
    The last of the dates of (V1, Phone contact 1, V2, V3, V4, V5, V6, Phone contact 2, V7, last treatment intake)
- Double-blind phase duration = Date of end of double-blind phase – Date of V1

2.5 Study Treatment and Compliance

The following data are recorded:

- V3:
  - Real number of returned unused tablets for Weeks 1 and 2
  - Dose prescribed for Week 3 (mg) (low=10 mg, medium=20 mg, high=40 mg)
- V4:
  - Real number of returned unused tablets for Week 3
  - Dose prescribed for Weeks 4 to 7 (low, medium, high)
- V5
  - Real number of returned unused tablets for Weeks 4, 5, 6, and 7
  - Dose prescribed for Weeks 8 to 12 (low, medium, high)
- V6
  - Real number of returned unused tablets for Weeks 8, 9, 10, 11, and 12
  - Dose prescribed for Week 13 (low, medium, high)
- V7
  - Real number of returned unused tablets for Week 13

Based on these data and the end of study data the following variables are calculated:

- Maximum prescribed dose during the double-blind phase = Maximum (20 mg, dose prescribed at V3, V4, and V5)
- Stable dose = Dose prescribed at V5

- Compliance between V2 and V3 (W1, W2):
  - Number of tablets prescribed:
    20 tablets of 5 mg for Week 1 and 10 tablets of 20 mg for Week 2

- Number of tablets taken:
  (20 – number of tablets of 5 mg returned) for Week 1
  (10 – number of tablets of 20 mg returned) for Week 2
- Dose taken:
  Number of tablets taken Week 1 x 5 mg + Number of tablets taken Week 2 x 20 mg
- Dose to be taken:
  - If Date of V3 – Date of V2 > 7:
    10 mg x 7 + [(Date of V3 – Date of V2) – 7] x 20 mg
  - If Date of V3 – Date of V2 <= 7:
    (Date of V3 – Date of V2) x 10 mg
- Compliance (%) = 100 x Dose taken / Dose to be taken

- Compliance during subsequent periods:
  V3-V4 (W3), V4-V5 (W4 to W7), V5-V6 (W8 to W12):
  - Number of tablets prescribed:
    If 10 mg/d was prescribed: 20 tablets of 5 mg x number of weeks
    If 20 mg/d was prescribed: 10 tablets of 20 mg x number of weeks
    If 40 mg/d was prescribed: 20 tablets of 20 mg x number of weeks
  - Number of tablets taken = Number of tablets prescribed – sum of numbers of tablets returned
  - Number of daily doses taken:
    If 10 mg/d was prescribed: Number of tablets taken/2
    If 20 mg/d was prescribed: Number of tablets taken
    If 40 mg/d was prescribed: Number of tablets taken/2
  - Number of daily doses to be taken = Date of next visit – date of visit
  - Compliance (%) = 100 x number of daily doses taken / number of daily doses to be taken
- Overall compliance =
  100 x total number of daily doses taken / total number of daily doses to be taken
  Where:
  Total number of daily doses taken = sum of number of daily doses taken in each period
  Total number of daily doses to be taken = sum of number of daily doses to be taken in each period
  NOTE: For patients who did not complete the double-blind phase of the study, overall compliance is calculated with the total number of daily doses taken and the total number of daily doses to be taken until the last visit before study interruption.
- Exposure to double-blind treatment (day):
  - For patients stopping during or after the double-blind phase:
    Date of last treatment intake – Date of first treatment intake + 1
  - For patients continuing in the open label phase:
    Date of V7 – Date of first treatment intake + 1

3 Statistical Methodology

3.1 Statistical Software

The SAS Version 9.2 statistical software package will be used for all statistical analyses.

3.2 Analysis Sets

The following analysis sets are considered:

Full Analysis Set (FAS): All randomized patients. The statistical analyses are based on the treatment to which the patient was randomized.

Safety Population (SAF): All patients who received at least one dose of study medication, and for whom at least one valid post-baseline evaluation (including any AE) is available. The statistical analyses are based on the treatment delivered to the patient at V2.

Per Protocol (PP) Population: All patients of the FAS without any major protocol deviation. This analysis will not be performed, when the PP sample size is at least 95% of the FAS sample size.

The analysis sets will be determined by a blinded review of the data prior to database lock.

The analysis on the FAS will be considered the primary analysis. The analysis of the demographic data and other baseline characteristics will be performed on the FAS. The efficacy analysis will be performed on the FAS and the PP Population for main and secondary endpoints.

The analysis of safety, concomitant medications, exposure, dosing, and compliance will be performed for the Safety Population.

GENERAL NOTE: Some of the efficacy variables (OSleR test, TMT, LSEQ, EQ5D) were only recorded at visit V7 for patients entering the open label part of the study. For this reason, all observations at V7 are not taken into account in the analyses of these variables.

3.3 Protocol Deviations

In this trial, a protocol deviation was assessed to be major if it could have a direct or indirect effect on the primary outcome. The final decision as to whether a deviation was considered major or minor was taken during the Blind Review meeting.

3.4 Baseline

Baseline is defined as Visit 2.

3.5 Missing Data

Last Observation Carried Forward (LOCF) was the planned MDI in the original protocol and previous trials of Pitolisant in Narcolepsy, the justification being the short length of the study allowing the LOCF approximation. Baseline Observation Carried Forward (BOCF) constitutes another relevant alternative. For patients early terminating the trial for a reason suspected with the treatment (as for instance, drug related AE, decision to stop for perception of lack of efficacy, pathology aggravation, ..), treatment failure will be concluded in imputing BOCF. For intercurrent reasons unrelated with the treatment, the LOCF imputation will be used. The allocation of treatment related/unrelated will be attributed during the Blind Data review Meeting.

For the main endpoint, Multiple Imputation[22] (MI) will be used as a supportive technique in this analysis. As MI ideally assumes Missing At Random (MAR) data, we will use the imputation MI-REL in which missing data will be imputed according their baseline value, out of treatment effect, with general model-1 used as the imputation model . This result is expected as conservative but allows non-Missing data at Random nMAR in relying to worst case placebo effect. To assume enough stability to the generated data files, we fixed the number of burn-in iterations to reach stationarity to NB=5000, the number of data sets to K=10, and the number of iterations to 100.

The following statistics will be provided: Knowing calculation of VB (Variance between imputed datasets: SD of the estimates across the imputed Data sets), VW (SD mean expected on non-missing data), VT (Total Variance VT = VB + VW + VB/m), Infinite DF: Degrees of Freedom (DF) as assumed by Barnard and Rubin[23]: RV or Relative Increases in Variance increase in total sampling variance that is due to missing information ([VB + VB/m]/VW), and FMI or Fraction of Missing Information: Proportion of the total variance due to missing data ([VB+ VB/m]/VT).

3.6 Summary Statistics

Unless specified otherwise, the following summary statistics will be used to describe the data:
For continuous variables: including the mean, 95% confidence interval (CI) on the mean, standard deviation (SD), minimum, 1st quartile, median, 3rd quartile, maximum, number of available and number of missing observations.
For Binary and categorical variables: number and percentage for each of the scores or categories, and the number of observations.

Descriptive statistics of onset data will be given:

- For all patients
- Broken down by treatment group
- Broken down by use of nCPAP
- Broken down by use of nCPAP and treatment group.

Descriptive statistics of efficacy and safety data will be given:

- Broken down by treatment group
- Broken down by use of nCPAP and treatment group.

For the primary efficacy variable the descriptions will also be given broken down by center.

For selected variables descriptive statistics will also be given for the change from baseline (visit minus baseline).

3.7 Inferential Aspects

The significance of the IMP compared with control on efficacy outcomes will be assessed according to a statistical model of covariance (Model-1) featured by a Generalized Mixed Model where the studied outcome at final time constitutes the dependent variable, and main terms being treatment as a fixed binary factor by adjusting for the baseline value of the studied outcome (fixed covariate) , use of CPAP (fixed factor), and center (random factor). A supportive model will include the interaction CPAP*Treatment (Model-2, see details in section 4.3). Model-1 will be used in safety analyses in adding age and gender as additional adjustment covariates. All statistical tests will be performed at the two-sided 95% confidence level.

All the endpoints referred in detail in the following section will be analyzed according the same statistical model allowing a comparison of results between the studied endpoints. Unless otherwise specified for each particular endpoint, the significance of the difference between the IMP compared with placebo will be assessed through a general model of analysis of Covariance (ANCOVA) in which the studied endpoint at final time (noted Y_f) constitutes the dependent variable of the model. Independent variables are (1) the baseline value of the studied endpoint (noted Y_b) analyzed as a fixed covariate, (2) the treatment effect (Noted Trt) coded 0 (placebo) or 1 (IMP) (cell mean contrast formulation), and (3) the center considered as a random factor of the intercept constituted by a fixed part k_0 and a random component $N(0,\sigma_c)$. This model is summarized by the following expression:

General Model 1:

$$Y_{if} = (k_0+\zeta) + k_b Y_b + k_t \, Trt + \epsilon_i \quad \epsilon_i = N(0,\sigma) \text{ and } \zeta = N(0,\sigma_c)$$

Because both CPAP and non-CPAP patients are included in this trial, we assume that using CPAP since baseline may impact the final value. We use a first supportive model-2 hypothesizing a main effect of CPAP use (0,1) from baseline and described as follows :

General Model 2:

$$Y_{if} = (k_0+\zeta) + k_b Y_b + k_t \, Trt + k_c \, CPAP + \epsilon_i \quad \epsilon_i = N(0,\sigma) \text{ and } \zeta = N(0,\sigma_c)$$

CPAP use may have both a main effect but also some interaction effect with treatment, meaning that the effect of treatment might be different for patients using or not CPAP. A model-3 admits a new term of interaction K_i CPAP*Trt :

General Model 3:

$$Y_{if} = (k_0+\zeta) + k_b Y_b + k_t \, Trt + k_c \, CPAP + K_i \, CPAP*Trt + \epsilon_i \quad \epsilon_i = N(0,\sigma) \text{ and } \zeta = N(0,\sigma_c)$$

The main analysis is defined as Model 1 with LOCF/BOCF Missing imputation on FAS sample (see section 2.4). All the other analyses are conducted for sensitivity purposes. For the main and secondary endpoints (ESS, OSLER, CGI, FATIGUE), the three models will be fitted in using both MD imputations (LBCF, MI). The three models will be compared: (a) By denoting L the likelihood, n the number of parameters in the model and N the sample size the Bayesian Information criteria is BIC= -2L+n*log(N). (b) The significance of change in the residual sum of squares (RSS) defined as follows: Let be GM the general model and NM the nested model within GM. The difference ΔRSS= RSS(NM)-RSS(GM) is always ≥0 and the ratio (ΔRSS/q)/RSS(GM)/(N-k) ~ F(q,N-k) in the hypothesis where ΔRSS=0. The summary of inferential models will be provided by the following table, with for each model the effect estimate and 95%CI (X [XL,XU] and corresponding P value, BIC and P value of the change of residual square.

Finally, unless otherwise specified, the significance test will be conducted at a two-sided significance level of .05.

3.7.1 Notes

1-The main model-1 does not assume a treatment-baseline interaction term, as required (CPMP, 2003). Our decision of using center as a random factor is justified by the useless estimate of each center, in focusing on the measurement of the standard deviation of the intercept. The treatment was considered as a fixed factor without center random component.

2- This model is a Linear Mixed Model (LMM) as it combines random and fixed factors, assumes linearity of baseline and hypothesizes the studied endpoint distributed according to a normal (Gaussian) distribution. General Model-1 constitutes the main model used in this trial for all the studied endpoints unless otherwise specified.

3-When the studied endpoint is binary, the above model will be implemented by a Generalized Linear Mixed Model GLMM optimizing the likelihood ratio to a binomial distribution featuring a logistic regression. Similarly, when the studied endpoint is a count, GLMM will be used based on a Poisson distribution. In case where the overdispersion is such that $\Phi > 2$, the so-called quasi-Poisson correction will be applied in correcting the standard error by a variance $\sigma^2 = \Phi\mu$ (μ being the observed mean).

3.7.2 Reporting format

The following is a hypothetical example of a mixed model featuring Analysis of Covariance and model of presentation. It includes model coefficients, 95% Confidence interval and P-values for the fixed covariates and the SD of the intercept across centers. To be clinically interpretable, the intercept will be based on the mean Baseline value (in centering baseline for the analysis). Computational details in appendix will be referenced in the legend.

3.8 Accounting for Multiple Centers

Treatment was dispensed to be balanced between centers, however, the randomization block size was 4, so that an asymmetric distribution is possible, particularly within small centers. To account for center variability and possible unbalance, center will be used as an adjustment factor. In the main analysis, random will be assimilated to a random factor adding to the fixed intercept, to account for and estimate the expected variability of the mean outcome value across centers. For sensitivity purposes, the analysis will be repeated in assuming center as a fixed factor.

3.9 Outcomes and Multiple Testing

Our main analysis was determined as the main endpoint (ESS) conducted on an Intent to treat Basis (FAS selection), through the mixed model precisely defined by the pre-specified covariates (see section …) and LOCF/BOCF missing data imputation technique. The secondary endpoints will be the OSLER test, and Clinical Global Impression. The other endpoints will be considered as exploratory endpoints.

Controlling the multiplicity of test will be performed according the fixed sequence [ref : FDA multiplicity of tests]. The main endpoint will be evaluated first, then, the OLSER test will be tested conditionally to the significance of the main endpoint, finally, the CGI will be tested conditionally to the OSLER test significance. The fixed sequence limits the familywise risk to a maximum type 1 error of .05.

3.10 Intermediate Analyses

A one-stage Futility stopping was based on Conditional Power, the probability to detect a significant result at the end of the double-blind phase, given the results observed at an intermediate time. Conditional Power was estimated (Lan and Wittes 1988; Lan and Zucker 1993). This analysis was carried out by a third party statistician when at least 80 patients were available, with futility threshold CPmin=.10 involving a slight increase of type 2 error (Proschan 1999)

3.11 Changes from the Analyses Foreseen in the Protocol

1.The protocol foresaw to consider as baseline for ESS and ECG data the mean of the values recorded at V1 and V2. Since it was considered that the data was likely to change between V1 and V2 because of the wash out period in between, it was decided to consider V2 as baseline for all endpoints.

2.To be consistent with the analysis of previous studies it was decided also to perform an analysis of the EQ-5D data based on a composite z-score of the descriptive system and VAS.

3.Due to possible sensitivity of results based on either the fixed or random assumption of center, Adjusting for the center fixed effect was added and compared with results based on the random center effect .

4.Test Multiplicity : The precise technique of control of type 1 is described in section 3.6.

5.Missing data imputation techniques : The two used techniques are described in details in section 3.4.

6.Alternative Inferential model to assess the effect of CPAP and possible interaction: see section 2.3.1

7.Osler : Because data are truncated at 40 minutes, we added a survival technique to assess the significance of the studied treatment on time to somnolence.

4 Contents Description

In the following sections, all the mentioned variables will be reported in TFL following a two way table (Treatment, CPAP). In what follows, only summary tables are requested. These tables will refer to corresponding detailed tables in TFL.

4.1 Sample Description

The number of patients will be provided in each of the analysis sets, in each center, and in each of the analysis sets at each visit. In addition, the date of first and last visits will be provided for each center and treatment.

A documented list will be given of patients excluded from the analysis sets and of protocol deviations. The analysis of protocol deviations will be performed on the basis of the FAS and consist of: Frequency distributions of patients having at least one major deviation, at least one major deviation by type of deviation, at least one deviation, and at least one deviation by type of deviation

Based on the FAS, two-way table reporting the number of patients having at least one major deviation with row= type of major deviations and treatment as column. In the last row of this table, Number of minor deviations(NmD) by Treatment.

Baseline data will report :

<u>Demographic</u> Data (Age (year), Age group, sex, childbearing potential of women, Professional activity, number of hours/24h, number of days/week, shift worker/professional driver),

Medical History (Significant surgical and medical history; Any history (no, yes), and if yes, status (no, yes), ongoing (no, yes) for each pre-defined body system, Patient under nCPAP at V1 and V2)

OSA and EDS History: Time since diagnosis (month), Apnea-hypopnea index (AHI), For patients using nCPAP: Time since residual sleepiness on nCPAP, nCPAP pressure (cm H_2O), Mean nocturnal SaO_2 (%)

Nocturnal sleep: Number and duration (h) of daytime sleep and sleepiness episodes, Number and duration (h) of nocturnal awakening episodes

Mini-Mental State Examination (MMSE)

Polysomnography: Total sleep time [TST] (min), total sleep period [TSP] (min), sleep latency (min), For wake after sleep onset, Stage 1, Stage 2, Stage 3, and REM: Duration (min), % of TST, Duration of TST < 90 (O2 saturation) (min), Mean nocturnal SaO2 (%), Apnea-hypopnea index (AHI), Number of awakenings, Respiratory Micro Arousal Index, Non-respiratory Micro Arousal Index, Index of periodic limb movements, For patients under nCPAP, leak of CPAP mask (none, minor only, major only, major and minor, unspecified)

Baseline values of all the efficacy endpoints: ESS, OSLER, CGI-S, fatigue, and other endpoints described in the Efficacy section.

All the CRF-reported baseline characteristics will be compared across treatment groups. Categorical variables will be reported as category count (%), ordinal and continuous variables not distributed according a normal distribution as Median [IQ range] (n) , and continuous variable distributed according normal distribution as Mean±SD (n). Homogeneity tests between treatment groups will be provided using two-sided Student's t-test for continuous variables, chi-squared tests for categorical variables (These tests not really needed, as the trial was randomized, but traditionally reported).

4.2 Course of the Study

Date of first and last inclusion visit and last date of end of double-blind phase will be given for the entire FAS, and broken down by study center. Descriptive statistics for double-blind phase duration, and frequency distributions of premature study discontinuation and its reasons, will be given

- For the entire FAS
- Broken down by treatment group including a chi-squared statistic for comparison
- Broken down by treatment group and center.

A documented list will be given of patients withdrawn from the study.

4.3 Primary Efficacy Variable and Related Variables (ESS)

1-Description: each variable will be reported by treatment and CPAP on ITT sample. In the summary table, mean values +SD (n) will be provided for ESS at each DB visit, mean change Final-Baseline and Mean change percent over baseline (See table ess-1 below), in separating CPAP and no CPAP and the whole sample. No imputation is needed at this stage.

2-Figures : Final and Mean change in time during DB and OL (like figure 5A,5B,6A,6B HAROSA1-CSR)

3-Inferential models: Models 1, 2 and 3 will be applied as described in section 3.7

4.4 Secondary Efficacy Variables

4.4.1 OSleR Test

Description: OSLER and OSLC at V2 and V6 and their ratio V6/V2 will be summarized by their geometric mean [95%CI] (n).

Inference: The significance of the Treatment effect on the log-transformed Log(OSLf) adjusted for its baseline Log(OSLb) will be assessed by a linear analysis of covariance according to above Models 1, 2 and 3. BOCF MD-allocation will be used. The results will be expressed as anti-logs of parameters and CI for clinical interpretation. Comparison between treatments will be provided both on ITT and PP.

Supportive Analysis: Because MWT was censored at 40 minutes, a supportive Cox proportional regression will be conducted in considering time to sleep onset as survival response, right-censored after 40 minutes. Patients early terminating for treatment-related or -unrelated reason will be considered as responders or right-censored at the time of interruption, respectively.

4.4.2 *Clinical Global Impression (CGI) and Patient Global Impression(PGI)*

Description: Frequency distributions for CGI-S at V1 and V2, CGI-C at V6 and V7, CGI-C improvement at V6 and V7, PGI at visit 6 and 7 will be provided. A summary of descriptive values will be provided as showing the frequency distribution of CGI-S into Mild, Moderate and Severe groups, followed by CGI mean values and PGI mean values at V6.
Inference: CGI mean value at V6 will be analyzed according to model 1. CGI improvement (no/yes) at V6 will be assessed using a nonlinear logistic mixed model according to Models 1, 2 and 3, followed by a comparison. Odds ratio and Risk Ratios will be provided. BOCF allocation will be used. Comparison between treatments will be provided both on ITT and PP. for PGI only Model 1 will be used by providing Odds ratio and Risk Ratio.

4.5 *Exploratory endpoints*

4.5.1 *Aggregate z-score of ESS and OSleR*

Description: The mean + SD (n) aggregate EDS composed by OSLER and ESS will be reported according the following table. Only ITT sample will be analyzed and no imputation used.

Inference: The significance of the Treatment effect on the EDS-Z score will be assessed by a linear analysis of covariance according to above Models 1, 2 and 3. BOCF MD-allocation will be used. Comparison between treatments will be provided both on ITT and PP.

4.5.2 *Therapy Response*

Description: Observed proportions will be provided for R_1 and R_2, and summarized as in below table Therapy-Response. Final values based on LCF/BCF imputations. Only ITT values are provided

Inference: CGI improvement (no/yes) at V6 will be assessed using a nonlinear logistic mixed model according to Models 1, 2 and 3, followed by a comparison. BOCF allocation will be used. ITT sample will be only reported. Comparison between treatments will be provided both on ITT and PP. Odds Ratios (from logistic inference) and Risk Ratio (from Quasi Poisson inference) will be provided.

4.5.3 *Sleep Diary*

Description: Mean +SD (n) will be provided for SDWD, SDNS, SDDS, and SDAD for baseline (v1-v2 period) and final (v5-v6) period, and of the difference between the baseline period (V1-V2) and the stable treatment period (V5-V6).

Inference: The significance of the Treatment effect on SDWD, SDAD, SDNS, and SDDS during the stable treatment period will be assessed by a linear analysis of covariance according to Main Model 1 adjustment. No missing data imputation will be used and only ITT sample will be analyzed.

4.5.4 Quality of Life Test Euro Qol-5D (EQ-5D)

Description: Frequency distributions will be provided for EQ5Di (i=1,5) at V2 and V6 and mean+SD be provided for EQ5DS, EQ5DVAS, and EQ5DZ at V2 and V6, and for the difference between V2 and V6 as shown in the below EQ5D table .

Inference: the significance of the Treatment effect on Quality of life SDWD, SDAD, SDNS, and SDDS during the stable treatment period will be assessed by a linear analysis of covariance according to Main Model 1 adjustment. No missing data imputation will be used and only ITT sample will be analyzed.. Further to observed unimodal and reasonably symmetrical distributions, linear model are used.

4.5.5 Leeds Sleep Evaluation Questionnaire (LSEQ) , Trail Making Tests A&B, and Pichot Fatigue

The analysis of these two tests is similar with a common presentation

Description: Descriptive statistics will be provided for GTS, QOS, AFS, BFW (LSEQ) , TMTA and TMTB (Trail test) and Pichot Fatigue at V2 and V6, and for the difference between V2 and V6. No imputation for these tables.

Inference: the significance of the Treatment effect on GTS, QOS, AFS, and BFW will be assessed by a linear analysis of covariance according to Main Model 1 adjustment. No missing data imputation will be used and only ITT sample will be analyzed. Further to observed unimodal and reasonably symmetrical distributions, linear model are used.

4.6 Safety Analysis

4.6.1 Adverse Events

4.6.1.1 Summary Statistics

The analysis of TEAEs will consist of:

- Frequency distributions of patients with TEAEs, with TEAESIs, with related TEAEs, with treatment-emergent serious adverse events (SAEs), and with TEAEs causing premature study discontinuation.
- Frequency distribution of patients with TEAEs and with related TEAEs by:
    - MedDRA SOC*
    - MedDRA SOC and preferred term (PT)*
    - MedDRA SOC, PT, and maximal intensity
    - MedDRA SOC, PT, and sex
    - MedDRA SOC, PT, and age group

  * These distributions will also mention the numbers of events
- Number of events and frequency distribution of patients with TEAESIs by:
    - Types of MedDRA PTs
    - Types of MedDRA PTs and maximal intensity
- Additional information to be added to the tables as defined as follows:

- Mean weighted number of EA per patient (mWnEA) defined as the sum of each EA weighted by the product of severity (.5=Mild, 1=Moderate, 2=severe) by duration (number of weeks) , thus MWnEA is the number of equivalent Moderate EA with one week duration.

  Each sub-table will be ordered by increasing value of the ratio Rate (imp/Plac) of the mWnEA
- Median duration of the TEAE in days
- Median [IQ] for incidence time.

4.6.1.2 Analysis

The frequencies of patients with TEAEs, with TEAESIs, with related TEAEs, with treatment-emergent SAEs, and with TEAEs causing premature study discontinuation, will be analyzed using a nonlinear logistic mixed model, considering treatment and use of nCPAP as fixed factors, and center as random factor.

4.6.2 Laboratory Evaluations

4.6.2.1 Summary Statistics

Description for hematology, biochemistry and electrolytes variables at each assessment time, change between V1 and V6, proportion above the ULN at each assessment time.

4.6.2.2 Inferential

Covariance analysis for each adjusted for baseline, age, gender, treatment.

4.6.3 Vital Signs

4.6.3.1 Summary Statistics

Mean and mean change of Weight, BMI, blood pressure, heart rate at each visit

4.6.3.2 Analysis

Covariance analysis for each adjusted for baseline, age, gender, treatment.

4.6.4 Physical Examination

4.6.4.1 Summary Statistics

Proportion of abnormal values for each category

4.6.4.2 Analysis

Logistic covariance analysis for each adjusted for baseline, age, gender, treatment, center .

4.6.5 ECG Data

4.6.5.1 Summary Statistics

Mean values and mean change of PR, QRS, QT, QTcB, QTcF at each visit.

Frequency distributions will be provided for sinusal rhythm and overall result, at each assessment time, and for QT500, QTcF450, and ΔQTcF.

4.6.5.2 Analysis

Covariance analysis for each adjusted for baseline, age, gender, treatment.

4.6.5.3 Data List

A data list will document individual values for any patient presenting with an outlier ECG value at any time point post dose (V3 to V7). The list will mention the patient number, treatment, age, gender, and all recordings of PR, QRS, QT, QTcB, and QTcF. Patients will be selected if ΔQTcF = yes or if for any post-baseline such that PR>220 or QRS>120 or QT>500 or QTcB>450 or QTcF>450

4.6.6 Beck Depression Inventory (BDI)

4.6.6.1 Summary Statistics

The mean +SD and mean change sum-score of BDI-II will be provided at each visit

4.6.6.2 Analysis

Covariance analysis for each adjusted for baseline, age, gender, treatment.

4.6.7 Amphetamine-like Withdrawal Symptoms

4.6.7.1 Summary Statistics

Frequency distributions will be provided for each symptom (yes, no) and for amphetamine-like withdrawal syndrome (yes, no) at each assessment time.

4.6.7.2 Analysis

No inferential analysis will be performed on amphetamine-like withdrawal symptoms.

4.6.8 Patient's Overall Evaluation of Tolerance

4.6.8.1 Summary Statistics

Frequency distributions will be provided for each assessment time (V3 for 1st and 2nd week, V4, V5, V6, V7).

4.6.8.2 Analysis

No inferential analysis will be performed on the patient's overall evaluation of tolerance.

4.7 Concomitant Medications

Frequency distributions will be provided of subjects using any concomitant medication, and subjects using any concomitant medication by Anatomical class, and by Anatomical and Therapeutic class.

4.8 Exposure, Dosing, and Compliance

Descriptive statistics will be given for double-blind treatment exposure.

Frequency distributions will be given for the dose prescribed at V3, V4, and V5 (stable dose), and the maximum dose prescribed. A comparison will be performed of the stable dose and the maximum dose prescribed using logistic regression, considering treatment and use of nCPAP as fixed factors and center as a random factor.

Descriptive statistics will be given for compliance during the periods V2-V3, V3-V4, V4-V5, and V5-V6, and overall compliance. A comparison will be made of compliance in the period V5-V6 and of overall compliance using a linear mixed effects model, considering treatment and use of nCPAP as fixed factors and center as a random factor.

5 Statistical Tables[1]

5.1 Demography and Baseline Characteristics

All summary tables in this section will be conducted on the FAS.

5.1.1 Demography

Table 14.1.1.1	Age – Descriptive Statistics
Table 14.1.1.2	Age group – Frequency distribution
Table 14.1.1.3	Sex – Frequency distribution
Table 14.1.1.4	Childbearing potential – Frequency distribution
Table 14.1.1.5	Professional activity – Frequency distribution
Table 14.1.1.6	Number of hours/24h, Number of days/week – Descriptive statistics
Table 14.1.1.7	Shift worker/professional driver – Frequency distribution

5.1.2 Medical History

Table 14.1.2.1	Any significant medical history – Frequency distribution
Table 14.1.2.2	Status of medical history – Frequency distribution
Table 14.1.2.3	Patient under nCPAP at V1 and V2 – Frequency distribution

5.1.3 Obstructive Sleep Apnoea (OSA) and Excessive Daytime Sleepiness

Table 14.1.3.1	Time since diagnosis – Descriptive statistics
Table 14.1.3.2	Apnea-hypopnea index (AHI) – Descriptive statistics
Table 14.1.3.3	For patients under nCPAP: Time since residual sleepiness on nCPAP (OSA nCPAP beginning) (day), nCPAP pressure (cm H_2O), mean nocturnal SaO_2 (%) – Descriptive statistics

5.1.4 Nocturnal Sleep

Table 14.1.4.1	Number of daytime sleep and sleepiness episodes, duration of daytime sleep and sleepiness episodes (h), number of nocturnal awakening episodes, duration of nocturnal awakening episodes (h) – Descriptive statistics

5.1.5 MMSE

Table 14.1.5.1	MMSE score – Descriptive statistics

5.1.6 Polysomnography

Table 14.1.6.1	Total sleep time [TST] (min), total sleep period [TSP] (min), sleep latency (min) – Descriptive statistics
Table 14.1.6.2	Wake after sleep onset, Stage 1, Stage 2, Stage 3, and REM sleep: Duration (min), % of TST – Descriptive statistics
Table 14.1.6.3	Duration of TST < 90 (O_2 saturation) (min), mean nocturnal SaO_2 (%), apnea-hypopnea index (AHI), number of awakenings, Respiratory Micro Arousal

[1] Table and listing numbers and titles are indicative, and are subject to adaptation if required to improve readability.

	Index, Non-Respiratory Micro Arousal Index, index of periodic limb movements – Descriptive statistics
Table 14.1.6.4	For patients under nCPAP, leak of CPAP mask (none, minor only, major only, major and minor, unspecified) – Frequency distribution

5.2 Efficacy

All summary tables in this section will be conducted on both the FAS and the PP Population. Tables 14.2.1 are for the FAS while Tables 14.2.2 concern the PP Population.

5.2.1 Primary Efficacy Variable and Related Variables

Table 14.2.1.1.1	Epworth Sleepiness Scale (ESS) at each assessment time - Descriptive statistics
Table 14.2.1.1.2	Final ESS (DBF-LOCF, DBF-BOCF) - Descriptive statistics
Table 14.2.1.1.3	Change between ESS at V2 and final ESS (DBF-LOCF, DBF-BOCF) (Final – V2) - Descriptive statistics
Table 14.2.1.1.4	Analysis of DBF-LOCF ESS – ANCOVA (primary analysis)
Table 14.2.1.1.5	Analysis of DBF-BOCF ESS – ANCOVA (sensitivity analysis)
Table 14.2.1.1.6	Analysis of DBF-LOCF ESS – ANCOVA (sensitivity analysis including nCPAP by treatment interaction)
Table 14.2.1.1.7	Analysis of DBF-LOCF ESS – ANCOVA (sensitivity analysis without BMI)
Table 14.2.1.1.8	Analysis of DBF-LOCF ESS – ANCOVA (sensitivity analysis without ESS and BMI at V2)
Table 14.2.1.1.9	Percentage change between ESS at V2 and final ESS (DBF-LOCF, DBF-BOCF) - Descriptive statistics
Table 14.2.1.1.10	Final ESS (DBF-LOCF, DBF-BOCF) - Descriptive statistics by center
Table 14.2.1.1.11	Epworth Sleepiness Scale (ESS) at each assessment time - Descriptive statistics by center
Table 14.2.1.1.12	Change between ESS at V2 and final ESS (DBF-LOCF, DBF-BOCF) (Final – V2) - Descriptive statistics by center

5.2.2 Secondary Efficacy Variables

5.2.2.1 Response

Table 14.2.1.2.1.1	Response (R_1 and R_2) – Frequency distribution and exact 95% confidence interval
Table 14.2.1.2.1.2	Response (R_1 and R_2) – Nonlinear logistic mixed model

5.2.2.2 Sleep Diary

Table 14.2.1.2.2.1	SDWD, SDAD, SDNS, and SDDS at each assessment period – Descriptive statistics
Table 14.2.1.2.2.2	For SDWD, SDAD, SDNS, and SDDS difference between the baseline period (V1-V2) and stable treatment period (V5-V6) – Descriptive statistics

Table 14.2.1.2.2.3 For SDWD, SDAD, SDNS, and SDDS during the stable treatment period (V5-V6) – ANCOVA

5.2.2.3 OSleR test

Table 14.2.1.2.3.1 OSL at V2 and V6 – Summary statistics

Table 14.2.1.2.3.2 OSL at V6/OSL at V2 - Summary statistics

Table 14.2.1.2.3.3 log(OSL) at V6 – ANCOVA

Table 14.2.1.2.3.4 OSLC at V2 and V6 - Frequency distribution and exact 95% confidence interval

Table 14.2.1.2.3.5 OSLC at V6 - Nonlinear logistic mixed model

5.2.2.4 EQ-5D

Table 14.2.1.2.4.1 EQ5Di (i=1,5) at V2 and V6 – Frequency distribution

Table 14.2.1.2.4.2 EQ5DS, EQ5DVAS, and EQ5DZ at V2 and V6 – Descriptive statistics

Table 14.2.1.2.4.3 For EQ5DS, EQ5DVAS, and EQ5DZ, difference between V2 and V6 – Descriptive statistics

Table 14.2.1.2.4.4 EQ5Di (i=1,5) at V6 – Nonlinear logistic mixed model

Table 14.2.1.2.4.5 EQ5DS, EQ5DVAS, and EQ5DZ at V6 – ANCOVA

5.2.2.5 LSEQ

Table 14.2.1.2.5.1 GTS, QOS, AFS, and BFW at V2 and V6 – Descriptive statistics

Table 14.2.1.2.5.2 Difference between GTS, QOS, AFS, and BFW at V2 and V6 – Descriptive statistics

Table 14.2.1.2.5.3 GTS, QOS, AFS, and BFW at V6 – ANCOVA

5.2.2.6 TMT

Table 14.2.1.2.6.1 TMTA and TMTB at V2 and V6 – Descriptive statistics

Table 14.2.1.2.6.2 For TMTA and TMTB difference between V2 and V6 – Descriptive statistics

Table 14.2.1.2.6.3 TMTA and TMTB at V6 – ANCOVA

Table 14.2.1.2.6.4 TMTA and TMTB deficiency at V2 and V6 – Frequency distribution and exact 95% confidence interval

5.2.2.7 CGI

Table 14.2.1.2.7.1 CGI-S at V1 and V2 – Frequency distribution

Table 14.2.1.2.7.2 CGI-C at V6 and V7 – Frequency distribution

Table 14.2.1.2.7.3 CGI-C improvement at V6 and V7 – Frequency distribution and exact 95% confidence interval

Table 14.2.1.2.7.4 CGI-C improvement at V6 – Nonlinear logistic mixed model

5.2.2.8 PGOE

Table 14.2.1.2.8.1 PGOE at each assessment time – Frequency distribution

Table 14.2.1.2.8.2 PGOE improvement at each assessment time – Frequency distribution and exact 95% confidence interval

Table 14.2.1.2.8.3	PGOE improvement at V6 – Nonlinear logistic mixed model

5.2.2.9 Pichot Fatigue Scale

Table 14.2.1.2.9.1	Pichot Fatigue Scale score at each assessment time – Descriptive statistics
Table 14.2.1.2.9.2	Difference between Pichot Fatigue Scale score at V2 and V6 – Descriptive statistics
Table 14.2.1.2.9.3	Pichot Fatigue Scale score at V6 – ANCOVA

5.2.2.10 Stable Activity

Table 14.2.1.2.10.1	Maintaining stable activity at each assessment time – Frequency distribution and exact 95% confidence interval
Table 14.2.1.2.10.2	Maintaining stable activity at V6 – Nonlinear logistic mixed model

5.2.2.11 Aggregate Z-score of ESS and OSleR

Table 14.2.1.2.11.1	Aggregate Z-score at each assessment time – Descriptive statistics
Table 14.2.1.2.11.2	Difference between Aggregate Z-score at V2 and V6 – Descriptive statistics
Table 14.2.1.2.11.3	Aggregate Z-score at V6 – ANCOVA

5.3 Safety

All summary tables in this section will be conducted on Safety Population.

5.3.1 <u>Adverse Events</u>

Table 14.3.1.1	Patients with TEAEs, with related TEAEs, with treatment-emergent SAEs, and with TEAEs causing premature study discontinuation – Frequency distribution and logistic regression
Table 14.3.1.2	Patients with TEAEs by MedDRA SOC and PT – Frequency distribution
Table 14.3.1.3	Patients with TEAEs by MedDRA SOC, PT, and maximum severity – Frequency distribution
Table 14.3.1.4	Patients with TEAEs by MedDRA SOC, PT, and sex – Frequency distribution
Table 14.3.1.5	Patients with TEAEs by MedDRA SOC, PT, and age group – Frequency distribution
Table 14.3.1.6	Patients with related TEAEs by MedDRA SOC and PT – Frequency distribution
Table 14.3.1.7	Patients with related TEAEs by MedDRA SOC, PT, and maximum severity – Frequency distribution
Table 14.3.1.8	Patients with related TEAEs by MedDRA SOC, PT, and sex – Frequency distribution
Table 14.3.1.9	Patients with related TEAEs by MedDRA SOC, PT, and age group – Frequency distribution
Table 14.3.1.10	Patients with TEAESIs by MedDRA PT – Frequency distribution and number of events

Table 14.3.1.11 Patients with TEAESIs by MedDRA PT and maximum severity – Frequency distribution and number of events

5.3.2 ***Laboratory Evaluations***

Table 14.3.2.1 Hematology variables at each assessment time and change from baseline – Descriptive statistics

Table 14.3.2.2 Hematology: Status of each variable at each assessment time – Frequency distribution

Table 14.3.2.3 Hematology: Status of each variable at V6 broken down by status at V1 – Frequency distribution

Table 14.3.2.4 Biochemistry variables at each assessment time and change from baseline – Descriptive statistics

Table 14.3.2.5 Biochemistry: Status of each variable at each assessment time – Frequency distribution

Table 14.3.2.6 Biochemistry: Status of each variable at V6 broken down by status at V1 – Frequency distribution

Table 14.3.2.7 Electrolyte variables at each assessment time and change from baseline – Descriptive statistics

Table 14.3.2.8 Electrolytes: Status of each variable at each assessment time – Frequency distribution

Table 14.3.2.9 Electrolytes: Status of each variable at V6 broken down by status at V1 – Frequency distribution

5.3.3 ***Vital Signs***

Table 14.3.3.1 Weight, BMI, blood pressure, and heart rate at each assessment time and change from baseline – Descriptive statistics

5.3.4 ***Physical Examination***

Table 14.3.4.1 Each body system, at each assessment time – Frequency distribution

5.3.5 ***ECG Data***

Table 14.3.5.1 Heart rate, PR, QRS, QT, QTcB, and QTcF at each assessment time, at baseline (average of V1 and V2), for the stable treatment period (average of V4, V5, and V6), and change from baseline – Descriptive statistics

Table 14.3.5.2 Sinusal rhythm and ECG overall result at each assessment time – Frequency distribution

Table 14.3.5.3 QT500, QTcF450, and ΔQTcF – Frequency distribution

5.3.6 ***BDI***

Table 14.3.6.1 BDI 13 items score at each assessment time – Descriptive statistics

Table 14.3.6.2 Difference between BDI 13 items score at V2 and V6 – Descriptive statistics

Table 14.3.6.3 BDI 13 items score category at each assessment time – Frequency distribution

Table 14.3.6.4 BDI Item G at each assessment time – Frequency distribution

Table 14.3.6.5	BDI 13 items score at V6 – ANCOVA
Table 14.3.6.6	BDI 13 items score category at V6 – Ordinal logistic regression

5.3.7 *Amphetamine-like Withdrawal Symptoms*

Table 14.3.7.1	For each symptom at each assessment time – Frequency distribution
Table 14.3.7.2	Amphetamine-like withdrawal syndrome at each assessment time – Frequency distribution

5.3.8 *Patient's Overall Evaluation of Tolerance*

Table 14.3.8.1	Patient's overall evaluation of tolerance at each assessment time – Frequency distribution

5.4 *Concomitant Medications*

All summary tables in this section will be conducted on the Safety Population.

Table 14.4.1	Use of any concomitant medication – Frequency distribution
Table 14.4.2	Use of any concomitant medication by Anatomical class– Frequency distribution
Table 14.4.3	Use of any concomitant medication by Anatomical and Therapeutic class – Frequency distribution

5.5 *Exposure, Dosing, and Compliance*

All summary tables in this section will be conducted on the Safety Population.

Table 14.5.1	Exposure to double-blind treatment – Descriptive Statistics
Table 14.5.2	Dose prescribed at V3, V4, and V5 (stable dose), and maximum dose prescribed – Frequency distribution and logistic regression
Table 14.5.3	Compliance during the periods V2-V3, V3-V4, V4-V5, and V5-V6, and overall compliance – Descriptive Statistics
Table 14.5.4	Compliance during the period V5-V6 and overall compliance – ANCOVA

5.6 *Sample Description*

- Patient Disposition

Table 14.6.1	Number of patients in each of the analysis sets
Table 14.6.2	Number of patients in each of the analysis sets in each center
Table 14.6.3	Number of patients in each of the analysis sets at each visit

- Protocol Deviations

Table 14.6.4	Patients having at least one major deviation - Frequency distribution
Table 14.6.5	Patients having at least one major deviation by type of deviation - Frequency distribution
Table 14.6.6	Patients having at least one deviation - Frequency distribution
Table 14.6.7	Patients having at least one deviation by type of deviation - Frequency distribution

5.7 Course of the Study

- Study Dates

Table 14.7.1	For the entire FAS, and broken down by center, date of first and last inclusion visit and date of last visit

- Study Duration and Premature Discontinuation

For the FAS and for the Safety set, broken down by treatment group:

Table 14.7.2	Double-blind phase duration – Descriptive Statistics
Table 14.7.3	Premature discontinuation - Frequency distribution
Table 14.7.4	Reasons for premature discontinuation - Frequency distribution

6 Listings of Individual Data

16.2.1	Discontinued Patients
16.2.2	Protocol Deviations
16.2.3	Patients Excluded from the Analysis Sets
16.2.4	Demography and Other Baseline Data
16.2.4.1	Demography
16.2.4.2	Obstructive Sleep Apnea, Excessive Daytime Sleepiness, and MMSE
16.2.4.3	Nocturnal Sleep
16.2.4.4	Medical History
16.2.4.5	Polysomnography
16.2.5	Study Treatment
16.2.5.1	Study Treatment Administration
16.2.5.2	Compliance
16.2.6	Efficacy Data
16.2.6.1	ESS
16.2.6.2	Sleep diary
16.2.6.3	OSLeR test
16.2.6.4	Quality of Life (EQ-5D)
16.2.6.5	Other Secondary Efficacy Variables
16.2.7	Adverse Events
16.2.7.1	Non-Treatment Emergent Events
16.2.7.2	Treatment Emergent Adverse Events
16.2.7.3	Deaths and Serious Adverse Events
16.2.7.4	Adverse Events Leading to Study Drug Interruption or Discontinuation
16.2.8	Laboratory Data
16.2.8.1	Laboratory Data – Hematology
16.2.8.2	Laboratory Data – Biochemistry

16.2.8.3	Laboratory Data – Electrolytes
16.2.8.4	Laboratory Data – Urinalysis
16.2.8.5	Laboratory Data – ß-HCG serum pregnancy test, HBsAg, HCV, HIV
16.2.9	Vital Signs
16.2.10	Physical Examination
16.2.11	ECG
16.2.11.1	ECG – Recorded Data
16.2.11.2	ECG – Outliers
16.2.12	Beck Depression Inventory
16.2.13	Amphetamine-like Withdrawal Symptoms and Patient's Overall Evaluation of Tolerance
16.2.14	Prior and Concomitant Medications

Whenever applicable, listings will present calculated variables. For quantitative assessments for which a change from baseline is described in the statistical tables, the change from baseline will also be listed.

If it is required to present much information on listings, listings may be presented in several parts, in order to enhance readability.

7 Overview of Assessments

PERIOD						W 1	W 2		W 3		W4 → W7		W8 → W12		W13		
**Visit**[1]	**V1 D-14**	1 Week Wash-out	Phone contact 1 D-7	1 Week	**V2 D0**	10 mg	20 mg	**V3 D14**	10/20/40 mg	**V4 D21**	Selected	**V5 D49**	Selected	**V6 D84**	One week wash-out	Phone contact 2 V6+3D	**V7**[6] End of study
Signature of Consent Form	X																
Medical questionnaire	X		X		X			X		X		X		X		X	X
Physical examination	X				X			X		X		X		X			X
MMSE	X																
Polysomnography[2]				X													
ESS	X				X			X		X		X		X			X
OSleR test[3]					X									X			
TMT PARTS A&B					X									X			
CGI-S	X				X												
CGI-C														X			X
BDI-13	X				X									X			X
LSEQ, EQ-5D					X									X			
Pichot Fatigue Scale					X									X			X
Patient's global opinion on the effect of investigational drug														X		X	X
Patient's overall evaluation of tolerance								X		X		X		X			X
Amphetamine like withdrawal symptoms																X	X
ECG	X				X			X		X		X		X			X
Safety biology[4]	X													X			
Delivery of sleep diary	X				X			X		X		X		X			
Review of Sleep diary[5]					X			X		X		X		X			X
Adverse events			X		X			X		X		X		X		X	X

1 – Each visit shall be carried out at the end of the relevant time period ± 3 days.

2 – Overnight polysomnographic recording performed from 22:00 until 7:00 (minimum 8 hours of recording) in the sleep laboratory only between V1 and V2 except if available in the previous 12 months.

3 – OSleR test: 3 sequences at 2 hours interval (at 9:00, 11:00, and 13:00).

4 – Complete biological examination: hematology (hemoglobin, hematocrit, red and white blood cell count (with differential), platelets, mean corpuscular volume, coagulation time (INR)), biochemistry (blood urea nitrogen (BUN), uric acid, creatinine, creatine kinase, ALAT, ASAT, GGT, alkaline phosphatases, total protein, total bilirubin, glucose, electrolytes (sodium, potassium, calcium, chloride, bicarbonates/CO_2), total cholesterol, triglycerides), serology (HIV, HCV, HBsAg at V1, β-HCG (for woman with child-bearing potential)), urinalysis: stick (with microscopy and bacteriological culture, if positive) at V1.

5 – At each visit, the patient shall bring back his sleep diary. Patient will be contacted in advance before each visit to remind him/her to fill in the sleep diary. The patient shall return the unused drug at each visit.

6 – Only for patients who are not entering into Open Label Extension period; for others see Open Label Extension period study diagram.

Statistical Analysis Plan for the Open Label Phase of Study P1513:

Efficacy and Safety of Pitolisant (BF2.649) in the Treatment of Excessive Daytime Sleepiness in Patients with Obstructive Sleep Apnoea Syndrome, Treated or Not by Nasal Continuous Positive Airway Pressure, but still complaining of Excessive Daytime Sleepiness

EUDRACT NUMBER: 2015-004561-85

**Version**	**Date**	**Author**	**Job Title**	**Status**
0.1	30 March 2018	[redacted]	Statistician	Draft
0.2	03 March 2021	[redacted]	Statistician	Draft
0.3	22 March 2021	[redacted]	Statistician	Draft
0.4	29 March 2021	[redacted]	Statistician	Draft
1.0	30 March 2021	[redacted]	Statistician	Final
1.1	31 May 2021	[redacted]	Statistician	Draft
2.0	1 June 2021	[redacted]	Statistician	Final
3.0	30 June 2021	[redacted]	Statistician	Final

For Approval:

Senior Consulting Statistician:	Prof. Dr. [redacted]<br>Faculty of Economics<br>UCL University, Mons<br>185, chaussée de Binche<br>B-7000 Mons<br>BELGIUM	[redacted]<br>Sig	21-07-2021<br>Date
Head of Biostatistics:	[redacted]<br>Data Investigation Company Europe<br>16 ‘t Hofveld<br>B-1082 Brussels<br>BELGIUM	[redacted]<br>Signature	20/07/2021<br>Date

Table of Contents

Table of Contents 2
1 Introduction 6
1.1 Aims 6
1.2 Study Design 6
1.3 Study Treatment 7
1.4 Objectives 7
1.4.1 Efficacy 7
1.4.2 Safety 7
2 Data Considered for the Analysis 8
2.1 Data Only Recorded at Onset of the Study 8
2.2 Efficacy Data 8
2.3 Safety Data 10
2.4 Course of the Study 13
2.5 Exposure, Treatment Dose, and Compliance 13
3 Statistical Considerations 15
3.1 Analysis Sets 15
3.2 Missing Data 15
3.3 Summary Statistics 15
3.4 Inferential Statistical Analysis 15
3.5 Accounting for Multiple Centers 16
3.6 Multiple Testing 16
3.7 Miscellaneous 16
3.8 Changes from the Analyses Foreseen in the Protocol 16
4 Generalities 17
4.1 Sample Description 17
4.2 Onset Data 17
4.3 Course of the Study 17
4.4 Data lists 17
4.5 Figure 18
5 Efficacy Analysis 19
5.1 ESS 19
5.1.1 Summary Statistics 19
5.1.2 Analysis 19
5.1.3 Graphics 19
5.2 Therapy Response 19
5.2.1 Description 19

5.2.2 Inference 19
5.3 Sleep Diary 20
5.3.1 Summary Statistics 20
5.3.2 Analysis 20
5.4 OSleR test 20
5.4.1 Summary Statistics 20
5.4.2 Inference 20
5.5 Quality of life test Euro Qol-5D (EQ-5D) 20
5.5.1 Summary Statistics 20
5.5.2 Analysis 20
5.6 Leeds Sleep Evaluation Questionnaire (LSEQ) 21
5.6.1 Summary Statistics 21
5.6.2 Analysis 21
5.7 Trail Making Test (TMT) Parts A&B 21
5.7.1 Summary Statistics 21
5.7.2 Analysis 21
5.8 Clinical Global Impression (CGI) 21
5.8.1 Summary Statistics 21
5.8.2 Inference 21
5.9 Patient's Global Opinion (PGOE) 21
5.9.1 Summary Statistics 21
5.9.2 Analysis 21
5.10 Pichot Fatigue Scale 22
5.10.1 Summary Statistics 22
5.10.2 Analysis 22
5.11 Stable Activity 22
5.11.1 Summary Statistics 22
5.11.2 Analysis 22
5.12 Aggregate z-score of ESS and OSleR 22
5.12.1 Summary Statistics 22
5.12.2 Analysis 22
6 Safety Analysis 23
6.1 Adverse Events 23
6.2 Laboratory evaluations 23
6.3 Vital Signs 23
6.4 Physical examination 23
6.5 ECG Data 24
6.5.1 Summary Statistics 24
6.5.2 Data List 24
6.6 Beck Depression Inventory (BDI) 24
6.7 Amphetamine-like withdrawal symptoms 24
6.8 Patient's Overall Evaluation of Tolerance 24
6.9 Previous and Concomitant Treatment 24
6.10 Exposure, Dosing, and Compliance 24

7 Statistical Tables *25*
7.1 Demography and Baseline Characteristics 25
7.1.1 Demography 25
7.1.2 Medical History 25
7.1.3 Baseline Data 25
7.2 Efficacy 25
7.2.1 Efficacy Variables Based on the ESS (Except Response) 25
7.2.2 Other Efficacy Variables 26
7.3 Safety Evaluation 27
7.3.1 Adverse Events 27
7.3.2 Laboratory evaluations 28
7.3.3 Vital Signs 28
7.3.4 Physical examination 28
7.3.5 ECG Data 28
7.3.6 BDI 29
7.3.7 Amphetamine-like Withdrawal Symptoms 29
7.3.8 Patient's Overall Evaluation of Tolerance 29
7.4 Concomitant Medications 29
7.5 Exposure, Dosing, and Compliance 29
7.6 Sample Description 29
7.7 Course of the Study 30
8 Listings of Individual Data *31*
9 Overview of Assessments *32*
10 Overview of additional summary tables *34*
10.1 Summary table 1: Primary efficacy variable ESS – Continuous analysis 34
10.2 Summary table 2: Efficacy variable ESS – Responder analysis 34
10.3 Summary table 3: Efficacy variable OSler 35
10.4 Summary table 4: Efficacy variable CGI 35
10.5 Summary table 5: Efficacy variables LSEQ, TMT and Aggregate z-score 36

ABBREVIATIONS

AE	Adverse Event
AESI	Adverse Event of Special Interest
AHI	Apnoea Hypopnoea Index
ALAT/SGPT	Alanine Aminotransferase
ANCOVA	Analysis of Covariance Test
ASAT/SGOT	Aspartate Aminotransferase
BDI-13	Beck Depression Inventory-13 items
BMI	Body Mass Index
CI	Confidence Interval
CPK	Creatine Kinase
CRF	Case Report Form
DSE	Diurnal Sleepiness Episodes
ECG	Electrocardiogram
EDS	Excessive Daytime Sleepiness
EEG	Electroencephalography
ES	Effect Size
ESS	Epworth Sleepiness Scale
FAS	Full Analysis Set
GCP	Good Clinical Practice
GGT	Gamma-glutamyltranspeptidase
INR	International Normalized Ratio
MCV	Mean Corpuscular Volume
MMSE	Mini Mental State Examination
nCPAP	nasal Continuous Positive Airway Pressure
OD	Once a day
ODL	Optimal Dose Level
OL-FAS	Open Label Full Analysis Set
OL-SAF	Safety Population
OL-PP	Open Label Per Protocol
OSA	Obstructive Sleep Apnoea syndrome
OSleR	Oxford Sleep Resistance Test
PGOE	Patient's Global Opinion of the Effect
PSG	Polysomnography
SAE	Serious Adverse Event
SaO_2	Oxygen Saturation
SD	Standard Deviation
ß-hCG	Human Chorionic Gonadotropin
ODL	Optimal Dose Level
TEAE	Treatment-Emergent Adverse Event
TEAESI	Treatment-Emergent Adverse Event of Special Interest
TMT	Trail Making Test
TST	Total Sleep Time
WHO	World Health Organization

1 Introduction

1.1 Aims

The purpose of this statistical analysis plan (SAP) for the open label phase of the study is to provide details for the statistical analysis, and to ensure that the statistical methodologies that will be used, and the summary tables, figures, and data listings (TFLs), are in line with standard practice, and complete and appropriate to reach valid conclusions regarding the study objectives.

The analysis of the pharmacokinetic data falls outside the scope of this SAP. This SAP is based on the last version of the protocol of the study, being Version 2.0, dated 1 February 2018 and of the CRF Version 1.2, dated 16 February 2016.

1.2 Study Design

This is a prospective, multicenter (several centers in Bulgaria), randomized, Phase III study of pitolisant given at 10, 20, or 40 mg per day versus placebo.

It is carried out in patients diagnosed with Obstructive Sleep Apnoea (OSA) syndrome, without important cardiovascular disease, experiencing Excessive Daytime Sleepiness (EDS) with Epworth Sleepiness Scale (ESS) score ≥ 12, having refused to be treated by nasal Continuous Positive Airway Pressure (nCPAP) or having been submitted to nCPAP therapy for a minimum period of 3 months, and still complaining of EDS.

The study consists of two parts, a 12-week double-blind part starting with an escalating dose period followed by treatment with the selected dose. Then, after one week of single-blind placebo wash-out period, if the patient holds the same position towards nCPAP therapy as before, an Open Label Extension period is proposed.

Patients who do not participate in the Open Label Extension period will have their end of the study visit. Patients willing to continue the pitolisant treatment administration will be given another information notice together with an informed consent form to be signed. This Open Label Extension period will consist of the same escalating-dose regimen as in the 1st part of the study, followed by a selected dose period with the active drug only, until 52 weeks after the treatment beginning. Then, patients will have a one week wash-out period prior to the end of study visit.

An overview of the visits and assessments is given in Section 6.

With the exception of spending an optional one night (if not done during the twelve months preceding the study) in the sleep laboratory for the full recording of nocturnal polysomnography between V1 and V2, the patient are to be ambulatory during the whole study period.

Polysomnography is to be performed (for patients submitted to nCPAP therapy – under nCPAP) between V1 and V2 or during the last 12 months, with Apnea-Hypopnea Index (AHI) ≥ 15 for patients without nCPAP therapy and ≤ 10 for patients under nCPAP therapy. Patients submitted to nCPAP therapy should be treated for at least 4 hours per day. The compliance is checked on the clock-time counter of the CPAP machine.

The following visits were planned for the patients participating in the open label phase of the study:

- V1 = Screening visit (Day -14)
- PC1 = Phone contact n°1 (D-7)
- V2 = Inclusion visit (D0): Start of up-titration for the double-blind phase
- V3 = Visit 3 (D14±3): End of up-titration and first dose adjustment
- V4 = Visit 4 (D21±3): Second dose adjustment and beginning of the stable dose phase
- V5 = Visit 5 (D49±3): Continuation of the stable dose phase

- V6 = Visit 6 (D84±3): End of double-blind phase and beginning of single-blind wash-out period
- PC2 = Phone contact n°2 (V6 + 3 days)
- V7 = Visit 7 (D91±3)
  - For patients not entering the open label phase: End of study visit
  - For patients entering the open label phase: Start of up-titration for the open label phase
- V8 = Visit 8 (D105±3): First dose adjustment
- V9 = Visit 9 (D112±3): Second dose adjustment
- V10 = Visit 10 (D196±3): Confirmed dose visit
- V11 = Visit 11 (D280±3): Confirmed dose visit
- V12 = Visit 12 (D364±3): End of open label phase and beginning of single-blind wash-out period
- PC3 = Phone contact n°3 (V12 + 3 days)
- V13 = Visit 13 (D371±3): End of study visit for patients who entered the open label phase

An overview of the visits and assessments for patients who participate in the open label phase is given in Section 9.

1.3 Study Treatment

Pitolisant and placebo are presented in identical tablets according to dosage, i.e. pitolisant tablets dosed at 5 mg or 20 mg and matching placebo.

During the double-blind phase patients were to take daily 10 mg (2 tablets of 5 mg) or 20 mg (1 tablet of 20 mg) or 40 mg (2 tablets of 20 mg) of pitolisant or matching placebo. During the open label extension period patients were to take daily 10 mg, 20 mg or 40 mg of pitolisant (i.e. 2 tablets of 5 mg or 1 or 2 tablets of 20 mg, respectively).

Study medication was to be administered orally, once a day (OD), in the morning, during breakfast, with a glass of water.

1.4 Objectives

1.4.1 Efficacy

Based on this trial, starting with a DB period of 12 weeks, patients randomized at V2 until V6 followed by a wash out period of one week and from V7 followed by 40 weeks of Open Label during which all the patients received the IMP, with a final washout period between V12 and V13, the objectives are multiple:

1. For patients continuously treated with the IMP, assessment of a sustainment effect reached at 12 weeks until at least one year, the alternative hypothesis being ESS12 ≤ ESS6.
2. Separately for the two randomized groups, estimate of the overall ESS decrease at one-year ESS12-ESS2.
3. Assessing the difference between the two groups on the overall decrease V2-V12
4. Assessing the change on the IMP group during the period of washout V6-V7 and V12-V13 and for all the patients during V12 and V13.

1.4.2 Safety

Assessing the long-term tolerance, and further investigating the co-variates or co-medications that affect the pharmacokinetics of pitolisant in the target population to allow future comparison to healthy subjects.

2 Data Considered for the Analysis

2.1 Data Only Recorded at Onset of the Study

- Patient number and center number
- Date of screening visit
- Demographic Data
  - Age (year)
  - Age group calculated considering the following categories: <35 years, ≥ 35 and < 50 years, ≥ 50 and < 65 years, ≥ 65 years
  - Sex
  - Professional activity, number of hours/24h, number of days/week, night shift worker
- Medical History
  - Time since diagnosis (month)

2.2 Efficacy Data

- Epworth Sleepiness Scale (ESS)
  - Values of ESS at every visit (V1 to V13)
  - The final open label stable treatment score (OLF) is defined as the average of the non-missing values at V11 and V12.
    If both values are missing two definitions are used for estimating the final score: OLF-LOCF/BOCF ESS:
    - LOCF imputation for patients dropping out during the OL phase for reasons not related to treatment
    - BOCF imputation (V2) for patients dropping out during the OL phase for reasons related to treatment

  The allocation of treatment relatedness was done based on the same reasons for dropout and their relation as defined in the DRM for the double-blind part. For patients dropping out because of adverse events the discontinuation was considered related to treatment whenever the adverse event was defined as related to treatment.

  - OLF-LOCF/BOCF ESS – V7 (change versus baseline open label)
  - OLF-LOCF/BOCF ESS – V2 (change versus baseline double blind)
  - OLF-LOCF/BOCF ESS – V6 (compare with treatment effect at the end of DB)
  - 100 x (OLF-LOCF/BOCF ESS – V7)/V7 (percentage change versus baseline open label)
  - 100 x (OLF-LOCF/BOCF ESS – V2)/V2 (percentage change versus baseline double blind)
  - Therapy response : We considered two definition of therapy response : (1) R_1= OLF-LOCF/BOCF ESS values not exceeding 10, considered as the upper limit of EDS (Johns, 2009); (2) R_2= OLF-LOCF/BOCF ESS values not exceeding 10 or decrease from V2 of at least -3.
- Sleep Diary.

For 3 days:

  - SDR1: At what time did you get up this morning?
  - SDR2: How many sleep/sleepiness episodes did you experience today?
  - SDR3: At what time did you go to sleep last night?
  - SDR4: What was the total duration of your sleep/sleepiness episodes today?

  The following variables are calculated based on the available data for each period (V1-V2, V2-V3, V3-V4, V4-V5, V5-V6, V6-V7, V7-V8, V8-V9, V9-V10, V10-V11, V11-V12, V12-V13):

  - Wakefulness duration for Day i = SDR3 of Day i+1 - SDR1 of Day i
  - SDWD = Mean wakefulness duration = mean wakefulness duration for Days 1 and 2

  - Alertness duration for Day i = SDR3 of Day i+1 - SDR1 of Day i – SDR4 of Day i
  - SDAD = Mean daily alertness duration = mean alertness duration for Days 1 and 2
  - SDNS = Mean daily number of sleep/sleepiness episodes = mean of SDR2 for Days 1, 2, 3
  - SDDS = Mean daily duration of sleep/sleepiness episodes = mean of SDR4 for Days 1, 2, 3
- Oxford Sleep Resistance Test (OSleR) [V2, V6, V7, V12 (optional)]:
  - OSL (sec) = Mean sleep latency (mean of the 3 tests conducted at 3 times of the visit day) Higher values indicate better performance. In order to be able to calculate geometric means, values of 0 are replaced by10 seconds (half the minimal possible positive value).
  - OSLC (yes, no):
    Success (OSLC='yes') defined as number of 3-6 and ≥ 7 errors = 0 for each of the 3 tests. In case any of the variables is missing OSLC is considered to be missing.
- Aggregate z-score of ESS and OSleR (V2, V6, V7, V12):
  - ESSOSZ = Z(ESS) + Z(log(OSL)
    The standardization is performed separately for patients under nCPAP and not under nCPAP using the mean and SD of ESS and log(OSL) at V2 for the corresponding patients of the double-blind Full Analysis Set.
- EQ-5D Health Questionnaire (V2, V6, V7, V12):
  - Descriptive system (5 items scored as 1, 2, or 3);
    EQ5DS = sum of the 5 items. In case any of the items is missing EQ5DS is considered to be missing.
  - Regrouped items:
    1 (no problems) is transformed to 0
    2 (some problems) and 3 (extreme problems) are transformed to 1
  - EQ5DVAS = Visual Analog Scale (VAS)
  - EQ5DZ = Z(EQ5DS) + Z(EQ5DVAS):
    The standardization is performed separately for patients under nCPAP and not under nCPAP using the mean and SD of EQ5DS and EQ5DVAS at V2 for the corresponding patients of the double-blind Full Analysis Set.
- Leeds Sleep Evaluation Questionnaire (LSEQ) (V2, V6, V7, V12):
  - Scores for items 1 to 10

  The following scales are used in the analysis:
  - GTS (getting to sleep) = Mean of items 1, 2, and 3
  - QOS (quality of sleep) = Mean of items 4 and 5
  - AFS (awake following sleep) = Mean of items 6 and 7
  - BFW (behavior following wakening) = Mean of items 8, 9, and 10

  In case one item of a scale is missing the average is calculated of the remaining items. In case more than one is missing the scale is considered missing.
- Trail Making Test (V2, V6, V7, V12):
  - TMTA (sec)
  - TMTA deficiency: TMTA > 78 sec
  - TMTB (sec)
  - TMTB deficiency: TMTB > 273 sec
- Clinical Global Impression of Severity (CGI-S) regarding excessive daytime sleepiness (V1, V2):
  - CGI-S assessed as: 1=normal (not at all ill), 2=borderline ill, 3=mildly ill, 4=moderately ill, 5=markedly ill, 6=severely ill, 7=among the most extremely ill patients

- Clinical Global Impression of Improvement (CGI-C) regarding excessive daytime sleepiness symptoms (V6, V7, V10 to V13):
  - CGI-C assessed as: Very much improved, much improved, minimally improved, no change, minimally worse, much worse, very much worse
  - CGI-C improvement:
    - Yes = Very much improved, much improved, or minimally improved
    - No = No change, minimally worse, much worse, or very much worse
- Patient's Global Opinion of the Effect (PGOE) of the Investigational Drug compared to pre-study condition (V6, Phone contact 2, V7, V10, V11, V12, Phone contact 3, V13):
  - PGOE assessed as: Marked effect, moderate effect, minimal effect, no change, minimally worse, much worse
  - PGOE improvement:
    - Yes = Marked effect, moderate effect, or minimal effect
    - No = No change, minimally worse, or much worse
- Pichot Fatigue Scale (V2, V6, V7, V9 to V13):
  - Pichot Fatigue Scale Score
- Activity [Phone contact 1, V2, V3, V6 to V13]:
  - Did the patient maintain a stable activity since the last visit (yes, no)

2.3 Safety Data

- Adverse events (throughout the study). The following data are recorded for each event:
  - Description
  - MedDRA term
  - Date of onset or intensity change
  - Intensity (mild, moderate, severe)
  - Frequency (once, intermittent and number and period, continuous, unknown)
  - Action taken with studied drug (none, dose modification, transitory interruption, discontinued)
  - Action taken with event (none, symptomatic treatment, complementary exploration, hospitalization and date)
  - Serious (yes, no)
  - Data to be provided if the event disappears or at the end of the study:
    - Control date
    - Outcome (recovery with or without sequelae and date, ongoing, resolved, unknown, death related to adverse reaction, death not related to adverse reaction)
    - Etiology (studied drug, concomitant treatment, associated disease, other, unknown)
    - Imputability (likely, possible, unlikely)

  Adverse events were coded using the Medical Dictionary for Regulatory Activities (MedDRA). All adverse events were attributed a MedDRA Preferred Term (PT) and the primary System Organ Class (SOC).

  Treatment-emergent adverse events (TEAEs) are events which start at least one day after V7 (or whose severity worsened after that time).

  An event is considered to be related to the study treatment if the assessment of imputability is likely or possible, or if the assessment is missing.

  In the CRF adverse events occurring with different intensity, frequency, seriousness, or action taken could be documented as separate episodes. For the analysis:
  - In case the first episode of the adverse event started on or after the first intake of study medication or the last episode stopped before the first intake of study medication, the episodes will be collapsed into a single adverse event. While collapsing episodes, the highest

seriousness, the worst severity and all actions taken with respect to the study medication will be retained. The event will be counted as a single event.
  - In case the adverse event started before the first intake of study medication and continued till after the first intake of study medication, and:
    - The severity did not increase on or after the first intake of study medication the episodes will be collapsed into a single adverse event.
    - The severity increased on or after the first intake of study medication, the episodes from the first increase on, on or after the first intake of study medication will be collapsed into a single adverse event and the episodes before the first intake of study medication will be collapsed into another single adverse event.

  When listing the data, the individual episodes will be presented.

- Adverse events of special interest (AESIs)
  As per Risk Management Plan for Pitolisant [Wakix (Pitolisant) EMEA_H_C_2616 PRAC Rapp Updated RMP update AR 2017-05-26.doc], the AESIs are:
  - Anxiety
  - Depression
  - Drug abuse and misuse
  - Drug dependence
  - Fertility disorders
  - Gastric disorders caused by hyperactivity
  - Insomnia
  - Proconvulsive potential
  - QT-interval prolongation
  - Rebound effect
  - Weight increase

  The MedDRA PT of the AESIs will be identified by Bioproject in the database. If pertinent MedDRA PTs may be combined for the analysis.
- Safety Laboratory Data (V1, V6, V12); Values and status (within the normal range, abnormality not clinically significant, clinically significant abnormality, not assessed) for each of the following:
  - Hematology: Red blood cells, hemoglobin, hematocrit, mean corpuscular volume (MCV), white blood cells, 5 differential (neutrophils, lymphocytes, monocytes, eosinophils, basophils) or 3 differential (granulocytes, lymphocytes, monocytes), platelets, coagulation time INR
  - Biochemistry: Blood urea nitrogen (BUN), uric acid, creatinine, creatine phosphokinase (CPK), ALAT/SGPT, ASAT/SGOT, GGT, alkaline phosphatase, total protein, total bilirubin, glucose, total cholesterol, triglycerides
  - Electrolytes: Sodium, potassium, calcium, chloride, bicarbonates/CO_2
  - Urine analysis: Dipstick test assessed as positive or negative (only V1)
  - ß-HCG serum pregnancy test assessed as positive or negative
  - HBsAg, HCV, HIV assessed as positive or negative (only V1)
- Vital signs (V1 to V13):
  - Height (cm) only at V1
  - Weight (kg) and BMI (kg/m^2)
  - Systolic and diastolic blood pressure (mm Hg), and heart rate (bpm)
- Physical Examination (V1 to V13); Status (normal, abnormal, not done) for each of the following body systems:
  - General condition
  - Cardiovascular examination
  - Respiratory examination

  - Abdominal examination
  - Neurological examination
  - Locomotor system examination
  - Dermatologic

  At V2 to V13 any change since the previous visit was also recorded.

- Electrocardiogram (ECG) (V1 to V13):
  - Heart rate (bpm)
  - Sinusal rhythm (no, yes)
  - PR (msec)
  - QRS (msec)
  - QT (msec)
  - QTcF (msec) (QT x $(HR/60)^{1/3}$)
  - QTcB (msec) calculated as QT x $(HR/60)^{1/2}$
  - Result (normal, abnormal)

  The final stable treatment value is defined as the average of the non-missing data at V11 and V12.
  - Additional variables based on QT and QTcF:
    - QT500: Any occurrence of QT post-dose (V8 to V13) > 500 msec (yes, no)
    - QTcF450: Any occurrence of QTcF post-dose (V8 to V13) > 450 msec (yes, no)
    - ΔQTcF ≥ 60 msec (yes, no), with
      ΔQTcF = Max [QTcF post-dose (V8 to V13)] – QTcF pre-dose (V7)
- Beck Depression Inventory (BDI) (V1, V2, V6, V7, V9 to V13):
  - 13 items score
  - 13 items score category: 0-4, 5-7, 8-15, ≥ 16
  - Item G
- Amphetamine-like withdrawal symptoms (Phone contact 2, V7, Phone contact 3, V13) (yes, no) for each of the following symptoms:
  - Dysphoria
  - Fatigue
  - Vivid and unpleasant dreams
  - Insomnia or hypersomnia
  - Increased appetite
  - Psychomotor retardation or agitation

  Amphetamine-like withdrawal syndrome is defined as dysphoria and 2 or more of the other symptoms. In case the assessment of dysphoria is missing or the assessment of more than one of the other symptoms are missing amphetamine-like withdrawal syndrome is considered missing.
- Patient’s Overall Evaluation of Tolerance (V3 for 1st and 2nd week, V4 to V7, V8 for 1st and 2nd week, V9 to V13) (good, moderate, poor)
- Concomitant medications; None (ticked or not), and if not ticked, for each:
  - Generic name
  - Dose and unit
  - Frequency
  - Route
  - Date beginning and date end or ongoing
  - Indication

  Medications will be coded according to the World Health Organization (WHO) drug dictionary, at the level of the Anatomical Therapeutic Chemical (ATC) coding and preferred drug name, taking indication and route into account, if available.

  Concomitant medications are defined as medications ongoing at V7 or started on or after V7.

In case the end date is (partially) missing but there is an indication that the medication was no longer ongoing at V7 or the partial end date clearly contains sufficient information to derive that medication was stopped before V7, the medication will not be considered as concomitant. If on the other hand, it cannot be unambiguously derived that medication was stopped before V7, the medication will be considered as concomitant.

2.4 Course of the Study

The following end of study data are collected on V13:

- Date of first study treatment intake
- Date of last study treatment intake
- Maximum dose administered
- End of study date
- Did the patient complete the study (yes, no), and if no reason(s) for discontinuation:
    - Reasons for discontinuation:
        - Non-eligible patient and specification
        - Discontinuation by the investigator and specification
        - Adverse event and specification
        - Double-blind broken and specification
        - Lost to follow-up and specification
        - Severe depression (BDI-13≥16 or item G>0) and specification
        - ECG Fridericia corrected QT interval > 450 and specification
        - Voluntary withdrawal of patient consent and specification
        - Major protocol violation or non-compliance and specification
        - Prohibited treatment intake and specification
        - Patient changed position towards nCPAP therapy and specification
        - Other reason and specification

Based on these data and on study dates the following variables are calculated:

- Date of end of open label phase: The last of the dates of (V6 to V13, Phone contact 2, last treatment intake)
- Open label phase duration = Date of end of open label phase – Date of V7
- Study duration = Date of end of open label phase – Date of V1

2.5 Exposure, Treatment Dose, and Compliance

The following data are considered (the numbers of tablets refer to 20 mg tablets):

- V8
    - Real number of returned unused tablets for Weeks 14 and 15
    - Dose prescribed for Week 16 (mg) (low=10 mg, medium=20 mg, high=40 mg)
- V9
    - Real number of returned unused tablets for Week 16
    - Dose prescribed for Weeks 17 to 28 (mg per day)
- V10
    - Real number of returned unused tablets for Weeks 17 to 28
    - Dose prescribed for Weeks 29 to 40 (mg per day)
- V11
    - Real number of returned unused tablets for Weeks 29 to 40

  - Dose prescribed for Weeks 41 to 52 (mg per day)
- V12:
  - Real number of returned unused tablets for Weeks 41 to 52

Based on these data and the end of study data the following additional variables are calculated:

- Maximum dose prescribed = Maximum (20 mg, dose prescribed at V8 to V11)
- Final stable dose = Dose prescribed at V11
- Compliance between V7 and V8 (W14 and W15)
  - Number of tablets prescribed:
    20 tablets of 5 mg for Week 1
    10 tablets of 20 mg for Week 2
    Number of tablets taken:
    (20 – number of tablets of 5 mg returned) for Week 1
    (10 – number of tablets of 20 mg returned) for Week 2
  - Dose taken:
    Number of tablets taken W1/W14 x 5 mg + Number of tablets taken W2/W15 x 20 mg
  - Dose to be taken:
    - If Date of V3 – Date of V2 > 7:
      10 mg x 7 + [(Date of V3 – Date of V2) – 7] x 20 mg
    - If Date of V3 – Date of V2 <= 7:
      (Date of V3 – Date of V2) x 10 mg
  - Compliance (%) = 100 x Dose taken / Dose to be taken
- Compliance during the periods V8-V9 (W16), V9-V10 (W17 to W28), V10-V11 (W29 to W40), V11-V12 (W41 to W52):
  - Number of tablets dispensed: Collected in CRF
  - Number of tablets taken = Number of tablets dispensed – sum of numbers of tablets returned
  - Number of daily doses taken:
    If 10 mg/d was prescribed: Number of tablets taken/2
    If 20 mg/d was prescribed: Number of tablets taken
    If 40 mg/d was prescribed: Number of tablets taken/2
  - Number of daily doses to be taken = Date of next visit – date of visit
  - Compliance (%) = 100 x number of daily doses taken / number of daily doses to be taken
- Overall compliance =
  100 x total number of doses taken / total number of doses to be taken

NOTE: For patients who did not complete the open label phase of the study, overall compliance is calculated with the total number of doses taken and the total number of doses to be taken until the last visit before study interruption.

NOTE: For patients were the compliance was missing and had to be recalculated by Balkan Trials as decided in the DRM (BPJ123_DRM_Report_OL_1_0) the overall compliance will be calculated as a weighted average of compliance calculated for each period using weights based on the duration of each period.

- Exposure to open label treatment (day) = Date of last study treatment intake – Date of V7

3 Statistical Considerations

3.1 Analysis Sets

The following analysis sets are considered:

Open Label Full Analysis Set (OL-FAS): All randomized patients who participated in the open label phase of the study. The statistical analyses are based on the treatment to which the patient was randomized.

Open Label Safety Population (OL-SAF): All patients who received at least one dose of open label study medication, and for whom at least one valid post-V7 evaluation (including any AE) is available. The statistical analyses are based on the treatment delivered to the patient at V2.

Other selections will be mentioned when needed in some analyses.

3.2 Missing Data

Missing data will be imputed only for the primary endpoint ESS and only for inferential purposes. The imputation will be performed as described in Section 2.2.

3.3 Summary Statistics

For all the studied endpoints, unless otherwise specified, descriptive results will be based on the OL-FAS, reported for visits without data imputation and presented for all patients, split by DB treatment group and split by DB treatment group stratified for use of nCPAP.

Unless specified otherwise, the following parameters will be used to summarize the data:

- Quantitative variables: mean, 95% confidence interval (CI) on the mean, standard deviation (SD), minimum, quartiles, maximum, number of available and number of missing observations.
- Ordinal, nominal variables: number and percentage for each of the scores or categories, and the number of observations.

3.4 Inferential Statistical Analysis

To evaluate maintenance of efficacy during the open label phase of the study descriptive statistics will be combined with simple statistical tests for patients in the OL-FAS that were treated with BF2.649 and placebo during the DB phase. For continuous data the t-test will be used and for categorical data the chi-square test.

Furthermore, for some of the endpoints, the significance of the difference between the IMP compared with placebo will be assessed through a general model of analysis of Covariance (ANCOVA) in which the studied endpoint at final time (noted Y_f) constitutes the dependent variable of the model. Independent variables considered as fixed effects are the baseline value of the studied endpoint (noted Y_b), the treatment effect (Noted Trt) coded 0 (placebo) or 1 (IMP) and the effect of use of nCPAP (0,1) from baseline. The center will be considered as a random factor of the intercept constituted by a fixed part k_0 and a random component $N(0,\sigma_c)$. This Model can be expressed as follows:

$$Y_{if} = (k_0+\zeta) + k_b\, Y_b + k_t\, Trt + k_c\, CPAP + \epsilon_i \qquad \epsilon_i = N(0,\sigma\ ) \text{ and } \zeta = N(0,\sigma_c)$$

For this model also LOCF/BOCF imputation will be used as described in section 3.4. Finally, unless otherwise specified, the significance test will be conducted at a two-sided significance level of .05.

Notes

1- This model does not assume a treatment-baseline interaction term, as required (CPMP, 2003). Our decision of using center as a random factor is justified by the useless estimate of each center, in focusing on the measurement of the standard deviation of the intercept. The treatment was considered as a fixed factor without center random component.

2- This model is a Linear Mixed Model (LMM) as it combines random and fixed factors, assumes linearity of baseline and hypothesizes the studied endpoint distributed according to a normal (Gaussian) distribution.

3.5 Accounting for Multiple Centers

This study is multicenter where treatment was dispensed to be balanced between centers, however, the randomization block size was 4, thus an asymmetric distribution is possible, particularly within small centers. There is no a priori reason to combine individual centers. The statistical analysis will account for possibility of unbalance between centers and treatment, in considering a random factor for center (mixed model including random center factor for ANCOVA).

3.6 Multiple Testing

All statistical tests for data from the open label phase will be performed two-sided, at the 5% level of significance. No corrections will be performed for multiplicity.

3.7 Miscellaneous

- All the summary tables provided in this report are expected to be automatically used in the Clinical Study Report. These tables will be provided as RTF /HTML/ WinWord compatible format (and not as text format). These tables can originate from SAS outputs: these outputs will be provided in appendix and a reference to these tables will be provided in the legend of the summary table.
- The used software will be SAS V9.4 release.
- Unless otherwise specified, the significance level will be fixed at 0.05 and all statistical tests will be conducted two-sided.

3.8 Changes from the Analyses Foreseen in the Protocol

- To be consistent with the analysis of previous studies it was decided also to perform an analysis of the EQ-5D data based on a composite z-score of the descriptive system and VAS.
- Alternative Inferential model to assess the effect of CPAP: see section 3.4

4 Generalities

4.1 Sample Description

The number of patients will be provided in each of the analysis sets, in each of the analysis sets in each center, and in each of the analysis sets at each visit. A documented list will be given of patients excluded from the analysis.
An analysis will be performed of protocol deviations occurring during the open label phase on the basis of the OL-FAS.

It will consist of:

- Frequency distributions by treatment and by use of nCPAP of patients having at least one major deviation, at least one major deviation by type of deviation, at least one deviation, and at least one deviation by type of deviation
- Documented list of protocol deviations

A protocol deviation was assessed to be major if it could have a direct or indirect effect on the outcome. The final decision as to whether a deviation was considered major or minor will be taken during the Blind Review meeting.

4.2 Onset Data

The analysis of the onset data will be descriptive. No statistical testing will be performed.

The following onset data will be considered:

- Demographic Data
  - Age (year)
  - Age group
  - Sex
  - Professional activity, number of hours/24h, number of days/week, night shift worker
- Medical History
  - Time since diagnosis (month)
- Baseline values
  - ESS at V2
  - OSLER at V2
  - CGI-S at V2
  - Pichot Fatigue scale at V2

4.3 Course of the Study

Date of first and last inclusion visit and date of last visit will be given for the entire OL-FAS and broken down by study center.

Descriptive statistics will be given for study duration and open label phase duration, and frequency distributions of study withdrawal and their reasons for the entire OL-FAS and OL-SAF Population, and broken down by treatment administered during the double-blind phase. Descriptive statistics of study duration will also be provided for the DB-FAS. No statistical testing will be performed.

4.4 Data lists

- For all patients of the DB-FAS: patient number, DB treatment, DB completer Y/N, OL-FAS Y/N, OL-PP Y/N
- For DB completers not in OL-FAS: patient number, DB treatment, reason not in OL-FAS

- For patients in OL-FAS but not in OL-PP: patient number, DB treatment, reason not in OL-PP, date V1, date V7, date violation (if major violation)/date discontinuation
- All protocol violations of patients in OL-FAS: patient number, DB treatment, date V1, date V7, date violation
- For all patients of the OL-FAS: patient number, DB treatment, date V1, date V7, did the patient discontinue the OL, and if yes, date and reason

4.5 Figure

A Consort/PRISMA Diagram Tree will visualize the follow up of the patients by differentiating between treatment groups.

5 Efficacy Analysis

5.1 ESS

5.1.1 Summary Statistics

ESS observed values will be reported at each visit for all patients, by treatment and by treatment, stratified by use of nCPAP. Furthermore, OLF-LOCF/BOCF ESS will be reported as well as OLF-LOCF/BOCF ESS – V7 (baseline open label), OLF-LOCF/BOCF ESS - V2 (baseline double blind), and OLF-LOCF/BOCF ESS – V6 and percentage change with respect to V7 and V2.

Data will be imputed only for OLF as defined in section 2.2

In an additional summary table (section 10.1) the mean, standard deviation and number of observations will be provided for the split based on the use of nCPAP. In the 'all patients' column, values will be reported as mean and 95%CI, and in the last column the difference between the two groups will be reported also with 95%CI. The summary table will also describe the analysis of the wash-out periods V7-V6 and V13-V12.

5.1.2 Analysis

The main efficacy variable OLF defined as the final ESS at one year for the two treatment groups will be tested with a t-test for variables OLF-LOCF/BOCF ESS as well as OLF-LOCF/BOCF ESS – V7 (baseline open label) and OLF-LOCF/BOCF ESS – V2 (baseline double blind). Furthermore, OLF-LOCF/BOCF ESS will be assessed using the ANCOVA model as described in section 3.4 by using Visit V2 as baseline adjustment.

5.1.3 Graphics

Graphs will be created showing the observed values at each visit for patients in both treatment groups as well as the marginal least squares mean estimates from the ANCOVA model. These graphs will be prepared for all patients in OL-FAS as well as split by the use of nCPAP.

5.2 Therapy Response

5.2.1 Description

Frequency distributions will be provided for both responses R_1 and R_2.

In an additional summary table (section 10.2) the observed proportions will be provided by treatment group for all patients as percentage and 95%CI. Furthermore, the data will also be split based on the use of nCPAP.

5.2.2 Inference

Therapy response (no/yes) at final OL time will be assessed using a nonlinear logistic mixed model. Odds Ratios (from logistic inference) and Risk Ratio (from Quasi Poisson inference) will be provided. Baseline V2 used as adjustment.

In the summary table, the difference between both treatment groups for all patients will be assessed by means of a chi-square test. The corresponding p-value will be shown in the table.

5.3 Sleep Diary

5.3.1 Summary Statistics

Descriptive statistics will be provided for SDWD, SDNS, SDDS, and SDAD for each period, for the difference between the baseline period (V6-V7) and the final stable treatment period (V11-V12), and for the difference between the baseline period (V6-V7) and the final period (V12-V13)

5.3.2 Analysis

No inferential analysis will be provided for these endpoints.

5.4 OSleR test

5.4.1 Summary Statistics

OSL at V2, V6, V7, and V12, V12/V2, and V12/V7 will be summarized by the geometric mean, minimum, 1st quartile, median, 3rd quartile, and maximum. Frequency distributions and exact 95% CIs will be given for OSLC at V2, V6, V7, and V12.

In an additional summary table (section 10.3) the OSLER score will be provided by means of the geometric mean and number of patients for each treatment group and for all patients as well as split based on the use of nCPAP. Within the whole group of patients, also the ratio between the two treatment groups will be reported with 95%CI.

5.4.2 Inference

The following analyses will only be conducted if the number of OSLER values collected at the last visit (V12) exceeds 75% of the sample size.

OSL

The significance of the treatment effect on the log-transformed Log(OSL V12) adjusted for its baseline Log(OSL V7) will be assessed according to the model described in section 3.4. The results will be expressed as anti-logs of parameters and CI for clinical interpretation.

In the additional summary table (section 10.3) the difference between both treatment groups will be assessed by means of a t-test based on geometric means. The corresponding p-value will be added to the last column.

OSLC

In the summary table, the difference between both treatment groups for all patients will be assessed by means of a chi-square test. The corresponding p-value will be shown in the table.

5.5 Quality of life test Euro Qol-5D (EQ-5D)

5.5.1 Summary Statistics

Frequency distributions will be provided for EQ5Di (i=1,5) at V2, V6, V7, and V12.

Descriptive statistics will be provided for EQ5DS, EQ5DVAS, and EQ5DZ at V2, V6, V7, and V12, and for the difference between V2 and V12, and between V7 and V12. The table will be presented for these endpoints exactly as the ESS table in limiting visits to V2, V6, V7 and V12 and with the same calculation.

5.5.2 Analysis

No inferential statistics will be conducted for this variable.

5.6 Leeds Sleep Evaluation Questionnaire (LSEQ)

5.6.1 Summary Statistics

Descriptive statistics will be provided for GTS, QOS, AFS, and BFW at the reported visits and for the difference between V2 and V12, and between V7 and V12.

An additional summary table will be presented for the 4 endpoints (see section 10.5).

5.6.2 Analysis

No inferential statistics will be provided.

5.7 Trail Making Test (TMT) Parts A&B

5.7.1 Summary Statistics

Descriptive statistics will be provided for TMTA and TMTB at V2, V6, V7, and V12, and for the difference between V2 and V12, and between V7 and V12.

An additional summary table will be presented for TMTA and TMTB (see section 10.5).

5.7.2 Analysis

No inferential statistics will be provided.

5.8 Clinical Global Impression (CGI)

5.8.1 Summary Statistics

Frequency distributions will be provided for

- CGI-S at V1 and V2
- CGI-C at V6, V7, V10, V11, V12, and V13
- CGI-C improvement at V6, V7, V10, V11, V12, and V13.

In an additional summary table (see section 10.4) % and 95% CI will be provided for CGI-S, CGI-C at V12, and CGI-C improvement at V12 and V13, by treatment group, for all patients and broken down by nCPAP use, as well as the difference between treatment groups.

5.8.2 Inference

In the summary table, the two treatment groups will be compared at V12 and V13 based on a chi-square test and the corresponding p-value will be reported.

5.9 Patient's Global Opinion (PGOE)

5.9.1 Summary Statistics

Frequency distributions will be provided of PGOE and frequency distributions and exact 95% CIs for PGOE improvement, for each assessment time (V6, Phone contact 2, V7, V10, V11, V12, Phone contact 3, V13). An additional summary table similar to the one for CGI will be provided (see section 10.4).

5.9.2 Analysis

In the summary table, the 2 treatment groups will be compared at V12 based on a chi-square test and the corresponding p-value will be reported.

5.10 Pichot Fatigue Scale

5.10.1 Summary Statistics

Observed values for Fatigue scale will be reported at each visit for all patients, by treatment, stratified by use of nCPAP. Furthermore, PFS at V12, PFS V12 – PFS V7 (baseline open label) and PFS V12 – PFS V2 (baseline double blind) will also be provided.

In an additional summary table (similar to the table of section 10.1) the mean, standard deviation and number of observations will be provided for the split based on the use of nCPAP. In the 'all patients' column, values will be reported as mean and 95%CI, and in the last column the difference between the two groups will be reported with 95%CI.

5.10.2 Analysis

The difference between the two treatment groups will be tested with a t-test for PFS at V12, PFS V12 – V7 (baseline open label) and PFS V12 – V2 (baseline double blind). Furthermore, PFS at V12 will be assessed using the ANCOVA model as described in section 3.4 by using Visit V2 as baseline adjustment.

5.11 Stable Activity

5.11.1 Summary Statistics

Frequency distributions and exact 95% CIs will be given for maintaining stable activity at each assessment time (Phone contact 1, V2, V3, V6 to V13).

5.11.2 Analysis

No inferential statistics will be provided.

5.12 Aggregate z-score of ESS and OSleR

5.12.1 Summary Statistics

Descriptive statistics will be provided for the aggregate z-score at V2, V6, V7, and V12, and for the difference between V2 and V12, and between V7 and V12. An additional summary table will be presented for the aggregate z-score (see Section 10.5).

5.12.2 Analysis

The following analysis will only be conducted if the number of OSLER values collected at the last visit (V12) exceeds 75% of the sample size.

The general model will be applied for the aggregate z-score at V12.

6 Safety Analysis

The analysis of safety will be descriptive except for vital signs.

6.1 Adverse Events

The analysis of AEs will consist of a systematic analysis of all TEAEs, derived from a spontaneous and assisted list of AEs, and an analysis of treatment-emergent adverse events of special interest. It will consist of:

- Frequency distributions of patients with TEAEs, with TEAESIs, with related TEAEs, with treatment-emergent serious adverse events (SAEs), and with TEAEs causing premature study discontinuation.
- Frequency distribution of patients with TEAEs and with related TEAEs by:
    - MedDRA SOC*
    - MedDRA SOC and preferred term (PT)*
    - MedDRA SOC, PT, and maximal intensity
    - MedDRA SOC, PT, and sex
    - MedDRA SOC, PT, and age group

  * These distributions will also mention the number of events
- Number of events and frequency distribution of patients with TEAESIs by:
    - Types of MedDRA PTs
    - Types of MedDRA PTs and maximal intensity

6.2 Laboratory evaluations

The analysis will consist of:

- Descriptive statistics for hematology, biochemistry and electrolytes variables at each assessment time.
- Descriptive statistics for the change between V1 and V12 and between V6 and V12.
- Frequency distributions for the status of each hematology, biochemistry and electrolytes variable (within the normal range, abnormality not clinically significant, abnormality probably due to study compound, abnormality probably due to current or concomitant disease, abnormality probably due to study compound and concomitant disease), at each assessment time.
- Frequency distributions for the status of each variable at V12 broken down by the status at V1 and the status at V12 broken down by the status at V6.

6.3 Vital Signs

Descriptive statistics will be provided for weight, BMI, blood pressure, and heart rate at each assessment time and for the difference between V2 and V8 to V13, and between V7 and V8 to V13.

The difference between the two both treatment groups will be calculated for each visit together with corresponding 95% confidence interval and p-value based on the t-test for difference.

A graphical representation will be provided for the mean systolic blood pressure at each visit.

6.4 Physical examination

Frequency distributions will be provided for each body system (normal, abnormal) at each assessment time.

6.5 ECG Data

6.5.1 Summary Statistics

Descriptive statistics will be provided for heart rate, PR, QRS, QT, QTcB, and QTcF at each assessment time, baseline (average of V1 and V2), and for the stable treatment period (average of V11 and V12). Descriptive statistics will also be provided for the difference in heart rate, PR, QRS, QT, QTcB, and QTcF, between baseline and V8 to V13, between baseline and the stable treatment period, between V7 and V8 to V13, and between V7 and the stable treatment period.

Frequency distributions will be provided for sinusal rhythm and overall result, at each assessment time, and for QT500, QTcF450, and ΔQTcF.

6.5.2 Data List

A data list will document individual values for any patient presenting with an outlier ECG value at any post dose time point (V8 to V13). The list will mention the patient number, treatment, age, gender, and all recordings of PR, QRS, QT, QTcB, and QTcF.

Patients will be selected if ΔQTcF = yes or if for any post-dose (V8 to V13) recording:
PR > 220 or QRS > 120 or QT > 500 or QTcB > 450 or QTcF > 450

6.6 Beck Depression Inventory (BDI)

Descriptive statistics will be provided for the 13 items score at V1, V2, V6, V7, and V9 to V13, and for the difference between V2 and V9 to V13, and between V7 and V9 to V13. Frequency distributions will be given for the 13 items score category and for Item G at V1, V2, V6, V7, and V9 to V13.

6.7 Amphetamine-like withdrawal symptoms

Frequency distributions will be provided for each symptom (yes, no) and for amphetamine-like withdrawal syndrome (yes, no) at each assessment time (Phone contact 2, V7, Phone contact 3, V13).

6.8 Patient's Overall Evaluation of Tolerance

Frequency distributions will be provided for each assessment time (V3 for 1st and 2nd week, V4 to V7, V8 for 1st and 2nd week, V9 to V13).

6.9 Previous and Concomitant Treatment

Frequency distributions will be provided of subjects using any concomitant medication, and subjects using any concomitant medication by Anatomical class, and by Anatomical and Therapeutic class.

6.10 Exposure, Dosing, and Compliance

Frequency distributions and descriptive statistics will be given for dose prescribed at V8, V9, V10, and V11 (final stable dose), and maximum dose. Descriptive statistics will be given for exposure to open label treatment, compliance during the periods V7-V8, V8-V9, V9-V10, V10-V11, and V11-V12, and overall compliance.

Furthermore, a comparison will be made between the stable doses of the double-blind period (V5) and open label period (V11). Number of patients receiving 10mg, 20mg or 40mg will be provided broken down by the stable dose (10mg, 20mg or 40mg) during the double-blind period.

7 Statistical Tables

7.1 Demography and Baseline Characteristics

All summary tables and listings in this section will be conducted on the OL-FAS.

7.1.1 Demography

Table 14.1.1.1	Age – Descriptive Statistics
Table 14.1.1.2	Age group – Frequency distribution
Table 14.1.1.3	Sex – Frequency distribution
Table 14.1.1.4	Professional activity – Frequency distribution
Table 14.1.1.5	Number of hours/24h, Number of days/week – Descriptive statistics
Table 14.1.1.6	Night shift worker – Frequency distribution

7.1.2 Medical History

Table 14.1.2.1	Time since diagnosis – Descriptive statistics

7.1.3 Baseline Data

Table 14.1.3.1	ESS at V2 – Descriptive statistics
Table 14.1.3.2	OSler at V2 – Descriptive statistics
Table 14.1.3.3	CGI-S at V2 – Descriptive statistics
Table 14.1.3.4	Pichot Fatigue Scale at V2 – Descriptive statistics

7.2 Efficacy

All tables and listings in this section will be conducted on the OL-FAS population. Tables for the OL-PP Population, tables and listings will only be shown in case the difference between OL-FAS and OL-PP exceeds 5%.

7.2.1 Efficacy Variables Based on the ESS (Except Response)

Table 14.2.1.1.1	Epworth Sleepiness Scale (ESS) at each assessment time – Descriptive statistics
Table 14.2.1.1.2	Final ESS (OLF-LOCF/BOCF) – Descriptive statistics + t-test
Table 14.2.1.1.3	Change between final ESS (OLF-LOCF/BOCF) and ESS at V7 (Final – V7) – Descriptive statistics + t-test
Table 14.2.1.1.4	Change between final ESS (OLF-LOCF/BOCF) and ESS at V2 (Final – V2) – Descriptive statistics + t-test
Table 14.2.1.1.5	Change between final ESS (OLF-LOCF/BOCF) and ESS at V6 (Final – V6) – Descriptive statistics + t-test
Table 14.2.1.1.6	Percentage change between final ESS (OLF-LOCF/BOCF) and ESS at V7 – Descriptive statistics + t-test
Table 14.2.1.1.7	Percentage change between final ESS (OLF-LOCF/BOCF) and ESS at V2 – Descriptive statistics + t-test
Table 14.2.1.1.8	Analysis of (OLF-LOCF/BOCF) – General model
Table 14.2.1.1.9	Additional summary table

7.2.2 Other Efficacy Variables

7.2.2.1 Response

Table 14.2.1.2.1.1	Response (R_1 and R_2) – Frequency distribution
Table 14.2.1.2.1.2	Additional summary table for R_1 + Chi-square test
Table 14.2.1.2.1.3	Additional summary table for R_2 + Chi-square test
Table 14.2.1.2.1.4	Analysis for R_1 – General model (logistic)
Table 14.2.1.2.1.5	Analysis for R_2 – General model (logistic)

7.2.2.2 Sleep Diary

Table 14.2.1.2.2.1	SDWD, SDAD, SDNS, and SDDS at each assessment period – Descriptive statistics
Table 14.2.1.2.2.2	For SDWD, SDAD, SDNS, and SDDS, difference between the baseline (V6-V7) and the final stable treatment period (V11-V12) – Descriptive statistics
Table 14.2.1.2.2.3	For SDWD, SDAD, SDNS, and SDDS, difference between the baseline (V6-V7) and the final period (V12-V13) – Descriptive statistics

7.2.2.3 OSleR test

Table 14.2.1.2.3.1	OSL at V2, V6, V7, and V12 – Summary statistics (geometric means)
Table 14.2.1.2.3.2	OSL at V12/V7 and V12/V2 – Summary statistics (geometric means)
Table 14.2.1.2.3.3	Additional summary table for OSler – Summary statistics + t-test (geometric means)
Table 14.2.1.2.3.4	Difference between log(OSL) at V12 and V7 – general model
Table 14.2.1.2.3.5	OSLC at V2, V6, V7, and V12 – Frequency distribution and exact 95% CI

7.2.2.4 EQ-5D

Table 14.2.1.2.4.1	EQ5Di (i=1,5) at V2, V6, V7, and V12 – Frequency distribution
Table 14.2.1.2.4.2	EQ5DS, EQ5DVAS, and EQ5DZ at V2, V6, V7, and V12 – Descriptive statistics
Table 14.2.1.2.4.3	For EQ5DS, EQ5DVAS, and EQ5DZ, difference between V12 and V7 – Descriptive statistics
Table 14.2.1.2.4.4	For EQ5DS, EQ5DVAS, and EQ5DZ, difference between V12 and V2 – Descriptive statistics

7.2.2.5 LSEQ

Table 14.2.1.2.5.1	GTS, QOS, AFS, and BFW at V2, V6, V7, and V12 – Descriptive statistics
Table 14.2.1.2.5.2	For GTS, QOS, AFS, and BFW, difference between V12 and V7 – Descriptive statistics
Table 14.2.1.2.5.3	For GTS, QOS, AFS, and BFW, difference between V12 and V2 – Descriptive statistics
Table 14.2.1.2.5.4	Additional summary table

7.2.2.6 TMT

Table 14.2.1.2.6.1	TMTA and TMTB at V2, V6, V7, and V12 – Descriptive statistics
Table 14.2.1.2.6.2	For the difference of TMTA and TMTB between V12 and V7 – Descriptive statistics
Table 14.2.1.2.6.3	For the difference of TMTA and TMTB between V12 and V2 – Descriptive statistics
Table 14.2.1.2.6.4	Additional summary table

7.2.2.7 CGI

Table 14.2.1.2.7.1	CGI-S at V1 and V2 – Frequency distribution
Table 14.2.1.2.7.2	CGI-C at V6, V7, V10, V11, V12 and V13 – Frequency distribution
Table 14.2.1.2.7.3	CGI-C improvement at V6, V7, V10, V11, V12 and V13 – Frequency distribution
Table 14.2.1.2.7.4	Additional summary table – Descriptive statistics + Chi-square test

7.2.2.8 PGOE

Table 14.2.1.2.8.1	PGOE at V6, Phone contact 2, V7, V10, V11, V12, Phone contact 3, and V13 – Frequency distribution
Table 14.2.1.2.8.2	PGOE improvement at V6, Phone contact 2, V7, V10, V11, V12, Phone contact 3, and V13 – Frequency distribution and exact 95% CI
Table 14.2.1.2.8.3	Additional summary table for PGOE improvement – frequency distribution + Chi-square test

7.2.2.9 Pichot Fatigue Scale

Table 14.2.1.2.9.1	Pichot Fatigue Scale score at V2, V6, V7, and V9, V10, V11, V12 and V13,
Table 14.2.1.2.9.2	Difference between V12 and V7 – Descriptive statistics
Table 14.2.1.2.9.3	Difference between V12 and V7 – Descriptive statistics
Table 14.2.1.2.9.4	Additional summary table for PFS – Descriptive statistics
Table 14.2.1.2.9.5	Pichot Fatigue Scale score at V12 – General model
Table 14.2.1.2.9.6	Difference between V12 and V7 – General model
Table 14.2.1.2.9.7	Difference between V12 and V2 – General model

7.2.2.10 Stable Activity

Table 14.2.1.2.10.1	Maintaining stable activity at Phone contact 1, and V2, V3, V6 to V13 – Frequency distribution and exact 95% CI

7.2.2.11 Aggregate z-score of ESS and OSleR

Table 14.2.1.2.11.1	Aggregate z-score at V2, V6, V7, and V12 – Descriptive statistics
Table 14.2.1.2.11.2	Difference between Aggregate z-score at V12 and at V7 – Descriptive statistics
Table 14.2.1.2.11.3	Difference between Aggregate z-score at V12 and at V2 – Descriptive statistics
Table 14.2.1.2.11.4	Additional summary table
Table 14.2.1.2.11.5	Analysis of the Aggregate z-score at V12 – General model

7.3 Safety Evaluation

All summary tables in this section will be conducted on the OL-SAF Population.

7.3.1 <u>Adverse Events</u>

Table 14.3.1.1	Patients with TEAEs, TEAEs of special interest, related TEAEs, treatment-emergent SAEs, and with TEAEs causing premature study discontinuation – Frequency distribution
Table 14.3.1.2	Patients with TEAEs by MedDRA SOC and PT – Frequency distribution
Table 14.3.1.3	Patients with TEAEs by MedDRA SOC, PT, and maximal intensity – Frequency distribution
Table 14.3.1.4	Patients with TEAEs by MedDRA SOC, PT, and sex – Frequency distribution
Table 14.3.1.5	Patients with TEAEs by MedDRA SOC, PT, and age group – Frequency distribution

Table 14.3.1.6	Patients with related TEAEs by MedDRA SOC and PT – Frequency distribution
Table 14.3.1.7	Patients with related TEAEs by MedDRA SOC, PT, and maximal intensity – Frequency distribution
Table 14.3.1.8	Patients with related TEAEs by MedDRA SOC, PT, and sex – Frequency distribution
Table 14.3.1.9	Patients with related TEAEs by MedDRA SOC, PT, and age group – Frequency distribution
Table 14.3.1.10	Patients with TEAESIs by MedDRA PT – Frequency distribution and number of events
Table 14.3.1.11	Patients with TEAESIs by MedDRA PT and maximal intensity – Frequency distribution and number of events

7.3.2 <u>Laboratory evaluations</u>

Table 14.3.2.1	Hematology variables at each assessment time and change between V1 and V12 and between V6 and V12 – Descriptive statistics
Table 14.3.2.2	Hematology: Status of each variable at each assessment time – Frequency distribution
Table 14.3.2.3	Hematology: Status of each variable at V12 broken down by the status at V1 and status at V12 broken down by the status at V6 – Frequency distribution
Table 14.3.2.4	Biochemistry variables at each assessment time and change between V1 and V12 and between V6 and V12 – Descriptive statistics
Table 14.3.2.5	Biochemistry: Status of each variable at each assessment time – Frequency distribution
Table 14.3.2.6	Biochemistry: Status of each variable at V12 broken down by the status at V1 and status at V12 broken down by the status at V6 – Frequency distribution
Table 14.3.2.7	Electrolyte variables at each assessment time and change between V1 and V12 and between V6 and V12 – Descriptive statistics
Table 14.3.2.8	Electrolytes: Status of each variable at each assessment time – Frequency distribution
Table 14.3.2.9	Electrolytes: Status of each variable at V12 broken down by the status at V1 and status at V12 broken down by the status at V6 – Frequency distribution

7.3.3 <u>Vital Signs</u>

Table 14.3.3.1	Weight, BMI, blood pressure, and heart rate at each assessment time, and change between V2 and V8 to V13 and between V7 and V8 to V13 – Descriptive statistics + t-test

7.3.4 <u>Physical examination</u>

Table 14.3.4.1	For each body system, at each assessment time – Frequency distribution

7.3.5 <u>ECG Data</u>

Table 14.3.5.1	Heart rate, PR, QRS, QT, QTcB, and QTcF at each assessment time, at baseline (average of V1 and V 2), for the stable treatment period (average of V11 and V12), and change from baseline and from V7 – Descriptive statistics
Table 14.3.5.2	Sinusal rhythm and ECG overall result at each assessment time – Frequency distribution

Table 14.3.5.3 QT500, QTcF450, and ΔQTcF – Frequency distribution

7.3.6 BDI

Table 14.3.6.1 BDI 13 items score at V1, V2, V6, V7, and V9 to V13, and change between V2 and V9 to V13 and between V7 and V9 to V13 – Descriptive statistics
Table 14.3.6.2 BDI 13 items score category at V1, V2, V6, V7, and V9 to V13 – Frequency distribution
Table 14.3.6.3 BDI Item G at G at V1, V2, V6, V7, and V9 to V13 – Frequency distribution

7.3.7 Amphetamine-like Withdrawal Symptoms

Table 14.3.7.1 For each symptom at each assessment time – Frequency distribution
Table 14.3.7.2 Amphetamine-like withdrawal syndrome at each assessment time – Frequency distribution

7.3.8 Patient's Overall Evaluation of Tolerance

Table 14.3.8.1 Patient's overall evaluation of tolerance at each assessment time – Frequency distribution

7.4 Concomitant Medications

All summary tables in this section will be conducted on the OL-SAF Population.
Table 14.4.1 Use of any concomitant medication – Frequency distribution
Table 14.4.2 Use of any concomitant medication by Anatomical class– Frequency distribution
Table 14.4.3 Use of any concomitant medication by Anatomical and Therapeutic class – Frequency distribution

7.5 Exposure, Dosing, and Compliance

All summary tables in this section will be conducted on the OL-SAF Population.
Table 14.5.1 Exposure to open label treatment – Descriptive Statistics
Table 14.5.2 Dose prescribed at V8, V9, V10, and V11 (final stable dose), and maximum dose – Frequency distribution
Table 14.5.3 Repartition of Doses – Frequency distribution
Table 14.5.4 Compliance during the periods V7-V8, V8-V9, V9-V10, V10-V11, and V11-V12, and overall compliance – Descriptive Statistics

7.6 Sample Description

- Patient Disposition
  Table 14.6.1 Number of patients in each of the analysis sets
  Table 14.6.2 Number of patients in each of the analysis sets in each center
  Table 14.6.3 Number of patients in each of the analysis sets at each visit
- Protocol Deviations (OL-FAS)
  Table 14.6.4 Patients having at least one major deviation - Frequency distribution
  Table 14.6.5 Patients having at least one major deviation by type of deviation - Frequency distribution
  Table 14.6.6 Patients having at least one deviation - Frequency distribution

Table 14.6.7 Patients having at least one deviation by type of deviation - Frequency distribution

7.7 *Course of the Study*

- Study Dates

  Table 14.7.1 For the entire OL-FAS, and broken down by center, date of first and last inclusion visit and date of last visit

- Study Duration and withdrawal

  For the OL-FAS and the OL-SAF set, broken down by treatment administered during the double-blind phase:

  Table 14.7.2 Study duration – Descriptive Statistics

  Table 14.7.3 Open label phase duration – Descriptive Statistics

  Table 14.7.4 Study withdrawal - Frequency distribution

  Table 14.7.5 Reasons for study withdrawal - Frequency distribution

8 Listings of Individual Data

16.2.1	Discontinued Patients
16.2.2	Protocol Deviations
16.2.3	Patients Excluded From the Analysis Sets
16.2.4	Demography and Other Baseline Data
16.2.4.1	Demography
16.2.4.2	Time Since Diagnosis
16.2.5	Study Treatment
16.2.5.1	Study Treatment Administration
16.2.5.2	Compliance
16.2.6	Efficacy Data
16.2.6.1	ESS
16.2.6.2	Sleep Diary
16.2.6.3	OSLeR Test
16.2.6.4	Quality of Life (EQ-5D)
16.2.6.5	Other Secondary Efficacy Variables
16.2.7	Adverse Events
16.2.7.1	Treatment Emergent Adverse Events
16.2.7.2	Deaths and Serious Adverse Events
16.2.7.3	Adverse Events Leading to Study Drug Interruption or Discontinuation
16.2.8	Laboratory Data
16.2.8.1	Laboratory Data – Hematology
16.2.8.2	Laboratory Data – Biochemistry
16.2.8.3	Laboratory Data – Electrolytes
16.2.8.4	Laboratory Data – Urinalysis
16.2.8.5	Laboratory Data – ß-HCG serum pregnancy test, HBsAg, HCV, HIV
16.2.9	Vital Signs
16.2.10	Physical Examination
16.2.11	ECG
16.2.11.1	ECG – Recorded Data
16.2.11.2	ECG – Outliers
16.2.12	Beck Depression Inventory
16.2.13	Amphetamine-like withdrawal symptoms and patient's overall evaluation of tolerance
16.2.14	Prior and Concomitant Medications

Whenever applicable, listings will present calculated variables. For quantitative assessments for which a change from baseline is described in the statistical tables, the change from baseline will also be listed.

If it is required to present much information on listings, listings may be presented in several parts, in order to enhance readability.

9 Overview of Assessments

Double-Blind period

Period	V1 D -14	1 Week Wash-out period	Phone cont. 1 D -7	1 Week	V2 D 0	W 1	W 2	V3 D 14	W 3	V4 D 21	W 4 → W 7	V5 D 49	W 8 → W 12	V6 D 84	W 13	Phone cont. 2 V6 +3D	V7 [6] End of study D 91
Visit[1]						10 mg	20 mg		10 mg or 20 mg or 40 mg		Selected dose (4 weeks)		Selected dose (5 weeks)		1 week wash-out period		
Signature of Consent Form	X																
Medical questionnaire	X		X		X			X		X		X		X		X	X
Physical examination	X				X			X		X		X		X			X
ESS	X				X			X		X		X		X			X
Polysomnography[2]				X													
OSleR test[3]					X									X			
MMSE	X																
TMT parts A & B					X									X			
CGI-S	X				X												
CGI-C														X			X
BDI-13	X				X									X			X
LSEQ, EQ-5D					X									X			
Pichot Fatigue Scale					X									X			X
Patient's global opinion on the effect of investigational drugs														X		X	X
Patient's overall evaluation of the tolerance								X		X		X		X			X
Amphetamine-like withdrawal symptoms questionnaire																X	X
ECG	X				X			X		X		X		X			X
Safety biology[4]	X													X			
Delivery of sleep diary	X				X			X		X		X		X			
Review of sleep diary[5]					X			X		X		X		X			X
Adverse events			X		X			X		X		X		X		X	X

1 – Each visit shall be carried out at the end of the relevant time period ± 3 days.
2 – Overnight polysomnography performed from 22:00 until 7:00 (minimum 8 hours of recording) in the sleep laboratory only between V1 and V2 except if available in the previous 12 months.
3 – OSleR test: 3 sequences at 2 hours interval (at 9:00, 11:00, and 13:00).
4 – Biological examination: Hematology (hemoglobin, hematocrit, red and white blood cell count (with differential), platelets, mean corpuscular volume, coagulation time (INR)), biochemistry (blood urea nitrogen (BUN), uric acid, creatinine, creatine kinase, ALAT, ASAT, GGT, alkaline phosphatases, total protein, total bilirubin, glucose, electrolytes (sodium, potassium, calcium, chloride, bicarbonates/CO_2), total cholesterol, triglycerides), serology (HIV, HCV, HBsAg at V1, β-HCG (woman of child-bearing potential)), urianalysis: stick (with microscopy and bacteriological culture, if positive) at V1.
5 – At each visit the patient shall bring back the sleep diary and return the unused drug. Patient will be contacted in advance before each visit to remind him/her to fill in the diary.
6 – Only for patients who are not entering into Open Label Extension period; for others see Open Label Extension period study diagram.

Open Label Extension period

**Period**		**W 14**	**W 15**		**W 16**		**W 17 → W 28**		**W 29 → W 40**		**W 41 → W 52**		**W 53**	**Phone cont. 3**	**V13**
**Visit**[1]	**V7**[2] D 91	10 mg	20 mg	**V8** D 105	10 mg or 20 mg or 40 mg	**V9** D 112	10 mg or 20 mg or 40 mg	**V10** D 196	10 mg or 20 mg or 40 mg	**V11** D 280	10 mg or 20 mg or 40 mg	**V12** D 364	1 week Wash-out period	V12 +3D	End of study D 371
Signature of Consent Form	X														
Medical questionnaire	X			X		X		X		X		X		X	X
Physical examination	X			X		X		X		X		X			X
ESS	X			X		X		X		X		X			X
OSleR test[3]	X											X*			
TMT parts A & B	X											X			
CGI-C	X							X		X		X			X
BDI-13	X					X		X		X		X			X
LSEQ, EQ-5D	X											X			
Pichot Fatigue Scale	X					X		X		X		X			X
Patient's global opinion on the effect of investigational drugs	X							X		X		X		X	X
Patient's overall evaluation of the tolerance	X			X		X		X		X		X			X
Amphetamine-like withdrawal symptoms questionnaire	X													X	X
ECG	X			X		X		X		X		X			X
Safety biology[4]												X			
Delivery of sleep diary	X			X		X		X		X		X			
Review of sleep diary[5]	X			X		X		X		X		X			X
Adverse events	X			X		X		X		X		X		X	X
Pharmacokinetics sampling[6]								X							

1 – Each visit shall be carried out at the end of the relevant time period ± 3 days.
2 – Only for patients who are entering into Open Label Extension period; for others see Double Blind period study diagram.
3 – OSleR test: 3 sequences at 2 hours interval (at 9:00, 11:00 and 13:00). Optional at V12.
4 – Complete biological examination: hematology (hemoglobin, hematocrit, red and white blood cell count (with differential), platelets, mean corpuscular volume, coagulation time (INR)), biochemistry (blood urea nitrogen (BUN), uric acid, creatinine, creatine kinase, ALAT, ASAT, GGT, alkaline phosphatases, total protein, total bilirubin, glucose, electrolytes (sodium, potassium, calcium, chloride, bicarbonates/CO_2), total cholesterol, triglycerides), serology (β-HCG (for woman with child-bearing potential)).
5 – At each visit, the patient shall bring back his sleep diary. Patient will be contacted in advance before each visit to remind him/her to fill in the sleep diary. The patient shall return the unused drug at each visit except at V13.
6 – for all patients.

10 Overview of additional summary tables

10.1 Summary table 1: Primary efficacy variable ESS – Continuous analysis

		nCPAP		No nCPAP		All Patients		
		Placebo	IMP	Placebo	IMP	Placebo	IMP	Diff
DB	V1	X ± SD (n)	X ± SD (n)	X ± SD (n)	X ± SD (n)	X [U,L]	X [U,L]	X [U,L]
	…	X ± SD (n)	X ± SD (n)	X ± SD (n)	X ± SD (n)	X [U,L]	X [U,L]	X [U,L]
	V6	X ± SD (n)	X ± SD (n)	X ± SD (n)	X ± SD (n)	X [U,L]	X [U,L]	X [U,L]
End DB	V(6+7)-V2	X ± SD (n)	X ± SD (n)	X ± SD (n)	X ± SD (n)	X [U,L]	X [U,L]	X [U,L]
Washout	Diff (V7-V6)	X ± SD (n)	X ± SD (n)	X ± SD (n)	X ± SD (n)	X [U,L]	X [U,L]	X [U,L]
OL visits	V7	X ± SD (n)	X ± SD (n)	X ± SD (n)	X ± SD (n)	X [U,L]	X [U,L]	X [U,L]
	…	X ± SD (n)	X ± SD (n)	X ± SD (n)	X ± SD (n)	X [U,L]	X [U,L]	X [U,L]
	V13	X ± SD (n)	X ± SD (n)	X ± SD (n)	X ± SD (n)	X [U,L]	X [U,L]	X [U,L]
Final OL	OL(V12)	X ± SD (n)	X ± SD (n)	X ± SD (n)	X ± SD (n)	X [U,L]	X [U,L]	X [U,L]
	OL(V12)-OL(V7)	X ± SD (n)	X ± SD (n)	X ± SD (n)	X ± SD (n)	X [U,L]	X [U,L]	X [U,L]
	OL(V12)-Base (V2)	X ± SD (n)	X ± SD (n)	X ± SD (n)	X ± SD (n)	X [U,L]	X [U,L]	X [U,L]
Washout	OL(V13)-OL(V12)	X ± SD (n)	X ± SD (n)	X ± SD (n)	X ± SD (n)	X [U,L]	X [U,L]	X [U,L]
Sustained efficacy	OL(V12)-OL(V6)	X ± SD (n)	X ± SD (n)	X ± SD (n)	X ± SD (n)	X [U,L]	X [U,L]	X [U,L]

10.2 Summary table 2: Efficacy variable ESS – Responder analysis

	No CPAP		CPAP		All Patients		
	Placebo	IMP	Placebo	IMP	Placebo	IMP	Diff
R1	X% (n)	X% (n)	X% (n)	X% (n)	% [U,L]	% [U,L]	X [U,L] P
R2	X% (n)	X% (n)	X% (n)	X% (n)	% [U,L]	% [U,L]	X [U,L] P

10.3 Summary table 3: Efficacy variable OSler

	nCPAP		No nCPAP		All Patients		
	Placebo	IMP	Placebo	IMP	Placebo	IMP	Ratio
Baseline	GM (n)	GM (n)	GM (n)	GM (n)	GM [U,L]	GM[U,L]	R [U,L]
V6	GM (n)	GM (n)	GM (n)	GM (n)	GM [U,L]	GM[U,L]	R [U,L]
V12	GM (n)	GM (n)	GM (n)	GM (n)	GM [U,L]	GM[U,L]	R [U,L], P
V12/V6	GM (n)	GM (n)	GM (n)	GM (n)	GM [U,L]	GM[U,L]	R [U,L], P
V12/V2	GM (n)	GM (n)	GM (n)	GM (n)	GM [U,L]	GM[U,L]	R [U,L], P

10.4 Summary table 4: Efficacy variable CGI

	nCPAP		No nCPAP		All Patients		
	Placebo	IMP	Placebo	IMP	placebo	IMP	Difference
CGI-S (V1) 0	X% (n)	X% (n)	X% (n)	X% (n)	X [U,L]	X [U,L]	
1	X% (n)	X% (n)	X% (n)	X% (n)	X [U,L]	X [U,L]	
…	X% (n)	X% (n)	X% (n)	X% (n)	X [U,L]	X [U,L]	
CGI-S (V2) 0	X% (n)	X% (n)	X% (n)	X% (n)	X [U,L]	X [U,L]	
1	X% (n)	X% (n)	X% (n)	X% (n)	X [U,L]	X [U,L]	
…	X% (n)	X% (n)	X% (n)	X% (n)	X [U,L]	X [U,L]	
CGI-C (V12) 0	X% (n)	X% (n)	X% (n)	X% (n)	X [U,L]	X [U,L]	
1	X% (n)	X% (n)	X% (n)	X% (n)	X [U,L]	X [U,L]	
2	X% (n)	X% (n)	X% (n)	X% (n)	X [U,L]	X [U,L]	
3	X% (n)	X% (n)	X% (n)	X% (n)	X [U,L]	X [U,L]	
4	X% (n)	X% (n)	X% (n)	X% (n)	X [U,L]	X [U,L]	
5	X% (n)	X% (n)	X% (n)	X% (n)	X [U,L]	X [U,L]	
6	X% (n)	X% (n)	X% (n)	X% (n)	X [U,L]	X [U,L]	
CGI-C imp. V12	X% (n)	X% (n)	X% (n)	X% (n)	X [U,L]	X [U,L]	p
V13	X% (n)	X% (n)	X% (n)	X% (n)	X [U,L]	X [U,L]	p

10.5 Summary table 5: Efficacy variables LSEQ, TMT and Aggregate z-score

		nCPAP		No nCPAP		All Patients		
		Placebo	IMP	Placebo	IMP	Placebo	IMP	Diff
DB visits	V2	X ± SD (n)	X ± SD (n)	X ± SD (n)	X ± SD (n)	X [U,L]	X [U,L]	X [U,L]
	V6	X ± SD (n)	X ± SD (n)	X ± SD (n)	X ± SD (n)	X [U,L]	X [U,L]	X [U,L]
OL visits	V7	X ± SD (n)	X ± SD (n)	X ± SD (n)	X ± SD (n)	X [U,L]	X [U,L]	X [U,L]
	V12	X ± SD (n)	X ± SD (n)	X ± SD (n)	X ± SD (n)	X [U,L]	X [U,L]	X [U,L]
Final OL	V12 – V7	X ± SD (n)	X ± SD (n)	X ± SD (n)	X ± SD (n)	X [U,L]	X [U,L]	X [U,L]
	V12 – V2	X ± SD (n)	X ± SD (n)	X ± SD (n)	X ± SD (n)	X [U,L]	X [U,L]	X [U,L]

11 Detailed Version History

Section	Brief description of change	Reason for change
2.1	Removal of definitions of risk factors and risk categories	Definitions were suggested by PL (in document SAP-H3-OL.docx dated 15/2/2021) but he did not specify their analysis (also not foreseen in protocol)
2.1	Removal of definition and analysis of dose repartition	This was also suggested by PL. The analysis has been moved to section 6.10.
2.2	For missing ESS data the following changes were made: LOCF is used if drop-out reason unrelated to treatment (instead of related). BOCF is used if drop-out reason related to treatment (instead of unrelated).	This was wrong in the previous version. It is in line with the suggestion of PL (section 3.7). The definition of LOCF (last of V7 to V10) could be added. BOCF could also be specified but this has no impact on the analysis since the reason for drop-out is always unrelated to treatment.
2.2	For ESS, details were added on the way it is decided whether a drop-out is related to study treatment.	Done for the benefit of the programmers and to be in line with the analysis of the double blind phase.
2.2	Definitions of the variables based on ESS have been added.	Also necessary for the programmers.
2.3	Provision for missing or partial dates of start of adverse events was removed.	These do not occur.
2.3	A provision was added for missing or partially missing dates of concomitant medications.	Necessary for some medications to decide whether they should be considered to be concomitant.
2.4	In the formula for the open-label phase duration, V6 was replaced by V7.	Wrong in the previous version.
2.5	Small changes were made to the definitions of compliance.	It appeared that the data recorded in the database were not identical to the annotated CRF.
2.5	A note was added how to calculate overall compliance in case the data were missing.	Due to the fact that the CRO in charge of monitoring (Balkan trials), did some recalculations and entered the results in the database.
3.1	The definition of the OL-SAF analysis set was adapted to only include patients with at least one valid post-V7 evaluation (instead of V6).	Wrong in the previous version.
3.2	The explanation of the use of LOCF/BOCF for missing ESS was removed and a reference was made to section 2.2.	
3.4	In note 2 the reference to model 1 was removed.	In document SAP-H3-OL.docx (of 15/2/2021) PL suggested to use 3 models for the analysis of most efficacy varibales. In document SAP-H3-OL-PLHT.docx he specified to consider the model with factors treatment and nCPAP (but no interaction) as the main model. This was later

		changed to a single model (as agreed by PL in the attachment of a mail on 21/3/2021). The reference to model 1 could therefore be removed.
3.8	The change with respect to the protocol concerning considering models with site as random effect and as fixed effect was removed.	Due to the large number of sites with few patients it was decided to remove the analysis with site as fixed effect.
3.8	The change with respect to the protocol concerning performing the analyis of time to somnolence in the Osler test was removed.	It is mentioned in section 5.4.2 that the inferential analysis is only to be done in case the number of OSLER values collected at the last visit (V12) exceeds 75% of the sample size. Since this was not the case the analysis was not performed and no change is to be mentioned.
4.5	The sentence concerning starting the PRISMA diagram from the beginning of the DB phase and showing the patients not participating to the OL was removed.	The diagram is only to concern the OL phase.
5.1	OLF was replaced by OLF-LOCF/BOCF ESS.	Clarification of the final value.
5.1.1	Summary statistics of change from baseline and percentage change have been added.	Not specified in the previous version.
5.1.1	Summary statistics of the 2 wash-out periods (V6-V7 and V12-V13) were added to the additional summary table requested by PL (in document SAP-H3-OL.docx dated 15/2/2021).	This was stated by PL as one of the objectives (in document SAP-H3-OL-PLHT.docx dated 7/3/2021).
5.2	Definition of response removed.	Is given in section 2.2.
5.2.2	Specification that model 2 is to be used was removed.	Only one model is used (see line 14).
5.4.2	Baseline and final OSL specified as V7 and V12.	Clarification.
5.4.2	It is specified that OSLC will be compared in the 2 treatment groups by means of a chi-square test.	In the original SAP it was foreseen that a McNemar test would be used. This was changed to be consistent with other analyses of binary variables (i.e. response).
5.5	It is specified that the additional summary table will be similar to the one for ESS.	Clarification for the programmers.
5.6.1, 5.7.1	It is specified that there will be a summary table.	To be consistent with the analyses of other variables.
5.8.1	The variables and summary statistics of the summary table are specified.	Clarification for the programmers.
5.8.2	It is added that the summary table will show the result of comparing the 2 groups at V13.	Suggested as an additional evaluation for a variable for which V13 is available.
5.9.1	It is specified that there will be a summary table.	Oversight in the previous version.
5.11.2	It is specified that no inferential analysis will be performed.	To be consistent with other not important secondary variables.

5.12.2	It is specified that the model will analyse the aggregate score at V12 (instead of the difference between V7 and V12).	To be in line with the analysis of ESS and Osler.
6.1	The stable dose periods for the 2 phases of the study are specified (V5 and V11).	Clarification.
7, 8, 10	Various changes	Changes were made in order to improve consistency with the other sections.

DEVIATIONS FOR PLANNED ANALYSIS FOR STUDY

HAROSA III – DOUBLE BLIND

PROJECT NAME/NUMBER (BPJ13)

Version History

Version	Date	Author	Job Title	Status
1.0	20 October 2021	[redacted]	Statistician	Final
2.0	14 January 2022	[redacted]	Statistician	Final
3.0	17 February 2022	[redacted]	Statistician	Final

Approvals

Clinical Project Manager:

[redacted]
Bioprojet Pharma
9, rue Rameau
F-75002 Paris
FRANCE

[redacted] Feb 17, 2022
Signature Date

Head of Biostatistics:

Dr. [redacted]
Data Investigation Company Europe
16 ‘t Hoveld
B-1082 Brussels
BELGIUM

[redacted] Feb 18, 2022
Signature Date

1. PURPOSE

The purpose of this document is to provide justification for deviations in the statistical analysis with respect to the double-blind phase of study P1513 (EUDRACT NUMBER: 2015-004561-85). More precisely, this document relates to the following documents:

- SAP: BPJ13_SAP_DB_1_0.pdf
- TFL: BPJ13_DB_TFL_T.pdf (Dated 14 Jan 2022)
- Statistical output: BPJ13_DB_TFL_S.pdf (dated 14 Jan 2022)
- TFL: BPJ13_DB_TFL_L1.pdf (Dated 16 Sep 2021)
- TFL: BPJ13_DB_TFL_L2.pdf (Dated 16 Sep 2021)

The information provided in this document can serve as input for the CSR section 9.8 'Changes in the Conduct of the Study or Planned Analyses'.

2. SCOPE

This document applies to the following subsections of the TFL for the Double-Blind period.

- 14.3.4.2 Proportion of Abnormal Values for each Body System at V6 - Logistic Regression
- 14.3.6.5 BDI 13 Items Score at V6 - Mixed Model
- 14.3.6.6 BDI 13 Items Score Category at V6 - Ordinal Logistic Regression
- Overall Compliance
- Missing data and BOCF imputation
- Models with Center as fixed effect

Full justification of the analysis is provided in following sections.

3. PHYSICAL EXAMINATION (PE, 14.3.4)

In this section, an event is defined as an abnormal result in a subject's physical examination. Consequently, a non-event is defined as a normal result.
The SAP foresees that for each body system abnormal PE results will be modelled with a logistic regression model adjusting for baseline, age, gender, treatment and center. The eventual model deviated from the SAP in tree aspects

- Only the model for the General Status Body System was considered because of the negligible prevalence of events for other body systems at V6, with a maximum of 2 events in the Cardiovascular body system. Since logistic regression models the probability of an event occurring, a prerequisite of such a model is a reasonable number of events. By rule of thumb, a minimum of ten events is acceptable. By any standard or rule of thumb, two or fewer events are unacceptable to the extent of being statistically impossible to model.
- A mixed logistic regression with a random intercept for center was used rather than a single-level model with center as a covariate due to the issue of **complete separation**. If a subgroup of the model consists solely of events or non-events, the logistic regression model yields erratic estimates and standard errors. This complete separation issue was the case for center as a covariate: some centers only reported normal physical examinations of General Status, which impacted the estimate, standard error and consequently p-value and confidence interval. For this reason, a mixed model was considered with center as a random effect rather than a fixed effect – which estimates the intercept variance rather than the effects of separate center dummy variables.
- It was stated ion the SAP that the model would also adjust for baseline. This was not performed since the baseline values are systematically identical and as such do not contribute to the model. There is a footnote stating 'The model does not adjust for the Baseline Physical Examination Value, because this value is systematically identical to the V6 value for General Status'

Furthermore, the analysis was only performed for V6 as was done for laboratory evaluations while for vital signs the analysis was performed for all visits V3 – V7. The reason for this is that the main interest is ate the end of the double-blind period (V6)

4. BECK'S DEPRESSION INVENTORY (BDI, 14.3.6)

4.1. Mixed Model for Numeric BDI (14.3.6.5)

The linear mixed model for BDI modelled log (BDI + 1) rather than BDI itself. To provide meaningful estimates, the Least Square Means were transformed back by means of exp (result) – 1. It must be taken into account that, while the Least Squares Estimates were transformed back in order to optimize interpretability, the difference in Least Square Means bears no substantive meaning and was therefore omitted. These changes in the original BDI variable were made due to (i). violation of the normality assumption and (ii). a non-positive definite Hessian matrix.

Violation of the normality assumption has the same consequences for the model as in a single-level-regression: if the observation-level residuals of the model are not normally distributed, the p-value and confidence interval width may be biased. As can be seen in figure 1, the residuals of the untransformed BDI scale are rather heavy-tailed (mainly towards high outliers), suggesting that there is a substantial number of unexplained extreme values in terms of depression. A traditional solution for right-skewed data and residuals is a logarithmic transformation of the original scale. However, this transformation is not possible since the BDI scale can take zero values. Therefore, the natural logarithm was taken of the original scale + 1 to circumvent this issue. As can be seen in figure 2, this transformation considerably reduced the heaviness of the tails.

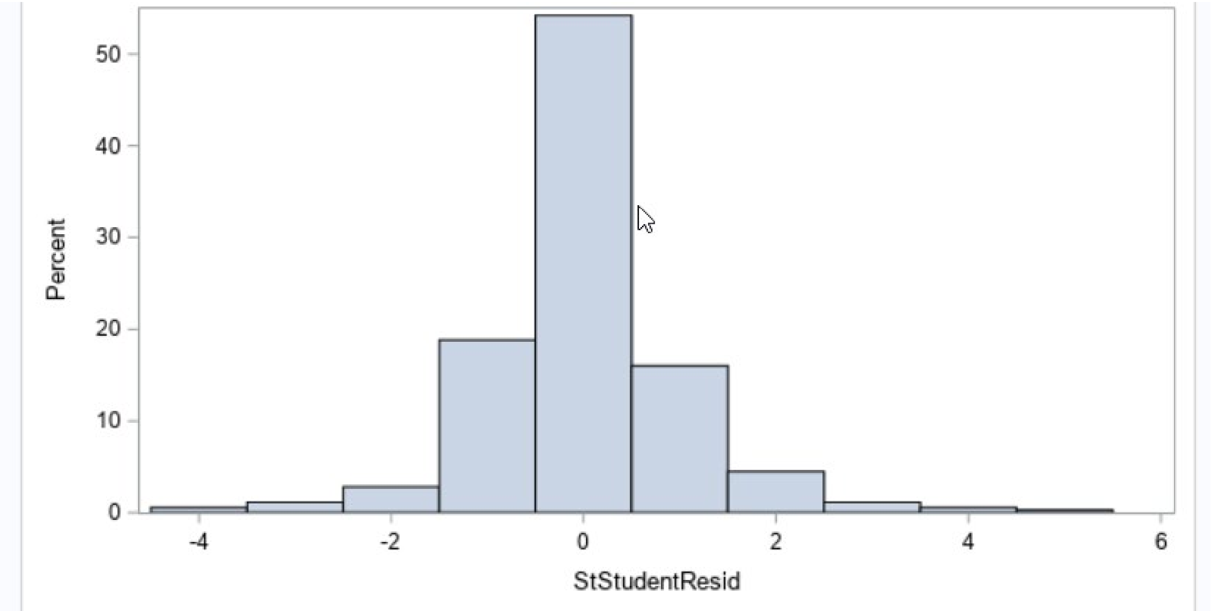

Figure 1: Histogram of studentized residuals of original BDI scale

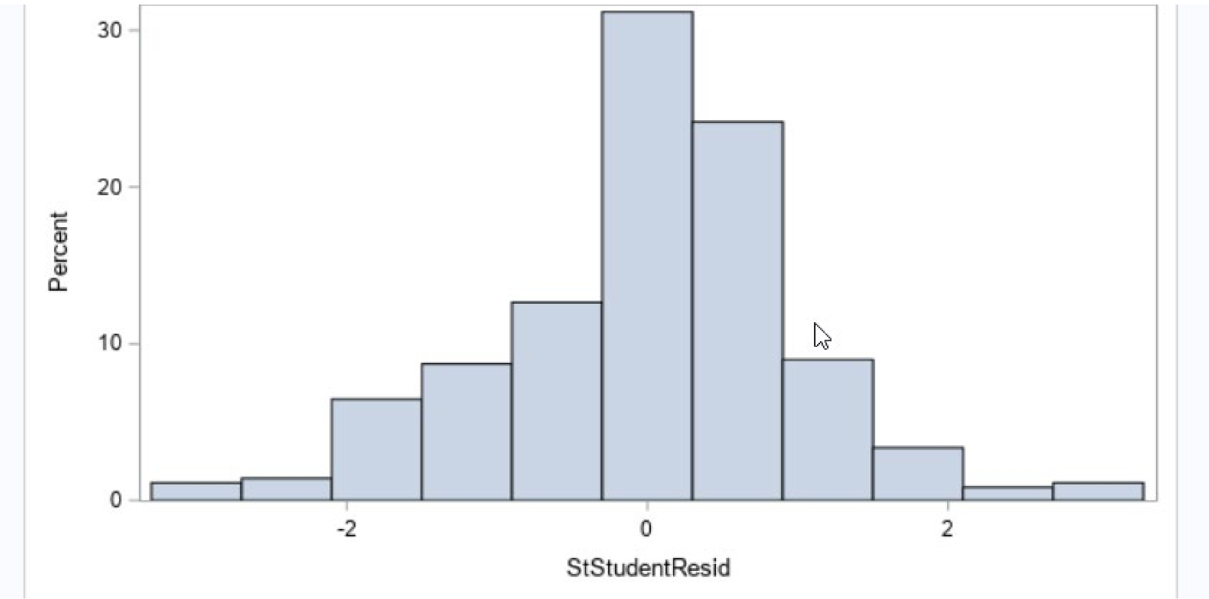

Figure 2: Histogram of studentized residuals of log-transformed BDI scale

While a violation of the normality assumption does not render a model invalid per se and the violation was not overly severe in this model, the original model also did have a non-positive definite Hessian matrix. This Hessian matrix issue is more problematic, since it indicates that the model cannot guarantee stable or optimal effect size estimates. After the aforementioned transformation, the model did not give any warning related to the Hessian matrix.

4.2. Ordinal Regression Model for Ordinal BDI

For the categorical/ordinal BDI score (No/Mild/Moderate/Severe Depression), the numeric BDI scale at baseline was used as a covariate, rather than dummies for the categorical BDI at baseline. The rationale behind this modelling choice was similar to the complete separation issue for physical examination: modelling the probability of having a higher degree of depression becomes biased if this degree of depression is constant for all individuals within a specific subgroup of the baseline BDI categories. In addition, the original BDI scale contains more information and there is no reason to assume a polynomial relation between baseline BDI and the probability to be more depressed at V6.

5. OVERALL COMPLIANCE (14.5.3)

The SAP originally defined separate compliance formulae for the period between V2-V3 and all subsequent periods:

- V2-V3: Compliance (%) = 100 * Dose taken / Dose to be taken (1)
- Subsequent periods: Compliance (%) = 100 * number of daily doses taken / number of daily doses to be taken (2)

The main (subtle) difference between both methods is that method (1) aggregates the doses to level of the entire period, rather than considering them on a daily basis: the initial number of tablets given to the subject is compared to the number of tablets returned. For reasons of accuracy and reliability, the aggregation method is generalized throughout all periods. The differences in output with the old method are negligible, and all inferential conclusions remain identical.

6. MISSING DATA

For endpoints OSler, Aggregate z-score and Clinical global impression (CGI) a BOCF imputation strategy was proposed. As described in section 3.5 of the SAP however, BOCF imputation is considered an alternative imputation strategy for patients terminating the trial early for a reason suspected to be related to treatment. However, in this trial there were no patients terminating the trial early for a reason that could be related to the treatment and for this reason BOCF imputation was not performed.

7. CENTER AS FIXED EFFECT

The SAP provides inconsistent instructions with respect to fitting models with center as fixed effect and models with center as random effect. More precisely, section 3.7 explicitly mentions that the center is a random factor and there is no model proposed using center as a fixed factor. In section 3.11 however there is an inconsistency in stating that both models should be compared. Based on section 3.7 and the fact that models with center as fixed effect were not estimated this comparison cannot be made.

DEVIATIONS FOR PLANNED ANALYSIS FOR STUDY

HAROSA III – OPEN LABEL

PROJECT NAME/NUMBER (BPJ13)

Version History

Version	Date	Author	Job Title	Status
1.0	14 January 2022	[redacted]	Statistician	Final
2.0	17 February 2022	[redacted]	Statistician	Final

Approvals

Clinical Project Manager	[redacted]<br>Bioprojet Pharma<br>9, rue Rameau<br>F-75002 Paris<br>FRANCE	[redacted]<br>Signature	Feb 17, 2022<br>Date
Head of Biostatistics:	Dr. [redacted]<br>Data Investigation<br>Company Europe<br>16, 't Hofveld<br>B-1082 Brussels<br>BELGIUM	[redacted]<br>Signature	Feb 18, 2022<br>Date

1. PURPOSE

The purpose of this document is to provide justification for deviations in the statistical analysis with respect to the SAP of the open label phase of study P1513 (EUDRACT NUMBER: 2015-004561-85). More precisely, this concerns the following documents

- SAP: BPJ13_SAP_OL_3_0.pdf
- TFL: BPJ13_OL_TFL_T.pdf (Dated 14 Jan 2022)
- TFL: BPJ13_OL_TFL_S.pdf (Dated 14 Jan 2022)
- TFL: BPJ13_OL_TFL_F.pdf (Dated 14 Jan 2022)

The information provided in this document can serve as input for the CSR section 9.8 'Changes in the Conduct of the Study or Planned Analyses'.

2. SCOPE

This document applies to the following subsections of the TFL for the Open Label period.

- Section 14.2.1.2.9: Pichot Fatigue Scale
- 14.2.1.1.8 Analysis of (OLF-LOCF) - General Model
- 14.5.4 Compliance During the Periods V7-V8, V8-V9, V9-V10, V10-V11, and V11-V12, and Overall Compliance - Descriptive Statistics By Treatment Group
- Adverse events: additional information provided not foreseen in the SAP
- Laboratory evaluations: Abnormal observations not further characterised as foreseen in the SAP

Full justification of the analysis is provided in the following sections.

3. PICHOT FATIGUE SCALE

With respect to the Pichot Fatigue Scale, it was planned in the SAP (Section 5.10.2) to perform a t-test for the differences between both treatment arms at visits V12, and for the change versus baseline endpoints V12-V7 and V12-V2. These tests were in the actual analysis replaced by an ANCOVA since this approach provides a more accurate insight for inference with respect to significant differences.

4. GENERAL MODEL FOR ESS

The general model was used and fit for all patients including the factor indicating if the patients were using nCPAP or not. The results are presented in table 14.2.1.1.8. In addition, the same model was fit separately to the subgroup of patient using nCPAP and the subgroup of patients not using nCPAP.
The reason for this additional set of models was to calculate the estimates needed to prepare the graphical representation of the average profile.

5. COMPLIANCE

The calculation of the overall compliance was adapted since there was an inconsistency in the description provided in the SAP. The SAP states the following
"Overall compliance = 100 x total number of doses taken / total number of doses to be taken"
But for some patients/visits the compliance over a certain period was recalculated and provided as such in the database. For those values it was impossible to calculate overall compliance as described in the SAP since the data that were available were not consistent with the recalculated compliance.
For this reason, and in order to be consistent for all patients the overall compliance was calculated based on the periodic compliance either based on the available data or on the recalculated values, using a weighted approach taking into account the timeframe.

6. ADVERSE EVENTS

In tables 14.3.1.2, 14.3.1.6 and 14.3.1.10 information was added with respect to mWnAE, duration and incidence, that was not foreseen in the SAP for the open label part. This information was added to the tables for consistency with the analysis for the double-blind period.

7. LABORATORY EVALUATIONS

The 6 tables in section 14.3.2 describing the status of laboratory evaluations do not mention the data for 'abnormal probably due to study compound' and 'abnormal probably due to current or concomitant medication', that are foreseen in the SAP.
These data were not available in the DB as was signalled by DICE prior to DB lock and approved by sponsor. Therefore, it was not possible to make any further distinction.

16.1.9.2 Population Pharmacokinetic Analysis Plan

The population pharmacokinetic analysis plan, version 1.0 dated 31/10/2019 is provided below.

POPULATION PK ANALYSIS PLAN

Population pharmacokinetic analysis of pitolisant in study:

"Efficacy and Safety of Pitolisant (BF2.649) in the Treatment of Excessive Daytime Sleepiness in Patients with Obstructive Sleep Apnoea Syndrome, Treated or Not by Nasal Continuous Positive Airway Pressure, but Still Complaining of Excessive Daytime Sleepiness"

**Protocol number:**	P15-13
**Drug name:**	Pitolisant (BF2.649)
**Drug development phase:**	Phase III
**Sponsor:**	Bioprojet Pharma<br>9, rue Rameau<br>75002 - Paris<br>France
**Pharmacometrician:**	[redacted]<br>PhinC Development<br>36, rue Victor Basch<br>91300 Massy<br>Phone: [redacted]
**Pharmacometrician:**	[redacted]<br>(same address as above)<br>Phone: [redacted]
**PhinC reference:**	PH15075
**Version - date:**	Version 1.0 - 31/10/2019

This study was conducted in full compliance with applicable Good Clinical Practices and regulations, including archiving.
This document is confidential and the property of Bioprojet Pharma - The information contained within may not be used, divulged, published, reproduced or otherwise disseminated without the approval of Bioprojet Pharma.

2 SIGNATURE PAGE

I herewith declare that I agree without reservation to the methods described in detail in this docum

**Author:**<br>Name: [redacted]<br>Pharmacometrician, Phinc Development	Date: [redacted]<br>+01'00'
Name: [redacted]<br>Pharmacometrician, Phinc Development	[redacted]<br>15:13:24 +01'00'
**Approval:**<br>Name: [redacted]<br>DMPK – Bioanalysis Head, Bioprojet Biot	[redacted]

3 TABLE OF CONTENTS

1 TITLE PAGE 1
2 SIGNATURE PAGE 2
3 TABLE OF CONTENTS 3
4 LIST OF ABBREVIATIONS AND DEFINITIONS OF TERMS 5
5 INTRODUCTION 7
6 ANALYSIS OBJECTIVE 7
7 STUDY P15-13 (HAROSA III) 7
7.1 Study objectives 7
7.2 Summary of study design 7
7.3 Treatments 8
7.4 Study population 8
7.5 Analytical determination 8
7.6 PK sampling 9
8 STUDY DATA 9
8.1 Justification for the number of subjects 9
8.2 Definition of population 9
8.2.1 Population Analysis Set 9
8.3 Analysis datasets 9
8.3.1 Concentrations 10
8.3.2 Dosing events and design variables 10
8.3.3 Covariates 10
8.3.4 Missing data 11
8.3.4.1 Dependent variable 11
8.3.4.2 Covariate 11
8.3.4.3 Design variables 11
8.3.5 Data cleaning and detection of outliers 11
9 MODEL DEVELOPMENT 12
9.1 Sequence of planned analysis 12
9.2 Structural pharmacokinetic model 13
9.2.1 popPK model of patients pooled with healthy volunteers 13
9.2.2 popPK model only on patients 14

9.3 Population PK model components, model building steps and qualification 14
9.3.1 Error models specification (random effects) 14
9.3.1.1 Models for inter-individual variabilities (IIV) 15
9.3.1.2 Model for residual variability 15
9.3.2 Covariate models.. 16
9.3.3 Statistical model selection 17
9.3.4 Estimation method and model acceptance 18
9.3.5 Models evaluation and goodness of fit... 18

10 EVALUATION OF PREDICTABILITY AND STABILITY OF THE MODEL................................19

11 DISPLAY OF RESULTS AND CONTENTS OF THE REPORT 19

11.1 Description of the data 19

11.2 Base model..20

11.3 Covariate selection and final model 20

11.4 Model evaluation 20

12 DERIVED DATA 20

13 SOFTWARE USED...21

14 REFERENCE LIST...22

4 LIST OF ABBREVIATIONS AND DEFINITIONS OF TERMS

α	Type I error for test statistics
ε	Random effect for residual variability/error, assumed to be distributed according to N(0, σ^2)
η	Random effect for Inter-individual variability, assumed to be distributed according to N(0, ω^2)
θ	Fixed effect parameter (of structural model or covariate)
σ^2	Variance for residual variability
ω^2	Variance for Inter-individual variability
AP	Analysis plan
BLQ	Below limit of quantification
BMI	Body mass index
CI	Confidence interval
CL, CL/F	Clearance, apparent clearance
CV	Coefficient of variation
DV	Dependent variable
EBE	Empirical bayes estimate
EDS	Excessive daytime sleepiness
ESS	Epworth sleepiness scale
EVID	Event Identification
e.g.	*Exempli gratia* (for example)
FO	First order approximation method
FOCE	First order conditional estimation method
GM	Geometric mean
GOF	Goodness of fit
h	Hour(s)
IIV	Inter individual variability
IPRED	Individual prediction
kg	kilogram
LOCF	last observation carried forward
LOQ	Limit of quantification
LRT	Likelihood ratio test
m	meter
Max	Maximum
MDV	Missing dependent variable
mg	Milligram(s)
min	Minute(s)
Min	Minimum
mL	Milliliter(s)
nCPAP	Nasal continuous positive airway pressure
ng	nanogram
NPDE	Normalized predicted distribution error

OFV (or OF)	NONMEM objective function value
OSA	Obstructive sleep apnea
pcVPC	prediction-corrected performance visual checks
PK	Pharmacokinetic
popPK	Population pharmacokinetics
PRED	Population prediction
PWRES	Population weighted residuals
QC	Quality control
SD	Standard deviation
V, V/F	Volume of distribution, apparent volume of distribution
VPC	Visual predictive check
vs.	*versus*
WRES	Weighted residuals

5 INTRODUCTION

The purpose of the analysis plan (AP) is to give details of the methodology and conventions that will be used for the population pharmacokinetic (popPK) analysis to be performed on the data from the Phase III study entitled:

"Efficacy and Safety of Pitolisant (BF2.649) in the Treatment of Excessive Daytime Sleepiness in Patients with Obstructive Sleep Apnoea Syndrome, Treated or Not by Nasal Continuous Positive Airway Pressure, but Still Complaining of Excessive Daytime Sleepiness".

The AP ensures the credibility of all study findings by means of predefined data analysis plan. The *pharmacometrician* prepared the AP with the objective to finalize it before starting the analysis.

6 ANALYSIS OBJECTIVE

The objective of the analysis will be to develop a popPK model for pitolisant administered once daily (in the morning, during breakfast) in patients with moderate to severe obstructive sleep apnea syndrome (OSA) in order to provide estimates of PK parameters and variability in this specific population and assess whether any potential covariates are likely to affect pitolisant PK.

7 STUDY P15-13 (HAROSA III)

7.1 STUDY OBJECTIVES

The first objective of this study is to demonstrate the efficacy and safety of pitolisant hydrochloride given at 10, 20, or 40 mg per day versus (*vs.*) placebo during 12 weeks for the Double Blind period, to treat the excessive daytime sleepiness (EDS) in patients with OSA refusing the nasal continuous positive airway pressure (nCPAP) therapy or treated by nCPAP but still complaining of EDS. The efficacy of pitolisant will be assessed separately in patients treated with nCPAP and in patients without nCPAP use.

The secondary objectives of the study include assessing the long-term tolerance as well as the maintenance of efficacy of pitolisant hydrochloride given at 10, 20 or 40 mg per day during 39 weeks of Open Label Extension period and further investigating the co-variates or co-medications that affect the pharmacokinetics of pitolisant in the target population to allow future comparison to healthy subjects.

7.2 SUMMARY OF STUDY DESIGN

This is a prospective, multicenter, randomized, double blind, phase III study of pitolisant versus (*vs.*) placebo during 12 weeks with, at first, an escalating dose period followed by a treatment at the selected dose.

Then, after one week of single-blind placebo wash-out period, if the patient holds the same position towards nCPAP therapy as before, an Open Label Extension period was proposed.

Patients who do not participate in the Open Label Extension period will have their end of the study visit.

Patients willing to continue the pitolisant treatment administration will be given another information notice together with an informed consent form to be signed. This Open Label Extension period will consist of the

same escalating-dose than in the first part, followed by a selected dose period with the active drug only, until 53 weeks after the treatment beginning. Then, patients will have one week wash-out period prior to the end of study visit.

The study will include about 400 patients. This number will allow having 360 completed treated patients (pitolisant, patients refusing nCPAP therapy = 120; placebo, patients refusing nCPAP therapy = 60, pitolisant, patients treated by nCPAP = 120; placebo, patients treated by nCPAP = 60) sufficient for the results analysis of efficacy during the Double Blind period (12 weeks).

The popPK analysis will be performed before the final database lock but only in patients from Open label period as PK will be measured only in this period. So, no procedure to maintain the blind will be applied.

7.3 Treatments

Pitolisant and placebo were presented in identical tablets according to dosage, *i.e.* pitolisant hydrochloride tablets dosed at 5 mg or 20 mg and matching placebo.

During the Double Blind period, patients will take daily 10 mg (2 tablets of 5 mg) or 20mg (1 tablet of 20 mg) or 40 mg (2 tablets of 20 mg) of pitolisant hydrochloride or placebo. During the Open Label Extension period, patients will take daily 10 mg, 20 mg or 40 mg of pitolisant hydrochloride (*i.e.* 2 tablets of 5 mg or 1 or 2 tablets of 20 mg, respectively).

Administration by oral route, once a day, in the morning, during breakfast, with a glass of water.

7.4 Study population

Patients exhibiting moderate* to severe** OSA, experiencing EDS with Epworth Sleepiness Scale (ESS) score ≥ 12, refusing to be treated by nCPAP or having been submitted to nCPAP therapy for a minimum period of 3 months, and still complaining of EDS.

* Moderate: ≥ 15 to ≤ 30 sleep obstructive related breathing events per hour
** Severe: > 30 sleep obstructive related breathing events per hour

7.5 Analytical determination

Analytical determination of study drug and of its main metabolites was to be carried out by the Bioprojet Biotech, 4 rue du Chesnay Beauregard, BP96205, 35762 Saint Grégoire, France.

Serum levels of pitolisant and of its main metabolites BP2.951, BP1.8054, BP1.9733, BP1.3473, BP1.3484, BP1.8186 and BP1.10556 will be evaluated using validated liquid chromatography coupled to tandem mass spectrometry methods (Bioprojet-Biotech study code B258, B349, B387 and B448, respectively). The limits of quantification are 0.1, 0.1, 1, 1, 5, 5, 1 and 1 ng/mL, respectively.

7.6 PK SAMPLING

The proposed PK sampling scheme was optimized according to a previous knowledge on pitolisant PK and an initial population PK model developed in healthy subjects [1]. Parameters of this initial population model were used together with study constraints (i.e. limitation of the number of PK blood samples and PK follow-up limited to a maximum of 8 h post-dose). The PFIM software was used for sampling time optimization and determination of population sample size for valuable analysis.

Blood samples (4 mL) were collected during Visit 10 (Open-label period) in a sub-group of at least 100 patients at pre-dose, 1.5, 3.0 and 8.0 hours (h) post-dose.

8 STUDY DATA

8.1 JUSTIFICATION FOR THE NUMBER OF SUBJECTS

A determination of the minimum number of patients necessary to generate appropriate popPK parameters was performed with the optimization design and requiring a minimum population of 90 patients according to the method described in Section 7.6.

Moreover, results from exploratory studies on pitolisant allow to estimate the Epworth sleepiness scale (ESS) residual variability and under some conditions presented in Section 13.2 of protocol, the sample size will be 120 and 240 (thus at least 360 patients in total) as this size corresponds to the interaction test requiring the largest sample size. Considering 10% drop out rate, approximately 400 patients will be selected.

8.2 DEFINITION OF POPULATION

8.2.1 Population Analysis Set

The popPK analysis will be performed on the population of subjects treated with pitolisant, for which complete actual (rather than randomised) treatment information is available along with complete sampling information. Placebo subjects will be removed from the analysis population.

8.3 ANALYSIS DATASETS

The analysis dataset will be created and formatted according to NONMEM requirement using SAS®.

The sponsor will provide values of pitolisant serum concentrations measured, time of sampling, dose amount and information about compliance (date and time of dosing).

Moreover, subjects' demographic characteristics and any other data that could contain information valuable for the popPK model (covariates for example) will be provided to PhinC Development.

As far as possible, all data used for the NONMEM analysis dataset will be taken from the monitored clinical database as the popPK analysis will be initiated before the final database lock. The serum concentrations will be loaded from an ASCII file and the data loaded will be checked versus the validated bioanalytical report.

Data conversion to NONMEM structure will be performed using SAS® programming.

NONMEM dataset will be assembled using the actual date and time of all recorded administration and of samples of pitolisant.

A QC will be performed between the monitored clinical database and the final NONMEM dataset. Once controlled the SAS® analysis dataset will be converted into an ASCII file readable by NONMEM and further used for popPK analysis.

8.3.1 Concentrations

All serum concentrations of pitolisant with their actual date and time of sampling will be used for the popPK model.

Theoretical relative sampling times will be included in the dataset for reporting purpose only.

No concentration will be excluded from the analysis unless:

- The sampling date and/or time is missing and cannot be reasonably imputed by its theoretical value
- No dosing information before the sampling is available
- The concentration is clearly identified as an outlier in the bioanalytical report (if available)

8.3.2 Dosing events and design variables

Dosing information just before the PK measurement will be included in the database using the actual date and time of administration as well as the actual amount of drug administered. Dosing events will be entered in NONMEM dataset using the appropriate flag variables with Event Identification (EVID=1) and Missing Dependent Variable (MDV=1), EVID=1 correspond to dose administration and MDV=1 correspond to a missing observation and dose administration.

The compliance starting the Open Label period needs to be check and fully included in the final NONMEM dataset, according to the following condition:

- If the compliance will be between 80 and 120 % in the Open label period, the dosing between 2 administrations will be assumed to be performed at the same time as the first one and repeated each 24 h using the Additional Doses (ADDL) and Inter-dose interval (II) NONMEM variables;
- If the compliance information indicates that the dosing was not complete, all efforts will be made to match the actual compliance.

8.3.3 Covariates

Continuous and categorical covariates may be used through the modelling to investigate their effect on PK parameters.

Categorical covariates will be handled as indicator variable for presence (covariate=1) or absence (covariate=0) for the factor.

Covariates will be fully defined in the data definition file, specifically the coding used for categorical covariates.

8.3.4 Missing data

8.3.4.1 Dependent variable

Some distinction will be made between concentrations non available due to values below the limit of quantification (BLQ) and missing data for any other reason (missing sample, not analyzed sample, premature discontinuation, etc.).

The number of BLQ data will be examined before the analysis. If BLQ samples represents no more than 15% of the total samples and does not appear to be dependant to the treatment group, then BLQ values will be removed from the analysis dataset (M1 method as documented by Beal[2]). If BLQ samples represent more than 15% of the total samples, or appeared to be treatment-dependent, they will be included in the data and modelled as censored observations using (M3 method as documented by Beal[(2)]).

Missing PK concentrations data, for any other reason will not be replaced and will be set to missing in the analysis dataset using the MDV=1.

8.3.4.2 Covariate

For any covariate, continuous or categorical, if more than 15% of the subjects present missing value, the covariate will be omitted from the analysis.

As a general rule for missing baseline covariates, unless considered inappropriate, the population median baseline value for continuous covariates, or the most reported category for categorical covariates will be imputed.

For time varying covariates, if any, data will be imputed within individual using the last observation carried forward (LOCF) method.

8.3.4.3 Design variables

Observations (or subjects) with missing information on amount of drug administered, dosing date (or time) and sampling date (or time) will be excluded unless strong evidence can determine that drug was administered (*e.g.* confirmation by the Investigator that the drug was really administered either in a monitoring report or the data-review report). In that case theoretical date and time of dosing or sampling will be used.

8.3.5 Data cleaning and detection of outliers

Data review will be performed to identify potential errors in the data variables that will be used for the popPK analysis. Errors of this type would include, but not be limited to, missing data, inconsistencies between scheduled PK sampling times and actual sampling times, obvious suspected errors in recording of dates and/or times (resulting in negative estimates of actual times, for example), physiologically unreasonable covariate values, and unit of measurement errors.

All information that might affect the administration, and thus concentration levels of pitolisant, will be scrutinized and could led to the exclusion of subject.

9 MODEL DEVELOPMENT

PopPK parameters will be estimated by non-linear mixed effect modelling using NONMEM. Mixed effects models describe the influence of both fixed effects and random effects. The random effects are typically used to capture the variability which can be split in residual variability (error) and between subject variability.

The population model will be defined by 4 basic components:

1. The structural popPK model components, which defines the PK parameters and describe the serum concentration-time profiles of pitolisant.
2. The inter-individual error model component, which describes the inter-individual variation (IIV) in PK parameters after correction for fixed effects.
3. The residual error model component, which describes the underlying distribution of the error in the measured concentrations.
4. The covariate model component, which describes the influence of fixed effects (i.e., demographic factors) on PK parameters.

9.1 SEQUENCE OF PLANNED ANALYSIS

As data will be collected between pre-dose and 8 h after pitolisant administration, it could be necessary to enrich the model with healthy volunteer's data in order to be able to capture pitolisant terminal phase. Therefore, a sequential approach will be used to assess if the healthy volunteers could be combined with patient population, as followed:

– The popPK model obtained in healthy volunteers (Erreur ! Signet non défini.) will be used to simulate 1000 replicates of patients' putative concentrations using actual patient settings. Patient settings includes the available dosing information (dose received, regimen, all information collected related to compliance, timing of administration and sampling time) and the covariates that were selected in the model. Measured patients concentrations will be superimposed to the 90% percentiles band and median of simulated data like for Visual predictive check (VPC):
    - If median, 5th and 95th percentile of pitolisant concentration in patients, will fall entirely in the 90 simulated healthy volunteer's band. Data of healthy volunteers with the same dosing regimen as patient and with both single and multiple doses will be combined to patients population;
    - If healthy volunteers model could not be applied to patients data, patient specific model will be build.

For both methods, the process for model refinement or model building will be the same and will follow the following strategy:

1. Selection of the simplest structural model, which predicts the serum concentration as a function of time and dose, based on smallest objective function and by the pattern in the residual plots. The best estimation method, the most appropriate IIV models, and the residual error model, are identified. The resulting model is called BASE.

2. Graphical exploration of the covariates: between individual covariates and post-hoc (FO) or conditional (FOCE) parameter estimates and between covariates and weighted residuals (WRES).
3. Univariate analysis (covariates are added only to parameters having η in BASE).
4. Multivariate analysis: all selected covariates are added together, the model is fit to data and a new value of objective function (OBJ) will be obtained and considered as a reference called FULL model
5. Selection of significant covariate to obtain the FINAL model

9.2 STRUCTURAL PHARMACOKINETIC MODEL

According to Section 9.1 and after sequential method, two analysis could be developed as follows:

9.2.1 popPK model of patients pooled with healthy volunteers

In order to pool patient and healthy volunteer's data on the popPK models, first of all, the original model performed on healthy volunteers (1) will be check if it can be predicted to the patient's data.

OSA patients' concentrations will be simulated using the original model developed in healthy volunteer's data and patient's characteristics (dosing regimen, sampling time and demographics). One thousand replicates of the OSA patients will be obtained and used to produce VPC plots:

– Simulation-based 90% prediction intervals (defined as the interval between the 5% and the 95% percentile from simulated datasets) will be obtained by time point for the 5%, the median and the 95% percentiles and presented graphically as bands.
– The 5%, 50% (median) and 95% percentiles of the original serum concentration will be calculated and presented graphically as line over time.
– Individual OSA patient's concentrations over time will be added on the figure.

Using these diagnostics it should be possible to conclude whether the original model:

1. **can predict patients data:** OSA patients data will be pooled with healthy volunteers (with the same dosing regimen), data and the model will be refined according to patients characteristics and covariates without change to the structural model.
2. **is acceptable but need to be modified:** the structural model need to be slightly adapted to capture patient's specific feature (shift in absorption, distribution or elimination phase). In that case the OSA patients data will be pooled with healthy volunteers data, a new structural model will be obtained and the model building process will be followed up to reach a final model.
3. **is unable to predict patient data:** the structural models appeared different and the difference cannot be captured by patient specific covariates. In that case a patient specific model will be built following all of steps of the model building (As presented in Section 9.2.2)

In all cases, model components, covariates and final model determination and qualification will be performed according to the methodology described in Section 9.3.

9.2.2 popPK model only on patients

For patients model only, the first step will focus on the development of the structural form for the fixed effects (θ) in the base model.

Model development will start from a one-compartmental model based on pitolisant concentrations. If this model fails to fit correctly the data, more complex models will be attempted (2 or more compartments, different forms of absorption or elimination, etc.).

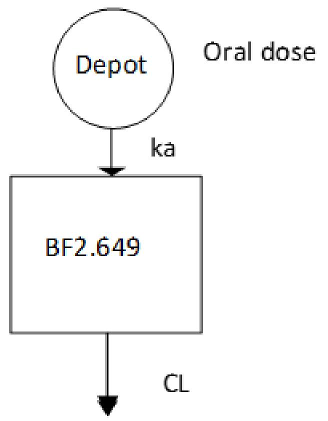

The model will be upgraded up to find the best structural model that fit the concentrations as a function of time and dose. The update of the model can consist on adding compartments, trying alternative absorption or elimination models or including structural covariates.

At this step, refinement of random effect model may not be necessary, therefore, the selection of structural model will be based on fixed effects only (no IIV, η) and simple residual models.

Once the structural model will be defined, the residual error model will be refined and finally the IIV will be introduced on the PK parameters for which the estimation of the variability can be supported by the data. At this step the error models will be diagonal (no covariance between variance components will be included).

The selection of the most parsimonious structural model with appropriate IIV and residual error will be called the BASE model.

9.3 POPULATION PK MODEL COMPONENTS, MODEL BUILDING STEPS AND QUALIFICATION

9.3.1 Error models specification (random effects)

Total variability (model errors) will be split as inter-individual and intra-individual components.

In population modelling, each subject is assumed to be sampled from a representative population. Thus, the individual PK profiles can be considered as a random realization of a population PK model, which by extension is equivalent to consider that each individual profile is obtained by a subject specific random realisation of each parameter of the model.

The model parameters are thus considered as random effects having a mean value (or typical value) and a variability. These variabilities are the inter-individual variability component.

Other sources of error (noise, error or measurements, model misspecification, etc.) are included in the residual error term and associated with the intra-individual variability component.

The objective of the population PK modelling is to obtain estimates of the typical values and the variability of the parameters, as well as of the residual error.

9.3.1.1 Models for inter-individual variabilities (IIV)

Let θ_i refers to the parameter value in the ith individual, θ_{TV} is the typical value of the parameter in the population, and η is a Gaussian random variable with a mean of 0 and a variance of ω^2.

Two models for IIV can be considered:

1. The exponential model:

$$\theta_i = \theta_{TV} e^{\eta_i}$$

The exponential model is useful for a lognormal distribution. In that case θ_{TV} denotes the median of the distribution.

2. The additive model:

$$\theta_i = \theta_{TV} + \eta_i$$

The additive model is a standard Gaussian model with constant standard deviation (SD).

As far as possible, the exponential model will be considered for PK parameters in order to ensure strictly positive values. However, in some circumstances, the additive model could provide a better fit and should be considered. The distribution of the random terms will be investigated to determine the best model.

9.3.1.2 Model for residual variability

Three different models of residual variability will be tested on measurable serum concentrations:

The additive error model: $Y=f+\varepsilon$

The multiplicative error model: $Y=f.(1+\varepsilon)$

The mixed error model: $Y=f+\varepsilon_1+f.\varepsilon_2$

Where Y are the observed concentrations, f are predicted concentration, and ε, ε_1 and ε_2 are random variables with a mean of 0 and variance respectively of σ^2, σ_1^2 and σ_2^2.

Residual variability will be expressed as a coefficient of variation (CV) for multiplicative error model and SD for additive model.

9.3.2 Covariate models

The objective of the inclusion of significant covariates in a model can be:

- explain the random variability (and thus reduce IIV)
- Understand causes of variability and apply the knowledge
- Improve the predictive performance of the model for further use

Let:

θ_{TV} be the typical value for a PK parameter (*e.g.* CL/F, V/F, etc.)

θ_{pop} be the population value for this parameter

COV_i be a covariate:

If COV_i is continuous,

If COV_i is categorical COV_i=1 means presence of the characteristic, COV_i=0 means absence of the characteristic.

θ_i be the fixed parameter associated to the covariate.

Commonly used functional form for continuous covariates are:

$$\theta_{TV} = \theta_{pop} + \theta_i(COV_i - Median_{Cov_i}) \text{ (linear)}$$

or

$$\theta_{TV} = \theta_{pop} \times (1 + \theta_i(COV_i - Median_{Cov_i})) \text{ (proportional)}$$

or

$$\theta_{TV} = \theta_{pop} \times \left(\frac{COV_i}{Median_{Cov_i}}\right)^{\theta_i} \text{ (power)}$$

or

$$\theta_{TV} = \theta_{pop} \times \exp\left(\theta_i(COV_i - Median_{Cov_i})\right)$$

The effect of categorical covariates will be evaluated using the following models as appropriate:

$$\theta_{TV} = \theta_{pop} + \theta_i \times COV_i \text{ (additive)}$$

or

$$\theta_{TV} = \theta_{pop} \times (1 + \theta_i \times COV_i) \text{ (proportional)}$$

The nature of the functional form for each (covariate, parameter) pair will be determined using a graphical approach involving individual predictions if the shrinkage value of the random effect associated to the parameter is limited (≤ 30%).

When using the FOCE method, if the shrinkage is >30%, the conditional individual values are considered to shrink toward the corresponding observations and to be uninformative at the individual level. In that case, the graphical approach should be used with caution and all structural forms will be considered and the form improving the most the OFV will be selected.

The graphical exploration will consist on scatter plots with smoothing curve for continuous covariates and box-plots for categorical covariates between each covariate and:

- Individual parameters estimated from the BASE model;
- Weighted residual from the BASE model.

The model of parameters including covariates will depend on the shape of the relationship evaluated during graphical exploration.

The following set of covariates will be investigated:

- Dose on absorption phase;
- Influence of body weight or BMI (whatever the most relevant, but not both simultaneously) on CL/F and V/F;
- Influence of gender on CL/F and V/F;
- Influence of co-medications (needs to be grouped by the pharmacological action in agreement with sponsor) on CL/F and V/F.
- Influence of stage of OSA (moderate or severe) on CL/F and V/F;

Only covariates, including type of drugs for co-medications, which were measured or reported in at least 15% of the population will be considered for the covariate model.

9.3.3 Statistical model selection

Once the BASE model was obtained, influence of predefined covariates will be investigated first using a univariate analysis.

The following procedure will be used for the univariate analysis:

- One covariate effect on a single model parameter is included in the BASE model per run;
- Take care not to include several covariates that are markers of the same feature (*e.g.* weight and BMI) in order to avoid colinearity. Choose the best;
- Addition of a covariate is significant if the LRT gives a p-value < 0.05 (*i.e.* $\Delta OF \geq 3.84$);
- Repeat these steps with all covariates and parameters suspected to be affected by the covariates;
- Rank the covariates by size of effect on OF by comparison to the base model (the largest decrease is ranked 1).

Once the univariate step is complete and covariates were selected, a backward multivariate analysis will be performed:

- Add all significant covariates selected by the univariate steps to the model and get a new OF for this FULL model;
- Proceed to a backward deletion until no covariate can be removed without significantly increasing the objective function:

- Remove the covariates once at a time, starting with the weakest obtained by the ranking of the univariate process. Fit to the data, get the OF and compare to the OF of the FULL model. Decide to keep the covariate in the model if the covariate is significant at the α^* level where α^* is an adjusted level insuring a maximal overall 5% type I error over all tests performed.
- Repeat this step until all covariates are tested versus the last accepted model.

- When all covariates are tested, get the FINAL model and compute 95% confidence intervals (CI) on the parameters. 95%CI will be computed as $\theta \pm 1.96 \times SE(\theta)$.

9.3.4 Estimation method and model acceptance

Due to the rich sampling nature of the data the FOCE method (with η-ε interaction) will be the preferred method *a-priori*.

At each step of the model building, intermediate models will be accepted if:

- Minimization is successful.
- Covariance step finishes without warning message.
- Number of significant digits on parameters is superior or equal to 3.
- Correlation between any 2 parameters (θ, η or ε) is inferior to 0.95.
- Absolute values of gradients at last iteration are superior to 0 to avoid local minima.

For the final model, in addition to these criteria, the following will be evaluated:

- 95%CI of estimates excludes 0. If 0 is included within the 95%CI, this will lead to exclusion of the corresponding parameter from the model unless it will be part of the structural PK model;
- Over-parameterization will be assessed using the condition number for which a value exceeding 1000 is indicative of severe ill-conditioning.

9.3.5 Models evaluation and goodness of fit

At each step of the model building appropriateness of the model must be assessed.

The following plots are applicable at each step of model building:

- Random scatter of residuals (PWRES and NPDE) around 0 against time, concentrations [DV], PK predictions (PPRED and IPRED) and covariates
- Random scatter of population predictions (PPRED) and individual predictions (IPRED) against concentrations (DV) around line of identity
- Plot of population prediction (PPRED), individual predictions (IPRED) and concentrations (DV) against time can be provided for the BASE and FINAL models

For the covariate models, the following plots will be provided:

- Index plots of residuals (PWRES) against covariate,
- Index plots of estimates (ETAs) against covariate.

Goodness of fit plots can be stratified by study protocol, age group or any other condition that is deemed relevant.

Only GOF for the BASE and FINAL (if applicable) model will be compiled for inclusion in the popPK report, all other intermediates GOF plots could be generated on request but will not be saved.

For the FINAL model, quality of individual PK parameters estimates will be assessed using shrinkages.

10 EVALUATION OF PREDICTABILITY AND STABILITY OF THE MODEL

Evaluation of predictability and stability of the FINAL popPK models will be performed using prediction-corrected performance visual checks (pcVPC). The pcVPC were shown to be a more powerful diagnostic tool than classical VPC to detect important model misspecifications in the context of single ascending dose studies [(3)]. Especially because they aim to correct for the differences within a time bin coming from independent variables such as dose level, largest source of differences in a single ascending dose study. Hence pcVPC reduced also the need for stratification by dose level, likely to be uninformative with 8 subjects by dose level.

The pcVPC will be computed and displayed as follow:

- Like for classical VPC, perform a large number of replication (N ≥ 1000) of the original dataset using the parameters estimated in the FINAL model through Monte Carlo (MC) simulations.
- pcYij= Yij.(PREDbin / PREDij)

  where:

  Yij = observation or simulated prediction for the ith individual and j^{th} time bin

  pcYij = prediction-corrected observation or simulated prediction

  PREDij = typical population prediction for the ith individual and j^{th} time bin

  PREDbin = median of typical population prediction for the specific bin of independent variable
- The 5%, 50% (median) and 95% percentiles of the original serum concentration or PD endpoint will be calculated and presented graphically over time.
- Simulation-based 90% prediction intervals (defined as the interval between the 5% and the 95% percentile from simulated datasets) will be obtained by time point for the 5%, the median and the 95% percentiles and presented graphically as bands.
- A line for the observed median or 5%-95% percentiles moving outside its respective simulation-based prediction interval denote a model misspecification regarding central tendency (median) or variability (5% -95% percentiles).

11 DISPLAY OF RESULTS AND CONTENTS OF THE REPORT

11.1 DESCRIPTION OF THE DATA

The data used for the population will be described in the report using the following information:

- Total number of patients entering the analysis;
- Total number of concentrations used in the analysis;
- Total number of missing concentrations, including BLQ;
- Subjects removed from the analysis listed with relevant subject characteristics and reason for withdrawal;
- Summary of demographic characteristics: descriptive statistics (N, mean, SD, minimum (Min), median, and maximum (Max)) for continuous variables and frequencies for categorical covariates;
- Descriptive statistics of concentrations, including number of observations available, number of observations BLQ or missing, mean, SD, GM, CV, Min, median, and Max;
- Graphical display of median concentrations over time in natural scale and log scale;

- Individual spaghetti plots in natural scale and log scale;
- Subjects considered as outliers specified with all relevant data available

11.2 BASE MODEL

Only outputs for the relevant steps will be included in the Appendix, other intermediate run outputs will be stored and kept on the network. However, all run will be summarized in a run record describing any major decisions and including an overview of the steps taken during the model development.

The run record will include a brief description of the run, the objective function value, if the model converged successfully, the parameter estimates with their standard error. If the analysis dataset change during the course of the analysis, the changes must be documented in the run record.

All parameters estimates in the BASE model will be presented in a table together with their standard errors and their confidence intervals. Inter-individual and residual variability models will also be reported.

GOF plots as defined in Section 0 will be included in the report.

11.3 COVARIATE SELECTION AND FINAL MODEL

Plots generated to screen for potential covariate relationships will be provided in the report (for example individual estimates of parameters versus potential covariates).

The covariate model building steps will be presented in a separate run record, displaying covariates that will be included in the FINAL model and those that were tested but not retained, together with the criteria on which the decision was based (ΔOF). A run record will be provided for the covariate selection.

The results for the FINAL model will be presented in a table together with the standard errors and the confidence intervals of the parameters estimates (model parameters, covariates and variability parameters).

GOF plots as defined in Section 0 will be included in the report.

The NONMEM input and output files for the BASE and FINAL models will be provided in an appendix.

11.4 MODEL EVALUATION

Figures for pcVPC will be presented in the report.

12 DERIVED DATA

For a 1-compartment model the PK exposure parameters will be calculated from individual predictions obtained with the final population PK model as follows:

$$AUC_{0-tau} = \frac{dose\ administered}{CL}$$

$$t_{1/2} = \frac{\ln 2}{k_e}, where\ k_e = \frac{CL}{V_1},$$

For a 2-compartment model the PK exposure parameters will be calculated from individual predictions obtained with the final population PK model as follows:

$$AUC_{0-tau} = \frac{dose\ administered}{CL}$$

$$t_{1/2\beta} = \frac{\ln 2}{\beta}, where\ \beta = \frac{1}{2}\left[k_{12} + k_{21} + k_{10} - \sqrt{(k_{12} + k_{21} + k_{10})^2 - 4k_{21}.k_{10}}\right]$$

13 SOFTWARE USED

SAS® software V9.4 will be used for:

- Structuring the analysis dataset from the source data (bioanalytical and clinical data base) to a format suitable to NONMEM;
- Produce graphs;
- Produce summary tables (*e.g.* description of the data, numerical PPC, etc.).

NONMEM version 7.4 will be used for:

- PopPK modelling;
- NONMEM will be used through PDx-POP version 5.2.2 interface.

14 REFERENCE LIST

1 PhinC Development. Population PK report “PH14056_PKpop_Version 1.0_20150304”. March 4th, 2015.

2 Beal SL. Ways to fit a PK model with some data below the quantification limit. *J. Pharmacokinet. Pharmacodyn.* 28, 481-504, 2001.

3 Bergstrand M, Hooker AC, Wallin JE, Karlsson MO. Prediction-Corrected Visual Predictive Checks for diagnosing Nonlinear Mixed-Effect Models. The AAPS Journal, Vol. 13 No. 2, June 2011.

16.1.9.3 Interim Safety Report

- DSMB Meeting minutes (December 16th, 2016)
- DSMB Meeting minutes (March 08th, 2017)
- DSMB Meeting minutes (September 13th, 2017)
- DSMB Meeting minutes (February 06th, 2019)

HAROSA III : BF2.649/Placebo(P1513)

Meeting of the Safety Data Monitoring Committee

Meeting Minutes

Attendees

[redacted] Bron Hospital, Lyon, France (HB)
[redacted] Biostatistician, Veeda Clinical Research, Brussels Belgium (LK)
[redacted], Bioprojet, Paris, France (IL)
[redacted], Bioprojet, Paris, France (YJ)
[redacted], Bioprojet, Paris, France (KG)
[redacted]ioprojet, Paris, France (DB)
Bioprojet, Paris, France (AK)
[redacted]ioprojet, Paris, France (VP)

Excused [redacted] Saint Antoine Hospital, Paris, France

Author of the minutes [redacted]

Time and place

- **Date:** Friday December 16th 2016
- **Time:** 10:00 - 11:00
- **Location:** Telephonic conference

Item	Discussion Topics / Minutes	Actions/Attachments
**Agenda**	▪ Safety review<br>▪ Recommendations<br>▪ Next meeting	
**Protocol specfics**	▪ Protocol design<br>▪ Goals<br>▪ Patients recruitment status<br>o 202 screened<br>o 183 randomized<br>o 113 completed double blind phase<br>o 80 patients with 100% data clean included in data review.<br>▪ Upcoming Harosa IV study with same design<br>o Study start 1Q2017	Harosa III and Harosa IV data will be reviewed at the future DSMBs

Item	Discussion Topics / Minutes	Actions/Attachments
**Review of Safety Data**	▪ Tables and listings of the 80 patients with data clean were reviewed (Annex 1)<br>▪ An analysis of data was provided by YJ (Annex 2)<br>▪ All AEs regardless of data cleanness status were reviewed.<br>▪ An analysis of data was provided by [redacted] Annex 3)<br>▪ Cases summaries were reviewed for Pts 4112 and 4116 (Annex 3)	▪ Bioprojet to provide additional information on<br>o AEs reported for Pts. 4112 (Henoch- Schönlein) and 4116 (Collapses)<br>o ECGs graphs<br>o Nervousness vs, nerves, vs irritability clarification
**Recommendation**	On the basis of the provided information no change to the study is recommended	
**Next meeting**	Next meeting: TC on either Wednesday March 8th at 10.30 AM (after confirmation with [redacted])	

Subjid	Age	Gender	CPAP	ESS1	ESS2	ESS3	ESS4	ESS5	ESS6	ESS7	OSL2	OSL6	CGIS1	CGIS2	CGIC6
341001, DB-001	56	0	0	15	14	12	10	9	10	8	26,3	40	3	3	2
341002, DB-002	55	1	0	12	14	13	13	12	10	11	23,6	40	3	4	2
341003, DB-003	59	0	1	14	12	12	11	10	5	2	29,6	40	3	3	2
341004, DB-004	41	1	0	12	13	9	8	10	9	15	40	40	3	3	3
341005, DB-005	47	1	0	12	12	10	11	8	7	6	28,9	40	3	3	2
341006, DB-007	67	0	0	14	14	12	9	10	6	3	30	40	3	4	2
341007, DB-011	37	0	0	12	13	10	11	8	6	2	33,6	40	3	3	2
341008, DB-006	50	1	0	14	15	13	13	12	10	4	39	30,9	3	4	1
341009, DB-008	57	0	1	12	15	14	13	9	8	4	33,5	40	3	4	2
341010, DB-043	60	0	0	12	14	10	9	8	2	7	40	40	3	3	2
341011, DB-012	69	0	1	12	12	9	8	7	7	5	30	40	3	4	2
341012, DB-009	63	0	0	19	17						34,1		4	4	
341013, DB-010	40	0	0	12	12	16	11	9	2	3	38	40	3	4	2
341014, DB-044	60	0	1	12	15	14	13	9	7	4	40	40	3	3	2
341015, DB-048	48	0	1	18	17	15	14	12	1	0	40	31,6	4	4	2
341016, DB-045	56	1	0	23	23	21	21	21	17	13	26,1	40	4	4	3
341017, DB-046	38	0	0	14	13	11	14	9	5	4	40	40	3	3	2
341018, DB-047	40	1	0	14	16	15	14	15	9	10	36,6	35	4	4	2
341019, DB-060	51	0	1	12	12	11	10	9	3	3	35,6	40	3	3	2
341020, DB-055	57	0	0	15	15	11	10	11	16	15	40	40	4	4	2
341022, DB-059	33	0	1	17	14	12	12	6	7	9	37	40	4	4	1
341023, DB-057	42	0	1	15	14	13	13	11	7	4	28,6	40	3	4	2
341024, DB-058	65	1	0	16	17	10	9	5	10	10	17	37	4	5	2
341025,	61	1	0	12									4		
341026, DB-066	65	0	1	13	13	8	6	7	3	4	22,4	40	3	3	2
351001, DB-013	57	0	1	13	13	12	12	10	9	15	29,3	37	2	2	3
351002, DB-014	41	0	1	15	16	16	14	8	7	10	24,4	40	3	3	1
351003, DB-015	58	1	1	16	17	17	14	12	9	12	32,9	40	3	3	2
351004, DB-073	62	0	1	16	16	14	13	13	11	11	31,9	38,4	3	3	3
351005, DB-074	59	0	0	16	17	15	13	12	11	13	32,7	37,5	3	3	2
353001, DB-025	70	0	1	14	14	12	12	11	12	12	6,9	12,6	4	4	3
353002, DB-026	40	0	0	15	16	16	16	16	16	15	4,9	9,5	4	4	3
353003,	37	0	0	15									4		
353004, DB-027	60	0	0	14	13	13	13	12	11	11	16,1	5,6	4	4	3
353005,	71	0	0	15									3		
353006, DB-029	46	0	0	15	15	16	14	14	14	14	13,2	17	3	3	2
353007, DB-028	61	0	1	15	15	15	14	14	14	15	6,8	17,7	4	4	3
353008, DB-033	68	0	0	14	14	13	14	13	12	15	16,4	11,1	3	3	4
353009, DB-037	55	0	0	15	15	16	15	15	13	14	3,4	11,8	4	4	3
353010, DB-035	52	0	0	14	14	14	13	14	13	14	4,9	24,4	4	4	1
353011, DB-030	55	1	0	16	15	14	14	13	12	12	3,8	8,2	5	5	3
353012, DB-034	53	0	0	19	18	18	18	17	16	15	9,4	20,4	5	5	3
353013, DB-039	54	0	0	13	13	13	13	11	10	10	10,8	15,4	4	4	3
353014, DB-031	72	1	0	14	14	14	13	12	12	12	9,5	11,4	4	4	3
353015, DB-036	51	0	0	18	18	17	16	16	13	16	10,1	19,2	5	5	2
353016, DB-032	71	0	0	15	15	15	14	14	13	15	8	9,1	4	4	3
353017, DB-038	71	0	1	16	16	14	13	12	9	9	2,8	9,2	5	5	3
353018, DB-050	53	1	0	18	19	18	17	13	12	12	10,1	31,2	5	5	1
353019, DB-040	62	0	0	16	16	15	15	14	13	15	9,2	12,1	4	4	4
353020, DB-041	59	0	0	17	18	17	15	13	11	15	10,7	14,9	5	5	3
353021, DB-042	52	0	0	14	14	15	14	12	10	13	6,5	8	4	4	4
353022, DB-051	59	1	0	16	16	15	14	14	12	14	3,8	12,5	4	4	2
353023, DB-049	44	0	0	17	18	17	14	14	13	17	9,3	9,3	4	4	3
353024, DB-079	57	1	1	16	16	14	14	15	14	15	9,1	12,1	4	4	3
353025, DB-053	45	1	0	13	14	13	12	10	10	13	11,2	32,5	4	4	1
353026, DB-054	46	0	0	13	14	14	12	13	13	13	10,5	32	4	4	1
353027, DB-080	68	0	1	13	13	12	13	13	12	12	33,6	23,7	4	4	4
353028, DB-081	61	0	1	13	13	13	14	14	13	14	20,7	14,9	4	4	4
353029, DB-052	51	0	1	14	15	14	11	11	11	11	14,7	18,6	4	4	2
353030, DB-082	66	0	1	14	14	14	13	13	12	12	33,5	22,7	5	5	4

Subjid	Age	Gender	CPAP	ESS1	ESS2	ESS3	ESS4	ESS5	ESS6	ESS7	OSL2	OSL6	CGIS1	CGIS2	CGIC6
353031, DB-083	58	1	1	15	15	14	14	13	12	12	14,6	32	4	4	1
353032, DB-084	59	0	1	15	15	14	14	13	11	11	15,3	36,5	4	4	1
353033, DB-085	63	1	1	13	14	14	12	10	9	11	7,2	14,2	4	4	3
353034,	41	0	0	19									5		
353035, DB-089	58	0	0	17	17	17	15	14	14	14	7,8	8,7	4	4	4
353036, DB-091	55	0	0	20	20	20	19	17	17	16	21,2	15,9	5	5	4
353037, DB-090	42	0	0	19	19	17	16	16	15	15	24,7	23,3	5	5	4
353038, DB-088	38	0	0	17	17	15	13	12	12	14	4,3	19,2	4	4	3
353039, DB-094	56	1	0	15	15	14	14	13	13	15	11	32,1	4	4	1
353040, DB-092	40	1	0	19	19	18	17	16	15	15	15,7	26,1	5	5	3
353041, DB-086	62	0	0	15	15	13	12	11	10	10	7,3	16,6	4	4	2
353042, DB-087	56	0	0	14	14	12	10	9	9	9	9,4	30,8	4	4	1
353043, DB-096	37	0	0	16	16	16	15	15	14	13	17	17,2	4	4	4
353044, DB-098	48	0	1	14	15	14	14	12	12	13	11,8	16	4	4	3
353045, DB-093	37	0	0	22	24	22	21	20	19	19	8,3	25,7	6	6	3
353046, DB-100	47	0	1	15	15	14	15	14	14	16	10,7	17,3	4	4	3
353047, DB-097	21	1	0	16	17	15	14	16	14	13	17,9	33,8	4	4	2
353048, DB-095	66	0	0	14	15	14	14	14	13	15	14,6	15,2	4	4	4
353049, DB-099	61	0	0	18	18	17	16	15	14	14	11	20,4	5	5	3
353050, DB-101	64	0	0	14	14	14	12	12	11	13	6,6	30,7	4	4	1
353051, DB-102	47	0	0	16	16	15	14	13	13	15	5,2	22,7	4	4	2
353052, DB-163	52	1	0	15	16	15	14	13	13	13	6,9	19,1	4	4	3
353053, DB-164	56	1	1	13	14	13	13	12	13	12	10,9	21,7	4	4	3
353054, DB-165	52	1	0	13	14	13	12	11	11	13	7,1	16,8	4	4	3
356001, DB-127	55	0	0	19	19	15	12	12	13	11	13,8	18,4	6	6	1

P15-13 _DSMB_ Demographics_

Subject ID	Age (years)	Sex	Weight (kg)	BP Sys (mmHg)	BP Dia (mmHg)	HR (beats/min)	nCPAP Therapy Status	Completed visit 7 and 100% SDV
341001, DB-001	56	Male	102	130	80	64	No	Yes
341002, DB-002	55	Female	70	130	80	72	No	Yes
341003, DB-003	59	Male	101	120	75	62	Yes	Yes
341004, DB-004	41	Female	94	120	70	61	No	Yes
341005, DB-005	47	Female	98	120	80	76	No	Yes
341006, DB-007	67	Male	92	127	85	75	No	Yes
341007, DB-011	37	Male	105	130	80	67	No	Yes
341008, DB-006	50	Female	100	130	80	67	No	Yes
341009, DB-008	57	Male	133	130	80	72	Yes	Yes
341010, DB-043	60	Male	120	120	80	78	No	Yes
341011, DB-012	69	Male	98	130	80	92	Yes	Yes
341012, DB-009	63	Male	102	135	82	88	No	No
341013, DB-010	40	Male	102	120	80	76	No	Yes
341014, DB-044	60	Male	101	125	80	77	Yes	Yes
341015, DB-048	48	Male	120	120	85	80	Yes	Yes
341016, DB-045	56	Female	105	110	80	76	No	Yes
341017, DB-046	38	Male	86	120	70	85	No	Yes
341018, DB-047	40	Female	95	120	80	67	No	Yes
341019, DB-060	51	Male	120	115	70	70	Yes	Yes
341020, DB-055	57	Male	105	125	83	74	No	Yes
341022, DB-059	33	Male	125	123	80	86	Yes	Yes
341023, DB-057	42	Male	100	130	80	75	Yes	Yes
341024, DB-058	65	Female	100	130	80	76	No	Yes
341026, DB-066	65	Male	103	130	75	66	Yes	Yes
351001, DB-013	57	Male	110	125	80	85	Yes	Yes
351002, DB-014	41	Male	123	130	80	84	Yes	Yes
351003, DB-015	58	Female	96	130	85	70	Yes	Yes
351004, DB-073	62	Male	100	125	80	63	Yes	Yes
351005, DB-074	59	Male	113	130	85	63	No	Yes
353001, DB-025	70	Male	110	125	80	77	Yes	Yes
353002, DB-026	40	Male	115	120	70	75	No	Yes
353004, DB-027	60	Male	130	130	80	87	No	Yes
353006, DB-029	46	Male	120	130	80	75	No	No
353007, DB-028	61	Male	118	120	75	67	Yes	Yes
353008, DB-033	68	Male	120	125	75	71	No	Yes
353009, DB-037	55	Male	102	140	80	74	No	Yes
353010, DB-035	52	Male	100	130	80	90	No	Yes
353011, DB-030	55	Female	105	140	90	96	No	Yes
353012, DB-034	53	Male	125	130	75	86	No	Yes
353013, DB-039	54	Male	114	140	75	88	No	Yes

353014, DB-031	72	Female	70	120	70	71	No	Yes
353015, DB-036	51	Male	110	125	80	70	No	Yes
353016, DB-032	71	Male	130	140	80	73	No	Yes
353017, DB-038	71	Male	76	120	80	66	Yes	Yes
353018, DB-050	53	Female	95	120	80	77	No	Yes
353019, DB-040	62	Male	99	135	80	87	No	Yes
353020, DB-041	59	Male	100	135	80	60	No	Yes
353021, DB-042	52	Male	114	120	70	74	No	Yes
353022, DB-051	59	Female	100	135	80	93	No	Yes
353023, DB-049	44	Male	95	120	70	85	No	Yes
353024, DB-079	57	Female	96	135	80	96	Yes	Yes
353025, DB-053	45	Female	114	130	75	64	No	Yes
353026, DB-054	46	Male	115	120	75	73	No	Yes
353027, DB-080	68	Male	110	140	80	84	Yes	No
353028, DB-081	61	Male	120	135	80	108	Yes	Yes
353029, DB-052	51	Male	110	125	80	62	Yes	Yes
353030, DB-082	66	Male	115	130	80	85	Yes	Yes
353031, DB-083	58	Female	106	125	70	68	Yes	Yes
353032, DB-084	59	Male	135	120	80	82	Yes	Yes
353033, DB-085	63	Female	93	130	80	85	Yes	Yes
353035, DB-089	58	Male	86	130	85	74	No	Yes
353036, DB-091	55	Male	122	120	80	80	No	Yes
353037, DB-090	42	Male	130	135	80	70	No	Yes
353038, DB-088	38	Male	94	110	70	80	No	Yes
353039, DB-094	56	Female	115	140	80	88	No	Yes
353040, DB-092	40	Female	117	140	80	78	No	Yes
353041, DB-086	62	Male	101	130	70	64	No	No
353042, DB-087	56	Male	90	120	70	52	No	Yes
353043, DB-096	37	Male	139	140	80	79	No	Yes
353044, DB-098	48	Male	120	120	80	86	Yes	Yes
353045, DB-093	37	Male	129	130	85	93	No	No
353046, DB-100	47	Male	125	130	80	93	Yes	Yes
353047, DB-097	21	Female	122	120	80	69	No	Yes
353048, DB-095	66	Male	121	130	80	75	No	Yes
353049, DB-099	61	Male	100	130	80	61	No	Yes
353050, DB-101	64	Male	110	130	80	77	No	Yes
353051, DB-102	47	Male	115	140	80	87	No	Yes
353052, DB-163	52	Female	92	125	70	66	No	Yes
353053, DB-164	56	Female	100	140	80	63	Yes	Yes
353054, DB-165	52	Female	82	125	80	93	No	Yes
353055, DB-166	47	Female	93	120	80	75	No	Yes
356001, DB-127	55	Male	130	140	90	85	No	Yes

Subject ID	Reported Term for the Adverse Event	Intensity	Outcome of Adverse Event	Age (years)	Sex	First day of treatment	Start Date/Time of Adverse Event	Number of days under treatment before AE onset	Causality	AE end date	Completed visit 7 and 100% SDV
341003, DB-003	acute viral infection	Moderate	Recovered	59	Male	2016-04-21	2016-08-08	109	Unlikely related	15/08/2016	Yes
341003, DB-003	nervous tension	Mild	Recovered	59	Male	2016-04-21	2016-05-06	15	Likely related	11/05/2016	Yes
341007, DB-011	headache	Mild	Recovered	37	Male	2016-04-26	2016-05-04	8	Likely related	10/05/2016	Yes
341011, DB-012	headache	Mild	Recovered	69	Male	2016-04-26	2016-08-16	112	Likely related	22/08/2016	Yes
341012, DB-009	Henoch–Schönlein purpura	Moderate	Recovered with sequelae	63	Male	2016-04-26	2016-05-01	5	Possibly related	10/05/2016	No
341013, DB-010	headache	Moderate	Recovered	40	Male	2016-04-26	2016-05-11	15	Possibly related	19/05/2016	Yes
341013, DB-010	nervousness	Mild	Recovered	40	Male	2016-04-26	2016-05-11	15	Possibly related	18/08/2016	Yes
341014, DB-044	nerves	Mild	Recovered	60	Male	2016-04-27	2016-05-05	8	Possibly related	11/05/2016	Yes
341016, DB-045	collapse	Mild	Recovered	56	Female	2016-04-28	2016-05-07	9	Possibly related	07/05/2016	Yes
341016, DB-045	collapse	Mild	Recovered	56	Female	2016-04-28	2016-05-09	11	Possibly related	09/05/2016	Yes
341020, DB-055	headache	Moderate	Recovered	57	Male	2016-05-27	2016-06-12	16	Possibly related	19/06/2016	Yes
341020, DB-055	insomnia	Moderate	Recovered	57	Male	2016-05-27	2016-09-10	106	Possibly related	11/09/2016	Yes
341021, DB-056	headache	Moderate	Recovered	58	Female	2016-05-27	2016-06-12	16	Possibly related	19/06/2016	No
341021, DB-056	headache	Moderate	Recovered	58	Female	2016-05-27	2016-09-15	111	Possibly related	22/12/2016	No
351003, DB-015	Worsening of existing Dislipidaemia	Mild	Not yet recovered	58	Female	2016-05-10	2016-08-02	84	Unlikely related	Ongoing	Yes
351003, DB-015	Worsening of exiting Dislipidaemia	Mild	Recovered	58	Female	2016-05-10	2016-08-02	84	Unlikely related	23/08/2016	Yes
353007, DB-028	Impaired vision	Moderate	Recovered	61	Male	2016-04-27	2016-07-28	92	Unlikely related	13/08/2016	Yes
353007, DB-028	Edemas under your eyes	Moderate	Recovered	61	Male	2016-04-27	2016-07-28	92	Unlikely related	13/08/2016	Yes
353009, DB-037	burning breasts- bit accident	Moderate	Not yet recovered	55	Male	2016-05-30	2016-09-17	110	Unlikely related	Ongoing	Yes
353016, DB-032	Irritability	Moderate	Recovered	71	Male	2016-04-28	2016-05-13	15	Likely related	19/05/2016	Yes
353021, DB-042	Headache	Moderate	Recovered	52	Male	2016-05-31	2016-06-08	8	Likely related	13/06/2016	Yes
353021, DB-042	Sleep disturbance, nightmares	Moderate	Recovered	52	Male	2016-05-31	2016-06-09	9	Likely related	13/06/2016	Yes
353033, DB-085	headache	Moderate	Recovered	63	Female	2016-07-02	2016-07-17	15	Likely related	20/06/2016	Yes
353033, DB-085	Insomnia	Moderate	Recovered	63	Female	2016-07-02	2016-10-16	106	Likely related	23/10/2016	Yes
353035, DB-089	Insomnia	Moderate	Recovered	58	Male	2016-07-04	2016-10-18	106	Likely related	24/10/2016	Yes
353041, DB-086	headache	Moderate	Recovered	62	Male	2016-07-02	2016-10-16	106	Likely related	23/10/2016	No
353045, DB-093	Phlegmona femoris dextra	Moderate	Recovered	37	Male	2016-07-05	2016-10-03	90	Unlikely related	10/10/2016	No
353048, DB-095	Single supraventricular extrasystoles.	Mild	Recovered	66	Male	2016-07-06	2016-08-24	49	Unlikely related	24/08/2016	Yes

P1513 Harosa III Treatment Emergent Adverse Events

P1513 Harosa III Adverse Events

Site name	Subject ID	Reported Term for the Adverse Event	Intensity	Outcome of Adverse Event	Age (years)	Sex	First day of treatment	Start Date/Time of Adverse Event	Number of days under treatment before AE onset	Causality	AE end date	Completed visit 7 and 100% SDV
341@SUMHA ALEKSANDROVSKA	341003, DB-003	acute viral infection	Moderate	Recovered	59	Male	2016-04-21	2016-08-08	109	Unlikely related	15/08/2016	Yes
341@SUMHA ALEKSANDROVSKA	341003, DB-003	nervous tension	Mild	Recovered	59	Male	2016-04-21	2016-05-06	15	Likely related	11/05/2016	Yes
341@SUMHA ALEKSANDROVSKA	341007, DB-011	headache	Mild	Recovered	37	Male	2016-04-26	2016-05-04	8	Likely related	10/05/2016	Yes
341@SUMHA ALEKSANDROVSKA	341011, DB-012	headache	Mild	Recovered	69	Male	2016-04-26	2016-08-16	112	Likely related	22/08/2016	Yes
341@SUMHA ALEKSANDROVSKA	341012, DB-009	Henoch–Schönlein purpura	Moderate	Recovered with sequelae	63	Male	2016-04-26	2016-05-01	5	Possibly related	19/05/2016	No
341@SUMHA ALEKSANDROVSKA	341013, DB-010	headache	Moderate	Recovered	40	Male	2016-04-26	2016-05-11	15	Possibly related	19/05/2016	Yes
341@SUMHA ALEKSANDROVSKA	341013, DB-010	nervousness	Mild	Recovered	40	Male	2016-04-26	2016-05-11	15	Possibly related	18/06/2016	Yes
341@SUMHA ALEKSANDROVSKA	341014, DB-044	nerves	Mild	Recovered	60	Male	2016-04-27	2016-05-05	8	Possibly related	11/05/2016	Yes
341@SUMHA ALEKSANDROVSKA	341016, DB-045	collapse	Mild	Recovered	56	Female	2016-04-28	2016-05-07	9	Possibly related	07/05/2016	Yes
341@SUMHA ALEKSANDROVSKA	341016, DB-045	collapse	Mild	Recovered	56	Female	2016-04-28	2016-05-09	11	Possibly related	09/05/2016	Yes
341@SUMHA ALEKSANDROVSKA	341020, DB-055	headache	Moderate	Recovered	57	Male	2016-05-27	2016-06-12	16	Possibly related	19/06/2016	Yes
341@SUMHA ALEKSANDROVSKA	341020, DB-055	insomnia	Moderate	Recovered	57	Male	2016-05-27	2016-09-10	106	Possibly related	11/09/2016	Yes
341@SUMHA ALEKSANDROVSKA	341021, DB-056	headache	Moderate	Recovered	58	Female	2016-05-27	2016-06-12	16	Possibly related	19/06/2016	No
341@SUMHA ALEKSANDROVSKA	341021, DB-056	headache	Moderate	Recovered	58	Female	2016-05-27	2016-09-15	111	Possibly related	22/12/2016	No
351@PMHAT PARASKEVA	351003, DB-015	Worsening of existing Dislipidaemia	Mild	Not yet recovered	58	Female	2016-05-10	2016-08-02	84	Unlikely related	Ongoing	Yes
351@PMHAT PARASKEVA	351003, DB-015	Worsening of exiting Dislipidaemia	Mild	Recovered	58	Female	2016-05-10	2016-08-02	84	Unlikely related	23/08/2016	Yes
353@KMHAT RILSKI	353007, DB-028	Impaired vision	Moderate	Recovered	61	Male	2016-04-27	2016-07-28	92	Unlikely related	13/08/2016	Yes
353@KMHAT RILSKI	353007, DB-028	Edemas under your eyes	Moderate	Recovered	61	Male	2016-04-27	2016-07-28	92	Unlikely related	13/08/2016	Yes
353@KMHAT RILSKI	353009, DB-037	burning breasts- bit accident	Moderate	Not yet recovered	55	Male	2016-05-30	2016-09-17	110	Unlikely related	Ongoing	Yes
353@KMHAT RILSKI	353016, DB-032	Irritability	Moderate	Recovered	71	Male	2016-04-28	2016-05-13	15	Likely related	19/05/2016	Yes
353@KMHAT RILSKI	353021, DB-042	Headache	Moderate	Recovered	52	Male	2016-05-31	2016-06-08	8	Likely related	13/06/2016	Yes
353@KMHAT RILSKI	353021, DB-042	Sleep disturbance, nightmares	Moderate	Recovered	52	Male	2016-05-31	2016-06-09	9	Likely related	13/06/2016	Yes
353@KMHAT RILSKI	353033, DB-085	headache	Moderate	Recovered	63	Female	2016-07-02	2016-07-17	15	Likely related	20/09/2016	Yes
353@KMHAT RILSKI	353033, DB-085	Insomnia	Moderate	Recovered	63	Female	2016-07-02	2016-10-18	108	Likely related	23/10/2016	Yes
353@KMHAT RILSKI	353035, DB-089	Insomnia	Moderate	Recovered	58	Male	2016-07-04	2016-10-18	106	Likely related	24/10/2016	Yes
353@KMHAT RILSKI	353041, DB-086	headache	Moderate	Recovered	62	Male	2016-07-02	2016-10-18	108	Likely related	23/10/2016	No
353@KMHAT RILSKI	353045, DB-093	Phlegmona femoris dextra	Moderate	Recovered	37	Male	2016-07-05	2016-10-03	90	Unlikely related	10/10/2016	No
353@KMHAT RILSKI	353048, DB-095	Single supraventricular extrasystoles	Mild	Recovered	66	Male	2016-07-06	2016-08-24	49	Unlikely related	24/08/2016	Yes

Visit/Form	Visit 1	Visit 2	Visit 3	Visit 4	Visit 5	Visit 6	Visit 7	Visit 1-7	Phone contact 1	Phone contact 2	Patient Information	Medical / Surgical History	Prior and Concomitant Treatments	Adverse Events
% data SDV	100	100	100	100	100	100	100	100	100	100	100	100	100	100
% data cleaned	100	100	100	100	100	100	100	100	100	100	100	100	100	100

P 15-13

Data quality

Site	341	342	351	353	356
Number of patients screened	90	24	5	78	3
Number of patients randomised	83	23	5	73	3
Number of patients completing DB phase	56	10	5	52	1
Number of protocol violations	20	0	0	33	0

Harosa III P1513

100% data clean patients during the completed double blind period

Confidential Information

bioprojet

DSMB – HAROSA III

DEC-15 2016

1 Demography 2
2 Safety data 2
3 Cases to investigate 3

1 DEMOGRAPHY

Sex	18-60 year	61-75 year	total	%
Female	20	3	23	28%
Male	38	21	59	72%
**Total**	**58**	**24**	**82**	**100%**

Much more male patients than female. Most of them have obesity (need to have the BMI)

BW range (kg)	Female	Male	total	%
70-90	3	4	7	9%
91-120	19	41	60	73%
>120	1	14	15	18%
**total**	**23**	**59**	**82**	**100%**

With CPAP	Female	Male	total	%
No	18	37	55	67%
Yes	5	22	27	33%
**total**	**23**	**59**	**82**	**100%**

2 SAFETY DATA

No serious case and no severe case

	AE	PT	PT% (n=82)
TEAE			
Mild	10	8	10%
Moderate	18	13	16%
Total	28	19	23%
ADR			
Mild	7	6	7%
Moderate	13	9	11%
Total	20	14	17%

	TEAE	PT	PT% (n=82)
headache	9	8	10%
insomnia	3	3	4%
nervousness	2	2	2%
collapse	2	1	1%
dislipidemia	2	1	1%
blurred vision	1	1	1%
burning breast	1	1	1%
irritability	1	1	1%
lid edema	1	1	1%
nerves	1	1	1%
nightmare	1	1	1%
phlegmona	1	1	1%
purpura	1	1	1%
Single supraventricular extrasystoles.	1	1	1%
viral infection	1	1	1%
Total	28	19	23%

	ADR	PT	PT% (n=82)
headache	9	8	10%
insomnia	3	3	4%
nervousness	2	2	2%
collapse	2	1	1%
irritability	1	1	1%
nerves	1	1	1%
nightmare	1	1	1%
purpura	1	1	1%
Total	20	14	17%

Reporting of TEAE and ADR is low when compared to other studies

No signal

3 CASES TO INVESTIGATE

Subject ID	TEAE	Intensity	Outcome of Adverse Event	Age (years)	Sex/ BW	Delay	causality
341012, DB-009	Henoch–Schönlein purpura	Moderate	Recovered with sequelae	63	Male/ 102kg	5 days	Possible

Drop out (?), Medhist, What sequelae

Subject	Number	Age/BW/Sex	TEAE	Intensity	outcome
341@SUMHA ALEKSANDROVSKA	341016, DB-045	56y / 105kg/ F	Collapse during one day or less	Mild	Recovered
			collapse during one day or less	Mild	Recovered

BF2.649 P1513 Harosa III

Case summary:

Patient 4112

Demographics:

63 years old male

Medical history:

Chronic atrial fibrillation as of June 1 1993

COPD 18 Nov 2009

Allergy to sulphonamides Apr 1st 1987

Arterial hypertension June 1st 1993

Concomitant medications

Bisoprolol June 1st 1993

Digoxin June 1st 1993

Furosemide June 1st 1993

Budesonide/ formoterol Nov 18th 2009

Tiotropium Nov 18th 2009

Desloratadine May 2nd 2016

Dexophen May 2nd 2016

Summary:

Initiated pitolisant (10mg OD) on April 26th

Henoch-Schönlein purpura reported on May 1st 2016 of moderate intensity. Pitolisant was discontinued on May 2nd . Desloratadine and dexophen both antihistamines on May 2nd 2016. Patient discontinued study on May May 2nd (V3) Event resolved on May 10th 2016.

Lab results were within normal ranges on April 13th 2016 at screening

BF2.649 P1513 Harosa III

Case summary:

Pt 4116

Demographics:

56 Year-old female patient

Weight: 105 kg

Medical history:

Diabetes type 2-2003

Arterial hypertension -2008

COPD mild -05 jul 2014

Concomitant meds:

Moxonidine- 2008

Bisoprolol-2008

Metformin- 2003

Glimepiride- 2008

Liraglutide-2003

Montelukast- 5 Jul 2014

tiptropium

Lercanidipine- 2008

Summary:

Patient initiated pitolisant on 29 April 2016 at 10mg OD. Dose escalated according to protocol to 20mg OD on May 4th 2016. A 'collapse' was reported on May 7th 2016. Event was moderate and felt possibly related to the study drug.

Dose was reduced to 10mg OD . No drug prescribed for AE treatment

Another collapse was reported on May 9th 2016 (only 1 day). No corrective action was taken and patient continued in study.

Of note patient continued double blind period until the end at 10mg OD and then switched to OL with 40mg OD

HAROSA III : BF2.649/Placebo(P1513)

Meeting of the Safety Data Monitoring Committee

Meeting Minutes

Attendees

[redacted] Hospital, Lyon, France (HB)
[redacted] Saint Antoine Hospital, Paris, France
[redacted], Biostatistician, Veeda Clinical Research, Brussels Belgium (LK)
[redacted] Bioprojet, Paris, France (IL)
[redacted] Bioprojet, Paris, France (YJ)
[redacted], Bioprojet, Paris, France (KG)

Excused [redacted]

Author of the minutes [redacted]

Time and place

- **Date:** Wednesday March 8th 2017
- **Time:** 10:30 - 11:30
- **Location:** Telephonic conference

Item	Discussion Topics / Minutes	Actions/Attachments
**Agenda**	▪ Safety review<br>▪ Recommendations<br>▪ Next meeting	
**Study status**	▪ **Patients recruitment status**<br>o 202 screened<br>o 187 randomized<br>o 184 completed double blind phase<br>o 175 patients entering OL phase<br>o Around 100 patients with 100% data clean<br>LPLV double blind: February 27th 2017<br>Database lock : July 2017 (to be confirmed)<br><br>▪ **Upcoming Harosa IV study with same design**<br>o Study start 2Q2017	Two sites (41 and 53) have included 83% of patients randomized. This will be addressed in the Study Analysis Plan to assess potential interaction between site and treatment.<br><br>Several protocol violations were identified at site 41 and 53. Sites audits are being conducted at both sites.
**Review of Safety Data**	▪ **Safety review**<br>No SAEs or severe AEs reported. Very few AEs and TEAEs (70) reported. For only 25% of patients and 19% TEAEs related to study drug.<br><br>o Tables and listings for 186 patients were reviewed (Annex 1):<br>o An analysis of data was provided by PPD (Annex 2)<br><br>o AEs were reviewed regardless of data cleaness status<br><br>o Cases summaries additional information was reviewed for Pts 4112 and 4116<br><br>o ECGs data and graphs were provided for all patients	▪ Bioprojet to provide additional information on<br>o AEs reported for Pt. 4116 were hypotonia was coded instead of collapse. Details are requested on hypotonia<br>o Nerves should be coded as nervousness as clarified by monitors<br>o Patient 4133 who had abnormal ECGs during open label phase.
**Recommendation**	On the basis of the provided information no change to the study is recommended	
**Next meeting**	Next meeting: TC on Wednesday September 13th at 10.30 AM	

Demographics Vitals

Subject ID	Age (years)	Sex	Weight (kg)	BMI (kg/m2)	BP Sys (mmHg)	BP Dia (mmHg)	HR (beats/min)	nCPAP Therapy Status	Completed visit 7 and 100% SDV
341001, DB-001	56	Male	102	32,9	130	80	64	No	Yes
341002, DB-002	55	Female	70	27,7	130	80	72	No	Yes
341003, DB-003	59	Male	101	36,7	120	75	62	Yes	No
341004, DB-004	41	Female	94	30,7	120	70	61	No	Yes
341005, DB-005	47	Female	98	34,7	120	80	76	No	Yes
341006, DB-007	67	Male	92	31,8	127	85	75	No	Yes
341007, DB-011	37	Male	105	34,3	130	80	67	No	Yes
341008, DB-006	50	Female	100	33,4	130	80	67	No	Yes
341009, DB-008	57	Male	133	39,3	130	80	72	Yes	Yes
341010, DB-043	60	Male	120	37,9	120	80	78	No	Yes
341011, DB-012	69	Male	98	36,4	130	80	92	Yes	Yes
341012, DB-009	63	Male	102	32,9	135	82	88	No	No
341013, DB-010	40	Male	102	32,9	120	80	76	No	No
341014, DB-044	60	Male	101	34,9	125	80	77	Yes	Yes
341015, DB-048	48	Male	120	39,2	120	85	80	Yes	Yes
341016, DB-045	56	Female	105	39,5	110	80	76	No	No
341017, DB-046	38	Male	86	28,7	120	70	85	No	No
341018, DB-047	40	Female	95	32,9	120	80	67	No	Yes
341019, DB-060	51	Male	120	36,2	115	70	70	Yes	No
341020, DB-055	57	Male	105	38,6	125	83	74	No	Yes
341021, DB-056	58	Female	85	31,2	120	80	71	No	No
341022, DB-059	33	Male	125	38,6	123	80	86	Yes	Yes
341023, DB-057	42	Male	100	33,8	130	80	75	Yes	Yes
341024, DB-058	65	Female	100	32,7	130	80	76	No	Yes
341026, DB-066	65	Male	103	34,8	130	75	66	Yes	Yes
341027, DB-063	25	Male	128	37,8	110	70	77	No	Yes
341028, DB-061	58	Male	108	35,7	125	75	68	Yes	No
341029, DB-064	60	Male	102	34,5	125	80	72	Yes	No
341031, DB-065	34	Male	85	28,7	120	70	57	Yes	No
341032, DB-067	63	Male	115	37,1	125	85	75	No	No
341033, DB-069	55	Male	120	37	125	75	81	Yes	No
341034, DB-068	57	Female	100	37,2	130	82	74	Yes	No
341037, DB-062	66	Male	98	36,4	130	75	64	Yes	No
341038, DB-071	52	Male	95	32,9	130	80	62	No	No
341039, DB-111	55	Male	[illegible]	38,1	125	75	68	Yes	No
341040, DB-070	64	Female	100	39,1	142	85	58	Yes	No

HAROSA III

G:\StudData\BalkanTrials\BAL001\CDM\DSMB Output v2.sas ececuted by mba 03MAR2017 13:10

Annex 1

Subject ID	Age (years)	Sex	Weight (kg)	BMI (kg/m2)	BP Sys (mmHg)	BP Dia (mmHg)	HR (beats/min)	nCPAP Therapy Status	Completed visit 7 and 100% SDV
341041, DB-104	64	Female	98	39,3	133	82	68	Yes	No
341042, DB-072	64	Male	130	37,6	140	84	94	Yes	No
341043, DB-105	36	Male	110	38,1	120	75	69	No	No
341044, DB-106	41	Female	90	33,1	125	78	64	No	No
341045, DB-108	63	Male	110	34,7	125	75	76	No	No
341046, DB-116	24	Male	126	38,9	130	80	99	No	No
341048, DB-107	40	Female	90	31,5	120	78	60	No	No
341049, DB-114	54	Male	110	35,1	120	75	82	Yes	No
341050, DB-103	61	Male	93	34,2	140	90	69	No	No
341051, DB-118	60	Female	110	35,1	120	80	60	Yes	No
341052, DB-117	41	Female	108	36,1	130	80	100	No	No
341053, DB-119	45	Female	110	38,5	120	80	74	Yes	No
341054, DB-110	63	Female	85	36,3	125	80	61	Yes	No
341055, DB-112	66	Female	109	39,1	140	85	84	Yes	No
341057, DB-121	42	Male	120	39,2	130	80	76	No	No
341058, DB-113	48	Male	110	34	135	85	72	Yes	No
341059, DB-115	41	Male	64	36,2	115	70	93	Yes	No
341060, DB-109	57	Male	94	38,6	115	75	93	Yes	No
341061, DB-120	62	Male	90	31,9	120	75	70	No	No
341062, DB-123	53	Male	102	32,2	140	82	84	Yes	No
341063, DB-122	57	Female	90	33,5	120	75	85	No	No
341064, DB-207	51	Male	89	32,7	130	83	76	No	No
341065, DB-193	49	Male	97	28	130	80	68	Yes	No
341066, DB-125	59	Male	105	36,3	110	70	60	Yes	No
341067, DB-194	59	Male	125	38,6	120	80	89	Yes	No
341068, DB-124	49	Male	96	36,1	132	80	65	Yes	No
341069, DB-126	57	Female	86	31,6	134	79	64	Yes	No
341070, DB-198	60	Male	108	31,2	138	82	76	Yes	No
341071, DB-197	36	Female	79	28,3	120	75	81	Yes	No
341072, DB-196	40	Male	135	37,4	132	80	100	Yes	No
341073, DB-200	57	Male	115	36,3	130	80	49	Yes	No
341074, DB-202	40	Female	96	32,4	115	70	90	Yes	No
341075, DB-215	48	Male	98	33,5	118	74	69	Yes	No
341076, DB-209	53	Male	125	37,7	120	85	77	Yes	No
341077, DB-199	58	Male	105	36,8	135	[illegible]	[illegible]	Yes	No
341078, DB-201	63	Female	90	33,5	120	85	72	Yes	No

Demo vitals

Demo Vitals

Subject ID	Age (years)	Sex	Weight (kg)	BMI (kg/m2)	BP Sys (mmHg)	BP Dia (mmHg)	HR (beats/min)	nCPAP Therapy Status	Completed visit 7 and 100% SDV
341080, DB-211	36	Male	105	34,3	120	82	80	Yes	No
341081, DB-208	49	Male	140	39,2	130	80	76	Yes	No
341082, DB-195	49	Male	111	38,4	135	85	78	Yes	No
341083, DB-206	52	Male	100	30,9	138	90	86	Yes	No
341084, DB-214	50	Female	92	33,4	130	80	84	Yes	No
341085, DB-204	67	Male	97	34,4	132	80	78	Yes	No
341087, DB-210	65	Male	108	36,5	135	82	87	Yes	No
341088, DB-213	58	Male	125	39,5	130	75	60	Yes	No
341089, DB-203	50	Male	109	38,2	125	74	77	Yes	No
341090, DB-212	71	Male	108	35,3	120	82	68	Yes	No
342001, DB-133	50	Male	95	30,7	130	85	90	No	Yes
342002, DB-138	38	Male	126	39,8	130	80	59	No	Yes
342003, DB-137	61	Male	100	39,1	125	75	60	No	Yes
342004, DB-134	55	Male	112	34,6	130	80	72	Yes	Yes
342005, DB-136	64	Male	97	33,2	125	80	73	Yes	Yes
342006, DB-139	52	Male	126	36,4	130	85	70	Yes	Yes
342007, DB-141	30	Male	87	25,1	120	75	75	Yes	Yes
342008, DB-142	61	Male	90	31,1	130	90	71	Yes	Yes
342009, DB-135	61	Female	70	24,8	135	80	70	No	Yes
342010, DB-144	63	Male	116	36,6	130	85	67	Yes	Yes
342011, DB-140	50	Male	72	23,8	120	80	70	Yes	Yes
342012, DB-143	42	Male	100	33,4	130	80	73	No	Yes
342013, DB-145	56	Male	80	30,9	130	80	71	Yes	Yes
342014, DB-146	61	Male	88	29,7	130	85	74	Yes	Yes
342015, DB-147	60	Female	93	34,6	130	85	70	No	Yes
342016, DB-148	44	Male	118	37,2	130	80	64	Yes	Yes
342017, DB-150	46	Female	110	38,1	140	80	74	Yes	Yes
342018, DB-149	53	Female	65	26	125	70	78	Yes	Yes
342019, DB-152	47	Male	100	34,6	140	85	78	Yes	No
42020, DB-151	54	Female	43	21,9	110	70	72	Yes	No
42021, DB-153	43	Male	130	38	130	85	78	Yes	Yes
42022, DB-155	58	Male	128	37,4	135	90	60	Yes	Yes
42023, DB-154	35	Male	73	23,8	130	80	66	Yes	Yes
51001, DB-013	57	Male	110	38,1	125	80	85	Yes	Yes
51002, DB-014	41	Male	[illegible]	[illegible]	130	80	64	Yes	Yes
51003, DB-015	58	Female	96	38,5	130	85	70	Yes	Yes

Subject ID	Age (years)	Sex	Weight (kg)	BMI (kg/m2)	BP Sys (mmHg)	BP Dia (mmHg)	HR (beats/min)	nCPAP Therapy Status	Completed visit 7 and 100% SDV
351004, DB-073	62	Male	100	31,6	125	80	63	Yes	Yes
351005, DB-074	59	Male	113	36,9	130	85	63	No	Yes
353001, DB-025	70	Male	110	39,9	125	80	77	Yes	Yes
353002, DB-026	40	Male	115	36,3	120	70	75	No	Yes
353004, DB-027	60	Male	130	39,7	130	80	87	No	Yes
353006, DB-029	46	Male	120	39,2	130	80	75	No	Yes
353007, DB-028	61	Male	118	39,9	120	75	67	Yes	Yes
353008, DB-033	68	Male	120	39,6	125	75	71	No	Yes
353009, DB-037	55	Male	102	32,9	140	80	74	No	Yes
353010, DB-035	52	Male	100	37,6	130	80	90	No	Yes
353011, DB-030	55	Female	105	39,5	140	90	96	No	Yes
353012, DB-034	53	Male	125	39,5	130	75	86	No	Yes
353013, DB-039	54	Male	114	37,7	140	75	88	No	Yes
353014, DB-031	72	Female	70	27,3	120	70	71	No	Yes
353015, DB-036	51	Male	110	33,2	125	80	70	No	Yes
353016, DB-032	71	Male	130	38	140	80	73	No	Yes
353017, DB-038	71	Male	76	26,3	120	80	66	Yes	Yes
353018, DB-050	53	Female	95	37,1	120	80	77	No	Yes
353019, DB-040	62	Male	99	35,9	135	80	87	No	Yes
353020, DB-041	59	Male	100	39,1	135	80	60	No	Yes
353021, DB-042	52	Male	114	39,4	120	70	74	No	Yes
353022, DB-051	59	Female	100	39,1	135	80	93	No	Yes
353023, DB-049	44	Male	95	30	120	70	85	No	Yes
53024, DB-079	57	Female	96	36,1	135	80	96	Yes	Yes
53025, DB-053	45	Female	114	38,1	130	75	64	No	Yes
53026, DB-054	46	Male	115	35,5	120	75	73	No	Yes
53027, DB-080	68	Male	110	39,4	140	80	84	Yes	Yes
53028, DB-081	61	Male	120	39,2	135	80	108	Yes	Yes
53029, DB-052	51	Male	110	39,4	125	80	62	Yes	Yes
53030, DB-082	66	Male	115	39,8	130	80	85	Yes	Yes
53031, DB-083	58	Female	106	38,9	125	70	68	Yes	Yes
53032, DB-084	59	Male	135	39,4	120	80	82	Yes	Yes
53033, DB-085	63	Female	93	39,2	130	80	85	Yes	Yes
53035, DB-089	58	Male	86	28,1	130	85	74	No	Yes
53036, DB-091	55	Male	[illegible]	[illegible]	[illegible]	[illegible]	[illegible]	[illegible]	Yes
53037, DB-090	42	Male	130	37,6	135	80	70	No	Yes

Demo
Vitalo

Subject ID	Age (years)	Sex	Weight (kg)	BMI (kg/m2)	BP Sys (mmHg)	BP Dia (mmHg)	HR (beats/min)	nCPAP Therapy Status	Completed visit 7 and 100% SDV
353038, DB-088	38	Male	94	32,5	110	70	80	No	Yes
353039, DB-094	56	Female	115	39,8	140	80	88	No	Yes
353040, DB-092	40	Female	117	39,5	140	80	78	No	Yes
353041, DB-086	62	Male	101	35,4	130	70	64	No	Yes
353042, DB-087	56	Male	90	31,1	120	70	52	No	Yes
353043, DB-096	37	Male	139	38,5	140	80	79	No	Yes
353044, DB-098	48	Male	120	39,2	120	80	86	Yes	Yes
353045, DB-093	37	Male	129	39,8	130	85	93	No	Yes
353046, DB-100	47	Male	125	39,5	130	80	93	Yes	Yes
353047, DB-097	21	Female	122	39,4	120	80	69	No	Yes
353048, DB-095	66	Male	121	39,1	130	80	75	No	Yes
353049, DB-099	61	Male	100	34,6	130	80	61	No	Yes
353050, DB-101	64	Male	110	36,3	130	80	77	No	Yes
353051, DB-102	47	Male	115	38,9	140	80	87	No	Yes
353052, DB-163	52	Female	92	35,9	125	70	66	No	Yes
353053, DB-164	56	Female	100	39,6	140	80	63	Yes	Yes
353054, DB-165	52	Female	82	28,4	125	80	93	No	Yes
353055, DB-166	47	Female	93	32,2	120	80	75	No	Yes
353057, DB-167	40	Male	131	38,3	130	80	71	No	Yes
353058, DB-168	48	Male	113	36,5	130	75	80	Yes	Yes
353059, DB-176	53	Male	112	35,3	120	80	74	Yes	No
353060, DB-171	53	Male	112	39,7	135	80	68	No	Yes
353061, DB-180	52	Male	102	32,6	120	80	73	Yes	Yes
353062, DB-181	45	Male	95	29,3	130	75	76	Yes	Yes
353063, DB-177	38	Male	132	39	120	80	82	Yes	Yes
353064, DB-174	67	Female	120	39,6	135	80	100	No	Yes
353065, DB-179	31	Male	106	34,6	120	80	111	No	Yes
353067, DB-169	59	Male	85	30,1	140	80	85	No	Yes
353068, DB-178	51	Male	111	38,4	120	80	64	No	Yes
53069, DB-170	59	Male	106	33,1	130	85	68	No	Yes
53070, DB-175	54	Male	100	34,6	120	70	69	No	No
53071, DB-172	56	Female	105	36,8	120	75	56	Yes	Yes
53072, DB-173	57	Male	116	38,8	135	80	77	Yes	Yes
53073, DB-183	66	Male	86	29,1	130	70	80	Yes	Yes
53074, DB-184	48	Male	103	32,9	120	80	68	Yes	Yes
53075, DB-185	60	Male	90	29,1	110	70	70	Yes	Yes

Vitals
Demo

Demo
Vitals

Subject ID	Age (years)	Sex	Weight (kg)	BMI (kg/m2)	BP Sys (mmHg)	BP Dia (mmHg)	HR (beats/min)	nCPAP Therapy Status	Completed visit 7 and 100% SDV
353076, DB-186	56	Male	110	34	120	80	65	Yes	No
353077, DB-187	45	Male	100	36,7	130	75	81	Yes	No
353078, DB-182	62	Male	129	38,9	125	75	63	Yes	No
356001, DB-127	55	Male	130	38,4	140	90	85	No	Yes
356002, DB-128	61	Male	128	39,1	145	85	86	No	Yes
356005, DB-129	38	Male	126	39,3	135	80	59	Yes	Yes

Site name	Subject ID	Reported Term for the Adverse Event	Outcome of Adverse Event	Age (years)	Sex	First day of treatment	Start Date/Time of Adverse Event	Causality	Completed visit 7 and 100% SDV
No patients.									

No SAEs
No SARs
No Severe AEs

TEAEs .

Subject ID	Reported Term for the Adverse Event	Outcome of Adverse Event	Age (years)	Sex	First day of treatment	Start Date/Time of Adverse Event	Causality	Completed visit 7 and 100% SDV
341003, DB-003	acute viral infection	Recovered	59	Male	2016-04-21	2016-08-08	Unlikely related	No
341003, DB-003	nervous tension	Recovered	59	Male	2016-04-21	2016-05-06	Likely related	No
341003, DB-003	insomnia	Recovered	59	Male	2016-04-21	2016-11-20	Likely related	No
341007, DB-011	headache	Recovered	37	Male	2016-04-26	2016-05-04	Likely related	Yes
341011, DB-012	headache	Recovered	69	Male	2016-04-26	2016-08-16	Likely related	Yes
341012, DB-009	Henoch–Schönlein purpura	Recovered with sequelae	63	Male	2016-04-26	2016-05-01	Possibly related	No
341013, DB-010	headache	Recovered	40	Male	2016-04-26	2016-05-11	Possibly related	No
341013, DB-010	anxiety	Recovered	40	Male	2016-04-26	2016-05-11	Possibly related	No
341014, DB-044	nerves	Recovered	60	Male	2016-04-27	2016-05-05	Possibly related	Yes
341016, DB-045	hypotonia	Recovered	56	Female	2016-04-28	2016-05-07	Possibly related	No
341016, DB-045	hypotonia	Recovered	56	Female	2016-04-28	2016-05-09	Possibly related	No
341020, DB-055	headache	Recovered	57	Male	2016-05-27	2016-06-12	Possibly related	Yes
341020, DB-055	insomnia	Recovered	57	Male	2016-05-27	2016-09-10	Possibly related	Yes
341021, DB-056	headache	Recovered	58	Female	2016-05-27	2016-06-12	Possibly related	No
341021, DB-056	headache	Recovered	58	Female	2016-05-27	2016-09-15	Possibly related	No
341027, DB-063	vertigo	Recovered	25	Male	2016-06-22	2016-10-06	Likely related	Yes
341028, DB-061	nervous tension	Recovered	58	Male	2016-06-21	2016-09-29	Likely related	No
341028, DB-061	stomach pain	Recovered	58	Male	2016-06-21	2016-12-12	Unlikely related	No
341029, DB-064	nervousness	Recovered	60	Male	2016-06-22	2016-10-06	Likely related	No
341031, DB-065	vertigo	Recovered	34	Male	2016-06-22	2016-06-30	Possibly related	No
341031, DB-065	insomnia	Recovered	34	Male	2016-06-22	2016-10-10	Likely related	No
341033, DB-069	headache	Recovered	55	Male	2016-06-29	2016-07-06	Possibly related	No
341033, DB-069	insomnia	Recovered	55	Male	2016-06-29	2016-07-07	Possibly related	No
341033, DB-069	diarrhea	Recovered	55	Male	2016-06-29	2016-08-17	Unlikely related	No
341033, DB-069	sinus tachycardia	Recovered	55	Male	2016-06-29	2016-10-19	Possibly related	No
341033, DB-069	sinus tachycardia	Recovered	55	Male	2016-06-29	2016-10-23	Possibly related	No
341034, DB-068	Vertigo	Recovered	57	Female	2016-06-28	2016-07-05	Possibly related	No
341034, DB-068	Vertigo	Recovered	57	Female	2016-06-28	2016-10-07	Possibly related	No
341037, DB-062	headache	Recovered	66	Male	2016-06-21	2016-09-28	Likely related	No
341037, DB-062	vertigo	Recovered	66	Male	2016-06-21	2016-06-30	Likely related	No
341038, DB-071	vomiting	Recovered	52	Male	2016-07-07	2017-01-14	Unlikely related	No
341043, DB-105	Insomnia	Recovered	36	Male	2016-07-12	2016-10-20	Unlikely related	No
341045, DB-108	nausea	Recovered	63	Male	2016-07-13	2016-07-21	Possibly related	No
341045, DB-108	insomnia	Recovered	63	Male	2016-07-13	2016-07-21	Possibly related	No
41046, DB-116	flu	Recovered	24	Male	2016-07-22	2017-01-04	Unlikely related	No
41054, DB-110	insomnia	Recovered	63	Female	2016-07-15	2016-08-01	Possibly related	No
41058, DB-113	insomnia	Recovered	48	Male	2016-07-18	2016-07-26	Possibly related	No

Subject ID	Reported Term for the Adverse Event TEAES	Outcome of Adverse Event	Age (years)	Sex	First day of treatment	Start Date/Time of Adverse Event	Causality	Completed visit 7 and 100% SDV
341059, DB-115	vertigo	Recovered	41	Male	2016-07-21	2016-11-02	Likely related	No
341060, DB-109	herpes zoster - left choulder	Recovered with sequelae	57	Male	2016-07-14	2016-07-28	Unlikely related	No
341060, DB-109	headache	Recovered	57	Male	2016-07-14	2016-11-03	Likely related	No
341066, DB-125	Hypertensive crisis	Recovered	59	Male	2016-09-26	2016-12-15	Unlikely related	No
341066, DB-125	heartburn	Recovered	59	Male	2016-09-26	2017-01-14	Unlikely related	No
341066, DB-125	insomnia	Recovered	59	Male	2016-09-26	2017-01-11	Possibly related	No
341068, DB-124	headache	Recovered	49	Male	2016-09-21	2017-01-11	Likely related	No
341069, DB-126	nervousness	Recovered	57	Female	2016-09-26	2017-01-10	Likely related	No
341071, DB-197	headache	Recovered	36	Female	2016-10-27	2017-02-08	Likely related	No
341072, DB-196	anxiety	Recovered	40	Male	2016-10-27	2017-02-08	Likely related	No
341077, DB-199	insomnia	Recovered	58	Male	2016-10-28	2017-02-11	Likely related	No
341078, DB-201	Flu	Recovered	63	Female	2016-10-31	2017-02-16	Unlikely related	No
341084, DB-214	impaired arterial hypertension	Recovered	50	Female	2016-11-10	2016-11-19	Possibly related	No
341087, DB-210	anxiety	Recovered	65	Male	2016-11-04	2017-02-16	Likely related	No
351003, DB-015	Worsening of existing Dislipidaemia	Not yet recovered	58	Female	2016-05-10	2016-08-02	Unlikely related	Yes
351003, DB-015	Worsening of exiting Dislipidaemia	Recovered	58	Female	2016-05-10	2016-08-02	Unlikely related	Yes
353007, DB-028	Impaired vision	Recovered	61	Male	2016-04-27	2016-07-28	Unlikely related	Yes
353007, DB-028	Edemas under your eyes	Recovered	61	Male	2016-04-27	2016-07-28	Unlikely related	Yes
353009, DB-037	burning breasts- bit accident	Not yet recovered	55	Male	2016-05-30	2016-09-17	Unlikely related	Yes
353016, DB-032	Irritability	Recovered	71	Male	2016-04-28	2016-05-13	Likely related	Yes
353021, DB-042	Headache	Recovered	52	Male	2016-05-31	2016-06-08	Likely related	Yes
353021, DB-042	Sleep disturbance, nightmares	Recovered	52	Male	2016-05-31	2016-06-09	Likely related	Yes
353033, DB-085	headache	Recovered	63	Female	2016-07-02	2016-07-17	Likely related	Yes
353033, DB-085	Insomnia	Recovered	63	Female	2016-07-02	2016-10-16	Likely related	Yes
353035, DB-089	Insomnia	Recovered	58	Male	2016-07-04	2016-10-18	Likely related	Yes
353035, DB-089	Insomnia	Recovered	58	Male	2016-07-04	2016-10-27	Likely related	Yes
353041, DB-086	headache	Recovered	62	Male	2016-07-02	2016-10-16	Likely related	Yes
353045, DB-093	Phlegmona femoris dextra	Recovered	37	Male	2016-07-05	2016-10-03	Unlikely related	Yes
353048, DB-095	Single supraventricular extrasystoles.	Recovered	66	Male	2016-07-06	2016-08-24	Unlikely related	Yes
353070, DB-175	hypertensive crisis	Recovered	54	Male	2016-09-15	2016-12-10	Unlikely related	No
353071, DB-172	insomnia	Recovered	56	Female	2016-09-12	2016-12-27	Likely related	Yes
353072, DB-173	insomnia	Recovered	57	Male	2016-09-13	2016-09-28	Likely related	Yes
353072, DB-173	insomnia	Recovered	57	Male	2016-09-13	2016-12-28	Likely related	Yes

Subject ID	Reported Term for the Adverse Event (AEs)	Outcome of Adverse Event	Age (years)	Sex	First day of treatment	Start Date/Time of Adverse Event	Causality	Completed visit 7 and 100% SDV
341003, DB-003	acute viral infection	Recovered	59	Male	2016-04-21	2016-08-08	Unlikely related	No
341003, DB-003	nervous tension	Recovered	59	Male	2016-04-21	2016-05-06	Likely related	No
341003, DB-003	insomnia	Recovered	59	Male	2016-04-21	2016-11-20	Likely related	No
341007, DB-011	headache	Recovered	37	Male	2016-04-26	2016-05-04	Likely related	Yes
341011, DB-012	headache	Recovered	69	Male	2016-04-26	2016-08-16	Likely related	Yes
341012, DB-009	Henoch–Schönlein purpura	Recovered with sequelae	63	Male	2016-04-26	2016-05-01	Possibly related	No
341013, DB-010	headache	Recovered	40	Male	2016-04-26	2016-05-11	Possibly related	No
341013, DB-010	anxiety	Recovered	40	Male	2016-04-26	2016-05-11	Possibly related	No
341014, DB-044	nerves	Recovered	60	Male	2016-04-27	2016-05-05	Possibly related	Yes
341016, DB-045	hypotonia	Recovered	56	Female	2016-04-28	2016-05-07	Possibly related	No
341016, DB-045	hypotonia	Recovered	56	Female	2016-04-28	2016-05-09	Possibly related	No
341020, DB-055	headache	Recovered	57	Male	2016-05-27	2016-06-12	Possibly related	Yes
341020, DB-055	insomnia	Recovered	57	Male	2016-05-27	2016-09-10	Possibly related	Yes
341021, DB-056	headache	Recovered	58	Female	2016-05-27	2016-06-12	Possibly related	No
341021, DB-056	headache	Recovered	58	Female	2016-05-27	2016-09-15	Possibly related	No
341027, DB-063	vertigo	Recovered	25	Male	2016-06-22	2016-10-06	Likely related	Yes
341028, DB-061	nervous tension	Recovered	58	Male	2016-06-21	2016-09-29	Likely related	No
341028, DB-061	stomach pain	Recovered	58	Male	2016-06-21	2016-12-12	Unlikely related	No
341029, DB-064	nervousness	Recovered	60	Male	2016-06-22	2016-10-06	Likely related	No
341031, DB-065	vertigo	Recovered	34	Male	2016-06-22	2016-06-30	Possibly related	No
341031, DB-065	insomnia	Recovered	34	Male	2016-06-22	2016-10-10	Likely related	No
341033, DB-069	headache	Recovered	55	Male	2016-06-29	2016-07-06	Possibly related	No
341033, DB-069	insomnia	Recovered	55	Male	2016-06-29	2016-07-07	Possibly related	No
341033, DB-069	diarrhea	Recovered	55	Male	2016-06-29	2016-08-17	Unlikely related	No
341033, DB-069	sinus tachycardia	Recovered	55	Male	2016-06-29	2016-10-19	Possibly related	No
341033, DB-069	sinus tachycardia	Recovered	55	Male	2016-06-29	2016-10-23	Possibly related	No
341034, DB-068	Vertigo	Recovered	57	Female	2016-06-28	2016-07-05	Possibly related	No
341034, DB-068	Vertigo	Recovered	57	Female	2016-06-28	2016-10-07	Possibly related	No
341037, DB-062	headache	Recovered	66	Male	2016-06-21	2016-09-28	Likely related	No
341037, DB-062	vertigo	Recovered	66	Male	2016-06-21	2016-06-30	Likely related	No
341038, DB-071	vomiting	Recovered	52	Male	2016-07-07	2017-01-14	Unlikely related	No
341043, DB-105	Insomnia	Recovered	36	Male	2016-07-12	2016-10-20	Unlikely related	No
341045, DB-108	nausea	Recovered	63	Male	2016-07-13	2016-07-21	Possibly related	No
341045, DB-108	insomnia	Recovered	63	Male	2016-07-13	2016-07-21	Possibly related	No
341046, DB-116	flu	Recovered	24	Male	2016-07-22	2017-01-04	Unlikely related	No
341054, DB-110	insomnia	Recovered	63	Female	2016-07-15	2016-08-01	Possibly related	No
341058, DB-113	insomnia	Recovered	48	Male	2016-07-18	2016-07-26	Possibly related	No

Subject ID	Reported Term for the Adverse Event<br>AES	Outcome of Adverse Event	Age (years)	Sex	First day of treatment	Start Date/Time of Adverse Event	Causality	Completed visit 7 and 100% SDV
341059, DB-115	vertigo	Recovered	41	Male	2016-07-21	2016-11-02	Likely related	No
341060, DB-109	herpes zoster - left choulder	Recovered with sequelae	57	Male	2016-07-14	2016-07-28	Unlikely related	No
341060, DB-109	headache	Recovered	57	Male	2016-07-14	2016-11-03	Likely related	No
341066, DB-125	Hypertensive crisis	Recovered	59	Male	2016-09-26	2016-12-15	Unlikely related	No
341066, DB-125	heartburn	Recovered	59	Male	2016-09-26	2017-01-14	Unlikely related	No
341066, DB-125	insomnia	Recovered	59	Male	2016-09-26	2017-01-11	Possibly related	No
341068, DB-124	headache	Recovered	49	Male	2016-09-21	2017-01-11	Likely related	No
341069, DB-126	nervousness	Recovered	57	Female	2016-09-26	2017-01-10	Likely related	No
341071, DB-197	headache	Recovered	36	Female	2016-10-27	2017-02-08	Likely related	No
341072, DB-196	anxiety	Recovered	40	Male	2016-10-27	2017-02-08	Likely related	No
341077, DB-199	insomnia	Recovered	58	Male	2016-10-28	2017-02-11	Likely related	No
341078, DB-201	Flu	Recovered	63	Female	2016-10-31	2017-02-16	Unlikely related	No
341084, DB-214	impaired arterial hypertension	Recovered	50	Female	2016-11-10	2016-11-19	Possibly related	No
341087, DB-210	anxiety	Recovered	65	Male	2016-11-04	2017-02-16	Likely related	No
351003, DB-015	Worsening of existing Dislipidaemia	Not yet recovered	58	Female	2016-05-10	2016-08-02	Unlikely related	Yes
351003, DB-015	Worsening of exiting Dislipidaemia	Recovered	58	Female	2016-05-10	2016-08-02	Unlikely related	Yes
353007, DB-028	Impaired vision	Recovered	61	Male	2016-04-27	2016-07-28	Unlikely related	Yes
353007, DB-028	Edemas under your eyes	Recovered	61	Male	2016-04-27	2016-07-28	Unlikely related	Yes
353009, DB-037	burning breasts- bit accident	Not yet recovered	55	Male	2016-05-30	2016-09-17	Unlikely related	Yes
353016, DB-032	Irritability	Recovered	71	Male	2016-04-28	2016-05-13	Likely related	Yes
353021, DB-042	Headache	Recovered	52	Male	2016-05-31	2016-06-08	Likely related	Yes
353021, DB-042	Sleep disturbance, nightmares	Recovered	52	Male	2016-05-31	2016-06-09	Likely related	Yes
353033, DB-085	headache	Recovered	63	Female	2016-07-02	2016-07-17	Likely related	Yes
353033, DB-085	Insomnia	Recovered	63	Female	2016-07-02	2016-10-16	Likely related	Yes
353035, DB-089	Insomnia	Recovered	58	Male	2016-07-04	2016-10-18	Likely related	Yes
353035, DB-089	Insomnia	Recovered	58	Male	2016-07-04	2016-10-27	Likely related	Yes
353041, DB-086	headache	Recovered	62	Male	2016-07-02	2016-10-16	Likely related	Yes
353045, DB-093	Phlegmona femoris dextra	Recovered	37	Male	2016-07-05	2016-10-03	Unlikely related	Yes
353048, DB-095	Single supraventricular extrasystoles.	Recovered	66	Male	2016-07-06	2016-08-24	Unlikely related	Yes
53070, DB-175	hypertensive crisis	Recovered	54	Male	2016-09-15	2016-12-10	Unlikely related	No
53071, DB-172	insomnia	Recovered	56	Female	2016-09-12	2016-12-27	Likely related	Yes
53072, DB-173	insomnia	Recovered	57	Male	2016-09-13	2016-09-28	Likely related	Yes
53072, DB-173	insomnia	Recovered	57	Male	2016-09-13	2016-12-28	Likely related	Yes

Site	341	342	351	353	356
Number of patients screened	83	23	5	73	3
Number of patients randomised	82	23	5	73	3
Number of patients completing DB phase	75	21	5	73	3
Number of protocol violations	174	19	2	65	0

Study ?1513
Performance

Visit/Form	Visit 1	Visit 2	Visit 3	Visit 4	Visit 5	Visit 6	Visit 7	Visit 1-7	Phone contact 1	Phone contact 2	Patient Information	Medical / Surgical History	Prior and Concomitant Treatments	Adverse Events
% data SDV	80,4	78,3	78,3	78,4	78,1	76,7	72	77,1	79,7	75,7	79,7	71,8	70	44,9
% data cleaned	65	68,4	70,6	74	68,9	66,1	66,1	44,1	79,7	75,1	79,7	78,9	75,2	37,8

Confidential Information

bioprojet

PHARMA

Harosa III

DSMB Mar-08 2017

Annex 2

This document strictly confidential is the property of BIOPROJET. Its diffusion without its prior consent is strictly forbidden

1 DEMOGRAPHY

Age range	Female	Male	Total
18-65y	44	126	170
66-75y	3	13	16
**Total**	**47**	**139**	**186**

A majority of men with obesity class I and II has been included. This is not unusual in clinical trials in this disease.

BMI	Female	Male	Total
normal	2	2	4
overweight	5	10	15
obesity class I	15	42	57
obesity class II	25	85	110
**Total**	**47**	**139**	**186**

Category	BMI (kg/m^2)	
	from	to
Very severely underweight		15
Severely underweight	15	16
Underweight	16	18.5
Normal (healthy weight)	18.5	25
Overweight	25	30
Obese Class I (Moderately obese)	30	35
Obese Class II (Severely obese)	35	40
Obese Class III (Very severely obese)	40	

Site	341	342	351	353	356	total
screened	83 (44%)	23 (12%)	5 (2%)	73 (39%)	3 (1%)	187 (1%)
randomised	82 (44%)	23 (12%)	5 (2%)	73 (39%)	3 (1%)	186 (1%)
completing DB phase	75 (42%)	21 (11%)	5 (2%)	73 (41%)	3 (1%)	177 (1%)
protocol violations	174 (66%)	19 (7%)	2 (1%)	65 (25%)	0 (0%)	260 (1%)

At beginning of study there were issues encountered with study drug. Labels had to be corrected and bottles of 5mg pitolisant (active or placebo) had to be removed due to a manufacturing issue. Therefore several patients had their randomization visit delayed beyond the protocol allowed limits. Nevertheless an audit was performed at site 53 on March 1-2 and an other audit is scheduled on March 9-10th 2017 at site 41.

Two sites out of 5 have included a majority of the patients. We may want to examine the potential site effect produced.

Site	341-353
screened	156 (83%)
randomised	155 (83%)
completing DB phase	148 (83%)
protocol violations	239 (91%)

2 TEAE

	AE	Patients n=186	Patients%
TEAE	70	46	25%
Severe TEAE	0		
SAE	0		
TEAE related	52	36	19%

No SAE or severe TEAE

Only 25% of patients reported TEAE (25%) and 19% a TEAE related to the study drug. This is showing a very good safety profile.

The number of TEAE and patients reporting TEAE are given in function of the causality in the tables below:

All TEAE	TEAE	Patients (n=186)	% Patient
insomnia	16	14	7,5%
headache	14	13	7,0%
vertigo	6	5	2,7%
anxiety	3	3	1,6%
nervousness	2	2	1,1%
nervous tension	2	2	1,1%
flu	2	2	1,1%
Hypertensive crisis	2	2	1,1%
hypotonia	2	1	0,5%
sinus tachycardia	2	1	0,5%
nerves	1	1	0,5%
burning breasts- bit accident	1	1	0,5%
herpes zoster - left choulder	1	1	0,5%
heartburn	1	1	0,5%
nausea	1	1	0,5%
Single supraventricular extrasystoles.	1	1	0,5%
Henoch–Schönlein purpura	1	1	0,5%
Sleep disturbance, nightmares	1	1	0,5%
Phlegmona femoris dextra	1	1	0,5%
diarrhea	1	1	0,5%
Worsening of existing Dislipidaemia	1	1	0,5%
impaired arterial hypertension	1	1	0,5%
stomach pain	1	1	0,5%
Impaired vision	1	1	0,5%
vomiting	1	1	0,5%
Worsening of exiting Dislipidaemia	1	1	0,5%
Edemas under your eyes	1	1	0,5%
Irritability	1	1	0,5%
acute viral infection	1	1	0,5%
**Total**	**70**	**46**	**24,7%**

Unrelated TEAE	TEAE	Patients (n=186)	% Patient
flu	2	2	1,1%
Hypertensive crisis	2	2	1,1%
acute viral infection	1	1	0,5%
burning breasts- bit accident	1	1	0,5%
diarrhea	1	1	0,5%
Edemas under your eyes	1	1	0,5%
heartburn	1	1	0,5%
herpes zoster - left choulder	1	1	0,5%
Impaired vision	1	1	0,5%
insomnia	1	1	0,5%
Phlegmona femoris dextra	1	1	0,5%
Single supraventricular extrasystoles.	1	1	0,5%
stomach pain	1	1	0,5%
vomiting	1	1	0,5%
Worsening of existing Dislipidaemia	1	1	0,5%
Worsening of exiting Dislipidaemia	1	1	0,5%
**Total**	**18**	**15**	**8,1%**

Related TEAE	TEAE	Patients (n=186)	% Patient
insomnia	15	13	7,0%
headache	14	13	7,0%
vertigo	6	5	2,7%
anxiety	3	3	1,6%
nervous tension	2	2	1,1%
nervousness	2	2	1,1%
hypotonia	2	1	0,5%
sinus tachycardia	2	1	0,5%
Henoch–Schönlein purpura	1	1	0,5%
impaired arterial hypertension	1	1	0,5%
Irritability	1	1	0,5%
nausea	1	1	0,5%
nerves	1	1	0,5%
Sleep disturbance, nightmares	1	1	0,5%
**Total**	**52**	**36**	**19,4%**

4116 - AE coded as "collapse": It was a collapse due to hypotonia. After revision investigator's assessment is that the event is not related with the study drug. There is no new similar events. The

site decided to code the AE as "hypotonia" rather than collapse. Patient is doing fine (receives 40mg OD in the OL study phase.

4112 (Heinoch Schönlein purpura) - The diagnosis had been established by dermatology and rheumatologist specialist on a outpatients consultation. There is no additional information on the case

The event occurred once and before the site's visit. There were no other symptoms in addition. The patients have not been hospitalized due to AE or due to other reason. On the visit's day the event was in process of recovering, but due to the ongoing hyperpigmentation the outcome was reported as "recovered with sequellae". The investigators' assessment is that it is an AE, it is not an SAE. The patient has not provided medical documentation related with the AE.

3 ECGs

No QTcF > 450 ms was reported

A few patients had a QT variation over 60 ms

Subject Id	Somme de Visit 1-Visit Specific Assessments-QT Interval (msec)	Somme de Visit 2-Visit Specific Assessments-QT Interval (msec)	Somme de Visit 3-Visit Specific Assessments-QT Interval (msec)	Somme de Visit 4-Visit Specific Assessments-QT Interval (msec)	Somme de Visit 5-Visit Specific Assessments-QT Interval (msec)	Somme de Visit 6-Visit Specific Assessments-QT Interval (msec)	Somme de Visit 7-Visit Specific Assessments-QT Interval (msec)
4134	350	399	388	410	395	391	379
4137	354	419	419	366	354	366	364
4153	273	380	392	394	371	399	398

Subject ID	Reported Term for the Adverse Event	Outcome of Adverse Event	Age (years)	Sex	First day of treatment	Start Date/Time of Adverse Event	Causality	Completed visit 7 and 100% SDV
341033, DB-069	sinus tachycardia	Recovered	55	Male	2016-06-29	2016-10-19	Possibly related	No
341033, DB-069	sinus tachycardia	Recovered	55	Male	2016-06-29	2016-10-23	Possibly related	No
353048, DB-095	Single supraventricular extrasystoles.	Recovered	66	Male	2016-07-06	2016-08-24	Unlikely related	Yes

Patient 4133 had all ECG graphs normal from V1 to V7 (Double blind) last ECG graph provided was dated October 3rd 2016. Additional information will be requested.

Patient 5348 reported single supraventricular extra systoles which were considered by the cardiologist as non-clinically significant as outlined in the table below.

Moreover, after 3 months double blind treatment patient ECG was normal.

**P1513 Pt 5348**	
**Visit 1-**	Normal
**Visit 2**<br>**Cardiologist's Comments**	Abnormal<br>single supraventriculare extrasistoles-NCS
**Visit 3-**<br>**Cardiologist's Comments**	Abnormal<br>single supraventriculare extrasistoles-NCS
**Visit 4-**	Normal
**Visit 5-**<br>**Cardiologist's Comments**	Abnormal<br>Single supraventricular extrasystoles. NCS.
**Visit 6-**	Normal
**Visit 7-**	Normal

ECG data March 2nd 2017

Subject Id	Visit 1-Visit Specific Assessments-Date of ECG	Visit 1-Visit Specific Assessments-Time of ECG	Visit 1-Visit Specific Assessments-Heart Rate (beats/min)	Visit 1-Visit Specific Assessments-Sinusal Rhythm	Visit 1-Visit Specific Assessments-PR Interval (msec)	Visit 1-Visit Specific Assessments-QRS Interval (msec)	Visit 1-Visit Specific Assessments-QT Interval (msec)	Visit 1-Visit Specific Assessments-QTcF Interval (msec)	Visit 1-Visit Specific Assessments-Result or Finding in Original Units	Visit 1-Visit Specific Assessments-Cardiologist's Comments
SubjectId	V1.V1_ASS.EGDAT	V1.V1_ASS.EGTIM	V1.V1_ASS.ORRES_HR1	V1.V1_ASS.ORRES_SNRH	V1.V1_ASS.ORRES_PR	V1.V1_ASS.ORRES_QRS	V1.V1_ASS.ORRES_QT	V1.V1_ASS.ORRES_QTCF	V1.V1_ASS.EGORRES	V1.V1_ASS.EGCOVAL
4101	2016-04-07	11:20	52	Yes	137	98	394		Normal	
4102	2016-04-08	09:20	73	Yes	168	89	367	392	Normal	
4103	2016-04-08	11:05	60	Yes	282	115	437		Abnormal	AV block 1 degree, Right branch block
4104	2016-04-11	10:05	64	Yes	153	97	378		Normal	
4105	2016-04-11	09:35	78	Yes	162	90	381		Normal	
4106	2016-04-11	12:00	70	Yes	157	97	396	417	Normal	
4107	2016-04-12	10:15	64	Yes	159	114	354		Normal	
4108	2016-04-12	10:00	57	Yes	237	99	430	423	Normal	
4109	2016-04-12	10:10	75	Yes	169	105	381	410	Normal	
4110	2016-04-13	10:00	72	Yes	163	106	399	424	Normal	
4111	2016-04-13	10:00	101	Yes	134	87	356		Normal	
4112	2016-04-13	10:50	87	No	0	122	363	411	Abnormal	NCS - chronic atrial fibrillation PR interval NA
4113	2016-04-14	10:10	86	Yes	172	113	349	393	Normal	
4114	2016-04-14	10:00	78	Yes	159	98	381		Normal	
4115	2016-04-15	10:50	81	Yes	124	102	382	422	Normal	
4116	2016-04-15	11:50	76	Yes	148	88	369	399	Normal	
4117	2016-04-18	09:40	86	Yes	144	89	355		Normal	
4118	2016-04-18	09:50	67	Yes	143	139	425	441	Normal	
4119	2016-04-18	10:15	74	Yes	147	109	371	398	Normal	
4120	2016-04-18	10:30	70	Yes	168	82	364	383	Normal	
4121	2016-04-19	10:15	74	Yes	170	94	378	405	Normal	
4122	2016-04-19	11:00	89	Yes	180	110	340	388	Normal	
4123	2016-04-22	13:15	91	Yes	135	94	321	369	Normal	
4124	2016-04-25	09:50	82	Yes	143	101	376	417	Normal	
4125	2016-04-25	12:00	71	Yes	126	113	375	397	Normal	
4126	2016-04-27	09:51	53	Yes	159	106	389		Normal	
4127	2016-05-04	10:20	78	Yes	143	107	347		Normal	
4128	2016-05-04	11:20	65	Yes	154	106	389		Normal	
4129	2016-05-10	09:30	86	No	0	106	352		Abnormal	atrial fibrillation
4130	2016-05-10	10:25	61	Yes	160	84	387	389	Normal	
4131	2016-05-11	11:16	60	Yes	181	98	422		Normal	
4132	2016-05-13	10:20	69	Yes	160	98	406	425	Abnormal	NCS - atrial premature
4133	2016-05-16	09:06	67	Yes	185	97	397	412	Normal	
4134	2016-05-16	10:55	87	Yes	182	81	350	396	Normal	
4135	2016-05-17	10:20	51	Yes	205	116	481	456	Abnormal	QTcF-456 msec (>450)
4136	2016-05-17	11:00	65	Yes	174	91	419	430	Normal	
4137	2016-05-18	08:36	75	Yes	181	75	354		Normal	
4138	2016-05-18	10:40	62	Yes	164	93	393	397	Normal	
4139	2016-05-26	10:00	69	Yes	153	104	407	426	Normal	
4140	2016-05-26	10:30	50	Yes	182	96	432	407	Normal	
4141	2016-05-26	12:15	78	Yes	196	94	372	406	Normal	
4142	2016-06-01	10:30	102	Yes	188	84	339	405	Normal	
4143	2016-06-01	12:40	71	Yes	172	105	378	400	Normal	
4144	2016-06-02	11:00	78	Yes	161	86	359	392	Normal	
4145	2016-06-02	11:10	70	Yes	154	112	404	425	Normal	
4146	2016-06-02	11:30	107	Yes	158	86	290	352	Abnormal	NCS sinusal tachycardia
4147	2016-06-03	10:45	91	Yes	203	150	385	442	Abnormal	NCS - left bundle branch block since 2013
4148	2016-06-06	12:10	60	Yes	180	78	382	382	Normal	
4149	2016-06-07	10:10	75	Yes	138	109	368	396	Normal	
4150	2016-06-07	10:30	77	Yes	169	103	362	393	Normal	
4151	2016-06-13	12:20	63	Yes	193	86	416	423	Normal	
4152	2016-06-13	14:10	116	Yes	149	105	311	387	Abnormal	NCS sinus tachycardia
4153	2016-06-14	12:40	89	Yes	157	94	273	311	Normal	
4154	2016-06-20	10:52	76	Yes	155	84	366	396	Normal	
4155	2016-06-20	10:56	96	Yes	133	92	328		Abnormal	supraventricular extrasistoles
4156										
4157	2016-06-22	11:50	81	Yes	156	106	399	441	Normal	
4158	2016-06-30	09:01	91	Yes	157	93	340	391	Normal	
4159	2016-06-30	10:53	89	Yes	139	91	332		Normal	
4160	2016-06-30	10:48	94	Yes	162	94	351		Normal	
4161	2016-07-06	11:30	96	Yes	160	80	322	377	Normal	
4162	2016-07-07	12:50	96	Yes	149	99	360	421	Normal	
4163	2016-07-14	10:50	83	Yes	126	81	360	401	Normal	
4164	2016-09-09	12:30	63	Yes	143	77	381	387	Normal	

4165	2016-09-10	10:00	66	Yes	195	118	378	390	Normal	
4166	2016-09-12	10:40	53	Yes	152	113	438	420	Normal	
4167	2016-09-13	12:25	85	No	0	99	317	356	Abnormal	NCS persistent atrial fibrillation since 2009 with normal heart rate and low risk
4168	2016-09-16	11:36	63	Yes	168	112	403		Normal	
4169	2016-09-19	09:09	65	Yes	181	96	405		Normal	
4170	2016-09-19	10:54	71	Yes	194	90	359	380	Normal	
4171	2016-09-20	09:27	75	Yes	186	101	374		Normal	
4172	2016-09-20	10:10	83	Yes	183	82	353	393	Normal	
4173	2016-09-21	10:50	46	Yes	168	114	432	395	Normal	
4174	2016-09-21	12:25	96	Yes	148	98	345	404	Normal	
4175	2016-09-27	13:15	66	Yes	166	125	387		Normal	
4176	2016-09-28	10:40	80	No	176	81	350	385	Normal	
4177	2016-09-28	12:28	84	Yes	151	90	350		Normal	
4178	2016-09-29	10:30	64	Yes	162	102	442	452	Normal	
4179	2016-09-29	10:35	64	Yes	183	117	378	386	Normal	
4180	2016-09-30	10:40	71	No	192	109	361	382	Normal	
4181	2016-10-04	13:00	74	Yes	162	133	390	418	Normal	
4182	2016-10-04	12:49	74	Yes	150	86	336	360	Normal	
4183	2016-10-05	12:11	75	Yes	194	100	353	380	Normal	
4184	2016-10-06	09:32	96	Yes	115	92	349	408	Normal	
4185	2016-10-06	11:27	61	Yes	159	93	364		Normal	
4187	2016-10-06	12:56	66	Yes	211	74	400		Normal	
4188	2016-10-07	11:05	58	Yes	172	106	434		Normal	
4189	2016-10-07	12:47	93	Yes	200	90	346		Normal	
4190	2016-10-07	12:20	68	Yes	169	114	380	396	Normal	
4201	2016-08-17	10:50	77	Yes	162	100	376	409	Normal	
4202	2016-08-18	09:42	67	Yes	142	110	366	380	Normal	
4203	2016-08-19	09:50	65	Yes	156	108	376	386	Abnormal	Sinus rhythm. Left axis deviation. Non-specific re-polarization changes.
4204	2016-08-21	13:58	76	Yes	156	100	374	405	Normal	
4205	2016-08-22	08:55	72	Yes	186	104	390	414	Normal	
4206	2016-08-23	12:30	80	Yes	176	82	354	390	Normal	
4207	2016-08-24	08:17	86	Yes	160	78	330	372	Normal	
4208	2016-08-24	08:34	63	Yes	154	104	416	423	Normal	
4209	2016-08-26	08:19	61	Yes	150	86	404	406	Normal	
4210	2016-08-26	10:09	62	Yes	140	82	412	417	Normal	
4211	2016-08-28	09:16	72	Yes	170	98	378	402	Normal	
4212	2016-08-29	09:04	71	Yes	134	92	370	391	Normal	
4213	2016-08-31	08:35	76	Yes	166	80	346	374	Normal	
4214	2016-08-31	10:59	68	Yes	170	106	404	421	Normal	
4215	2016-08-31	10:43	63	Yes	156	72	396	402	Normal	
4216	2016-09-07	09:47	63	Yes	156	90	388	394	Normal	
4217	2016-09-09	09:09	76	Yes	144	94	388	420	Normal	
4218	2016-09-11	08:40	83	Yes	198	82	378	421	Normal	
4219	2016-09-27	09:45	78	No	154	74	354	386	Normal	
4220	2016-10-05	09:48	67	Yes	114	84	392	407	Normal	
4221	2016-10-07	09:28	77	Yes	150	90	384	417	Normal	
4222	2016-10-07	08:55	52	Yes	134	94	468	446	Normal	
4223	2016-10-10	08:44	60	Yes	120	100	392	392	Normal	
4224	2016-10-13	19:17	58	Yes	124	86	412	407	Normal	
5101	2016-04-15	08:24	65	Yes	148	90	382	392	Normal	
5102	2016-04-20	08:21	81	Yes	154	94	360	398	Normal	
5103	2016-04-25	08:15	83	No	164	82	380	423	Normal	
5104	2016-05-03	08:20	56	No	186	86	416	407	Normal	
5105	2016-05-12	08:17	56	Yes	136	78	446	436	Normal	
5301	2016-04-11	08:25	77	Yes	200	80	400	435	Normal	
5302	2016-04-11	10:35	71	Yes	180	80	380	402	Normal	
5303	2016-04-12	08:35	89	Yes	200	120	380	433	Abnormal	Left position, left front hemiblock, complete right bundle branch block - NCS
5304	2016-04-12	09:10	84	Yes	180	110	360	403	Abnormal	Left position, incomplete right bundle branch block - NCS
5305	2016-04-13	09:35	62	Yes	200	80	400	404	Normal	
5306	2016-04-13	10:10	79	Yes	200	80	380	416	Normal	
5307	2016-04-14	08:16	70	Yes	200	80	380	400	Normal	
5308	2016-04-14	08:47	66	Yes	200	80	380	392	Normal	
5309	2016-04-15	08:10	77	Yes	180	80	380	413	Normal	
5310	2016-04-15	09:30	94	Yes	180	80	380	441	Normal	
5311	2016-04-18	08:25	104	Yes	180	120	360	432	Abnormal	Sinus tachycardia. Full right bundle branch block. - NCS
5312	2016-04-18	10:00	65	Yes	180	80	380	390	Normal	
5313	2016-04-19	08:40	90	Yes	180	80	360	412	Normal	
5314	2016-04-19	09:01	73	Yes	180	80	380	406	Normal	
5315	2016-04-19	09:06	80	Yes	200	80	400	440	Normal	

5316	2016-04-19	09:20	71	Yes	200	80	380	402	Normal	
5317	2016-04-20	09:00	71	Yes	180	80	400	423	Normal	
5318	2016-04-20	09:15	89	Yes	180	80	380	433	Normal	
5319	2016-04-20	09:45	63	Yes	200	80	380	386	Normal	
5320	2016-04-20	11:10	57	Yes	200	80	400	393	Normal	
5321	2016-04-21	08:25	75	Yes	200	80	380	409	Normal	
5322	2016-04-22	08:50	97	Yes	180	80	380	446	Normal	
5323	2016-04-22	09:20	84	Yes	200	80	380	425	Normal	
5324	2016-04-22	09:46	73	Yes	180	80	400	427	Abnormal	Negative T-wave in II, III, AVF. NCS
5325	2016-04-25	08:41	79	Yes	180	80	380	416	Normal	
5326	2016-04-25	08:46	74	Yes	200	80	380	408	Normal	
5327	2016-04-25	10:31	72	Yes	200	120	400	425	Abnormal	Complete right bundle branch block. - NCS
5328	2016-04-26	08:40	100	Yes	180	80	370	439	Normal	
5329	2016-04-26	09:55	84	Yes	180	80	400	447	Normal	
5330	2016-04-26	10:41	94	Yes	210	120	380	441	Abnormal	Left position, left front hemiblock, complete right bundle branch block. NCS
5331	2016-04-27	08:10	61	Yes	200	80	400	402	Normal	
5332	2016-04-27	09:06	81	Yes	180	90	380	420	Normal	
5333	2016-06-06	09:11	84	Yes	180	80	380	425	Normal	
5334	2016-06-07	10:31	64	Yes	200	120	400	409	Abnormal	Complete right bundle branch block. NSC.
5335	2016-06-07	10:55	59	Yes	200	120	400	398	Abnormal	Complete right bundle branch block. NCS.
5336	2016-06-08	09:10	70	Yes	190	80	390	411	Normal	
5337	2016-06-08	09:38	78	Yes	190	80	400	437	Normal	
5338	2016-06-08	10:00	76	Yes	180	80	400	433	Normal	
5339	2016-06-10	07:36	86	Yes	180	80	380	428	Normal	
5340	2016-06-10	09:00	76	Yes	180	80	380	411	Normal	
5341	2016-06-10	09:16	65	Yes	200	80	400	411	Normal	
5342	2016-06-10	09:30	56	Yes	200	80	400	391	Normal	
5343	2016-06-14	08:29	69	Yes	200	80	390	409	Normal	
5344	2016-06-28	09:15	77	Yes	200	80	380	413	Normal	
5345	2016-06-28	09:35	86	Yes	200	80	380	428	Normal	
5346	2016-06-28	10:16	97	Yes	210	110	380	446	Abnormal	Left position, incomplete right bundle branch block. NCS.
5347	2016-06-29	09:00	67	Yes	200	80	400	415	Normal	
5348	2016-06-29	10:30	77	Yes	200	80	380	413	Normal	
5349	2016-07-01	07:27	63	Yes	200	80	400	407	Normal	
5350	2016-07-05	08:30	79	Yes	190	90	390	427	Normal	
5351	2016-07-05	10:10	80	Yes	200	90	380	418	Normal	
5352	2016-07-11	07:35	65	Yes	180	80	380	390	Normal	
5353	2016-07-11	07:50	76	Yes	200	80	390	422	Normal	
5354	2016-08-01	08:45	84	Yes	180	80	390	436	Normal	
5355	2016-08-01	09:10	66	Yes	200	80	400	413	Normal	
5356	2016-08-02	09:26	64	Yes	190	80	390	398	Normal	
5357	2016-08-03	08:45	70	Yes	200	80	380	400	Normal	
5358	2016-08-03	09:40	76	Yes	190	80	390	422	Normal	
5359	2016-08-04	09:15	76	Yes	200	80	400	433	Normal	
5360	2016-08-04	09:48	100	Yes	200	80	360	427	Normal	
5361	2016-08-05	08:55	85	Yes	200	80	390	438	Normal	
5362	2016-08-05	09:11	62	Yes	200	80	400	404	Normal	
5363	2016-08-05	10:08	75	Yes	200	80	400	431	Normal	
5364	2016-08-09	09:35	77	Yes	200	80	380	413	Normal	
5365	2016-08-09	09:29	87	Yes	190	80	380	430	Normal	
5366	2016-08-15	08:10	86	Yes	190	80	380	428	Normal	
5367	2016-08-15	08:20	73	Yes	200	80	400	427	Normal	
5368	2016-08-17	08:41	68	Yes	200	80	400	417	Normal	
5369	2016-08-23	09:01	64	Yes	200	80	390	398	Normal	
5370	2016-08-25	08:57	77	Yes	200	80	380	413	Normal	
5371	2016-08-25	09:20	61	Yes	200	80	410	412	Normal	
5372	2016-08-25	10:25	72	Yes	200	80	390	414	Normal	
5373	2016-10-13	09:02	82	Yes	190	110	400	444	Abnormal	Incomplete right bundle branch block. NCS.
5374	2016-10-13	08:57	65	Yes	200	80	380	390	Normal	
5375	2016-10-13	08:51	100	Yes	190	80	370	439	Normal	
5376	2016-10-13	10:11	93	Yes	200	80	380	440	Normal	
5377	2016-10-13	10:41	90	Yes	200	80	380	435	Normal	
5378	2016-10-13	10:41	72	Yes	190	80	380	404	Normal	
5601	2016-08-18	08:37	80	No	225	120	356	392	Normal	
5602	2016-09-12	08:44	86	No	151	93	353	398	Normal	
5603	2016-09-13	10:28	85	Yes	164	89	343	385	Normal	
5604	2016-09-27	08:32	66	Yes	148	92	368	380	Normal	
5605	2016-10-13	08:42	50	Yes	142	106	407	383	Normal	

Visit 2-Visit Specific Assessments-Date of ECG	Visit 2-Visit Specific Assessments-Time of ECG	Visit 2-Visit Specific Assessments-Heart Rate (beats/min)	Visit 2-Visit Specific Assessments-Sinusal Rhythm	Visit 2-Visit Specific Assessments-PR Interval (msec)	Visit 2-Visit Specific Assessments-QRS Interval (msec)	Visit 2-Visit Specific Assessments-QT Interval (msec)	Visit 2-Visit Specific Assessments-QTcF Interval (msec)	Visit 2-Visit Specific Assessments-Result or Finding in Original Units	Visit 2-Visit Specific Assessments-Cardiologist's Comments
V2.V2_ASS.EGDAT	V2.V2_ASS.EGTIM	V2.V2_ASS.ORRES_HR1	V2.V2_ASS.ORRES_SNRH	V2.V2_ASS.ORRES_PR	V2.V2_ASS.ORRES_QRS	V2.V2_ASS.ORRES_QT	V2.V2_ASS.ORRES_QTCF	V2.V2_ASS.EGORRES	V2.V2_ASS.EGCOVAL
2016-04-20	08:13	60	Yes	282	115	437		Abnormal	AV block 1st degree
2016-04-21	08:20	73	Yes	168	89	367	392	Normal	
2016-04-21	08:36	58	Yes	145	91	387		Normal	
2016-04-22	08:48	64	Yes	153	97	378		Normal	
2016-04-22	08:23	78	Yes	162	90	381		Normal	
2016-04-25	11:52	68	No	158	98	379	395	Normal	
2016-04-26	10:45	63	Yes	157	114	354		Normal	
2016-04-25	08:15	57	Yes	237	99	430	423	Normal	
2016-04-25	09:15	75	Yes	169	105	381	410	Normal	
2016-04-27	08:20	81	Yes	157	98	383	423	Normal	
2016-04-26	08:15	103	Yes	129	92	355		Normal	
2016-04-26	08:58	82	No	0	190	353	392	Abnormal	NCS - chronic atrial fibrillation
2016-04-26	08:53	81	Yes	184	117	358	396	Normal	
2016-04-27	08:16	80	Yes	147	98	369		Normal	
2016-05-04	07:20	81	Yes	124	102	382	422	Normal	
2016-04-28	06:50	74	Yes	149	85	366	393	Normal	
2016-05-03	08:35	81	Yes	140	96	350		Normal	
2016-05-03	07:00	67	Yes	143	139	425	441	Normal	
2016-05-31	09:10	74	Yes	147	109	371	398	Normal	
2016-05-27	08:53	86	Yes	173	77	333	375	Normal	
2016-05-27	08:53	88	Yes	177	93	363	412	Normal	
2016-05-30	08:05	89	Yes	180	110	340	388	Normal	
2016-05-27	08:20	97	Yes	155	87	315	370	Normal	
2016-05-30	07:45	70	Yes	135	96	389	410	Normal	
2016-06-27	11:46	66	Yes	150	108	374		Normal	
2016-06-22	07:56	77	Yes	119	102	355		Normal	
2016-06-21	07:56	68	Yes	135	120	404		Normal	
2016-06-22	08:48	72	No	0	116	360		Abnormal	atrial fibrillation
2016-06-22	08:55	57	Yes	167	85	403		Normal	
2016-06-27	08:30	88	Yes	106	98	361	410	Normal	
2016-06-29	08:37	64	Yes	200	92	399	408	Normal	
2016-06-28	10:10	68	Yes	195	84	399	416	Normal	
2016-06-21	08:36	64	Yes	163	78	419		Normal	
2016-07-07	07:45	63	Yes	171	98	369	375	Normal	
2016-07-15	06:25	65	Yes	154	106	421	432	Normal	
2016-07-07	09:39	50	Yes	179	98	434	408	Normal	
2016-07-12	07:50	69	Yes	187	87	392	411	Normal	
2016-07-11	12:02	108	Yes	162	104	330	401	Normal	
2016-07-12	11:47	68	Yes	173	106	397	414	Normal	
2016-07-13	09:00	62	Yes	187	92	369	373	Normal	
2016-07-13	10:03	60	Yes	158	113	402	402	Normal	
2016-07-22	08:35	99	Yes	179	93	316	373	Normal	
2016-07-21	09:15	75	No	0	159	434	468	Abnormal	atrial fibrilation - AE, and QTcR> 450 ms (468)
2016-07-13	09:02	58	Yes	178	78	372	368	Normal	
2016-07-20	10:25	80	Yes	124	105	358	394	Normal	
2016-07-12	11:20	72	Yes	147	98	395	420	Normal	
2016-07-25	07:20	60	Yes	179	86	394	394	Normal	
2016-07-22	07:30	100	Yes	158	82	339	402	Normal	
2016-07-25	07:20	74	Yes	167	100	380	408	Normal	
2016-07-15	10:05	80	Yes	153	90	343	378	Normal	
2016-07-15	06:25	84	Yes	167	96	367		Abnormal	ventricular extrasistoles
2016-07-26	07:15	76	Yes	159	110	381	412	Normal	
2016-07-18	10:30	70	Yes	177	85	385	405	Normal	
2016-07-21	08:00	89	Yes	139	91	332		Normal	
2016-07-14	07:52	93	Yes	170	89	336		Normal	

2016-07-26	07:25	72	Yes	142	86	369	392	Normal	
2016-07-29	07:30	84	Yes	142	91	367	411	Normal	
2016-07-28	08:15	93	Yes	125	90	347	402	Normal	
2016-11-03	09:55	75	Yes	136	82	368	396	Normal	
2016-10-26	06:55	68	Yes	154	117	372	388	Normal	
2016-09-26	08:50	63	Yes	140	98	392	398	Normal	
2016-10-26	09:26	93	No	0	86	315	365	Abnormal	NCS - persistent atrium fibrilation without change compare to V1 - NCS
2016-09-21	08:12	63	Yes	154	115	405		Normal	
2016-09-26	08:53	54	Yes	169	125	413		Normal	
2016-10-28	08:14	70	Yes	206	94	360	379	Normal	
2016-10-27	09:26	78	Yes	187	98	328		Normal	
2016-10-27	11:08	104	Yes	172	89	339	407	Normal	
2016-10-31	07:30	49	Yes	159	103	468	437	Normal	
2016-11-01	09:50	98	Yes	168	98	341	402	Normal	
2016-11-18	08:08	66	Yes	147	124	389		Normal	
2016-11-04	08:10	80	No	173	92	355	391	Normal	
2016-10-28	09:59	92	Yes	136	84	345		Normal	
2016-10-31	08:52	79	Yes	165	84	380	416	Normal	
2016-11-07	08:35	89	Yes	160	106	344	392	Normal	
2016-11-03	06:50	76	Yes	154	140	379	410	Normal	
2016-10-26	10:51	80	Yes	160	74	329	362	Normal	
2016-11-03	10:20	76	Yes	192	105	371	401	Normal	
2016-11-10	08:15	94	Yes	121	89	333	387	Normal	
2016-11-01	11:18	77	Yes	166	94	375		Normal	
2016-11-04	08:48	80	Yes	191	80	377		Abnormal	left arterior hemiblock
2016-11-08	07:50	59	Yes	181	106	407		Normal	
2016-11-01	08:11	75	Yes	210	81	333		Normal	
2016-11-07	08:20	67	Yes	156	112	379	393	Normal	
2016-08-25	11:49	94	Yes	148	102	346	402	Normal	
2016-08-31	08:03	60	Yes	160	104	384	384	Normal	
2016-08-30	09:44	58	Yes	166	96	410	405	Normal	
2016-08-28	08:40	78	Yes	158	106	374	408	Normal	
2016-08-29	09:00	76	Yes	190	100	384	415	Normal	
2016-09-02	09:39	86	Yes	168	80	350	395	Normal	
2016-09-07	08:38	74	Yes	168	80	336	360	Normal	
2016-09-07	08:36	67	Yes	158	102	416	432	Normal	
2016-08-29	07:41	67	Yes	132	82	390	405	Normal	
2016-09-09	08:32	60	Yes	132	86	426	426	Normal	
2016-09-02	08:44	69	Yes	170	92	378	396	Normal	
2016-09-08	08:12	90	Yes	152	96	346	396	Normal	
2016-09-09	08:17	74	Yes	164	84	350	375	Normal	
2016-09-10	08:33	66	Yes	170	104	416	429	Normal	
2016-09-10	08:26	67	Yes	152	76	402	417	Normal	
2016-09-12	09:04	65	No	160	92	384	394	Normal	
2016-09-23	09:47	80	Yes	130	100	388	427	Normal	
2016-09-22	08:43	81	Yes	202	80	382	422	Normal	
2016-10-11	13:54	94	Yes	154	74	332	386	Normal	
2016-10-10	14:28	72	Yes	120	94	382	406	Normal	
2016-10-13	09:08	76	No	144	92	386	418	Normal	
2016-10-18	08:37	56	No	138	88	446	436	Normal	
2016-10-17	08:45	63	Yes	124	100	390	396	Normal	
2016-04-27	08:28	86	Yes	108	90	344	388	Normal	
2016-05-04	08:14	84	Yes	170	92	346	387	Normal	
2016-05-10	08:29	73	Yes	154	82	400	427	Normal	
2016-06-27	08:16	61	Yes	174	88	426	428	Normal	
2016-06-29	08:12	61	Yes	142	78	424	426	Normal	
2016-04-23	07:22	77	Yes	220	80	380	413	Normal	
2016-04-26	06:30	75	Yes	180	80	380	409	Normal	
2016-04-26	06:41	87	Yes	180	110	360	407	Abnormal	left position incomplete, right bundle branch block
2016-04-27	06:21	75	Yes	200	80	380	409	Normal	
2016-04-27	06:31	67	Yes	200	80	400	415	Normal	

2016-04-28	07:10	71	Yes	200	80	380	402	Normal	
2016-05-30	07:40	74	Yes	180	80	380	408	Normal	
2016-04-29	07:20	90	Yes	180	80	380	435	Normal	
2016-04-28	07:15	96	Yes	180	120	360	421	Abnormal	Complete right bundle branch block. NCS.
2016-04-29	07:10	86	Yes	200	80	380	428	Normal	
2016-05-30	07:25	88	Yes	200	80	380	432	Normal	
2016-04-28	07:26	71	Yes	200	80	380	402	Normal	
2016-04-29	07:40	70	Yes	200	90	380	400	Normal	
2016-04-28	07:32	73	Yes	180	80	400	427	Normal	
2016-05-30	07:20	66	Yes	180	80	400	413	Normal	
2016-05-31	07:41	77	Yes	180	80	400	435	Normal	
2016-05-30	07:27	87	Yes	200	80	380	430	Normal	
2016-05-31	07:19	60	Yes	200	80	400	400	Normal	
2016-05-31	07:27	74	Yes	200	80	380	408	Normal	
2016-06-01	07:16	93	Yes	180	80	380	440	Normal	
2016-05-31	07:32	85	Yes	200	80	380	427	Normal	
2016-06-22	07:35	81	Yes	190	80	390	431	Normal	
2016-06-04	07:30	64	Yes	200	80	400	409	Normal	
2016-06-04	07:42	73	Yes	180	80	380	406	Normal	
2016-06-22	07:20	84	Yes	210	120	390	436	Abnormal	Complete right bundle branch block. NCS.
2016-06-23	07:23	108	Yes	200	80	360	438	Abnormal	Sinus tachycardia, not significant T waves in V4-V6. NCS.
2016-06-01	07:04	62	Yes	180	80	400	404	Normal	
2016-06-23	07:36	85	Yes	210	120	360	404	Abnormal	Left position, left front hemiblock, complete right bundle branch block. NCS.
2016-06-24	07:28	68	Yes	200	80	400	417	Normal	
2016-06-24	07:22	82	Yes	200	80	390	433	Normal	
2016-07-02	07:18	85	Yes	190	80	370	416	Normal	
2016-07-04	06:56	74	Yes	200	120	400	429	Abnormal	complete right bundle branch block-NCS
2016-07-04	07:10	80	Yes	190	80	380	418	Normal	
2016-07-04	07:15	70	Yes	200	80	400	421	Normal	
2016-07-02	07:34	80	Yes	190	80	390	429	Normal	
2016-07-05	07:15	88	Yes	190	80	380	432	Normal	
2016-07-04	07:25	78	Yes	190	80	380	415	Normal	
2016-07-02	07:25	64	Yes	200	80	400	409	Normal	
2016-07-02	07:32	52	Yes	190	80	400	381	Normal	
2016-07-06	07:14	79	Yes	200	80	380	416	Normal	
2016-07-07	07:05	86	Yes	190	80	380	428	Normal	
2016-07-05	07:06	93	Yes	200	80	370	428	Normal	
2016-07-12	07:01	93	Yes	190	110	380	440	Abnormal	left position,incomplete right bundle branch bloch-NCS
2016-07-07	07:11	69	Yes	200	80	390	409	Normal	
2016-07-06	07:10	75	Yes	180	80	380		Abnormal	single supraventriculare extrasistoles-NCS
2016-07-08	07:00	61	Yes	180	80	400	402	Normal	
2016-07-19	07:00	77	Yes	200	80	390		Normal	
2016-07-19	07:01	87	Yes	200	90	370	419	Normal	
2016-09-02	07:15	66	Yes	180	80	380	392	Normal	
2016-09-02	07:20	63	Yes	190	80	400	407	Normal	
2016-09-04	07:16	93	Yes	180	80	370	428	Normal	
2016-09-04	07:20	75	Yes	200	80	390	420	Normal	
2016-09-08	07:15	71	Yes	210	80	380		Normal	
2016-09-08	07:07	80	Yes	190	80	390		Normal	
2016-09-15	07:17	74	Yes	210	80	380	408	Normal	
2016-09-12	07:16	68	Yes	200	80	400	417	Normal	
2016-09-27	07:25	73	Yes	200	80	390	416	Normal	
2016-09-28	07:12	76	Yes	190	80	380	411	Normal	
2016-09-16	07:15	82	Yes	190	80	390	433	Normal	
2016-09-14	07:12	100	Yes	200	80	370	439	Normal	
2016-09-26	07:28	111	Yes	180	80	360	442	Abnormal	sinus tachycardia -NCS
2016-09-09	07:10	85	Yes	190	80	380	427	Normal	
2016-09-16	07:24	64	Yes	200	80	400	409	Normal	
2016-09-09	07:20	68	Yes	200	80	390		Normal	
2016-09-15	07:15	69	Yes	210	80	380	398	Normal	
2016-09-12	07:12	56	Yes	210	80	420	410	Normal	
2016-09-13	07:20	77	Yes	210	80	400	435	Normal	

2016-10-24	07:21	80	Yes	190	110	400	440	Abnormal	Incomplete right bundle branch block. NCS.
2016-10-24	07:26	68	Yes	200	80	380	396	Normal	
2016-10-25	07:20	70	Yes	200	80	390	411	Normal	
2016-10-25	07:15	65	Yes	200	80	380	390	Normal	
2016-10-25	07:15	81	Yes	200	80	380	420	Normal	
2016-10-24	07:10	63	Yes	190	80	370	376	Normal	
2016-08-31	08:25	85	Yes	235	120	353	396	Normal	
2016-09-26	08:37	86	Yes	146	94	355	400	Normal	
2016-10-27	08:31	59	Yes	144	109	405	403	Normal	

Visit 3-Visit Specific Assessments-Completion Status	Visit 3-Visit Specific Assessments-Date of ECG	Visit 3-Visit Specific Assessments-Time of ECG	Visit 3-Visit Specific Assessments-Reason Not Performed	Visit 3-Visit Specific Assessments-Heart Rate (beats/min)	Visit 3-Visit Specific Assessments-Sinusal Rhythm	Visit 3-Visit Specific Assessments-PR Interval (msec)	Visit 3-Visit Specific Assessments-QRS Interval (msec)	Visit 3-Visit Specific Assessments-QT Interval (msec)	Visit 3-Visit Specific Assessments-QTcF Interval (msec)	Visit 3-Visit Specific Assessments-Result or Finding in Original Units	Visit 3-Visit Specific Assessments-Cardiologist's Comments
V3.V3_ASS.EGPERF	V3.V3_ASS.EGDAT	V3.V3_ASS.EGTIM	V3.V3_ASS.EGREASND	V3.V3_ASS.ORRES_HR1	V3.V3_ASS.ORRES_SNRH	V3.V3_ASS.ORRES_PR	V3.V3_ASS.ORRES_QRS	V3.V3_ASS.ORRES_QT	V3.V3_ASS.ORRES_QTCF	V3.V3_ASS.EGORRES	V3.V3_ASS.EGCOVAL
Yes	2016-05-04	08:14		68	Yes	128	118	411		Abnormal	right branch block
Yes	2016-05-05	08:15		73	Yes	168	89	367	392	Normal	
Yes	2016-05-05	08:10		58	Yes	136	92	370		Normal	
Yes	2016-05-05	08:25		64	Yes	153	97	378		Normal	
Yes	2016-05-05	10:55		78	Yes	162	90	381	416	Normal	
Yes	2016-05-09	09:04		61	Yes	170	91	421	423	Normal	
Yes	2016-05-10	08:26		64	Yes	159	114	354		Normal	
Yes	2016-05-09	07:50		57	Yes	237	99	430	423	Normal	
Yes	2016-05-10	08:20		75	Yes	169	105	381	410	Normal	
Yes	2016-05-10	08:30		72	Yes	163	106	399	424	Normal	
Yes	2016-05-11	08:46		100	Yes	133	87	356		Normal	
No			dropped out								
Yes	2016-05-10	08:04		86	Yes	172	113	349	393	Normal	
Yes	2016-05-11	09:16		94	Yes	145	98	353		Normal	
Yes	2016-05-18	08:05		81	Yes	124	102	382	422	Normal	
Yes	2016-05-11	08:20		74	Yes	149	85	366	393	Normal	
Yes	2016-05-17	08:58		81	Yes	140	96	350		Normal	
Yes	2016-05-17	08:40		67	Yes	141	141	428	444	Normal	
Yes	2016-06-13	09:15		74	Yes	142	97	366	393	Normal	
Yes	2016-06-10	08:14		70	Yes	168	82	364	383	Normal	
Yes	2016-06-10	08:24		75	Yes	156	98	401	432	Normal	
Yes	2016-06-13	10:20		80	Yes	188	115	333	367	Normal	
Yes	2016-06-10	08:40		99	Yes	153	92	313	370	Normal	
Yes	2016-06-13	08:14		82	Yes	143	101	376	417	Normal	
Yes	2016-07-11	11:20		65	Yes	149	106	374		Normal	
Yes	2016-07-06	11:12		78	Yes	143	107	347		Normal	
Yes	2016-07-05	09:44		68	Yes	135	120	404		Normal	
Yes	2016-07-06	10:05		72	No	0	116	360		Abnormal	atrial fibrillation
Yes	2016-07-06	11:38		54	Yes	161	89	417		Normal	
Yes	2016-07-11	08:40		66	Yes	162	98	392	405	Normal	
Yes	2016-07-13	08:07		65	Yes	184	98	415	426	Normal	
Yes	2016-07-12	10:40		74	Yes	182	86	388	416	Normal	
Yes	2016-07-05	10:32		64	Yes	163	78	419		Normal	
Yes	2016-07-21	07:40		62	Yes	164	93	393	397	Normal	
Yes	2016-07-28	08:15		73	Yes	155	103	407	434	Normal	
Yes	2016-07-21	09:58		61	Yes	167	96	421	423	Normal	
Yes	2016-07-26	08:15		74	Yes	189	146	379	406	Normal	
Yes	2016-07-25	09:10		104	Yes	177	106	347	417	Normal	
Yes	2016-07-26	09:48		75	Yes	169	107	379	408	Normal	
Yes	2016-07-28	09:25		66	Yes	170	81	373	385	Normal	
Yes	2016-07-27	09:10		84	Yes	186	111	381	426	Normal	
Yes	2016-08-05	10:00		89	Yes	164	80	309	352	Normal	
Yes	2016-07-28	08:53		58	Yes	177	86	374	370	Normal	
Yes	2016-08-03	08:40		83	Yes	138	105	353	393	Normal	
Yes	2016-07-26	08:07		78	Yes	164	94	380	415	Normal	
Yes	2016-08-08	08:40		63	Yes	193	86	416	423	Normal	
Yes	2016-08-05	09:50		107	Yes	156	80	322	390	Normal	
Yes	2016-08-08	09:30		78	Yes	174	101	392	428	Normal	
Yes	2016-07-29	10:04		80	Yes	153	90	343	378	Normal	
Yes	2016-07-29	08:25		88	Yes	151	92	355		Normal	
Yes	2016-08-09	08:20		82	Yes	162	102	404	448	Normal	
Yes	2016-08-01	07:53		75	Yes	167	86	373	402	Normal	
Yes	2016-08-04	08:50		91	Yes	142	93	344		Normal	
Yes	2016-07-28	09:45		94	Yes	162	94	351		Normal	
Yes	2016-08-10	10:08		74	Yes	138	86	375	402	Normal	
Yes	2016-08-12	09:00		92	Yes	125	96	360	415	Normal	
Yes	2016-08-11	09:10		87	Yes	129	82	361	409	Normal	
Yes	2016-11-17	09:55		76	Yes	134	88	379	410	Normal	
Yes	2016-11-09	09:10		69	Yes	186	120	373	391	Normal	
Yes	2016-10-12	11:40		53	Yes	132	106	428	411	Normal	
Yes	2016-11-09	08:45		71	No	0	88	361	382	Abnormal	longterm cronic atroal fibrillation without change compare to V1 - NCS
Yes	2016-10-07	12:38		64	Yes	164	113	401		Normal	
Yes	2016-10-12	08:56		68	Yes	179	104	416		Normal	
Yes	2016-11-11	11:07		72	Yes	184	78	359	381	Normal	
Yes	2016-11-10	08:38		78	Yes	187	98	328		Normal	

Yes	2016-11-10	08:41		78	Yes	166	86	363	396	Normal	
Yes	2016-11-14	10:40		45	Yes	172	93	427	388	Normal	
Yes	2016-11-15	08:30		97	Yes	135	90	338	397	Normal	
Yes	2016-12-02	09:07		79	Yes	167	110	351		Normal	
Yes	2016-11-18	09:55		81	No	183	85	351	388	Normal	
Yes	2016-11-11	08:19		85	Yes	143	97	359		Normal	
Yes	2016-11-14	09:06		73	Yes	158	93	402	429	Normal	
Yes	2016-11-21	10:15		88	Yes	170	114	348	395	Normal	
Yes	2016-11-17	09:10		78	Yes	148	141	367	401	Normal	
Yes	2016-11-09	09:26		87	Yes	139	83	337	381	Normal	
Yes	2016-11-17	08:14		96	Yes	176	96	343	401	Normal	
Yes	2016-12-01	08:20		93	Yes	132	92	344	398	Normal	
Yes	2016-11-15	08:32		77	Yes	166	94	375		Normal	
Yes	2016-11-18	09:19		67	Yes	193	85	407		Normal	
Yes	2016-11-23	10:20		62	Yes	188	98	429		Normal	
Yes	2016-11-16	08:56		75	Yes	210	81	333		Normal	
Yes	2016-11-22	11:15		69	Yes	173	130	381	399	Normal	
Yes	2016-09-08	09:01		77	Yes	154	102	378	411	Normal	
Yes	2016-09-15	08:25		76	No	162	104	358	387	Normal	
Yes	2016-09-13	09:45		60	Yes	160	98	434	434	Normal	
Yes	2016-09-11	09:22		77	No	156	102	366	398	Normal	
Yes	2016-09-11	09:01		73	No	192	100	386	412	Normal	
Yes	2016-09-16	09:08		82	No	170	82	354	393	Normal	
Yes	2016-09-21	09:01		74	No	170	80	338	362	Normal	
Yes	2016-09-21	08:59		57	Yes	160	104	430	423	Normal	
Yes	2016-09-12	08:54		69	Yes	150	82	396	415	Normal	
Yes	2016-09-23	09:01		59	Yes	130	92	434	432	Normal	
Yes	2016-09-16	08:46		68	No	180	94	382	398	Normal	
Yes	2016-09-22	08:15		89	No	156	96	346	395	Normal	
Yes	2016-09-23	08:36		72	Yes	164	84	352	374	Normal	
Yes	2016-09-24	08:32		67	Yes	170	106	328	340	Normal	
Yes	2016-09-24	09:02		65	Yes	156	74	406	417	Normal	
Yes	2016-09-26	09:19		77	No	172	92	378	411	Normal	
Yes	2016-10-07	09:38		80	Yes	132	106	398	438	Normal	
Yes	2016-10-07	09:16		77	Yes	176	78	386	419	Normal	
Yes	2016-10-26	13:54		74	No	114	88	374	401	Normal	
Yes	2016-10-27	10:15		82	No	164	98	386	428	Normal	
Yes	2016-11-01	09:08		54	No	132	86	450	434	Normal	
Yes	2016-10-31	09:15		65	Yes	126	100	388	398	Normal	
Yes	2016-05-11	08:22		72	Yes	140	90	362	385	Normal	
Yes	2016-05-18	09:12		84	Yes	168	94	374	418	Normal	
Yes	2016-05-26	08:07		81	Yes	148	82	382	422	Normal	
Yes	2016-07-11	09:13		61	Yes	180	84	430	432	Normal	
Yes	2016-07-13	09:19		60	Yes	148	78	414	414	Normal	
Yes	2016-05-05	07:17		84	Yes	210	80	380	425	Normal	
Yes	2016-05-13	07:40		75	Yes	200	80	380	409	Normal	
Yes	2016-05-10	08:23		78	Yes	200	110	400	437	Abnormal	left position incomplete, right bundle branch block
Yes	2016-05-12	08:01		81	Yes	210	100	400	442	Normal	
Yes	2016-05-11	07:30		74	Yes	200	80	380	408	Normal	
Yes	2016-05-12	07:25		74	Yes	200	80	380	408	Normal	
Yes	2016-06-13	07:57		74	Yes	200	80	380	408	Normal	
Yes	2016-05-13	07:57		99	Yes	180	80	360	425	Normal	
Yes	2016-05-12	09:00		97	Yes	200	120	360	423	Abnormal	Complete right bundle branch block. NCS
Yes	2016-05-14	07:40		92	Yes	200	80	380	438	Normal	
Yes	2016-06-13	07:26		88	Yes	200	90	380	432	Normal	
Yes	2016-05-12	07:21		73	Yes	180	80	380	406	Normal	
Yes	2016-05-13	07:37		87	Yes	200	80	380	430	Normal	
Yes	2016-05-12	10:10		70	Yes	200	80	380	400	Normal	
Yes	2016-06-13	08:00		75	Yes	180	80	380	409	Normal	
Yes	2016-06-14	08:16		84	Yes	170	80	390	436	Normal	
Yes	2016-06-14	08:20		68	Yes	190	80	400	417	Normal	
Yes	2016-06-14	08:10		65	Yes	200	80	400	411	Normal	
Yes	2016-06-13	08:40		74	Yes	200	80	390	418	Normal	
Yes	2016-06-15	08:30		98	Yes	180	80	380	448	Normal	
Yes	2016-06-14	09:00		101	Yes	200	80	360	428	Normal	
Yes	2016-07-06	08:00		69	Yes	200	80	400	419	Abnormal	negative T-wave in II,III,aVF-NCS
Yes	2016-06-18	08:02		77	Yes	200	80	390	424	Normal	
Yes	2016-06-18	08:14		78	Yes	180	80	390	426	Normal	
Yes	2016-07-06	07:32		85	Yes	210	120	390	438	Abnormal	complete right bundle branch block-NCS
Yes	2016-07-07	07:44		120	Yes	190	80	350	441	Abnormal	sinus tachicardia-NCS
Yes	2016-06-15	08:18		69	Yes	180	80	400	419	Normal	

Yes	2016-07-07	07:49		85	Yes	200	120	380	427	Abnormal	left position,left front hemiblok,complete right bundle branch block-NCS
Yes	2016-07-08	07:50		63	Yes	200	80	400	407	Normal	
Yes	2016-07-08	08:01		77	Yes	190	80	380	413	Normal	
Yes	2016-07-16	08:02		77	Yes	190	80	390	424	Normal	
Yes	2016-07-18	07:55		65	Yes	200	130	410		Abnormal	Complete right bundle block NCS.
Yes	2016-07-18	08:51		85	Yes	200	80	390	438	Normal	
Yes	2016-07-18	08:41		69	Yes	190	80	390	409	Normal	
Yes	2016-07-16	10:03		71	Yes	190	80	400	423	Normal	
Yes	2016-07-19	08:00		86	Yes	200	80	380		Normal	
Yes	2016-07-18	08:30		89	Yes	200	80	380	433	Normal	
Yes	2016-07-16	08:43		67	Yes	200	80	390	405	Normal	
Yes	2016-07-16	08:30		49	Yes	190	80	420	393	Abnormal	sinus bradykardia-NCS
Yes	2016-07-19	07:11		77	Yes	200	80	380		Normal	
Yes	2016-07-22	08:23		81	Yes	190	80	400		Normal	
Yes	2016-07-19	08:16		91	Yes	200	80	370	425	Normal	
Yes	2016-07-26	07:20		99	Yes	200	110	380	449	Abnormal	left position,incomplete right bundle branch block-NCS
Yes	2016-07-21	07:17		81	Yes	200	80	390		Normal	
Yes	2016-07-20	08:31		68	Yes	200	80	380		Abnormal	single supraventriculare extrasistoles-NCS
Yes	2016-07-22	07:21		69	Yes	180	80	380	398	Normal	
Yes	2016-08-02	07:27		101	Yes	200	80	370		Normal	
Yes	2016-08-04	07:29		83	Yes	200	80	380	423	Normal	
Yes	2016-09-16	08:13		62	Yes	180	80	400	404	Normal	
Yes	2016-09-16	07:40		68	Yes	190	80	380	396	Normal	
Yes	2016-09-19	08:00		87	Yes	190	80	390	441	Normal	
Yes	2016-09-19	07:53		65	Yes	200	80	400	411	Normal	
Yes	2016-09-22	07:30		68	Yes	200	80	380	396	Normal	
Yes	2016-09-22	08:01		84	Yes	180	80	380	425	Normal	
Yes	2016-09-29	07:23		74	Yes	210	80	380	408	Normal	
Yes	2016-09-26	07:35		67	Yes	180	80	400	415	Normal	
Yes	2016-10-09	07:45		72	Yes	190	80	400	425	Normal	
Yes	2016-10-12	07:23		73	Yes	200	80	400	427	Normal	
Yes	2016-10-01	07:30		75	Yes	190	80	390	420	Normal	
Yes	2016-09-28	07:51		91	Yes	190	90	370	425	Normal	
Yes	2016-10-10	07:38		102	Yes	180	80	360	430	Abnormal	Sinus tachycardia. NCS.
Yes	2016-09-23	07:17		79	Yes	190	80	380	416	Normal	
Yes	2016-10-01	07:50		65	Yes	200	80	390	401	Normal	
Yes	2016-09-23	07:41		57	Yes	210	80	410	403	Abnormal	Sinus bradicardya - NCS
Yes	2016-09-29	07:41		70	Yes	210	90	380	400	Normal	
Yes	2016-09-26	08:00		57	Yes	200	80	410	403	Normal	
Yes	2016-09-27	07:30		73	Yes	200	90	390	416	Normal	
Yes	2016-11-07	08:01		73	Yes	180	110	400	427	Abnormal	Incomplete right bundle branch block. NCS.
Yes	2016-11-07	09:31		72	Yes	190	80	380	404	Normal	
Yes	2016-11-08	08:16		72	Yes	200	80	390	414	Normal	
Yes	2016-11-08	07:34		72	Yes	200	80	380	404	Normal	
Yes	2016-11-08	07:15		75	Yes	200	80	390	420	Normal	
Yes	2016-11-07	08:10		75	Yes	190	80	380	409	Normal	
Yes	2016-09-14	07:24		92	Yes	220	115	351	405	Normal	
Yes	2016-10-10	08:51		83	Yes	184	91	359	400	Normal	
Yes	2016-11-10	08:07		52	Yes	137	109	401	382	Normal	

Visit 4-Visit Specific Assessments-Completion Status	Visit 4-Visit Specific Assessments-Date of ECG	Visit 4-Visit Specific Assessments-Time of ECG	Visit 4-Visit Specific Assessments-Heart Rate (beats/min)	Visit 4-Visit Specific Assessments-Sinusal Rhythm	Visit 4-Visit Specific Assessments-PR Interval (msec)	Visit 4-Visit Specific Assessments-QRS Interval (msec)	Visit 4-Visit Specific Assessments-QT Interval (msec)	Visit 4-Visit Specific Assessments-QTcF Interval (msec)	Visit 4-Visit Specific Assessments-Result or Finding in Original Units	Visit 4-Visit Specific Assessments-Cardiologist's Comments
V4.V4_ASS.EGPERF	V4.V4_ASS.EGDAT	V4.V4_ASS.EGTIM	V4.V4_ASS.ORRES_HR1	V4.V4_ASS.ORRES_SNRH	V4.V4_ASS.ORRES_PR	V4.V4_ASS.ORRES_QRS	V4.V4_ASS.ORRES_QT	V4.V4_ASS.ORRES_QTCF	V4.V4_ASS.EGORRES	V4.V4_ASS.EGCOVAL
Yes	2016-05-11	08:35	53	Yes	137	98	394		Normal	
Yes	2016-05-11	08:20	73	Yes	168	89	367	392	Normal	
Yes	2016-05-11	08:50	52	Yes	137	98	394		Normal	
Yes	2016-05-13	08:50	64	Yes	153	97	378		Normal	
Yes	2016-05-13	08:47	78	Yes	162	90	381	416	Normal	
Yes	2016-05-17	11:56	61	Yes	170	91	421	423	Normal	
Yes	2016-05-17	08:40	65	Yes	155	114	354		Normal	
Yes	2016-05-16	08:10	57	Yes	237	99	430	423	Normal	
Yes	2016-05-17	08:45	75	Yes	169	105	381	410	Normal	
Yes	2016-05-17	08:35	72	Yes	163	106	399	424	Normal	
Yes	2016-05-18	08:03	92	Yes	123	102	364		Normal	
Yes	2016-05-17	09:55	84	Yes	184	113	359	402	Normal	
Yes	2016-05-18	08:58	68	Yes	168	90	388		Normal	
Yes	2016-05-25	12:30	81	Yes	124	102	382	422	Normal	
Yes	2016-05-19	08:20	74	Yes	149	85	366	393	Normal	
Yes	2016-05-26	11:30	87	Yes	143	89	356		Normal	
Yes	2016-05-25	08:50	67	Yes	143	139	425	441	Normal	
Yes	2016-06-20	09:13	75	Yes	135	98	348	375	Normal	
Yes	2016-06-17	08:17	81	Yes	165	83	349	386	Normal	
Yes	2016-06-17	08:12	74	Yes	176	99	392	420	Normal	
Yes	2016-06-20	09:42	80	Yes	193	112	347	382	Normal	
Yes	2016-06-17	08:30	91	Yes	143	98	337	387	Normal	
Yes	2016-06-20	09:29	70	Yes	135	96	389	410	Normal	
Yes	2016-07-18	09:12	66	Yes	141	111	382		Normal	
Yes	2016-07-13	11:34	78	Yes	143	107	347		Normal	
Yes	2016-07-13	11:30	68	Yes	135	120	404		Normal	
Yes	2016-07-13	10:28	84	No	0	97	357		Abnormal	atrial fibrillation
Yes	2016-07-13	08:27	70	Yes	172	84	317		Normal	
Yes	2016-07-18	08:40	67	Yes	159	96	386	400	Normal	
Yes	2016-07-20	08:11	67	Yes	185	97	397	412	Normal	
Yes	2016-07-19	08:00	64	Yes	175	84	410	419	Normal	
Yes	2016-07-12	10:47	80	Yes	170	75	366		Normal	
Yes	2016-07-28	08:40	66	Yes	172	85	368	380	Normal	
Yes	2016-08-04	09:00	67	Yes	149	108	412	427	Normal	
Yes	2016-07-28	09:00	50	Yes	182	96	432	407	Normal	
Yes	2016-08-02	09:53	72	Yes	161	85	368	391	Normal	
Yes	2016-08-01	08:15	104	Yes	170	107	349	419	Normal	
Yes	2016-08-02	09:30	75	Yes	165	105	368	396	Normal	
Yes	2016-08-04	09:45	62	Yes	187	92	369	373	Normal	
Yes	2016-08-03	07:54	55	Yes	155	110	411	399	Normal	
Yes	2016-08-12	09:40	83	Yes	194	81	328	365	Normal	
Yes	2016-08-04	10:10	70	Yes	176	81	378	398	Normal	
Yes	2016-08-10	08:35	82	Yes	149	108	354	393	Normal	
Yes	2016-08-02	08:11	73	Yes	160	98	385	411	Normal	
Yes	2016-08-15	09:10	54	Yes	193	94	418	404	Normal	
Yes	2016-08-12	09:50	113	Yes	155	86	316	390	Normal	
Yes	2016-08-15	08:40	79	Yes	163	110	394	432	Normal	
Yes	2016-08-05	10:20	67	Yes	159	90	361	375	Normal	
Yes	2016-08-05	10:34	96	Yes	133	92	328		Normal	
Yes	2016-08-16	09:40	103	Yes	180	108	359	430	Normal	
Yes	2016-08-08	07:54	76	Yes	180	86	367	397	Normal	
Yes	2016-08-12	10:17	93	Yes	139	92	344		Normal	
Yes	2016-08-04	09:19	86	Yes	154	100	357	403	Normal	
Yes	2016-08-16	08:50	75	Yes	167	92	366	394	Normal	
Yes	2016-08-19	09:10	97	Yes	146	100	362	425	Normal	
Yes	2016-08-18	08:00	80	Yes	131	79	370	407	Normal	
Yes	2016-11-24	08:24	74	Yes	134	82	372	399	Normal	

Yes	2016-11-16	09:10	86	Yes	188	112	343	387	Normal	
Yes	2016-10-18	08:50	54	Yes	140	107	435	420	Normal	
Yes	2016-11-16	09:24	83	No	0	86	340	379	Abnormal	NCS longterm chronic atrial fibrillation without change compare to V1
Yes	2016-10-14	08:27	63	Yes	168	112	403		Normal	
Yes	2016-10-19	08:13	54	Yes	169	125	413		Normal	
Yes	2016-11-18	10:55	66	Yes	195	99	386	398	Normal	
Yes	2016-11-17	08:55	74	Yes	160	102	341		Normal	
Yes	2016-11-17	08:57	87	Yes	184	85	329	372	Normal	
Yes	2016-11-21	09:10	49	Yes	160	110	424	396	Normal	
Yes	2016-11-22	08:35	96	Yes	165	92	364	426	Normal	
Yes	2016-12-09	09:57	83	Yes	155	120	356		Normal	
Yes	2016-11-25	08:50	80	No	182	90	357	393	Normal	
Yes	2016-11-18	08:55	87	Yes	143	97	359		Normal	
Yes	2016-11-21	09:28	88	Yes	171	98	369	419	Normal	
Yes	2016-11-28	08:45	69	Yes	181	109	361	378	Normal	
Yes	2016-11-24	08:50	76	Yes	161	138	385	417	Normal	
Yes	2016-11-16	10:06	74	Yes	151	77	344	369	Normal	
Yes	2016-11-24	08:37	95	Yes	183	101	355	414	Normal	
Yes	2016-12-09	08:30	88	Yes	128	93	353	401	Normal	
Yes	2016-11-22	10:35	64	Yes	156	92	381		Normal	
Yes	2016-11-25	09:34	72	Yes	191	91	398		Normal	
Yes	2016-11-29	08:25	58	Yes	173	106	410		Normal	
Yes	2016-11-22	10:49	68	Yes	199	82	369		Normal	
Yes	2016-09-15	08:12	74	No	164	102	374	401	Normal	
Yes	2016-09-21	08:26	73	No	164	110	368	393	Normal	
Yes	2016-09-20	10:09	58	No	162	98	430	425	Normal	
Yes	2016-09-18	08:44	78	No	156	100	370	404	Normal	
Yes	2016-09-19	09:03	74	No	190	98	384	412	Normal	
Yes	2016-09-23	09:43	84	Yes	170	82	352	394	Normal	
Yes	2016-09-28	08:14	76	No	176	80	340	368	Normal	
Yes	2016-09-28	08:22	61	No	156	102	422	424	Normal	
Yes	2016-09-19	08:31	74	No	140	84	384	412	Normal	
Yes	2016-09-30	08:56	59	No	134	86	428	426	Normal	
Yes	2016-09-23	09:02	68	No	178	92	376	392	Normal	
Yes	2016-09-29	08:17	88	No	160	96	346	393	Normal	
Yes	2016-09-30	09:01	70	No	162	86	356	375	Normal	
Yes	2016-10-01	09:00	65	No	166	106	390	401	Normal	
Yes	2016-10-01	09:22	69	No	152	72	400	419	Normal	
Yes	2016-10-04	20:31	60	Yes	160	92	392	392	Normal	
Yes	2016-10-14	09:21	79	Yes	136	98	392	430	Normal	
Yes	2016-10-13	09:12	96	Yes	170	76	358	419	Normal	
Yes	2016-10-31	14:41	72	Yes	118	86	386	410	Normal	
Yes	2016-11-03	08:37	82	Yes	152	90	386	428	Normal	
Yes	2016-11-08	08:56	54	Yes	138	86	456	440	Normal	
Yes	2016-11-07	08:30	66	Yes	130	98	388	401	Normal	
Yes	2016-05-18	08:29	75	Yes	128	90	348	375	Normal	
Yes	2016-05-25	08:31	85	Yes	170	94	370	416	Normal	
Yes	2016-06-03	08:14	77	Yes	142	84	394	428	Normal	
Yes	2016-07-18	09:09	59	Yes	186	86	428	426	Normal	
Yes	2016-07-20	09:39	58	Yes	144	78	426	421	Normal	
Yes	2016-05-15	07:36	82	Yes	200	80	380	422	Normal	
Yes	2016-05-19	08:00	66	Yes	200	80	400	413	Normal	
Yes	2016-05-17	08:18	76	Yes	200	110	400	433	Abnormal	left position incomplete, right bundle branch block. NCS.
Yes	2016-05-19	08:30	73	Yes	200	80	380	406	Normal	
Yes	2016-05-18	08:00	70	Yes	200	80	400	421	Normal	
Yes	2016-05-19	08:11	68	Yes	200	80	400	417	Normal	
Yes	2016-06-20	07:29	72	Yes	200	80	380	404	Normal	
Yes	2016-05-19	08:41	90	Yes	180	80	380	435	Normal	
Yes	2016-05-19	08:44	93	Yes	200	120	380		Abnormal	Complete right bundle branch block. NCS.
Yes	2016-05-20	08:10	95	Yes	200	80	380	443	Normal	
Yes	2016-06-20	07:25	93	Yes	200	80	360	417	Normal	
Yes	2016-05-19	08:15	67	Yes	200	80	400	415	Normal	
Yes	2016-05-21	07:55	82	Yes	200	80	380	422	Normal	

Yes	2016-05-19	08:20	67	Yes	200	80	400	415	Normal	
Yes	2016-06-19	08:06	71	Yes	200	80	390	413	Normal	
Yes	2016-06-21	08:28	77	Yes	180	80	400	435	Normal	
Yes	2016-06-20	08:26	63	Yes	200	80	400	407	Normal	
Yes	2016-06-21	07:19	67	Yes	190	80	400	415	Normal	
Yes	2016-06-19	08:10	71	Yes	200	80	400	423	Normal	
Yes	2016-06-22	07:47	75	Yes	180	80	400	431	Normal	
Yes	2016-06-21	07:05	85	Yes	200	80	380	427	Normal	
Yes	2016-07-13	08:09	67	Yes	190	80	390	405	Normal	
Yes	2016-06-25	08:38	76	Yes	200	80	380	411	Normal	
Yes	2016-06-25	08:47	68	Yes	190	80	380	396	Normal	
Yes	2016-07-13	07:55	74	Yes	200	130	400	429	Abnormal	complete right bundle branch block-NCS
Yes	2016-07-14	07:17	117	Yes	180	80	360	450	Abnormal	sinus tachicardia-NCS
Yes	2016-06-22	08:36	63	Yes	180	80	400	407	Normal	
Yes	2016-07-14	07:59	85	Yes	200	120	370	416	Abnormal	left position,left front hemiblok,complete right bundle branch block-NCS
Yes	2016-07-15	08:34	64	Yes	200	80	400	409	Normal	
Yes	2016-07-15	08:50	85	Yes	190	80	380	427	Normal	
Yes	2016-07-23	08:01	77	Yes	200	80	390	424	Normal	
Yes	2016-07-25	07:53	72	Yes	200	140	400	425	Abnormal	complete right bundle branch block-NCS
Yes	2016-07-25	07:40	78	Yes	200	80	380		Normal	
Yes	2016-07-25	07:16	73	Yes	200	80	390		Normal	
Yes	2016-07-23	09:18	70	Yes	190	80	390	411	Normal	
Yes	2016-07-26	09:06	89	Yes	200	80	380	433	Normal	
Yes	2016-07-26	07:44	93	Yes	180	80	380	440	Normal	
Yes	2016-07-23	08:24	66	Yes	200	80	400	413	Normal	
Yes	2016-07-23	08:19	49	Yes	200	80	400	374	Abnormal	sinus bradikardia-NCS
Yes	2016-07-27	07:59	83	Yes	190	80	380	423	Normal	
Yes	2016-07-28	07:15	87	Yes	200	80	390	441	Normal	
Yes	2016-07-26	07:29	96	Yes	200	80	370	433	Normal	
Yes	2016-08-02	08:15	92	Yes	200	110	380	438	Abnormal	left position,incomplete right bundle branch bloch-NCS
Yes	2016-07-28	07:24	76	Yes	200	80	400	433	Normal	
Yes	2016-07-27	10:31	80	Yes	200	80	380	418	Normal	
Yes	2016-08-01	08:56	63	Yes	200	80	400	407	Normal	
Yes	2016-08-09	07:40	75	Yes	200	80	400	431	Normal	
Yes	2016-08-09	08:55	82	Yes	190	80	380	422	Normal	
Yes	2016-09-23	08:00	67	Yes	180	80	400	415	Normal	
Yes	2016-09-23	07:56	67	Yes	190	80	390	405	Normal	
Yes	2016-09-27	08:11	87	Yes	180	80	380	430	Normal	
Yes	2016-09-27	08:14	64	Yes	180	80	400	409	Normal	
Yes	2016-09-29	07:30	75	Yes	200	80	380	409	Normal	
Yes	2016-09-29	07:57	74	Yes	200	80	380	408	Normal	
Yes	2016-10-06	07:29	75	Yes	210	80	400	431	Normal	
Yes	2016-10-03	07:34	72	Yes	200	80	380	404	Normal	
Yes	2016-10-18	07:27	82	Yes	190	80	400	444	Normal	
Yes	2016-10-21	07:16	75	Yes	200	80	400	431	Normal	
Yes	2016-10-07	07:41	79	Yes	190	80	400	438	Normal	
Yes	2016-10-04	07:33	86	Yes	190	80	380	428	Normal	
Yes	2016-10-18	07:48	116	Yes	180	80	360	448	Abnormal	Sinus tachycardia. NCS.
Yes	2016-09-30	07:40	83	Yes	180	80	390	435	Normal	
Yes	2016-10-07	07:24	66	Yes	200	80	390	403	Normal	
Yes	2016-09-30	08:11	65	Yes	200	80	400	411	Normal	
Yes	2016-10-05	07:30	83	Yes	200	80	380	423	Normal	
Yes	2016-10-03	08:40	51	Yes	210	80	420	398	Abnormal	Sinus bradycardia. NCS.
Yes	2016-10-04	07:11	80	Yes	200	80	380	418	Normal	
Yes	2016-11-14	07:31	68	Yes	190	110	400	417	Abnormal	Incomplete right bundle branch block. NCS.
Yes	2016-11-14	08:18	80	Yes	200	80	390	429	Normal	
Yes	2016-11-15	07:46	85	Yes	190	80	390	438	Normal	
Yes	2016-11-16	07:16	79	Yes	200	80	380	416	Normal	
Yes	2016-11-15	07:18	75	Yes	200	80	390	420	Normal	
Yes	2016-11-14	08:11	66	Yes	190	80	370	382	Normal	
Yes	2016-09-21	11:50	67	No	253	126	386	400	Normal	
Yes	2016-10-17	07:58	85	Yes	147	90	345	387	Normal	
Yes	2016-11-17	07:53	60	Yes	133	110	389	389	Normal	

Visit 5-Visit Specific Assessments-Completion Status	Visit 5-Visit Specific Assessments-Date of ECG	Visit 5-Visit Specific Assessments-Time of ECG	Visit 5-Visit Specific Assessments-Heart Rate (beats/min)	Visit 5-Visit Specific Assessments-Sinusal Rhythm	Visit 5-Visit Specific Assessments-PR Interval (msec)	Visit 5-Visit Specific Assessments-QRS Interval (msec)	Visit 5-Visit Specific Assessments-QT Interval (msec)	Visit 5-Visit Specific Assessments-QTcF Interval (msec)	Visit 5-Visit Specific Assessments-Result or Finding in Original Units	Visit 5-Visit Specific Assessments-Cardiologist's Comments
V5.V5_ASS.EGPERF	V5.V5_ASS.EGDAT	V5.V5_ASS.EGTIM	V5.V5_ASS.ORRES_HR1	V5.V5_ASS.ORRES_SNRH	V5.V5_ASS.ORRES_PR	V5.V5_ASS.ORRES_QRS	V5.V5_ASS.ORRES_QT	V5.V5_ASS.ORRES_QTCF	V5.V5_ASS.EGORRES	V5.V5_ASS.EGCOVAL
Yes	2016-06-08	08:10	67	Yes	235	106	392		Abnormal	right branch block
Yes	2016-06-08	08:10	64	Yes	157	85	397	406	Normal	
Yes	2016-06-08	09:40	59	Yes	148	89	379		Normal	
Yes	2016-06-08	09:10	64	Yes	153	97	378		Normal	
Yes	2016-06-08	08:30	78	Yes	162	90	381	416	Normal	
Yes	2016-06-14	09:45	65	Yes	171	94	398	409	Normal	
Yes	2016-06-14	08:22	64	Yes	159	114	354		Abnormal	right branch block
Yes	2016-06-14	11:40	57	Yes	237	99	430	423	Normal	
Yes	2016-06-14	08:40	76	Yes	176	100	374	405	Normal	
Yes	2016-06-15	09:04	77	Yes	133	105	375	408	Normal	
Yes	2016-06-14	08:13	104	Yes	127	93	349		Abnormal	sinus tachycardia
Yes	2016-06-14	09:23	88	Yes	174	112	360	409	Normal	
Yes	2016-06-15	10:45	80	Yes	147	98	369		Normal	
Yes	2016-06-17	10:30	72	Yes	157	108	384	408	Normal	
Yes	2016-06-15	08:40	74	Yes	149	85	366	393	Normal	
Yes	2016-06-21	11:46	86	Yes	144	89	355		Normal	
Yes	2016-06-22	07:40	59	Yes	140	141	451	448	Normal	
Yes	2016-07-18	08:40	81	Yes	136	97	338	374	Normal	
Yes	2016-07-15	10:22	70	Yes	168	82	364	383	Normal	
Yes	2016-07-15	11:25	78	Yes	164	96	391	427	Normal	
Yes	2016-07-18	09:20	66	Yes	202	110	356	367	Normal	
Yes	2016-07-14	08:25	105	Yes	154	94	316	381	Normal	
Yes	2016-07-18	09:28	69	Yes	144	106	372	390	Normal	
Yes	2016-08-16	08:11	70	Yes	148	102	346		Normal	
Yes	2016-08-10	09:05	77	Yes	119	102	355		Normal	
Yes	2016-08-09	10:26	72	Yes	148	110	403		Abnormal	ventricular exstrasistoles, NCS
Yes	2016-08-10	09:41	84	No	0	101	345		Abnormal	not clinically significant
Yes	2016-08-10	08:12	76	Yes	172	87	365	395	Normal	
Yes	2016-08-15	09:10	71	Yes	158	102	373	395	Normal	
Yes	2016-08-22	09:16	69	Yes	184	92	395	414	Normal	
Yes	2016-08-17	09:50	71	Yes	179	82	395	418	Normal	
Yes	2016-08-09	10:20	75	Yes	181	75	354		Normal	
Yes	2016-08-25	11:00	68	Yes	171	105	374	390	Normal	
Yes	2016-09-01	08:30	67	Yes	140	106	395	410	Normal	
Yes	2016-08-25	08:50	57	Yes	195	87	421	414	Normal	
Yes	2016-08-30	09:52	79	Yes	204	86	352	386	Normal	
Yes	2016-08-29	09:59	104	Yes	199	82	302	363	Normal	
Yes	2016-08-30	08:23	77	Yes	165	107	371	403	Normal	
Yes	2016-09-01	09:33	57	Yes	160	88	375	369	Normal	
Yes	2016-08-31	08:32	65	Yes	168	110	403	414	Normal	
Yes	2016-09-09	09:10	93	Yes	165	87	320	370	Normal	
Yes	2016-09-01	09:35	64	Yes	182	78	366	374	Normal	
Yes	2016-09-07	09:45	85	Yes	140	106	342	384	Normal	
Yes	2016-08-31	10:51	80	Yes	149	93	382	420	Normal	
Yes	2016-09-12	09:10	56	Yes	203	91	421	411	Normal	
Yes	2016-09-09	09:20	110	Yes	124	82	316	387	Normal	
Yes	2016-09-12	09:20	88	Yes	148	102	371	422	Normal	
Yes	2016-09-02	09:28	66	Yes	154	94	365	377	Normal	
Yes	2016-09-02	09:18	88	Yes	151	92	355		Normal	
Yes	2016-09-13	08:10	76	Yes	172	108	404	437	Normal	
Yes	2016-09-09	10:07	80	Yes	179	89	357	393	Normal	
Yes	2016-09-08	11:38	91	Yes	142	93	344		Normal	
Yes	2016-09-01	09:51	76	Yes	152	94	394		Normal	
Yes	2016-09-14	11:00	78	Yes	154	92	359	392	Normal	
Yes	2016-09-16	09:10	91	Yes	140	94	368	423	Normal	
Yes	2016-09-15	08:50	75	Yes	132	77	364	392	Normal	
Yes	2016-12-23	08:58	77	Yes	130	79	353	384	Normal	

Yes	2016-11-15	12:33	55	Yes	142	101	429	417	Normal	
Yes	2016-12-14	09:30	103	No	0	88	321	384	Abnormal	NCS longterm chronic atrial fibrillation without change compare to V1
Yes	2016-11-17	10:51	63	Yes	154	115	405		Normal	
Yes	2016-11-15	07:51	60	Yes	171	110	405		Normal	
Yes	2016-12-15	09:07	70	Yes	180	98	335		Normal	
Yes	2016-12-15	09:24	100	Yes	181	83	340		Normal	
Yes	2016-12-19	09:30	54	Yes	169	98	410	396	Normal	
Yes	2016-12-20	10:45	96	Yes	148	98	345	404	Normal	
Yes	2017-01-06	08:04	60	Yes	155	109	375		Normal	
Yes	2016-12-23	08:35	80	No	176	81	350	385	Normal	
Yes	2016-12-16	10:38	92	Yes	136	84	345		Normal	
Yes	2016-12-19	09:33	77	Yes	162	91	391	425	Normal	
Yes	2016-12-27	10:10	87	Yes	171	114	343	388	Normal	
Yes	2016-12-22	09:00	71	Yes	158	142	376	398	Normal	
Yes	2016-12-14	09:20	77	Yes	148	71	339	368	Normal	
Yes	2016-12-22	08:31	98	Yes	150	111	335	395	Normal	
Yes	2017-01-05	08:15	104	Yes	124	88	339	407	Normal	
Yes	2016-12-19	11:32	64	Yes	156	92	381		Normal	
Yes	2016-12-21	08:50	62	Yes	182	96	438		Normal	
Yes	2016-12-28	08:30	78	Yes	168	105	367		Normal	
Yes	2016-12-19	11:34	68	Yes	199	82	369		Normal	
Yes	2016-10-14	09:41	93	Yes	152	104	356	412	Normal	
Yes	2016-10-19	08:41	58	No	160	104	386	382	Normal	
Yes	2016-10-18	10:02	66	Yes	162	98	396	409	Normal	
Yes	2016-10-16	08:46	74	Yes	156	102	374	401	Normal	
Yes	2016-10-18	08:50	81	Yes	184	108	390	431	Normal	
Yes	2016-10-21	09:44	80	No	168	84	356	392	Normal	
Yes	2016-10-26	08:34	77	No	184	80	340	369	Normal	
Yes	2016-10-26	09:16	63	Yes	156	106	426	433	Normal	
Yes	2016-10-17	09:05	79	No	144	82	350	384	Normal	
Yes	2016-10-28	08:34	56	Yes	134	86	434	424	Normal	
Yes	2016-10-21	08:44	68	No	174	96	384	400	Normal	
Yes	2016-10-27	08:19	86	No	168	96	346	390	Normal	
Yes	2016-10-28	08:48	72	No	168	84	350	372	Normal	
Yes	2016-10-29	08:44	67	No	166	106	404	419	Normal	
Yes	2016-10-29	08:33	67	No	158	74	406	421	Normal	
Yes	2016-10-31	11:10	63	Yes	158	104	380	386	Normal	
Yes	2016-11-11	10:05	79	Yes	134	100	392	430	Normal	
Yes	2016-11-10	08:27	68	Yes	180	80	388	405	Normal	
Yes	2016-11-28	13:37	71	Yes	118	86	390	413	Normal	
Yes	2016-12-01	09:03	74	Yes	146	90	400	429	Normal	
Yes	2016-12-06	08:34	52	Yes	132	92	462	440	Normal	
Yes	2016-12-05	09:15	62	Yes	128	98	388	392	Normal	
Yes	2016-06-15	08:22	78	Yes	146	90	370	404	Normal	
Yes	2016-06-22	08:14	82	Yes	176	92	362	402	Normal	
Yes	2016-06-28	08:20	72	Yes	132	84	402	427	Normal	
Yes	2016-08-15	10:06	68	Yes	178	86	420	438	Normal	
Yes	2016-08-17	09:11	61	Yes	140	76	422	424	Normal	
Yes	2016-06-12	07:51	74	Yes	180	80	380	408	Normal	
Yes	2016-06-14	08:01	76	Yes	200	80	390	422	Normal	
Yes	2016-06-14	08:01	76	Yes	200	90	400	433	Normal	
Yes	2016-06-15	07:15	84	Yes	200	80	380	425	Normal	
Yes	2016-06-15	07:00	70	Yes	200	80	400	421	Normal	
Yes	2016-06-16	07:11	78	Yes	190	80	380	415	Normal	
Yes	2016-07-18	08:01	64	Yes	180	80	390	398	Normal	
Yes	2016-06-16	08:45	95	Yes	180	80	380	443	Normal	
Yes	2016-06-16	08:35	92	Yes	190	120	360	415	Abnormal	Complete right bundle branch block NCS.
Yes	2016-06-17	07:41	83	Yes	200	80	380	423	Normal	
Yes	2016-07-18	08:16	85	Yes	200	90	380	427	Normal	
Yes	2016-06-16	07:10	75	Yes	200	80	400	431	Normal	
Yes	2016-06-17	09:33	88	Yes	200	80	380	432	Normal	

Yes	2016-06-16	09:21	67	Yes	200	80	380	394	Normal	
Yes	2016-07-18	07:59	62	Yes	200	80	400		Normal	
Yes	2016-07-19	07:14	78	Yes	190	80	390		Normal	
Yes	2016-07-19	07:20	63	Yes	190	80	400		Normal	
Yes	2016-07-19	07:14	61	Yes	180	80	380		Normal	
Yes	2016-07-19	07:11	77	Yes	190	80	390		Normal	
Yes	2016-07-20	09:00	73	Yes	200	80	400	427	Normal	
Yes	2016-07-19	07:32	79	Yes	200	80	380		Normal	
Yes	2016-08-10	07:30	76	Yes	200	80	400	433	Abnormal	Non significant negative T-wave in II,III,aVF-NCS
Yes	2016-07-23	09:00	74	Yes	200	80	380	408	Normal	
Yes	2016-07-23	09:04	80	Yes	200	80	380	418	Normal	
Yes	2016-08-10	07:51	81	Yes	200	140	400	442	Abnormal	complete right bundle branch block-NCS
Yes	2016-08-11	07:10	117	Yes	190	80	360	450	Abnormal	sinus tachicardia-NCS
Yes	2016-07-20	08:01	75	Yes	180	80	390	420	Normal	
Yes	2016-08-11	07:44	82	Yes	200	130	380	422	Abnormal	left position,left front hemiblock,complete right bundle branch block-NCS
Yes	2016-08-12	07:27	73	Yes	190	80	400	427	Normal	
Yes	2016-08-12	07:49	79	Yes	200	80	380	416	Normal	
Yes	2016-08-19	07:50	85	Yes	200	80	380	427	Normal	
Yes	2016-08-22	09:10	64	Yes	190	140	400	409	Abnormal	complete right bundle branch block-NCS
Yes	2016-08-22	07:41	77	Yes	200	80	390	424	Normal	
Yes	2016-08-22	07:17	73	Yes	200	80	390	416	Normal	
Yes	2016-08-19	07:51	71	Yes	200	80	390	413	Normal	
Yes	2016-08-23	07:32	78	Yes	180	80	380	415	Normal	
Yes	2016-08-23	07:51	86	Yes	190	80	390	440	Normal	
Yes	2016-08-19	07:41	79	Yes	200	80	390	427	Normal	
Yes	2016-08-19	07:13	55	Yes	200	80	410	398	Normal	
Yes	2016-08-24	08:01	68	Yes	200	80	390	407	Normal	
Yes	2016-08-26	08:01	82	Yes	200	80	380	422	Normal	
Yes	2016-08-23	08:05	99	Yes	190	80	380	449	Normal	
Yes	2016-08-31	07:45	89	Yes	200	90	390	445	Normal	
Yes	2016-08-25	07:37	89	Yes	200	80	390	445	Normal	
Yes	2016-08-24	09:31	80	Yes	200	80	390	429	Abnormal	Single supraventricular extrasystoles. NCS.
Yes	2016-08-26	07:27	73	Yes	190	80	400		Normal	
Yes	2016-09-06	08:00	59	Yes	200	80	400	398	Normal	
Yes	2016-09-06	08:05	79	Yes	200	80	390	427	Normal	
Yes	2016-10-21	09:02	78	Yes	180	80	400	437	Normal	
Yes	2016-10-21	09:09	63	Yes	190	80	400	407	Normal	
Yes	2016-10-23	08:01	95	Yes	180	80	370	431	Normal	
Yes	2016-10-23	08:14	67	Yes	200	80	400	415	Normal	
Yes	2016-10-27	08:01	73	Yes	200	80	380	406	Normal	
Yes	2016-10-28	07:30	79	Yes	190	80	390	427	Normal	
Yes	2016-11-03	08:00	73	Yes	210	80	380	406	Normal	
Yes	2016-10-31	07:01	83	Yes	190	80	380	423	Normal	
Yes	2016-11-15	08:01	66	Yes	190	80	400	413	Normal	
Yes	2016-11-16	07:20	76	Yes	200	80	400	433	Normal	
Yes	2016-11-04	07:37	73	Yes	200	80	400	427	Normal	
Yes	2016-11-02	07:57	85	Yes	190	80	380	427	Normal	
Yes	2016-11-14	07:11	105	Yes	180	80	360	434	Abnormal	Sinus tachycardia. NCS.
Yes	2016-10-28	07:12	80	Yes	190	80	380	418	Normal	
Yes	2016-11-04	08:30	71	Yes	200	80	390	413	Normal	
Yes	2016-10-28	07:22	64	Yes	200	80	400	409	Normal	
Yes	2016-11-03	08:30	80	Yes	200	80	380	418	Normal	
Yes	2016-10-31	09:48	52	Yes	210	80	420	400	Abnormal	Sinus Bradycardia NCS.
Yes	2016-11-01	07:33	84	Yes	200	80	380	425	Normal	
Yes	2016-12-12	08:22	67	Yes	190	110	400	415	Abnormal	Incomplete right bundle branch block. NCS.
Yes	2016-12-12	07:37	72	Yes	190	80	380	404	Normal	
Yes	2016-12-13	08:10	72	Yes	200	80	400	425	Normal	
Yes	2016-12-13	09:30	91	Yes	190	80	370	425	Normal	
Yes	2016-12-13	08:06	77	Yes	200	80	380	413	Normal	
Yes	2016-12-12	08:37	71	Yes	200	80	380	402	Normal	
Yes	2016-10-19	13:43	68	Yes	250	119	368	384	Normal	
Yes	2016-11-14	08:07	84	Yes	155	88	337	377	Normal	
Yes	2016-12-15	08:25	54	Yes	153	113	409	395	Normal	

Visit 6-Visit Specific Assessments-Completion Status	Visit 6-Visit Specific Assessments-Date of ECG	Visit 6-Visit Specific Assessments-Time of ECG	Visit 6-Visit Specific Assessments-Reason Not Performed	Visit 6-Visit Specific Assessments-Heart Rate (beats/min)	Visit 6-Visit Specific Assessments-Sinusal Rhythm	Visit 6-Visit Specific Assessments-PR Interval (msec)	Visit 6-Visit Specific Assessments-QRS Interval (msec)	Visit 6-Visit Specific Assessments-QT Interval (msec)	Visit 6-Visit Specific Assessments-QTcF Interval (msec)	Visit 6-Visit Specific Assessments-Result or Finding in Original Units	Visit 6-Visit Specific Assessments-Cardiologist's Comments
V6.V6_ASS.EGPERF	V6.V6_ASS.EGDAT	V6.V6_ASS.EGTIM	V6.V6_ASS.EGREASND	V6.V6_ASS.ORRES_HR1	V6.V6_ASS.ORRES_SNRH	V6.V6_ASS.ORRES_PR	V6.V6_ASS.ORRES_QRS	V6.V6_ASS.ORRES_QT	V6.V6_ASS.ORRES_QTCF	V6.V6_ASS.EGORRES	V6.V6_ASS.EGCOVAL
Yes	2016-07-13	07:25		74	Yes	201	109	396		Normal	
Yes	2016-07-13	07:00		65	Yes	171	85	399	410	Normal	
Yes	2016-07-14	10:25		80	Yes	140	87	349		Normal	
Yes	2016-07-15	07:38		78	Yes	173	89	364		Normal	
Yes	2016-07-15	07:55		69	Yes	161	91	368		Normal	
Yes	2016-07-19	12:05		77	Yes	168	97	379	412	Normal	
Yes	2016-07-20	11:05		88	Yes	164	113	338		Normal	
Yes	2016-07-19	06:30		60	Yes	244	94	430	430	Normal	
Yes	2016-07-19	08:36		74	Yes	170	104	381	409	Normal	
Yes	2016-07-20	09:20		76	Yes	158	98	362	392	Normal	
Yes	2016-07-20	10:26		89	Yes	132	89	372		Normal	
Yes	2016-07-18	11:36		84	Yes	186	111	381	426	Normal	
Yes	2016-07-20	08:05		69	Yes	85	95	399		Normal	
Yes	2016-07-27	06:55		74	Yes	158	106	380	408	Normal	
Yes	2016-07-20	06:50		74	Yes	151	90	389	417	Normal	
Yes	2016-07-22	07:35		67	Yes	157	93	396		Normal	
Yes	2016-07-22	06:30		59	Yes	140	141	451	448	Normal	
Yes	2016-08-18	09:20		78	Yes	126	91	334	365	Normal	
Yes	2016-08-19	08:54		74	Yes	156	91	369	396	Normal	
Yes	2016-08-22	12:02		74	Yes	175	105	391	419	Normal	
Yes	2016-08-22	09:00		82	Yes	196	126	348	386	Normal	
Yes	2016-08-18	09:25		76	Yes	157	97	362	392	Normal	
Yes	2016-08-22	11:06		98	Yes	180	105	381	449	Normal	
Yes	2016-09-20	08:22		64	Yes	152	102	363		Normal	
Yes	2016-09-14	07:32		90	Yes	150	110	362		Normal	
Yes	2016-09-14	09:14		72	Yes	150	99	377		Abnormal	ventricular extrasistoles
Yes	2016-09-14	08:29		82	No	0	98	339		Abnormal	atrial fibrillation
Yes	2016-09-16	08:15		76	Yes	172	84	365		Normal	
Yes	2016-09-19	09:10		84	Yes	135	98	348	389	Normal	
Yes	2016-09-26	11:57		56	Yes	314	104	422	412	Normal	
Yes	2016-09-21	07:50		72	Yes	188	84	391	415	Normal	
Yes	2016-09-13	07:52		80	Yes	170	75	366		Normal	
Yes	2016-09-29	06:30		69	Yes	131	95	368	386	Normal	
Yes	2016-10-07	08:25		56	Yes	144	112	429	419	Normal	
Yes	2016-09-29	09:09		58	Yes	175	122	427	422	Normal	
Yes	2016-10-04	09:04		74	Yes	198	89	364	390	Normal	
Yes	2016-10-03	07:07		94	Yes	205	80	316	367	Normal	
Yes	2016-10-04	11:50		64	Yes	168	114	373	381	Normal	
Yes	2016-10-06	09:01		66	Yes	164	79	366	378	Normal	
Yes	2016-10-05	09:06		56	Yes	149	124	407	398	Normal	
Yes	2016-10-14	07:40		86	Yes	168	86	332	374	Normal	
Yes	2016-10-06	07:30		65	Yes	167	76	390	401	Normal	
Yes	2016-10-12	08:20		83	Yes	138	105	353	393	Normal	
Yes	2016-10-04	11:47		67	Yes	149	89	389	404	Normal	
Yes	2016-10-17	06:55		65	Yes	190	94	400	411	Normal	
Yes	2016-10-14	07:50		119	Yes	128	85	323	406	Normal	
Yes	2016-10-17	06:50		73	Yes	165	96	399	426	Normal	
Yes	2016-10-07	09:51		81	Yes	149	94	344	380	Normal	
Yes	2016-10-06	08:55		91	Yes	151	92	347		Abnormal	ventricular extrasistoly
Yes	2016-10-18	06:40		86	Yes	168	112	363	409	Normal	
Yes	2016-10-12	08:12		80	Yes	150	92	379	417	Normal	
Yes	2016-10-18	11:16		95	Yes	133	84	330		Normal	
Yes	2016-10-06	09:20		83	Yes	177	95	370		Normal	
Yes	2016-10-18	08:10		83	Yes	145	86	343	382	Normal	
Yes	2016-10-24	06:30		106	Yes	129	94	349	422	Normal	
Yes	2016-10-20	10:12		92	Yes	124	80	359	414	Normal	
Yes	2016-12-21	08:44		60	Yes	135	107	414	414	Normal	
Yes	2017-01-19	07:48		78	No	0	57	329	359	Abnormal	NCS longterm chronic atrial fibrillation without change compare to V1
Yes	2016-12-20	08:28		72	Yes	151	114	426		Normal	
Yes	2016-12-20	08:39		68	Yes	170	110	414		Normal	
Yes	2017-01-17	09:29		77	Yes	169	97	356		Normal	

Yes	2017-01-17	09:33		91	Yes	171	85	345		Normal	
Yes	2017-01-24	06:50		65	Yes	170	101	391	402	Normal	
Yes	2017-01-25	08:32		97	Yes	135	90	338	397	Normal	
Yes	2017-02-14	08:56		61	Yes	152	113	371		Normal	
Yes	2017-01-27	08:25		78	No	175	82	349	381	Normal	
Yes	2017-01-20	07:57		74	Yes	128	84	356		Normal	
Yes	2017-01-23	08:23		75	Yes	157	114	420	452	Normal	
Yes	2017-01-31	09:16		82	Yes	168	104	334	371	Normal	
Yes	2017-01-26	08:15		71	Yes	172	99	355	375	Normal	
Yes	2017-02-09	08:05		106	Yes	123	89	322	389	Normal	
Yes	2017-01-20	08:56		64	Yes	156	92	381		Normal	
Yes	2017-01-25	08:20		67	Yes	221	73	405		Normal	
Yes	2017-02-01	09:04		71	Yes	170	114	383		Normal	
Yes	2017-01-27	08:17		64	Yes	199	83	351		Normal	
Yes	2016-11-18	08:32		71	Yes	154	104	382	404	Normal	
Yes	2016-11-23	08:32		71	Yes	168	108	374	396	Normal	
Yes	2016-11-23	09:57		67	Yes	166	98	392	407	Normal	
Yes	2016-11-17	08:13		69	No	190	110	382	400	Normal	
Yes	2016-11-21	10:09		88	Yes	178	96	358	407	Normal	
Yes	2016-11-27	09:55		85	Yes	152	80	354	398	Normal	
Yes	2016-11-30	08:43		98	Yes	164	82	306	360	Normal	
Yes	2016-11-30	08:50		63	Yes	156	104	422	429	Normal	
Yes	2016-11-22	09:13		64	Yes	152	86	398	407	Normal	
Yes	2016-12-05	10:47		65	No	142	86	418	429	Normal	
Yes	2016-11-27	09:29		73	Yes	168	94	376	401	Normal	
Yes	2016-12-01	08:32		71	Yes	170	96	370	391	Normal	
Yes	2016-12-02	08:35		79	No	180	82	334	366	Normal	
Yes	2016-12-03	08:49		68	Yes	174	104	350	365	Normal	
Yes	2016-12-03	09:09		64	Yes	148	74	424	433	Normal	
Yes	2016-12-07	09:35		60	Yes	148	90	398	398	Normal	
Yes	2016-12-16	08:45		83	No	136	94	366	408	Normal	
Yes	2016-12-16	09:25		96	No	180	78	362	423	Normal	
Yes	2017-01-06	08:10		81	Yes	166	92	378	418	Normal	
Yes	2017-01-10	08:00		57	No	138	86	428	421	Normal	
Yes	2017-01-09	09:01		103	Yes	108	88	322	386	Normal	
Yes	2016-07-21	08:14		68	Yes	138	90	370	386	Normal	
Yes	2016-07-27	08:12		83	Yes	172	94	382	426	Normal	
Yes	2016-08-02	08:16		79	Yes	152	84	384	421	Normal	
Yes	2016-09-20	08:17		52	Yes	182	88	434	414	Normal	
Yes	2016-09-19	08:36		60	Yes	136	78	426	426	Normal	
Yes	2016-07-15	07:15		72	Yes	200	80	390	414	Normal	
Yes	2016-07-25	07:07		78	Yes	200	80	390	426	Normal	
Yes	2016-07-19	07:08		89	Yes	200	80	390		Normal	
Yes	2016-07-20	07:16		88	Yes	200	80	390	443	Normal	
Yes	2016-07-20	07:10		78	Yes	190	80	390		Normal	
Yes	2016-07-21	07:11		83	Yes	200	80	380		Normal	
Yes	2016-08-22	07:20		69	Yes	200	80	400		Normal	
Yes	2016-07-22	07:01		93	Yes	180	80	370	428	Normal	
Yes	2016-07-21	07:15		98	Yes	180	120	370	436	Abnormal	Complete right bundle branch block. NCS.
Yes	2016-07-22	07:10		72	Yes	200	80	390	414	Normal	
Yes	2016-08-22	07:00		81	Yes	200	80	380		Normal	
Yes	2016-07-21	07:16		83	Yes	200	80	390		Normal	
Yes	2016-07-22	07:15		83	Yes	200	80	380	423	Normal	
Yes	2016-07-21	07:11		80	Yes	200	80	390		Normal	
Yes	2016-08-22	07:18		67	Yes	200	80	400	415	Normal	
Yes	2016-08-24	07:10		70	Yes	190	80	400	421	Normal	
Yes	2016-08-22	07:14		56	Yes	200	80	400		Normal	
Yes	2016-08-23	07:10		82	Yes	200	80	400	444	Normal	
Yes	2016-08-23	07:15		65	Yes	200	80	400	411	Normal	
Yes	2016-08-25	07:09		102	Yes	200	80	370	442	Abnormal	Sinus tahycardia. NCS.
Yes	2016-08-22	07:22		86	Yes	200	80	380	428	Normal	
Yes	2016-09-14	07:20		67	Yes	200	80	400	415	Abnormal	Non significant negative T-wave in II,III,aVF-NCS
Yes	2016-08-26	07:14		69	Yes	200	80	400	419	Normal	
Yes	2016-08-26	07:09		68	Yes	190	80	400	417	Normal	
Yes	2016-09-15	07:12		67	Yes	210	120	410	425	Abnormal	complete right bundle branch block-NCS
Yes	2016-09-14	07:16		85	Yes	190	80	400	449	Normal	
Yes	2016-08-25	07:11		65	Yes	200	80	390	401	Normal	

Yes	2016-09-15	07:11		85	Yes	210	120	390	438	Abnormal	left position,left front hemiblock,complete right bundle branch blfck-NCS
Yes	2016-09-16	07:12		60	Yes	200	80	400	400	Normal	
Yes	2016-09-19	07:17		83	Yes	200	80	380	423	Normal	
Yes	2016-09-26	07:14		71	Yes	200	80	380	402	Normal	
Yes	2016-09-27	07:10		60	Yes	200	140	400	400	Abnormal	complete right bundle branch block-NCS
Yes	2016-09-28	07:10		77	Yes	200	80	390	424	Normal	
Yes	2016-09-27	07:23		82	Yes	190	80	400	444	Normal	
Yes	2016-09-26	07:20		66	Yes	190	80	400	413	Normal	
Yes	2016-09-27	07:09		85	Yes	180	80	370	416	Normal	
Yes	2016-09-27	07:28		77	Yes	190	80	380	413	Normal	
Yes	2016-09-21	07:23		62	Yes	200	80	390	394	Normal	
Yes	2016-09-21	07:20		66	Yes	200	80	400	413	Normal	
Yes	2016-09-28	07:14		81	Yes	190	80	390	431	Normal	
Yes	2016-09-29	07:10		78	Yes	200	80	400	437	Normal	
Yes	2016-10-12	07:10		86	Yes	190	80	390	440	Normal	
Yes	2016-10-04	07:25		88	Yes	200	80	360	409	Normal	
Yes	2016-09-27	07:15		73	Yes	200	80	380	406	Normal	
Yes	2016-09-29	07:15		79	Yes	180	80	390	427	Normal	
Yes	2016-09-30	07:10		65	Yes	190	80	400	411	Normal	
Yes	2016-10-11	07:12		81	Yes	200	100	390	431	Normal	
Yes	2016-10-11	07:10		84	Yes	200	80	370	414	Normal	
Yes	2016-11-24	07:16		68	Yes	180	80	400	417	Normal	
Yes	2016-11-24	07:05		70	Yes	190	80	380	400	Normal	
Yes	2016-11-25	07:11		94	Yes	180	80	370	430	Normal	
Yes	2016-11-25	07:07		65	Yes	200	80	400	411	Normal	
Yes	2016-12-01	07:15		85	Yes	200	80	380	427	Normal	
Yes	2016-12-01	07:10		85	Yes	180	80	380	427	Normal	
Yes	2016-12-09	07:21		71	Yes	210	80	380	402	Normal	
Yes	2016-12-05	07:11		97	Yes	200	80	360	423	Normal	
Yes	2016-12-20	07:11		77	Yes	190	90	400	435	Normal	
Yes	2016-12-21	07:05		72	Yes	200	80	390	414	Normal	
Yes	2016-12-09	07:15		81	Yes	190	80	400	442	Normal	
Yes	2016-12-07	07:11		88	Yes	190	90	370	420	Normal	
Yes	2016-12-19	07:11		88	Yes	180	80	380	432	Normal	
Yes	2016-12-02	07:10		84	Yes	180	80	390	436	Normal	
Yes	2016-12-09	07:30		75	Yes	210	80	390	420	Normal	
Yes	2016-12-02	07:18		83	Yes	200	80	400	446	Normal	
Yes	2016-12-08	07:11		80	Yes	200	80	380	418	Normal	
Yes	2016-12-05	07:13		61	Yes	200	80	410	412	Normal	
Yes	2016-12-07	07:10		79	Yes	200	80	380	416	Normal	
Yes	2017-01-16	07:10		76	Yes	190	110	400	433	Abnormal	incomplete right bundle branch block. NCS.
Yes	2017-01-16	07:05		67	Yes	200	80	390	405	Normal	
Yes	2017-01-17	07:22		89	Yes	200	80	380	433	Normal	
Yes	2017-01-17	07:14		85	Yes	190	80	380	427	Normal	
Yes	2017-01-17	07:20		68	Yes	210	80	380	432	Normal	
Yes	2017-01-16	07:00		83	Yes	200	80	390	435	Normal	
Yes	2016-11-23	07:49		75	Yes	242	119	371	400	Normal	
Yes	2016-12-16	08:08		91	Yes	154	96	337	387	Normal	
Yes	2017-01-19	10:01		56	Yes	139	108	379	370	Normal	

Visit 7-Visit Specific Assessments-Completion Status	Visit 7-Visit Specific Assessments-Date of ECG	Visit 7-Visit Specific Assessments-Time of ECG	Visit 7-Visit Specific Assessments-Reason Not Performed	Visit 7-Visit Specific Assessments-Heart Rate (beats/min)	Visit 7-Visit Specific Assessments-Sinusal Rhythm	Visit 7-Visit Specific Assessments-PR Interval (msec)	Visit 7-Visit Specific Assessments-QRS Interval (msec)	Visit 7-Visit Specific Assessments-QT Interval (msec)	Visit 7-Visit Specific Assessments-QTcF Interval (msec)	Visit 7-Visit Specific Assessments-Result or Finding in Original Units	Visit 7-Visit Specific Assessments-Cardiologist's Comments
V7.V7_ASS.EGPERF	V7.V7_ASS.EGDAT	V7.V7_ASS.EGTIM	V7.V7_ASS.EGREASND	V7.V7_ASS.ORRES_HR1	V7.V7_ASS.ORRES_SNRH	V7.V7_ASS.ORRES_PR	V7.V7_ASS.ORRES_QRS	V7.V7_ASS.ORRES_QT	V7.V7_ASS.ORRES_QTCF	V7.V7_ASS.EGORRES	V7.V7_ASS.EGCOVAL
Yes	2016-08-01	09:04		66	Yes	230	109	413		Abnormal	AVblok 1st degree, NCS
Yes	2016-07-27	06:55		65	Yes	168	90	405	416	Normal	
Yes	2016-08-02	09:28		64	Yes	146	104	367		Normal	
Yes	2016-07-27	07:50		74	Yes	172	90	377		Normal	
Yes	2016-07-28	08:25		71	Yes	161	89	376		Normal	
Yes	2016-08-04	11:45		62	Yes	162	97	427	432	Normal	
Yes	2016-07-28	09:15		68	Yes	159	113	365		Normal	
Yes	2016-07-28	07:20		59	Yes	231	103	437	435	Normal	
Yes	2016-08-02	07:25		79	Yes	174	114	389	426	Normal	
Yes	2016-07-27	08:45		89	Yes	164	101	358	408	Normal	
Yes	2016-08-01	09:46		88	Yes	128	86	357		Normal	
Yes	2016-08-05	10:11		85	Yes	187	113	363	408	Normal	
Yes	2016-07-29	08:57		83	Yes	151	94	385		Abnormal	ventricular extrasistola
Yes	2016-08-04	07:10		80	Yes	145	102	348	383	Normal	
Yes	2016-08-03	06:55		112	Yes	156	80	325	400	Normal	
Yes	2016-08-01	08:53		67	Yes	155	95	382		Normal	
Yes	2016-08-01	06:55		64	Yes	144	143	425	434	Normal	
Yes	2016-08-29	07:10		81	Yes	137	94	347	384	Normal	
Yes	2016-08-26	09:16		64	Yes	165	84	365	373	Normal	
Yes	2016-08-31	10:20		87	Yes	170	90	357	404	Normal	
Yes	2016-08-29	09:04		68	Yes	192	120	379	395	Normal	
Yes	2016-08-25	06:30		74	Yes	150	106	365	391	Normal	
Yes	2016-08-31	07:25		64	Yes	146	106	407	416	Normal	
Yes	2016-09-27	08:21		60	Yes	160	112	371		Normal	
Yes	2016-09-21	08:54		79	Yes	144	106	352		Normal	
Yes	2016-09-21	08:07		69	Yes	152	106	396		Abnormal	ventricular extrasistoles
Yes	2016-09-21	10:12		84	No	0	104	350		Abnormal	atrial fibrillation
Yes	2016-09-26	09:37		76	Yes	172	84	365		Normal	
Yes	2016-09-26	09:10		66	Yes	156	97	398	411	Normal	
Yes	2016-10-03	11:10		63	Yes	192	105	396	402	Normal	
Yes	2016-09-29	08:14		76	Yes	195	86	379	410	Normal	
Yes	2016-09-20	08:17		74	Yes	173	76	364		Normal	
Yes	2016-10-07	06:50		69	Yes	169	97	369	387	Normal	
Yes	2016-10-14	08:26		59	Yes	161	109	434	432	Normal	
Yes	2016-10-06	09:45		49	Yes	181	92	452	422	Normal	
Yes	2016-10-11	08:56		76	Yes	195	88	360	390	Normal	
Yes	2016-10-10	10:10		96	Yes	192	102	337	394	Normal	
Yes	2016-10-11	08:25		77	Yes	165	107	371	403	Normal	
Yes	2016-10-13	10:15		57	Yes	160	88	375	369	Normal	
Yes	2016-10-13	08:53		53	Yes	199	106	429	412	Normal	
Yes	2016-10-21	06:50		89	Yes	165	84	333	380	Normal	
Yes	2016-10-13	10:18		63	Yes	171	70	367	373	Normal	
Yes	2016-10-19	08:20		96	Yes	136	102	360	421	Normal	
Yes	2016-10-11	07:45		77	Yes	156	92	335	364	Normal	
Yes	2016-10-24	07:00		64	Yes	191	94	405	414	Normal	
Yes	2016-10-21	07:20		120	Yes	124	78	312	393	Normal	
Yes	2016-10-24	06:55		76	Yes	156	98	398	431	Normal	
Yes	2016-10-14	09:16		63	Yes	156	87	351	357	Normal	
Yes	2016-10-13	10:47		83	Yes	164	90	368		Abnormal	ventricular extrasystola
Yes	2016-10-25	06:45		107	Yes	144	106	354	429	Normal	
Yes	2016-10-19	08:38		75	Yes	169	89	356	383	Normal	
Yes	2016-10-25	09:57		94	Yes	144	95	325		Normal	
Yes	2016-10-19	09:44		91	Yes	156	93	353		Normal	
Yes	2016-10-25	09:50		72	Yes	157	86	372	395	Normal	
Yes	2016-10-31	09:20		92	Yes	135	102	349	402	Normal	
Yes	2016-10-27	08:56		65	Yes	122	77	357	367	Normal	
Yes	2016-12-28	11:58		58	Yes	133	110	435	430	Normal	
Yes	2017-01-26	09:05		83	No	0	86	340	379	Abnormal	NCS - longterm persistent atrial fibrillation without change compare to V1
Yes	2016-12-27	08:18		61	Yes	124	114	407		Normal	
Yes	2016-12-27	08:08		70	Yes	171	117	432		Normal	
Yes	2017-01-23	11:10		80	Yes	186	105	369		Normal	

Yes	2017-01-23	11:13		98	Yes	181	89	322		Normal	
Yes	2017-01-31	08:40		47	Yes	169	105	442	407	Normal	
Yes	2017-02-01	10:05		97	Yes	163	94	359	421	Normal	
Yes	2017-02-21	09:15		68	Yes	163	125	376		Normal	
Yes	2017-02-03	09:15		81	No	176	85	356	393	Normal	
Yes	2017-01-27	11:00		72	Yes	130	94	368		Normal	
Yes	2017-01-30	09:44		62	Yes	158	105	436	441	Normal	
Yes	2017-02-07	08:40		83	Yes	166	120	347	387	Normal	
Yes	2017-02-02	06:45		101	Yes	174	100	340	404	Normal	
Yes	2017-02-16	11:35		94	Yes	140	94	359	417	Normal	
Yes	2017-01-27	11:05		79	Yes	171	85	352		Normal	
Yes	2017-02-01	09:01		69	Yes	203	94	418		Normal	
Yes	2017-02-09	08:55		62	Yes	175	110	414		Normal	
Yes	2017-02-03	08:33		74	Yes	216	83	341		Normal	
Yes	2016-11-25	09:25		88	Yes	154	102	362	411	Normal	
Yes	2016-12-01	08:15		69	No	170	102	360	377	Normal	
Yes	2016-11-30	10:18		61	Yes	160	94	392	394	Normal	
Yes	2016-11-27	08:37		75	Yes	160	102	372	401	Normal	
Yes	2016-11-28	09:23		84	Yes	174	96	368	412	Normal	
Yes	2016-12-02	09:26		87	Yes	160	80	350	396	Normal	
Yes	2016-12-07	09:34		96	Yes	158	82	306	358	Normal	
Yes	2016-12-07	09:21		67	Yes	156	102	418	434	Normal	
Yes	2016-11-29	09:01		64	No	148	84	396	405	Normal	
Yes	2016-12-09	09:34		66	No	144	86	418	431	Normal	
Yes	2016-12-02	08:31		69	Yes	174	94	382	400	Normal	
Yes	2016-12-08	10:00		78	No	166	94	352	384	Normal	
Yes	2016-12-09	08:45		74	Yes	164	82	340	365	Normal	
Yes	2016-12-10	08:05		59	Yes	160	108	428	426	Normal	
Yes	2016-12-10	08:30		62	No	150	74	426	431	Normal	
Yes	2016-12-13	12:16		63	Yes	152	92	400	407	Normal	
Yes	2016-12-23	09:04		75	No	140	96	388	418	Normal	
Yes	2016-12-22	08:46		99	No	174	76	354	418	Normal	
Yes	2017-01-12	09:12		81	Yes	152	90	380	420	Normal	
Yes	2017-01-17	07:59		54	No	140	86	430	415	Normal	
Yes	2017-01-16	08:33		93	Yes	120	90	326	377	Normal	
Yes	2016-07-28	08:12		71	Yes	138	90	386	408	Normal	
Yes	2016-08-03	08:15		85	Yes	164	94	384	431	Normal	
Yes	2016-08-11	08:30		83	Yes	148	82	366	408	Normal	
Yes	2016-09-26	08:46		59	Yes	182	88	410	408	Normal	
Yes	2016-09-27	08:32		64	Yes	130	78	418	427	Normal	
Yes	2016-07-27	07:15		78	Yes	210	80	380	415	Normal	
Yes	2016-07-29	07:21		79	Yes	190	80	390	427	Normal	
Yes	2016-07-27	07:10		83	Yes	200	80	380	423	Normal	
Yes	2016-07-27	07:20		95	Yes	200	80	380	443	Normal	
Yes	2016-07-27	07:05		77	Yes	200	80	380	413	Normal	
Yes	2016-07-28	07:19		74	Yes	200	80	390		Normal	
Yes	2016-08-29	07:11		71	Yes	190	80	390	413	Normal	
Yes	2016-07-28	07:11		100	Yes	180	80	379	449	Normal	
Yes	2016-07-28	07:22		75	Yes	200	120	400	431	Abnormal	Complete right bundle branch block NCS
Yes	2016-07-28	07:16		83	Yes	200	80	380	423	Normal	
Yes	2016-08-29	07:06		81	Yes	200	80	380	420	Normal	
Yes	2016-07-28	07:07		80	Yes	200	80	390	429	Normal	
Yes	2016-08-01	08:00		94	Yes	200	80	370	430	Normal	
Yes	2016-07-28	07:07		78	Yes	200	80	390	426	Normal	
Yes	2016-08-29	07:04		61	Yes	190	80	400		Normal	
Yes	2016-08-30	07:20		67	Yes	200	80	400	415	Normal	
Yes	2016-08-30	07:10		70	Yes	200	80	400	421	Normal	
Yes	2016-08-30	07:12		89	Yes	180	80	390	445	Normal	
Yes	2016-08-29	07:14		71	Yes	200	80	380	402	Normal	
Yes	2016-08-31	07:15		92	Yes	190	80	380	438	Normal	
Yes	2016-08-29	07:21		81	Yes	200	80	390		Normal	
Yes	2016-09-21	07:15		65	Yes	190	80	400	411	Abnormal	Non significant negative T-wave in II,III,aVF-NCS
Yes	2016-09-03	07:00		70	Yes	200	80	400	421	Normal	
Yes	2016-09-03	07:11		63	Yes	200	80	400	407	Normal	
Yes	2016-09-21	07:37		75	Yes	200	120	400	431	Abnormal	complete right bundle branch block-NCS
Yes	2016-09-21	07:11		84	Yes	200	80	380	425	Normal	
Yes	2016-08-31	07:14		71	Yes	190	80	390	413	Normal	

Yes	2016-09-21	07:17		76	Yes	200	120	380	411	Abnormal	left position,left front hemiblock,complete right bundle branch blfck-NCS
Yes	2016-09-24	07:21		62	Yes	200	80	400	404	Normal	
Yes	2016-09-24	07:26		80	Yes	200	80	380	418	Normal	
Yes	2016-10-03	07:12		78	Yes	190	80	380	415	Normal	
Yes	2016-10-03	07:16		64	Yes	200	140	400	409	Abnormal	Complete right bundle branch block. NCS.
Yes	2016-10-03	07:11		77	Yes	200	80	390	424	Normal	
Yes	2016-10-03	07:14		75	Yes	200	80	390	420	Normal	
Yes	2016-10-03	07:11		69	Yes	200	80	380	398	Normal	
Yes	2016-10-04	07:17		87	Yes	200	80	380	430	Normal	
Yes	2016-10-03	07:23		111	Yes	200	80	360	442	Abnormal	Sinus tachicardya - NCS.
Yes	2016-10-01	07:20		61	Yes	200	80	400	402	Normal	
Yes	2016-10-01	07:15		51	Yes	210	80	420	398	Abnormal	Sinus bradycardia - NCS.
Yes	2016-10-05	07:09		86	Yes	200	80	380	428	Normal	
Yes	2016-10-06	07:12		117	Yes	200	80	350	437	Normal	
Yes	2016-10-18	07:10		93	Yes	200	80	370	428	Normal	
Yes	2016-10-11	07:15		94	Yes	200	80	380	441	Normal	
Yes	2016-10-05	07:42		81	Yes	190	80	380	420	Normal	
Yes	2016-10-05	07:20		78	Yes	180	80	380	415	Normal	
Yes	2016-10-07	07:20		85	Yes	200	80	400	449	Normal	
Yes	2016-10-18	07:21		81	Yes	200	100	390	431	Normal	
Yes	2016-10-18	07:11		79	Yes	200	80	380	416	Normal	
Yes	2016-12-02	07:08		77	Yes	180	80	400	435	Normal	
Yes	2016-12-02	07:10		69	Yes	190	80	380	398	Normal	
Yes	2016-12-02	07:15		98	Yes	180	80	370	436	Normal	
Yes	2016-12-02	07:20		69	Yes	200	80	400	419	Normal	
Yes	2016-12-08	07:14		73	Yes	200	80	380	406	Normal	
Yes	2016-12-08	07:20		87	Yes	180	80	380	430	Normal	
Yes	2016-12-16	07:19		72	Yes	210	80	400	425	Normal	
Yes	2016-12-12	07:10		73	Yes	200	80	400	427	Normal	
Yes	2016-12-28	07:15		70	Yes	190	80	380	400	Normal	
Yes	2016-12-28	09:01		68	Yes	190	80	380	396	Normal	
Yes	2016-12-16	07:10		84	Yes	190	80	400	447	Normal	
Yes	2016-12-14	07:10		80	Yes	200	80	390	429	Normal	
Yes	2016-12-27	07:12		88	Yes	190	80	380	432	Normal	
Yes	2016-12-09	07:05		77	Yes	190	80	380	413	Normal	
Yes	2016-12-16	08:52		83	Yes	210	80	380	423	Normal	
Yes	2016-12-09	07:02		63	Yes	200	80	400	407	Normal	
Yes	2016-12-15	07:10		83	Yes	200	90	380	423	Normal	
Yes	2016-12-12	07:04		74	Yes	200	80	400	429	Normal	
Yes	2016-12-13	07:15		76	Yes	210	80	390	422	Normal	
Yes	2017-01-23	07:20		76	Yes	190	110	400	433	Abnormal	Incomplete right bundle branch block. NCS.
Yes	2017-01-23	07:10		65	Yes	200	80	390	401	Normal	
Yes	2017-01-24	07:10		86	Yes	200	90	380	428	Normal	
Yes	2017-01-24	07:15		69	Yes	190	80	390	409	Normal	
Yes	2017-01-24	07:20		76	Yes	210	80	380	411	Normal	
Yes	2017-01-23	07:17		93	Yes	210	90	370	428	Normal	
Yes	2016-11-30	12:38		71	Yes	241	130	371	392	Normal	
Yes	2016-12-26	13:23		101	Yes	152	93	331	394	Normal	
Yes	2017-01-26	12:11		59	Yes	140	108	382	380	Normal	

HAROSA III : BF2.649/Placebo(P1513)

Meeting of the Safety Data Monitoring Committee

Meeting Minutes - Final

Attendees

[redacted] ospital, Lyon, France [redacted]
[redacted] int Antoine Hospital, Paris, France [redacted]
[redacted] Biostatistician, DICE, Brussels Belgium [redacted]
[redacted] projet, Paris, France [redacted]
[redacted] aris, France [redacted]
[redacted], France [redacted]

Author of the minutes [redacted]

Time and place

- **Date:** Wednesday 13 September 2017
- **Time:** 10:30 - 12:00
- **Location:** Telephone conference

Item	Discussion Topics / Minutes	Actions/Attachments
**Agenda**	▪ Safety review<br>▪ Recommendations<br>▪ Next meeting	
**Study status**	▪ **Patients recruitment status**<br>o 202 screened<br>o 187 randomized (102 with nCPAP, 85 without nCPAP)<br>o 184 completed double blind phase<br>o 175 patients entering OL phase<br>o Around 119 patients with 100% data clean<br>LPLV double blind: 27 February 2017<br>LPLV open label: 7 December 2017<br>Blind review of all data: after completion of open label phase	Two sites (41 and 53) have included 83% of patients randomized. This will be addressed in the SAP to assess potential interaction between site and treatment.
**Review of Safety Data**	TEAEs were reported for 29% of the patients, related TEAEs for 20%. One treatment-emergent SAE was reported (Non-Hodgkin lymphoma), considered not-related to the study treatment, and resulting in treatment discontinuation. There were no severe events.<br>o Tables and listings were reviewed<br>o AEs were reviewed regardless of data cleanness status<br>o ECGs data were provided for all patients	▪ Bioprojet to provide additional information on the following AEs:<br>o Renal crisis and sinus tachycardia: Pt 4133<br>o Absolute arrhythmia in atrial fibrillation: Pt 5378<br>o Single supraventricular extrasystoles: Pt 5348<br>o Phlegmona femoris dextra: Pt 5345
**Recommendation**	During a closed meeting it was decided that on the basis of the provided information no change to the study is recommended	
**Next meeting**	Next meeting	Since the study is almost terminated (database lock foreseen in December 2017) it was decided there would be no further meeting.

Study P1513 (HAROSA III) : BF2.649/Placebo

Meeting of the Safety Data Monitoring Committee

Meeting Minutes - Final

Attendees

[redacted], Bron Hospital, Lyon, France [redacted]
[redacted], Biostatistician, DICE, Brussels Belgium [redacted]
[redacted], Medical Director, Bioprojet, Paris, France [redacted]
[redacted], Senior CRA, Bioprojet, Paris, France [redacted]
[redacted], Head of Pharmacovigilance Department, Bioprojet, Paris, France [redacted]
[redacted], Clinical Project Manager, Bioprojet, Paris, France [redacted]

Author of the minutes

[redacted]

Time and place

- **Date:** Wednesday 6 February 2019
- **Time:** 10:00 - 12:00
- **Location:** Telephone conference

Item	Discussion Topics / Minutes	Actions/Attachments
**Agenda**	▪ Chair and attendance<br>▪ Conflict of Interest<br>▪ Safety review<br>▪ Recommendation<br>▪ Next meeting	
**Chair**	▪ It was reconfirmed that [redacted] would remain Head of the SDMC and that [redacted] would prepare the minutes.	
**Attendance**	▪ One of the members of the SDMC, [redacted], could not attend the meeting for health reasons.	
**Conflicts of Interest**	▪ On request of Bioprojet it was enquired whether the members of the SDMC had any conflict of interest. The 2 members present at the meeting [redacted] stated they had no conflict of interest.	
**Study status**	▪ **Patients recruitment status**<br>o 329 patients screened<br>o 300 randomized (102 with nCPAP, 85 without nCPAP)<br>o 220 completed double blind phase<br>o 18 patients did not enter the OL phase<br>o 170 completed OL phase<br>o 112 patients are ongoing<br>o LPFV foreseen for March 2019	
**Review of Safety Data**	▪ During the previous meeting additional information was requested concerning AEs in patients 4133, 5378, 5348, and 5345. This information was sent to the participants and reviewed during the meeting. For patients 4133, 5345, and 5348 no further action is required. For patient 5378 it was mentioned that the medical condition would be monitored.<br>Tables and listings were reviewed<br>▪ 109 TEAEs were reported for 68 of the 300 randomized patients (23%), among which 72 were considered related. Two events were considered severe (insomnia and anxiety reported by the same patient). Three SAEs were reported all considered unlikely related to the study medication:<br>- Two TESAEs, ‘Non-Hodgkin lymphoma’, and ‘hospitalization with a diagnosis of local bacterial infection of the skin’<br>- One SAE that occurred before randomization, ‘cholecystitis calculosa’.<br>▪ All ECGs considered abnormal and with QTc above 400 were reviewed.	▪ Bioprojet to provide additional information concerning further monitoring of patient 5378 following absolute arrhythmia in atrial fibrillation.<br>▪ No further action required<br>▪ It was requested that during the next meeting a list should be provided of all ECG data for patients with an abnormal ECG or any QTc of at least 450 msec, or elongation of at least 60 msec.
**Recommendation**	During a closed meeting it was decided that on the basis of the provided information no change to the study is recommended.	
**Next meeting**		Unless there is reason for concern the next meeting will take place in November or December 2019.
